# Synthesis of a Mechanically Planar Chiral Rotaxane Ligand for Enantioselective Catalysis

**DOI:** 10.1016/j.chempr.2020.02.006

**Published:** 2020-04-09

**Authors:** Andrew W. Heard, Stephen M. Goldup

**Affiliations:** 1 School of Chemistry, University of Southampton, Highfield, Southampton SO17 1BJ, UK

**Keywords:** SDG9: Industry, innovation, and infrastructure

## Abstract

Rotaxanes are interlocked molecules in which a molecular ring is trapped on a dumbbell-shaped axle because of its inability to escape over the bulky end groups, resulting in a so-called mechanical bond. Interlocked molecules have mainly been studied as components of molecular machines, but the crowded, flexible environment created by threading one molecule through another has also been explored in catalysis and sensing. However, so far, the applications of one of the most intriguing properties of interlocked molecules, their ability to display stereogenic units that do not rely on the stereochemistry of their covalent subunits, termed “mechanical chirality,” have yet to be properly explored, and prototypical demonstration of the applications of mechanically chiral rotaxanes remain scarce. Here, we describe a mechanically planar chiral rotaxane-based Au complex that mediates a cyclopropanation reaction with stereoselectivities that are comparable with the best conventional covalent catalyst reported for this reaction.

## Highlights

• We synthesized a mechanically planar chiral rotaxane for catalytically active gold ions
• We demonstrate enantioselective catalysis with a mechanically planar chiral rotaxane
• Our results suggest that mechanical stereochemistry has untapped potential in enantioselective catalysis

## The Bigger Picture

Molecules that exist in non-identical mirror image forms are referred to as chiral. Chirality can arise because of various molecular features in which atoms are held in fixed orientations that are themselves chiral, and typically such “stereogenic units” are maintained by direct bonds between atoms. Molecular chirality can also arise by threading a dumbbell-shaped molecule through a molecular ring to generate a rotaxane. However, these molecules have not been investigated significantly because until recently they were extremely hard to make in one mirror image form. Here, we report the first example of a catalyst based on such a “mechanically chiral” rotaxane. Catalysis with chiral molecules is extremely important in modern chemistry because it is one of the most efficient ways to make chiral molecules for applications in healthcare and other areas. Our results demonstrate that mechanically chiral molecules are a promising and underexplored platform for generating such catalysts.

## Graphical Abstract

**Figure:**
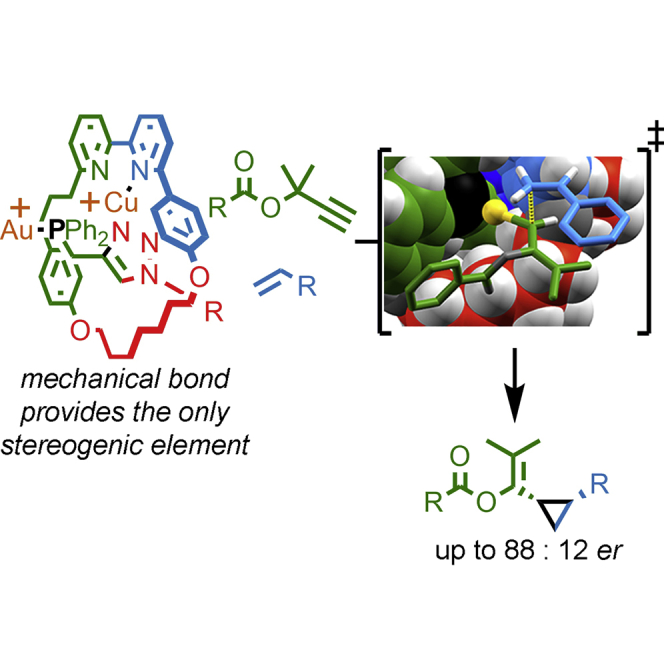

## Teaser

We report an enantioselective catalyst based on a “mechanically chiral” rotaxane. Catalysis with chiral molecules is extremely important in modern chemistry because it is one of the most efficient ways to make chiral molecules for applications in many areas. Our results demonstrate, for the first time, that mechanically chiral molecules are a promising and underexplored platform for generating such catalysts. We achieve enantioselectivities for the Au^I^-catalyzed Ohe-Uemura cyclopropanation of benzoate esters comparable to previously reported covalent catalysts.

## Introduction

**Figure 1:**
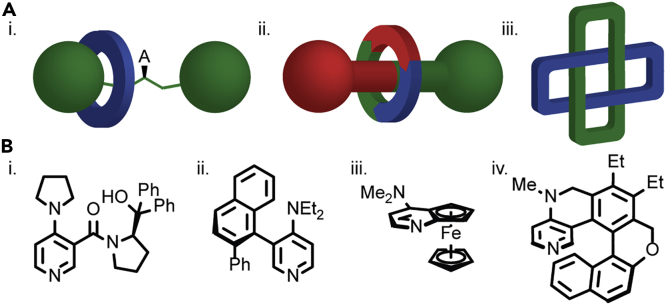
Different Forms of Chirality in Mechanically and Covalently Bonded Molecules (A) Examples of (i) co-conformational, (ii) conditional mechanical, and (iii) unconditional topological stereogenic units. (B) Examples of covalently bonded chiral acyl transfer catalysts based on (i) point,^11^ (ii) axial,^12^ (iii) planar,^13^ and (iv) helical^14^ stereogenic units.

Interlocked molecules such as rotaxanes, in which a dumbbell-shaped axle is threaded through a macrocycle, and catenanes, in which two or more macrocycles are held together in a manner akin to links in a chain,^1^ are most commonly investigated as components of molecular machines,^2^ building on the pioneering work of Stoddart and Sauvage, who were awarded the Nobel Prize for their efforts in 2016.^3^, ^4^, ^5^ In contrast, one of the most intriguing structural properties of interlocked molecules, their ability to display enantiotopic stereogenic elements that do not rely on covalent stereochemistry,^6^ has received much less attention, despite the possibility of such enantiomerism being discussed early in the development of the field.^7^^,^^8^ Such “mechanical” stereogenic units can arise because of desymmetrization of one of the covalent subunits by the relative position of the other (co-conformational chirality), the combination of subunits with appropriate symmetry properties (conditional mechanical chirality), or the unconditional topology of the mechanical bond itself (Figure 1A).^6^^,^^9^^,^^10^

**Scheme 1:**
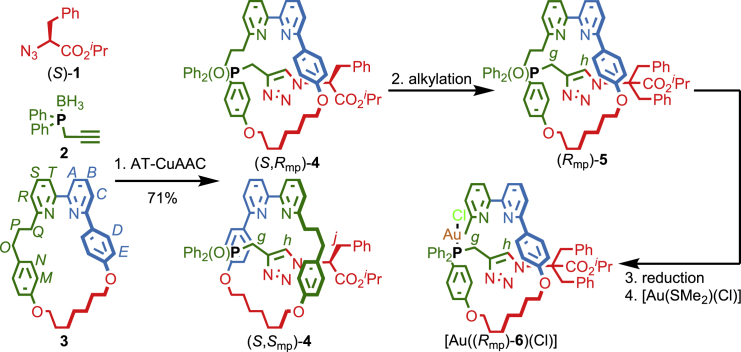
Synthesis of Mechanically Planar Chiral Rotaxane Precatalysts Reagents and conditions: (1) (i) [Cu(MeCN)_4_]PF_6_, ^1^H-sponge, CH_2_Cl_2_, room temperature (RT), 8 h; (ii) KCN, MeOH-CH_2_Cl_2_ (1:1), RT, 30 min; (iii) H_2_O_2_ (35% w/w in H_2_O), CH_2_Cl_2_, RT, 5 min. 72% combined yield over 3 steps prior to separation of diastereomers. (*S*,*R*_mp_)-**4**: 30%, 98% *ee*, >99: <1 *dr*; (*S*,*S*_mp_)-**4**: 24%, (*S*,*S*_mp_)-**4**-(*R*,*S*_mp_)-**4**-(*S*,*R*_mp_)-**4** = 98.4:1.0:0.6. (2) LiHMDS, tetrahydrofuran, −78°C then, BnI, −78°C to RT, 18 h. (*R*_mp_)-**5**: 81% (98% *ee*). (*S*_mp_)-**5** 63% (98% *ee*; data not shown, see Supplemental Information). (3) HSiCl_3_, NEt_3_, PhMe, CH_2_Cl_2_, 100°C, 3 days. (4) (Me_2_S)AuCl, CH_2_Cl_2_, RT, 1 h. (*R*_mp_)-**5**: 64% yield over two steps (98% *ee*). (*S*_mp_)-**6**: 62% (98% *ee*; data not shown, see Supplemental Information).

The relative paucity of even prototypical applications of mechanically chiral molecules is at least in part because enantiopure samples were historically hard to synthesize, with the pioneering work carried out by Vögtle, Okamoto, and Sauvage,^15^^,^^16^ requiring the use of chiral stationary phase high-performance liquid chromatography (HPLC) to separate the enantiomeric products from a racemic mixture. Using this approach, Vogtle and co-workers showed that mechanically planar chiral rotaxanes and topologically chiral catenanes displayed strong electronic circular dichroism (CD),^15^ Hirose and co-workers disclosed a mechanically planar chiral rotaxane that selectively binds and senses the enantiomers of small chiral molecules,^17^ and Takata and co-workers demonstrated that the mechanically planar chiral stereogenic unit can direct the helical twist of a poly-diacetylene material.^18^ More recently, Saito and co-workers demonstrated the separation of co-conformationally mechanically planar chiral rotaxanes and used the link between the rate of racemization and co-conformational motion to determine the energy barrier for shuttling^19^ and Credi and co-workers demonstrated a co-conformationally mechanically planar chiral molecule that shuttles between achiral and chiral states, the latter of which could be biased by the binding of a small chiral guest.^20^

However, of these unusual forms of stereochemistry, only co-conformational point chirality has been exploited in catalysis; in 2015, Leigh and co-workers demonstrated an enantioselective co-conformationally covalent point chiral organocatalyst (Figure 1Ai) that mediated enamine and iminium activation.^21^^,^^22^ (The same authors have developed molecular ratchets and motors based on this stereogenic element.^23^^,^^24^) In contrast, the full complement of covalent stereogenic units,^25^ including point,^11^ axial,^12^ planar,^13^ and helical^14^ chirotopic elements^26^ have been applied in the development of new scaffolds to mediate enantioselective processes (Figure 1B) since the Nobel Prize was awarded in 2001 to Noyori, Knowles, and Sharpless for their contributions to the development of enantioselective catalysis.^27^, ^28^, ^29^ Indeed, recent work has aimed at expanding the mechanisms by which stereochemical information is transferred to the reaction space including the use of chiral counterions,^30^ chiral-at-metal systems,^31^ helical artificial^32^ and natural^33^^,^^34^ polymers, chiral solvents,^35^ chiral capsules,^36^ and other confined environments.^37^

Building on our recent effort to improve access to mechanically chiral molecules through the use of chiral derivatizing units^38^^,^^39^ and auxiliaries,^40^ here we demonstrate the first example of enantioselective catalysis with a mechanically planar chiral rotaxane, one of the simplest conditional mechanical stereogenic units, which arises when an achiral macrocycle with C_nh_ point group symmetry encircles an achiral axle with C_nv_ point group symmetry.^6^^,^^9^^,^^10^ Our rotaxane catalyst, whose structure was not designed or optimized, displays enantioselectivities in an Au^I^-mediated cyclopropanation reaction comparable to the best reported covalent catalyst.^41^ Our results suggest that mechanical stereochemistry has untapped potential in the development of new enantioselective catalytic systems.

## Results and Discussion

### Synthesis and Characterization of Mechanically Planar Chiral Complex [Au(6)(Cl)]

To demonstrate the potential of mechanical stereochemistry in catalysis, we selected a Au^I^-mediated reaction for our study. Au^I^-mediated reactions are inherently difficult to render enantioselective as a result of the linear coordination chemistry of the metal ion.^42^ These challenges are typically overcome through the use of large, monodentate ligands that project substituents into the reaction space or di-Au^I^ complexes, in which aurophilic interactions pre-organize the complex with one metal ion playing the role of the catalyst and the other of a structural unit,^42^^,^^43^ although employing secondary interactions in bifunctional catalysts is a promising emerging strategy.^44^, ^45^, ^46^ Given that we have previously shown that the mechanical bond can be used to project steric bulk around an Au^I^ center, leading to highly diastereoselective catalysis,^47^ we proposed that similar effects might be observed in the case of a mechanically chiral derivative, leading to enantioselective catalysis.

Rotaxane Au^I^ complex [Au(**6**)(Cl)] was synthesized using our small macrocycle modification^48^ of Leigh’s active template^49^ Cu-mediated alkyne-azide cycloaddition reaction (AT-CuAAC),^50^^,^^51^ employing amino-acid-derived azide **1** as a stereo-differentiating unit,^39^ borane-protected propargylic phosphine **2** as the alkyne coupling partner, and readily available C_1h_ (C_s_) symmetric macrocycle **3**,^52^ as the key mechanical bond forming step. We typically carry out the AT-CuAAC reaction in the presence of excess N^*i*^Pr_2_Et, which accelerates the reaction by favoring the formation of the key macrocycle-Cu^I^-acetylide complex intermediate. However, in this case, N^*i*^Pr_2_Et was found to cause epimerization of the azide stereocenter, resulting in a mixture of all four possible stereoisomeric products. Replacing N^*i*^Pr_2_Et with Proton Sponge drastically reduced the epimerization side reaction, allowing the mixture of diastereomeric phosphine oxides **4** to be separated^53^ with excellent stereochemical purity after demetallation and oxidative work-up. Using this sequence, we were able to isolate rotaxanes (*S*,*R*_mp_)-**4** (98% *ee*, >99: <1 *dr*) and (*S*,*S*_mp_)-**4** ((*S*,*S*_mp_)-**4**-(*R*,*S*_mp_)-**4**-(*S*,*R*_mp_)-**4** = 98.4:1.0:0.6, i.e., >98% *ee* in the mechanical stereogenic unit) in an acceptable combined yield of 54%. Alkylation of diastereomer (*S*,*R*_mp_)-**4** with BnI erased the covalent stereogenic unit to produce rotaxane (*R*_mp_)-**5**, in which the mechanical bond provides the sole stereogenic unit in excellent yield and enantiopurity (81% and 98% *ee*). Subsequent reduction of the phosphine oxide moiety and coordination of AuCl produced the Au^I^ precatalyst [Au((*R*_mp_)−**6**)(Cl)], the enantiopurity of which was assumed to be the same as that of (*R*_mp_)-**5** (98% *ee*) as the mechanical bond is configurationally stable. The same procedures starting from (*S*,*S*_mp_)-**4** produced [Au((*S*_mp_)−**6**)(Cl)] (98% *ee*).

**Figure 2:**
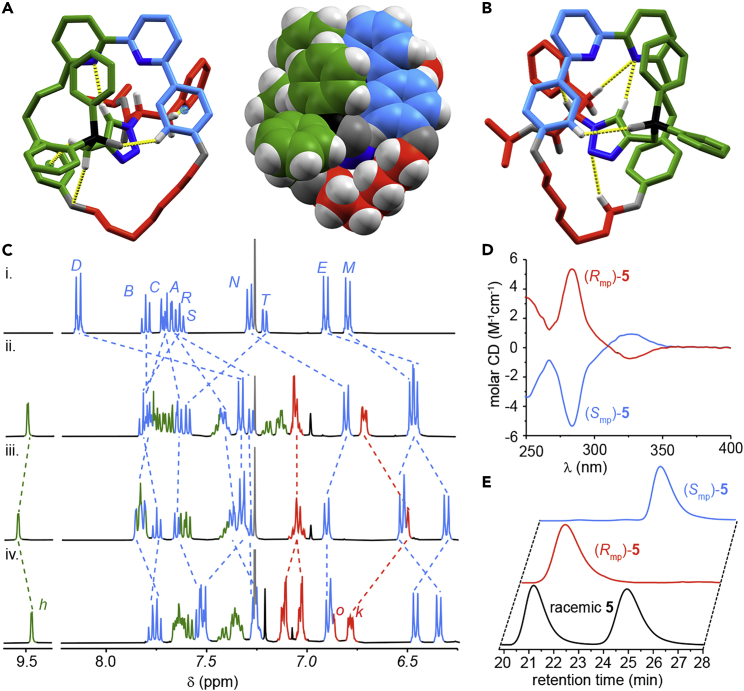
Characterization of Rotaxanes **4** and **5** (A) Solid-state structure of (*S*,*R*_mp_)-**4** with selected intercomponent interactions highlighted (atom labels and colors [O, dark gray; N, dark blue] as in Scheme 1, selected distances [Å]: H_*g*_•••O = 2.4, H_*g*_•••centroid = 2.6, H_*h*_•••N = 2.5, H_*j*_•••centroid = 3.2, and H_*E*_•••O = 2.5). (B) Solid-state structure of (*S*,*S*_mp_)-**4** with selected intercomponent interactions (atom labels and colors [O, dark gray; N, dark blue] as in Scheme 1, selected distances [Å]: H_*h*_•••N = 2.4, H_*i*_•••C = 2.6, H_*j*_•••N = 2.7, and H_*E*_•••O = 2.7). It should be noted that the asymmetric unit contains an oxidized derivative of (*S*,*S*_mp_)-**4** as a disordered impurity.^54^ The figure depicts the component of the unit cell that is unaffected by this disorder. (C) Partial ^1^H NMR (CDCl_3_, 400 MHz, 298 K) of (i) macrocycle **3**, (ii) rotaxane (*S*,*R*_mp_)-**4**, (iii) rotaxane (*S*,*S*_mp_)-**4**, and (iv) rotaxane (*R*_mp_)-**5**. Selected signals are assigned and color coded (see Scheme 1 for labels; H_*k*_ and H_*o*_, assigned arbitrarily, are the *ortho* protons of the diastereotopic axle benzyl groups). Signals corresponding to macrocycle **3** are all shown in blue for clarity.

Rotaxanes **4**, **5**, and [Au(**6**)(Cl)] were isolated and characterized in full by NMR, mass spectrometry (MS), HPLC (**4** and **5**), and CD (see Supplemental Information for full details). The absolute stereochemistries of phosphine oxides (*S*,*R*_mp_)-**4** and (*S*,*S*_mp_)-**4**^54^ were assigned by single-crystal X-ray diffraction (SC-XRD)^55^ (Figures 2A and 2B); the internal stereochemical reference provided by the azide-derived unit allowed the orientation of the macrocycle to be determined unambiguously and the stereochemical labels were assigned using our established approach (see Supplemental Information for details).^6^^,^^9^^,^^10^ The absolute stereochemistry of rotaxanes **5** and [Au(**6**)(Cl)] were inferred by noting that the mechanical stereochemistry of the corresponding diastereomeric starting materials cannot be altered in subsequent reactions.

The ^1^H NMR spectra of diastereomers (*S*,*R*_mp_)-**4** and (*S*,*S*_mp_)-**4** (Figures 2Cii and 2Ciii, respectively) display the typical features of such interlocked molecules;^48^ many of the signals corresponding to the axle and macrocycle components, including H_*D*_, H_*E*_, H_*M*_, and H_*N*_ are shielded relative to the non-interlocked macrocycle (Figure 2Ci), and triazole proton H_*h*_ appears at a high chemical shift due to the formation of an intercomponent C–H•••N hydrogen bond with the bipyridine, as observed in the solid-state structures (Figures 2A and 2B). However, their ^1^H NMR spectra are clearly distinct, in keeping with the diastereomeric relationship between the two products, as are their CD spectra (see Supplemental Information). Alkylation of rotaxanes **4** to give rotaxanes **5**, produced materials with identical ^1^H NMR spectra (Figure 2Biv) but mirror image CD spectra (Figure 2D), in keeping with the enantiomeric relationship between these products. Strikingly, in addition to the expected shielding/deshielding of signals, the aromatic protons corresponding to the diastereotopic benzylic units of the axle in rotaxanes **5** are clearly distinct (e.g., benzylic *ortho* protons H_*k*_ and H_*o*_), suggesting that the stereochemistry of the mechanical bond is well expressed onto the axle.

### Enantioselective Cyclopropanation Reactions Mediated by Rotaxane [Au((*R*_mp_)−**6**)(Cl)]

With the precatalyst [Au((*R*_mp_)−**6**)(Cl)] in hand, we investigated its behavior in the enantioselective Au^I^-mediated variant of the Ohe-Uemura^56^ cyclopropanation of alkenes by propargylic esters originally reported by Toste and co-workers using (*R*)-DTBM-SEGPHOS(AuCl)_2_ and resulting in stereoselectivities from 60% to 94% *ee*.^41^ More recently, Fuerstner and co-workers reported a mono-dentate 1,1-bi-2-naphthol-derived phosphoramite ligand for the same reaction,^57^ and Toste and co-workers reported a reaction system that employs Au nanoclusters embedded in a chiral self-assembled monolayer.^58^

Under conditions previously optimized for an analogous achiral rotaxane-based catalyst,^47^ [Au((*R*_mp_)−**6**)(Cl)] mediated the reaction of benzoyl ester **7** with styrene (**8**) to produce cyclopropanes **9** in excellent selectivity for the *cis* diastereomer (Table 1, entry 1). The role of the Cu^I^ additive is to bind to the bipyridine moiety, preventing the Lewis base inhibition of the Au^I^ center; reactions in the absence of Cu^I^ were unsuccessful (entry 2).^47^ Other cationic additives failed to activate the catalyst (see Supplemental Information). Analysis of the purified major diastereomer by chiral stationary phase HPLC revealed reasonable enantioselectivity for (1*S*,2*R*)-**9** (*er* = 72:28).^59^ As expected, replacing [Au((*R*_mp_)−**6**)(Cl)] with [Au((*S*_mp_)−**6**)(Cl)] produced **9** with opposite enantioselectivity (entry 3). Variation of the solvent led to changes in the observed *er* of *cis*-**9** but no significant improvement (entries 4–7). Cooling the reaction to 0°C improved the *er* of the major diastereomer to 79:21 (entry 8). Cooling the reaction mixture further led to no significant improvement and slowed the process considerably (entry 9). For comparison, the same reaction mediated by (*R*)-DTBM-SEGPHOS(AuCl)_2_ reported by Toste and co-workers produces cyclopropanes **9** in moderately higher and opposite stereoselectivity (entry 10).

**Table 1:** Optimization of an Enantioselective Cyclopropanation Reaction Mediated by [Au(6)(Cl)]^a^


Entry^a^	Solvent	Temperature (°C)	Time (h)	*cis*:*trans*^b^	*erc*_*is*_^c^	*ert*_*rans*_^c^
1	CDCl_3_	25	1	95:5	72:28	58:42
2^d^	CDCl_3_	25	1	n.r.	–	–
3^e^	CDCl_3_	25	1	95:5	29:71	42:58
4	MeNO_2_	25	1	87:13	53:47	65:35
5	CD_2_Cl_2_	25	1	83:17	64:36	66:34
6	CCl_4_	25	1	86:14	71:29	58:42
7	PhMe	25	1	85:15	69:31	56:44
8^f^	CDCl_3_	0	6	94:6	79:21	62:38
9	CDCl_3_	−35	24	96:4	79:21	61:39
10^g^	MeNO_2_	25	0.5	>20:<1	16:84	–

a [Au(**6**)(Cl)] with 84% *ee* was used for screening experiments unless otherwise stated.

b Determined by ^1^H NMR analysis of the crude reaction product using C_2_Cl_4_H_2_ as an internal standard (yield determination).

c Determined by HPLC.

d Reaction was conducted without the Cu^I^ additive.

e Reaction conducted with [Au((*S*_mp_)−6)(Cl)].

f Reaction conducted with [Au(**6**)(Cl)] with *er* = 99:1 stereopurity.

g Reaction outcome reported by Toste and co-workers for (*R*)-DTBM-SEGPHOS(AuCl)_2_.^41^

**Figure 3:**
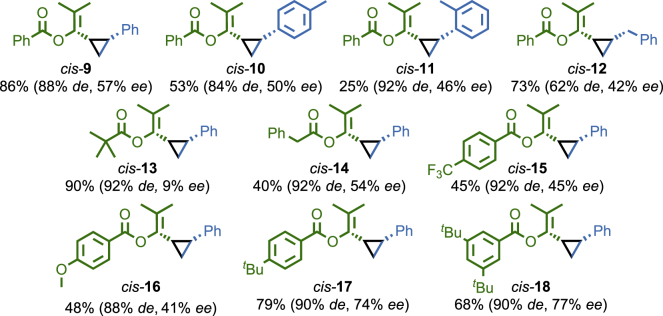
Cyclopropane Products Synthesized Using [Au((*R*_mp_)−**6**)(Cl)] All reactions carried out under the conditions shown in Table 1, entry 8. Combined yields of cyclopropanes and *de* were determined by ^1^H NMR analysis of the crude reaction product using C_2_Cl_4_H_2_ as an internal standard. *ee* of the major *cis* diastereomer determined by HPLC analysis of purified samples.^59^

With suitable conditions in hand (Table 1, entry 8), we performed a brief investigation of the effect of substrate on the stereoselectivity of reactions mediated by [Au((*R*_mp_)−**6**)(Cl)] (Figure 3). Variation of the styrene component in the reaction of benzoate ester **7** gave cyclopropanes **10** and **11** in similar *ee* and *de* to **9**, although the yield of the reaction was much lower in the case of 2-Me-substituted product **11**. Replacing styrene with allyl benzene gave cyclopropane **12** in reasonable enantioselectivity but lower diastereoselectivity, as has previously been observed for aliphatic alkenes.^41^ Conversely, variation of the propargylic ester component had a significant effect on the reaction stereoselectivity. Whereas (*R*)-DTBM-SEGPHOS(AuCl)_2_ is reported to deliver higher stereoselectivity with the pivaloyl derivative of propargyl ester **7**, in the case of [Au((*R*_mp_)−**6**)(Cl)], cyclopropane **13** was produced with almost no enantioselectivity. Pleasingly, phenylacetate ester-derived cyclopropane **14** was produced in comparable selectivity to **9**, confirming that α-alkyl esters are tolerated by [Au(**6**)(Cl)] and suggesting that the steric bulk of the pivolyl moiety is responsible for the loss of stereoselectivity in the case of **13**. Variation of the benzoyl moiety to introduce strongly electron-withdrawing or -donating groups (cyclopropanes **15** and **16**, respectively) led to a reduction in reaction enantioselectivity. In contrast, bulky alkyl groups on the benzoate moiety increased the reaction *ee*; *p*-^*t*^Bu benzoyl cyclopropane **17** and 3,5-di-^*t*^Bu-substituted cyclopropane **18** were produced in good yield and enantioselectivity. Cyclopropanes **9**–**18** were isolated by flash chromatography prior to HPLC analysis; the catalyst and any associated decomposition products were readily removed from the product mixture.

### Modeling of the Au^I^-Mediated Cyclopropanation of Styrene

Detailed modeling of interlocked molecules is challenging, given both their size and flexibility. Previously, the catalytic behavior of an interlocked catenane organocatalyst was studied computationally by considering the catalytic fragment alone on the assumption that the rest of the structure did not play a direct role in the reaction.^60^ In the case of [Au(**6**)(Cl)], this clearly would not be a reasonable assumption as the mechanical bond is the sole source of stereochemistry. Also, the implied difference in activation barrier, even for the most selective example reported above (**18**) is only ∼4.5 kJmol^−1^, a relatively small value for such a complex system where multiple conformations of the catalyst may be mechanistically relevant. These caveats notwithstanding, in order to gain some qualitative insight into how interactions between the reacting substrates and the rotaxane structure might influence the stereoselectivity of the reaction, we conducted preliminary computational modeling of the reaction of propargylic ester **7** and styrene (**8**) mediated by [Au(*R*_mp_-**6**)(Cl)].

**Figure 4:**
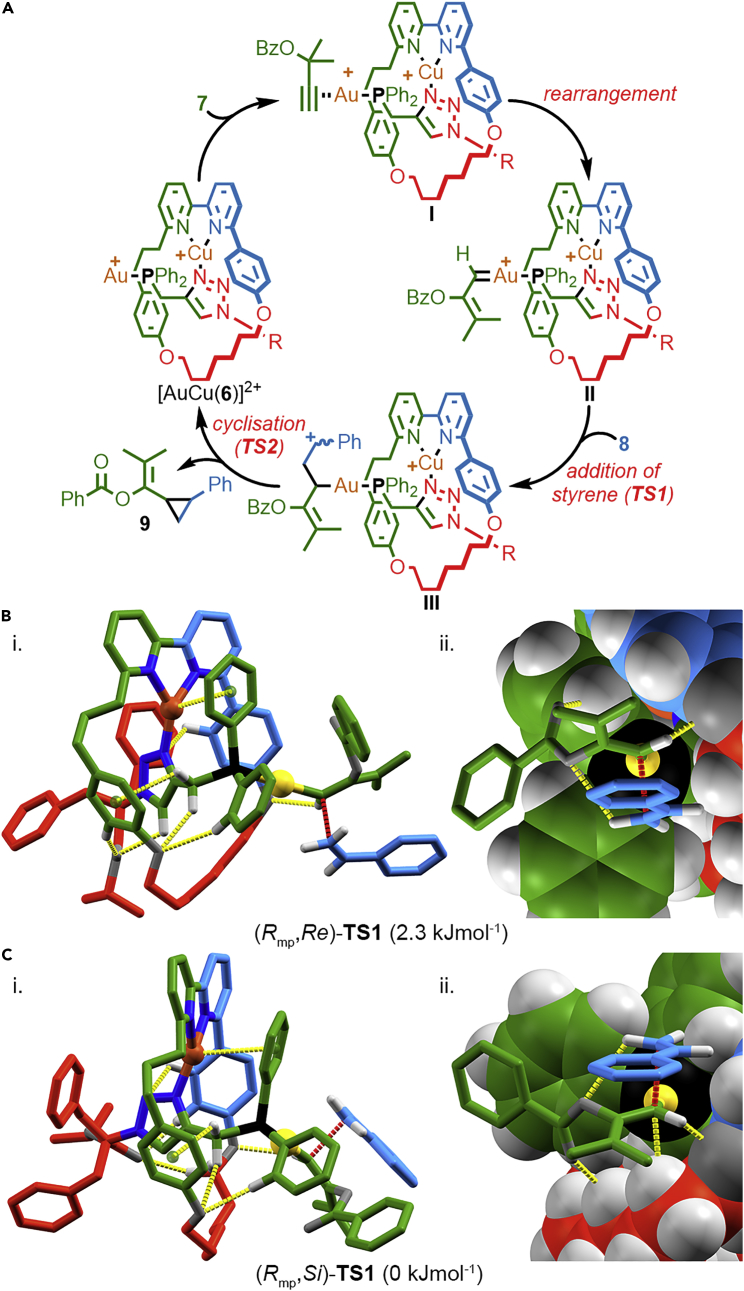
Reaction Pathway and Modeled Transition State Structures (A) Reaction pathway presumed for the reaction of [Au(**6**)(Cl)] based on molecular modeling (Gaussian ‘09, CAM-B3LYP, 6-31G∗/SDD(Au)) of the reaction of **7** and **8** mediated by [Au(PPh_3_)(Cl)]. R = C(Bn)_2_CO_2_^*i*^Pr. (B) Modeled (CHCl_3_, CAM-B3LYP, 6-31G/SDD) structure of **TS1** leading to (1*R*,2*S*)-**9** for the reaction of **7** with **8** mediated by [AuCu((*R*_mp_)-**6**)]^2+^ in (i) sticks representation and (ii) close-up of the transition state fragment in mixed space-filling and sticks representation. Selected intercomponent interactions and the carbene-styrene interaction associated with the reaction coordinate are highlighted in yellow and red, respectively. (C) Modeled (CHCl_3_, CAM-B3LYP, 6-31G/SDD) structure of **TS1** leading to (1*S*,2*R*)-**9** for the reaction of **7** with **8** mediated by [AuCu((*R*_mp_)-**6**)]^2+^ in (i) sticks representation and (ii) close-up of the transition state fragment in mixed space-filling and sticks representation. Selected intercomponent interactions and the carbene-styrene interaction associated with the reaction coordinate are highlighted in yellow and red, respectively.

In brief (for full details see Supplemental Information), we began by locating the lowest energy transition state (CAM-B3LYP/6-31G∗/SDD(Au)) for the reaction of **7** with **8** mediated by [Au(PPh_3_)(Cl)], building on previous work by Echavarren and co-workers.^61^ In keeping with this previous report, the reaction of the carbene derived from **7** with **8** was found to be a two-step process. We thus assumed a similar pathway for the reaction mediated by [Au(**6**)(Cl)] (Figure 4A); coordination of Cu^I^ and abstraction of the Cl ligand gives rise to the proposed active catalyst [AuCu(**6**)]^2+^, which coordinates to alkyne **7** to give complex **I** that undergoes a rearrangement to produce key carbene intermediate **II**. Addition of styrene to **II** produces carbocation **III** via key transition state **TSI**, in the process setting the stereochemistry of C^1^ of the cyclopropane product. Subsequent rapid ring closure gives rise to cyclopropane **9** and regenerates the catalyst.

In order to investigate the reaction mediated by rotaxane [Au(**6**)(Cl)], the transition state model found for the reaction mediated by [Au(PPh_3_)(Cl)] was modified by attachment to the Cu^I^-coordinated metallorotaxane.^62^, ^63^, ^64^ A conformational search (Spartan '10, MMF)^65^ with the transition state fragment frozen yielded low-energy conformers for each diastereomeric complex, which were optimized using density functional theory (DFT) (Gaussian ‘09,^66^ CAM-B3LYP, 6–31G/SDD(Cu,Au)), again with the transition state fragment frozen, to identify the lowest energy conformation. Transition state optimization, first using an ONIOM method (CAM-B3LYP:UFF, 6–31G/SDD(Au)), followed by a full DFT optimization (CAM-B3LYP, 631G/SDD(Cu,Au)) first in the gas phase then in solvent (CHCl_3_, polarizable continuum model) yielded transition state models (*R*_mp_,*Re*)-**TS1** and (*R*_mp_,*Si*)-**TS1** (Figures 4B and 4C, respectively) that were determined to be first order saddle points with a single imaginary frequency.

Examining the models of (*R*_mp_,*Re*)-**TS1** and (*R*_mp_,*Si*)-**TS1** (Figures 4B and 4C, respectively) reveals that, in spite of their size and large number of rotatable bonds, the modeled catalyst structure is actually relatively rigid because of steric crowding combined with the coordination of the Cu^I^ ion. A complex network of short intra- and intercomponent contacts including CH hydrogen bonds, CH-π interactions, and cation-π interaction between the Cu^I^ ion and one of the Ph rings of the phosphine ligand are predicted to stabilize the system further and project the Au^I^ center bearing the reactive carbene moiety toward the macrocycle, into the space around one of the phenoxy ether moieties. It is perhaps noteworthy that the optimized structures are similar to the solid-state structures of rotaxanes **4** determined by X-ray diffraction in which the phosphine substituent (O) is also projected toward the same aryl ether moiety. Crowding around the Au^I^ carbene moiety due to the mechanical bond is clearly seen in the space-filling models of (*R*_mp_,*Re*)-**TS1** and (*R*_mp_,*Si*)-**TS1** (Figures 4Bii and 4Cii, respectively); the macrocycle provides a sterically crowded environment that shields one face of the carbene unit and restricts the rotation of the substrate around the Au-P axis. The substrates are stabilized in the rotaxane environment through a number of non-covalent interactions, in particular a C(carbene)H–O interaction in both structures and a CH-C(carbene) interaction in the case of (*R*_mp_,*Si*)-**TS1**. Thus, the modeling suggests that a mechanically bonded structure provides a well-expressed chiral environment for the catalysis to take place within, which is consistent with the reasonable enantioselectivities achieved experimentally.

Finally, comparison of the calculated relative energies of (*R*_mp_,*Re*)-**TS1** and (*R*_mp_,*Si*)-**TS1** revealed remarkable agreement, given the size of the system, between experiment and theory; (*R*_mp_,*Si*)-**TS1** was found to be favored by ∼2.3 kJmol^−1^, corresponding to a stereoselectivity of 74:26 in favor of the major observed product (1*S*,2*R*)-**9**. However, caution should be taken when interpreting this level of agreement; modeling in the gas phase (6–31G/SDD) predicted the opposite stereoselectivity ((*R*_mp_,*Re*)-**TS1** favored by ∼1.7 kJmol^−1^). Conversely, re-optimization of **TS1** with the larger 6–31G∗ basis set in the gas phase or in CHCl_3_ (single point calculation)^67^ resulted in a predicted selectivity for the correct diastereomer that exceeds what is observed experimentally, demonstrating the uncertainty in the absolute values generated in such complex systems. Furthermore, although extending the modeling to the reactions leading to cyclopropanes **15** and **16** revealed reasonable agreement with experiment, the same calculations for the reaction leading to cyclopropanes **13** predicted a high selectivity, in contrast to the low selectivity observed experimentally (see Supplemental Information for details).

Thus, the molecular models of (*R*_mp_,*Re*)-**TS1** and (*R*_mp_,*Si*)-**TS1** should be considered qualitative, providing some insight into the potential interactions and a pictorial representation of the chiral environment created by the mechanical bond around the reacting Au^I^ carbene. A more detailed study, combined with many more comparisons between experiment and theory, would be required to determine the details of the key intermolecular interactions that lead to the observed stereoselectivity.

### Conclusions

Although the first enantiopure mechanically planar chiral rotaxane was reported over two decades ago,^15^ this is, to our knowledge, the first time that this stereogenic unit has been applied in catalysis. The results presented clearly demonstrate that the mechanically planar chiral stereogenic unit can direct enantioselective catalysis. The results are particularly pleasing given that rotaxane **6** was not explicitly designed or optimized for the reaction presented and yet achieves stereoselectivities with benzoate esters of 42%–77% *ee*, comparable to a similar reaction mediated by optimized covalent catalyst (*R*)-DTBM-SEGPHOS(AuCl)_2_ (68% *ee*). By extension, our results suggest that other mechanical stereogenic units^6^ such as the axial and topological chiral units in catenanes have unexplored potential in catalytic applications.

However, the stereoselectivities observed in this cyclopropanation reaction are lower than those reported when pivloate esters, which are not tolerated by [Au(**6**)(Cl)], were employed with the best covalent catalysts (76% to 94% *ee*),^41^ clearly demonstrating that challenges remain to be overcome for mechanically chiral rotaxanes to become useful tools in organic synthesis. It should also be noted that preliminary attempts to apply [Au(**6**)(Cl)] to other Au^I^-mediated reactions were unsuccessful (see Supplemental Information), suggesting that our success in this one reaction is serendipitous rather than an indication that the mechanically planar chiral stereogenic unit is somehow a “magic bullet” for enantioselective gold catalysis. Indeed, this is consistent with results with covalent catalysts (e.g., (*R*)-DTBM-SEGPHOS(AuCl)_2_—see Supplemental Information) that have been optimized for one Au^I^-mediated reaction but often perform poorly in others.^45^ Furthermore, despite recent progress in the area,^38^, ^39^, ^40^ the synthesis of mechanically interlocked molecules is still challenging, in the example presented, specifically due to the low stereoselectivity observed in the mechanical bond forming step and epimerization of the stereodirecting unit derived from the α-chiral azide that complicates the purification. This synthetic challenge clearly complicates the optimization of catalyst frameworks to deliver enhanced enantioselectivity. However, recent progress in the development of new methodologies to access enantiopure mechanically chiral molecules suggests that this synthetic challenge can and is being addressed, and pleasingly, based on the preliminary molecular modeling presented, it seems that modern computational chemistry may well be able to aid the design process.

Thus, in the future, we see a place for mechanical chirality in catalysis, particularly where it is otherwise challenging to project chiral information into the reaction space, as in the Au^I^-mediated reaction presented here; the crowded, three-dimensional^68^^,^^69^ nature of the mechanical bond appears to be well suited to generating a chiral pocket for chemical reactions to take place within, similar in some ways to enzymatic active sites with their combination of steric hindrance and weak attractive interactions with the substrate. Furthermore, combining chiral mechanical stereogenic units with the well-developed chemistry of interlocked molecular shuttles^2^^,^^70^, ^71^, ^72^, ^73^ should allow the influence of the stereogenic mechanical bond to be modulated^74^ in a stimuli-responsive manner in order to develop switchable chiral catalysts, for instance, to produce both hands of a given chiral product in high enantioselectivity^75^^,^^76^ Indeed, during the preparation of this manuscript, this principle was demonstrated in the context of co-conformational covalent point chirality.^77^ The same principles may also hold in the development of enantioselective sensors for chiral molecules. What is clear, based on these results, is that the chemical applications^78^^,^^79^ of mechanically chiral interlocked molecules deserve further investigation.

## Data and Code Availability

The accession numbers for the solid-state structures of (*S*,*R*_mp_)-**4** and (*S*,*S*_mp_)-**4** reported in this paper are CCDC: 1950723, 1980985, respectively. Processed compound characterization data are available freely from the University of Southampton repository (https://doi.org/10.5258/SOTON/D1223).

## Supplemental Information

Document S1. Schemes S1–S6, Figures S1–S185, Tables S1–S18, Supplemental Experimental Procedures, and Supplemental References

Data S1. Model_AuPPh3Cl_Full_Reaction.cif

Data S2. Model_AuPPh3Cl_TS1.cif

Data S3. Model_Rmp_Confs_I.cif

Data S4. Model_Table_S17_Structures.cif

Data S5. Model_Table_S18_Structures.cif

Document S2. Article plus Supplemental Information
