# Supplementary material for: Synthesis of a Mechanically Planar Chiral Rotaxane Ligand for Enantioselective Catalysis
Source: Chem. 2020 Apr 9;6(4):994–1006. doi: 10.1016/j.chempr.2020.02.006 (PMC7153771; doi:10.1016/j.chempr.2020.02.006)
Supplement: Document S1. Schemes S1–S6, Figures S1–S185, Tables S1–S18, Supplemental Experimental Procedures, and Supplemental References [file mmc1.pdf]

Chem, Volume *6*

Supplemental Information

Synthesis of a Mechanically Planar Chiral Rotaxane Ligand for Enantioselective Catalysis

Andrew W. Heard and Stephen M. Goldup

1. General Experimental Information *3*
2. Synthesis and Characterisation of Catalysts [Au(6)(Cl)] *5*
Rotaxanes (*S*,*R*$_{mp}$)-4 and (*S*,*S*$_{mp}$)-4 5
Rotaxane (*R*$_{mp}$)-5 19
Rotaxane (*S*$_{mp}$)-5 28
Rotaxane [Au((*R*$_{mp}$)-6)(Cl)] 32
Rotaxane [Au((*S*$_{mp}$)-6)(Cl)] 40
^{1}H NMR Stack Plot Demonstrating the binding of Cu^{I} to the Rotaxane Framework 42
3. Crystallographic Data: Assignment of Relative and Absolute Stereochemistry of (S,R$_{mp}$)-4 and (S,S$_{mp}$)-4 ..... *43*
Rotaxane (*S*,*R*$_{mp}$)-4 SCXRD Data (XRAY_ SRmp4.cif) 43
Rotaxane (*S*,*S*$_{mp}$)-4 SCXRD Data (XRAY_ SSmp4.cif) 46
4. Synthesis of Cyclopropanation Substrates *51*
Substituted 2-methyl-3-butyn-2-yl benzoate general procedure 51
2-methyl-3-butyn-2-yl benzoate, 7 51
2-methyl-3-butyn-2-yl pivaloate, S3' 53
2-methyl-3-butyn-2-yl phenylacetate, S4 54
2-methyl-3-butyn-2-yl 4-trifluoromethylbenzoate, S5 56
2-methyl-3-butyn-2-yl 4-methoxybenzoate, S6 58
2-methyl-3-butyn-2-yl 4-tertbutylbenzoate, S7 59
2-methyl-3-butyn-2-yl 3,5-ditertbutylbenzoate, S8 61
5. Cyclopropanation Reactions *63*
Screening of reaction conditions 63
Determination of the absolute stereochemistry of cyclopropanes 9 64
Cyclopropanation General Procedures 65
Cyclopropanes 9[6] 66
Cyclopropane 10 72
Cyclopropanes 12 80
Cyclopropanes 13[6] 86
Cyclopropanes 14 90
Cyclopropanes 15 94
Cyclopropanes 16 102
Cyclopropanes 17 106
Cyclopropanes 18 113
6. Additional Reaction Scope *124*

Cycloisomerisation 1 .... **124**

Cycloisomerisation 2 .... **127**

Intramolecular Hydroamination of Allenes **130**

7. Molecular Modelling ***133***

Models of the reaction of ester 7 and styrene (8) mediated by [Au(PPh$_3$)(Cl)] **133**

Diastereomeric transition states for the reaction of 7 with 8 mediated by [Au(6)(Cl)] **134**

Transition states for the formation of cyclopropanes 13, 15 and 16 mediated by [Au(6)(Cl)] **137**

8. References ***139***

1. General Experimental Information

Unless otherwise stated, all reagents were purchased from commercial sources (Acros Organics, Alfa Aesar, Fisher Scientific, FluoroChem, Sigma Aldrich and VWR) and used without further purification. Styrene was purified by vacuum distillation prior to usage and stored under inert N_2 atmosphere. $[Cu(MeCN)_4]PF_6$ was prepared as described by Pigorsch and Köckerling.[1] Anhydrous solvents were purchased from Acros Organics. Petrol refers to the fraction of petroleum ether boiling in the range 40-60 °C. IPA refers to isopropanol. THF refers to tetrahydrofuran. EDTA-NH_3 solution refers to an aqueous solution of NH_3 (17% w/w) saturated with sodium-ethylenediaminetetraacetate. $CDCl_3$ (without stabilising agent) was distilled over $CaCl_2$ and K_2CO_3 prior to use. Compounds **1**,[2] **2**,[3] and **3**[4] were synthesised according to literature procedure. Unless otherwise stated, all reactions were performed in oven dried glassware under an inert N_2 atmosphere with purchased anhydrous solvents. Unless otherwise stated experiments carried out in sealed vessels were performed in CEM microwave vials, with crimped aluminium caps and PTFE septa.

Flash column chromatography was performed using Biotage Isolera-4 or Isolera-1 automated chromatography system. SiO_2 cartridges were purchased commercially from Teledyne Technologies, or Biotage (SNAP or ZIP, 50 µm irregular silica, default flow rates). Neutralised SiO_2 refers to ZIP cartridges which were eluted with petrol-NEt_3 (99 : 1, 5 column volumes), followed by petrol (5 column volumes). H_2O saturated SiO_2 refers to ZIP cartridges first eluted with H_2O saturated petrol-Et_2O-EtOAc (5 : 3 : 2 shaken with H_2O and the layers separated) followed by petrol-Et_2O-EtOAc (5 : 3 : 2) before loading the compound. Analytical TLC was performed on pre-coated silica gel plates on aluminum (0.25 mm thick, 60F254, Merck, Germany) and observed under UV light (254 nm) or visualised with $KMnO_4$ stain.

All melting points were determined using a Griffin apparatus and are uncorrected. NMR spectra were recorded on Bruker AV400 or AV500 instrument, at a constant temperature of 298 K. Chemical shifts are reported in parts per million from low to high field and referenced to residual solvent. Coupling constants (*J*) are reported in Hertz (Hz). Standard abbreviations indicating multiplicity were used as follows: m = multiplet, quint = quintet, q = quartet, t = triplet, d = doublet, s = singlet, app. = apparent, br = broad, sept = septet. Signal assignment was carried out using 2D NMR methods (COSY, NOESY, TOCSY, HSQC, HMBC or ^{31}P-^{1}H HMBC) where necessary. In some cases, complex multiplets with multiple contributing proton signals, exact assignment was not possible. In interlocked compounds, all proton signals corresponding to axle

components are in lower case, and all proton signals corresponding to the macrocycle components are in upper case. For mixtures of diastereomeric cyclopropanes, upper case is used to denote the major diastereoisomer, and lower case is used to denote the minor diastereoisomer. Low resolution mass spectrometry was carried out by the mass spectrometry services at University of Southampton (Waters TQD mass spectrometer equipped with a triple quadrupole analyser with UHPLC injection [BEH C18 column; MeCN-H_2O gradient {0.2% formic acid}]). High resolution mass spectrometry was carried out either by the mass spectrometry service at the University of Edinburgh (ThermoElectron MAT 900) or by the mass spectrometry services at the University of Southampton (MaXis, Bruker Daltonics, with a Time of Flight (TOF) analyser; samples were introduced to the mass spectrometer via a Dionex Ultimate 3000 autosampler and uHPLC pump in a gradient of 20% MeCN in hexane to 100% acetonitrile (0.2% formic acid) over 5-10 min at 0.6 mL/min; column: Acquity UPLC BEH C18 (Waters) 1.7 micron 50 × 2.1mm). Circular dichroism spectra were acquired on an Applied Photo-physics Chirascan spectropolarimeter, recorded using Applied Photophysics software Ver. 4.2.0 in dried spectroscopic grade $CHCl_3$, following overnight desiccation of the sample, at a concentration range of 0.1-1 10^{-4} M, in a quartz cell of 1 cm path length, at a temperature of 293 K.

Stereochemical purity was determined by Chiral Stationary Phase HPLC on a Waters Acquity Arc Instrument at 303 K, with *n*-hexane-isopropanol isocratic eluents. Regis Technologies (*S*,*S*)-Whelk-O1 (1-(3,5-dinitrobenzamido)-1,2,3,4-tetrahydrophenanthrene stationary phase), RegisPack (tris-(3,5-dimethylphenyl) carbamoyl amylose stationary phase) and RegisCell (tris-(3,5-dimethylphenyl) carbamoyl cellulose stationary phase) columns were used throughout (5 micron, column dimensions 25 cm x 4.6 mm). The absolute stereochemistry of mechanically planar chiral rotaxanes **4**-**6** was determined by X-ray crystallography of rotaxanes **4**, with the mechanical stereogenic unit assumed to be invariant through subsequent steps. Stereochemical labels were assigned using our established approach.[5] The absolute stereochemistry of cyclopropanes **9** was determined by comparison with the known stereochemical outcome of the reaction mediated by (*R*)-DTBM-SEGPHOS®$(AuCl)_2$.[6]

2. Synthesis and Characterisation of Catalysts [Au(6)(Cl)]

Rotaxanes (S,R_{mp})-**4** and (S,S_{mp})-**4**

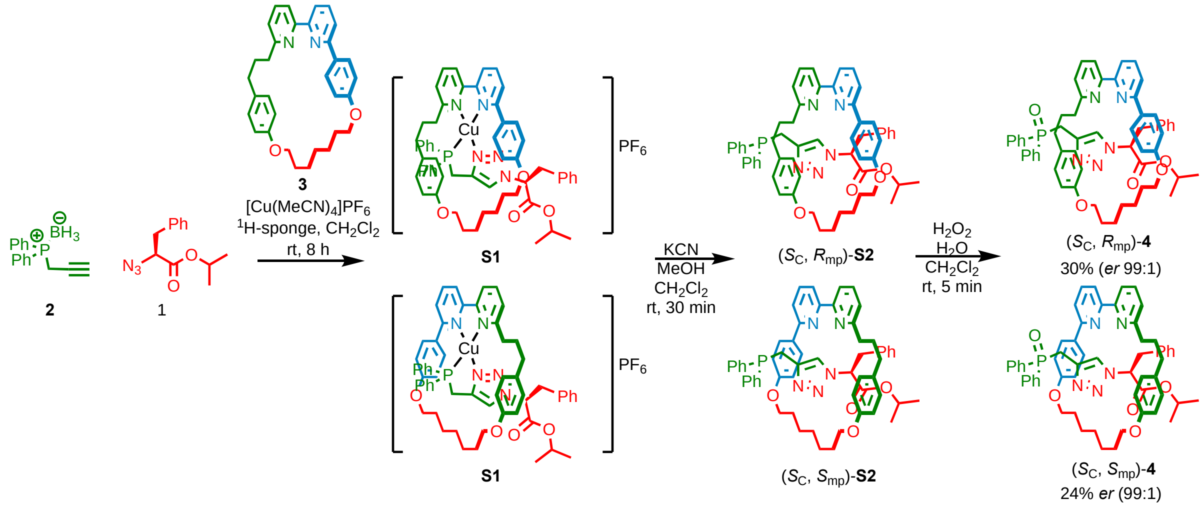

Scheme S1: Synthesis of (S,R_{mp})-**4** and (S,S_{mp})-**4**.

3 (200 mg, 0.418 mmol, 1.0 eq.), **2** (298 mg, 1.25 mmol, 3.0 eq.), **1** (292 mg, 1.25 mmol, 3.0 eq.), $[Cu(MeCN)_4]PF_6$ (150 mg, 0.401 mmol, 0.96 eq.) and ^{1}H-sponge (89.5 mg, 0.418 mmol, 1.0 eq.) were stirred in anhydrous CH_2Cl_2 (10 mL) at rt for 8 h under N_2. The orange solution was diluted with CH_2Cl_2 (10 mL), washed with sat. EDTA-NH_3 (25 mL) and then brine (25 mL). The combined aqueous washes were extracted with CH_2Cl_2 (3 x 25 mL). The combined organic extracts were dried ($MgSO_4$), and the solvent removed *in vacuo*. The residue was purified by column chromatography (neutralised SiO_2 (see general experimental), petrol-CH_2Cl_2 1 : 1 with 0→5% MeOH gradient), yielding an orange foam containing both diastereoisomers of **S1** (393 mg, 0.343 mmol, *dr* 1.0 : 1.1, 82%).

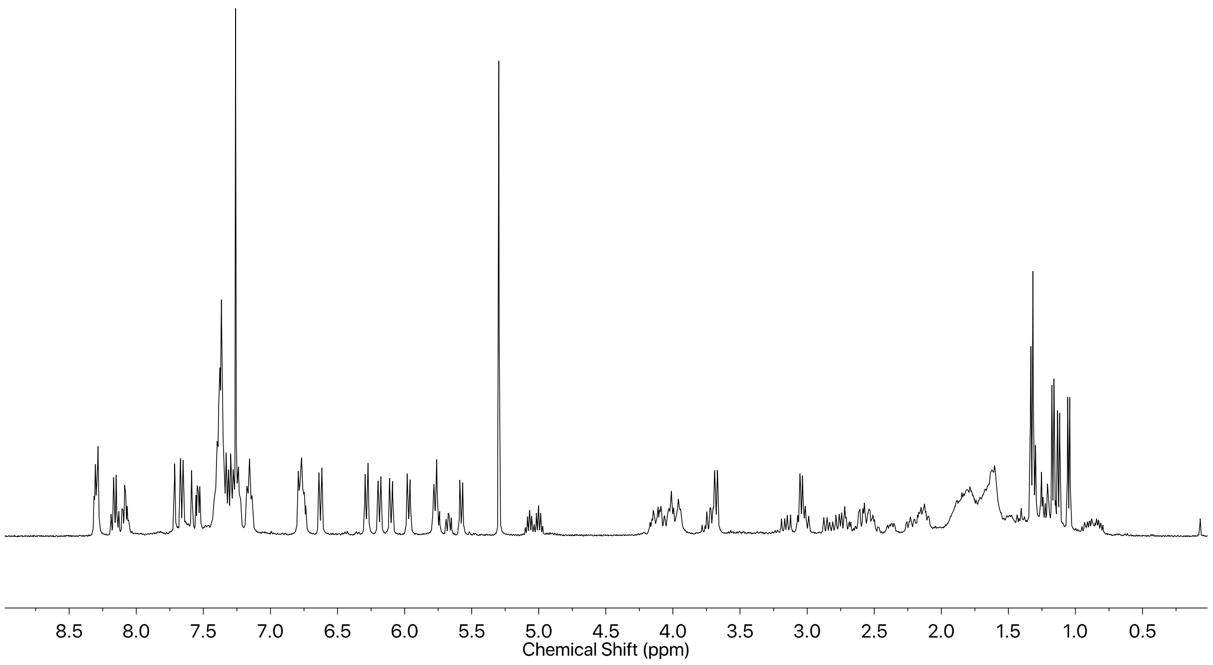

Figure S1: ^{1}H NMR (400 MHz, $CDCl_3$) of the diastereomeric mixture of Cu complexes **S1**.

The diastereomeric mixture of **S1** (393 mg, 0.343 mmol, 1.0 eq.) in CH_2Cl_2 (2.0 mL) was added to a solution of KCN (100 mg, 1.54 mmol, 4.5 eq.) in MeOH (2.0 mL), and the reaction mixture stirred for 30 min at rt. The solution was washed with brine (15 mL), aqueous H_2O_2 (35% w/w, 20 mL), brine (20 mL), and the combined aqueous washes were extracted with CH_2Cl_2 (3 x 15 mL). The combined organic phases were dried ($MgSO_4$) and the solvent removed *in vacuo* yielding a yellow foam (S,R_{mp})-**4**:(S,S_{mp})-**4** (295 mg, 0.309 mmol, *dr* 1.0 : 1.1, 90%). (S,R_{mp})-**4** and (S,S_{mp})-**4** were separated by column chromatography (H_2O saturated SiO_2, isocratic petrol-Et_2O-EtOAc 5 : 3 : 2) to give (S,R_{mp})-**4** (121 mg, 0.127 mmol, *er* = 99 : 1) and (S,S_{mp})-**4** (97 mg, 0.102 mmol, (S,R_{mp})-**4** : (R,S_{mp})-**4** : (S,S_{mp})-**4** = 0.6 : 1.0 : 98.4).

The absolute stereochemistry of the products was determined by SC-XRD (*vide infra* for a full discussion) and the stereolabels assigned based on our established approach using the priority atoms A-D indicated below. Stereochemical purity was determined by HPLC using racemic samples of rotaxanes **4** (synthesised as above) for comparison (**Figure S9**, **Figure S10**). The stereochemical purity of (S,R_{mp})-**4** was ultimately confirmed by analysis of its derivative, (R_{mp})-**5** (**Figure S32**), due to better HPLC peak separation, although it should be noted that direct HPLC analysis of (S,R_{mp})-**4** (**Figure S11**) is consistent with this value.

(S,R_{mp})-**4**

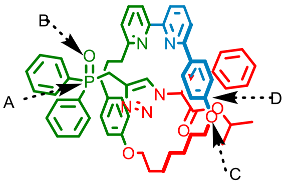

(S,R_{mp})-**4**

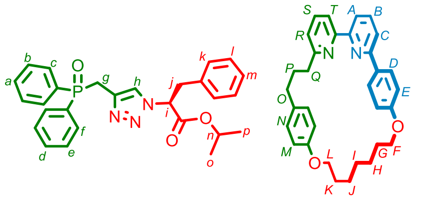

(S,R_{mp})-**4**

δ_H ($CDCl_3$, 400 MHz) 9.49 (1H, d, J = 2.0, **H_h**), 7.81 (1H, t, J = 7.8, **H_S**), 7.77 (2H, td, J = 9.0, 1.4, **H_f**), 7.76 (1H, t, J = 7.8, **H_B**), 7.71 (2H, ddd, J = 11.4, 7.4, 1.5, **H_c**), 7.63 (1H, d, J = 7.8, **H_R**), 7.59 (1H, d, J = 7.8, **H_T**), 7.44 (2H, ddd, J = 8.3, 7.4, 1.4, **H_e**), 7.41 (1H, d, J = 7.7, **H_C**), 7.38 (1H, m, **H_d**), 7.32 (2H, dt, J = 8.8, 2.5, **H_D**), 7.28 (1H, d, J = 7.8, **H_A**), 7.20 (1H, td, J = 7.4, 1.4, **H_a**), 7.12 (2H, td, J = 7.4, 2.6, **H_b**), 6.80 (2H, dt, J = 8.7, 2.3, **H_N**), 7.08-7.02 (3H, m, **H_l**, **H_m**), 6.72 (2H, dd, J = 7.0, 2.4, **H_k**), 6.48 (2H, dt, J = 8.6, 2.0, **H_M**), 6.47 (2H, dt, J = 8.8, 2.4, **H_E**), 4.73 (1H, dd, J = 12.4, 4.7, **H_i**), 4.51 (1H, sept., J = 6.2, **H_n**), 4.32 (1H, app. q (ddd), J = 7.4, **H_Q**), 3.90 (2H, m, **H_F**, **$H_{F'}$**), 3.82 (1H, dt, J = 7.8, 4.1, **$H_{Q'}$**), 3.57 (1H, dd, J = 15.9, 13.6, **H_g**), 3.13 (1H, t(dd), J = 12.8, **H_j**), 2.99 (1H, dd, J = 15.9, 11.1, **$H_{g'}$**), 2.80-2.71 (2H, m, **H_O**, **$H_{O'}$**), 2.64 (2H, td, J = 8.6, 3.8, **H_L**, **$H_{L'}$**), 2.22 (1H, dd, J = 13.5, 4.7, **$H_{j'}$**), 2.07-1.95 (2H, m, **H_P**, **H_K**), 1.92-1.73 (4H, m, **$H_{P'}$**, **$H_{K'}$**, **H_J**, **$H_{J'}$**), 1.65-1.42 (2H, m, **H_G**, **$H_{G'}$**), 0.95-0.70 (4H, m, **H_H**, **$H_{H'}$**, **H_I**, **$H_{I'}$**), 0.69 (3H, d, J = 6.3, **H_p**), 0.44 (3H, d, J = 6.3, **H_o**).

δ_C ($CDCl_3$, 101 MHz) 169.0, 163.0, 159.2, 158.8, 157.7, 157.5, 157.0, 137.2, 137.0, 136.0, 135.6 (d, J = 7.2), 134.5 (d, J = 79.0), 132.5 (d, J = 78.8), 131.8, 131.5 (d, J = 2.6), 131.3 (d, J = 2.6), 131.2 (d, J = 3.4), 131.1 (d, J = 3.3), 130.6, 129.4, 129.3, 128.9, 128.7 (d, J = 11.6), 128.4 (d, J = 11.6), 128.1, 127.4 (d, J = 5.3), 126.5, 121.9, 120.3, 120.0, 119.5, 115.1, 114.3, 68.6, 67.9, 66.2, 62.3, 37.1, 36.4, 35.1, 31.3, 28.8, 28.7, 28.0 (d, J = 69.1), 25.5, 25.1, 24.0, 21.2, 20.9.

$\delta_{31P\{1H\}}$ ($CDCl_3$, 202 MHz) 28.7.

HR-ESI-MS m/z = 952.4564 $[M + H]^+$ (calc. m/z for $C_{59}H_{62}N_5O_5P$ 952.4561).

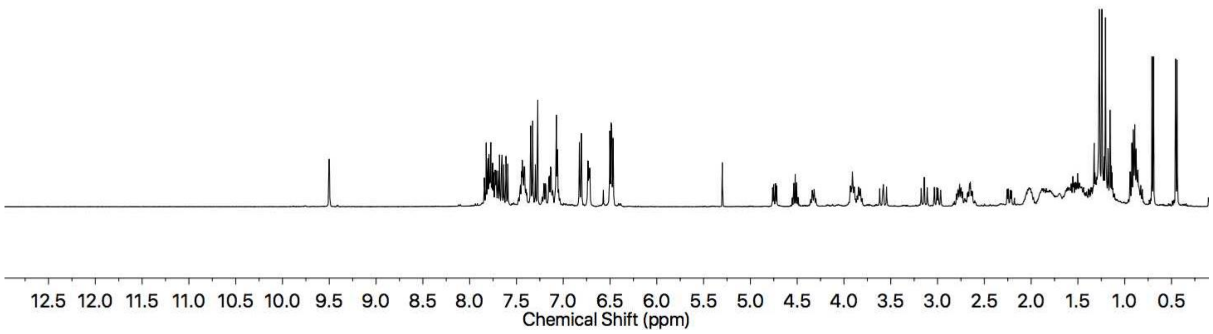

Figure S2: ^{1}H NMR (400 MHz, $CDCl_3$) of (S,R_{mp})-**4**.

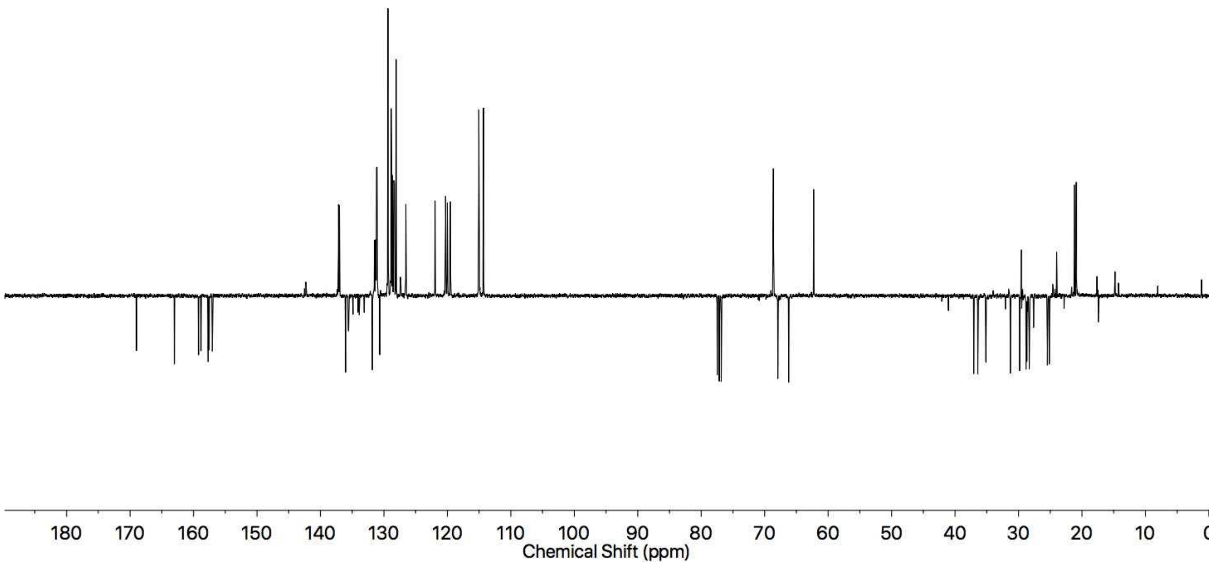

Figure S3: JMOD NMR (101 MHz, $CDCl_3$) of (S,R_{mp})-**4**.

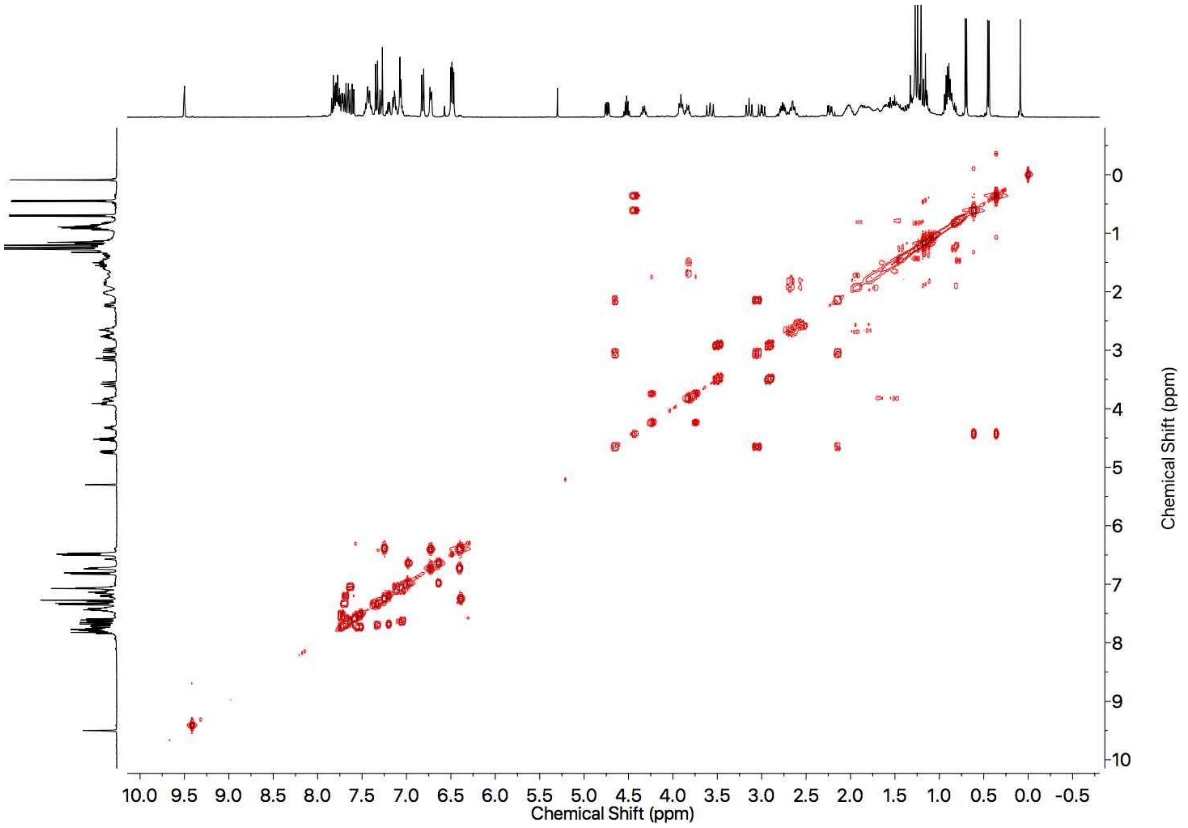

Figure S4: COSY NMR ($CDCl_3$) of (S,R_{mp})-**4**.

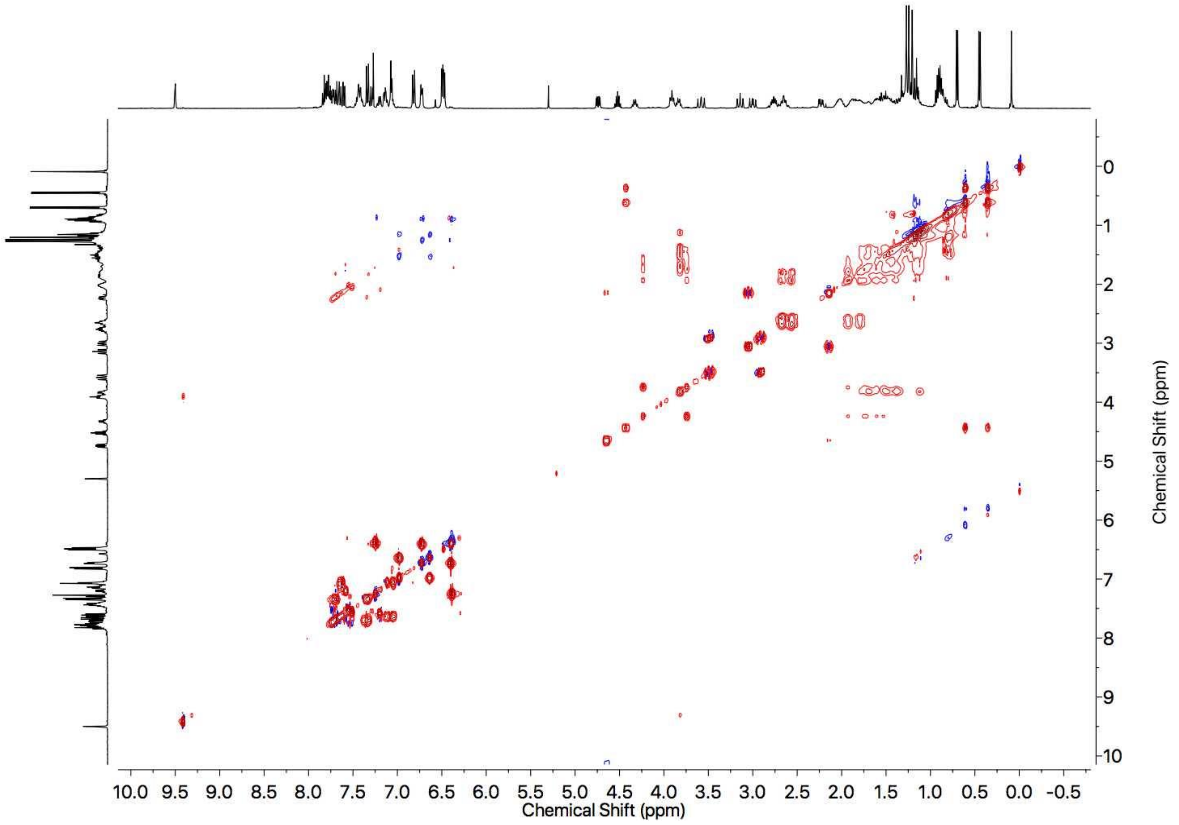

Figure S5: TOCSY NMR ($CDCl_3$) of (S,R_{mp})-**4**.

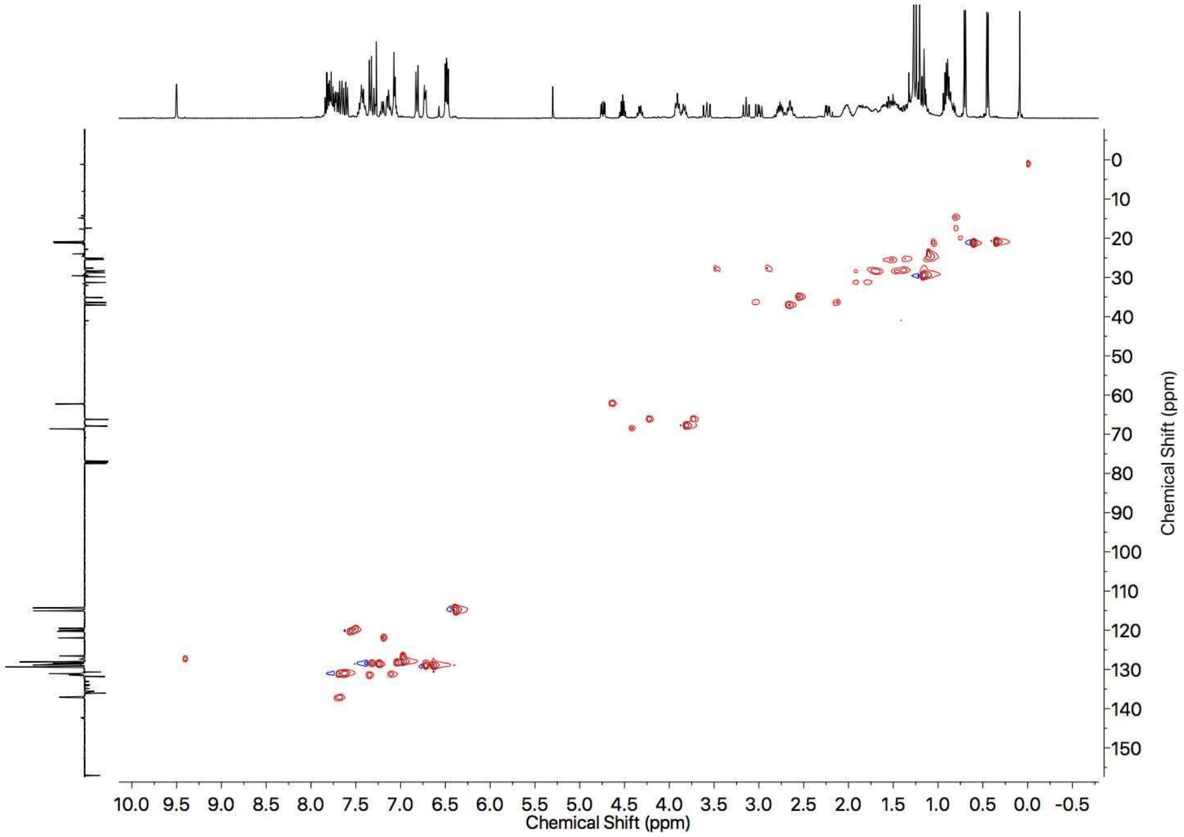

Figure S6: HSQC NMR ($CDCl_3$) of (*S*,*R*$_{mp}$)-**4**.

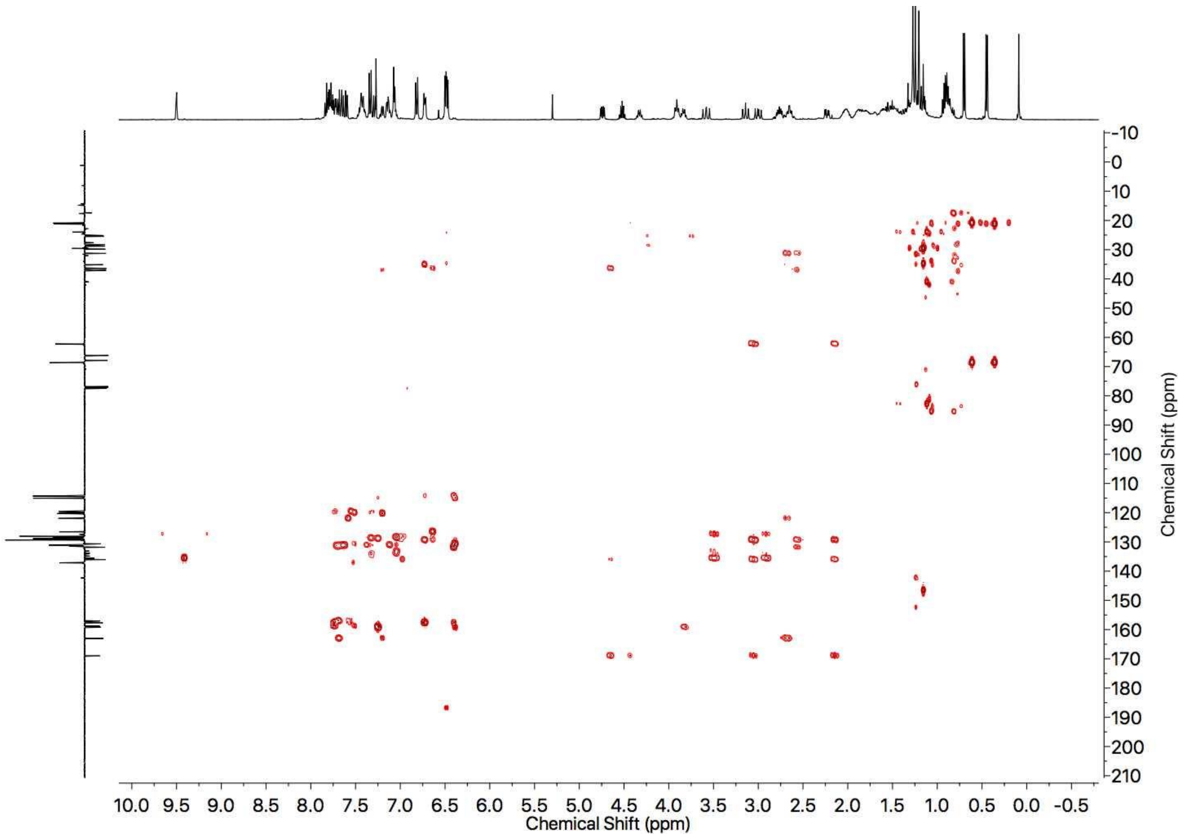

Figure S7: HMBC NMR ($CDCl_3$) of (*S*,*R*$_{mp}$)-**4**.

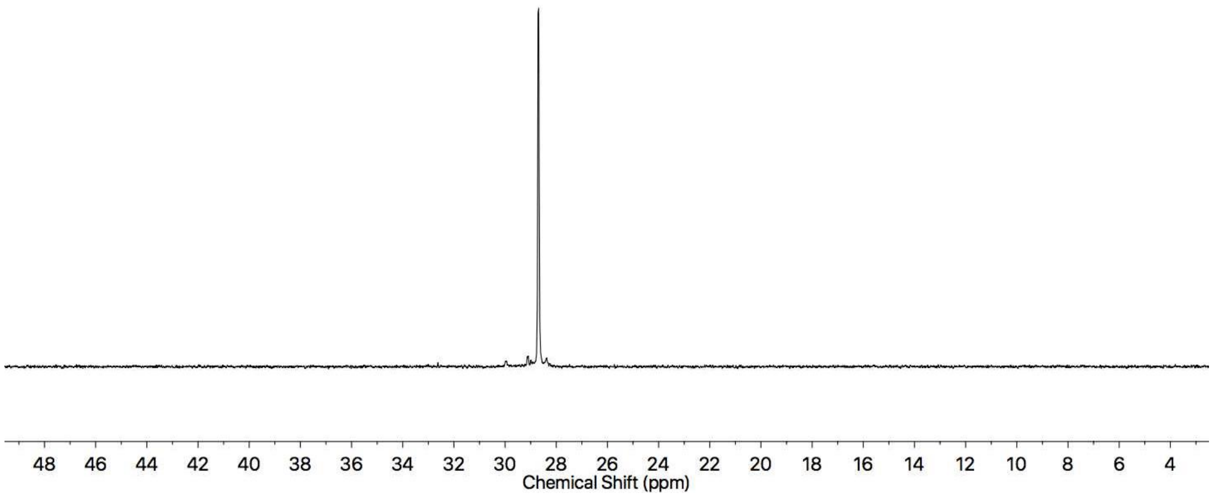

Figure S8: $^{31}P\{^{1}H\}$ NMR (202 MHz, $CDCl_3$) of (*S*,*R*$_{mp}$)-**4**.

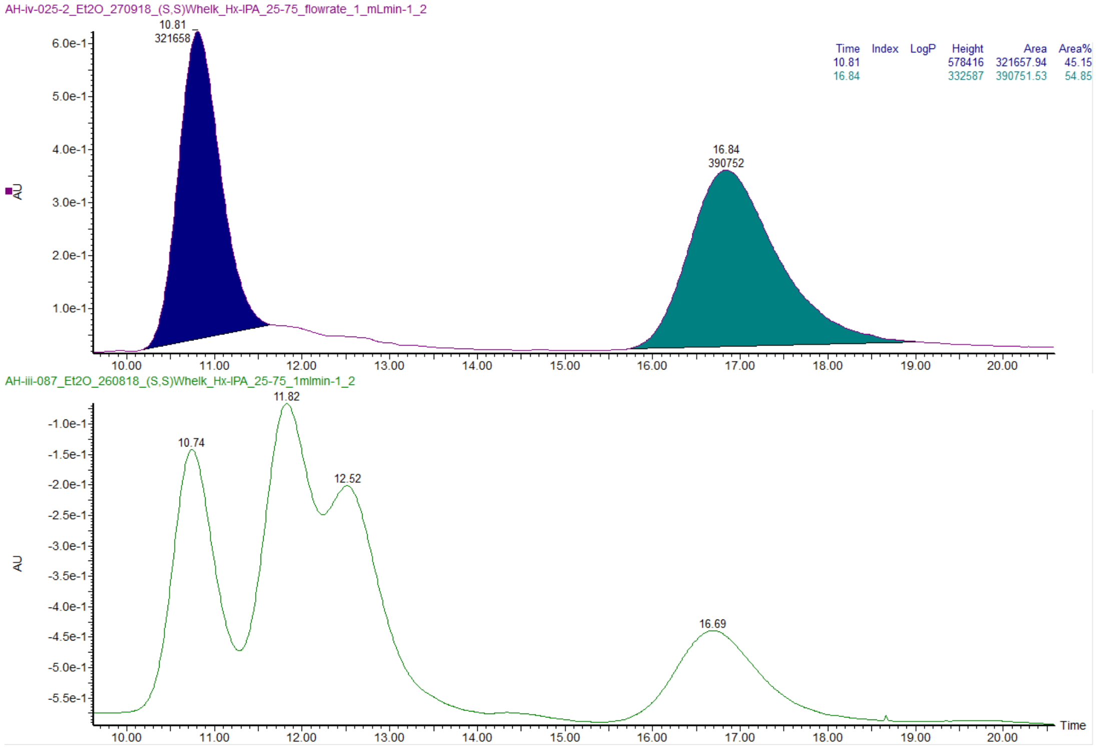

Figure S9: Chiral Stationary Phase HPLC ((*S*,*S*)Whelk, isocratic *n*-hexane-isopropanol 25 : 75, 303 K, load solvent Et_2O, 5 µL injection, flowrate 1 $mLmin^{-1}$) of the crude mixture of rotaxanes **4** (top) and the equivalent racemate (bottom). Retention times (min): (*S*,*R*$_{mp}$)-**4** 10.7, (*R*,*S*$_{mp}$)-**4** 11.8, (*R*,*R*$_{mp}$)-**4** 12.5, (*S*,*S*$_{mp}$)-**4** 16.7.

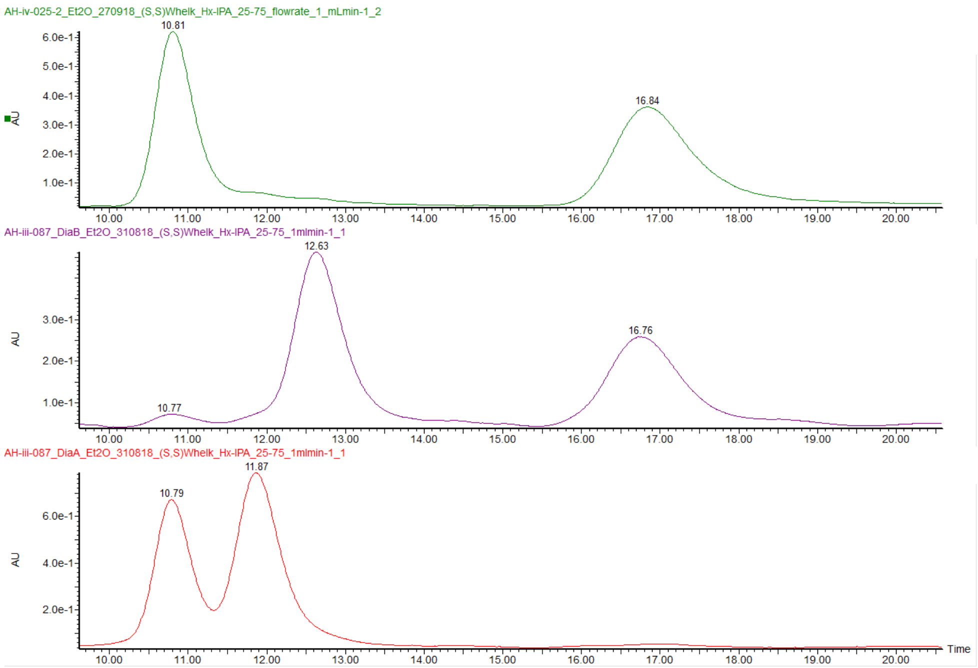

Figure S10: Chiral Stationary Phase HPLC ((*S*,*S*)Whelk, isocratic *n*-hexane-isopropanol 25 : 75, 303 K, load solvent Et_2O, 5 µL injection, flowrate 1 $mLmin^{-1}$) of the crude mixture of rotaxanes **4** (top) and the racemates of highly diastereomerically enriched samples (middle and bottom). Retention times (min): (S,R_{mp})-**4** 10.7, (R,S_{mp})-**4** 11.8, (R,R_{mp})-**4** 12.5, (S,S_{mp})-**4** 16.7.

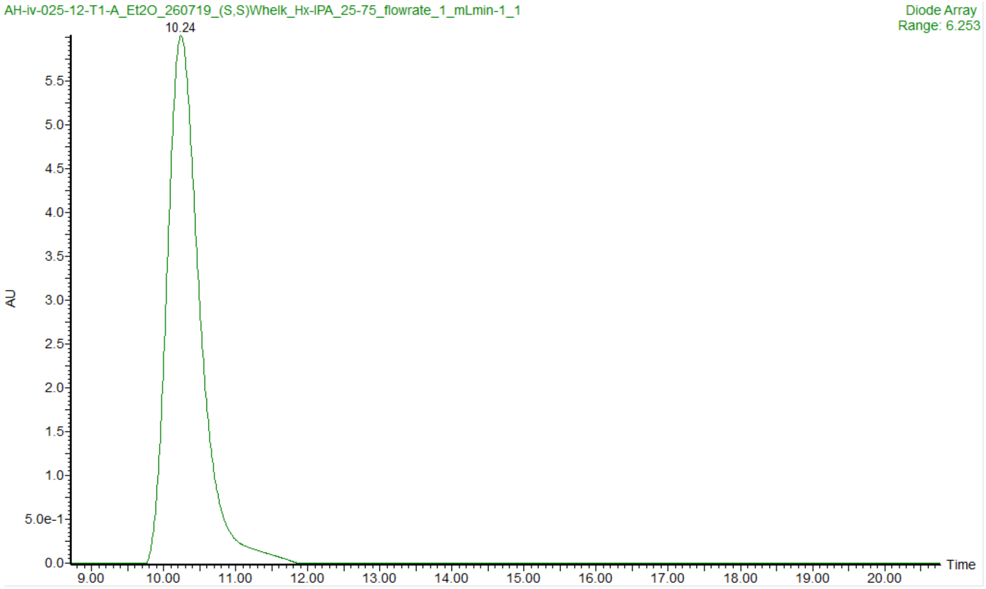

Figure S11: Chiral Stationary Phase HPLC ((*S*,*S*)Whelk, isocratic *n*-hexane-isopropanol 25 : 75, 303 K, load solvent Et_2O, 5 µL injection, flowrate 1 $mLmin^{-1}$) of 99 : 1 *er* (S,R_{mp})-**4.** Due to overlap of the peaks, the stereochemical purity is inferred from analysis of (S,R_{mp})-**5**. The HPLC trace shown here is consistent with this.

(*S*,*S*$_{mp}$)-**4**

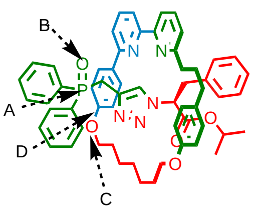

(*S*,*S*$_{mp}$)-**4**

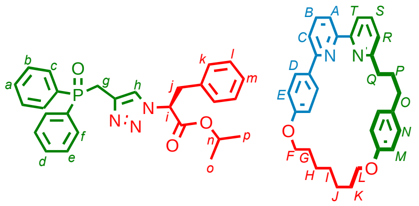

(*S*,*S*$_{mp}$)-**4**

δ$_H$ (CDCl$_3$, 400 MHz) 9.54 (1H, d, *J* = 1.8, ***H***$_h$), 7.87-7.79 (4H, m, ***H***$_b$, ***H***$_e$), 7.83 (1H, t, *J* = 7.6, ***H***$_B$), 7.75 (1H, t, *J* = 7.6, ***H***$_S$), 7.65 (1H, d, *J* = 7.6, ***H***$_A$), 7.62 (1H, d, *J* = 7.6, ***H***$_T$), 7.59 (1H, d, *J* = 8.0, ***H***$_a$ or ***H***$_d$), 7.44-7.35 (4H, m, ***H***$_c$, ***H***$_f$), 7.33 (1H, d, *J* = 7.7, ***H***$_C$), 7.32 (2H, dt, *J* = 8.9, 2.4, ***H***$_D$), 7.31-7.29 (1H, m, ***H***$_a$ or ***H***$_d$), 7.27 (1H, d, *J* = 7.6, ***H***$_R$), 6.91 (2H, d, *J* = 8.8, ***H***$_N$), 7.08-7.01 (3H, m, ***H***$_l$, ***H***$_m$), 6.53 (2H, d, *J* = 8.9, ***H***$_M$), 6.52 (2H, dd, *J* = 7.5, 1.9, ***H***$_k$), 6.31 (2H, dt, *J* = 9.1, 2.2, ***H***$_E$), 4.64 (1H, sept., *J* = 6.2, ***H***$_n$), 3.96 (1H, app. q., *J* = 6.7, ***H***$_F$), 3.82 (2H, t, *J* = 7.6, ***H***$_L$), 3.74 (1H, t, *J* = 6.6, ***H***$_{F'}$), 3.71 (1H, dd, *J* = 11.9, 4.3, ***H***$_i$), 3.56 (1H, dd, *J* = 15.1, 12.0, ***H***$_g$), 3.52 (1H, dd, *J* = 11.9, 4.3, ***H***$_j$), 3.18 (1H, t, *J* = 15.1, ***H***$_{g'}$), 2.92 (1H, t, *J* = 11.9, ***H***$_{j'}$), 2.80 (2H, dt, *J* = 14.0, 3.6, ***H***$_Q$, ***H***$_{Q'}$), 2.73 (1H, dt, *J* = 13.6, 5.0, ***H***$_O$), 2.58 (1H, dt, *J* = 13.6, 6.8, ***H***$_O$'), 2.00-1.87 (3H, m, ***H***$_P$, ***H***$_{P'}$, ***H***$_G$), 1.80-1.46 (5H, m, ***H***$_{G'}$, ***H***$_J$, ***H***$_{J'}$), 1.31-1.10 (2H, m, ***H***$_K$, ***H***$_{K'}$), 0.94-0.79 (4H, m, ***H***$_H$, ***H***$_{H'}$, ***H***$_I$, ***H***$_{I'}$),0.99 (3H, d, *J* = 6.2, ***H***$_p$), 0.63 (3H, d, *J* = 6.3, ***H***$_o$).

δ$_C$ (CDCl$_3$, 101 MHz) 167.8, 163.1, 159.4, 158.9, 157.6, 157.2, 137.1 (d, *J* = 3.4), 135.7 (d, *J* = 7.4), 135.5, 134.3 (d, *J* = 11.8), 133.3 (d, *J* = 12.5), 131.5, 131.4, 131.3 (d, *J* = 2.7) 131.3, 131.1 (d, *J* = 9.8), 130.9, 129.1, 129.1, 128.5 (d, *J* = 7.4), 128.4 (d, *J* = 7.4), 128.3, 128.1, 126.5, 126.2 (d, *J* = 6.3), 122.5, 120.2 (d, *J* = 17.6), 119.4, 115.6, 114.0, 68.7, 68.3, 66.0, 63.3, 37.3, 37.3, 35.1, 32.6, 29.7, 29.4, 29.3, 28.5, 28.3 (d, *J* = 22.5), 27.8, 25.0, 24.7, 23.9, 21.5, 21.1.

δ$_{31P\{1H\}}$ (CDCl$_3$, 202 MHz) 29.8.

HR-ESI-MS m/z = 952.4564 [M + H]$^+$ (calc. m/z for $C_{59}H_{62}N_5O_5P$ 952.4561).

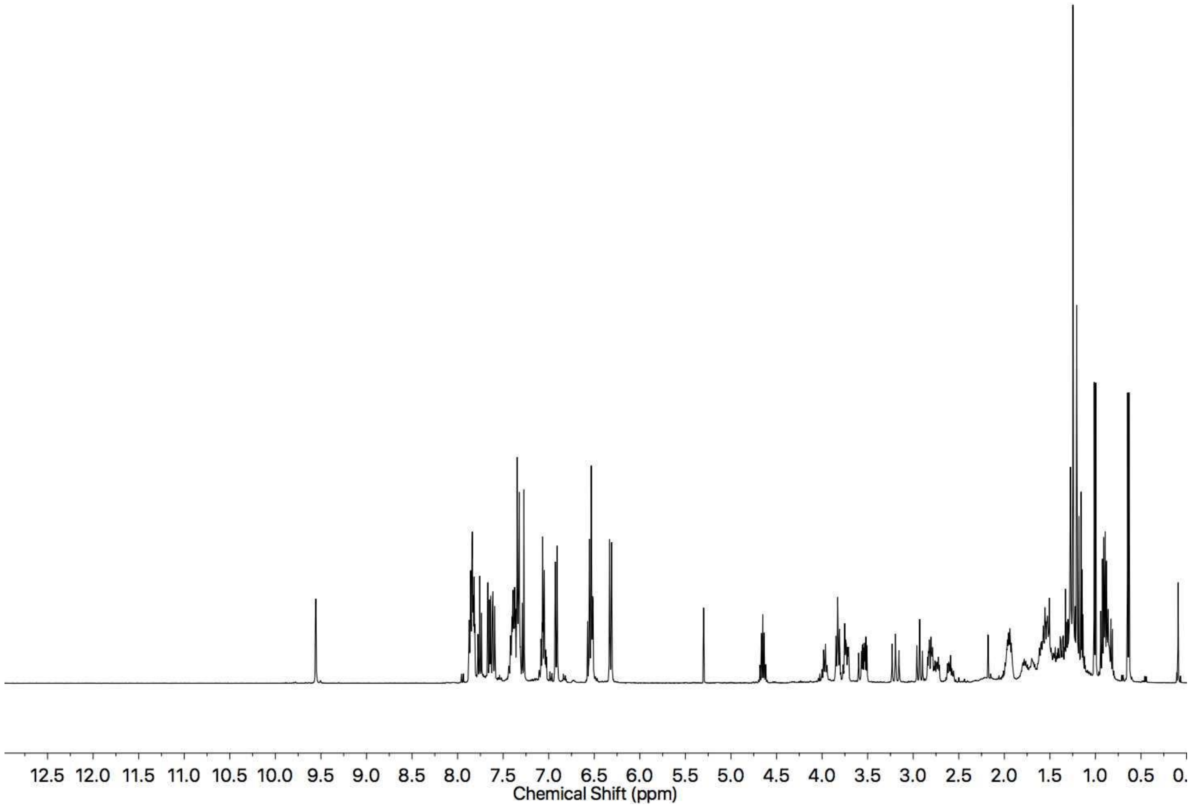

Figure S12: ^{1}H NMR (400 MHz, $CDCl_3$) of (*S*,*S*$_{mp}$)-**4**.

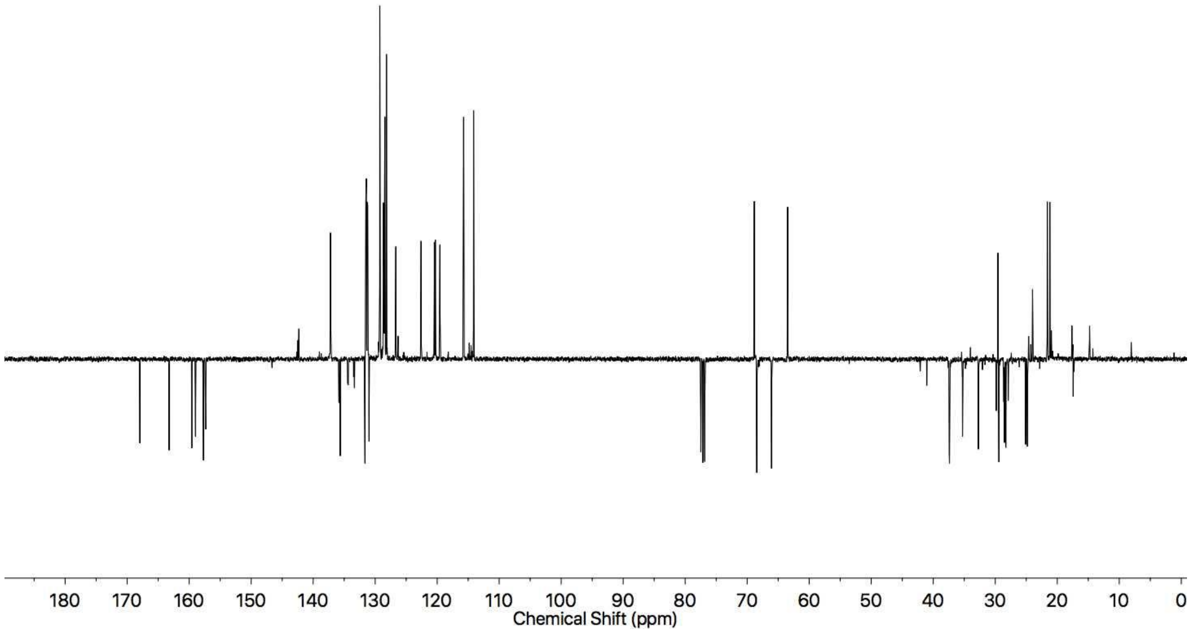

Figure S13: JMOD NMR (101 MHz, $CDCl_3$) of (*S*,*S*$_{mp}$)-**4**.

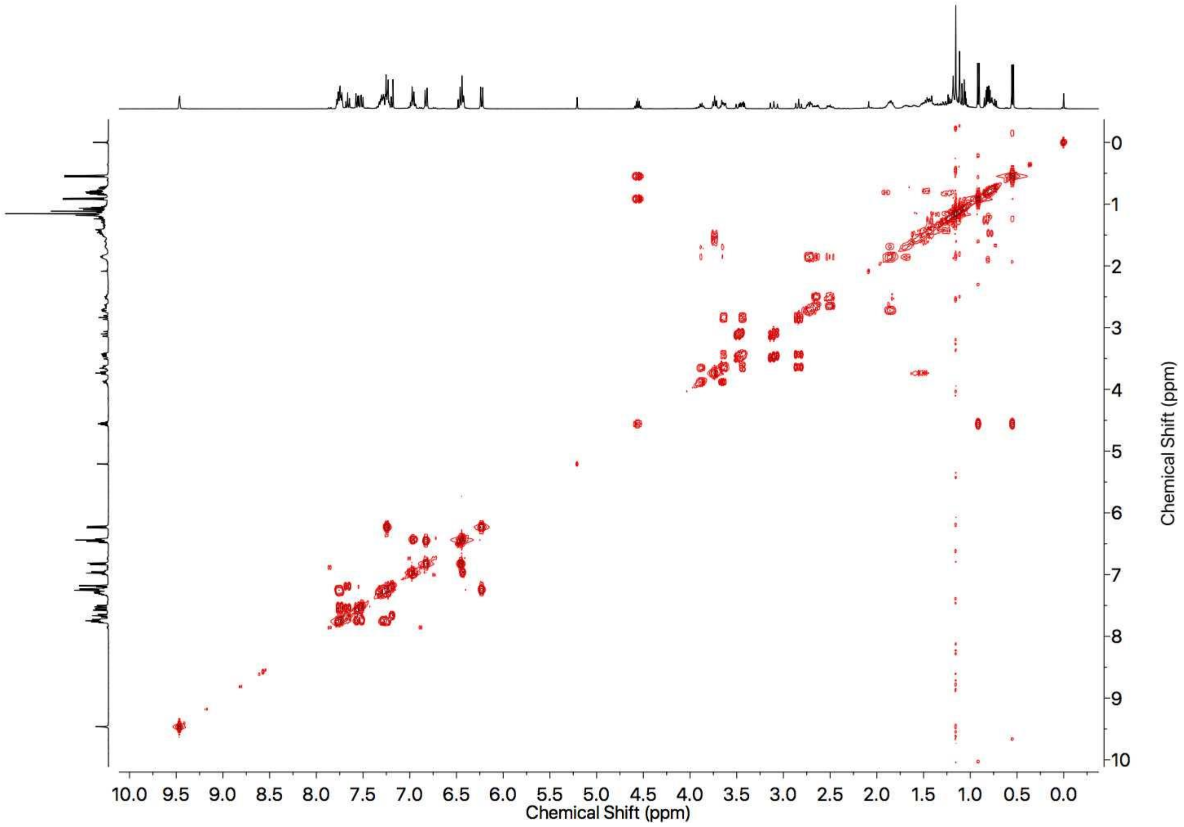

Figure S14: COSY NMR ($CDCl_3$) of (*S*,*S*mp)-**4**.

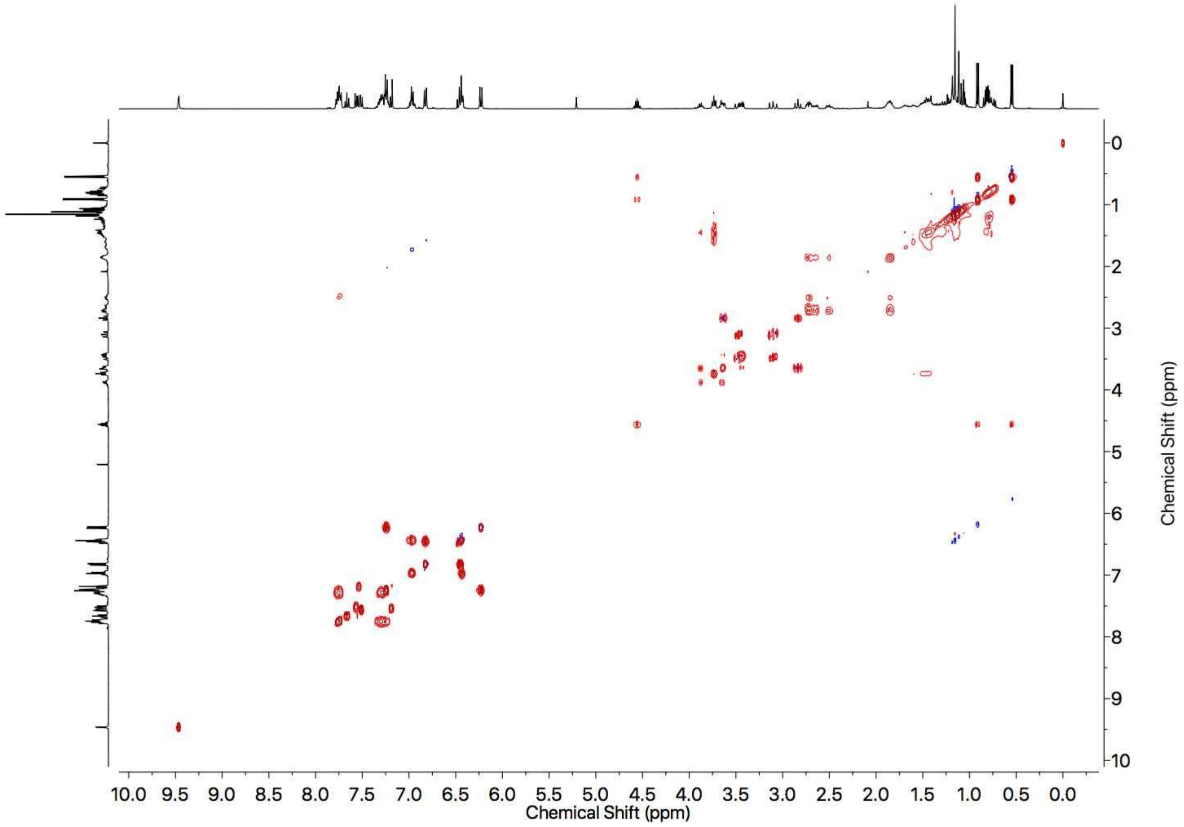

Figure S15: TOCSY NMR ($CDCl_3$) of (*S*,*S*mp)-**4**.

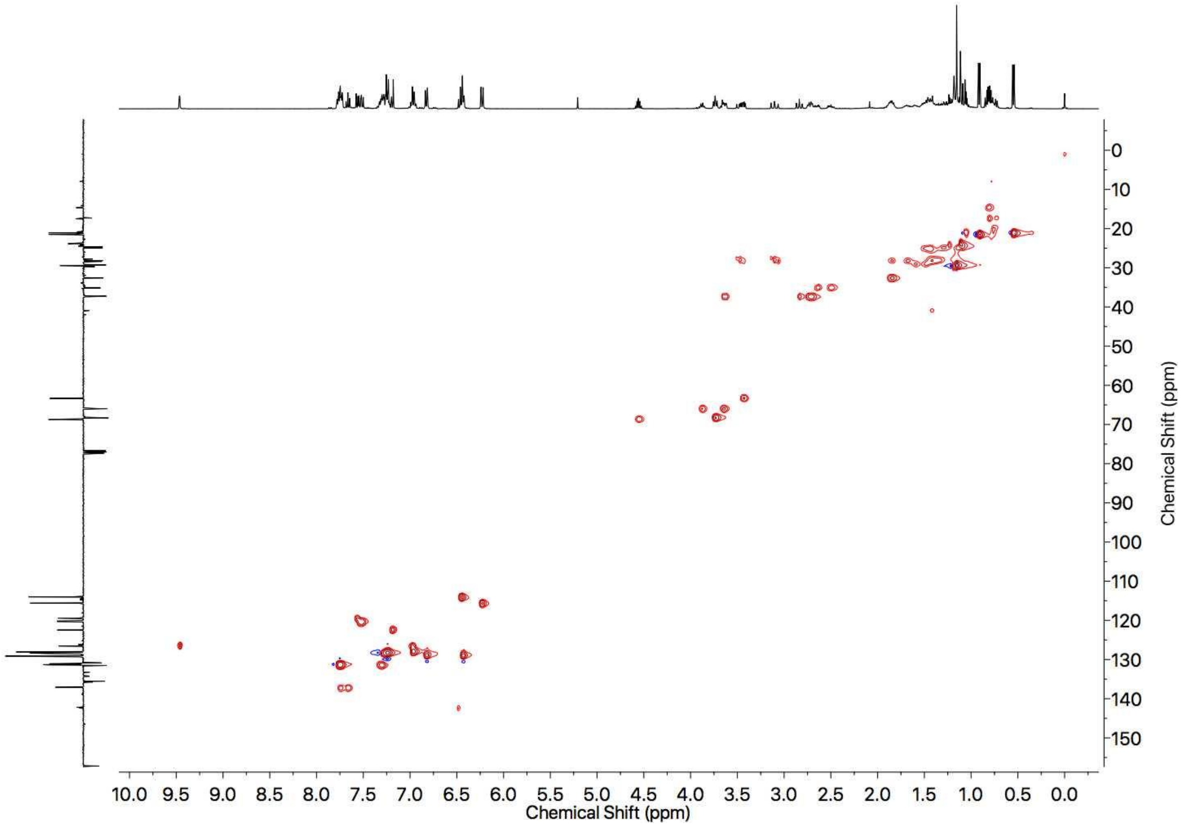

Figure S16: HSQC NMR ($CDCl_3$) of (*S*,*S*mp)-**4**.

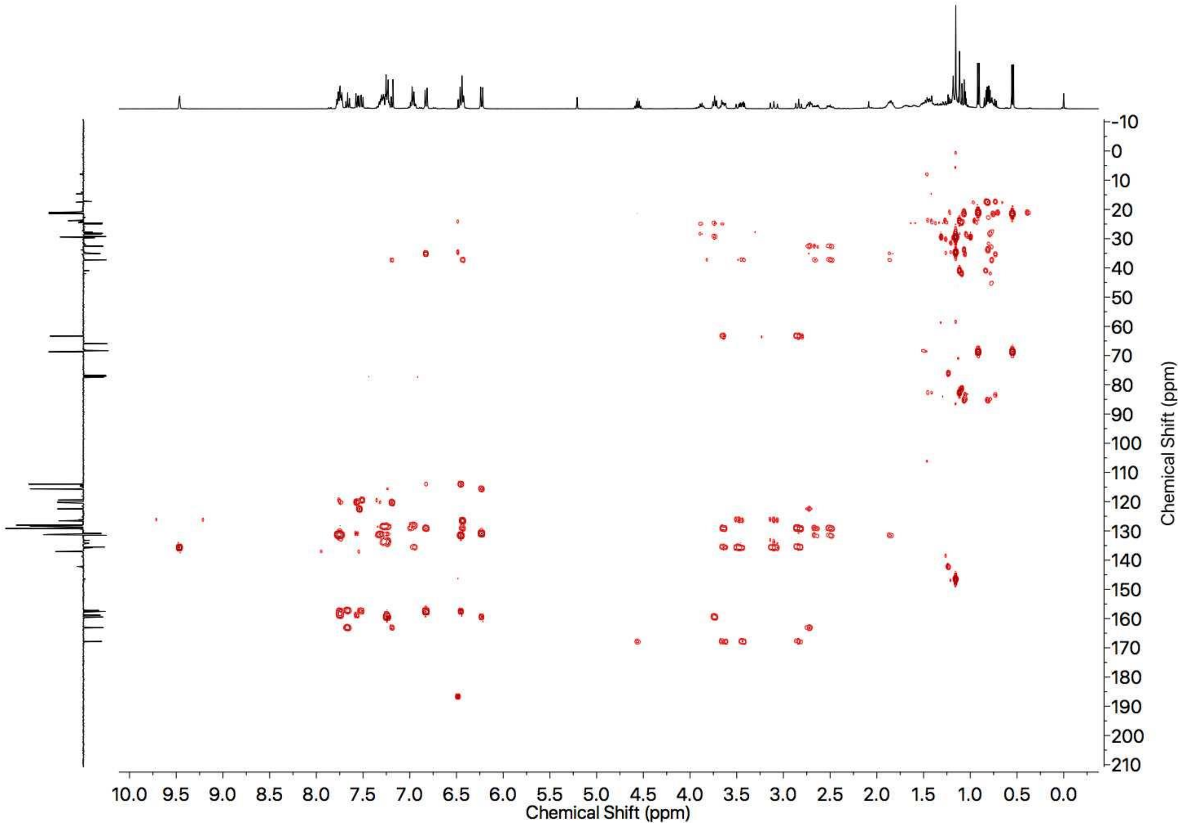

Figure S17: HMBC NMR ($CDCl_3$) of (*S*,*S*mp)-**4**.

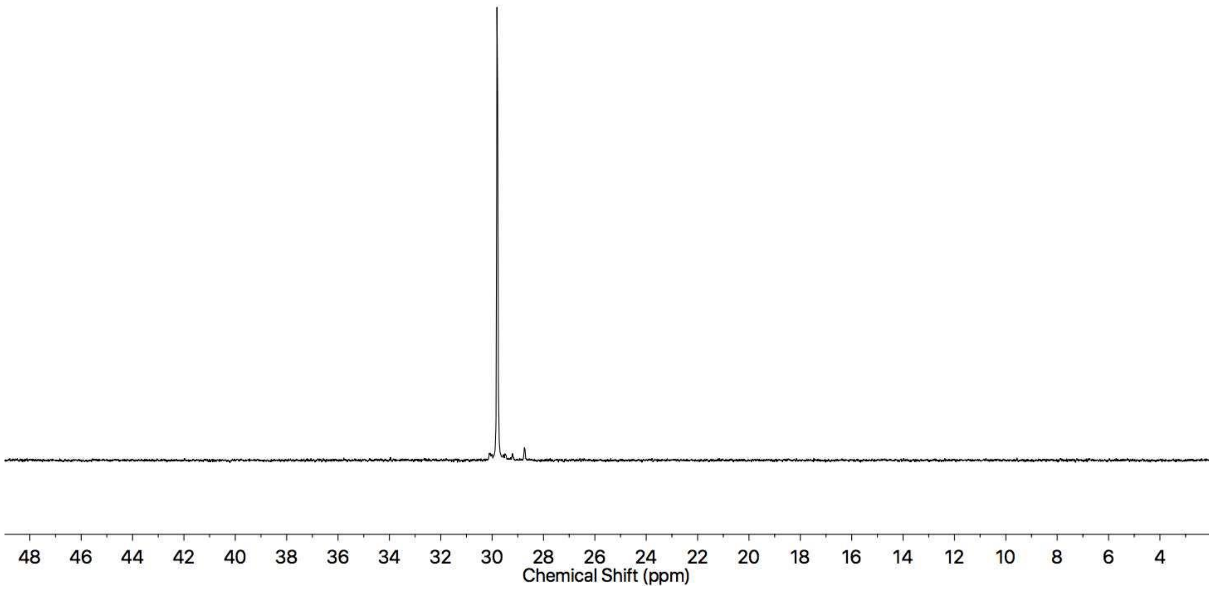

Figure S18: $^{31}P\{^{1}H\}$ NMR (202 MHz, $CDCl_3$) of (S,S_{mp})-**4**.

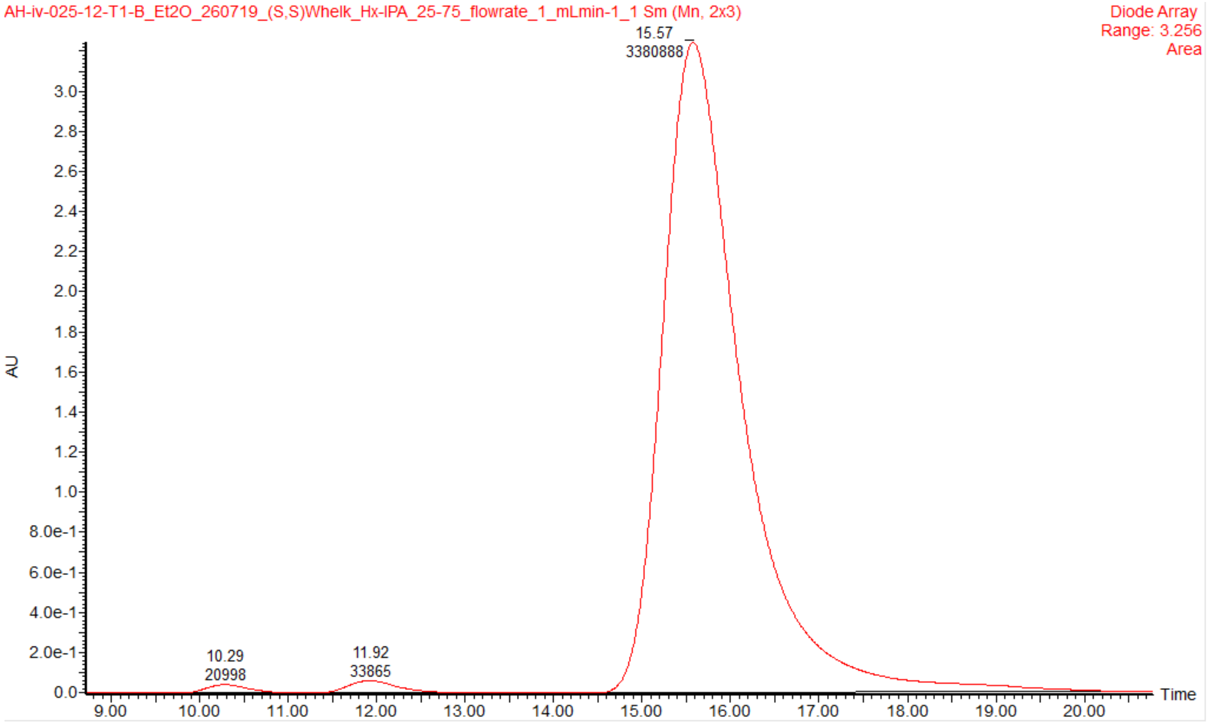

Figure S19: Chiral Stationary Phase HPLC ((S,S)Whelk, isocratic n-hexane-isopropanol 25 : 75, 303 K, load solvent Et_2O, 5 µL injection, flowrate 1 mLmin^{-1}). (S,R_{mp})-**4** : (R,S_{mp})-**4** : (S,S_{mp})-**4**, 0.6 : 1.0 : 98.4. Mechanically planar stereogenic element ratio (R_{mp}) : (S_{mp}) 1 : 99.

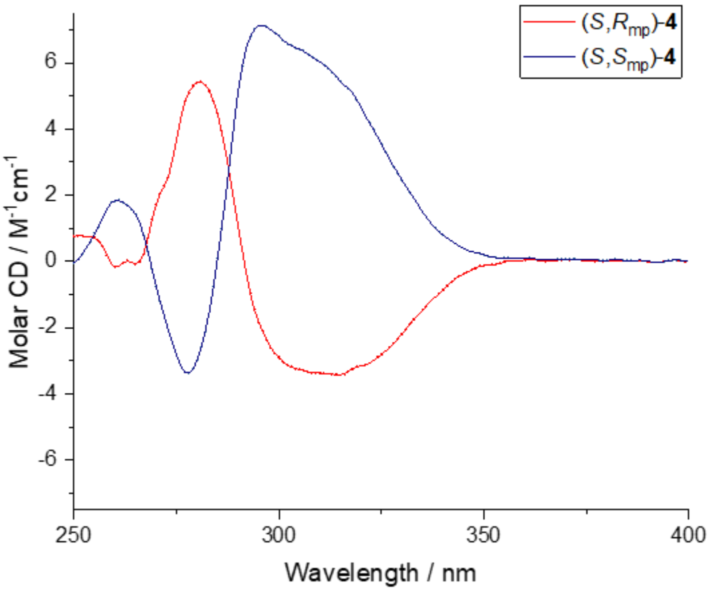

Figure S20: Circular Dichroism spectra of (*S*,*R*$_{mp}$)-**4** (53.4 µM, 99 : 1 *er*) and (*S*,*S*$_{mp}$)-**4** (58.8 µM, (*S*,*S*$_{mp}$)-**4** : (*R*,*S*$_{mp}$)-**4** : (*S*,*R*$_{mp}$)-**4**, 98.4 : 1.0 : 0.6) at 293 K in $CHCl_3$.

Rotaxane (R_{mp})-**5**

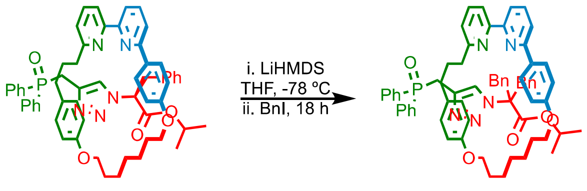

Scheme S2: Synthesis of (R_{mp})-**5**.

(S,R_{mp})-**4** (61.4 mg, 0.064 mmol, 1.0 eq.) was dissolved in anhydrous THF (3 mL) and transferred into a dry CEM MW vial under N_2. The solution was cooled to -78 °C and stirred for 20 min. Lithium bis(trimethylsilyl)amide (1 M in THF, 0.32 mL, 0.32 mmol, 5.0 eq.) was added to the reaction mixture and stirred for 10 min. Benzyl iodide (1 M in THF, 0.65 mL, 0.65 mmol, 10.0 eq.) was added. The reaction was allowed to warm to rt and stirred for 18 h. The reaction mixture was diluted with saturated NH_4Cl (30 mL) and extracted with CH_2Cl_2 (3 x 20 mL). The combined organic extracts were dried ($MgSO_4$) and solvent removed *in vacuo*. The residue was purified by column chromatography (SiO_2, petrol-EtOAc 0→50%) to give a yellow foam product (R_{mp})-**5** (53.7 mg, 0.051 mmol, 81%, *er* 99 : 1). *Enantiopurity was assessed by chiral stationary phase HPLC*. The absolute mechanical stereochemistry was inferred from that of the starting materials and the sterolabels assigned based on our established approach[5] using the priority atoms indicated below.

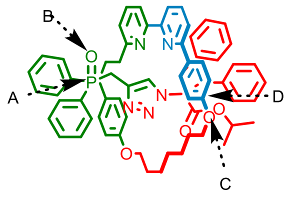

(R_{mp})-**5**

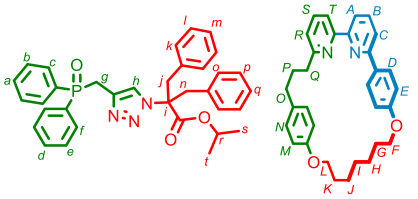

(R_{mp})-**5**

δ_H (CDCl$_3$, 400 MHz) 9.48 (1H, d, J = 1.6, $\boldsymbol{H}_h$), 7.77 (1H, t, J = 8.1, $\boldsymbol{H}_S$), 7.74 (1H, t, J = 8.1, $\boldsymbol{H}_B$), 7.64 (4H, ddd, J = 8.2, 7.7, 2.3, $\boldsymbol{H}_c$, $\boldsymbol{H}_f$), 7.58 (1H, d, J = 8.1, $\boldsymbol{H}_T$), 7.54 (1H, d, J = 7.8, $\boldsymbol{H}_A$), 7.52 (2H, d, J = 8.5, $\boldsymbol{H}_D$), 7.36 (4H, td, J = 7.6, 2.7, $\boldsymbol{H}_b$, $\boldsymbol{H}_e$), 7.29-7.23 (4H, m, $\boldsymbol{H}_a$, $\boldsymbol{H}_d$, $\boldsymbol{H}_R$, $\boldsymbol{H}_C$), 7.14-7.09 (3H, m, $\boldsymbol{H}_l$, - $\boldsymbol{H}_m$), 7.06-7.01 (3H, m, $\boldsymbol{H}_p$, $\boldsymbol{H}_q$), 6.90 (2H, d, J = 8.5, $\boldsymbol{H}_N$), 6.89 (2H, dd, J = 7.2, 2.1, $\boldsymbol{H}_k$), 6.79 (2H, dd, J = 7.1, 2.6, $\boldsymbol{H}_o$), 6.49 (2H, d, J = 8.5, $\boldsymbol{H}_E$), 6.35 (2H, d, J = 8.3, $\boldsymbol{H}_M$), 4.60 (1H, sept., J = 6.2, $\boldsymbol{H}_r$), 3.45 (1H, d, J = 15.0, $\boldsymbol{H}_g$), 3.93-3.81 (4H, m, $\boldsymbol{H}_L$, $\boldsymbol{H}_{L'}$, $\boldsymbol{H}_F$, $\boldsymbol{H}_{F'}$), 3.39 (1H, d, J = 14.8, $\boldsymbol{H}_j$), 3.09-2.97 (2H, m, $\boldsymbol{H}_Q$, $\boldsymbol{H}_{Q'}$), 2.92 (1H, d, J = 14.7, $\boldsymbol{H}_n$), 2.74 (1H, d, J = 15.2, $\boldsymbol{H}_g$), 2.68 (1H, br. d, J = 14.8, $\boldsymbol{H}_O$), 2.63-2.54 (1H, m, $\boldsymbol{H}_{j'}$), 2.38 (1H, d, J = 14.7, $\boldsymbol{H}_{n'}$), 2.02-1.89 (3H, m, $\boldsymbol{H}_P$, $\boldsymbol{H}_{P'}$, $\boldsymbol{H}_{O'}$), 1.87-1.77 (2H, m, $\boldsymbol{H}_J$, $\boldsymbol{H}_{J'}$), 1.75-1.62 (2H, m, $\boldsymbol{H}_G$, $\boldsymbol{H}_{G'}$), 1.61-1.48 (2H, m, $\boldsymbol{H}_K$, $\boldsymbol{H}_{K'}$), 1.47-1.36 (2H, m, $\boldsymbol{H}_I$, $\boldsymbol{H}_{I'}$), 0.94-0.86 (2H, m, $\boldsymbol{H}_H$, $\boldsymbol{H}_{H'}$), 0.71 (3H, d, J = 6.3, $\boldsymbol{H}_s$), 0.66 (3H, d, J = 6.3, $\boldsymbol{H}_t$).

δ_C (CDCl$_3$, 101 MHz) 169.1, 163.1, 159.4, 158.5, 158.1, 157.8, 157.7, 137.9 (d, J = 7.2), 137.0, 136.9, 136.5 (2C, d, J = 48.6), 132.0, 131.5, 131.2, 131.2, 131.1 (2C, d, J = 9.3), 130.6, 130.0, 129.9, 129.4, 128.4 (2C, d, J = 8.4), 128.3. (2C, d, J = 8.4), 127.7 (d, J = 6.4), 126.4, 126.1, 120.6, 120.4, 120.1, 119.0, 115.0, 114.6, 70.8, 69.7, 68.5, 66.9, 44.3, 42.7, 36.5, 34.7, 29.5, 28.9, 28.6, 28.1, 26.8 (d, J = 72.6), 25.5, 24.9, 24.0, 21.1, 21.0.

$\delta_{31P\{1H\}}$ (CDCl$_3$, 202 MHz) 28.1.

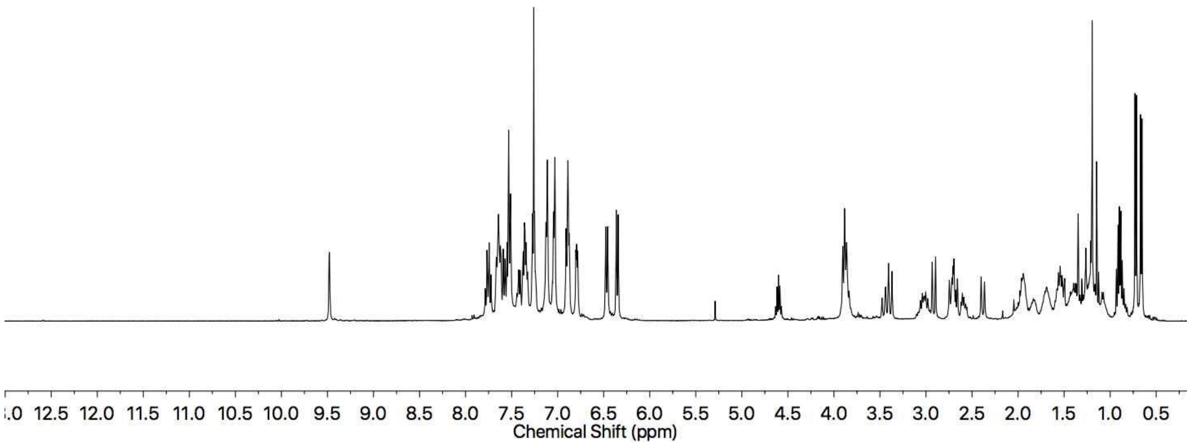

Figure S21: ^{1}H NMR (400 MHz, $CDCl_3$) of (R_{mp})-**5**.

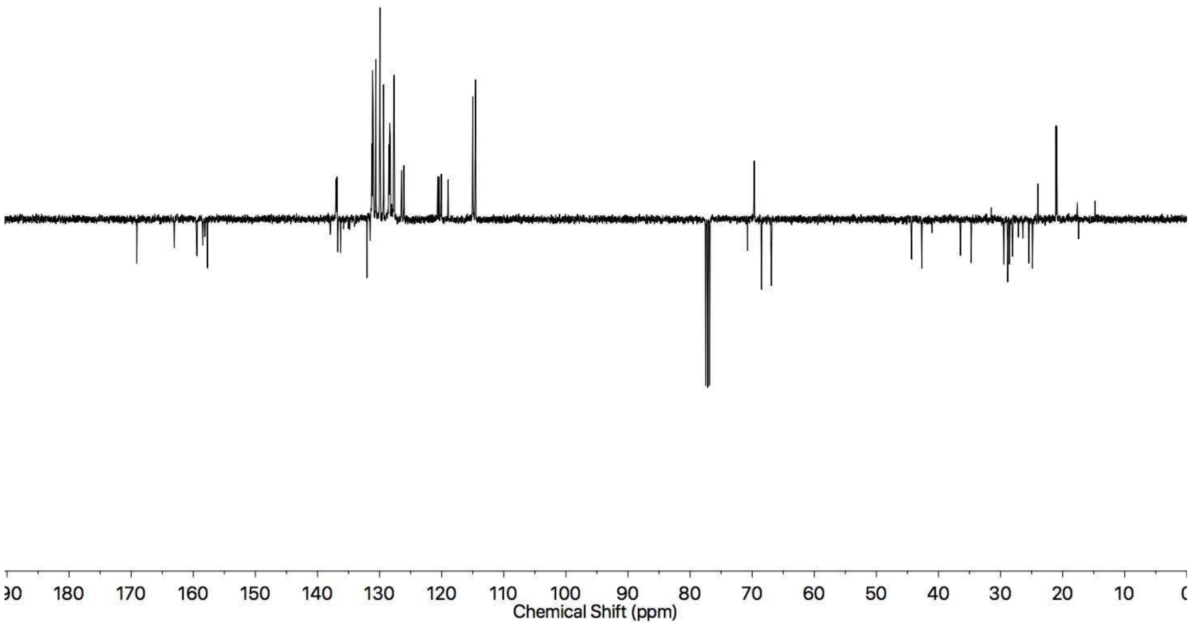

Figure S22: JMOD NMR (101 MHz, $CDCl_3$) of (R_{mp})-**5**.

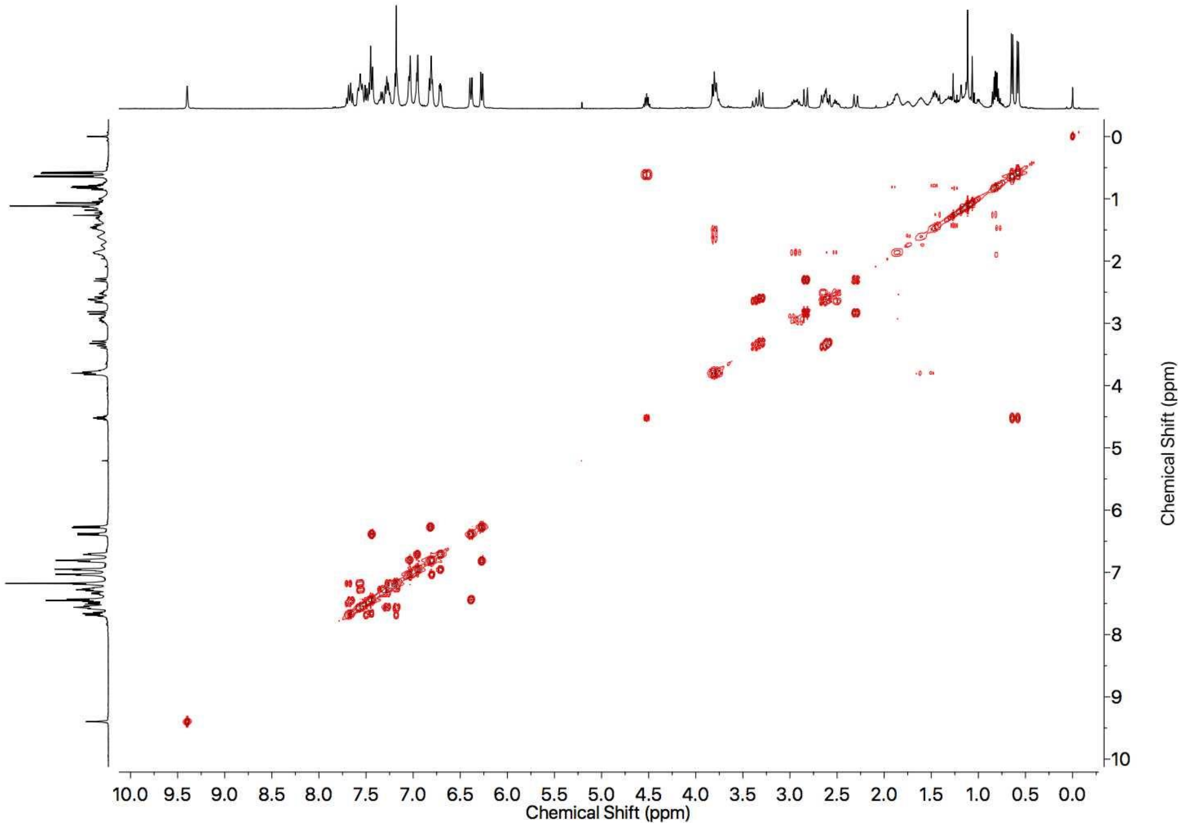

Figure S23: COSY NMR ($CDCl_3$) of (R_{mp})-**5**.

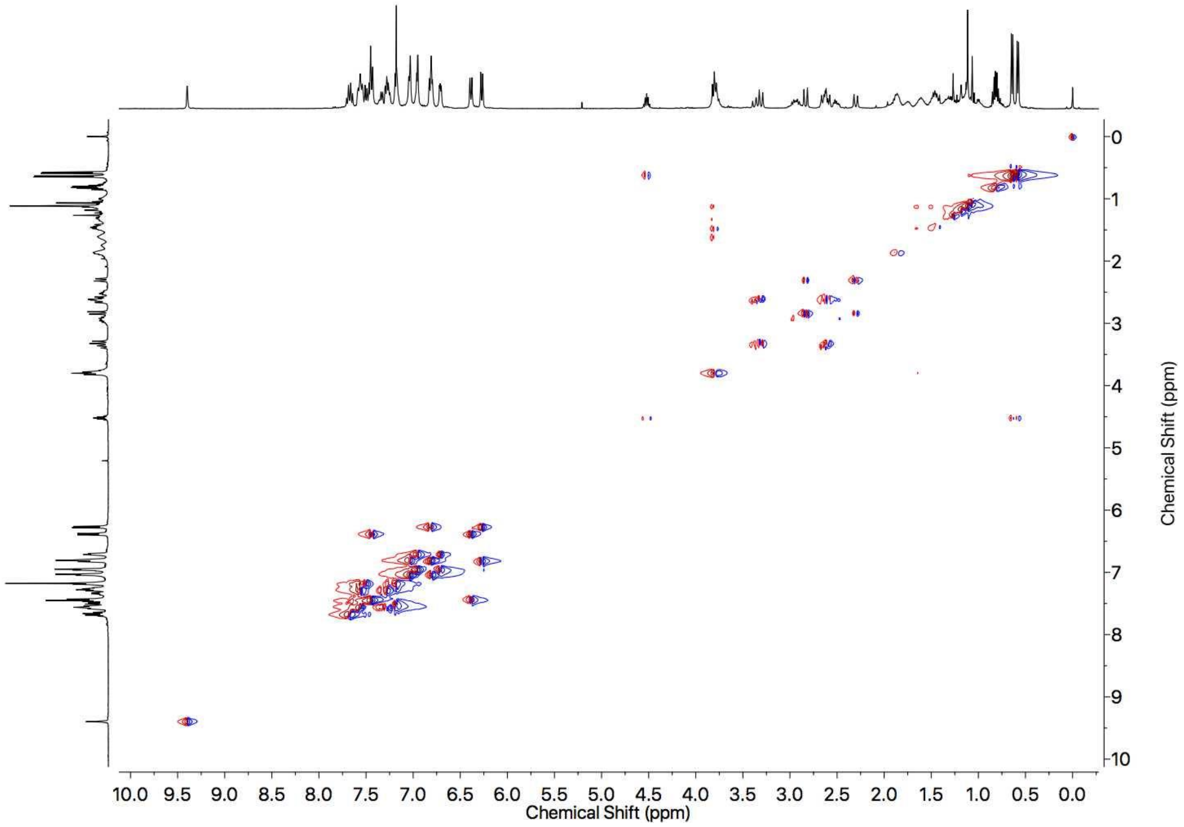

Figure S24: TOCSY NMR ($CDCl_3$) of (R_{mp})-**5**.

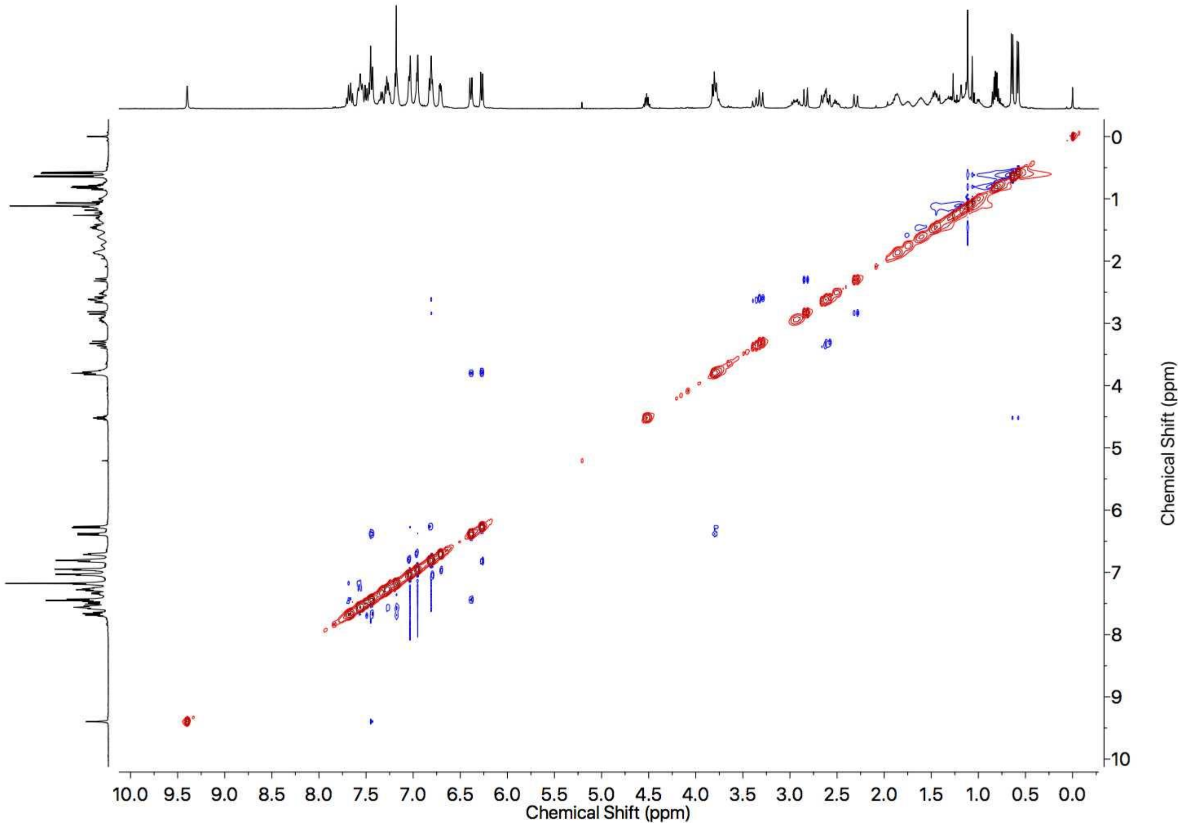

Figure S25: NOESY NMR ($CDCl_3$) of (R_{mp})-**5**.

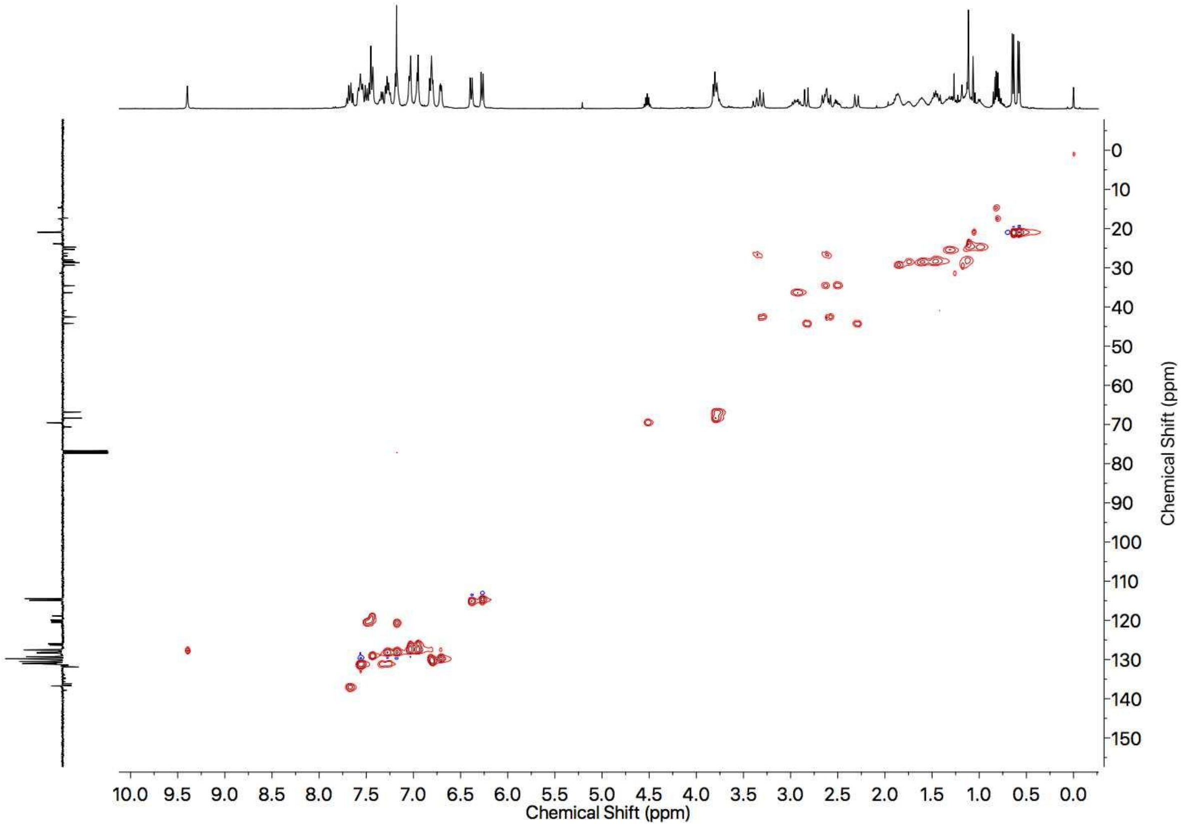

Figure S26: HSQC NMR ($CDCl_3$) of (R_{mp})-**5**.

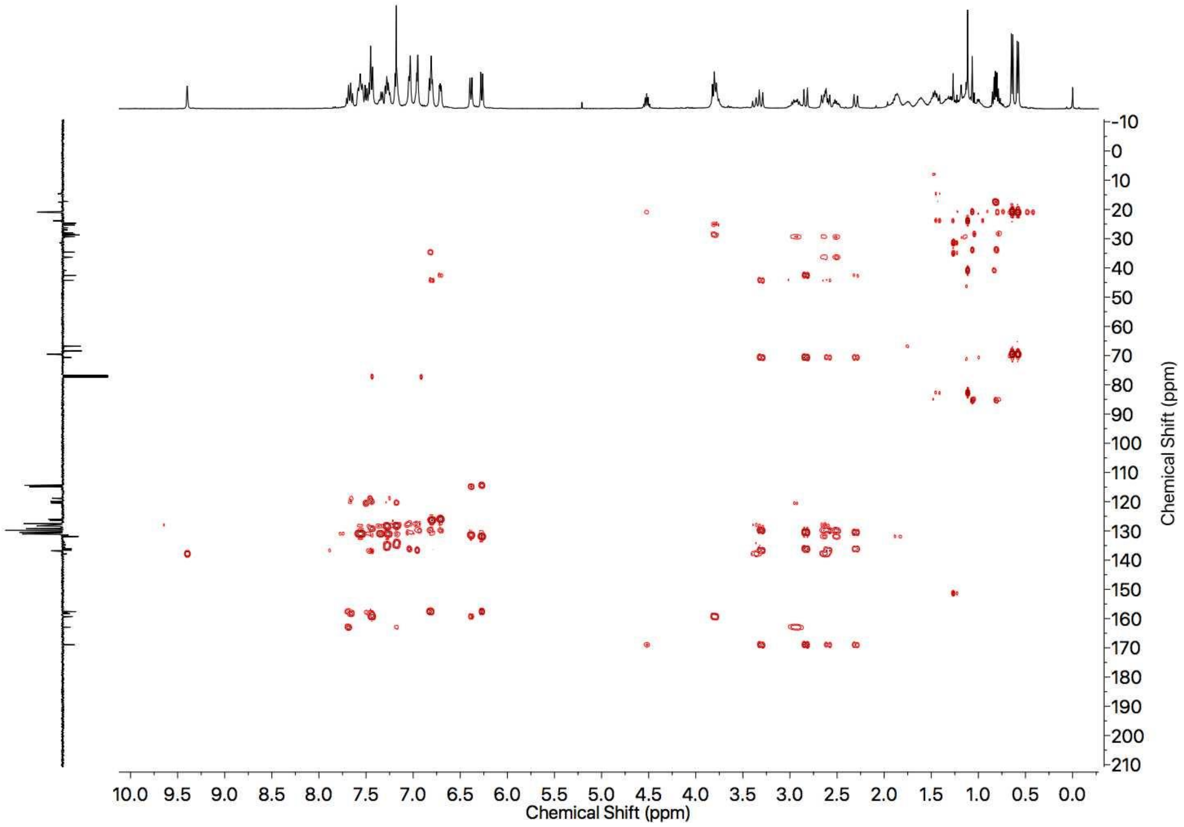

Figure S27: HMBC NMR ($CDCl_3$) of (R_{mp})-**5**.

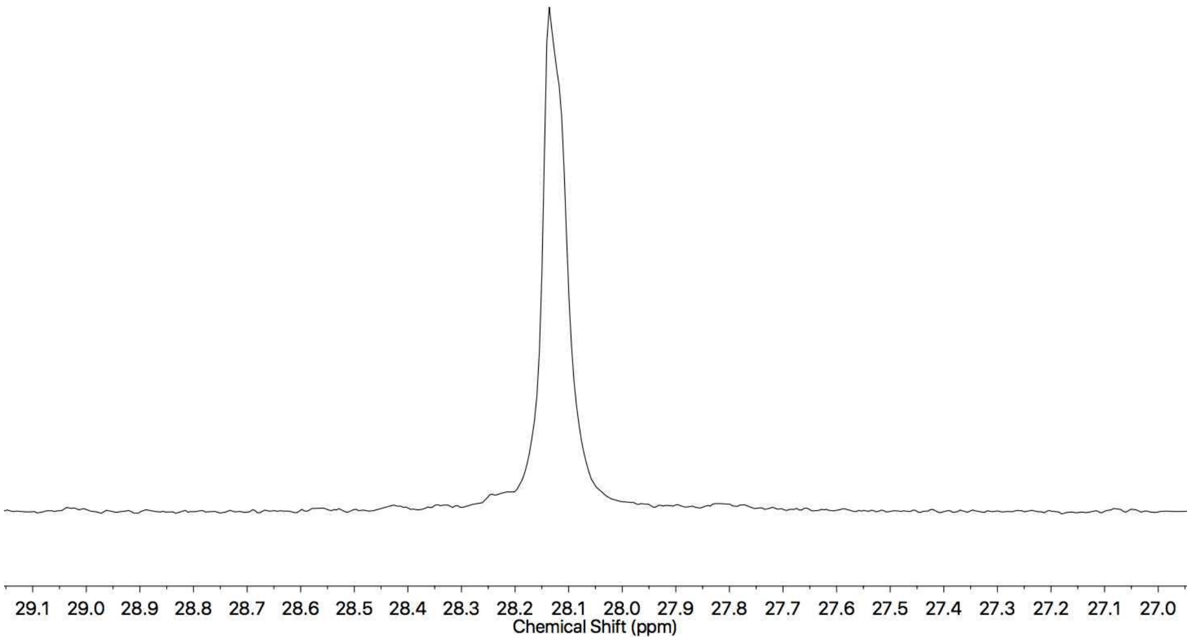

Figure S28: $^{31}P\{^1H\}$ NMR (202 MHz, $CDCl_3$) of (R_{mp})-**5**.

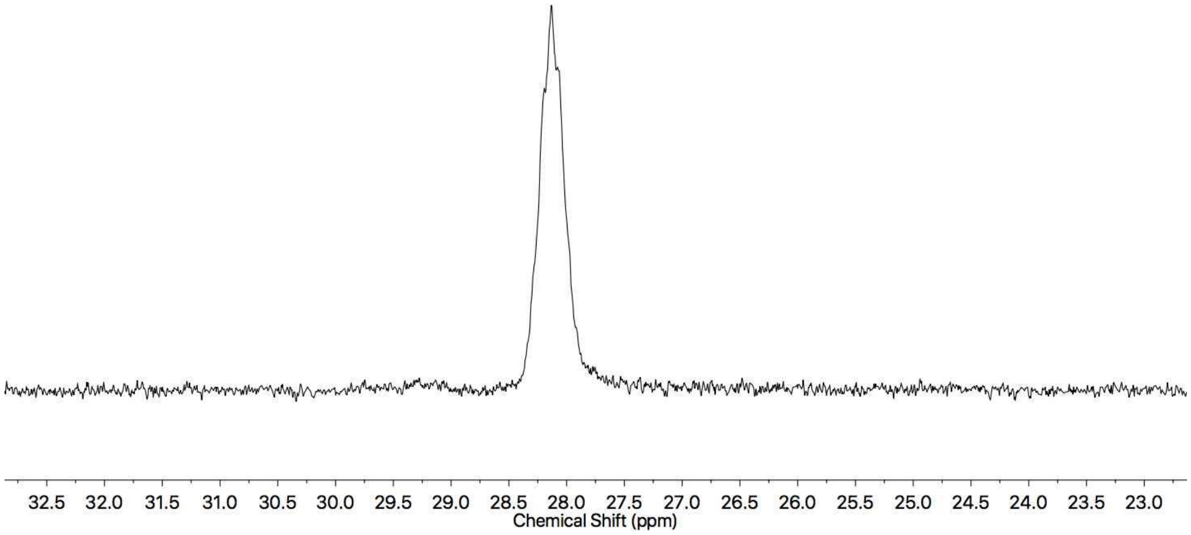

Figure S29: ^{31}P NMR (202 MHz, $CDCl_3$) of (R_{mp})-**5**.

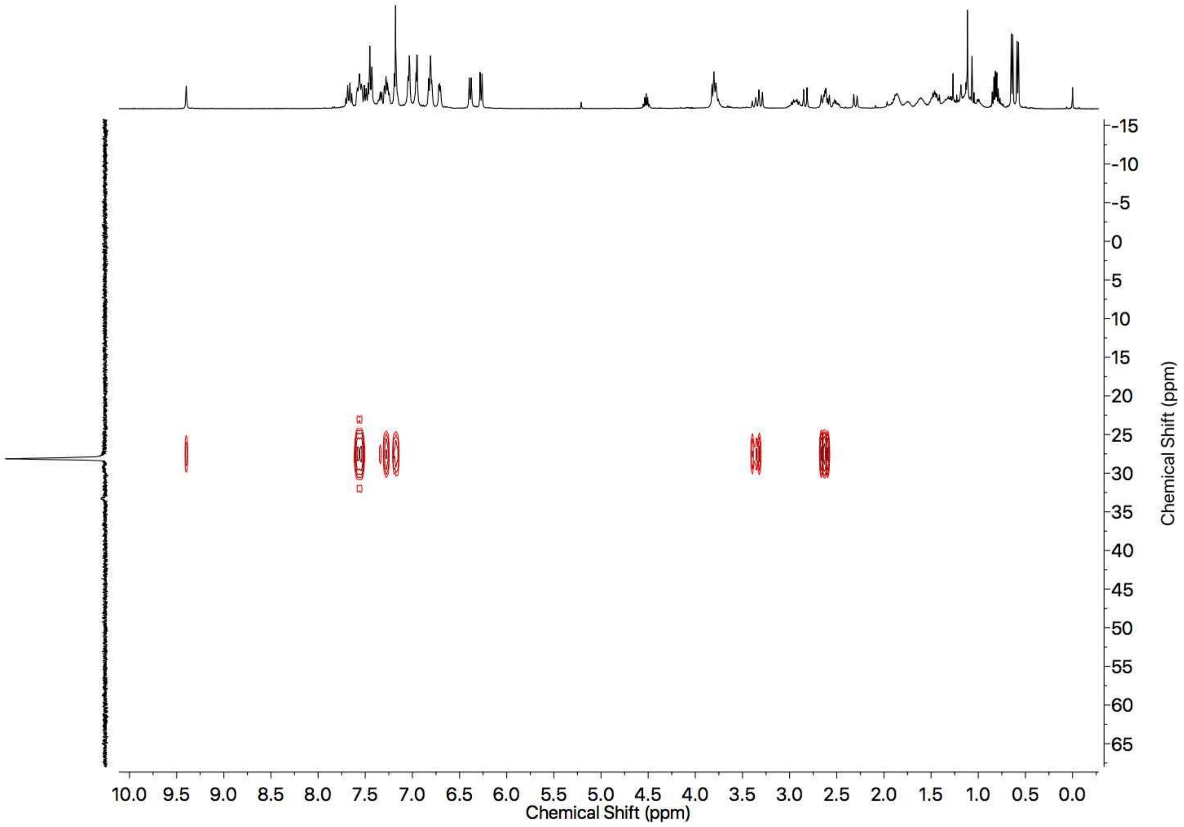

Figure S30: ^{31}P-^{1}H HMBC NMR ($CDCl_3$) of (R_{mp})-**5**.

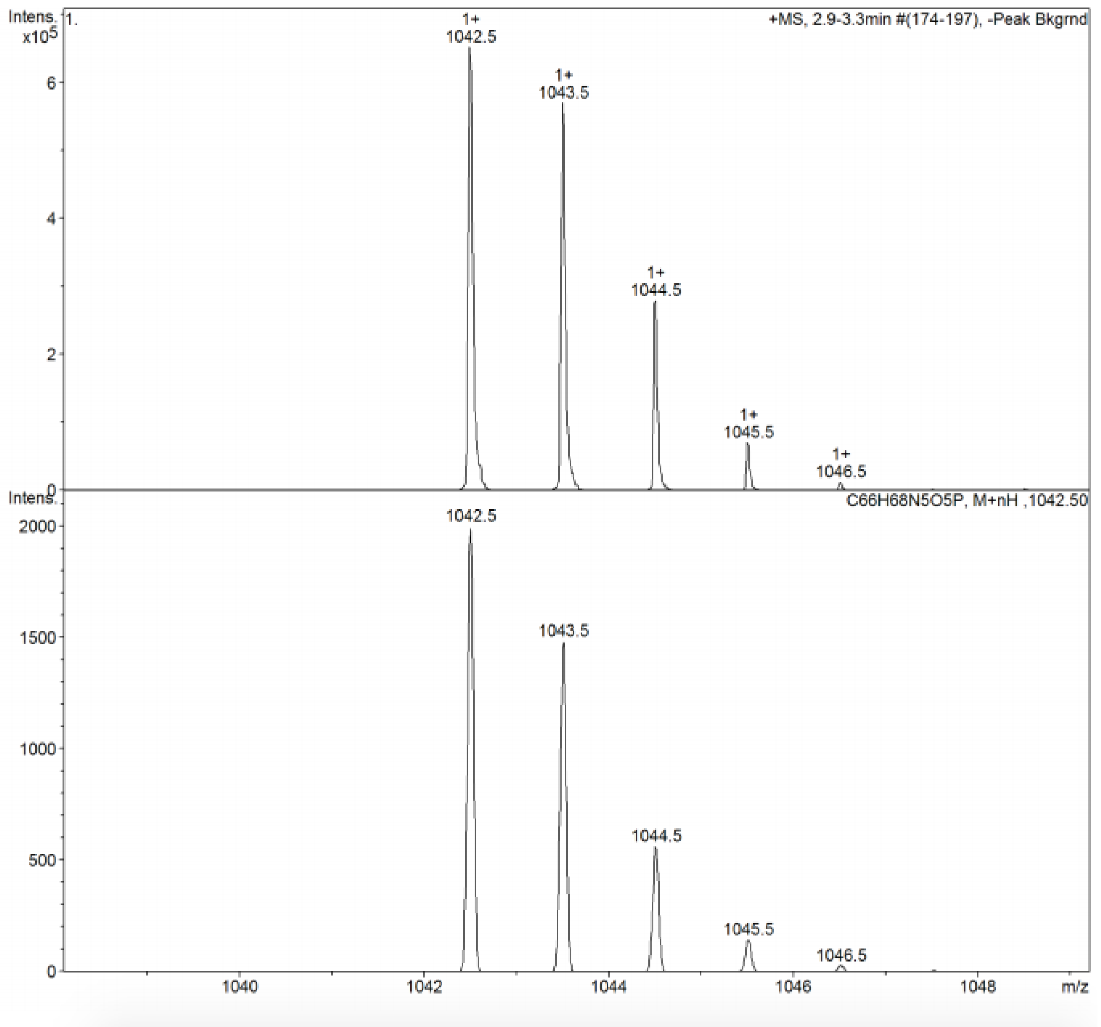

Figure S31: HR-ESI-MS of (R_{mp})-**5**. m/z 1042.5 [M + H]$^+$ (calc. m/z for $C_{66}H_{69}N_5O_5P$ 1042.5).

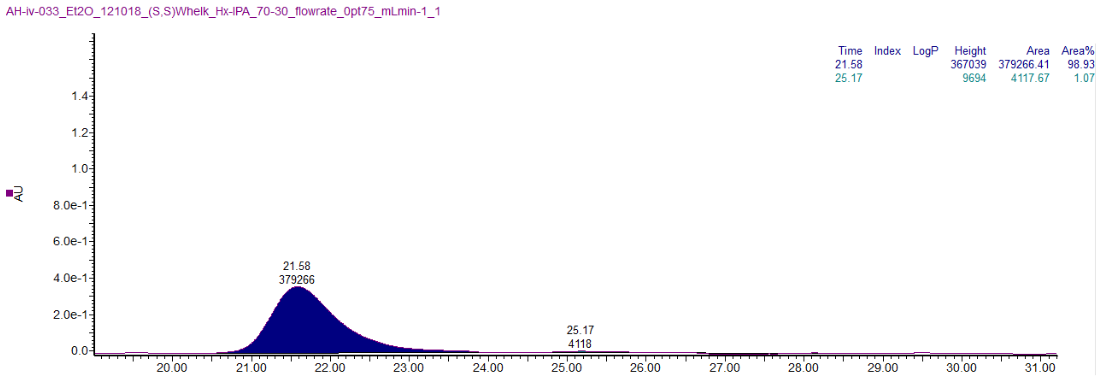

Figure S32: Chiral Stationary Phase HPLC ((*S*,*S*)Whelk, isocratic *n*-hexane-isopropanol 70 : 30, 303 K, load solvent Et_2O, 5 µL injection, flowrate 0.75 mLmin^{-1}) of 99 : 1 *er* (R_{mp})-**5**. Retention times (min): (R_{mp})-**5** 21.6, (S_{mp})-**5** 25.2.

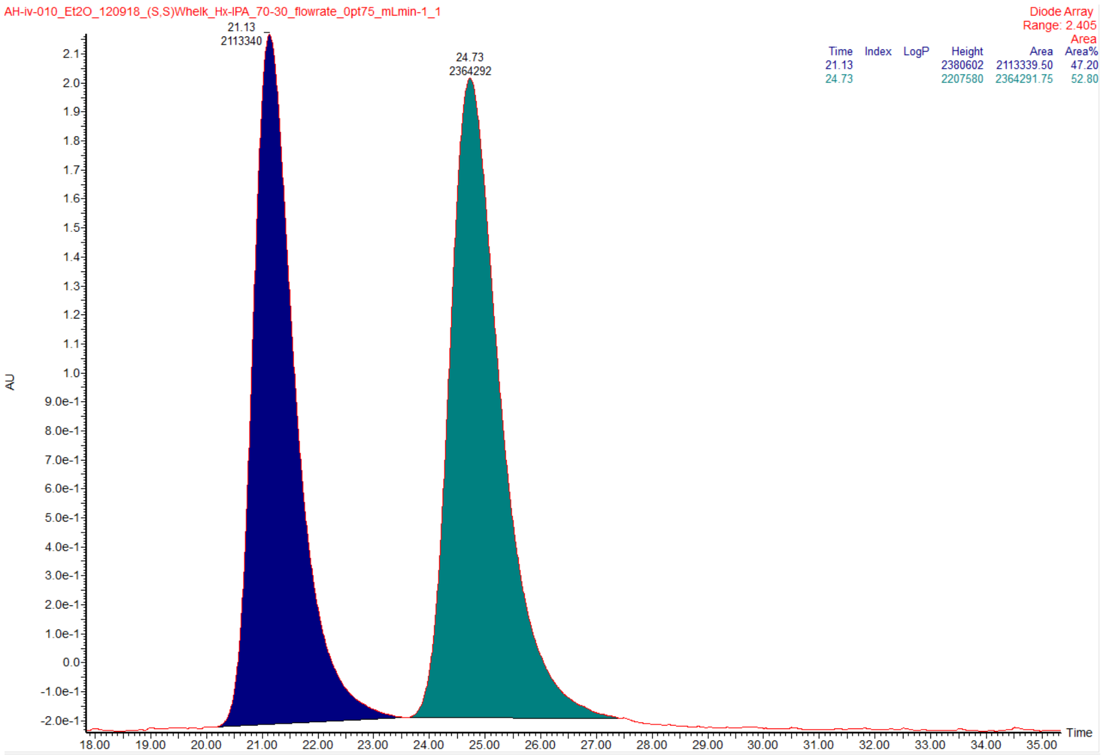

Figure S33: Chiral Stationary Phase HPLC ((*S*,*S*)Whelk, isocratic *n*-hexane-isopropanol 70 : 30, 303 K, load solvent Et_2O, 5 µL injection, flowrate 0.75 $mLmin^{-1}$) of racemate **5**. Retention times (min): (R_{mp})-**5** 21.1, (S_{mp})-**5** 24.7.

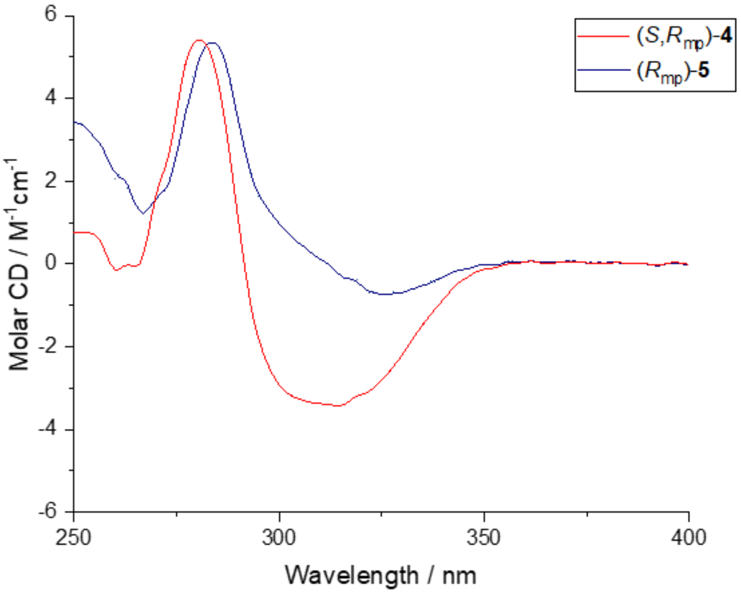

Figure S34: Circular Dichroism spectra of (*S*,R_{mp})-**4** (53.4 µM, 99 : 1 *er*) and (R_{mp})-**5** (66.2 µM, 99 : 1 *er*) at 293 K in $CHCl_3$.

Rotaxane (S_{mp})-**5**

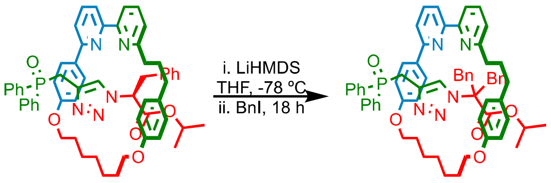

(S_C, S_{mp})-**4** (S_{mp})-**5** 63%, *ee* 98%

Scheme S3: Synthesis of (S_{mp})-**5**.

(*S*,S_{mp})-**4** (55.9 mg, 0.059 mmol, 1.0 eq.) was dissolved in anhydrous THF (3 mL) and transferred into a dry CEM MW vial under N_2. The solution was cooled to -78 °C and stirred for 20 min. Lithium bis(trimethylsilyl)amide (1 M in THF, 0.29 mL, 0.29 mmol, 5.0 eq.) was added to the reaction mixture and stirred for 10 min. Benzyl iodide (1 M in THF, 0.59 mL, 0.59 mmol, 10.0 eq.) was added. The reaction was allowed to warm to rt and stirred for 18 h. The reaction mixture was diluted with saturated NH_4Cl (30 mL) and extracted with CH_2Cl_2 (3 x 20 mL). The combined organic extracts were dried ($MgSO_4$) and solvent removed *in vacuo*. The reidue was purified by column chromatography (SiO_2, petrol-EtOAc 0→50%) to give a yellow foam product (S_{mp})-**5** (38.4 mg, 0.037 mmol, 63%, 1 : 99 *er*). Enantiopurity was assessed by chiral stationary phase HPLC. The absolute mechanical stereochemistry was inferred from that of the starting material and the stereolabel assigned based on our established approach[5] using the priority atoms indicated below.

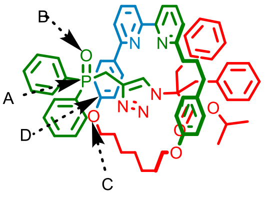

(S_{mp})-**5**

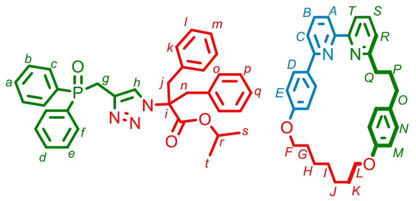

(S_{mp})-**5**

(S_{mp})-**5** same as (R_{mp})-**5** above

δ_H ($CDCl_3$, 400 MHz) 9.48 (1H, d, J = 1.6, H_h), 7.77 (1H, t, J = 8.1, H_S), 7.74 (1H, t, J = 8.1, H_B), 7.64 (4H, ddd, J = 8.2, 7.7, 2.3, H_c, H_f), 7.58 (1H, d, J = 8.1, H_T), 7.54 (1H, d, J = 7.8, H_A), 7.52 (2H, d, J = 8.5, H_D), 7.36 (4H, td, J = 7.6, 2.7, H_b, H_e), 7.29-7.23 (4H, m, H_a, H_d, H_R, H_C), 7.14-7.09 (3H, m, H_l, - H_m), 7.06-7.01 (3H, m, H_p, H_q), 6.90 (2H, d, J = 8.5, H_N), 6.89 (2H, dd, J = 7.2, 2.1, H_k), 6.79 (2H, dd, J = 7.1, 2.6, H_o), 6.49 (2H, d, J = 8.5, H_E), 6.35 (2H, d, J = 8.3, H_M), 4.60 (1H, sept., J = 6.2, H_r), 3.45 (1H, d, J = 15.0, H_g), 3.93-3.81 (4H, m, H_L, $H_{L'}$, H_F, $H_{F'}$), 3.39 (1H, d, J = 14.8, H_j), 3.09-2.97 (2H, m, H_Q, $H_{Q'}$), 2.92 (1H, d, J = 14.7, H_n), 2.74 (1H, d, J = 15.2, H_g), 2.68 (1H, br. d, J = 14.8, H_O), 2.63-2.54 (1H, m, $H_{j'}$), 2.38 (1H, d, J = 14.7, $H_{n'}$), 2.02-1.89 (3H, m, H_P, $H_{P'}$, $H_{O'}$), 1.87-1.77 (2H, m, H_J, $H_{J'}$), 1.75-1.62 (2H, m, H_G, $H_{G'}$), 1.61-1.48 (2H, m, H_K, $H_{K'}$), 1.47-1.36 (2H, m, H_I, $H_{I'}$), 0.94-0.86 (2H, m, H_H, $H_{H'}$), 0.71 (3H, d, J = 6.3, H_s), 0.66 (3H, d, J = 6.3, H_t).

δ_C ($CDCl_3$, 101 MHz) 169.1, 163.1, 159.4, 158.5, 158.1, 157.8, 157.7, 137.9 (d, J = 7.2), 137.0, 136.9, 136.5 (2C, d, J = 48.6), 132.0, 131.5, 131.2, 131.2, 131.1 (2C, d, J = 9.3), 130.6, 130.0, 129.9, 129.4, 128.4 (2C, d, J = 8.4), 128.3. (2C, d, J = 8.4), 127.7 (d, J = 6.4), 126.4, 126.1, 120.6, 120.4, 120.1, 119.0, 115.0, 114.6, 70.8, 69.7, 68.5, 66.9, 44.3, 42.7, 36.5, 34.7, 29.5, 28.9, 28.6, 28.1, 26.8 (d, J = 72.6), 25.5, 24.9, 24.0, 21.1, 21.0.

$\delta_{31P\{1H\}}$ ($CDCl_3$, 202 MHz) 28.1.

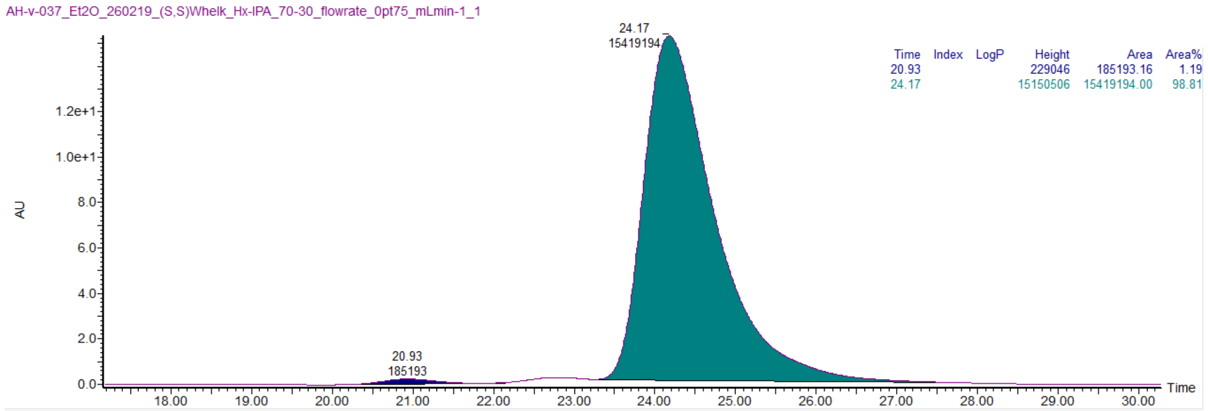

Figure S35: Chiral Stationary Phase HPLC ((*S*,*S*)Whelk, isocratic *n*-hexane-isopropanol 70 : 30, 303 K, load solvent Et_2O, 5 µL injection, flowrate 0.75 mLmin^{-1}) of 1 : 99 *er* (S_{mp})-**5**. Retention times (min): (R_{mp})-**5** 20.9, (S_{mp})-**5** 24.2.

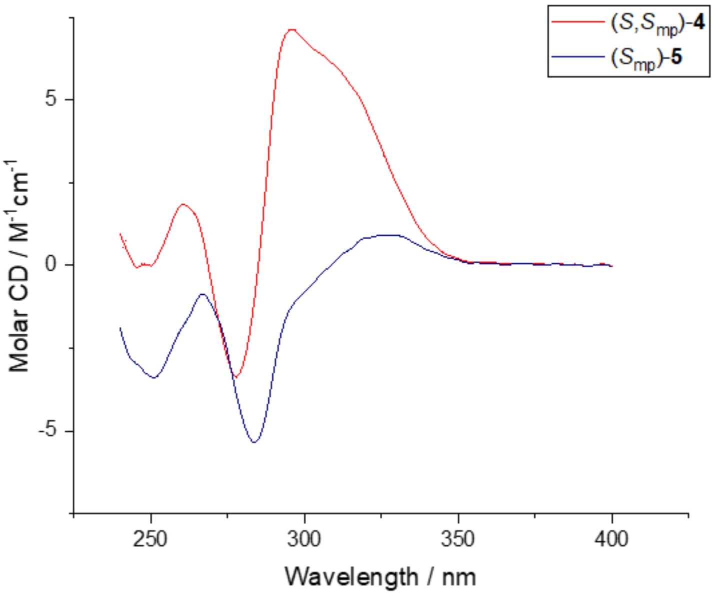

Figure S36: Circular Dichroism spectra of (*S*,S_{mp})-**4** (58.8 µM, (*S*,S_{mp})-**4** : (*R*,S_{mp})-**4** : (*S*,R_{mp})-**4**, 98.4 : 1.0 : 0.6) and (S_{mp})-**5** (54.7 µM, 99 : 1 *er*) at 293 K in $CHCl_3$.

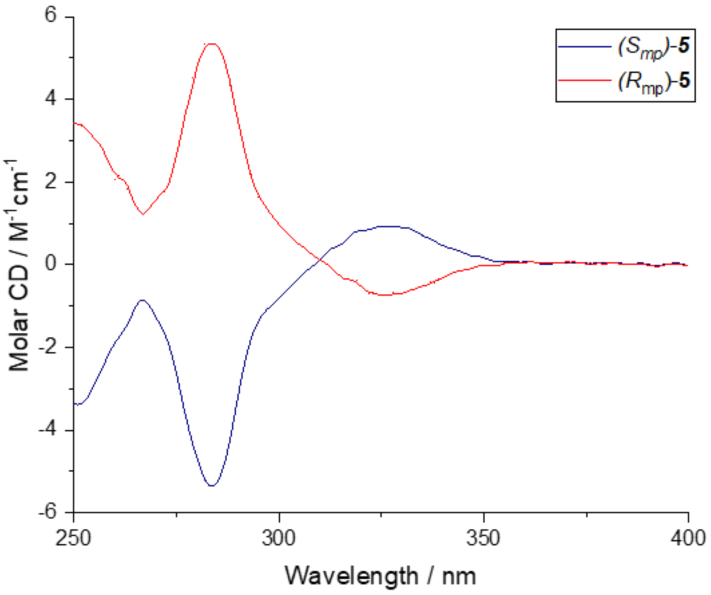

Figure S37: Circular Dichroism spectra of (R_{mp})-**5** (66.2 µM, 99 : 1 *er*) and (S_{mp})-**5** (54.7 µM, 99 : 1 *er*) at 293 K in $CHCl_3$.

Rotaxane [Au((R_{mp})-**6**)(Cl)]

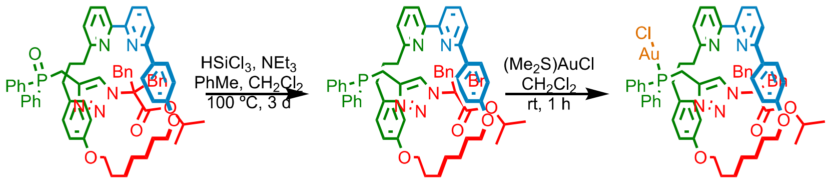

Scheme S4: Synthesis of [Au((R_{mp})-**6**)(Cl)].

A Young's tube was dried under reduced pressure with a heat gun three times and filled with nitrogen in-between each period, as per Schlenk technique. Under a high flow of nitrogen, anhydrous NEt_3 (3.0 mL, 21.5 mmol, 200 eq.) was added, followed by $HSiCl_3$ (1.1 mL, 10.7 mmol, 100 eq.) slowly. After 5 minutes, (R_{mp})-**5** (112 mg, 0.107 mmol, 1eq.) was transferred in anhydrous PhMe (5.5 mL) and then anhydrous CH_2Cl_2 (1.1 mL). The vessel was sealed and stirred at 100 °C for 3 days. After 3 days the solution was cooled to rt, washed with NaOH (1 M, 40 mL) and extracted in CH_2Cl_2 (4 x 30 mL). The combined organic phases were dried ($MgSO_4$), and the solvent removed *in vacuo*. This work up was repeated 4 times until NEt_3.HCl was absent from the ^{1}H NMR, yielding an orange oil (R_{mp})**-6** (76.2 mg, 0.074 mmol, 69%).

A dry vial was charged with (Me_2S)AuCl (22.1 mg, 0.074 mmol, 1.0 eq.) and (R_{mp})-**6** was transferred in anhydrous CH_2Cl_2 (1.5 mL). The solution was stirred at rt for 1 h. The solution was filtered through Celite® and eluted with Et_2O. The solvent was removed *in vacuo*. The residue was purified by column chromatography (SiO_2, petrol-Et_2O 0→100%) to give [Au((R_{mp})-**6**)(Cl)] as a white foam (86.2 mg, 0.068 mmol, 93%), the enantiopurity of which (*er* = 99 : 1) was inferred from the enantiopurity of the (R_{mp})-**5** starting material and the stereolabel assigned based on our established approach[5] using the priority atoms indicated below.

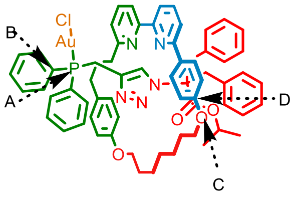

[Au((R_{mp})-**6**)Cl]

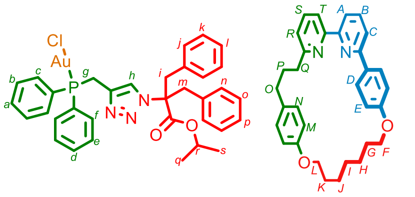

[Au((R_{mp})-**6**)Cl]

δ_H ($CDCl_3$, 400 MHz) 9.82 (1H, d, *J* = 2.3, H_h), 7.86 (1H, t, *J* = 7.8, H_S), 7.77 (1H, t, *J* = 7.7, H_B), 7.74 (2H, dd, *J* = 11.9, 5.0, H_f), 7.69 (1H, d, *J* = 7.7, H_T), 7.57 (1H, dd, *J* = 7.5, 0.7, H_A), 7.52 (1H, dd, *J* = 7.9, 0.7, H_C), 7.49 (1H, ddt (dq), *J* = 7.2, 1.3, H_d), 7.44 (2H, td, *J* = 7.9, 2.5, H_e), 7.37 (2H, ddd, *J* = 12.7, 7.9, 1.5, H_c) 7.33 (2H, d, *J* = 8.9, H_D), 7.31 (1H, d, *J* = 7.7, H_R), 7.29-7.27 (1H, m, H_a), 7.19 (2H, td, *J* = 7.9, 2.6, H_b), 7.16-7.10 (3H, m, H_o, H_p), 7.07-7.03 (3H, m, H_j, H_l), 6.94 (2H, dd, *J* = 7.5, 1.7, H_M), 6.76-6.71 (2H, m, H_k), 6.61 (2H, d, *J* = 8.4, H_N), 6.31 (2H, d, *J* = 8.4, H_n), 6.15 (2H, d, *J* = 8.9, H_E), 4.60 (1H, sept., *J* = 6.3, H_r), 3.17 (1H, d, *J* = 14.8, H_i), 3.04 (1H, d, *J* = 14.8, H_m), 3.01 (1H, dd, *J* = 14.8, 13.3, H_g), 2.94-2.85 (1H), 2.79 (1H, dd, *J* = 14.8, 12.0, $H_{g'}$), 2.75-2.65 (1H), 2.59 (1H, d, *J* = 14.8, $H_{i'}$), 2.54-2.44 (1H), 2.28 (1H, d, *J* = 14.8, $H_{m'}$), 2.18-1.88 (3H), 1.78-1.03 (14H), 0.68 (3H, d, *J* = 6.4, H_q), 0.63 (3H, d, *J* = 6.4, H_s). It was not possible to unambiguously assign alkyl region of macrocycle (H_F-H_L, H_O-H_Q).

δ_C ($CDCl_3$, 101 MHz) 169.0, 162.3, 158.7, 158.5, 158.4, 157.9, 157.4, 137.9, 137.8, 137.1, 137.0, 136.6, 136.1, 134.4, 134.2, 133.3, 133.2, 132.0, 131.5, 131.5, 131.2, 131.1, 131.0, 130.9, 130.2, 129.9, 129.8, 129.6, 128.9, 128.8, 128.7, 128.6, 128.6, 127.7, 127.6, 126.2, 126.2, 121.8, 120.7, 120.4, 119.0, 114.5, 114.2, 70.6, 69.6, 68.0, 57.2, 65.9, 44.0, 42.8, 35.7, 34.2, 30.3, 29.5, 28.4, 28.2, 27.9, 25.4, 25.3, 25.0, 24.8, 20.8, 20.8, 15.3.

64 signals corresponding to all 53 environments (11 doublets coupling to ^{31}P)

$\delta_{31P\{1H\}}$ ($CDCl_3$, 202 MHz) 26.2.

δ_{31P} ($CDCl_3$, 202 MHz) 26.2 (1P, broad quin.d, *J* 12.6, 3.6).

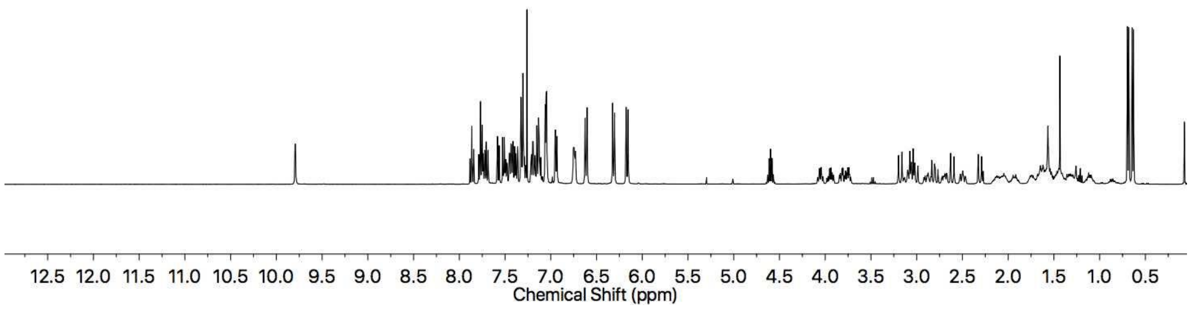

Figure S38: ^{1}H NMR (400 MHz, $CDCl_3$) of [Au((*R*$_{mp}$)-**6**)(Cl)].

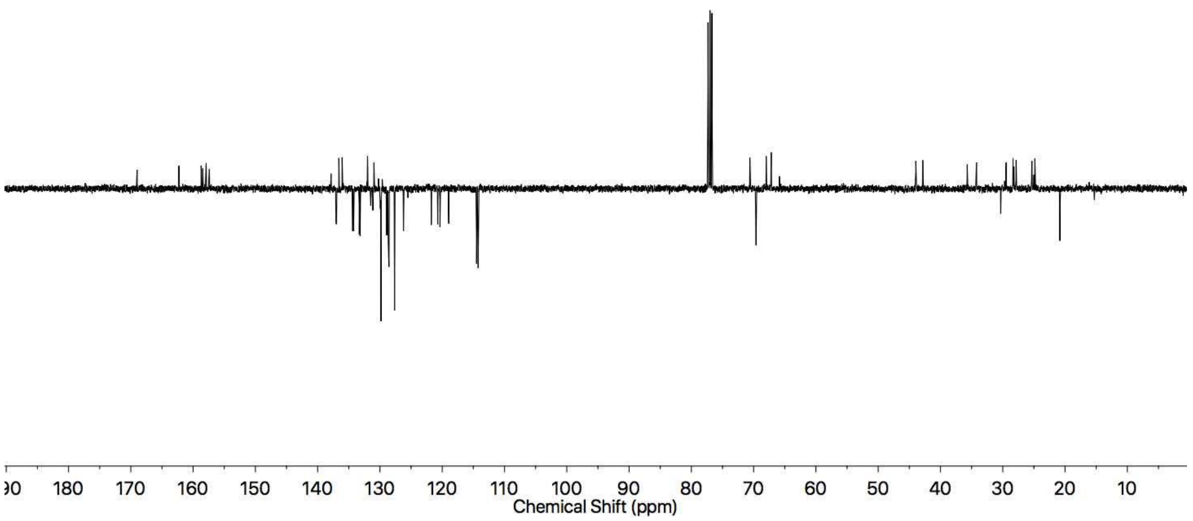

Figure S39: JMOD NMR (101 MHz, $CDCl_3$) of [Au((*R*$_{mp}$)-**6**)(Cl)].

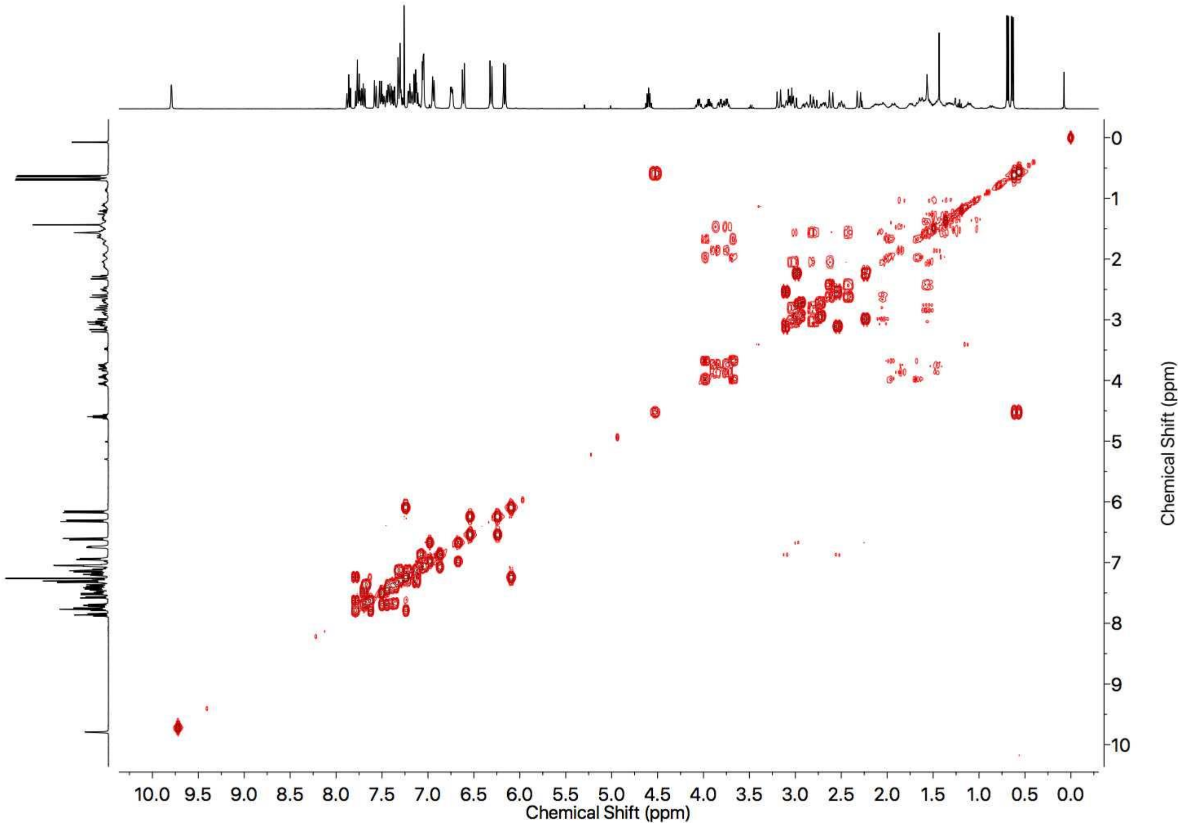

Figure S40: COSY NMR ($CDCl_3$) of [Au((*R*$_{mp}$)-**6**)(Cl)].

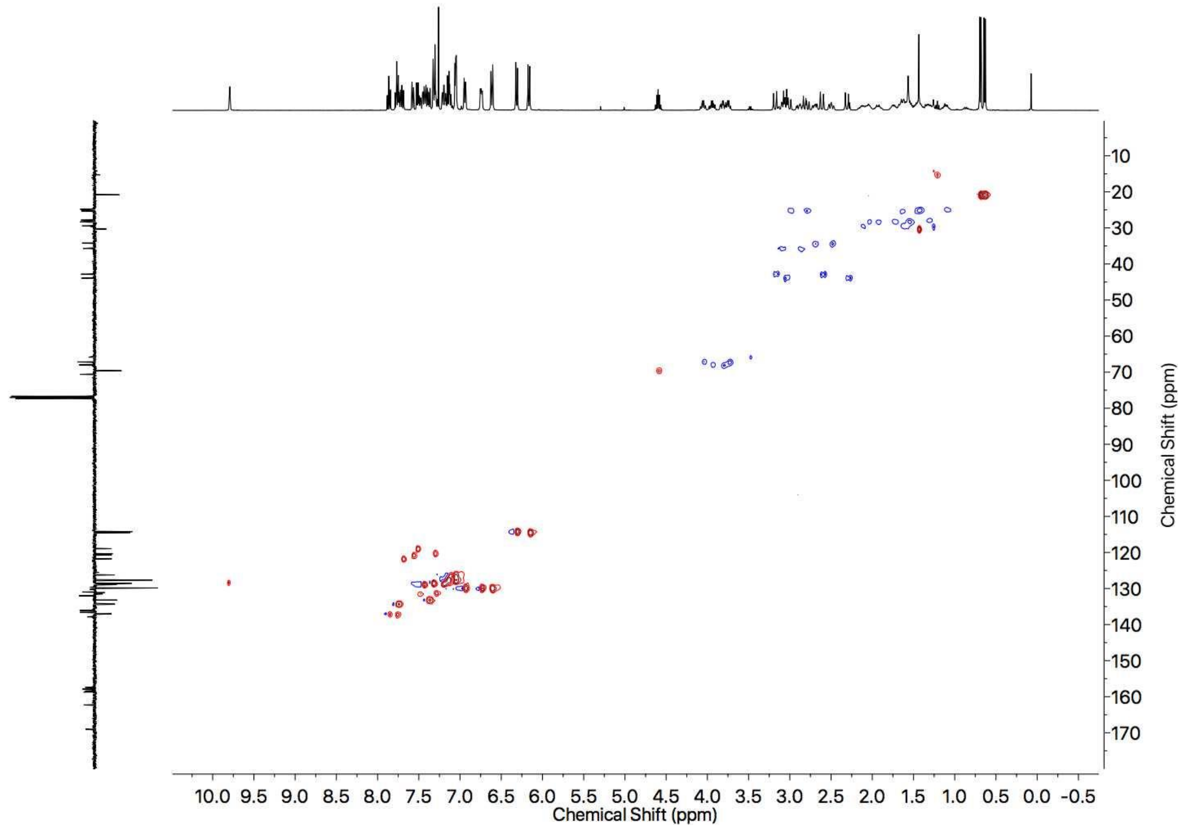

Figure S41: HSQC NMR ($CDCl_3$) of [Au((*R*$_{mp}$)-**6**)(Cl)].

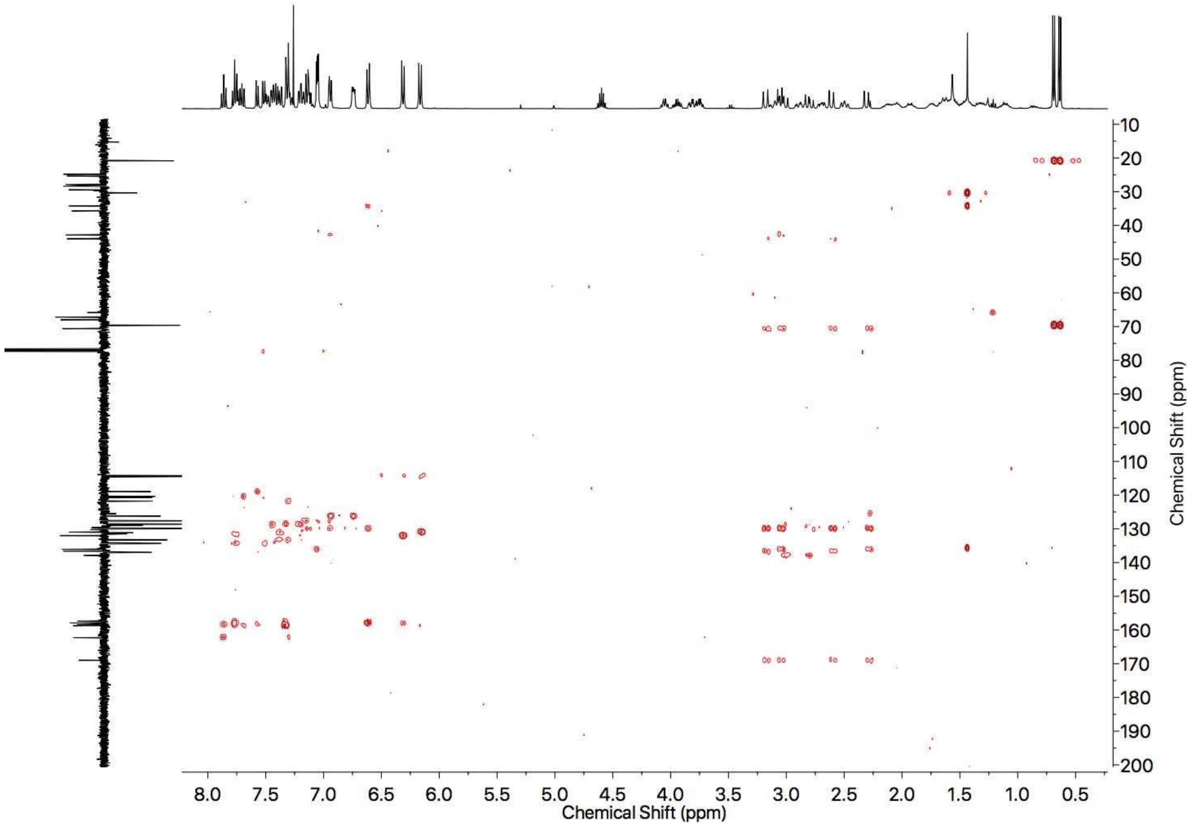

Figure S42: HMBC NMR ($CDCl_3$) of [Au((*R*$_{mp}$)-**6**)(Cl)].

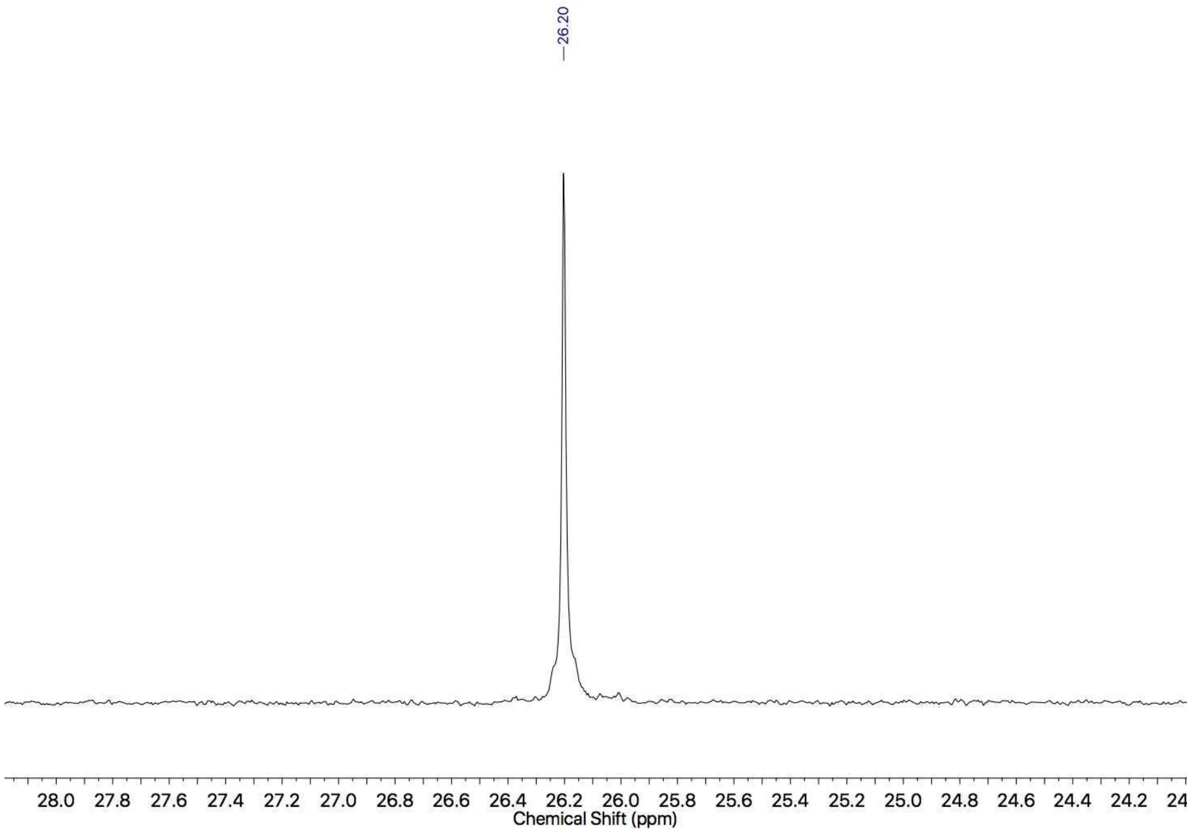

Figure S43: $^{31}P\{^1H\}$ NMR (202 MHz, $CDCl_3$) of [Au((*R*$_{mp}$)-**6**)(Cl)].

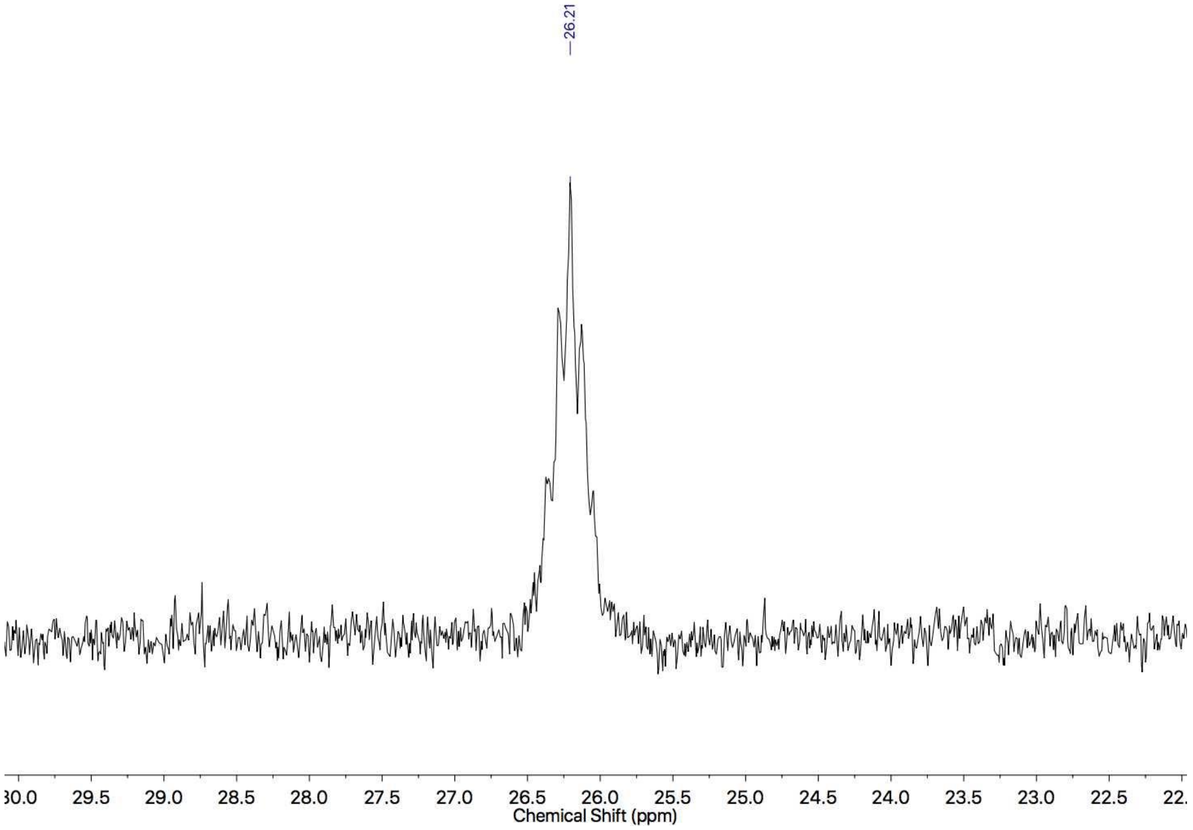

Figure S44: ^{31}P NMR (202 MHz, $CDCl_3$) of [Au((R_{mp})-**6**)(Cl)].

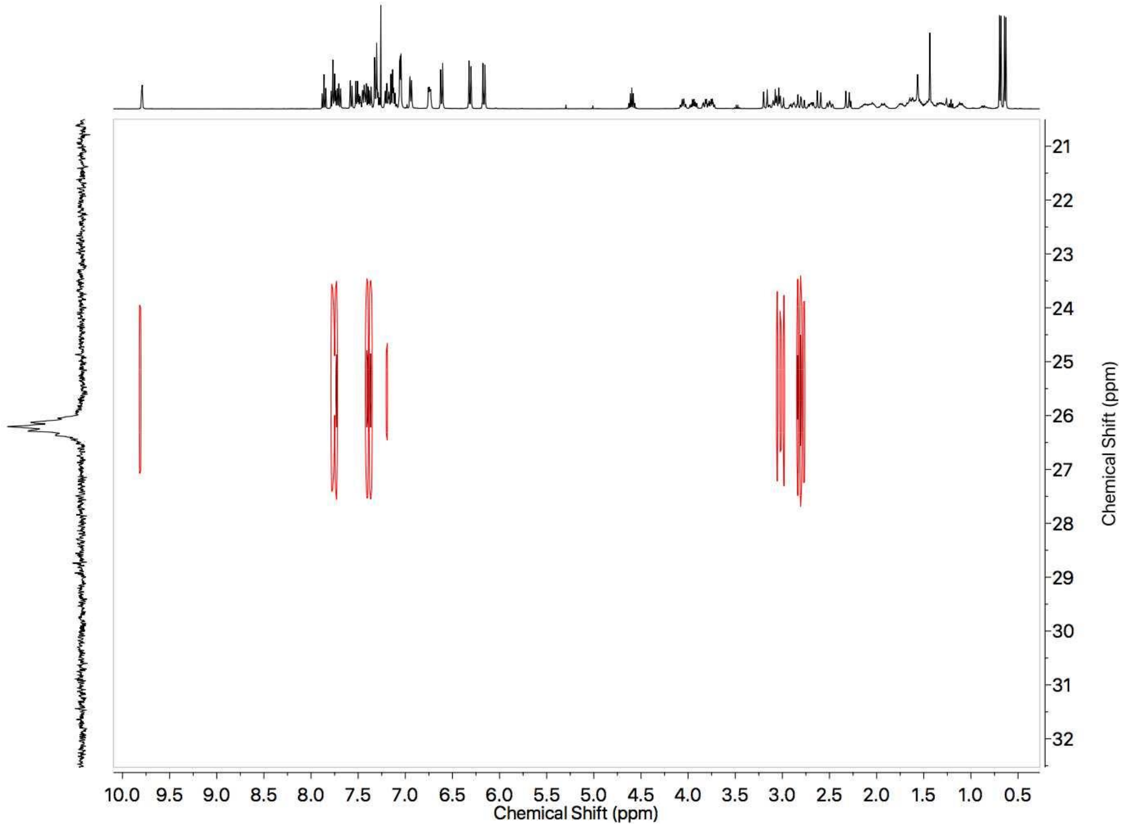

Figure S45: ^{1}H-^{31}P HMBC NMR ($CDCl_3$) of [Au((R_{mp})-**6**)(Cl)].

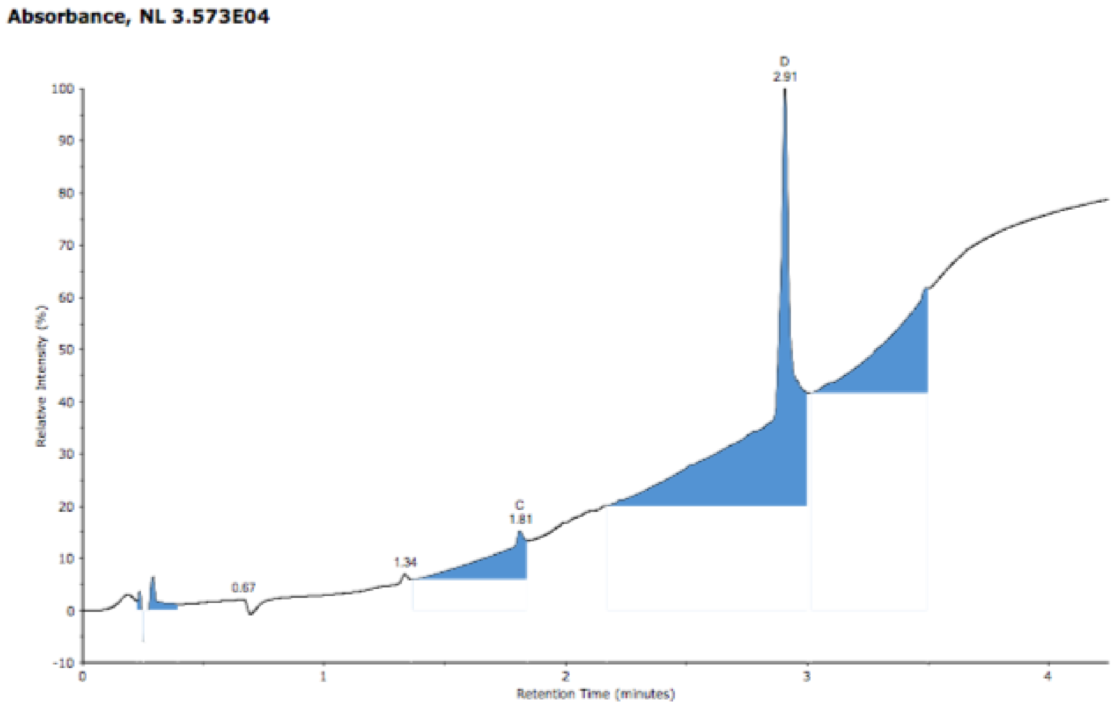

Figure S46: LC-ESI-MS chromatography of [Au((*R*$_{mp}$)-**6**)(Cl)].

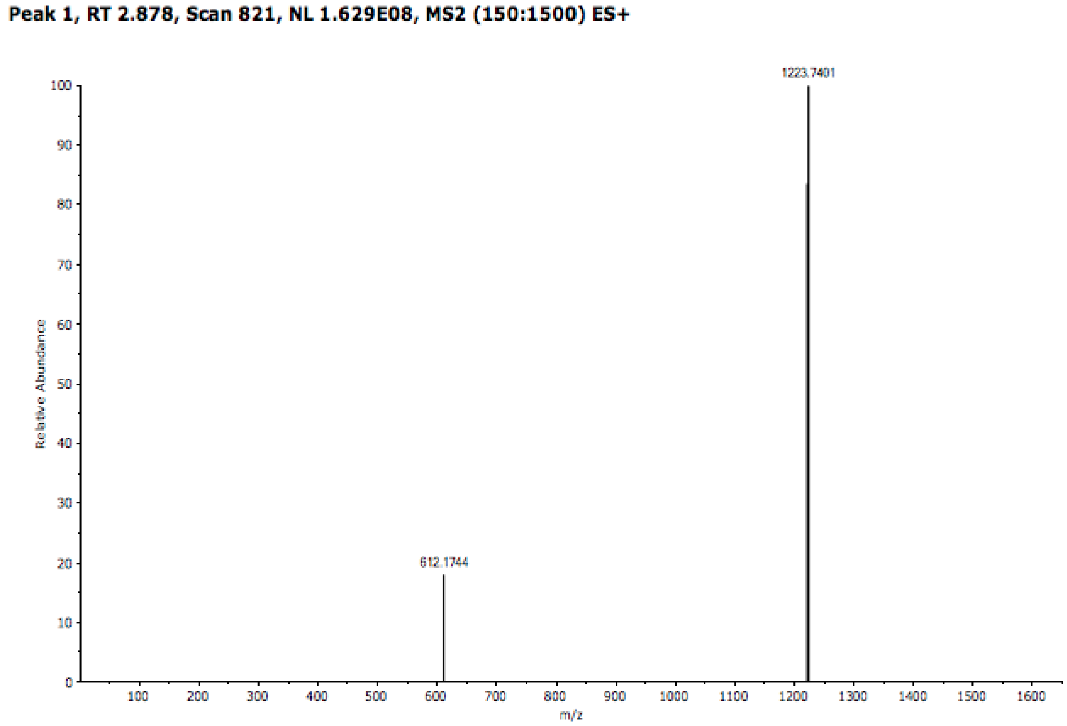

Figure S47: Low resolution LC-ESI-MS spectrum of [Au((*R*$_{mp}$)-**6**)(Cl)].

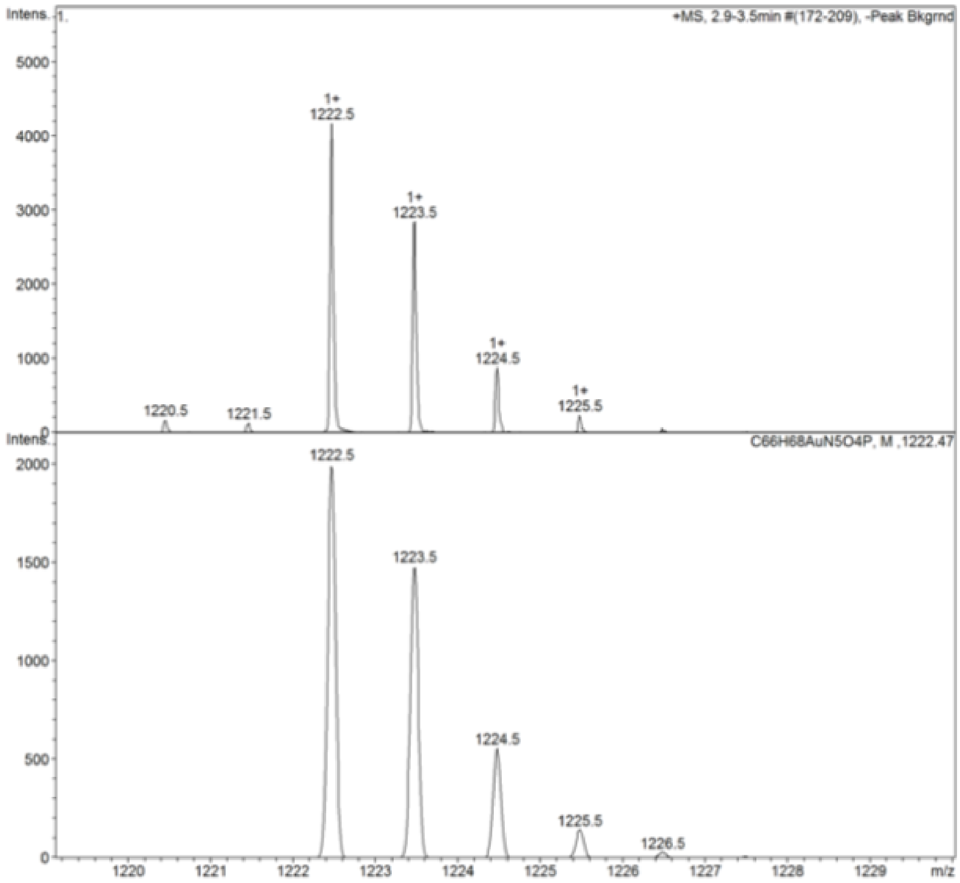

Figure S48: High resolution ESI-MS spectrum of [Au((R_{mp})-**6**)(Cl)]. $[M - Cl]^+$ 1222.5 (calc. m/z for $C_{66}H_{68}N_5O_4PAu$ 1222.47), $[M + H - Cl]^{2+}$ 611.7 (calc. m/z for $C_{66}H_{68}N_5O_4PAu$ 611.74).

Rotaxane [Au((S_{mp})-**6**)(Cl)]

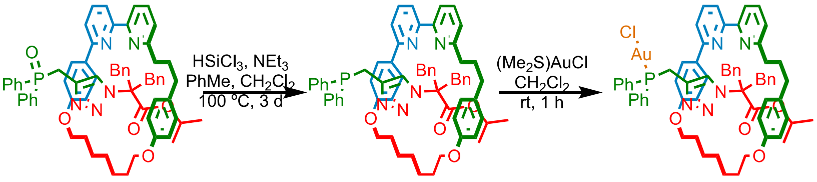

Scheme S5: Synthesis of [Au((S_{mp})-**6**)(Cl)].

A Young's tube was dried under reduced pressure with a heat gun three times and filled with nitrogen in-between each period, as per Schlenk technique. Under a high flow of nitrogen, anhydrous NEt_3 (2.5 mL, 17.9 mmol, 200 eq.) was added, followed by $HSiCl_3$ (0.9 mL, 8.95 mmol, 100 eq.) slowly. After 5 minutes, (S_{mp})-**5** (93.3 mg, 0.090 mmol, 1eq.) was transferred in anhydrous PhMe (4.6 mL) and then anhydrous CH_2Cl_2 (0.9 mL). The vessel was sealed and stirred at 100 ^oC for 3 days. After 3 days the solution was cooled to rt, washed with NaOH (1 M, 40 mL) and extracted in CH_2Cl_2 (4 x 30 mL). The combined organic phases were dried ($MgSO_4$), and the solvent removed *in vacuo*. This work up was repeated 4 times until NEt_3.HCl was absent from the ^1H NMR, yielding an orange oil (S_{mp})-**6** (85.3 mg, 0.083 mmol, 92%).

A dry vial was charged with (Me_2S)AuCl (24.4 mg, 0.083 mmol, 1.0 eq.) and (S_{mp})-**6** was transferred in anhydrous CH_2Cl_2 (2.0 mL). The solution was stirred at rt for 1 h. The solution was filtered through Celite® and eluted with Et_2O. The solvent was removed *in vacuo*. The residue was purified by column chromatography (SiO_2, petrol-Et_2O 0→100%) to give [Au((S_{mp})-**6**)(Cl)] as a white foam (70.5 mg, 0.056 mmol, 67%, 62% yield over two steps), the enantiopurity of which (*er* = 99 : 1) was inferred from the enantiopurity of the (S_{mp})-**5** starting material and the stereolabel assigned based on our established approach[5] using the priority atoms indicated below.

[Au((S_{mp})-**6**)(Cl)] same as [Au((R_{mp})-**6**)(Cl)] above

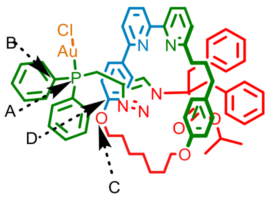

[Au((S_{mp})-**6**)Cl]

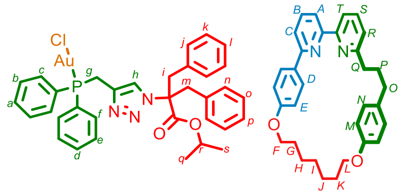

[Au((S_{mp})-**6**)Cl]

δ_H ($CDCl_3$, 400 MHz) 9.82 (1H, d, *J* = 2.3, H_h), 7.86 (1H, t, *J* = 7.8, H_S), 7.77 (1H, t, *J* = 7.7, H_B), 7.74 (2H, dd, *J* = 11.9, 5.0, H_f), 7.69 (1H, d, *J* = 7.7, H_T), 7.57 (1H, dd, *J* = 7.5, 0.7, H_A), 7.52 (1H, dd, *J* = 7.9, 0.7, H_C), 7.49 (1H, ddt (dq), *J* = 7.2, 1.3, H_d), 7.44 (2H, td, *J* = 7.9, 2.5, H_e), 7.37 (2H, ddd, *J* = 12.7, 7.9, 1.5, H_c) 7.33 (2H, d, *J* = 8.9, H_D), 7.31 (1H, d, *J* = 7.7, H_R), 7.29-7.27 (1H, m, H_a), 7.19 (2H, td, *J* = 7.9, 2.6, H_b), 7.16-7.10 (3H, m, H_o, H_p), 7.07-7.03 (3H, m, H_j, H_l), 6.94 (2H, dd, *J* = 7.5, 1.7, H_M), 6.76-6.71 (2H, m, H_k), 6.61 (2H, d, *J* = 8.4, H_N), 6.31 (2H, d, *J* = 8.4, H_n), 6.15 (2H, d, *J* = 8.9, H_E), 4.60 (1H, sept., *J* = 6.3, H_r), 3.17 (1H, d, *J* = 14.8, H_i), 3.04 (1H, d, *J* = 14.8, H_m), 3.01 (1H, dd, *J* = 14.8, 13.3, H_g), 2.94-2.85 (1H), 2.79 (1H, dd, *J* = 14.8, 12.0, $H_{g'}$), 2.75-2.65 (1H), 2.59 (1H, d, *J* = 14.8, $H_{i'}$), 2.54-2.44 (1H), 2.28 (1H, d, *J* = 14.8, $H_{m'}$), 2.18-1.88 (3H), 1.78-1.03 (14H), 0.68 (3H, d, *J* = 6.4, H_q), 0.63 (3H, d, *J* = 6.4, H_s). It was not possible to unambiguously assign the alkyl region of macrocycle (H_F-H_L, H_O-H_Q).

δ_C ($CDCl_3$, 101 MHz) 169.0, 162.3, 158.7, 158.5, 158.4, 157.9, 157.4, 137.9, 137.8, 137.1, 137.0, 136.6, 136.1, 134.4, 134.2, 133.3, 133.2, 132.0, 131.5, 131.5, 131.2, 131.1, 131.0, 130.9, 130.2, 129.9, 129.8, 129.6, 128.9, 128.8, 128.7, 128.6, 128.6, 127.7, 127.6, 126.2, 126.2, 121.8, 120.7, 120.4, 119.0, 114.5, 114.2, 70.6, 69.6, 68.0, 57.2, 65.9, 44.0, 42.8, 35.7, 34.2, 30.3, 29.5, 28.4, 28.2, 27.9, 25.4, 25.3, 25.0, 24.8, 20.8, 20.8, 15.3.

64 signals corresponding to all 53 environments (11 doublets coupling to ^{31}P).

$\delta_{31P\{1H\}}$ ($CDCl_3$, 202 MHz) 26.2.

δ_{31P} ($CDCl_3$, 202 MHz) 26.2 (1P, broad quin.d, *J* 12.6, 3.6).

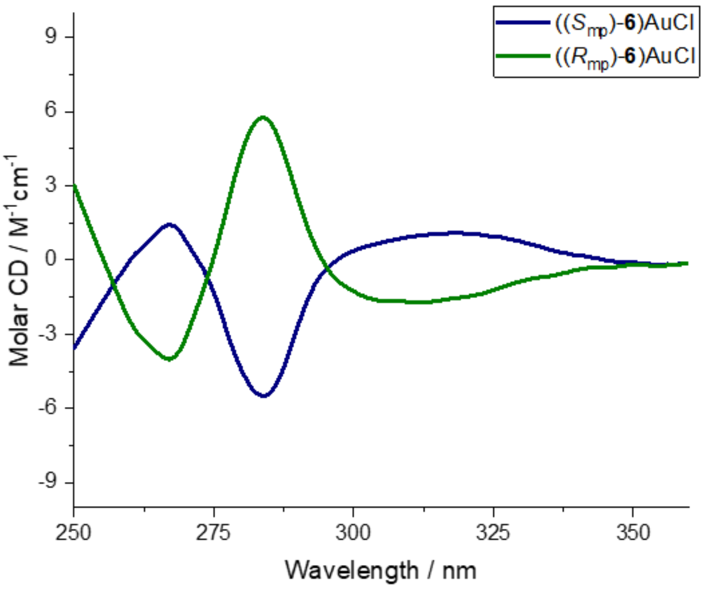

Figure S49: Circular Dichroism spectra of [Au((*R*mp)-**6**)(Cl)] (32.1 µM) and [Au((*S*mp)-**6**)(Cl)] (55.6 µM) at 293 K in $CHCl_3$. Both samples were 99 : 1 *er*.

^{1}H NMR Stack Plot Demonstrating the binding of Cu^I to the Rotaxane Framework

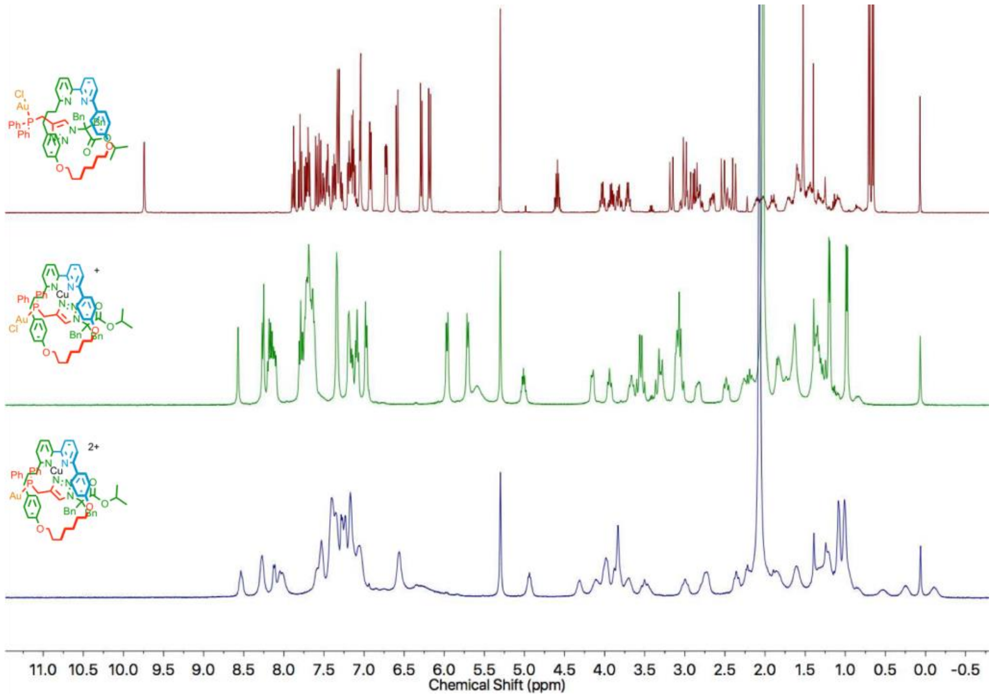

Figure S50: ^{1}H NMR stack plot ($CDCl_3$, 400MHz) of pre-catalyst [Au((*R*mp)-**6**)(Cl)] (top) showing triazole ^{1}H shift on addition of $[Cu(MeCN)_4]PF_6$ (middle), and further shift upon chloride abstraction on addition of $AgSbF_6$ to form the active catalyst (bottom). Counter-ions omitted for clarity.

3. Crystallographic Data: Assignment of Relative and Absolute Stereochemistry of (*S*,*R*$_{mp}$)-4 and (*S*,*S*$_{mp}$)-4

Rotaxane (*S*,*R*$_{mp}$)-4 SCXRD Data (XRAY_ SRmp4.cif)

Enantiopure single crystals of (*S*,*R*$_{mp}$)-**4** were grown from (*S*,*R*$_{mp}$)-**4** (diastereomerically pure, *er* 99 : 1, see **Figure S52**, **Figure S53**) by vapor diffusion of *n*-pentane into a saturated solution in CH_2Cl_2. Data was collected at 100 K using a Rigaku 007 HF diffractometer equipped with a HYPix6000 enhanced sensitivity detector. Cell determination, data collection, data reduction, cell refinement and absorption correction were performed with CrysAlisPro. The crystal structure was solved using Olex2 with SHELXT dual methods and refined against F^2 with SHELXL refinement package using anisotropic thermal displacement parameters for all non-hydrogen atoms. H atoms were placed in calculated position and refined using a riding model.[7]

Note on the assignment of the absolute stereochemistry of (*S*,*R*$_{mp}$)-4: SC-XRD analysis of (*S*,*R*$_{mp}$)-**4** allows the relative covalent and mechanical stereochemistry to be directly determined as (*S**,*R**$_{mp}$). Furthermore, the absolute stereochemistry of the azide-derived stereocenter is known to be (*S*) due to the known stereochemistry of the starting material, (S)-**1**. This allows us to unambiguously assign the absolute stereochemistry of the sample to be (*S*,*R*$_{mp}$)-**4**.

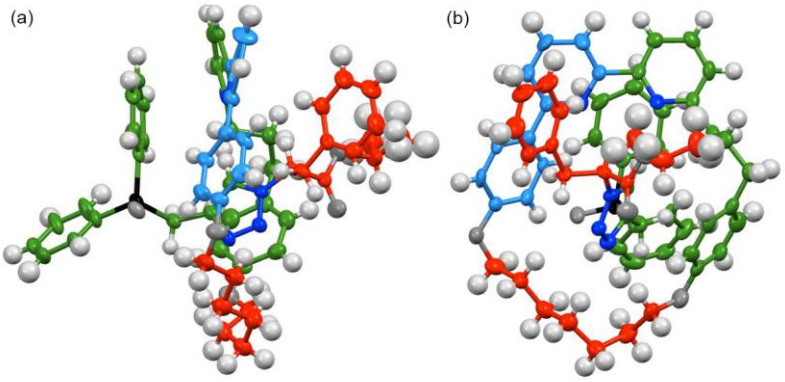

Figure S51: The SCXRD derived asymmetric unit of (*S*,*R*$_{mp}$)-**4** in thermal elipsoid (50%) representation from the (a) side and (b) the front.

Table S1. Crystal Structure Parameters for (*S*,R_{mp})-**4**.

Compound	(*S*,R_{mp})-**4**
Empirical formula	$C_{59}H_{62}N_5O_5P$
Formula weight	952.10
Temperature/K	100(2)
Crystal system	orthorhombic
Space group	$P2_12_12_1$
a/Å	12.69420(10)
b/Å	19.2864(2)
c/Å	20.6222(2)
α/°	90
β/°	90
γ/°	90
Volume/Å^3	5048.84(8)
Z	4
ρ_{calc}g/cm^3	1.253
μ/mm^{-1}	0.920
F(000)	2024.0
Crystal size/mm^3	0.05 × 0.05 × 0.05
Radiation	CuKα (λ = 1.54184)
2Θ range for data collection/°	6.274 to 140.93
Index ranges	-15 ≤ h ≤ 15, -23 ≤ k ≤ 23, -25 ≤ l ≤ 24
Reflections collected	96525
Independent reflections	9590 [R_{int} = 0.0620, R_{sigma} = 0.0295]
Data/restraints/parameters	9590/3/633
Goodness-of-fit on F^2	1.083
Final R indexes [I>=2σ (I)]	R_1 = 0.0462, wR_2 = 0.1146
Final R indexes [all data]	R_1 = 0.0495, wR_2 = 0.1166
Largest diff. peak/hole / e Å^{-3}	0.37/-0.25
Flack parameter	-0.008(8)

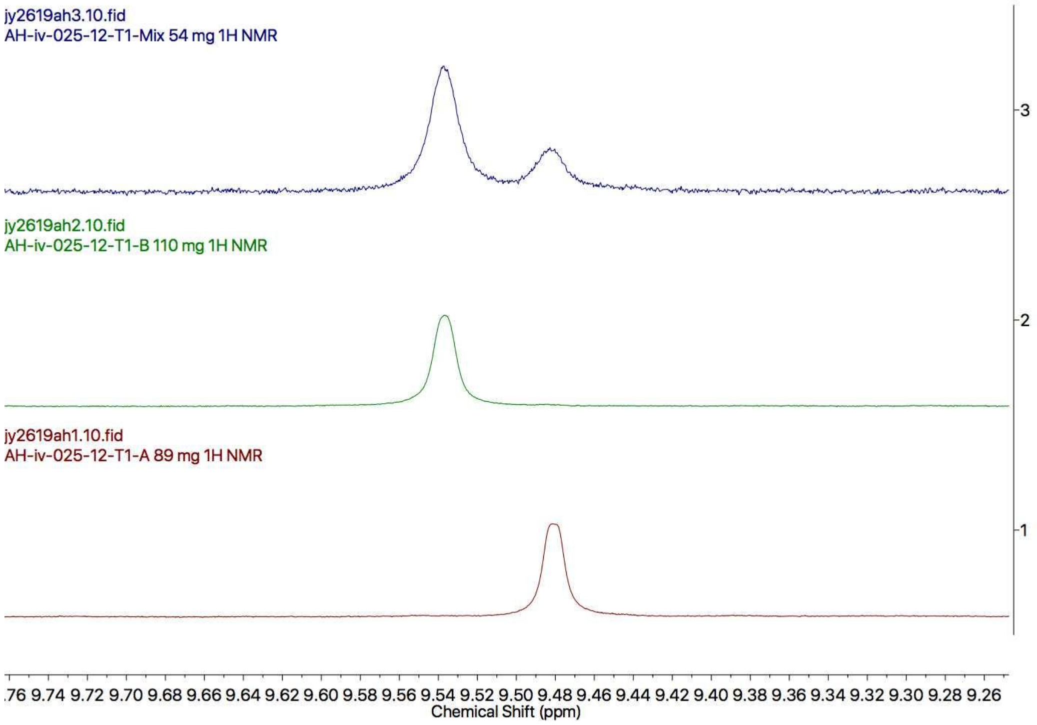

Figure S52: Partial ^{1}H NMR (400 MHz, $CDCl_3$) of a mixture of diastereomeric rotaxanes **4** (top), (*S*,*S*$_{mp}$)-**4** (middle) and the sample of (*S*,*R*$_{mp}$)-**4** (bottom) used for crystallisation to demonstrate the diastereomeric purity of the sample.

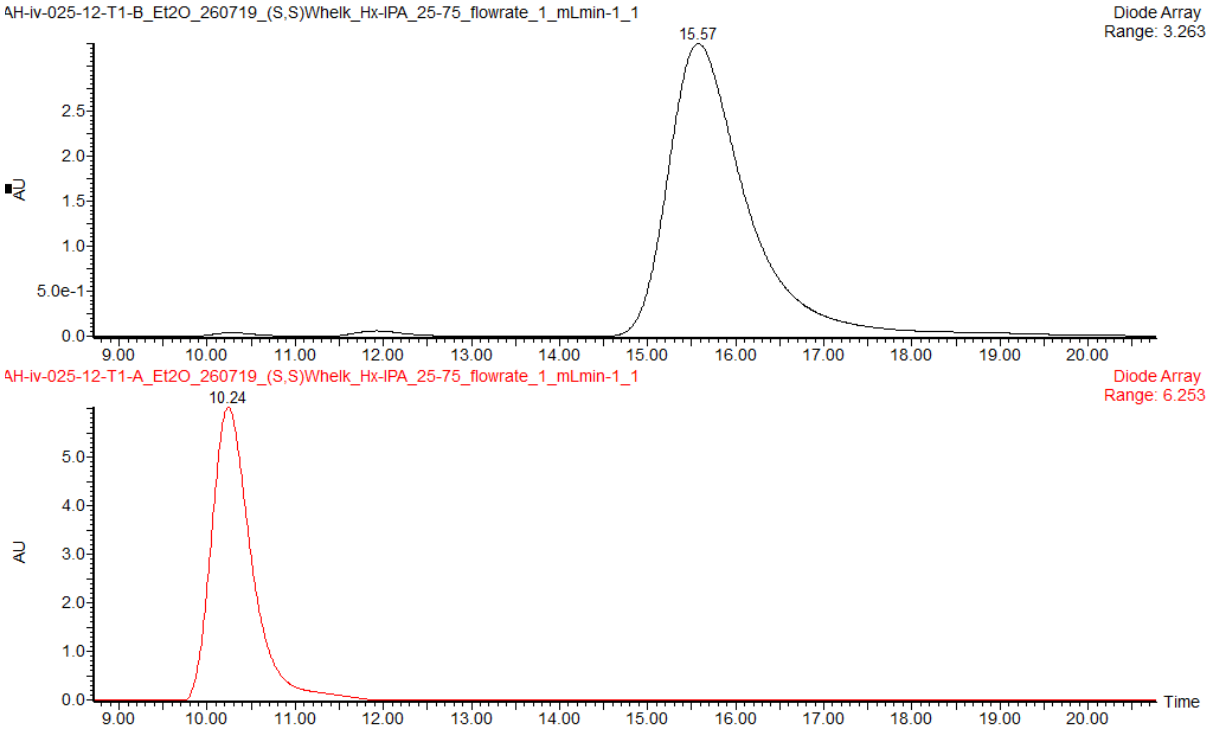

Figure S53: Chiral Stationary Phase HPLC ((*S*,*S*)Whelk column, isocratic *n*-hexane-isopropanol 25 : 75, 303 K, load solvent Et_2O, flowrate: 1.0 mLmin^{-1}) of (*S*,*S*$_{mp}$)-**4** (top) and the sample of (*S*,*R*$_{mp}$)-**4** (bottom) used for crystallisation, demonstrating the diastereo- and enantiopurity of the sample. Retention times (min): (*S*,*R*$_{mp}$)-**4** 10.2, (*R*,*S*$_{mp}$)-**4** 11.4, (*R*,*R*$_{mp}$)-**4** 12.1, (*S*,*S*$_{mp}$)-**4** 15.6.

Rotaxane (S,S_{mp})-4 SCXRD Data (XRAY_ SSmp4.cif)

Racemic single crystals of (S*,S*$_{mp}$)-**4** were grown from a diastereomerically pure, scalemic sample of (S,S_{mp})-**4** (*er* 88 : 12; **Figure S55**, **Figure S56**, **Figure S57**) by vapor diffusion of *n*-pentane into a saturated solution in Et_2O. Data was collected at 100 K using a FRE+ HF diffractometer equipped with a Saturn 724+ enhanced sensitivity detector. Cell determination, data collection, data reduction, cell refinement and absorption correction were performed with CrysAlisPro. The crystal structure was solved using Olex2 with SHELXT dual methods and refined against F^2 with SHELXL refinement package using anisotropic thermal displacement parameters for all non-hydrogen atoms. H atoms were placed in calculated position and refined using a riding model.[7]

Important Note: Although the bulk sample of (S,S_{mp})-**4** was of high purity, as judged by NMR and HPLC, SC-XRD analysis revealed additional electron density that could not be accounted for by solvent or other impurities. Ultimately, we found the data was consistent with 25% of the molecules in the unit cell being peroxide oxidation product **X**; modelling a 50% occupancy of an O_2 unit in which one O atom is situated 1.32 Å from the benzylic position of one of the two molecules in the asymmetric unit produced a solution that agrees well with the diffraction data. Importantly, this solution is also chemically reasonable – the position found to be oxidized is activated to radical pathways, although it is not clear if the oxidation takes place during the peroxide work up (*vide supra*), or during crystallization due to adventitious oxygen (**Scheme S6**). Regardless, oxidation product **X** is not observed as an impurity by HPLC, NMR or MS analysis of bulk samples of either diastereomer of **4**, or its derivatives, suggesting it is present only in trace quantities. Thus, it appears that **X** is incorporated selectively during the crystallization process, resulting in enrichment of the crystal obtained in this minor species. Although this complicates the SC-XRD analysis of the sample of (S*,S*$_{mp}$)-**4**, once this element of disorder is taken into consideration, the structure solution is of reasonable quality. Importantly, the data is consistent with the relative stereochemistry of the (S*,S*$_{mp}$)-**4** diastereomer being epimeric with that determined by SC-XRD for (S,R_{mp})-**4** (*vide supra*). Finally, the major enantiomer in the scalemic sample could be determined to be (S,S_{mp})-**4** based on the known covalent stereochemistry of azide (S)-**1** from which it is derived.

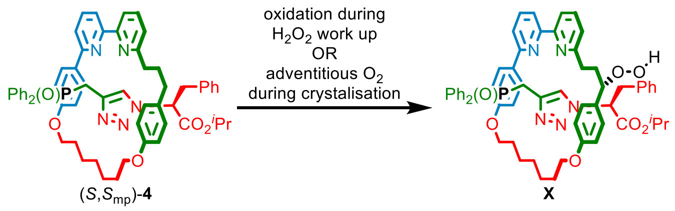

Scheme S6: Oxidation of (S,S_{mp})-**4** to give peroxide **X**, which is observed as an impurity by SC-XRD

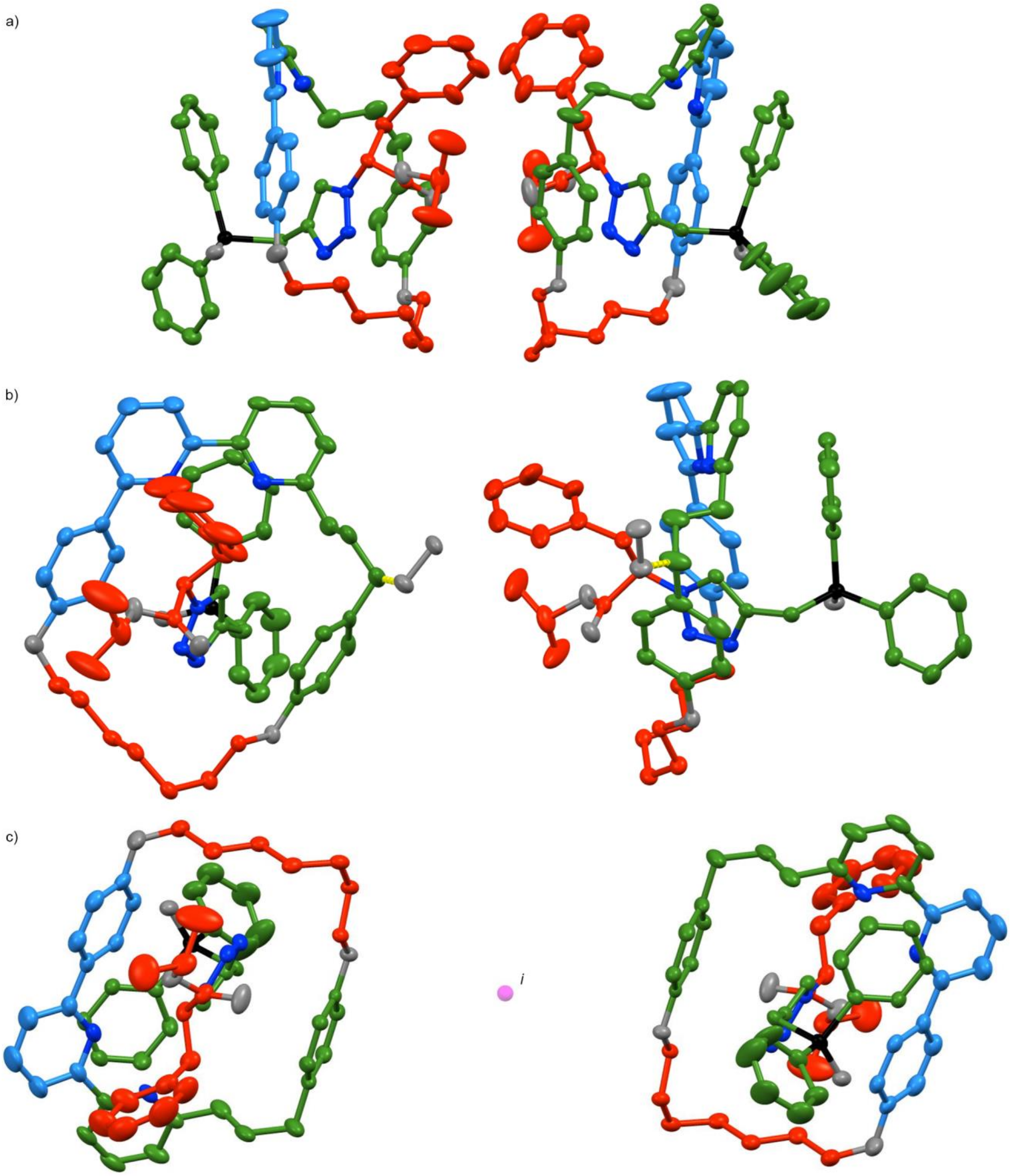

Figure S54: SCXRD structures of (S*,S*$_{mp}$)-**4** (crystallised from 88 : 12 *er* mixture, *n*-pentane vapour diffusion into Et_2O) in ellipsoid representation (50% probability, H atoms omitted for clarity). a) The asymmetric unit showing (S*,S*$_{mp}$)-**4** only (left hand molecule has 50% occupancy with peroxide **X**). b) Front and side views showing both (S*,S*$_{mp}$)-**4** and **X**, the latter being represented by the presence of an O_2 unit 1.32 Å from the benzylic C. c) An enantiomeric pair of (S*,S*$_{mp}$)-**4** related by an inversion centre, *i*, in the unit cell.

Table S2. X-ray diffraction data for (S*,S*$_{mp}$)-**4**

Compound	(S^*,S^*_{mp})-4
Empirical formula	$C_{118}H_{124}N_{10}O_{11}P_2$
Formula weight	1920.20
Temperature/K	100.0(2)
Crystal system	monoclinic
Space group	$P2_1/n$
a/Å	21.0723(2)
b/Å	18.6958(2)
c/Å	26.7361(2)
α/°	90
β/°	90.9780(10)
γ/°	90
Volume/Å^3	10531.51(17)
Z	4
ρ_{calc}g/cm^3	1.211
μ/mm^{-1}	0.107
F(000)	4080.0
Crystal size/mm^3	0.4 × 0.04 × 0.04
Radiation	Mo Kα (λ = 0.71075)
2Θ range for data collection/°	3.272 to 54.968
Index ranges	$-27 \le h \le 27$, $-24 \le k \le 24$, $-34 \le l \le 33$
Reflections collected	236269
Independent reflections	24147 [R_{int} = 0.0733, R_{sigma} = 0.0432]
Data/restraints/parameters	24147/207/1330
Goodness-of-fit on F^2	1.071
Final R indexes [I>=2σ (I)]	R_1 = 0.0770, wR_2 = 0.1780
Final R indexes [all data]	R_1 = 0.1072, wR_2 = 0.1916
Largest diff. peak/hole / e Å^{-3}	0.69/-0.51

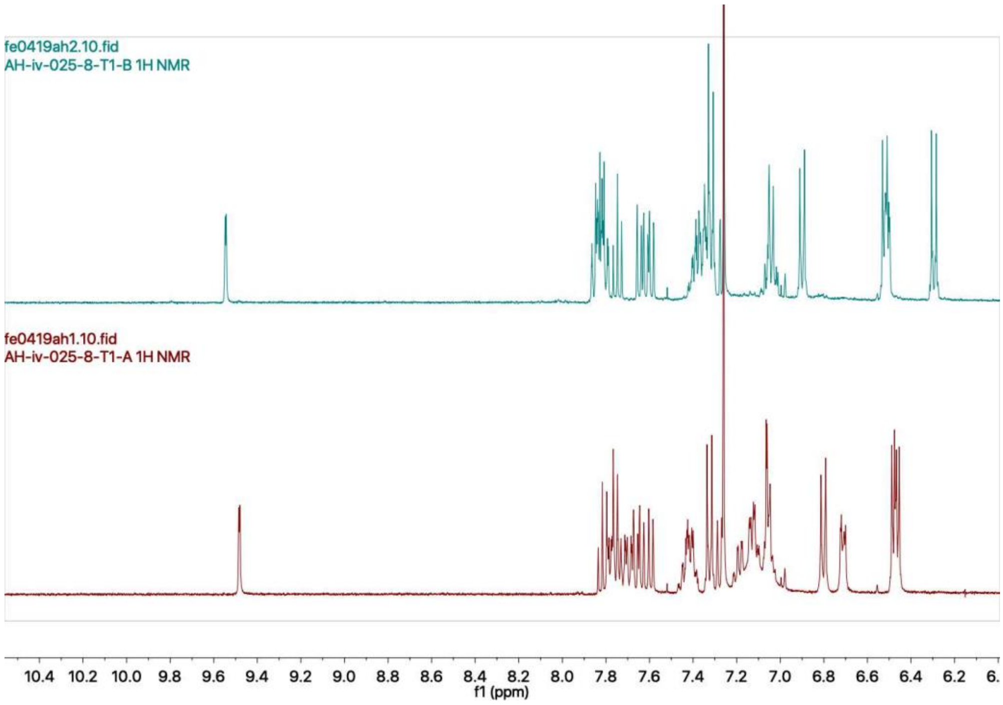

Figure S55: ^{1}H NMR (400 MHz, $CDCl_3$) of (*S*,*R*$_{mp}$)-**4** (bottom) and the sample of (*S*,*S*$_{mp}$)-**4** (top) used for crystallisation, demonstrating the diastereopurity of the sample.

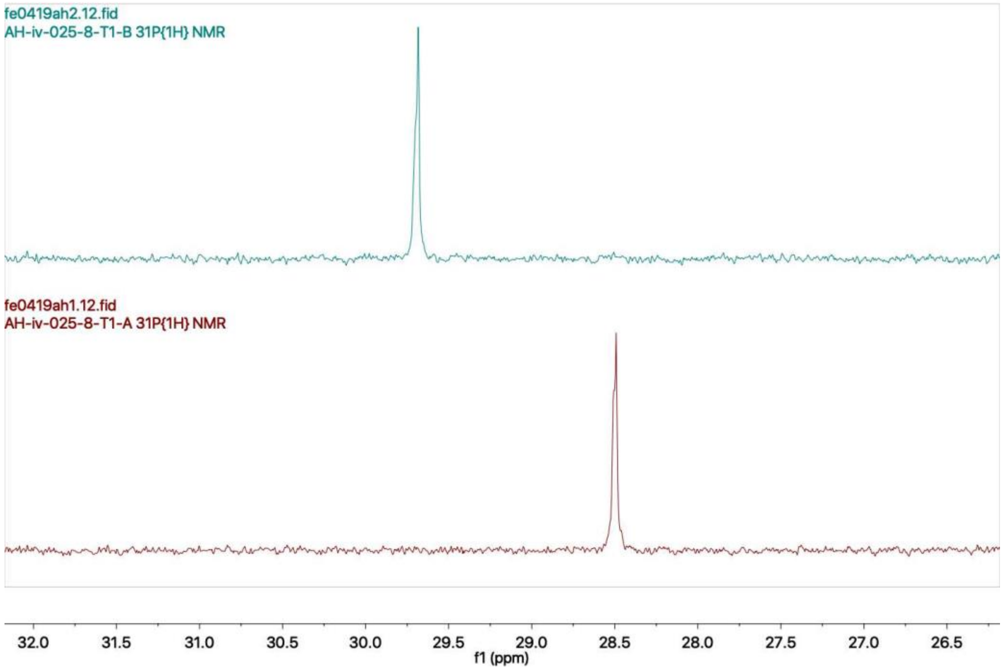

Figure S56: ^{31}P{^{1}H} (202 MHz, $CDCl_3$) NMR of (*S*,*R*$_{mp}$)-**4** (bottom) and the sample of (*S*,*S*$_{mp}$)-**4** (top) used for crystallisation, demonstrating the diastereopurity of the sample

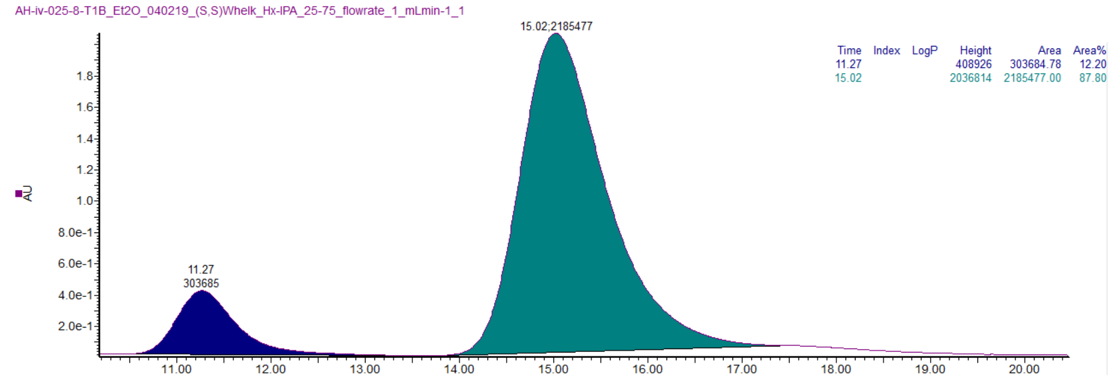

Figure S57: Chiral Stationary Phase HPLC ((*S*,*S*)Whelk column, isocratic *n*-hexane-isopropanol 25 : 75, 303 K, load solvent Et_2O, flowrate: 1.0 $mLmin^{-1}$) of the sample of (S,S_{mp})-**4** used for crystallisation demonstrating the enantiopurity of the sample to be 88 : 12 *er*. Retention times (min): (R,R_{mp})-**4** 11.3, (S,S_{mp})-**4** 15.0.

4. Synthesis of Cyclopropanation Substrates

Substituted 2-methyl-3-butyn-2-yl benzoate general procedure

Carboxylic acid (1.0 mmol, 1.0 eq.) was heated under reflux at 80 °C, under inert atmosphere, with $SOCl_2$ (0.58 mL, 8.0 mmol, 8.0 eq.) for 3 h. The reaction was cooled and excess $SOCl_2$ was removed *in vacuo*. The residue was cooled to 0 ˚C, and a solution of 2-methyl-3-butyn-2-ol (0.10 mL, 1.0 mmol, 1.0 eq.) and 4-(dimethylamino)pyridine (0.244 g, 2.0 mmol, 2.0 eq) in $CDCl_3$ (2.0 mL, 0.5 M concentration of 2-methyl-3-butyn-2-ol) was added dropwise. The resulting solution was stirred for 17 h at 35 ˚C. Solvent was removed under reduced pressure, and the residue was purified by column chromatography (SiO_2, petrol-Et_2O 0→5%).

2-methyl-3-butyn-2-yl benzoate, **7**[8]

Colourless oil (0.120 g, 0.63 mmol, 63%)

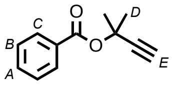

δ_H ($CDCl_3$, 400 MHz) 8.02 (2H, dd, *J* = 7.0, 2.0, H_C), 7.55 (1H, t, *J* = 7.5, H_A), 7.43 (2H, t, *J* = 7.0, H_B), 2.58 (1H, s, H_E), 1.83 (6H, s, H_D).

δ_C ($CDCl_3$, 101 MHz) 165.0, 133.0, 131.0, 129.7, 128.4, 84.8, 72.7, 72.4, 29.2.

GCMS [$M^{+\cdot}$] m/z 188.2.

HR-EI-MS m/z 188.0831 $[M]^{+\cdot}$ (calc. m/z for $C_{12}H_{12}O_2$ 188.0832).

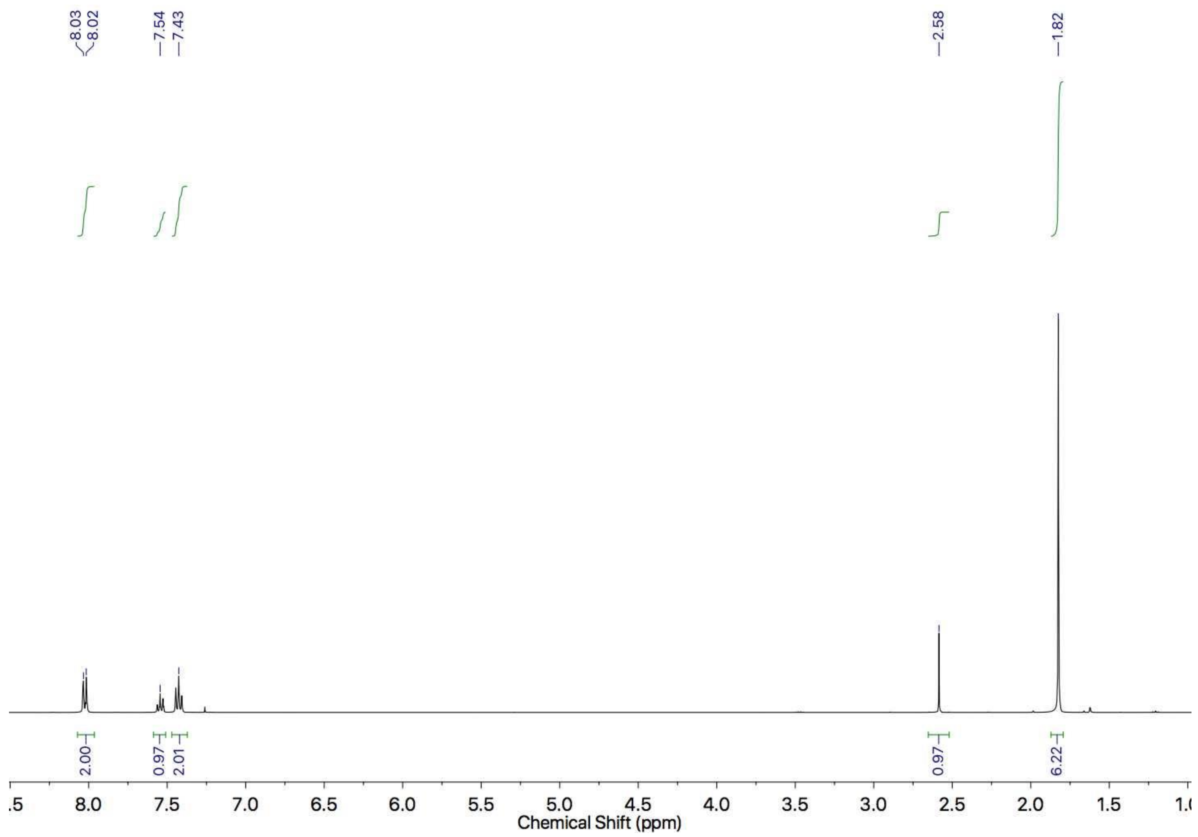

Figure S58: ^{1}H NMR (400 MHz, $CDCl_3$) of **7**.

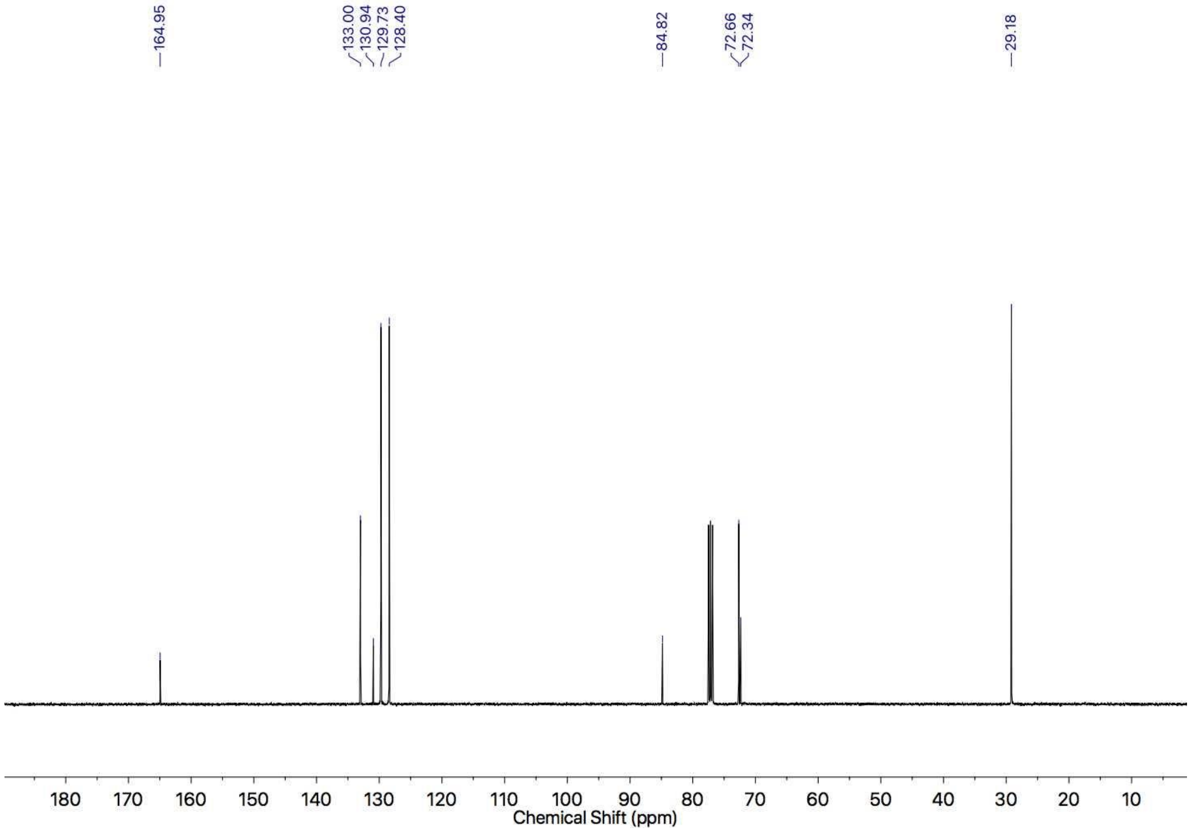

Figure S59: ^{13}C NMR (101 MHz, $CDCl_3$) of **7**.

2-methyl-3-butyn-2-yl pivaloate, **S3**[9,10]

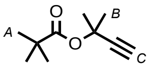

A solution of trimethylacetyl chloride (1.23 g, 10.2 mmol, 1.0 eq.) (in 6.0 mL CH_2Cl_2), was added dropwise to a solution of 2-methyl-3-butyn-2-ol (0.85 g, 10.2 mmol, 1.0 eq.) and 4-(dimethylamino)pyridine (1.87 g, 15.3 mmol, 1.5 eq.) in $CHCl_3$ (6.0 mL) at 0 ˚C. The resulting solution was stirred at 30 ˚C for 16 h. The reaction mixture was diluted with CH_2Cl_2, washed with $NaHCO_3$, and extracted in CH_2Cl_2 and Et_2O. The combined organic phases were dried ($MgSO_4$) and the solvent was removed *in vacuo*. The residue was purified by column chromatography (SiO_2, petrol-Et_2O 0→50%), yielding a pale colourless oil (0.996 g, 6.65 mmol, 58%).

δ_H ($CDCl_3$, 400 MHz) 2.48 (1H, s, $\boldsymbol{H}_C$), 1.64 (6H, s, $\boldsymbol{H}_B$), 1.16 (9H, s, $\boldsymbol{H}_A$).

δ_C ($CDCl_3$, 101 MHz) 176.8, 85.0, 72.0, 71.2, 39.1, 28.9, 27.1.

GC-EI-MS m/z 168.1 [$M^{+\cdot}$].

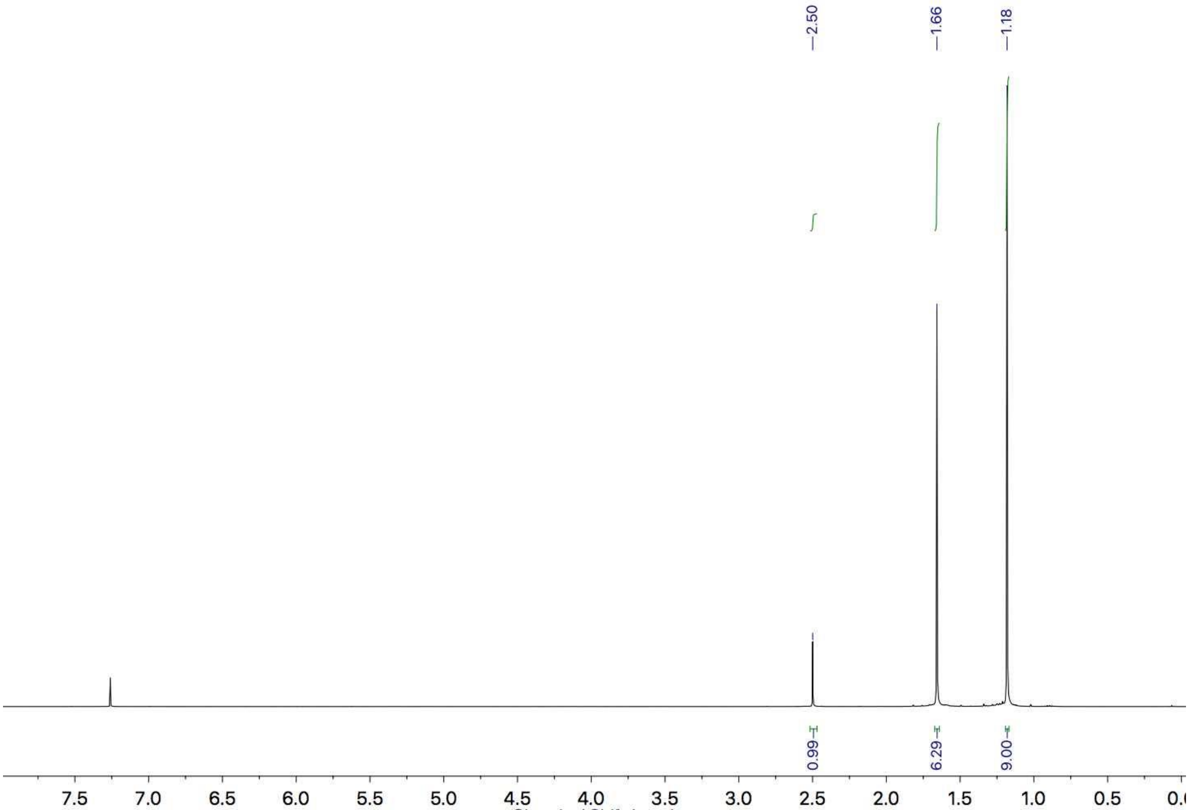

Figure S60: ^{1}H NMR (400 MHz, $CDCl_3$) of **S3**.

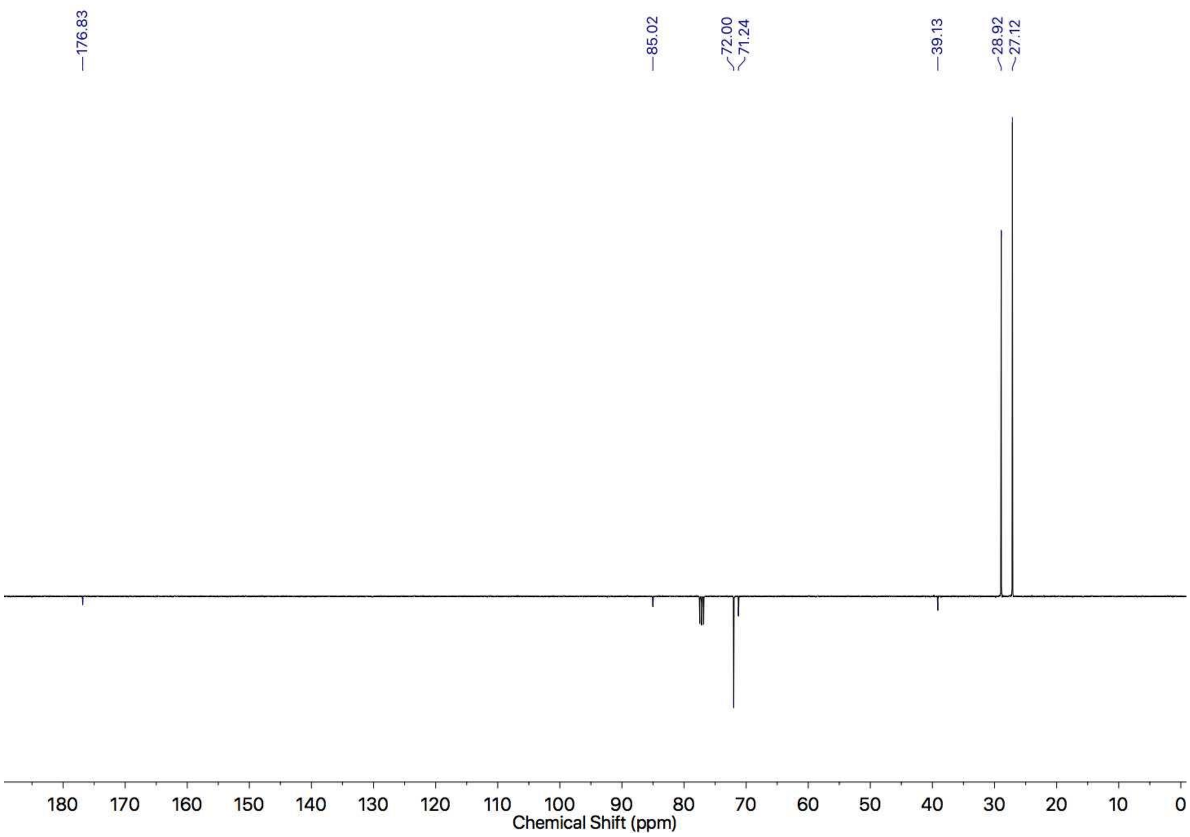

Figure S61: JMOD NMR (101 MHz, $CDCl_3$) of **S3**.

2-methyl-3-butyn-2-yl phenylacetate, **S4**[11]

Phenylacetic acid (0.554 g, 4.0 mmol, 1.0 eq.) was heated under reflux at 80 °C, under inert atmosphere, with $SOCl_2$ (2.32 mL, 32.0 mmol, 8.0 eq.) for 3 h. The reaction was cooled and excess $SOCl_2$ was removed *in vacuo*. The residue was cooled to 0 ˚C, and a solution of 2-methyl-3-butyn-2-ol (0.44 mL, 4.4 mmol, 1.1 eq.) and 4-(dimethylamino)pyridine (0.244 g, 2.0 mmol, 0.5 eq) in $CDCl_3$ (4.0 mL) was added dropwise. The resulting solution was stirred for 16 h at rt. The solvent was removed *in vacuo*, and the residue was purified by column chromatography (SiO_2, petrol-Et_2O 10→30%) yielding a colourless oil (200 mg, 0.989 mmol, 25%).

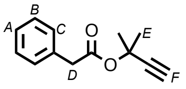

δ_H ($CDCl_3$, 400 MHz) 7.35-7.24 (5H, m, ***H***A, ***H***B, ***H***C), 3.60 (2H, s, ***H***D), 2.52 (1H, s, ***H***F), 1.67 (6H, s, ***H***E).

δ_C ($CDCl_3$, 101 MHz) 169.9, 134.1, 129.4, 128.6, 127.1, 84.7, 72.6, 72.3, 42.1, 29.0.

HR-EI-MS m/z 202.1217 [$M^{+\cdot}$] (calc. m/z for $C_{13}H_{14}O_2$ 202.0994).

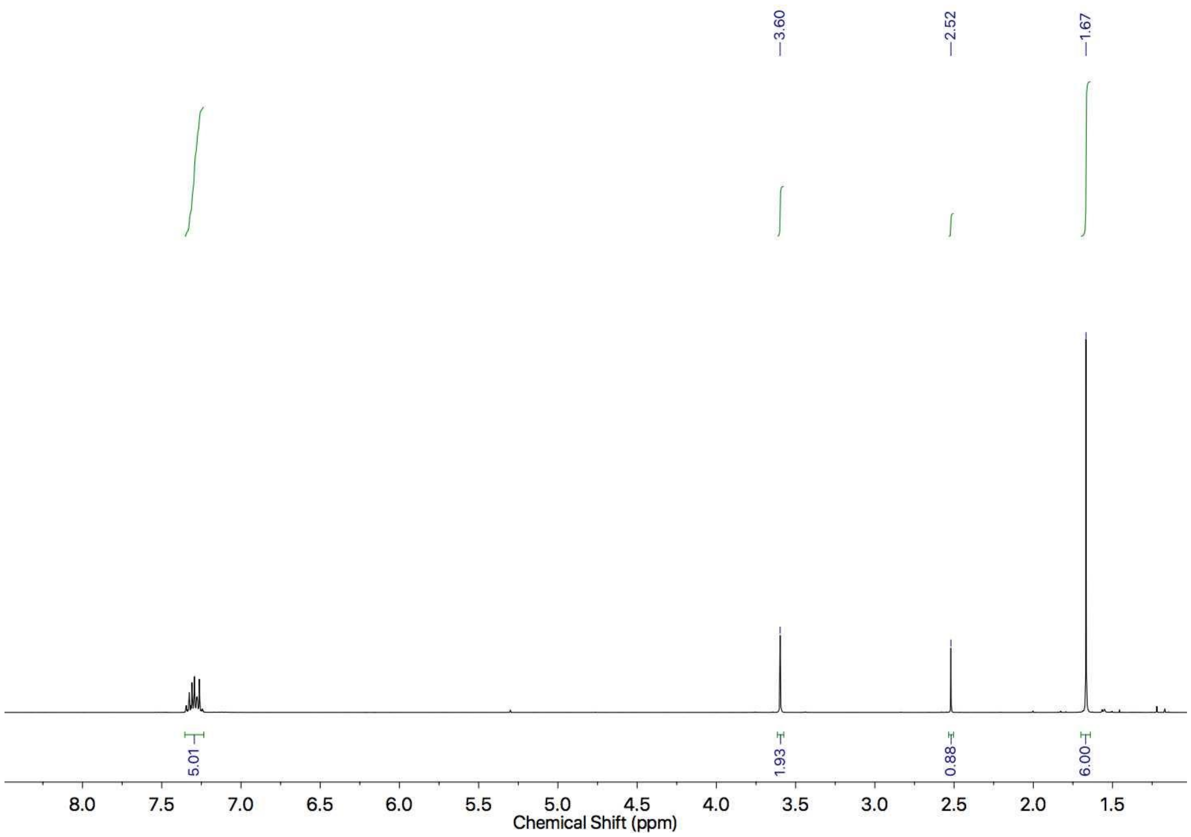

Figure S62: ^{1}H NMR (400 MHz, $CDCl_3$) of **S4**.

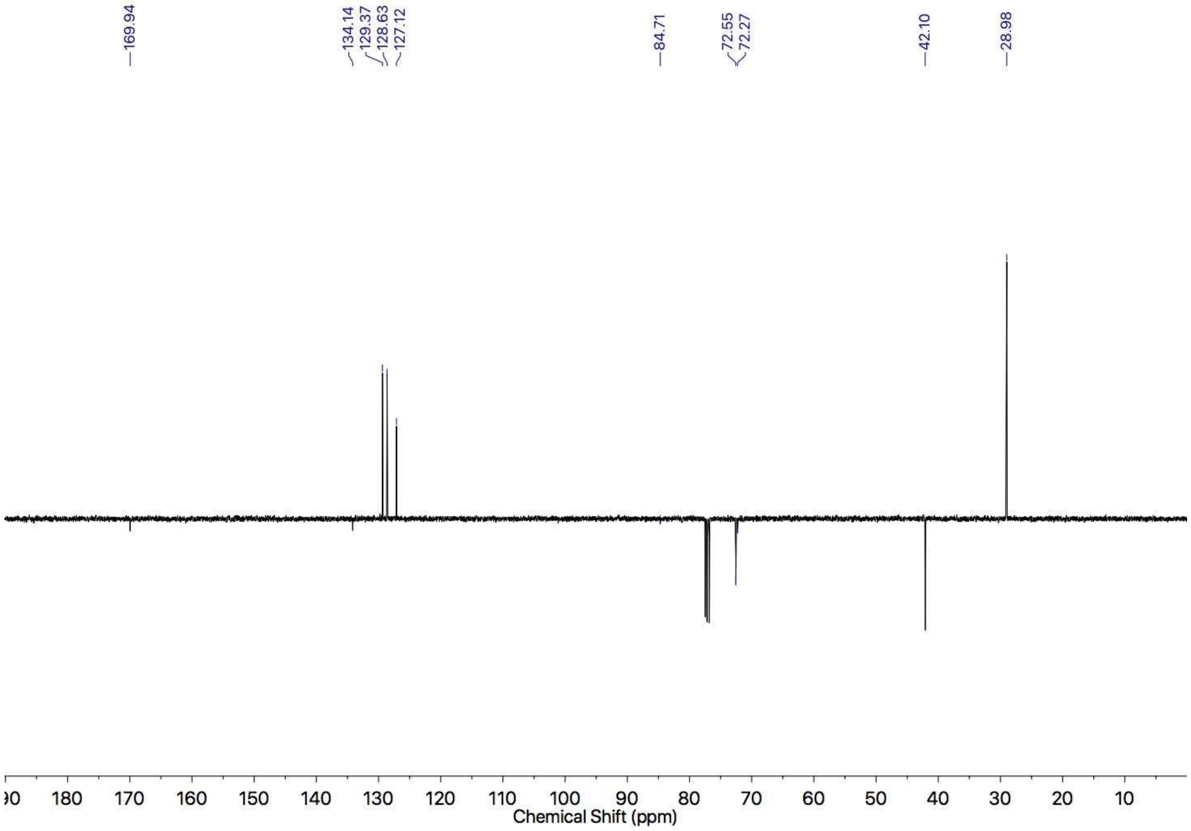

Figure S63: JMOD NMR (101 MHz, $CDCl_3$) of **S4**.

2-methyl-3-butyn-2-yl 4-trifluoromethylbenzoate, **S5**

White solid (128 mg, 0.50 mmol, 50%) m.p. 32-34 °C

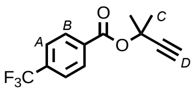

δ_H ($CDCl_3$, 400 MHz) 8.11 (2H, d, *J* = 8.1, ***H***B), 7.70 (2H, d, *J* = 8.2, ***H***A), 2.61 (1H, s, ***H***D), 1.84 (6H, s, ***H***C).

δ_C ($CDCl_3$, 101 MHz) 163.7, 134.5 (q, *J* = 32.7), 134.3 (q, *J* = 18.2), 130.1, 125.4 (q, *J* = 3.7), 122.4, 84.4, 73.1, 73.2, 29.1.

δ_F ($CDCl_3$, 376 MHz) 63.3 (s).

$\delta_{F\{H\}}$ ($CDCl_3$, 376 MHz) 63.3.

HR-EI-MS m/z 256.0701 [$M^{+\cdot}$] (calc. m/z for $C_{13}H_{11}O_2F_3$ 256.0706).

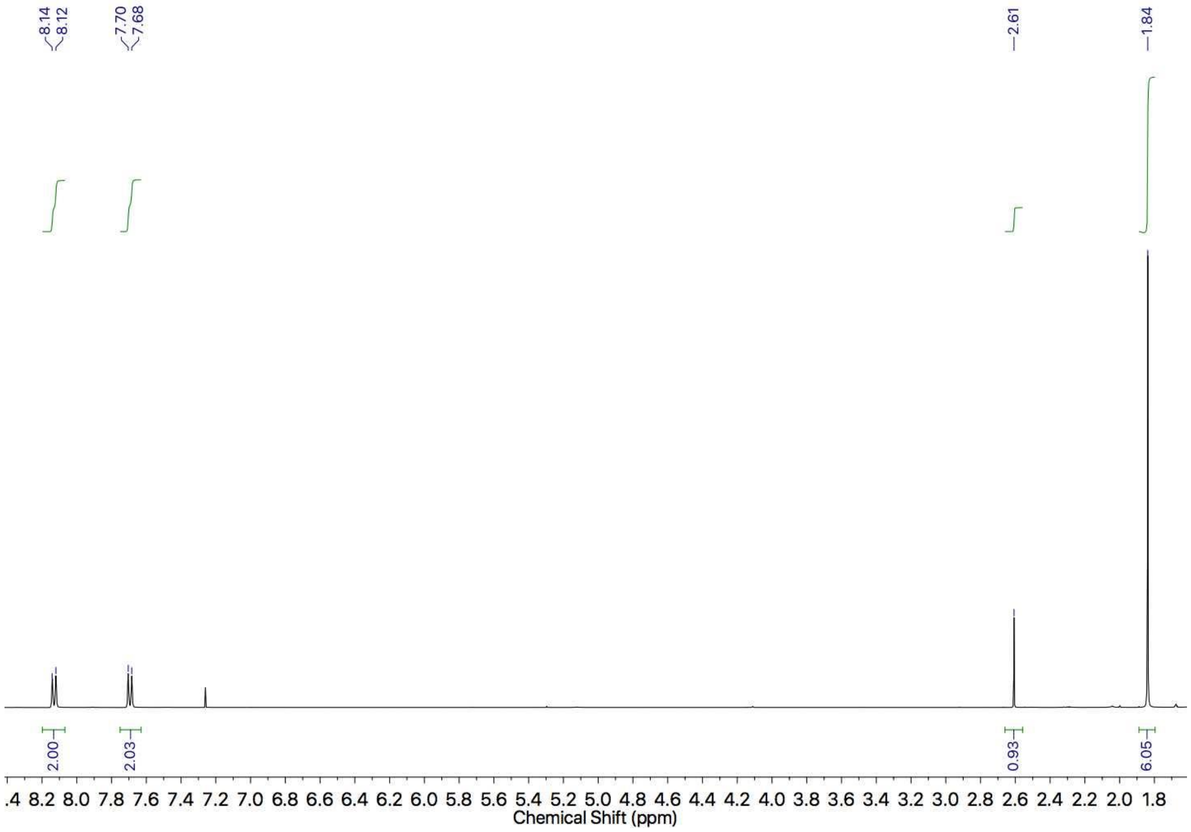

Figure S64: ^{1}H NMR (400 MHz, $CDCl_3$) of **S5**.

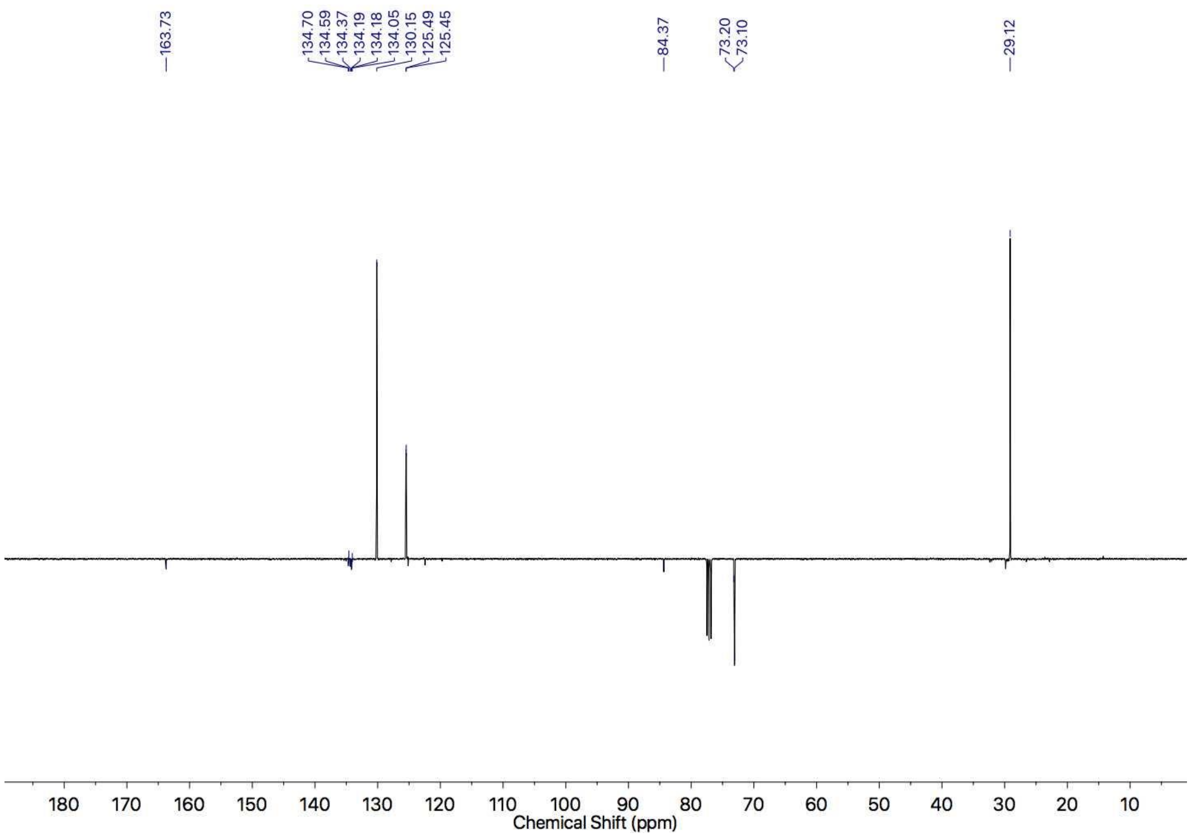

Figure S65: JMOD NMR (101 MHz, $CDCl_3$) of **S5**.

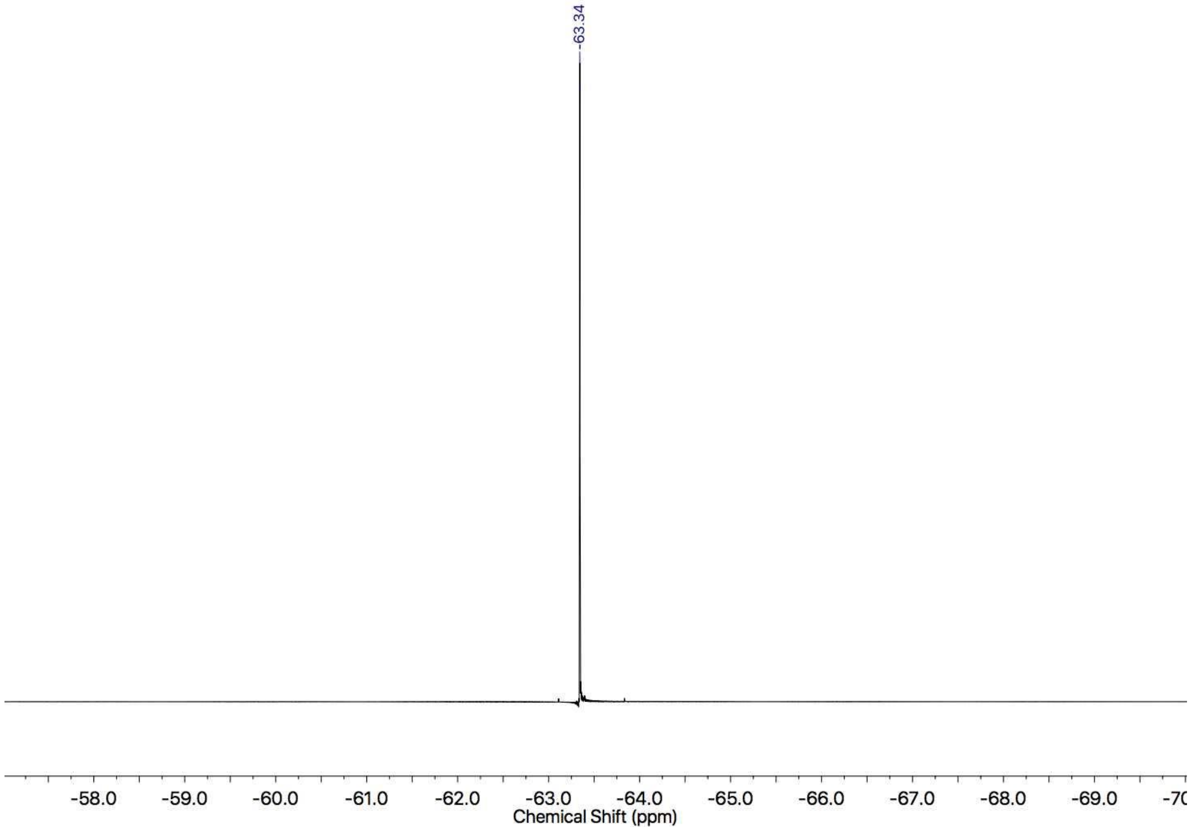

Figure S66: $^{19}F\{^1H\}$ NMR (376 MHz, $CDCl_3$) of **S5**.

2-methyl-3-butyn-2-yl 4-methoxybenzoate, **S6**[12]

Colourless oil (209 mg, 0.96 mmol, 96%)

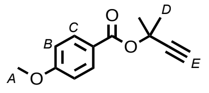

δ_H ($CDCl_3$, 400 MHz) 7.98 (2H, dt, *J* = 9.1, 2.4, $\boldsymbol{H}_C$), 6.91 (2H, dt, *J* = 9.1, 2.4, $\boldsymbol{H}_B$), 3.86 (3H, s, $\boldsymbol{H}_A$), 2.57 (1H, s, $\boldsymbol{H}_E$), 1.81 (6H, s, $\boldsymbol{H}_D$).

δ_C ($CDCl_3$, 101 MHz) 164.8, 163.5, 131.8, 123.4, 113.7, 85.1, 72.5, 72.0, 55.6, 29.2.

HR-EI-MS m/z 318.0933 $[M]^{+\cdot}$ (calc. m/z for $C_{13}H_{14}O_3$ 218.0937).

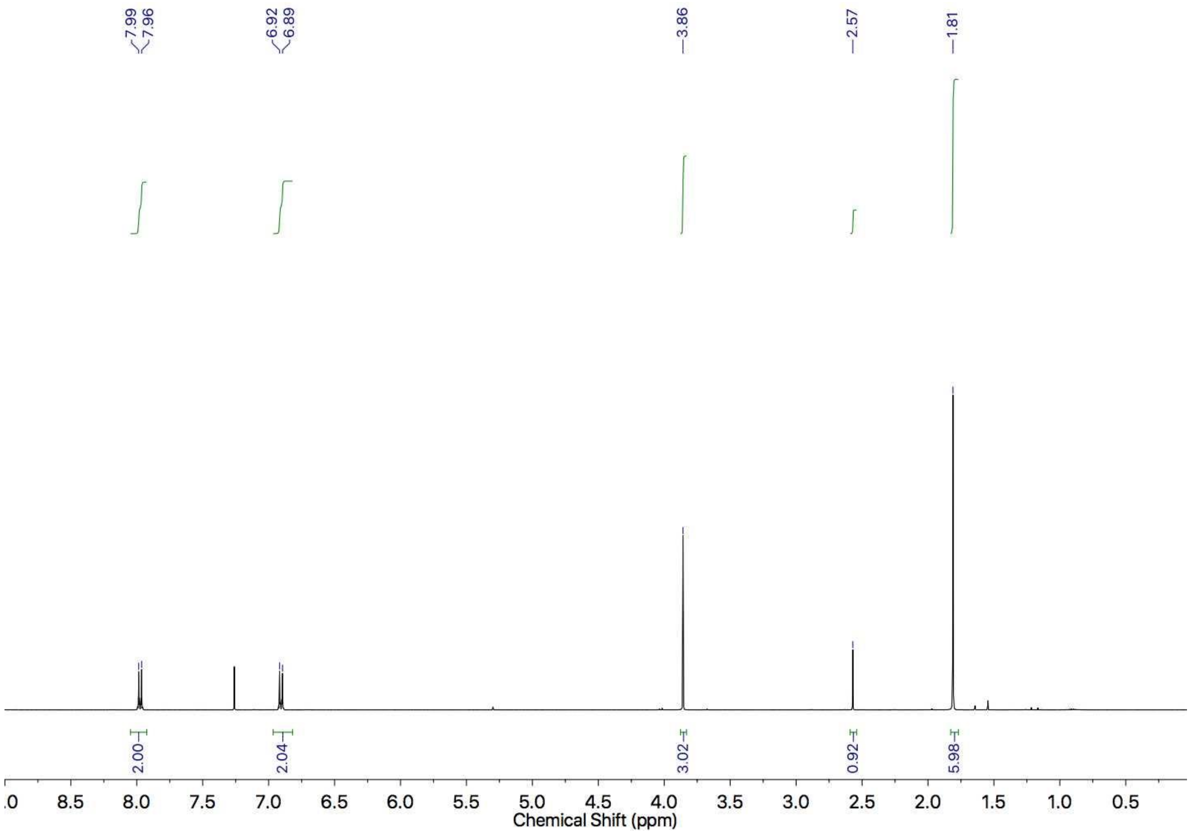

Figure S67: ^{1}H NMR (400 MHz, $CDCl_3$) of **S6**.

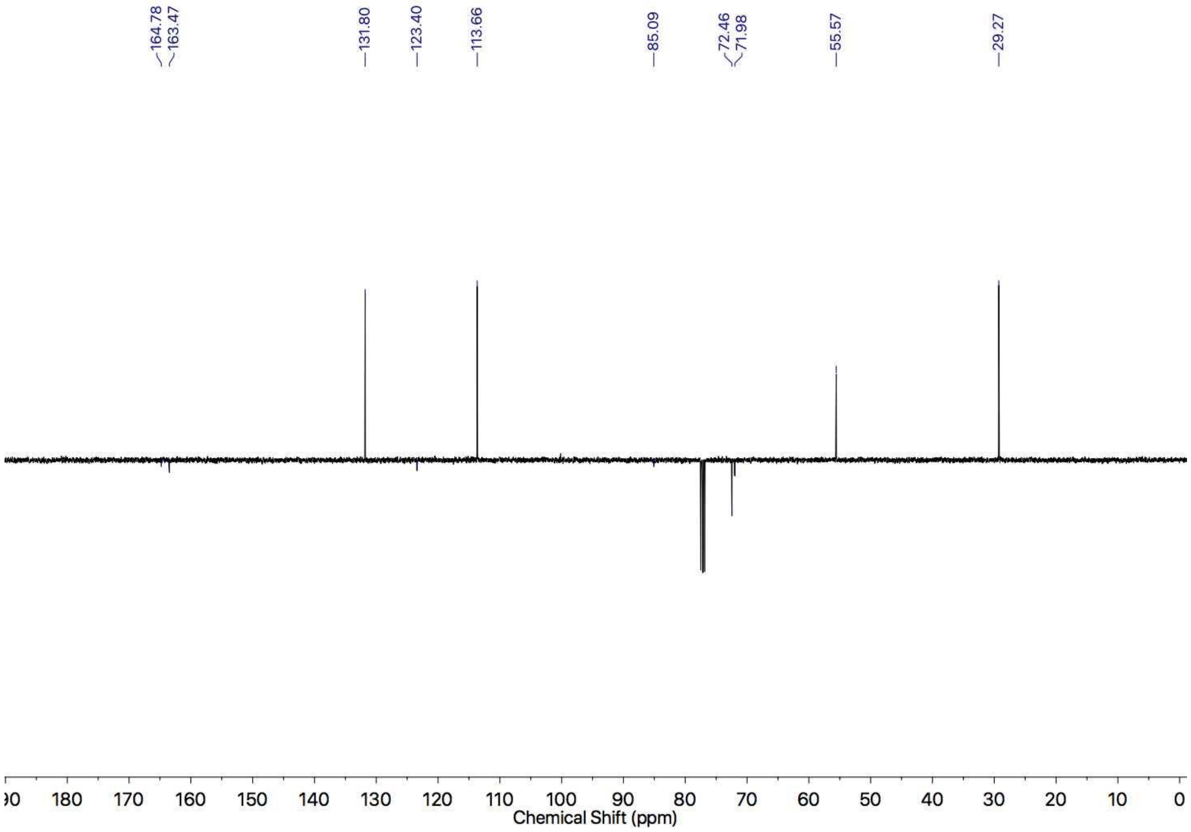

Figure S68: JMOD NMR (101 MHz, $CDCl_3$) of **S6**.

2-methyl-3-butyn-2-yl 4-tertbutylbenzoate, **S7**

Colourless oil (240 mg, 0.982 mmol, 98%)

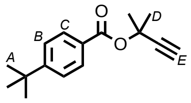

δ_H ($CDCl_3$, 400 MHz) 7.95 (2H, dt, *J* = 8.8, 2.1, ***H***$_C$), 7.45 (2H, dt, *J* = 8.8, 2.1, ***H***$_B$), 2.57 (1H, s, ***H***$_E$), 1.81 (6H, s, ***H***$_D$), 1.33 (18H, s, ***H***$_A$).

δ_C ($CDCl_3$, 101 MHz) 165.0, 156.7, 129.6, 128.2, 125.4, 85.0, 72.5, 72.1, 35.2, 31.3, 29.2.

HR-EI-MS m/z 244.1457 $[M]^{+\cdot}$ (calc. m/z for $C_{16}H_{20}O_2$ 244.1458).

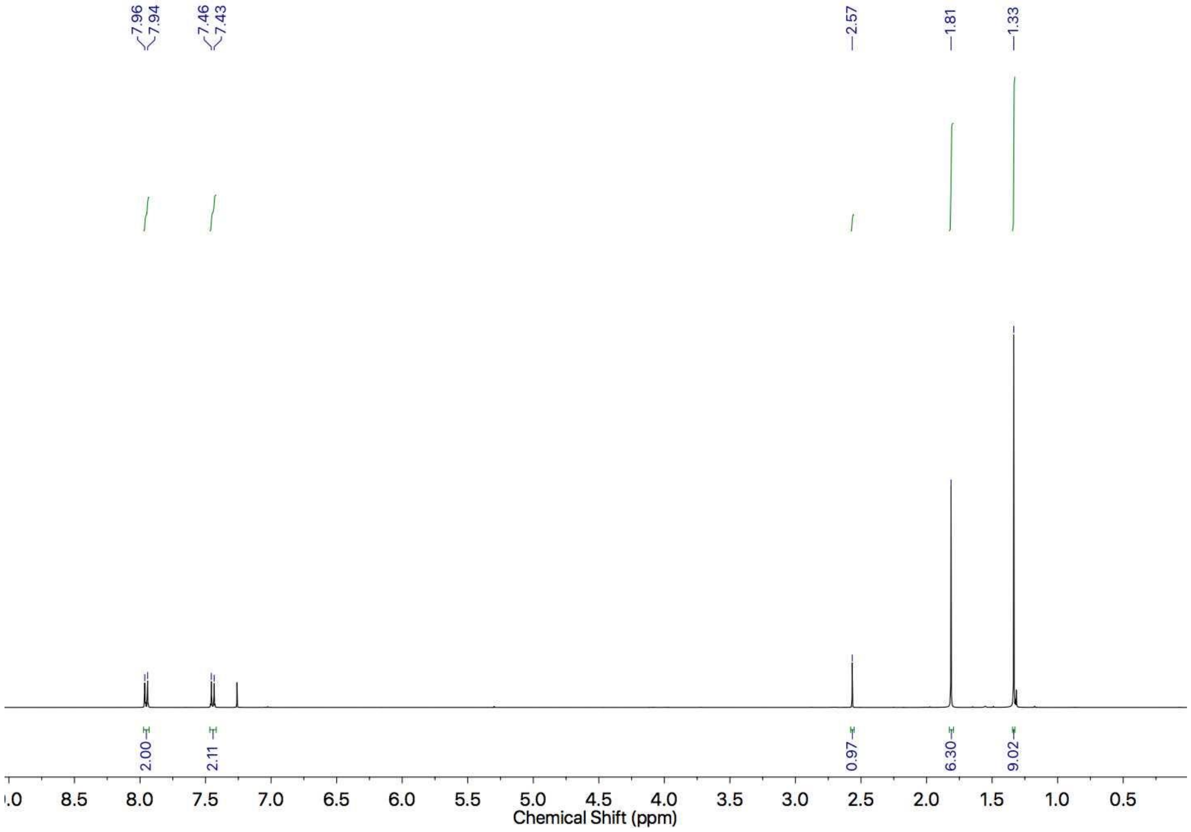

Figure S69: ^{1}H NMR (400 MHz, $CDCl_3$) of **S7**.

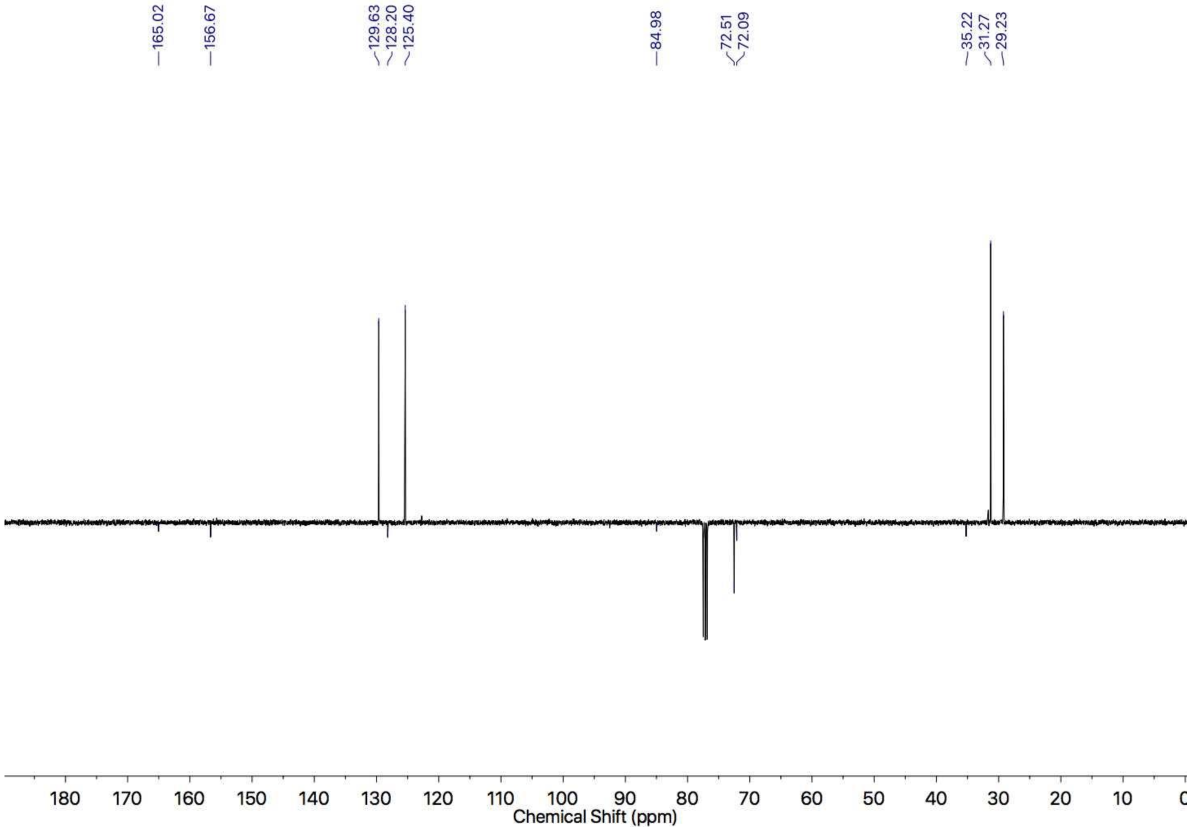

Figure S70: JMOD NMR (101 MHz, $CDCl_3$) of **S7**.

2-methyl-3-butyn-2-yl 3,5-ditertbutylbenzoate, **S8**

Colourless oil (188 mg, 0.626 mmol, 63%)

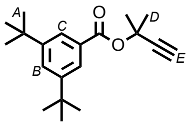

δ_H ($CDCl_3$, 400 MHz) 7.88 (2H, d, *J* = 1.8, $\boldsymbol{H}_C$), 7.62 (1H, t, *J* = 1.8, $\boldsymbol{H}_B$), 2.58 (1H, s, $\boldsymbol{H}_E$), 1.83 (6H, s, $\boldsymbol{H}_D$), 1.34 (18H, s, $\boldsymbol{H}_A$).

δ_C ($CDCl_3$, 101 MHz) 165.8, 151.1, 130.2, 127.3, 123.9, 85.1, 72.5, 72.1, 35.1, 31.5, 29.2.

HR-EI-MS m/z 300.2080 $[M]^{+\cdot}$ (calc. m/z for $C_{20}H_{28}O_2$ 300.2084).

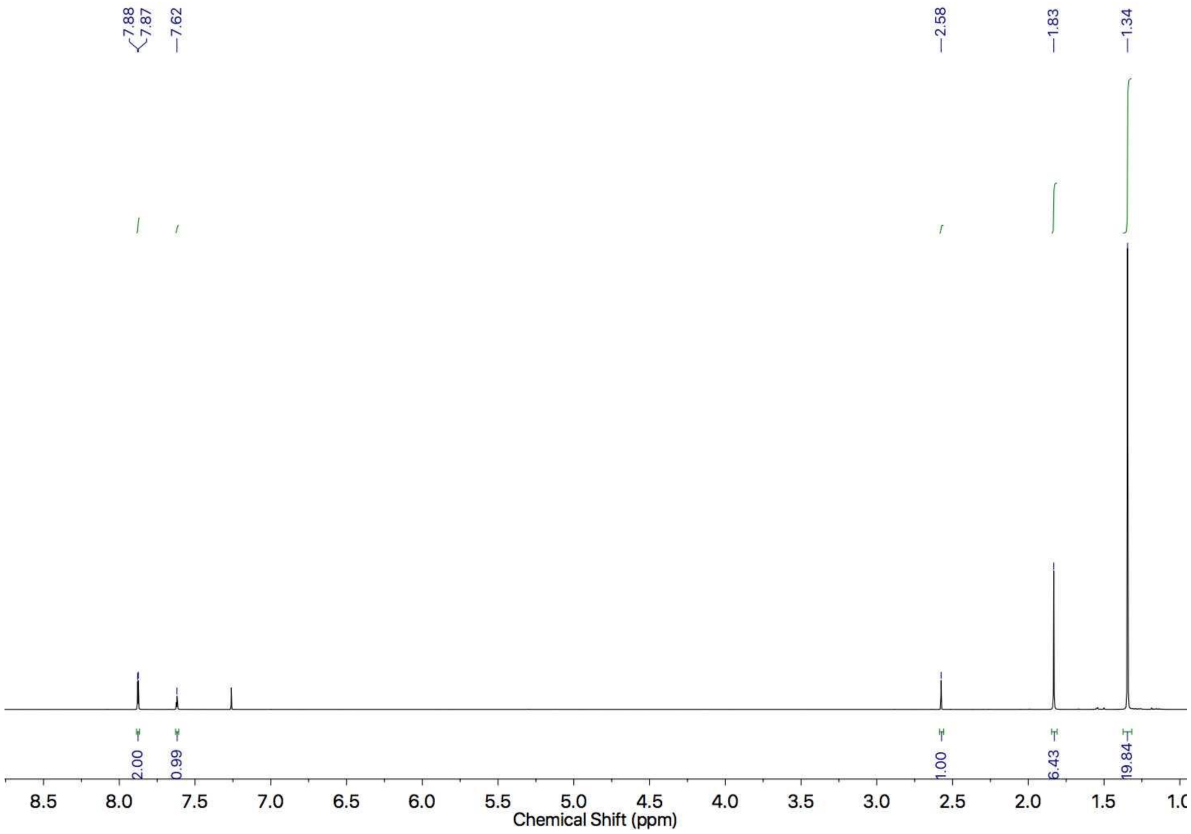

Figure S71: ^{1}H NMR (400 MHz, $CDCl_3$) of **S8**.

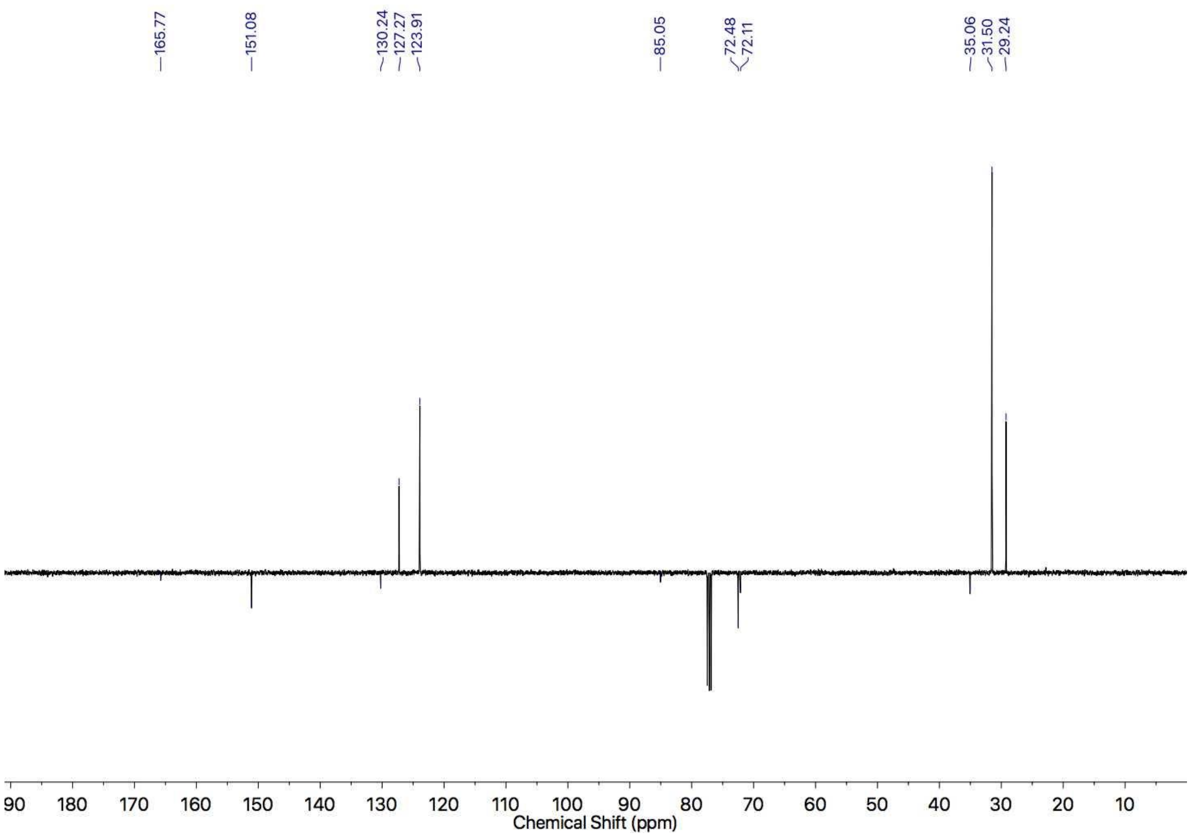

Figure S72: JMOD NMR (101 MHz, $CDCl_3$) of **S8**.

5. Cyclopropanation Reactions

Screening of reaction conditions

Optimum catalytic conditions were determined by screening the reaction of **7** with **8** mediated by [Au(**6**)(Cl)] for a range of solvents, additives and temperatures (**Table S3**). Unless otherwise stated, the stereopurity of the rotaxane catalyst used was 97 : 3 *er*. The best conditions (entry 9) obtained are given in the general procedure (*vide infra*) and were used throughout the experiments that follow.

Table S3. Screening of conditions for the reaction of 7 and 8 mediated by [Au(6)(Cl)]

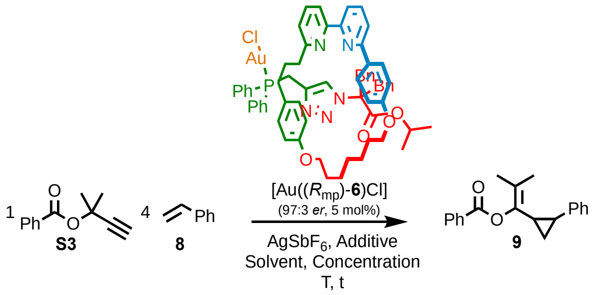

Entry[a]	Solvent	T (°C)	Additive	t (h)	Yield 9 (%) (*cis* : *trans*[b])	*e.r.*$_{cis}$[c]	*e.r.*$_{trans}$[c]
1	$CDCl_3$	25	-	1	-	-	-
2	$CDCl_3$	25	Cu^I	1	42% (95 : 5)	72 : 28	58 : 42
3[b]	$CDCl_3$	25	Cu^I	1	35% (95 : 5)	29 : 71	42 : 58
4	$MeNO_2$	25	Cu^I	1	69% (87 : 13)	53 : 47	65 : 35
5	CD_2Cl_2	25	Cu^I	1	13% (83 : 17)	64 : 36	66 : 34
6	CCl_4	25	Cu^I	1	46% (86 : 14)	71 : 29	58 : 42
7	PhMe	25	Cu^I	1	17% (85 : 15)	69 : 31	56 : 44
8[e]	$CDCl_3$	0	-	6	-	-	-
9[e]	$CDCl_3$	0	Cu^I	6	86% (94 : 6)	79 : 21	62 : 38
10[e]	$CDCl_3$	0	Zn^{II}	6	0% (-)	-	-
11[e]	$CDCl_3$	0	H^+	6	1% (-)	-	-
12[e]	$CDCl_3$	0	-	6	0% (-)	-	-
13	$CDCl_3$	-35	Cu^I	24	25% (96 : 4)	79 : 21	61 : 39
14[f]	$MeNO_2$	25	-	0.5	73% (>20 : 1)	16 : 84	-

[a][Au((***R***$_{mp}$)-**6**)(Cl)] with e.r. = 97 : 3 stereopurity was used for screening experiments unless otherwise stated. [b]Determined by ^1H NMR analysis of the crude reaction product using $C_2H_2Cl_4$ as an internal standard for yield determination. [c]Determined by HPLC. [d]Reaction conducted with [Au((*S*$_{mp}$)-**6**)(Cl)] of 3 : 97 ***er***. [e]Reaction conducted with [Au(**6**)(Cl)] with e.r. = 99 : 1 stereopurity. [a] Cu^I refers to $[Cu(MeCN)_4]PF_6$, Zn^{II} refers to $Zn(OTf)_2$, H^+ refers to HOTs.H_2O, Ag^I refers to $AgSbF_6$. [f]Reaction outcome reported by Toste and co-workers for (*R*)-DTBM-SEGPHOS®$(AuCl)_2$.[6]

Determination of the absolute stereochemistry of cyclopropanes 9

The absolute stereochemistry of cyclopropanes **9** was determined by comparing the HPLC chromatogram of the major *cis* diastereomer produced by [Au(**6**)(Cl)], with that produced using (*R*)-DTBM-SEGPHOS®$(AuCl)_2$ under conditions reported by Toste and co-workers, the stereochemical outcome of which is known.[6] Using this approach, the reactions mediated by [Au((R_{mp})-**6**)Cl] and [Au((S_{mp})-**6**)Cl] (**Table S3**, entries 2 and 3) were shown to yield (1*S*,2*R*)-**9** and (1*R*,2*S*)-**9** respectively as their major products.

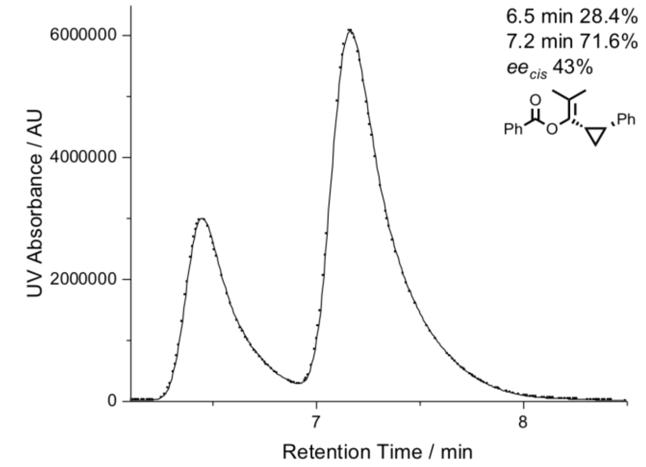

Figure S73: Chiral Stationary Phase HPLC (RegisPack, *n*-hexane-isopropanol 99 : 1, 303 K, load Et_2O, flowrate 0.75 mLmin^{-1}) of 72 : 28 *er cis*-**9** produced using [Au((R_{mp})-**6**)Cl] (97 : 3 *er*). Retention times (min): (1*R*,2*S*)-**9** 6.6, (1*S*,2*R*)-**9** 7.4.

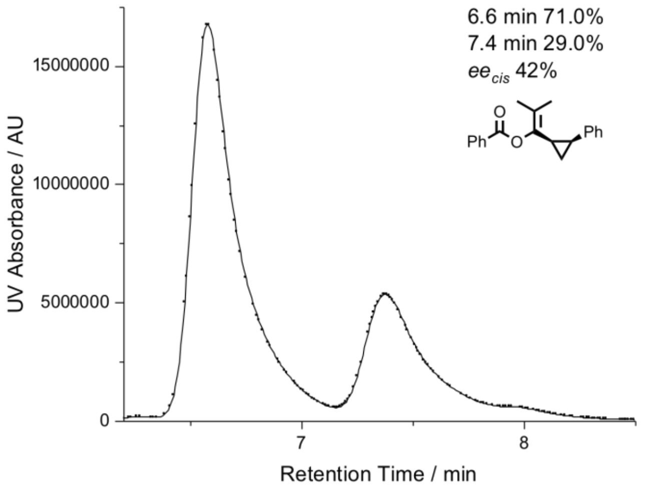

Figure S74: Chiral Stationary Phase HPLC (RegisPack, *n*-hexane-isopropanol 99 : 1, 303 K, load Et_2O, flowrate 0.75 mLmin^{-1}) of 29 : 71 *er cis*-**9** produced using [Au((S_{mp})-**6**)Cl] (3 : 97 *er*). Retention times (min): (1*R*,2*S*)-**9** 6.6, (1*S*,2*R*)-**9** 7.4.

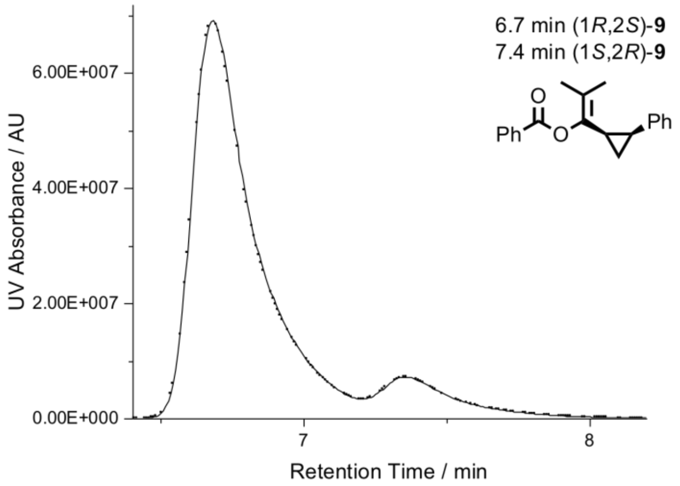

Figure S75: Chiral Stationary Phase HPLC (RegisPack, *n*-hexane-isopropanol 99 : 1, 303 K, load Et_2O, flowrate 0.75 $mLmin^{-1}$) of *cis*-**9** produced using (*R*)-DTBM-SEGPHOS®$(AuCl)_2$ in according to literature conditions to determine absolute stereochemistry.[6] Retention times (min): (1*R*,2*S*)-**9** 6.6, (1*S*,2*R*)-**9** 7.4.

Cyclopropanation General Procedures

Catalyst [Au(6)(Cl)]: A foil-covered, CEM MW vial was charged with $AgSbF_6$ (1.0 mg, 2.97 µmol, 0.05 eq.) and $[Cu(MeCN)_4]PF_6$ (1.1 mg, 3.0 µmol, 0.05 eq.), then purged with N_2. LAuCl was added as solution in $CDCl_3$ (0.059 M, 50 µL 3.0 µmol, 0.05 eq.) and the solution cooled to 0 ˚C. After 5 minutes, 2-methyl-3-butyn-2-yl ester (59.4 µmol, 1.0 eq.) and alkene (238 µmol, 4.0 eq.) were added in $CDCl_3$ (0.54 mL) (0.1 M with respect to the alkyne), and the solution stirred for 6 h at 0 ˚C . After 6 h, $C_2H_2Cl_4$ (20.0 µL, 0.189 mmol) was added, the solution was filtered through Celite®, and the yield and *dr* was determined by ^1H NMR. The reaction mixture was purified by column chromatography (SiO_2, petrol-Et_2O 0→10%) yielding isolated *cis* and *trans* cyclopropanes. Where possible, the enantiopurity of both diastereoisomers was determined by chiral stationary phase HPLC.

$[Au(PPh_3)(Cl)]$: Reactions with $[Au(PPh_3)(Cl)]$ were performed as per the general procedure at rt for 2 h in the absence of $[Cu(MeCN)_4]PF_6$ to provide racemic samples for HPLC method development.

(*R*)-DTBM-SEGPHOS®$(AuCl)_2$: Performed as per literature procedure (rt, $MeNO_2$, 2.5 mol% [Au(L)(Cl) (2.5 mol%), $AgSbF_6$ (5 mol%), benzoyl ester (1 eq., 0.05 M), alkene (4 eq.), $MeNO_2$, rt, 2 h).[6]

Cyclopropanes **9**[6]

**Catalyst**	**Yield / %**	***dr***	***er*cis**	***er*trans**
$(Ph_3P)AuCl$	96	89 : 11	1 : 1	1 : 1
[Au((*R*mp)-**6**)(Cl)]	86	94 : 6	78.5 : 21.5	62 : 38
(*R*)-DTBM-SEGPHOS®$(AuCl)_2$	73	>20 : 1	16 : 84	-

Table S4. Summary of reactions leading to cyclopropanes **9**.

cis-**9**

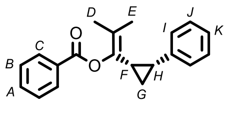

δ_H ($CDCl_3$, 400 MHz) 7.88 (2H, d, *J* = 7.5, H_C), 7.58 (1H, tt, *J* = 7.5, 1.5, H_A), 7.43 (2H, tt, *J* = 7.5, 1.0, H_B), 7.26 (2H, dd, *J* = 7.0, 6.5, H_J), 7.20 (1H, tt, *J* = 7.0, 1.5, H_K), 7.12 (2H, dd, *J* = 7.5, 1.5, H_I), 2.43-2.28 (2H, m, H_H, H_F), 1.65 (3H, s, H_F), 1.49 (3H, s, H_D), 1.29 (1H, ddd (td), *J* = 9.0, 5.5, H_G), 1.11 (1H, dt (ddd), *J* = 5.5, 5.5, $H_{G'}$).

δ_C ($CDCl_3$, 101 MHz) 164.8, 139.5, 138.7, 133.2, 130.0, 129.9, 128.5, 127.8, 127.8, 125.7, 123.6, 23.9, 21.6, 18.8, 17.8, 11.9.

HR-EI-MS m/z 292.1455 [$M^{+\cdot}$] (calc. m/z for $C_{20}H_{20}O_2$ 292.1458).

trans-**9**

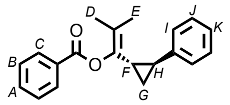

δ_H ($CDCl_3$, 400 MHz) 8.13 (2H, dd, *J* = 8.1, 1.4, H_C), 7.61 (1H, tt, *J* = 7.4, 2.7, H_A), 7.49 (2H, app. t, *J* = 7.6, H_B), 7.26 (2H, br. t, *J* = 7.5, H_J), 7.16 (1H, tt, *J* = 7.4, 1.3, H_K), 7.10 (2H, dd, *J* = 7.7, 1.5, H_I), 7.28-7.18 (2H, m, H_F, H_H), 1.86 (3H, s, H_E), 1.63 (3H, s, H_D), 1.21 (1H, ddd, *J* = 8.7, 6.2, 4.9, H_G), 1.16 (1H, ddd, *J* = 8.9, 6.0, 5.1, $H_{G'}$).

δ_C ($CDCl_3$, 101 MHz) 164.9, 142.2, 141.0, 133.4, 130.1, 129,8, 128.7, 128.5, 126.1, 125.9, 121.1, 23.8, 23.5, 19.0, 18.3, 14.8.

HR-EI-MS m/z 292.1455 [$M^{+\cdot}$] (calc. m/z for $C_{20}H_{20}O_2$ 292.1458).

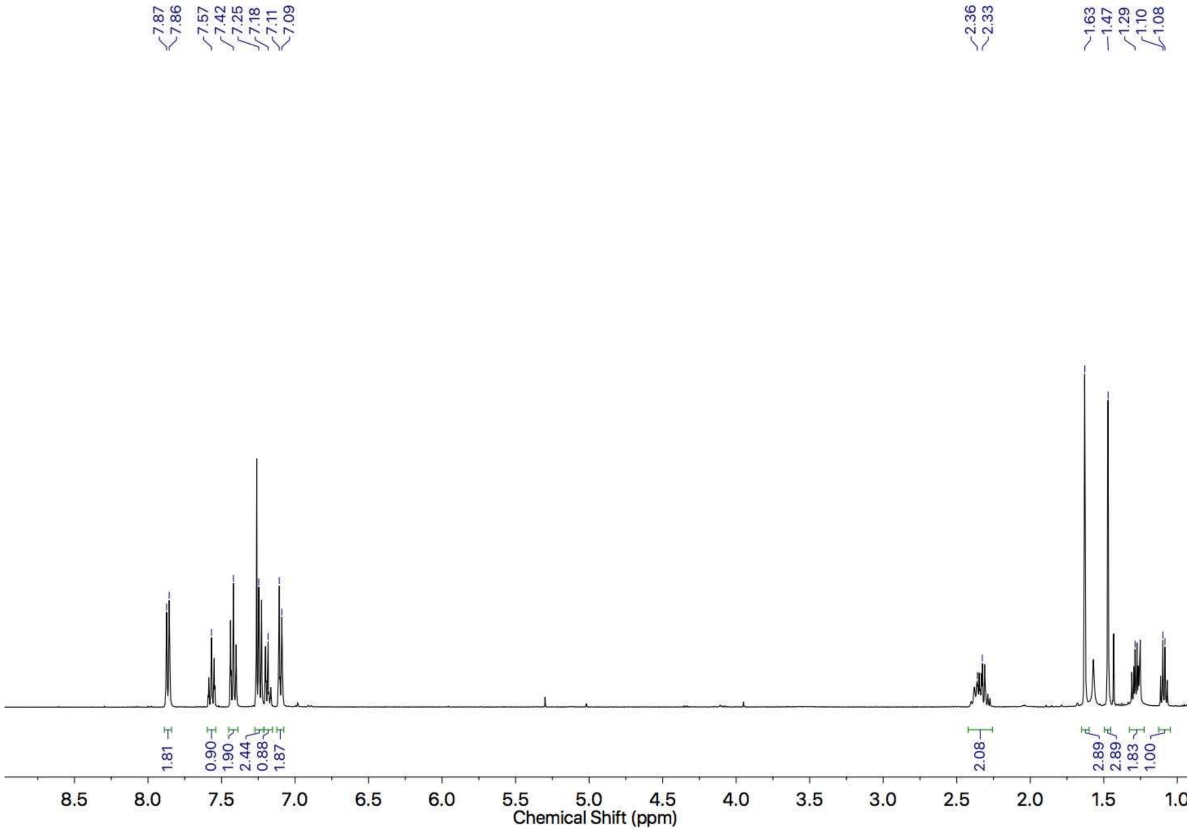

Figure S76: ^{1}H NMR (400 MHz, $CDCl_3$) of *cis*-**9**.

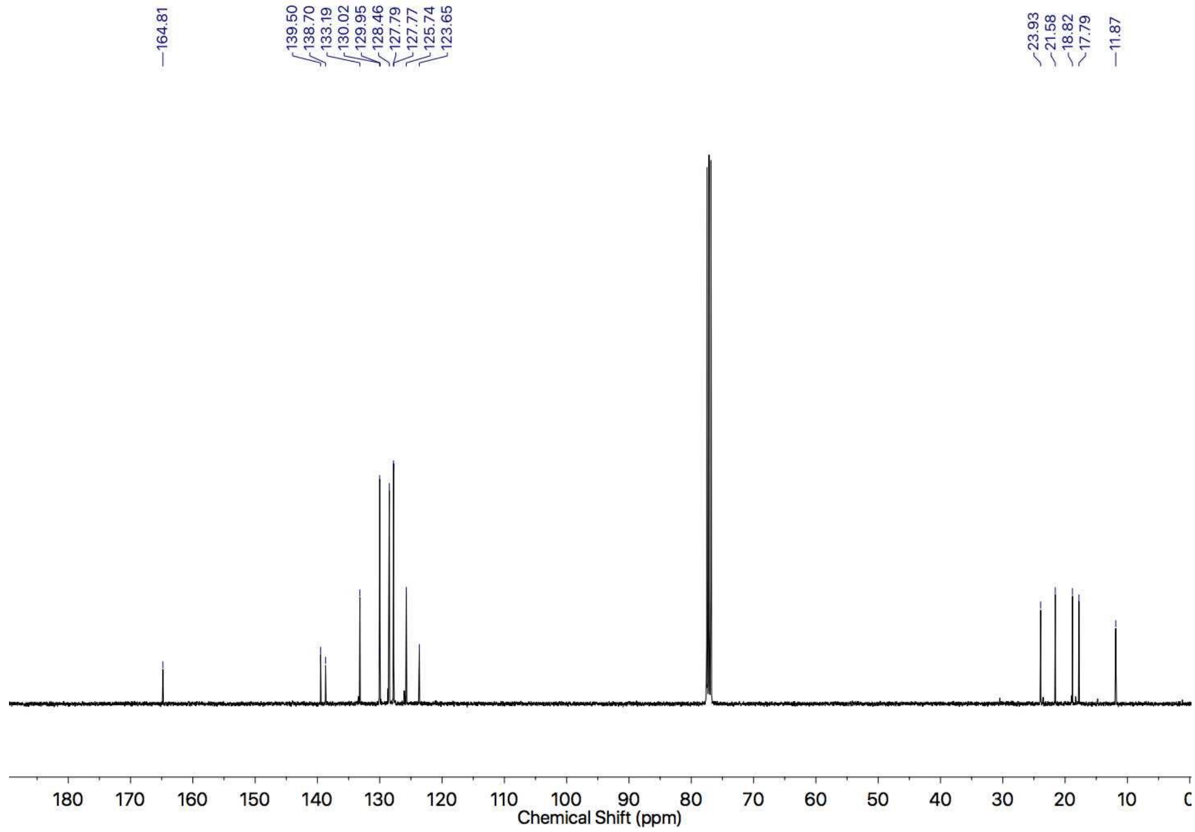

Figure S77: ^{13}C NMR (101 MHz, $CDCl_3$) of *cis*-**9**.

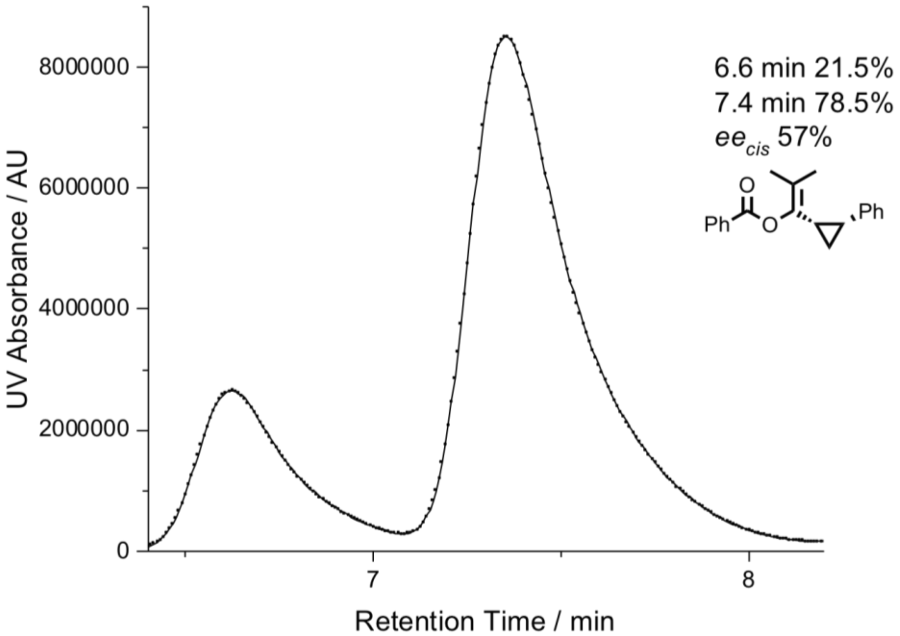

Figure S78: Chiral Stationary Phase HPLC (RegisPack, *n*-hexane-isopropanol 99 : 1, 303 K, load Et_2O, flowrate 0.75 $mLmin^{-1}$) of 78.5 : 21.5 *er cis*-**9**. Retention times (min): (1*R*,2*S*)-**9** 6.6, (1*S*,2*R*)-**9** 7.4.

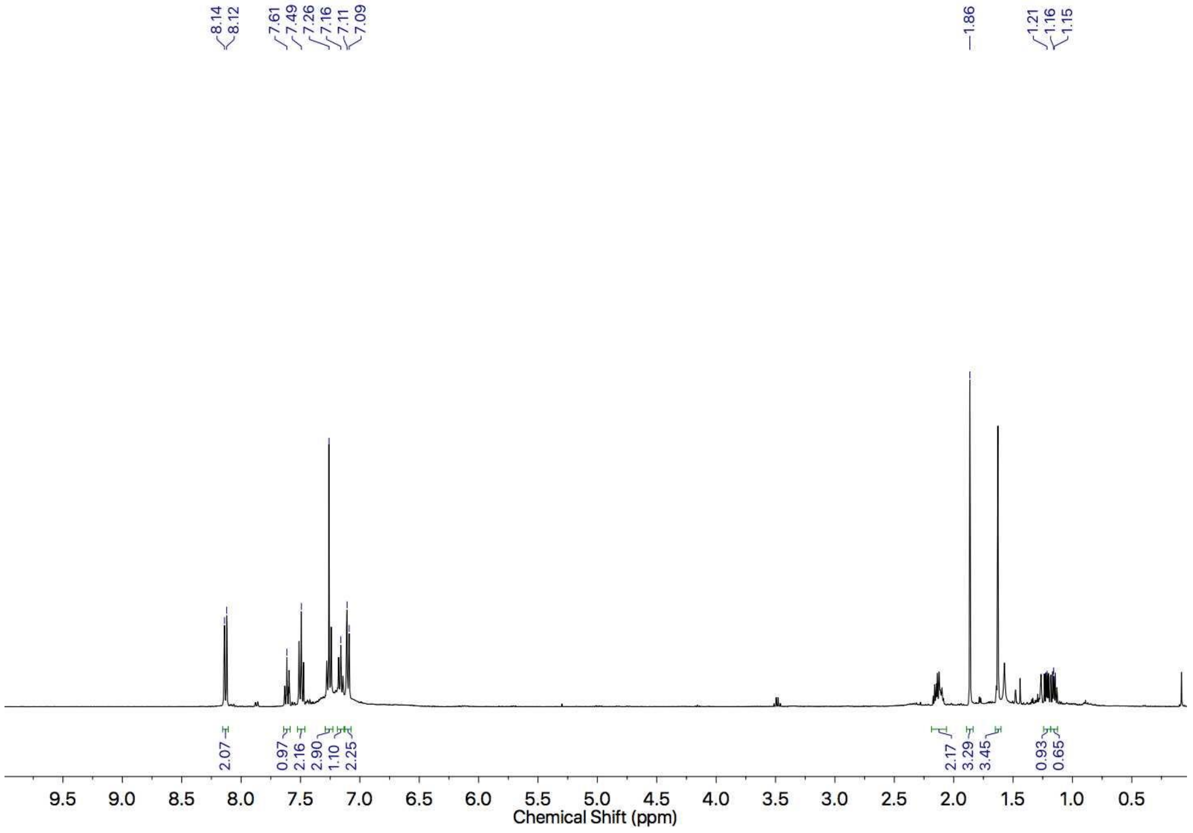

Figure S79: ^1H NMR (400 MHz, $CDCl_3$) of *trans*-**9**.

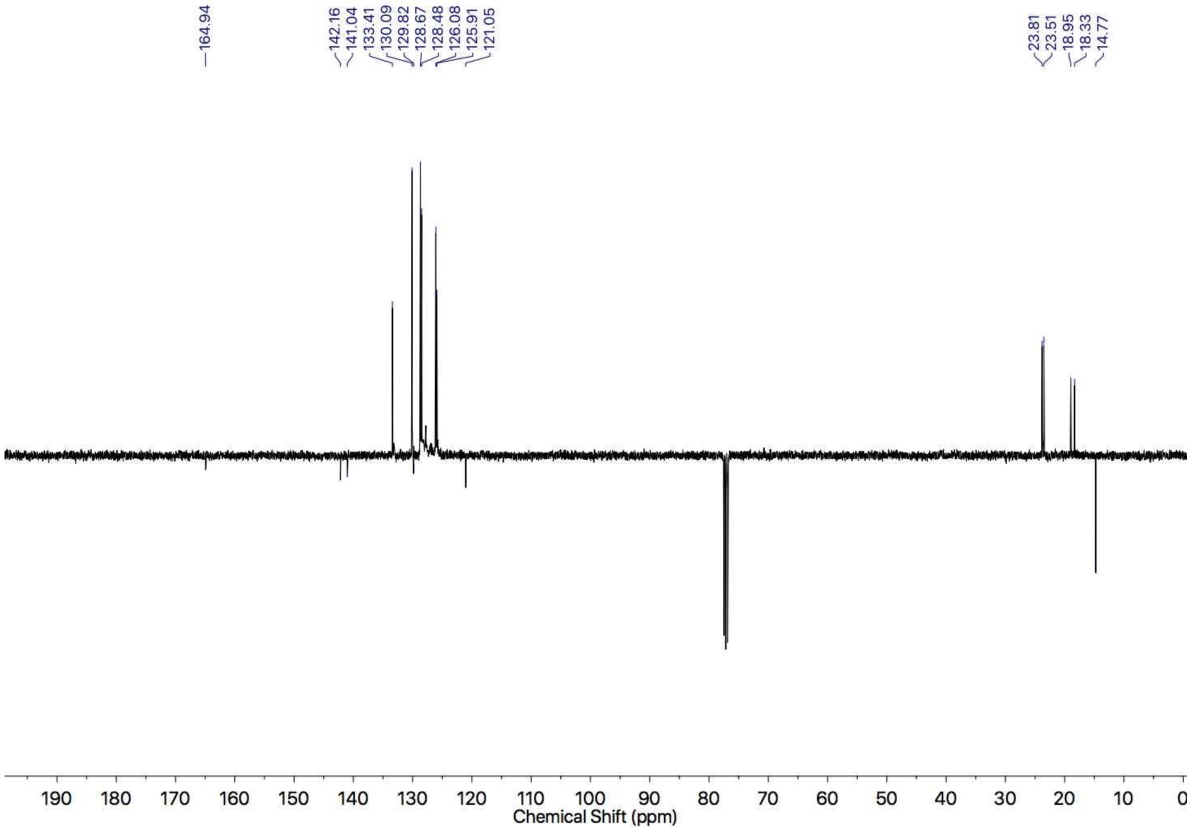

Figure S80: JMOD NMR (101 MHz, $CDCl_3$) of *trans*-**9**.

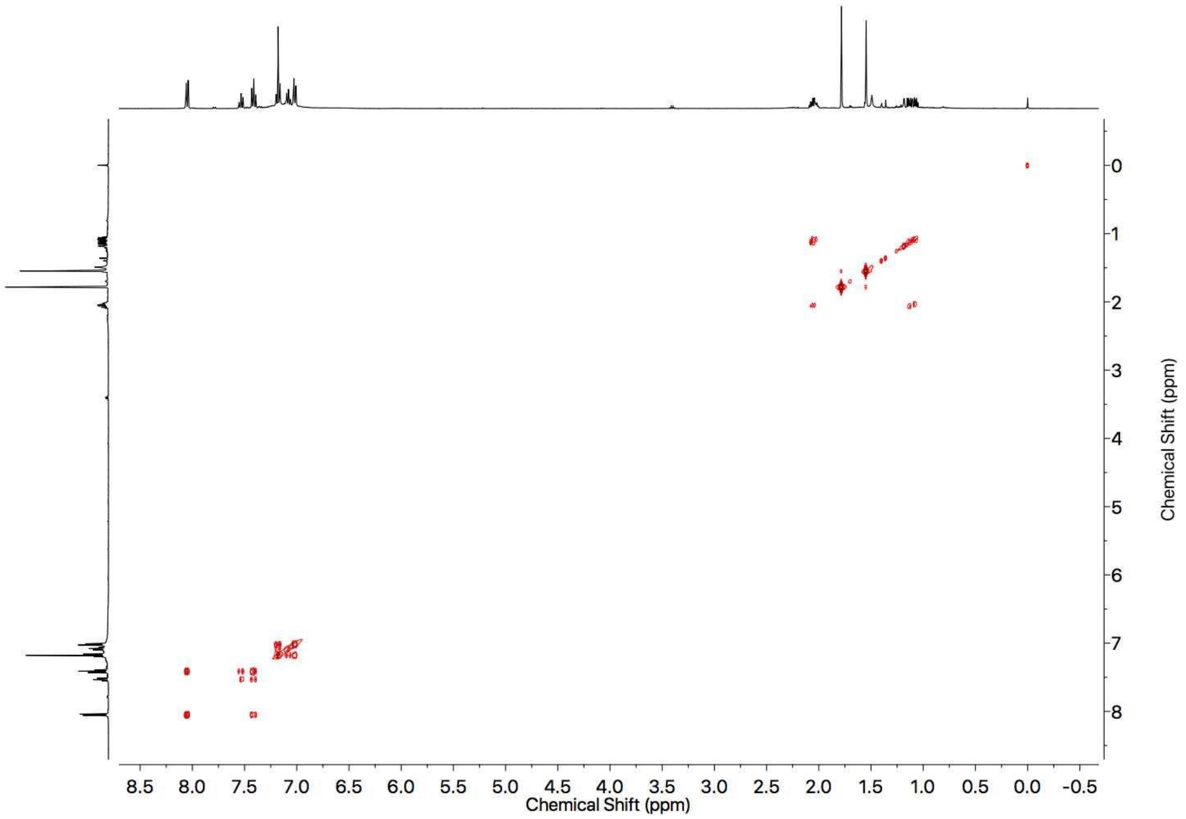

Figure S81: COSY NMR ($CDCl_3$) of *trans*-**9**.

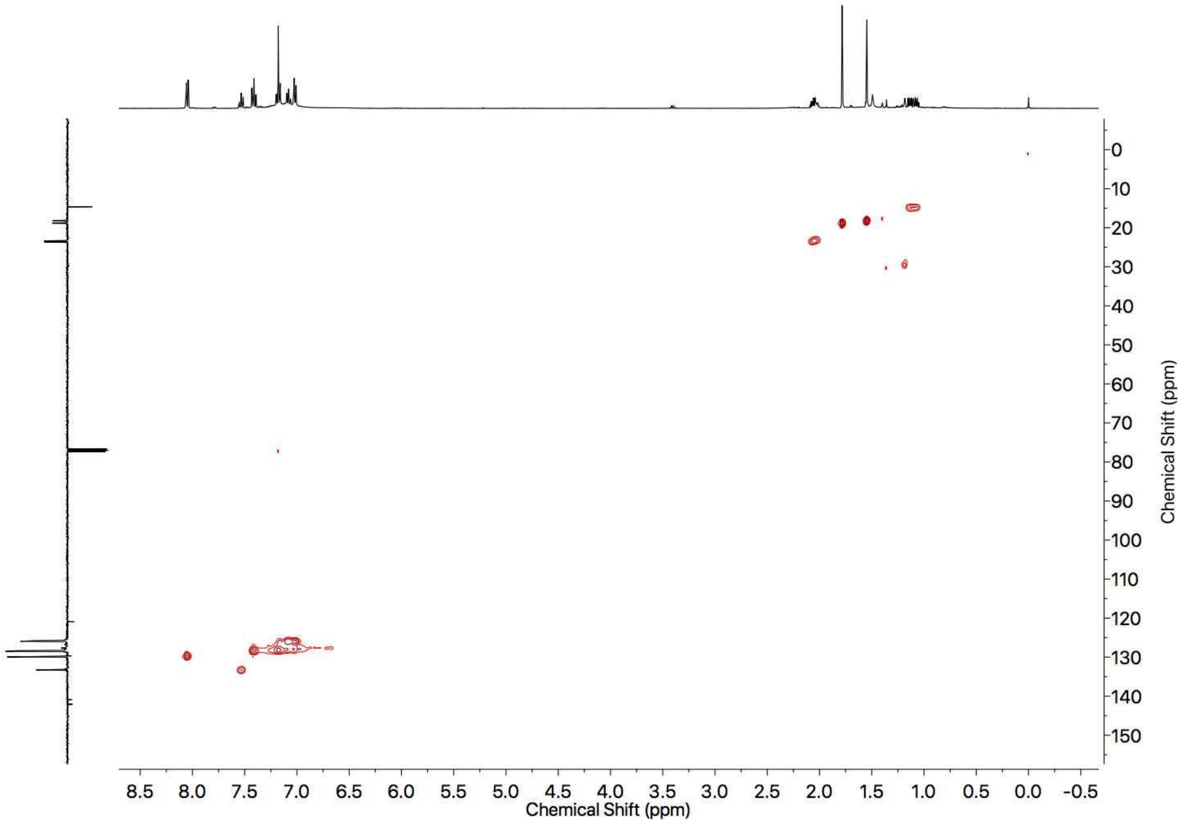

Figure S82: HSQC NMR ($CDCl_3$) of *trans*-**9**.

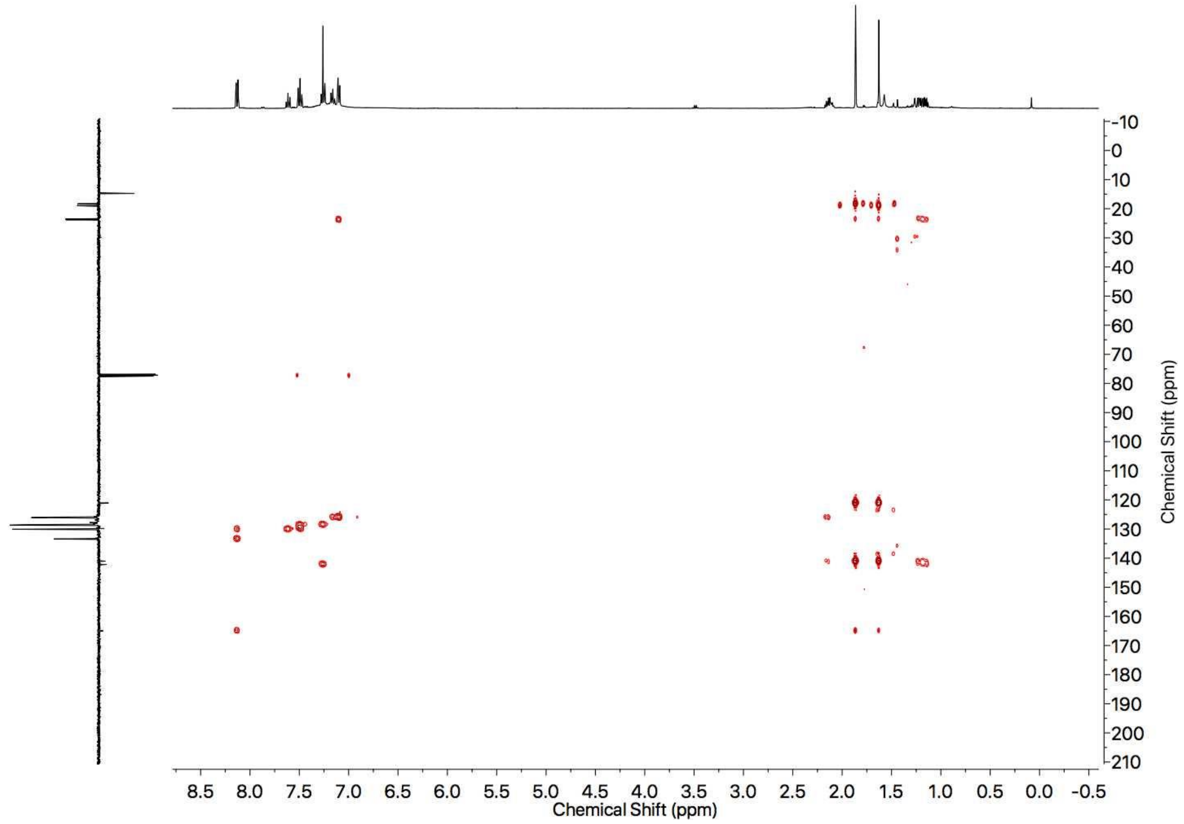

Figure S83: HMBC NMR ($CDCl_3$) of *trans*-**9**.

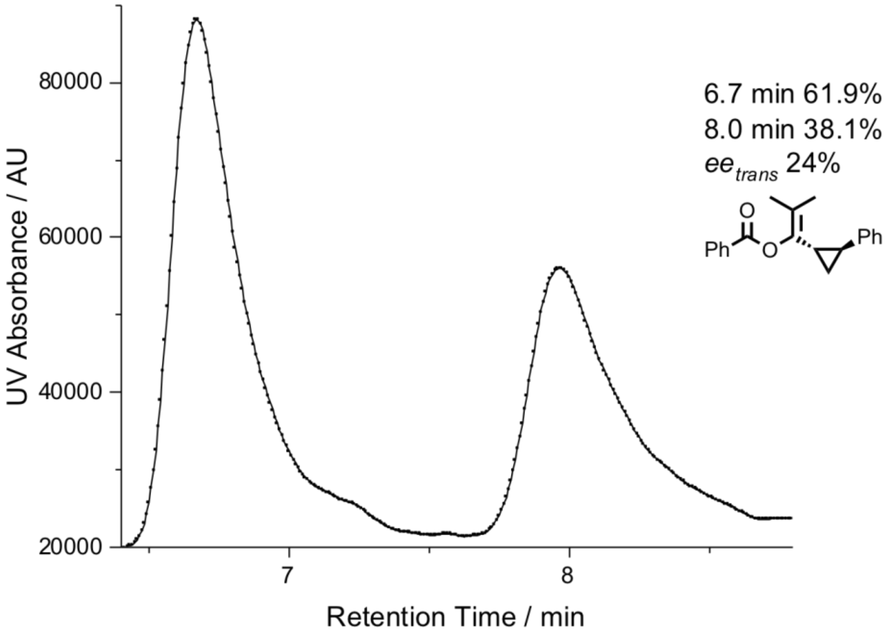

Figure S84: Chiral Stationary Phase HPLC (RegisPack, *n*-hexane-isopropanol 99 : 1, 303 K, load Et_2O, flowrate 0.75 mLmin^{-1}) of 62 : 38 *er trans*-**9**. Retention times (min): 6.7, 8.0.

Cyclopropane **10**

**Catalyst**	**Yield / %**	***dr***	***er*cis**
$(Ph_3P)AuCl$[a]	86	4.4 : 1	1 : 1
[Au((*R*mp)-**6**)(Cl)][b]	53	92 : 8	75 : 25

Table S5. Summary of reactions leading to cyclopropanes **10**.

cis-**10**

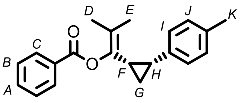

δ_H ($CDCl_3$, 400 MHz) 7.88 (2H, dd, *J* = 7.9, 1.1, ***H*C**), 7.57 (1H, tt, *J* = 7.4, 1.3, ***H*A**), 7.42 (2H, tt, *J* = 7.8, 1.5, ***H*B**), 7.06 (2H, d, *J* = 8.1, ***H*J**), 7.00 (2H, d, *J* = 8.1, ***H*I**), 2.35 (3H, s, ***H*K**), 2.34-2.25 (2H, m, ***H*F**, ***H*G**), 1.66 (3H, s, ***H*E**), 1.49 (3H, s, ***H*D**), 1.26 (1H, td, *J* = 8.9, 5.4, ***H*H**), 1.07 (1H, dt, *J* = 6.7, 5.9, ***H*H'**).

δ_C ($CDCl_3$, 101 MHz) 164.8, 138.9, 136.3, 135.1, 133.1, 130.0, 130.0, 128.5, 128.4, 127.7, 123.4, 23.6, 21.3, 21.2, 18.8, 17.8, 11.7.

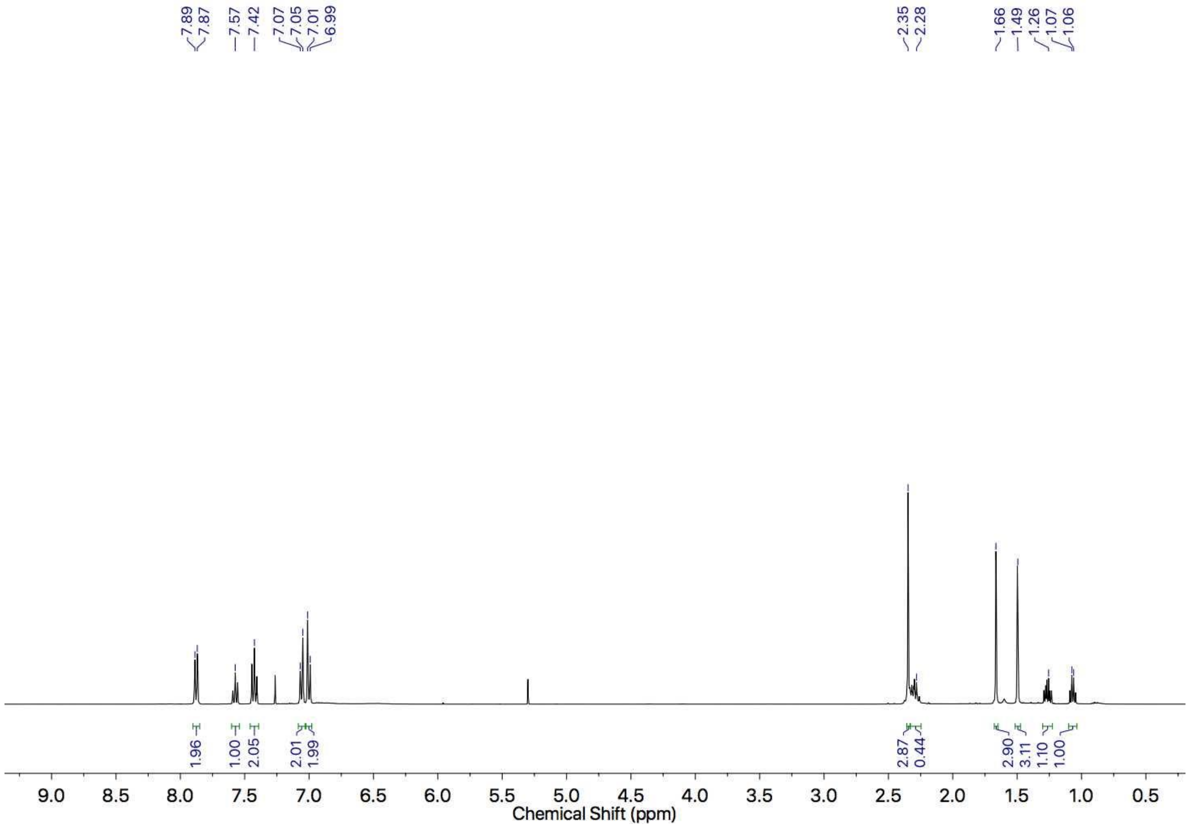

Figure S85: ^1H NMR (400 MHz, $CDCl_3$) of *cis*-**10**.

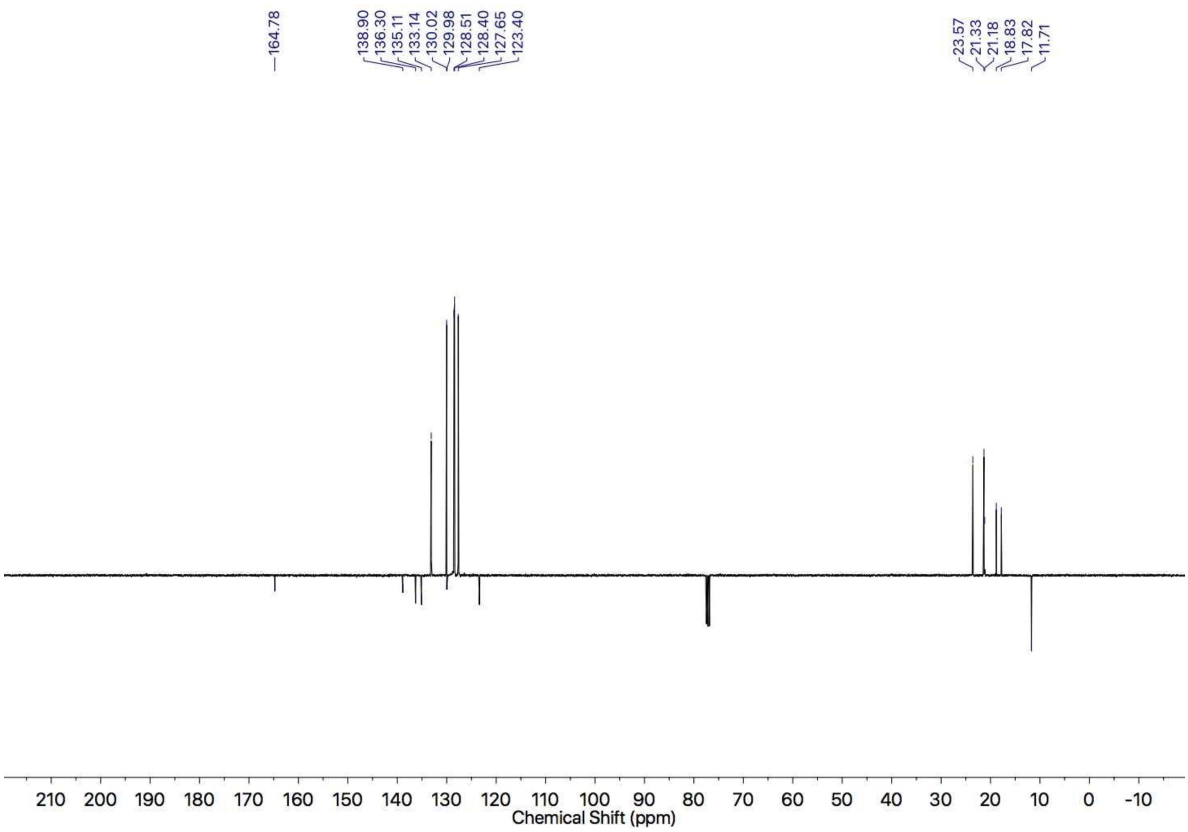

Figure S86: JMOD NMR (101 MHz, $CDCl_3$) of *cis*-**10**.

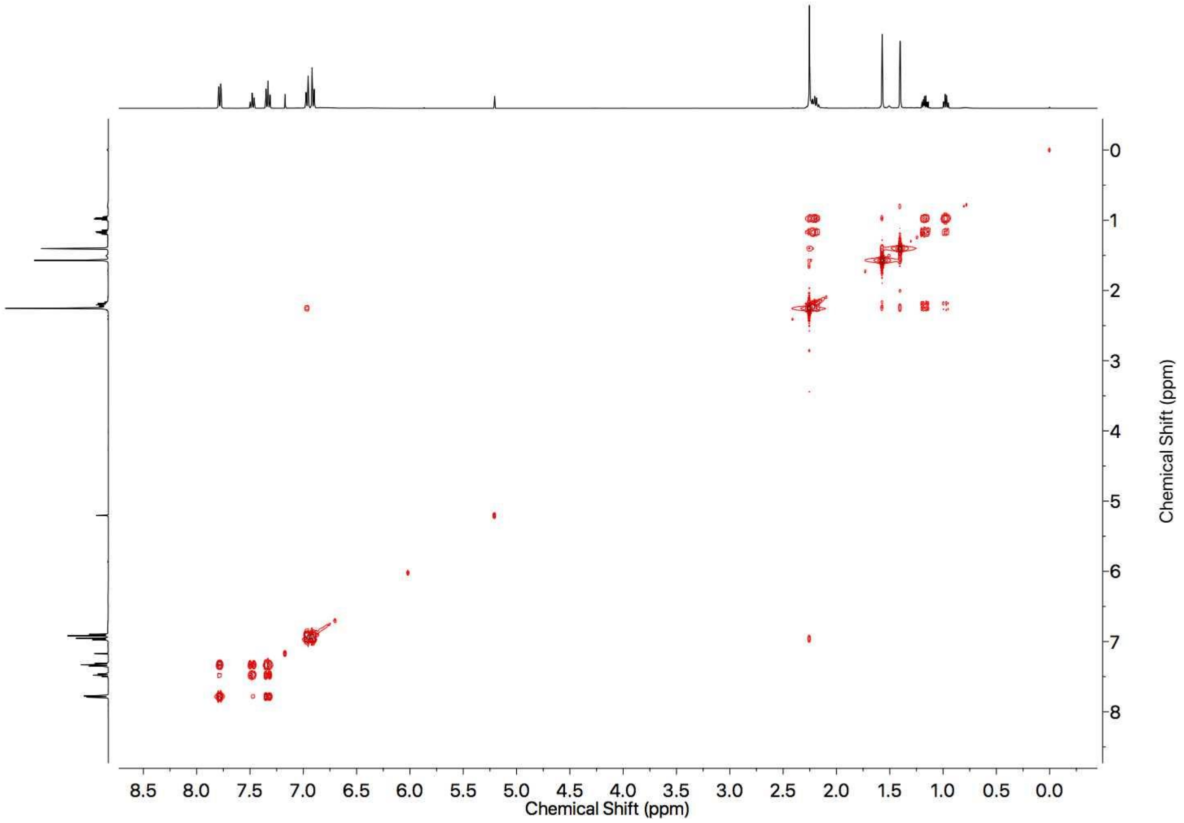

Figure S87: COSY NMR ($CDCl_3$) of *cis*-**10**.

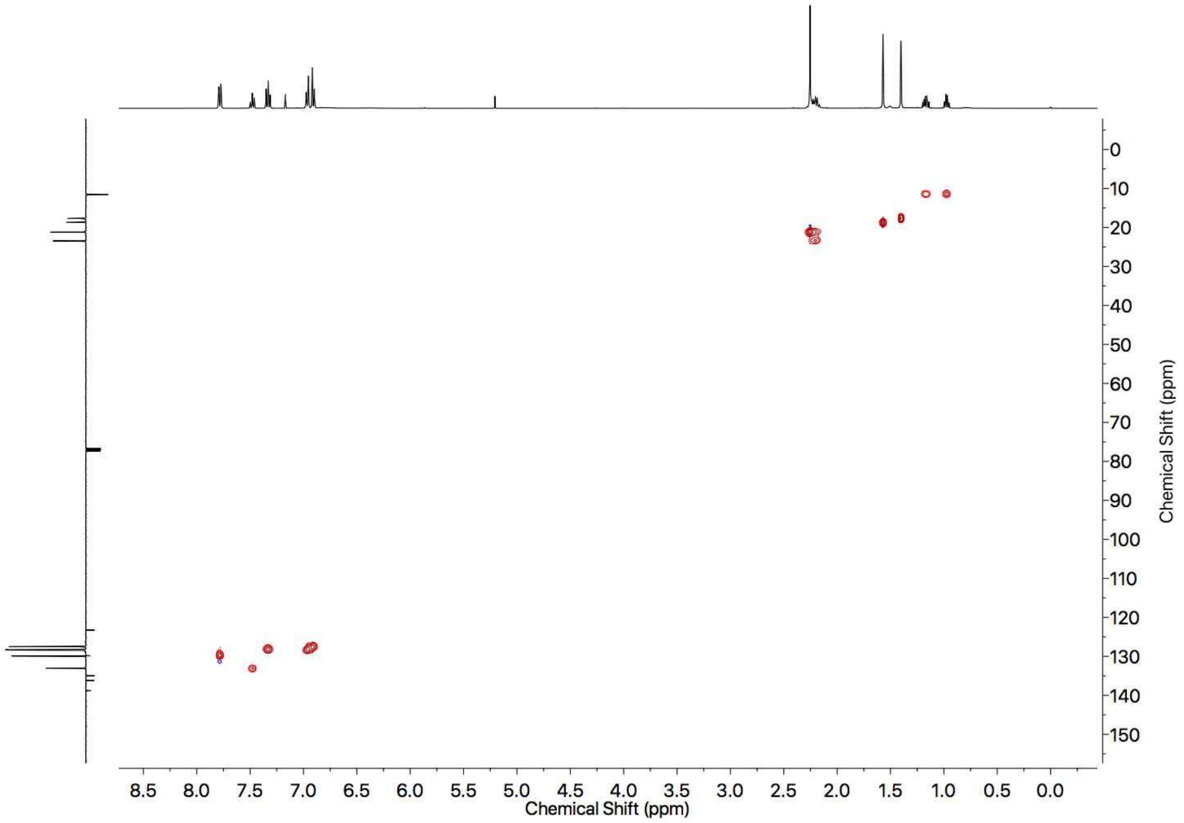

Figure S88: HSQC NMR ($CDCl_3$) of *cis*-**10**.

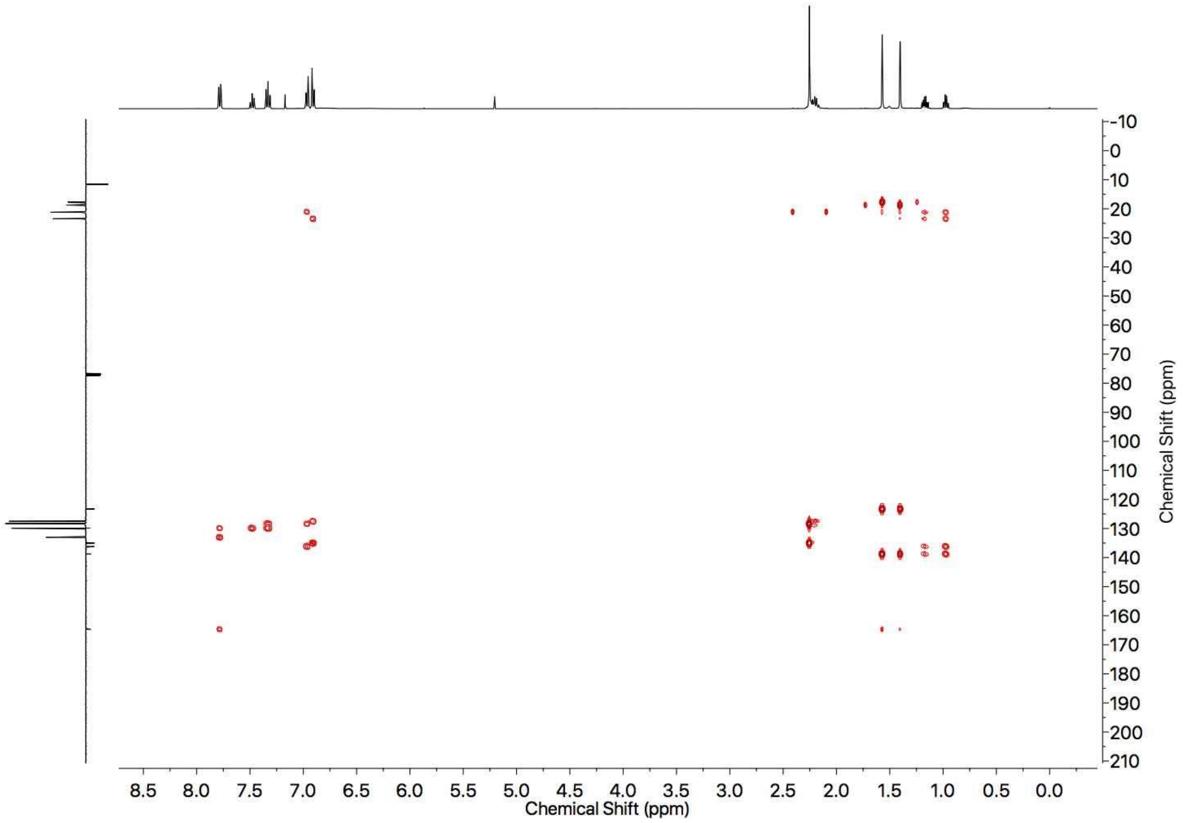

Figure S89: HMBC NMR ($CDCl_3$) of *cis*-**10**.

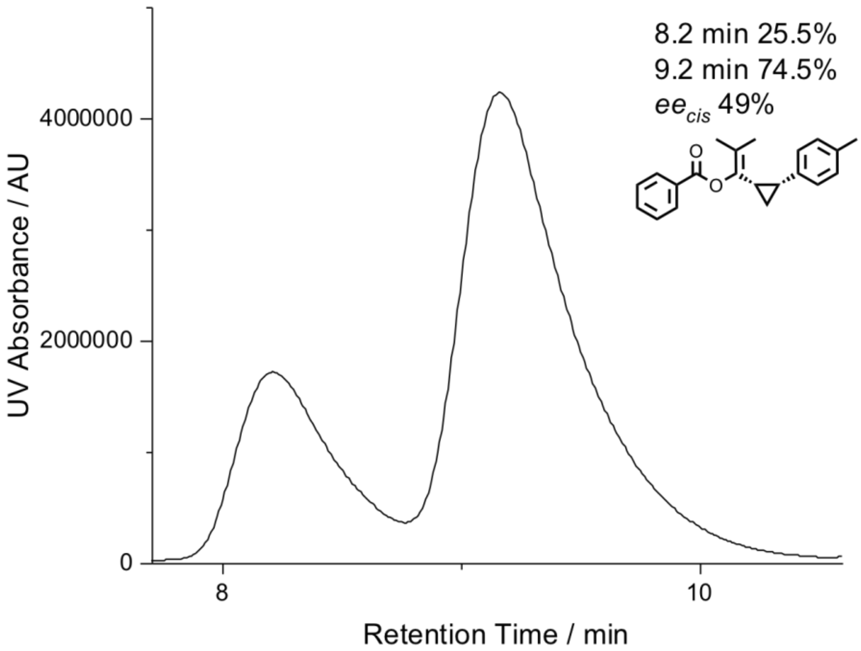

Figure S90: Chiral Stationary Phase HPLC (RegisPack, *n*-hexane-isopropanol 99.5 : 0.5, 303 K, load petrol, flowrate 0.75 $mLmin^{-1}$) of 25 : 75 *er cis*-**10**. Retention times (min): 8.2, 9.2. The absolute stereochemistry of the products was not determined. The (1*S*,2*R*)-**10** isomer is shown for illustrative purposes only.

Cyclopropane **11**

**Catalyst**	**Yield / %**	***dr***	***er*$_{cis}$**
(Ph_3P)AuCl[a]	66	4.5 : 1	1 : 1
[Au((R_{mp})-**6**)(Cl)][b]	25	25 : 1	73 : 27

Table S6. Summary of reactions leading to cyclopropanes **11**.

cis-**11**

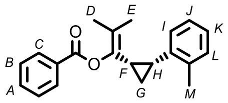

δ_H ($CDCl_3$, 400 MHz) 7.68 (2H, dd, *J* = 8.3, 1.2, ***H***$_C$), 7.54 (1H, tt, *J* = 7.4, 1.2, ***H***$_A$), 7.37 (2H, dd, *J* = 7.9, 7.6, ***H***$_B$), 7.14-7.11 (2H, m, ***H***$_L$, ***H***$_K$), 7.07 (1H, dt, *J* = 8.9, 3.9, ***H***$_J$), 6.96 (2H, d, *J* = 7.4, ***H***$_I$), 2.43 (1H, td, *J* = 8.9, 5.7, ***H***$_F$), 2.34 (3H, s, ***H***$_M$), 2.28 (1H, td, *J* = 8.6, 6.8, ***H***$_G$), 1.83 (3H, s, ***H***$_E$), 1.44 (3H, s, ***H***$_D$), 1.29 (1H, td, *J* = 8.9, 5.4, ***H***$_H$), 1.18 (1H, dt, *J* = 6.2, 5.9, ***H***$_{H'}$).

δ_C ($CDCl_3$, 101 MHz) 164.4, 139.4, 138.3, 137.2, 133.1, 130.2, 129.7, 129.6, 128.3, 127.1, 125.9, 125.4, 122.1, 21.2, 20.2, 19.8, 19.0, 18.2, 10.3.

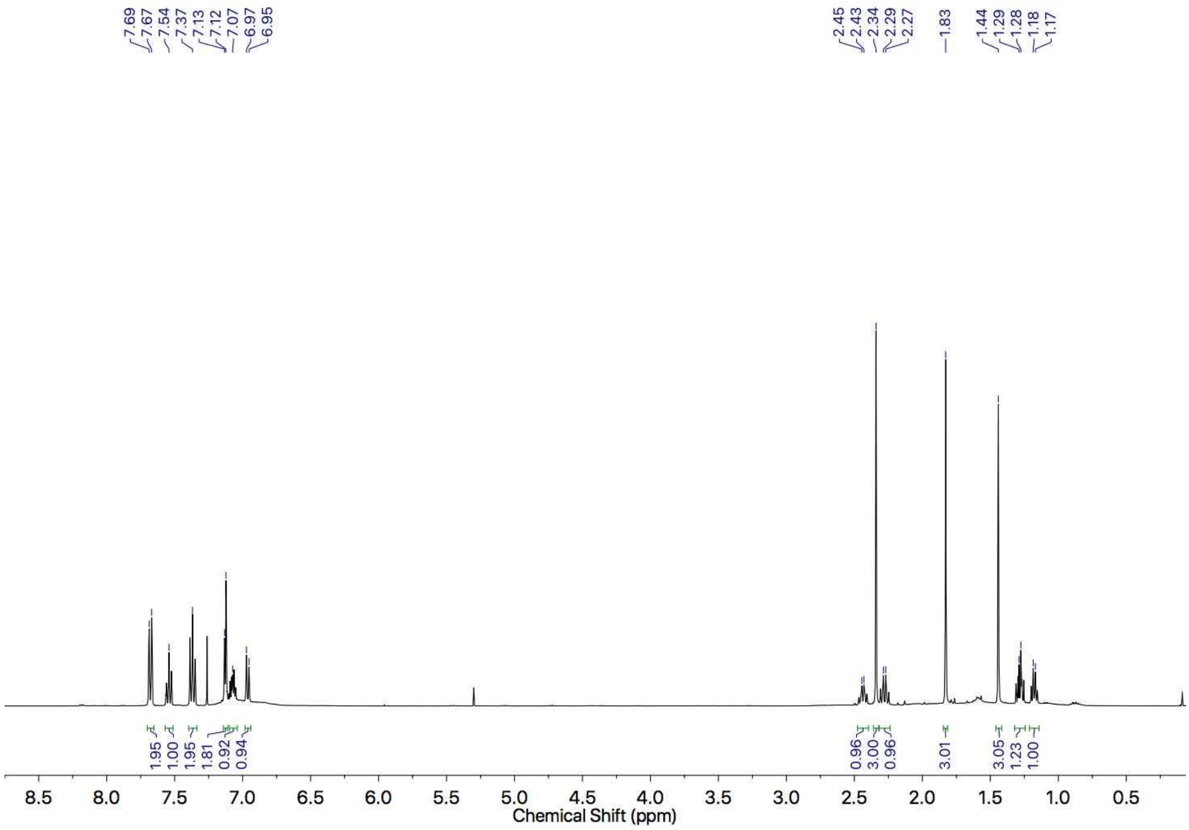

Figure S91: ^{1}H NMR (400 MHz, $CDCl_3$) of *cis*-**11**.

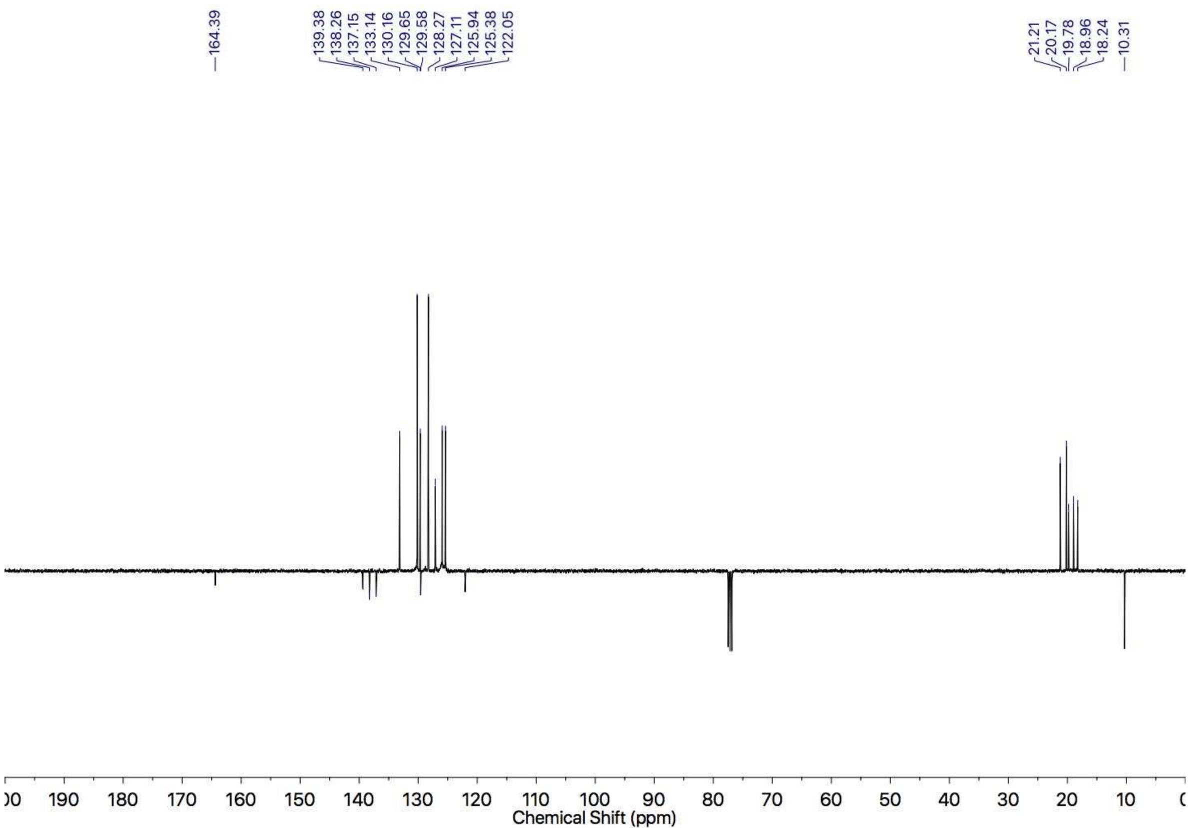

Figure S92: JMOD NMR (101 MHz, $CDCl_3$) of *cis*-**11**.

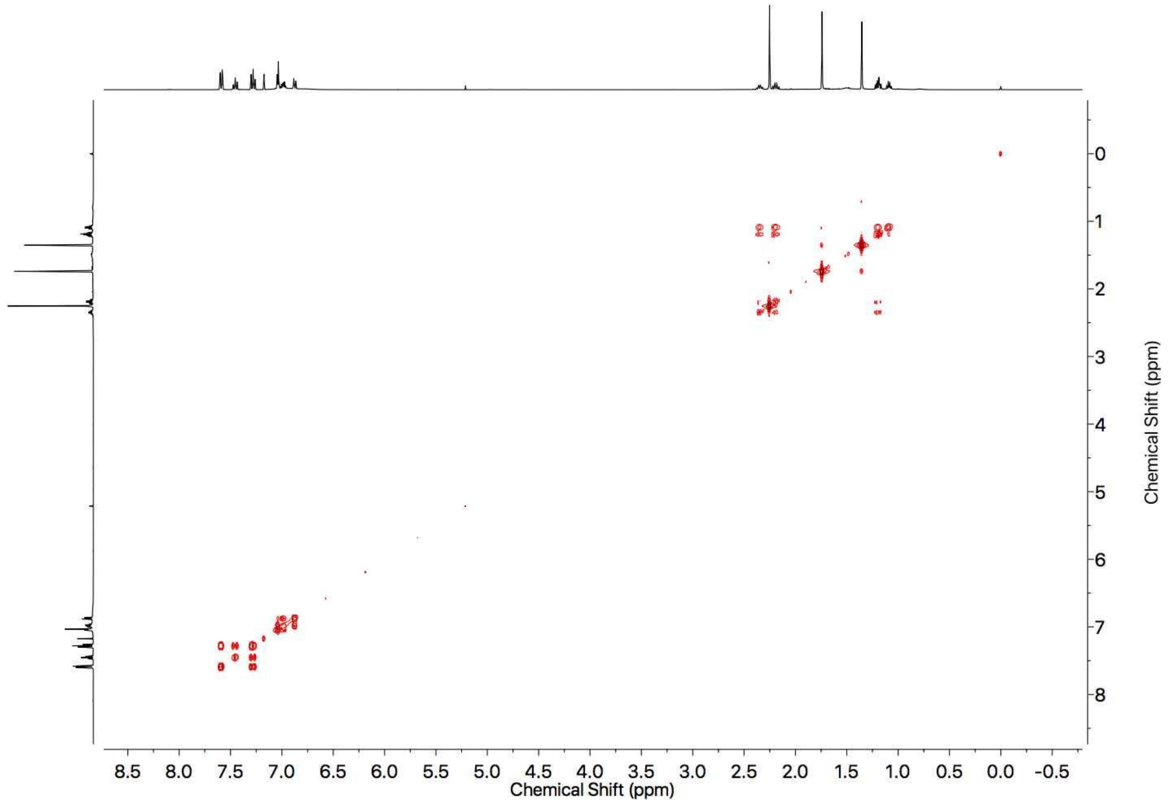

Figure S93: COSY NMR ($CDCl_3$) of *cis*-**11**.

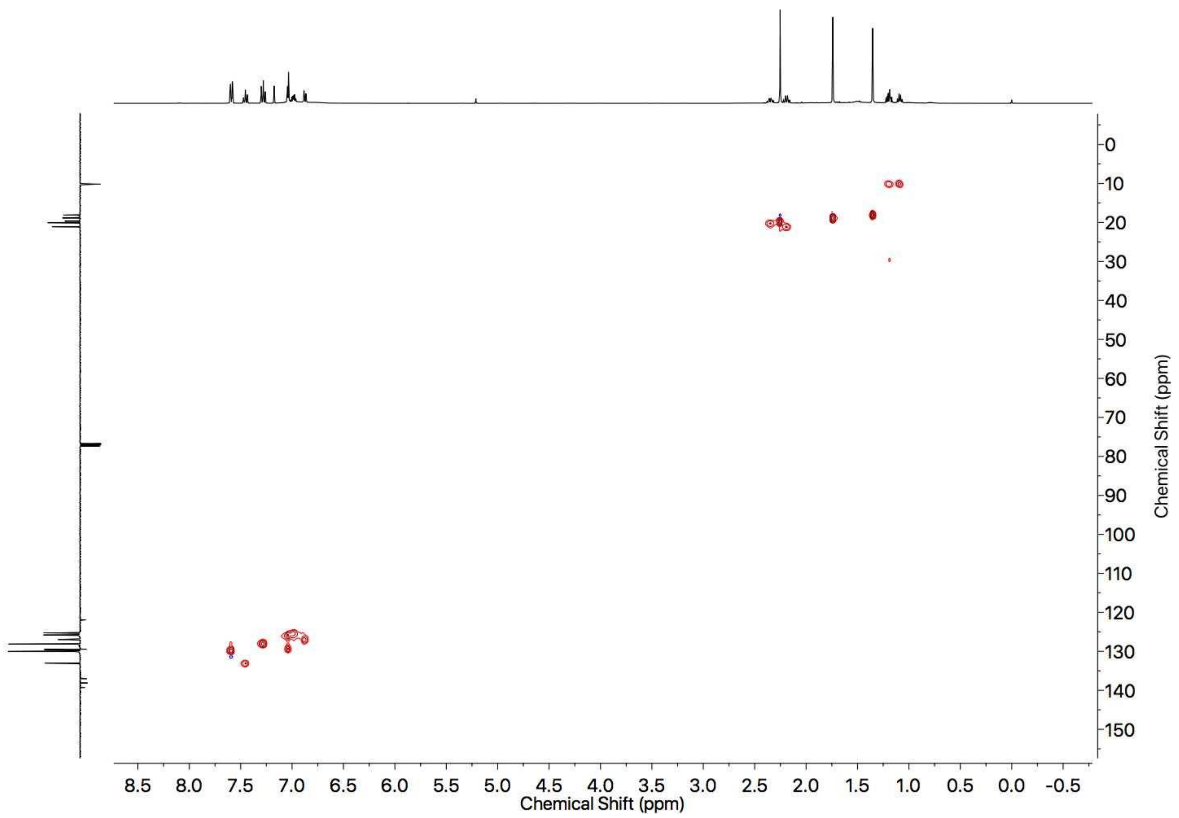

Figure S94: HSQC NMR ($CDCl_3$) of *cis*-**11**.

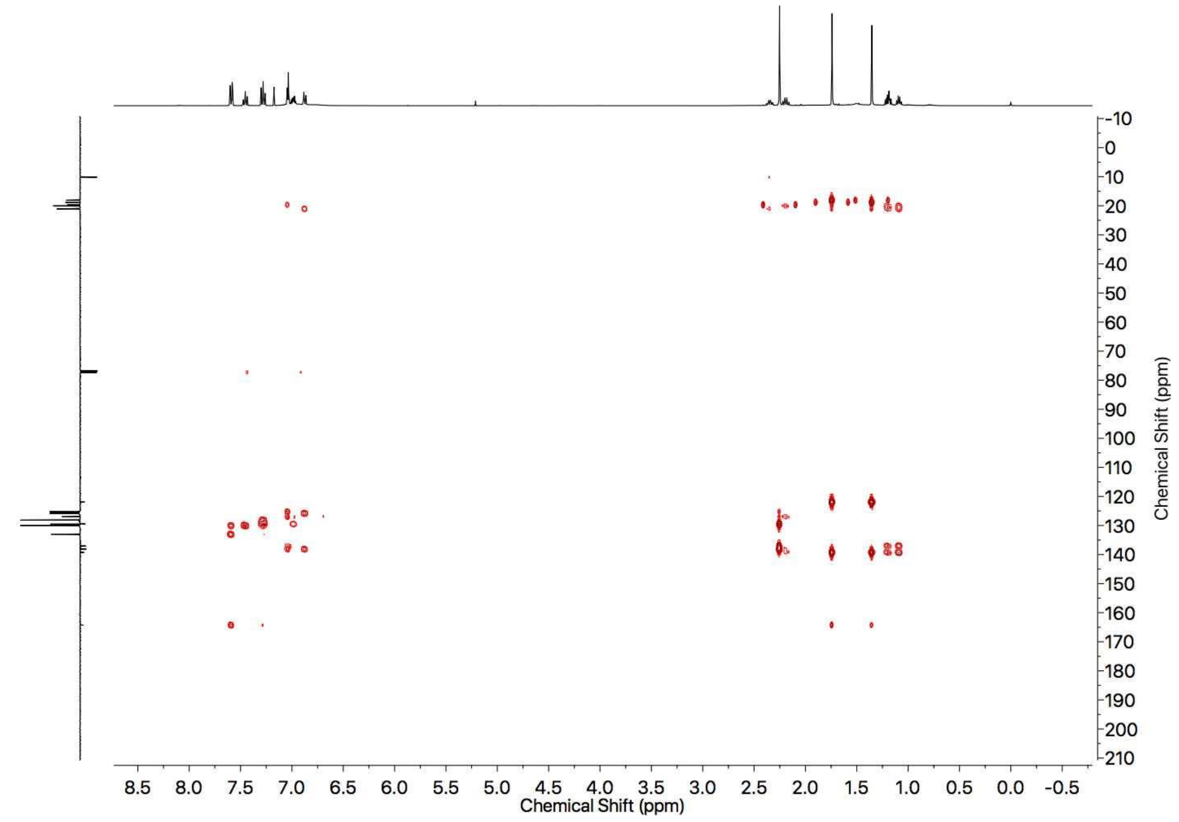

Figure S95: HMBC NMR ($CDCl_3$) of *cis*-**11**.

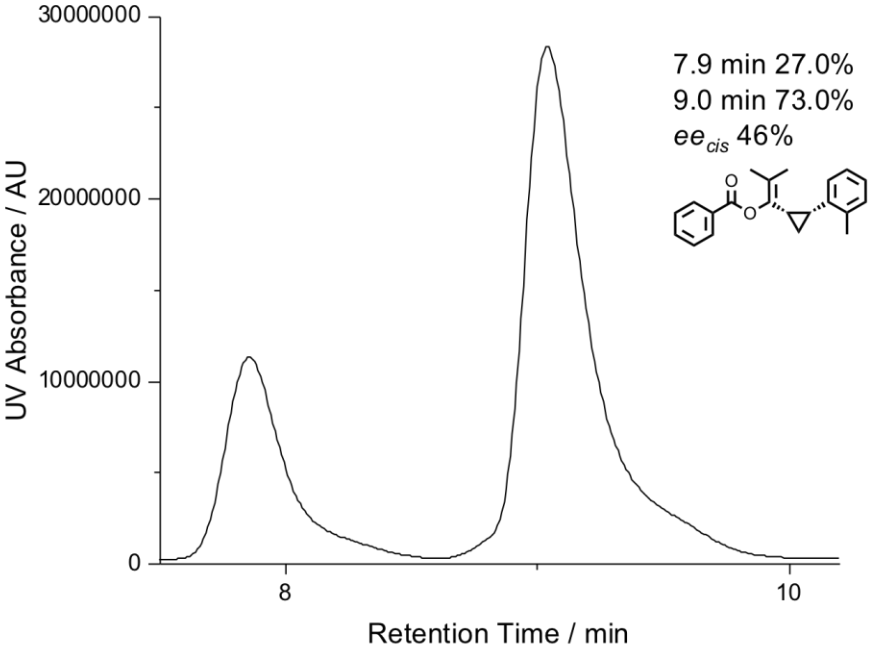

Figure S96: Chiral Stationary Phase HPLC (RegisCell, *n*-hexane-isopropanol 99.5 : 0.5, 303 K, load petrol, flowrate 0.75 mLmin^{-1}) of 27 : 73 *er cis*-**11**. Retention times (min): 7.9, 9.0. The absolute stereochemistry of the products was not determined. The (1*S*,2*R*)-**11** isomer is shown for illustrative purposes only.

Cyclopropanes **12**

**Catalyst**	**Yield / %**	***dr***	***er*cis**	***er*trans**
$(Ph_3P)AuCl$[a]	77	56 : 44	1 : 1	1 : 1
[Au((*R*mp)-**6**)(Cl)][b]	73	82 : 18	71 : 29	53 : 47

Table S7. Summary of reactions leading to cyclopropanes **12**.

cis-**12** and *trans*-**12** were not fully separated

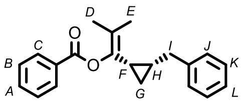

δ_H ($CDCl_3$, 400 MHz) 8.04 (2H, dd, *J* = 8.5, 1.4, ***H***C), 7.63-7.55 (1H, m, ***H***A), 7.50-7.42 (1H, m, ***H***B), 7.31-7.12 (5H, m, ***H***J, ***H***K, ***H***L), 2.71 (1H, dd, *J* = 14.8, 6.5, ***H***I), 2.59 (1H, dd, *J* = 14.7, 7.3, ***H***I′), 1.82 (3H, s, ***H***E), 1.76-1.70 (1H, m, ***H***F), 1.58 (3H, s, ***H***D), 1.31-1.25 (1H, m, ***H***G), 0.84 (1H, dt, *J* = 8.5, 5.1, ***H***H), 0.67 (1H, dt, *J* = 8.5, 5.1, ***H***H′).

δ_C ($CDCl_3$, 101 MHz) 164.8, 141.6, 141.2, 133.2, 130.0, 129.9, 128.5, 128.4, 126.1, 122.7, 39.6, 19.8, 18.9, 18.8, 18.7, 18.3, 12.5.

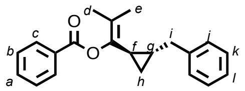

δ_H ($CDCl_3$, 400 MHz) 8.11 (2H, dd, *J* = 8.4, 1.31, ***H***c), 7.63-7.55 (1H, m, ***H***a), 7.50-7.42 (1H, m, ***H***b), 7.31-7.12 (5H, m, ***H***j, ***H***k, ***H***l), 2.85 (1H, dd, *J* = 14.8, 6.1, ***H***i), 2.50 (1H, dd, *J* = 15.0, 8.6, ***H***i′), 2.07-1.99 (1H, m, ***H***f), 1.79 (3H, s, ***H***e), 1.66 (3H, s, ***H***d), 1.40-1.31 (1H, m, ***H***g), 0.96 (1H, ddd, *J* = 13.5, 8.8, 5.0, ***H***h), 0.47 (1H, q, *J* = 5.6, ***H***h′).

δ_C ($CDCl_3$, 101 MHz) 164.8, 142.1, 140.2, 133.3, 130.0, 129.9, 128.6, 128.5, 128.4, 125.9, 120.1, 35.9, 19.5, 18.7, 18.1, 17.4, 11.5. (***C***h, ***C***j, ***C***k, ***C***l in 8 signal in the range 128.6-125.9).

HR-EI-MS m/z 306.1607 $[M^{+\cdot}]$ (calc. m/z for $C_{21}H_{22}O_2$ 306.1620).

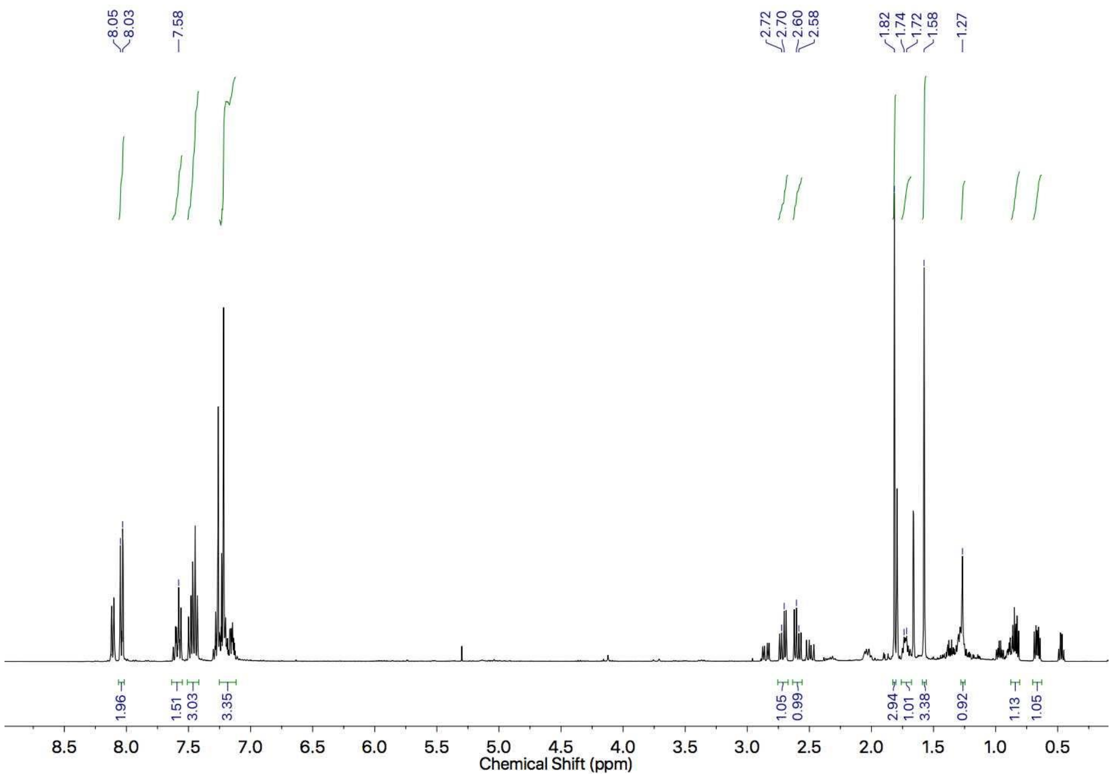

Figure S97: ^{1}H NMR (400 MHz, $CDCl_3$) of the mixture of *cis*-**12** and *trans*-**12** focussing on the major *cis* isomer.

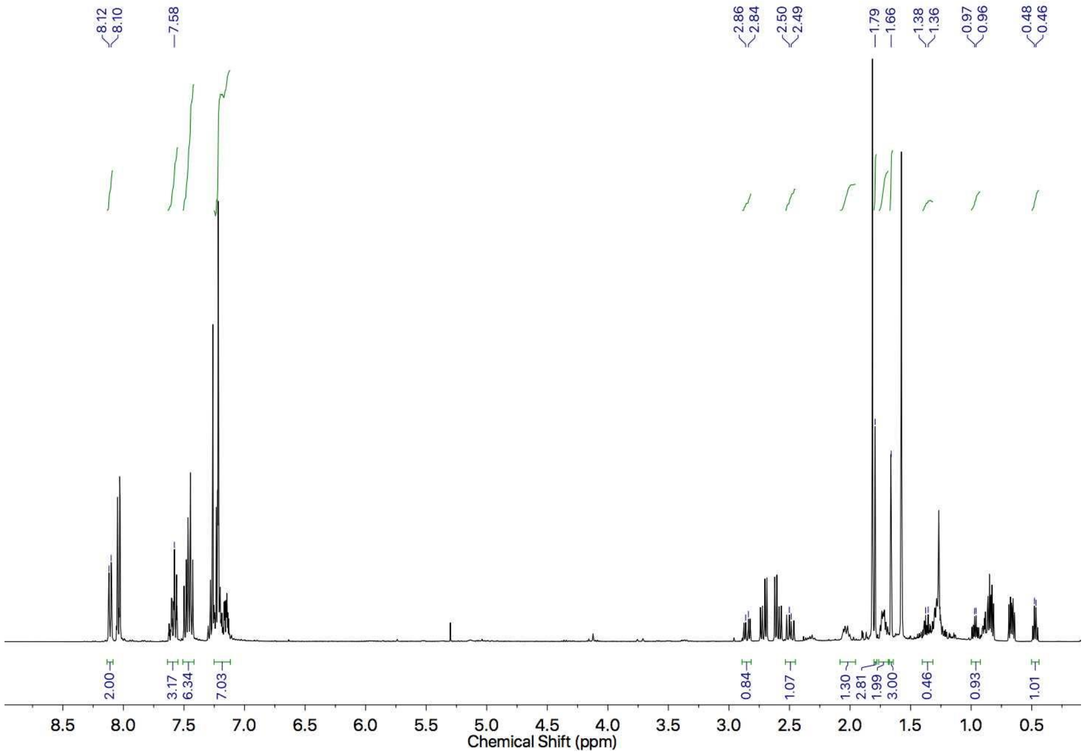

Figure S98: ^{1}H NMR (400 MHz, $CDCl_3$) of the mixture of *cis*-**12** and *trans*-**12** focussing on the minor *trans* isomer.

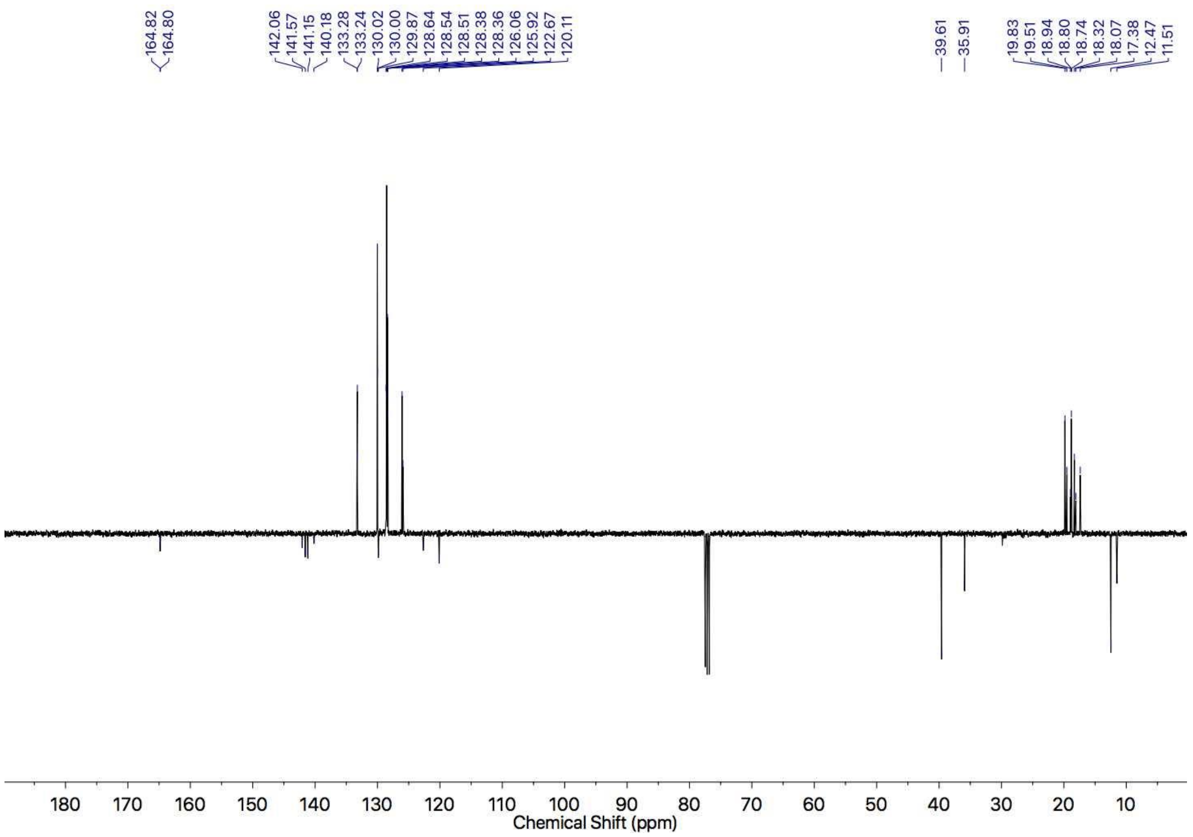

Figure S99: JMOD NMR (101 MHz, $CDCl_3$) of cyclopropanes **12**.

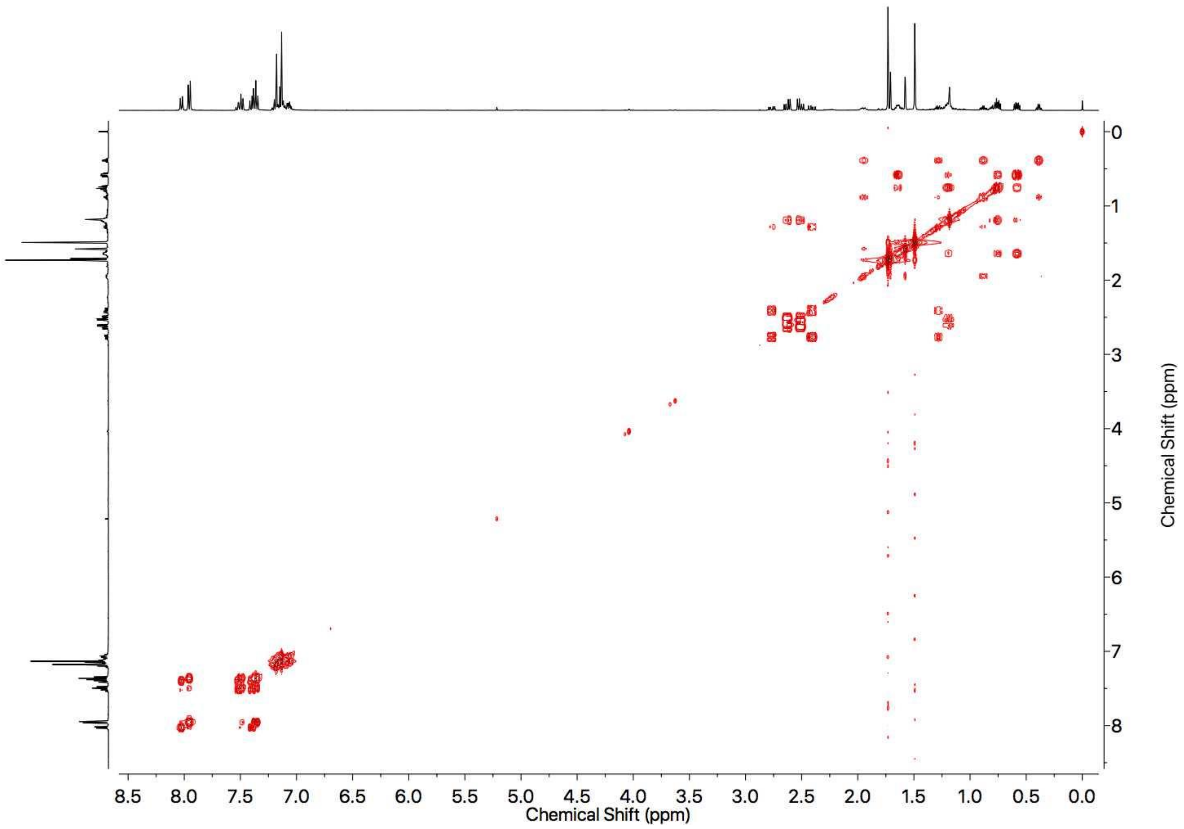

Figure S100: COSY NMR ($CDCl_3$) of cyclopropanes **12**.

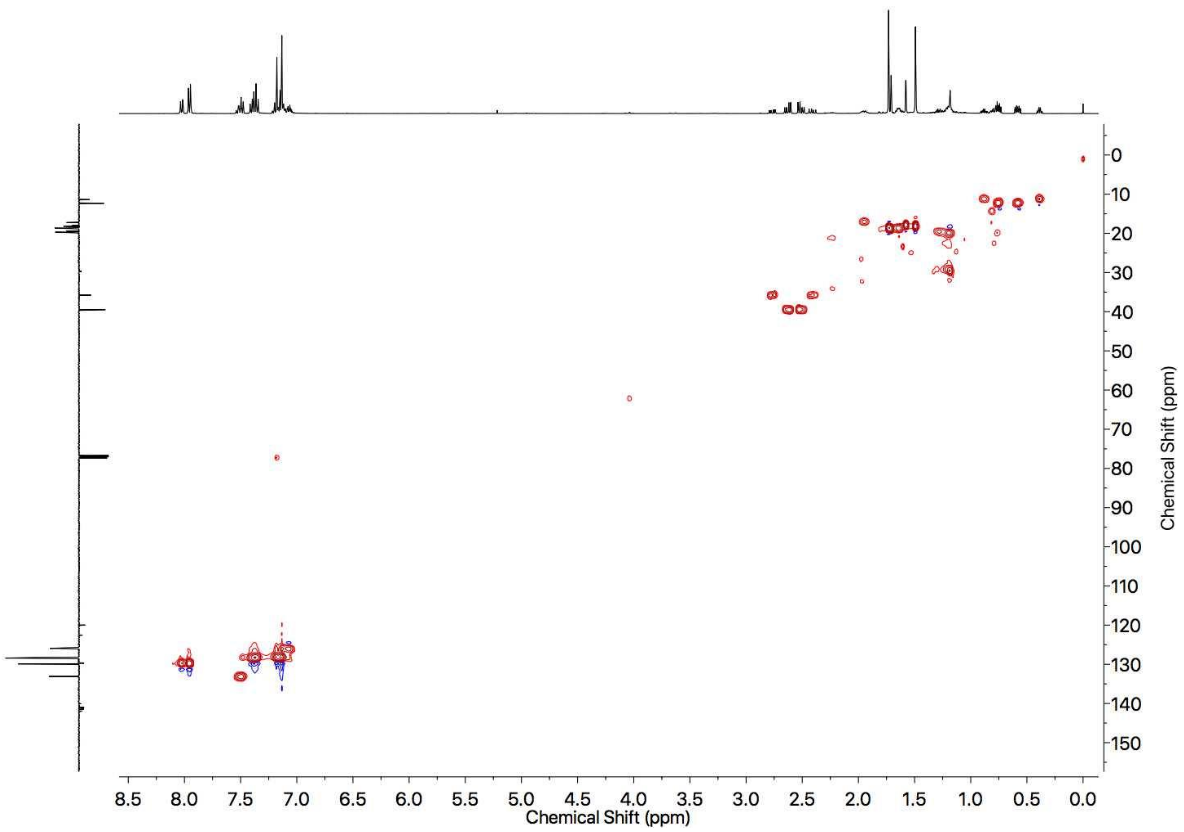

Figure S101: HSQC NMR ($CDCl_3$) of cyclopropanes **12**.

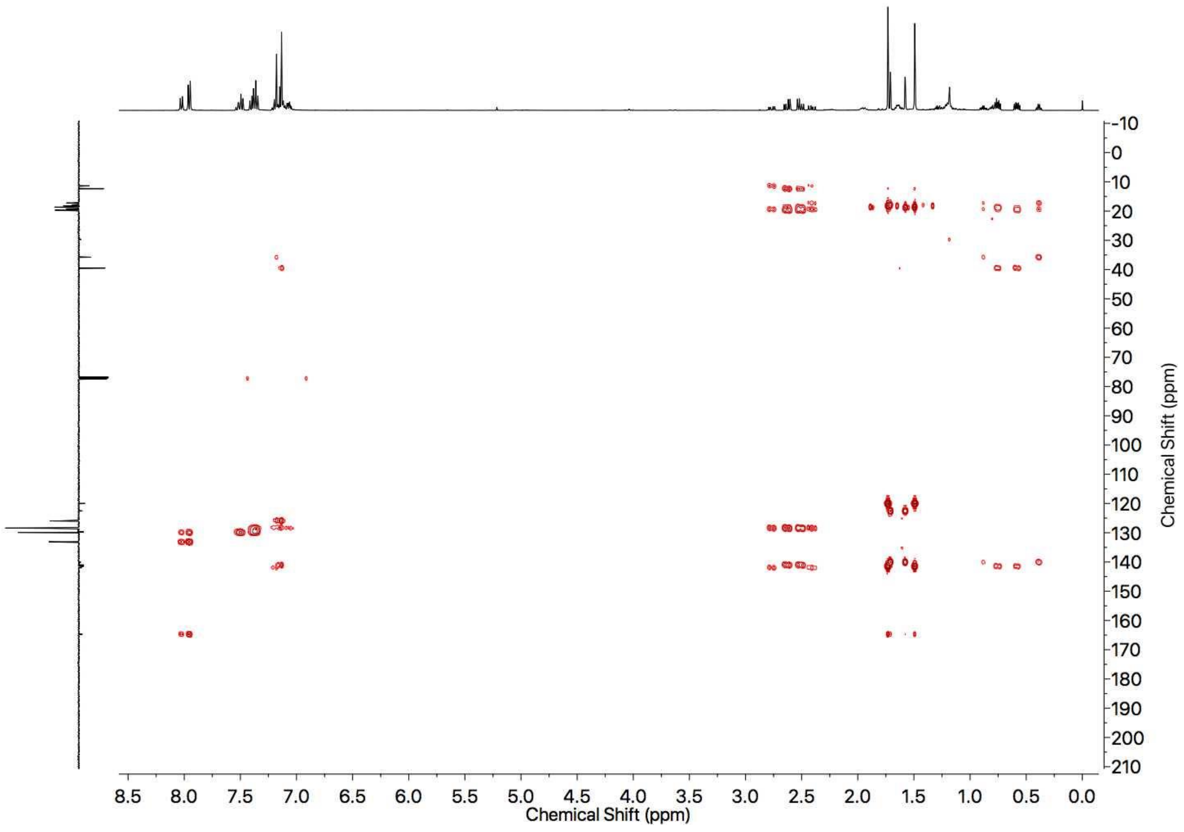

Figure S102: HMBC NMR ($CDCl_3$) of cyclopropanes **12**.

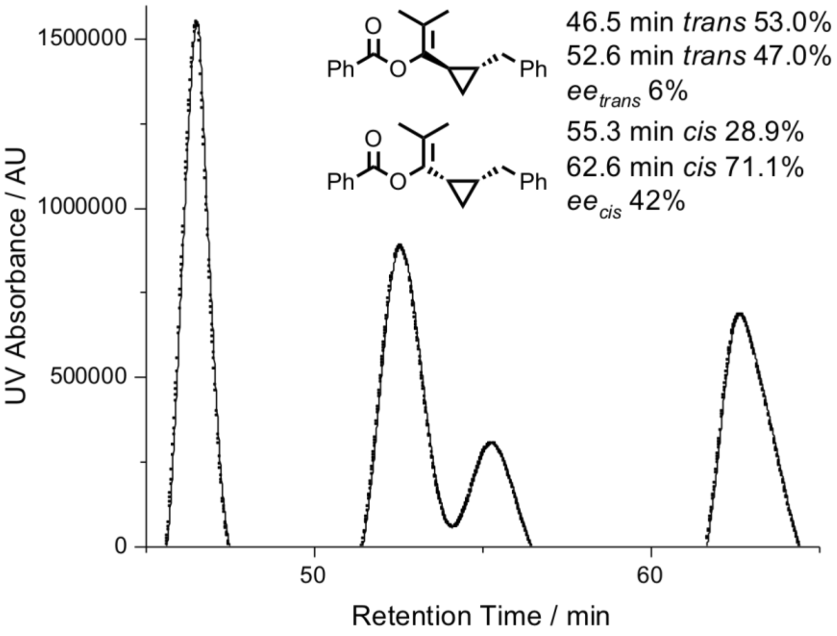

Figure S103: Chiral Stationary Phase HPLC ((S,S)Whelk, isocratic *n*-hexane-isopropanol 99.9 : 0.1, 303 K, loading solvent petrol, 5 µL injection, flowrate 0.25 mLmin^{-1}) 1 : 1 *er trans*-**12** and 64 : 36 *er cis*-**12** (*dr trans-cis* 1.8 : 1.0, not representative of the crude reaction product analysed by ^{1}H NMR). Retention times (min): *trans*-**12** 46.5, *trans*-**12** 52.6, (1*R*,2*R*)-*cis*-**12** 55.3, (1*S*,2*S*)-*cis*-**12** 62.6. The absolute stereochemistry of the products was not determined. (1*S*,2*R*)-**12** is shown for illustrative purposes only.

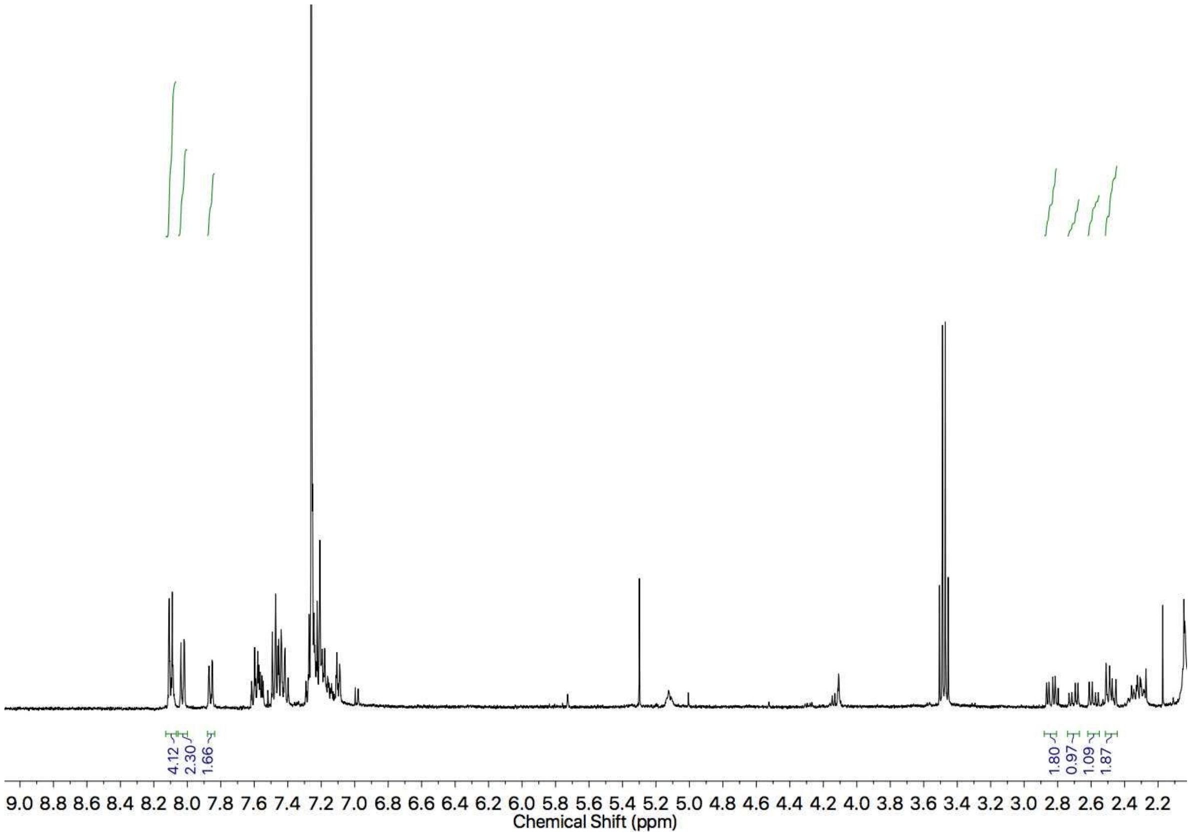

Figure S104: ^{1}H NMR (400 MHz, $CDCl_3$) of *trans*-**12** and *cis*-**12** diastereomer mixture (*dr* 1.8 : 1.0) from column chromatography fraction used for chiral HPLC.

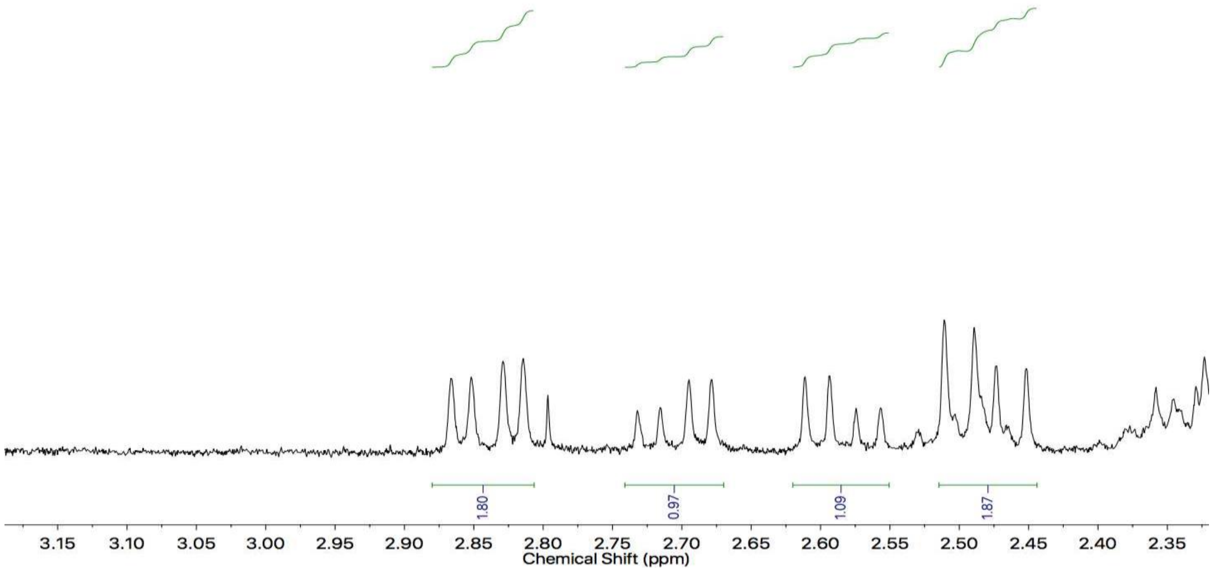

Figure S105: ^{1}H NMR (400 MHz, $CDCl_3$) of *trans*-**12** and *cis*-**12** diastereomer mixture (*dr* 1.8 : 1.0) from column chromatography fraction used for chiral HPLC.

Cyclopropanes **13**[6]

**Catalyst**	**Yield / %**	***dr***	***er*$_{cis}$**	***er*$_{trans}$**
$(Ph_3P)AuCl$	44	92 : 8	1 : 1	-
[Au((*R*$_{mp}$)-**6**)(Cl)]	90	97 : 3	55 : 45	-
(*R*)-DTBM-SEGPHOS®$(AuCl)_2$	70	>20 : 1	91 : 9	-

Table S8. Summary of reactions leading to cyclopropanes **13**.

Cis-**13**

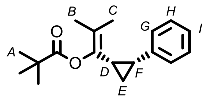

δ_H ($CDCl_3$, 400 MHz) 7.21 (2H, tt, *J* = 7.5, 1.6, ***H***$_H$), 7.14 (1H, tt, *J* = 7.4, 1.3, ***H***$_I$), 7.03 (2H, dd, *J* = 7.5, 1.3, ***H***$_G$), 2.28 (1H, d, *J* = 6.5, ***H***$_E$), 2.26 (1H, d, *J* = 6.5, ***H***$_D$), 1.48 (3H, s, ***H***$_C$), 1.41 (3H, s, ***H***$_B$), 1.26 (1H, m, ***H***$_F$), 1.22 (9H, s, ***H***$_A$), 1.00 (1H, dt, *J* = 6.0, 6.0, ***H***$_{F'}$).

δ_C ($CDCl_3$, 101 MHz) 176.9, 139.6, 138.2, 127.6, 127.4, 125.6, 123.2, 39.0, 27.4, 24.1, 21.9, 18.7, 17.4, 11.9.

HR-EI-MS m/z 272.1766 [$M^{+\cdot}$] (calc. m/z for $C_{18}H_{24}O_2$ 272.1771).

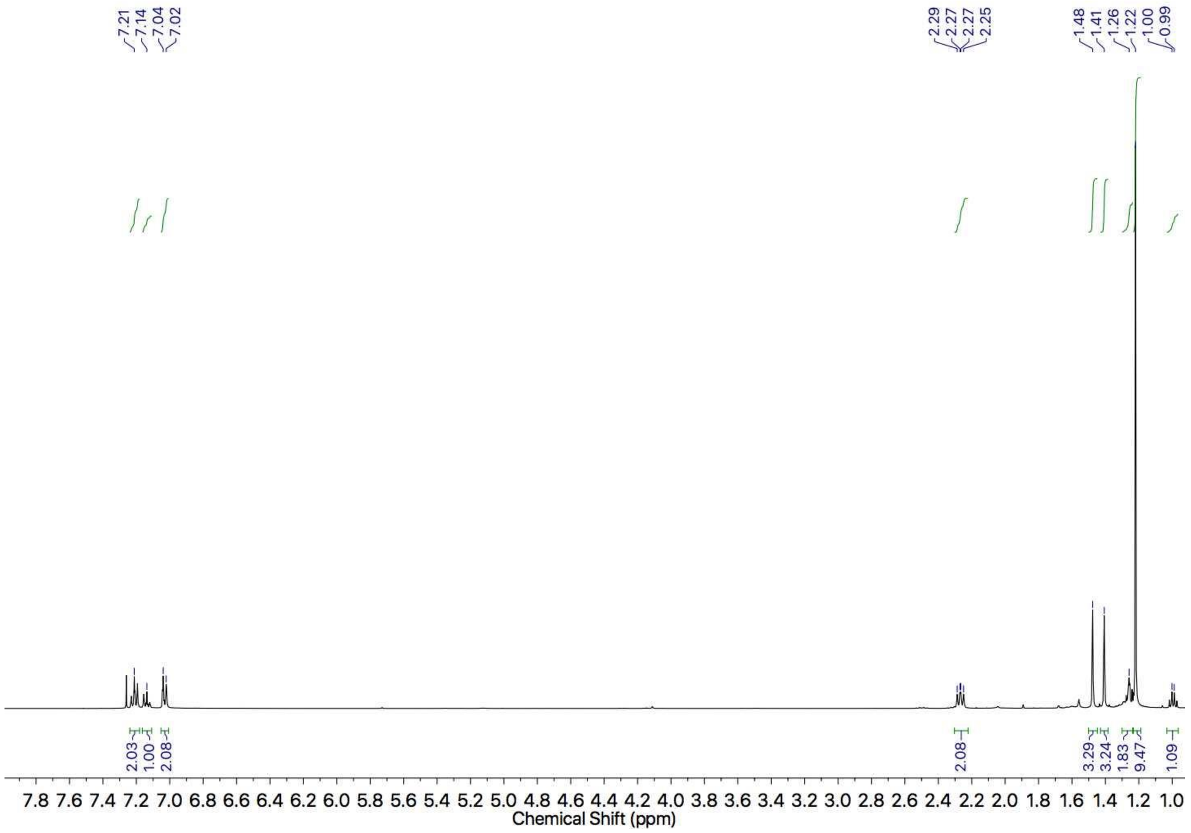

Figure S106: ^1H NMR (400 MHz, $CDCl_3$) of *cis*-**13**.

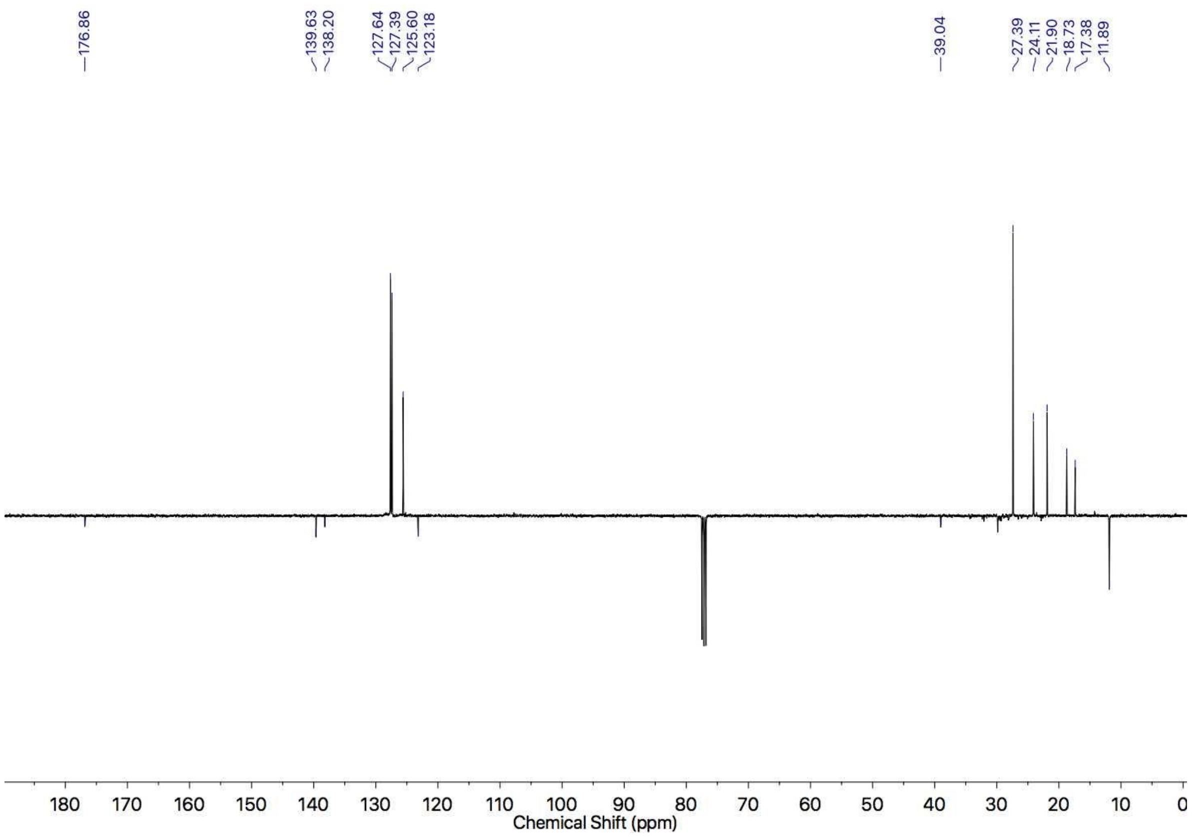

Figure S107: JMOD NMR (101 MHz, $CDCl_3$) of *cis*-**13**.

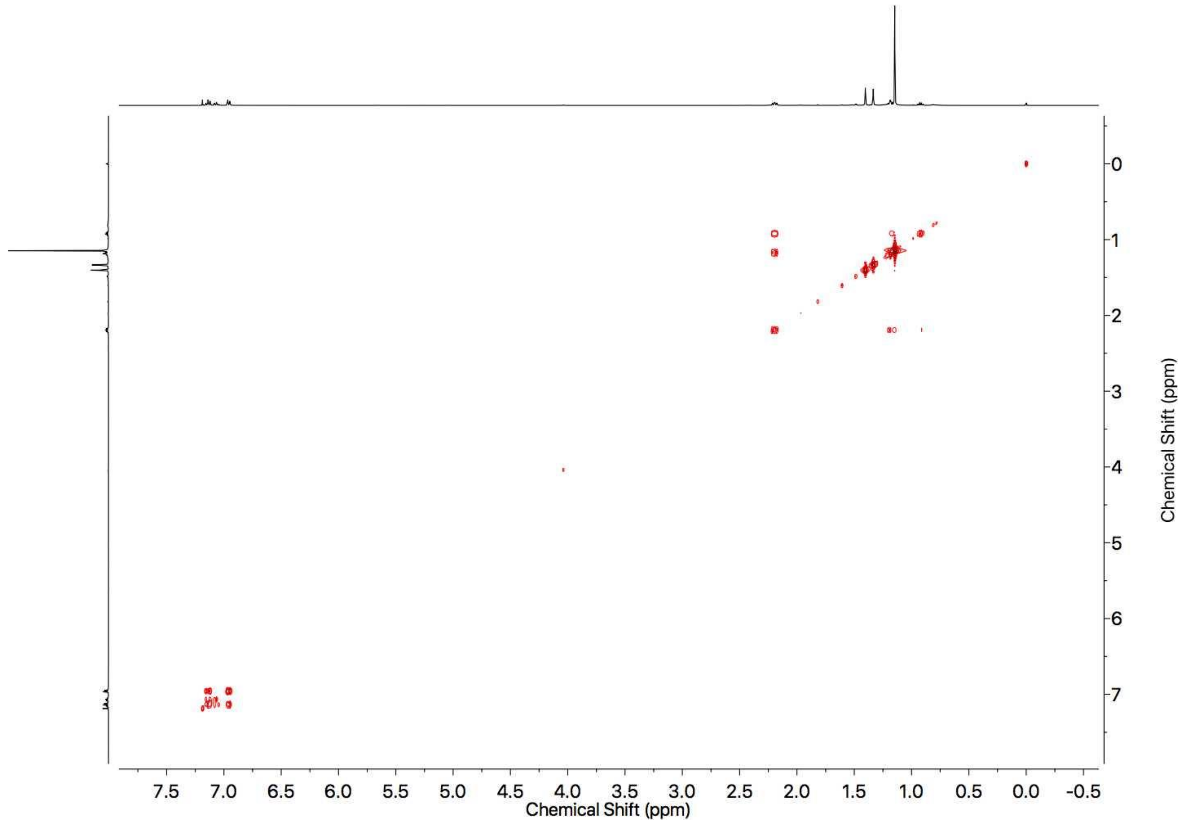

Figure S108: COSY NMR ($CDCl_3$) of *cis*-**13**.

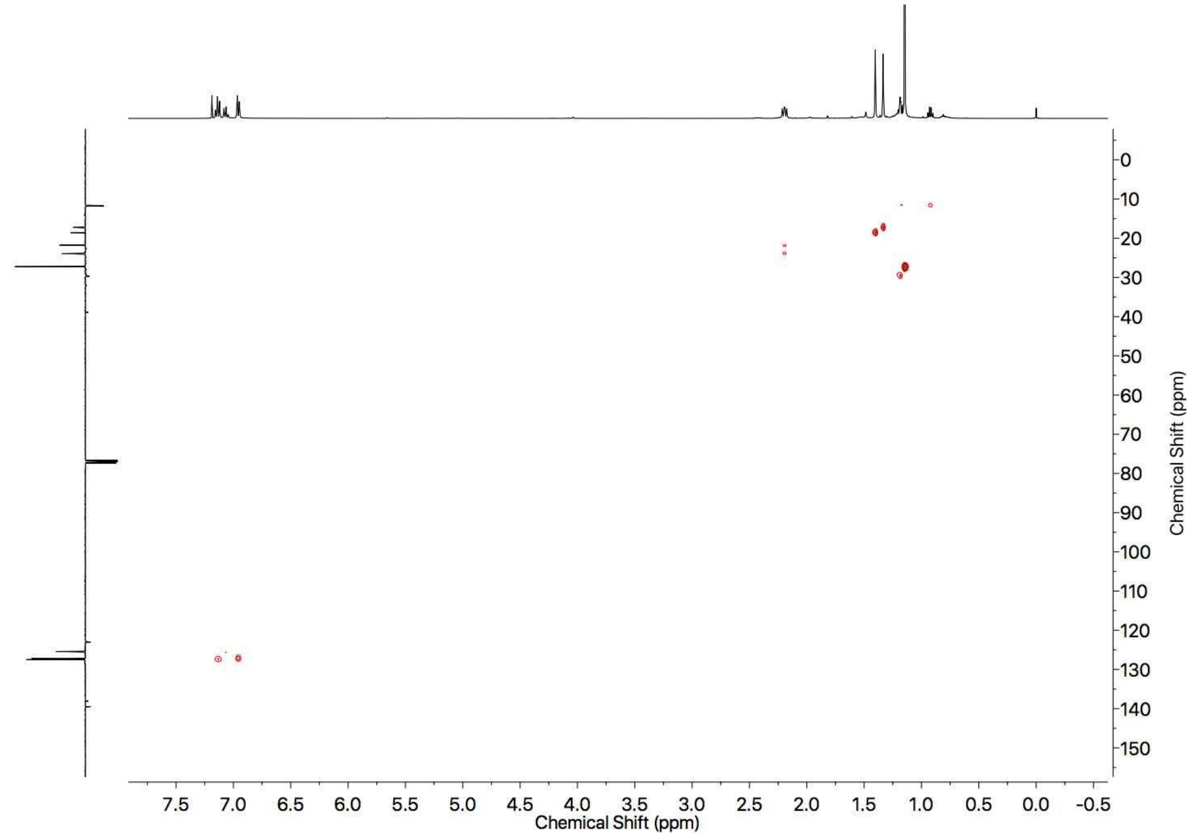

Figure S109: HSQC NMR ($CDCl_3$) of *cis*-**13**.

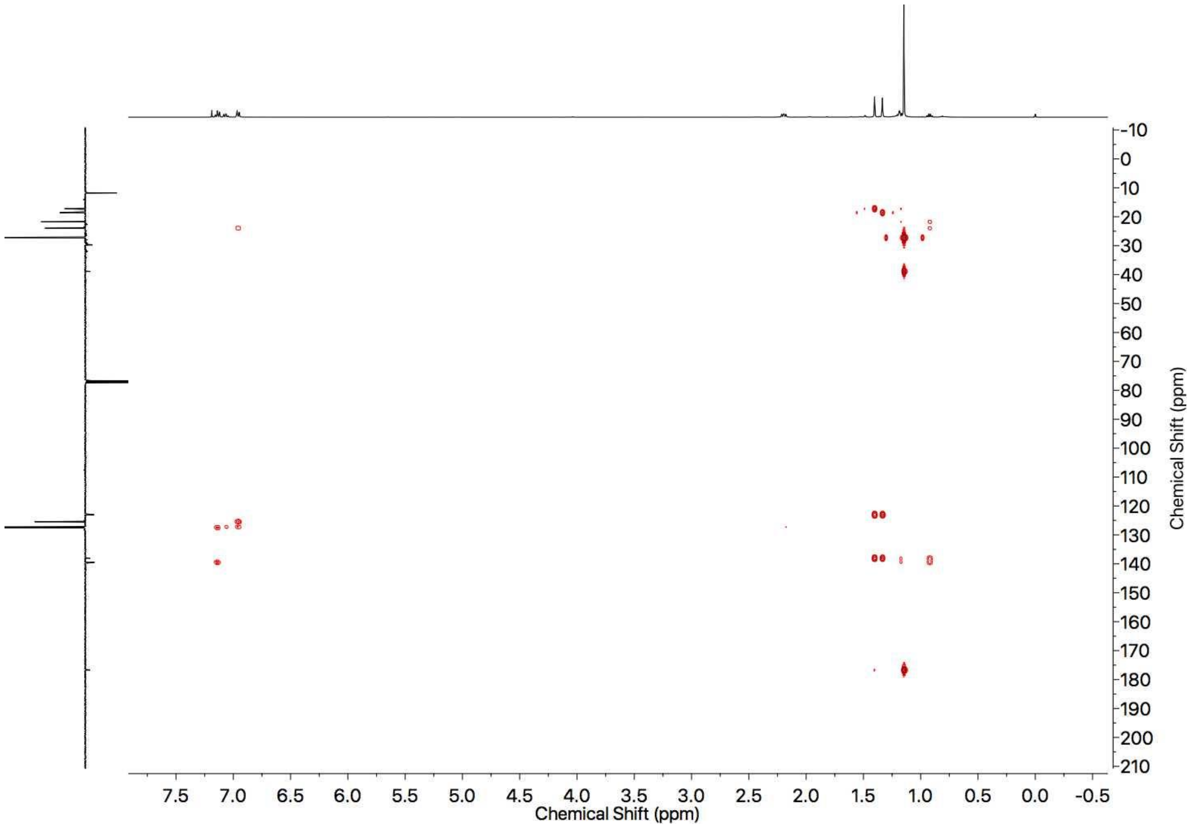

Figure S110: HMBC NMR ($CDCl_3$) of *cis*-**13**.

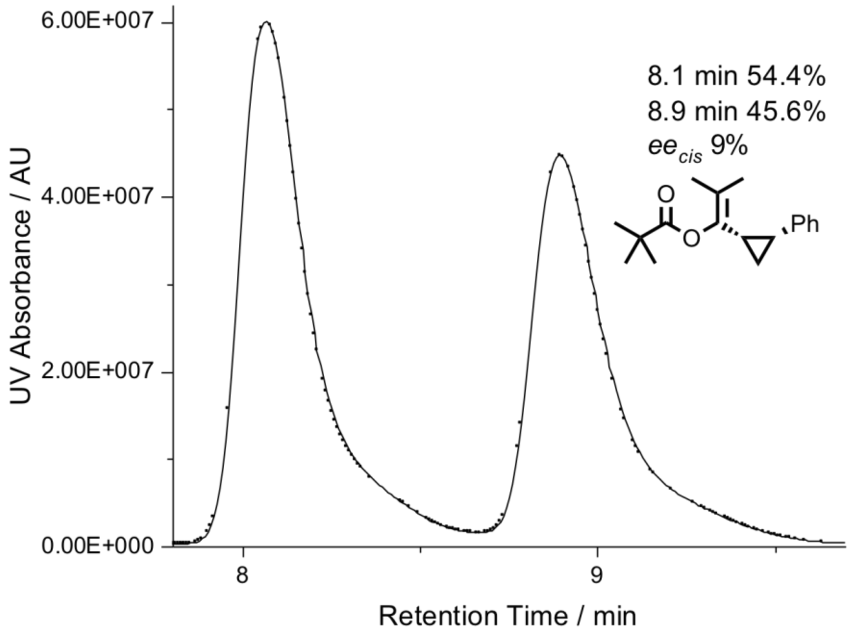

Figure S111: Chiral Stationary Phase HPLC (RegisCell, *n*-hexane-isopropanol 99.5 : 0.5, 303 K, load solvent Et_2O, flowrate 0.50 $mLmin^{-1}$) of 54 : 46 *er cis*-**13**. Retention times (min): 8.1, 8.9. The absolute stereochemistry of the products was not determined. The (1*S*,2*R*)-**13** isomer is shown for illustrative purposes only.

Cyclopropanes **14**

**Catalyst**	**Yield / %**	***dr***	***er*$_{cis}$**	***er*$_{trans}$**
$(Ph_3P)AuCl$	63	90 : 10	1 : 1	1 : 1
[Au((*R*$_{mp}$)-**6**)(Cl)]	40	97 : 3	77 : 23	-

Table S9. Summary of reactions leading to cyclopropanes **14**.

Cis-**14**

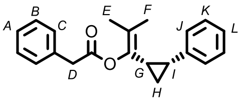

δ_H ($CDCl_3$, 400 MHz) 7.38-7.28 (5H, m, ***H***$_A$, ***H***$_B$, ***H***$_C$), 7.21 (2H, tt, *J* = 7.2, 1.7, ***H***$_K$), 7.15 (1H, tt, *J* = 7.2, 1.5, ***H***$_L$), 6.95 (2H, dt, *J* = 6.9, 1.9, ***H***$_J$), 3.61 (2H, s, ***H***$_D$), 2.27-2.21 (2H, m, ***H***$_G$, ***H***$_H$), 1.47 (3H, s, ***H***$_F$), 1.34 (3H, s, ***H***$_E$), 1.13 (1H, td, *J* = 8.8, 5.4, ***H***$_I$), 0.82 (1H, td, *J* = 6.4, 5.4, ***H***$_{I'}$).

δ_C ($CDCl_3$, 101 MHz) 169.8, 139.4, 138.4, 134.1, 129.5, 128.7, 127.7, 127.4, 127.3, 125.6, 123.5, 41.5, 24.1, 21.7, 18.6, 17.5, 11.4.

HR-EI-MS m/z 306.1607 [$M^{+\cdot}$] (calc. m/z for $C_{21}H_{22}O_2$ 306.1620).

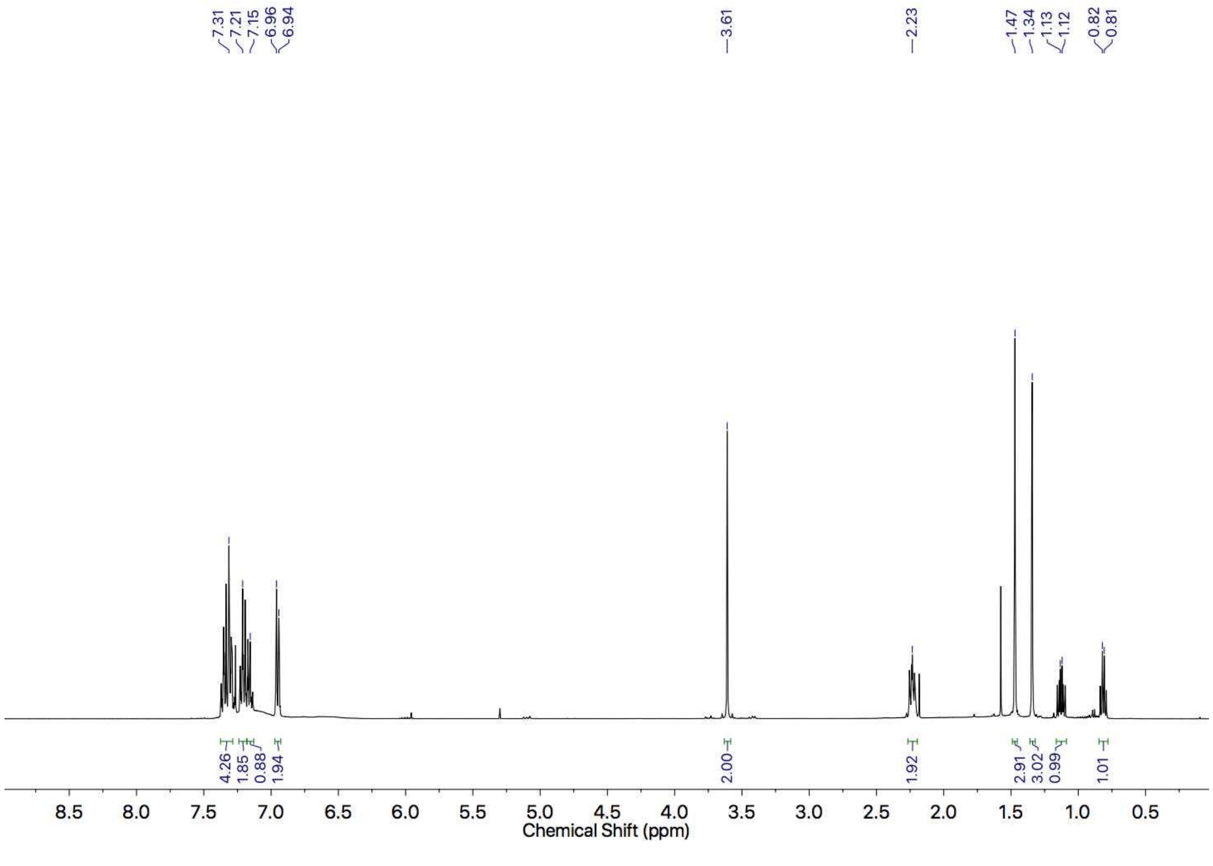

Figure S112: ^1H NMR (400 MHz, $CDCl_3$) of *cis*-**14**.

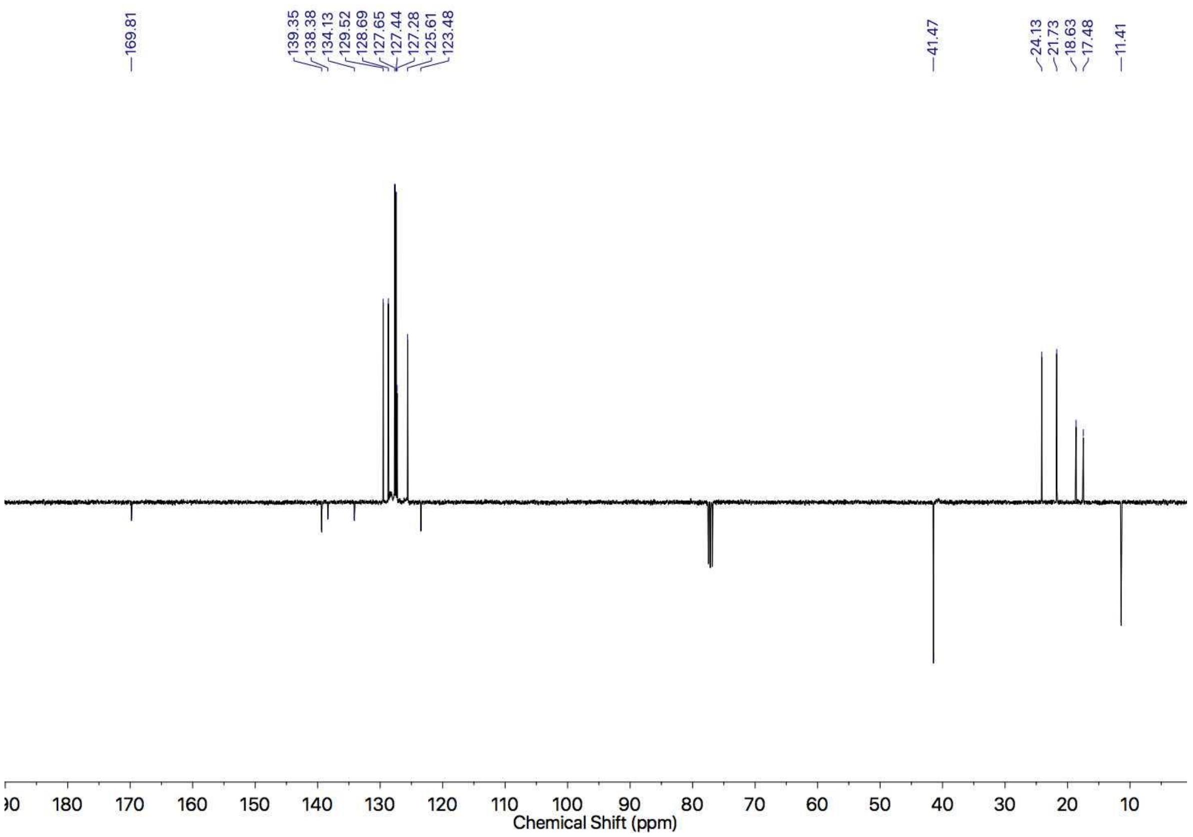

Figure S113: JMOD NMR (101 MHz, $CDCl_3$) of *cis*-**14**.

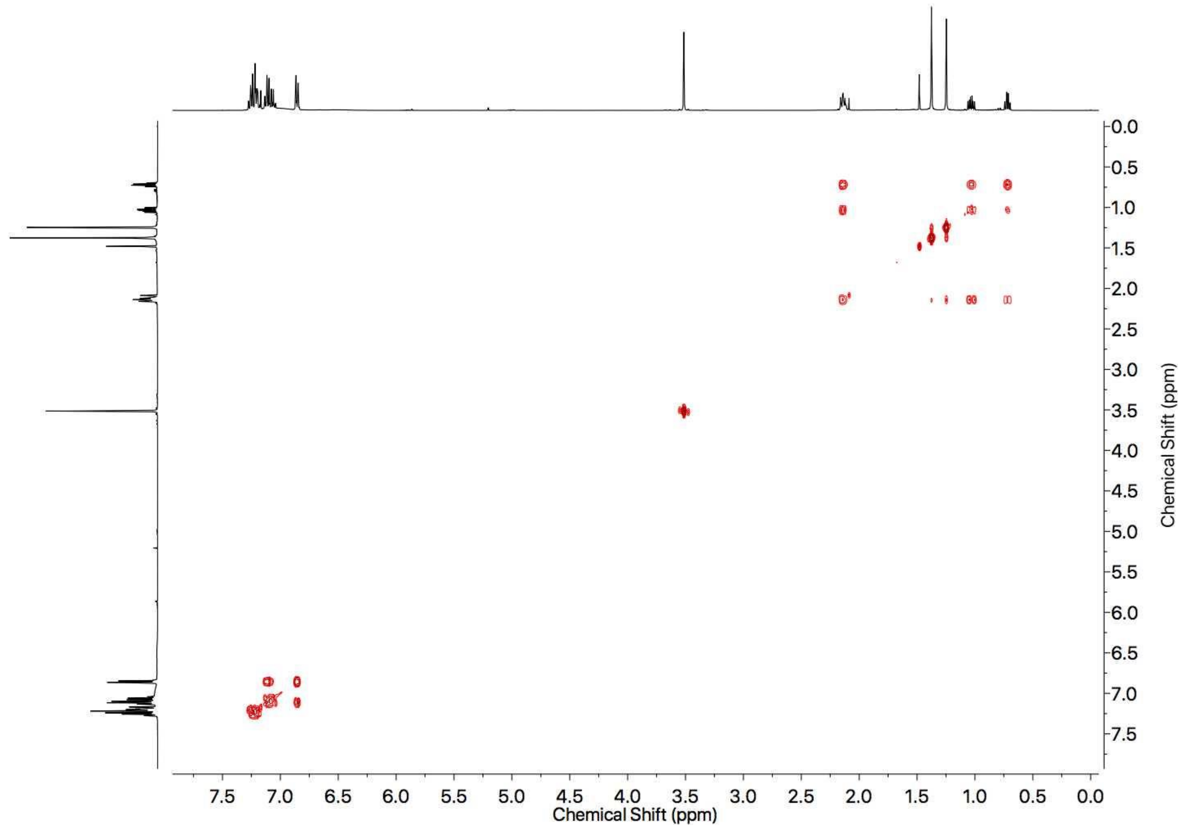

Figure S114: COSY NMR ($CDCl_3$) of *cis*-**14**.

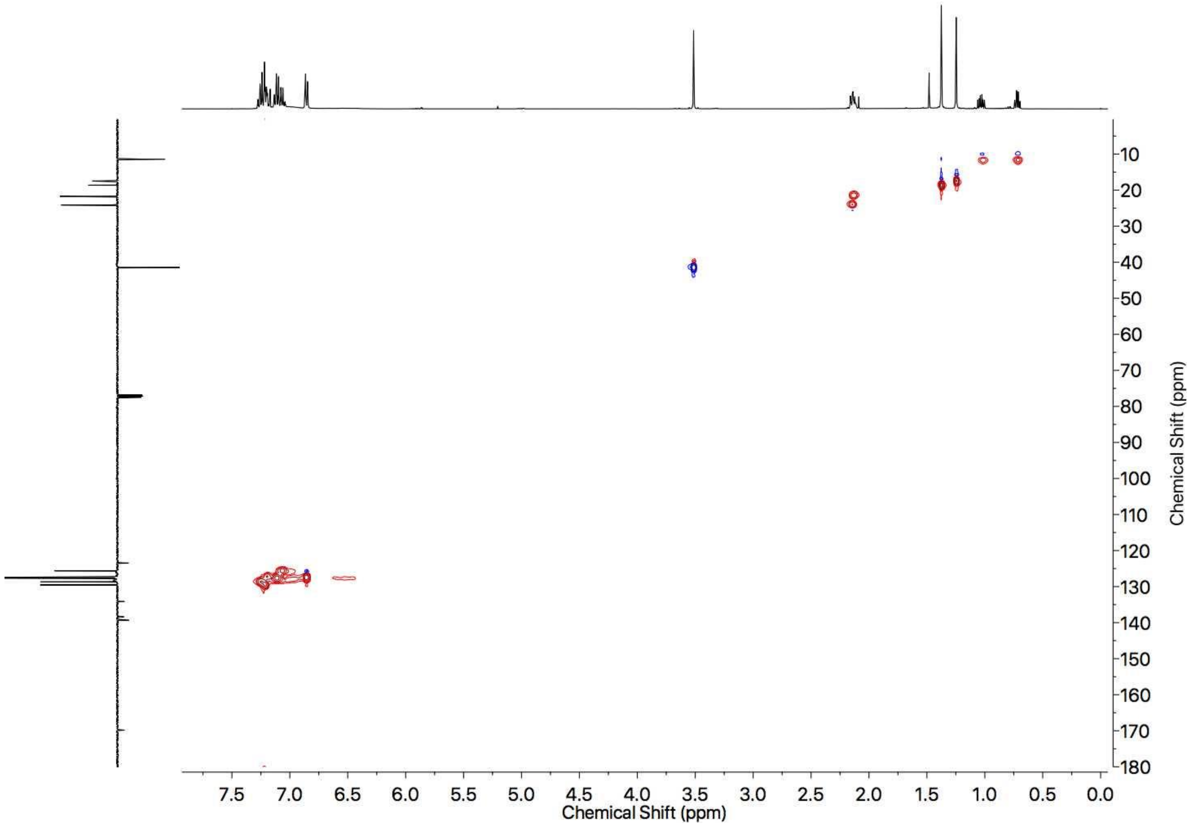

Figure S115: HSQC NMR ($CDCl_3$) of *cis*-**14**.

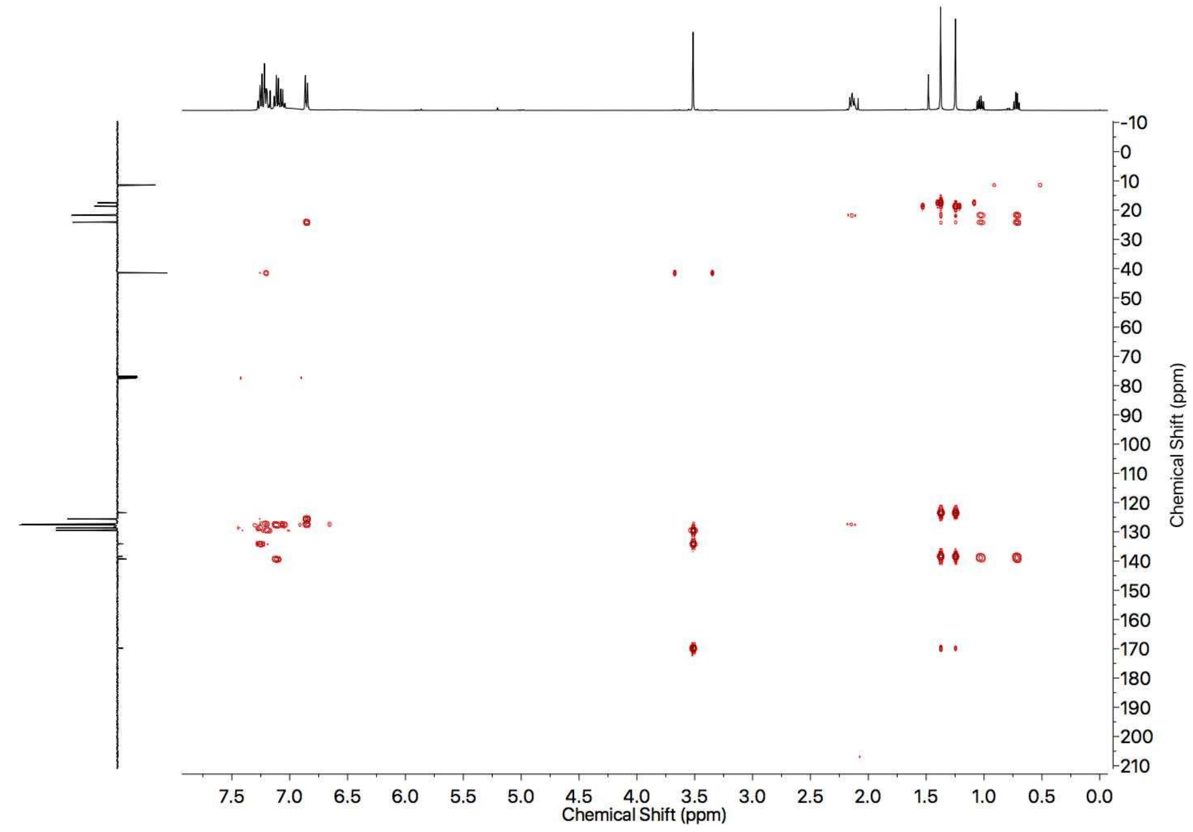

Figure S116: HMBC NMR ($CDCl_3$) of *cis*-**14**.

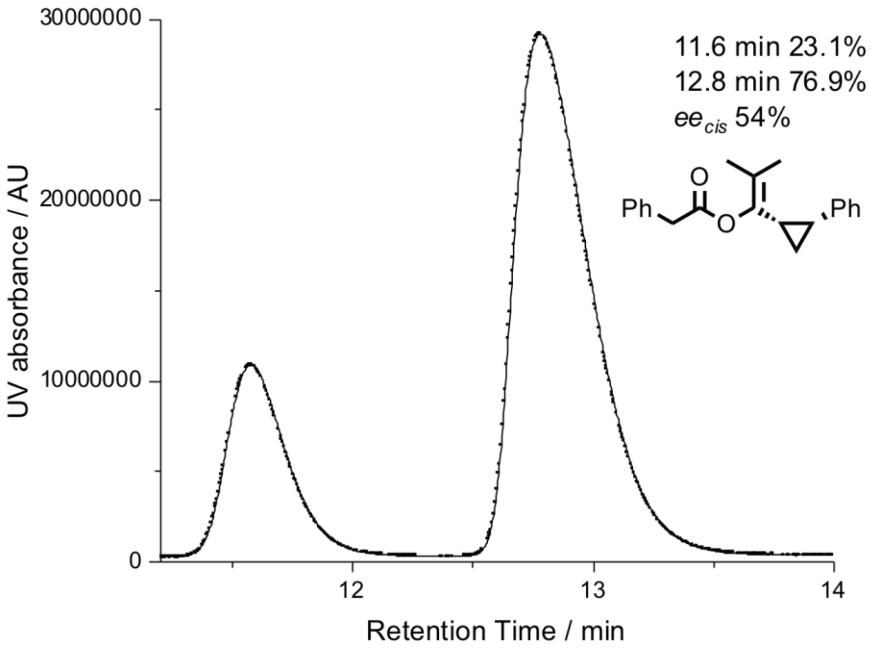

Figure S117: Chiral Stationary Phase HPLC ((*S*,*S*)Whelk, *n*-hexane-isopropanol 99.5 : 0.5, 303 K, load petrol, flowrate 0.50 mLmin^{-1}) of 77 : 23 *er cis*-**14**. Retention times (min): 11.6, 12.8. The absolute stereochemistry of the products was not determined. The (1*S*,2*R*)-**14** isomer is shown for illustrative purposes only.

Cyclopropanes **15**

**Catalyst**	**Yield / %**	***dr***	***er*cis**	***er*trans**
$(Ph_3P)AuCl$[a]	79	82 : 18	1 : 1	1 : 1
[Au((*R*mp)-**6**)(Cl)][b]	45	96 : 4	73 : 27	-

Table S10. Summary of reactions leading to cyclopropanes **15**.

Cis-**15**

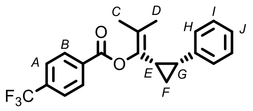

δ_H ($CDCl_3$, 400 MHz) 7.88 (2H, d, *J* = 8.2, ***H*B**), 7.66 (2H, d, *J* = 8.4, ***H*A**), 7.25 (2H, tt, *J* = 6.5, 2.0, ***H*I**), 7.20 (1H, tt, *J* = 7.1, 1.5, ***H*J**), 7.09 (2H, dd, *J* = 8.4, 1.7, ***H*H**), 2.47-2.28 (2H, m, ***H*E**, ***H*F**), 1.70 (3H, s, ***H*D**), 1.48 (3H, s, ***H*C**), 1.33 (1H, ddd, *J* = 14.3, 8.8, 5.4, ***H*G**), 1.09 (1H, q, *J* = 6.0, ***H*G′**).

δ_C ($CDCl_3$, 101 MHz) 163.5, 139.4, 138.9, 134.7 (q, *J* = 32.4), 134.5, 130.4, 127.9, 127.8, 125.9, 125.5 (q, *J* = 3.7), 124.0, 122.4, 23.7, 21.2, 18.9, 17.8, 12.2.

δ_F ($CDCl_3$, 376 MHz) -63.4.

$\delta_{F\{H\}}$ ($CDCl_3$, 376 MHz) -63.4.

HR-EI-MS m/z 360.1325 [$M^{+\cdot}$] (calc. m/z for $C_{21}H_{19}O_2F_3$ 360.1332).

Trans-**15**

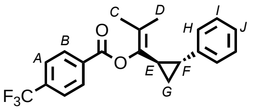

δ_H ($CDCl_3$, 400 MHz) 8.24 (2H, d, *J* = 8.2, ***H*B**), 7.78 (2H, d, *J* = 8.2, ***H*A**), 7.30-7.23 (2H, m, ***H*I**), 7.17 (1H, tt, *J* = 7.5, 1.3, ***H*J**), 7.09 (2H, dd, *J* = 7.8, 1.5, ***H*H**), 2.13 (1H, ddd, *J* = 9.5, 7.7, 3.2, ***H*E**), 2.03 (1H, d, *J* = 1.2, ***H*F**), 1.87 (3H, s, ***H*D**), 1.62 (3H, s, ***H*C**), 1.20-1.15 (2H, m, ***H*G**, ***H*G′**).

δ_C ($CDCl_3$, 101 MHz) 167.6, 164.5, 141.1, 135.5, 134.5, 130.5, 128.5, 126.1, 126.0, 125.8, 122.7, 121.6, 24.0, 23.3, 19.0, 18.3, 14.9.

δ_F ($CDCl_3$, 376 MHz) -63.4.

$\delta_{F\{H\}}$ ($CDCl_3$, 376 MHz) -63.4.

HR-EI-MS m/z 360.1326 [$M^{+\cdot}$] (calc. m/z for $C_{21}H_{19}O_2F_3$ 360.1332).

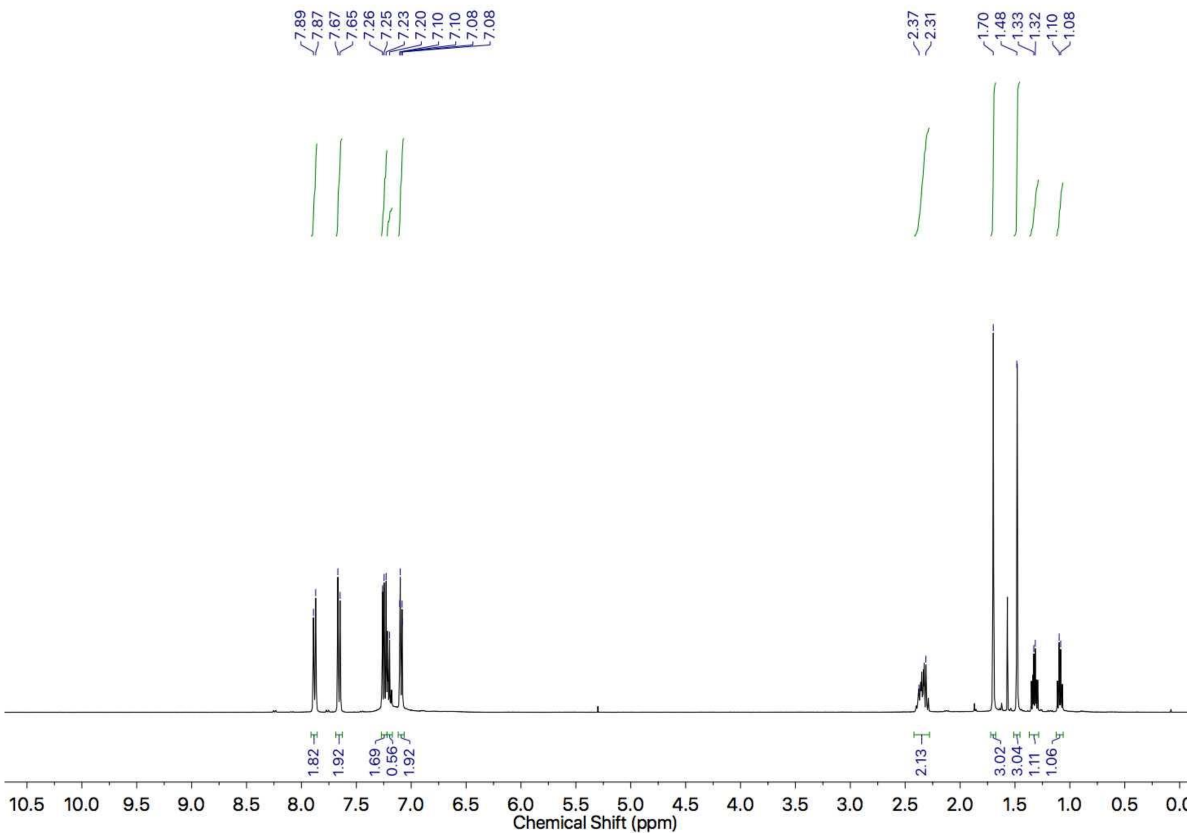

Figure S118: ^{1}H NMR (400 MHz, $CDCl_3$) of *cis*-**15**.

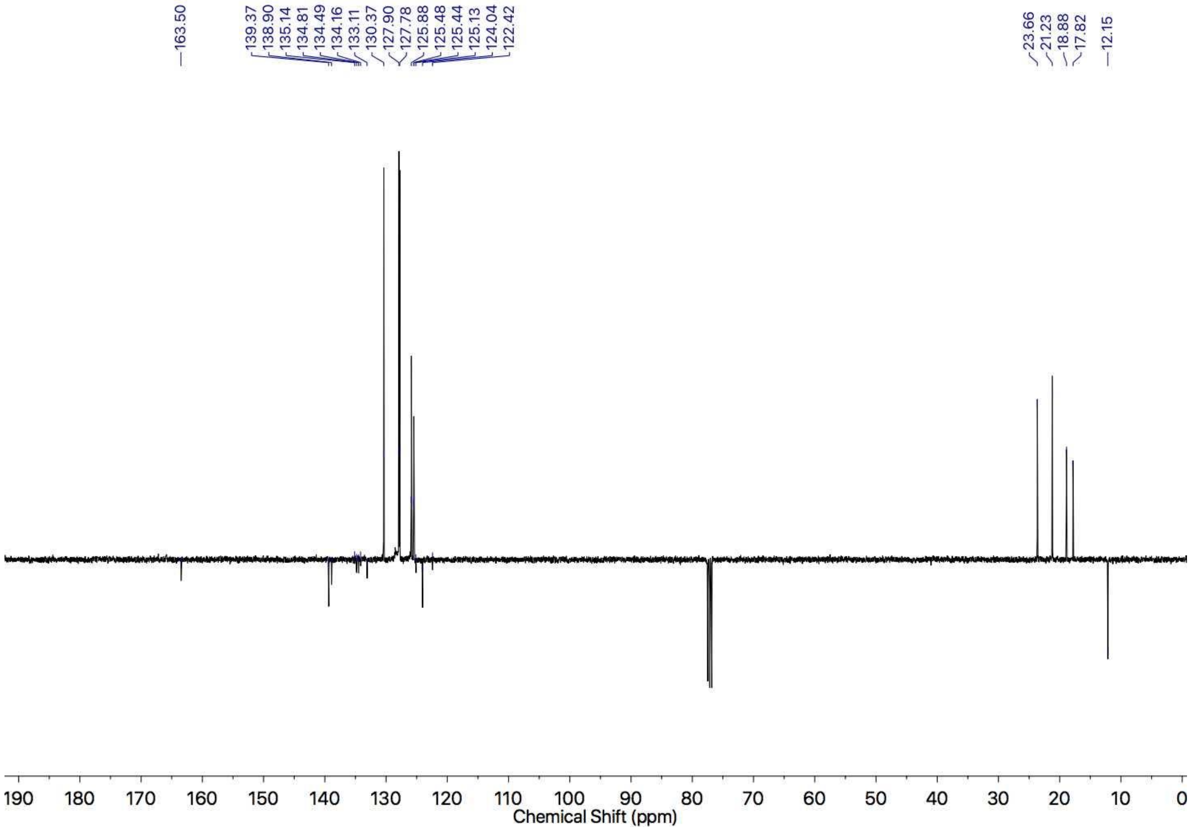

Figure S119: JMOD NMR (101 MHz, $CDCl_3$) of *cis*-**15**.

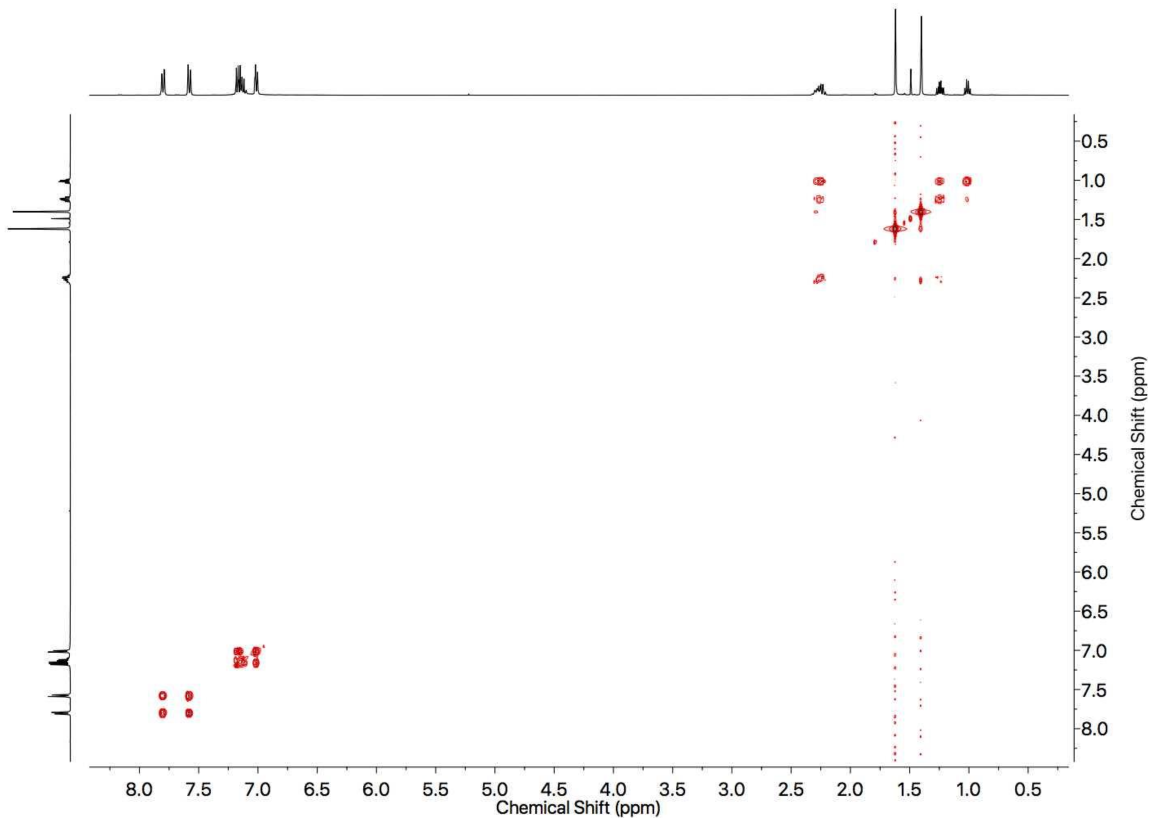

Figure S120: COSY NMR ($CDCl_3$) of *cis*-**15**.

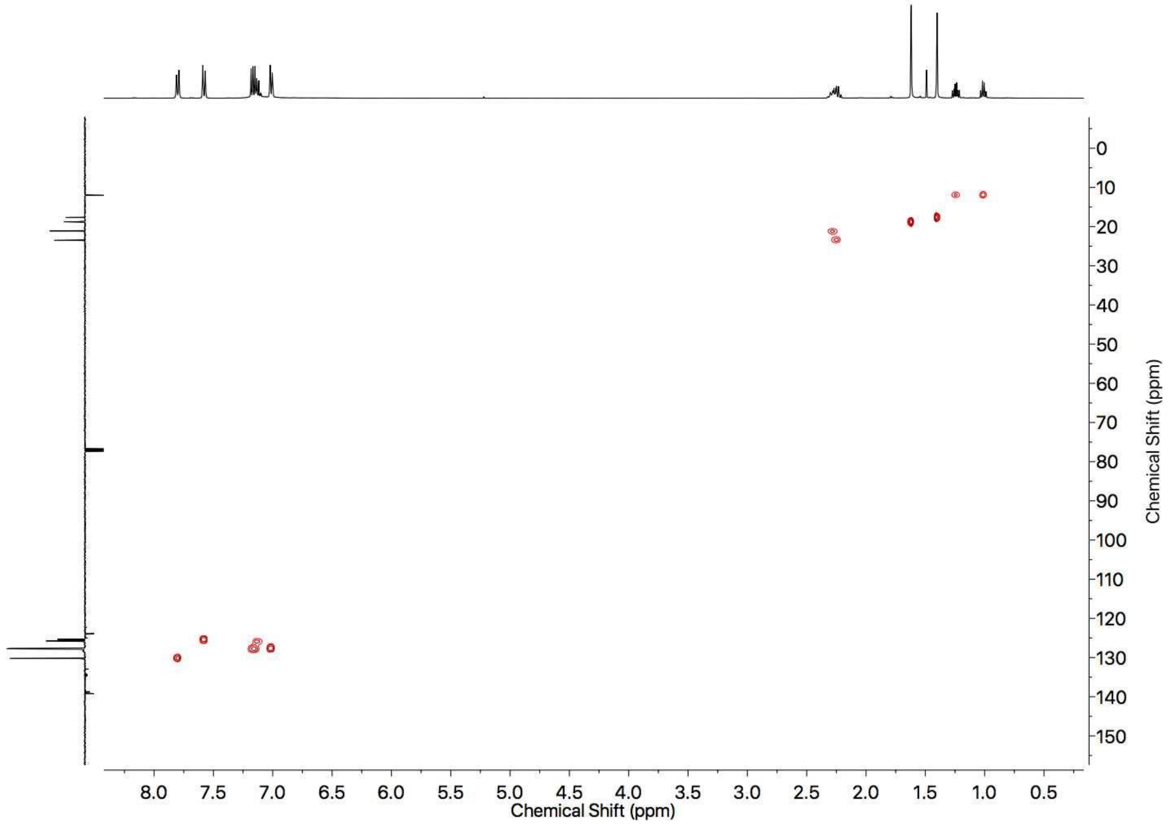

Figure S121: HSQC NMR ($CDCl_3$) of *cis*-**15**.

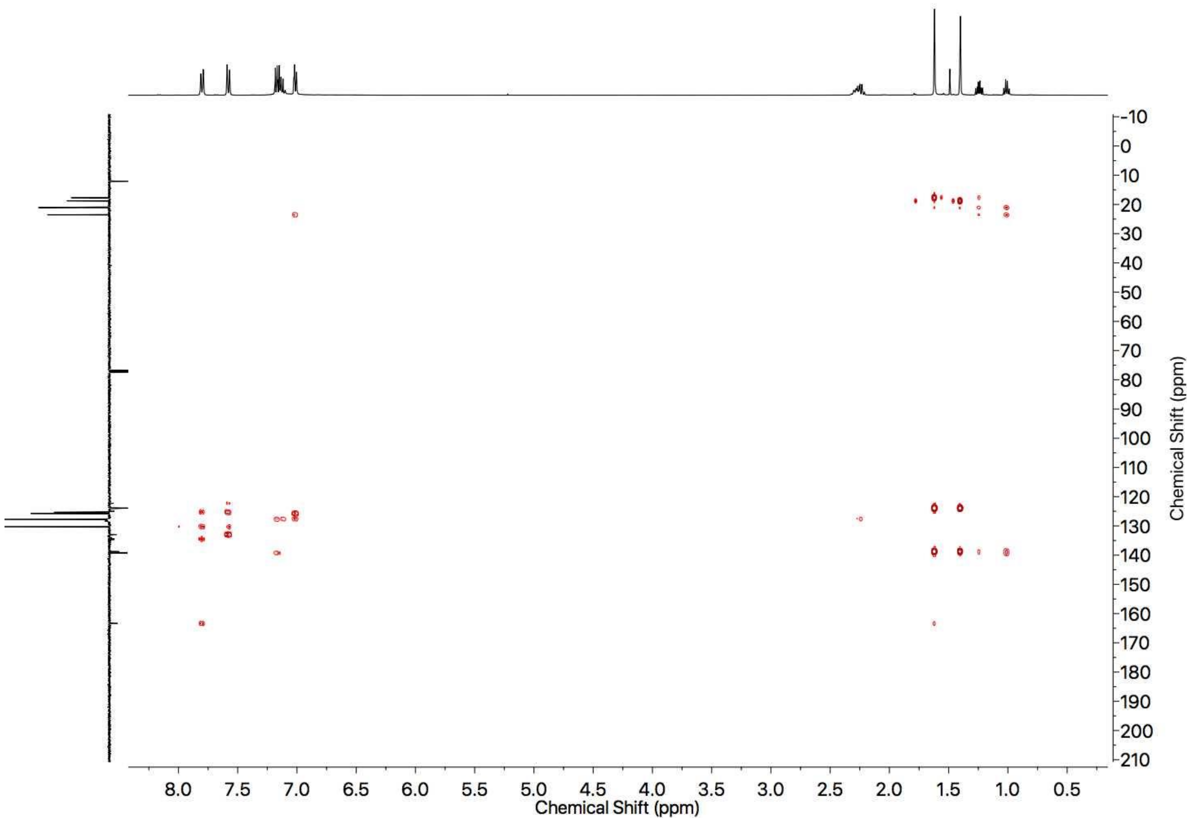

Figure S122: HMBC NMR ($CDCl_3$) of *cis*-**15**.

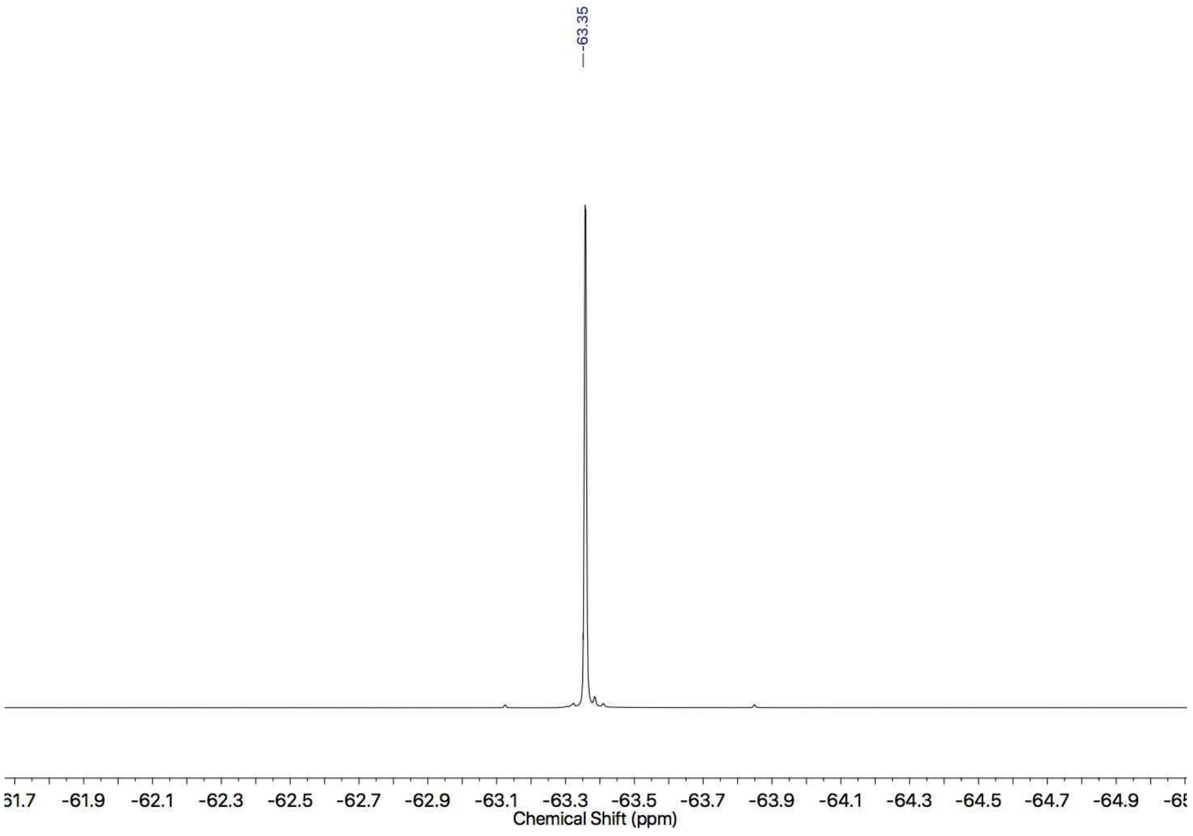

Figure S123: ^{19}F NMR (376 MHz, $CDCl_3$) of *cis*-**15**.

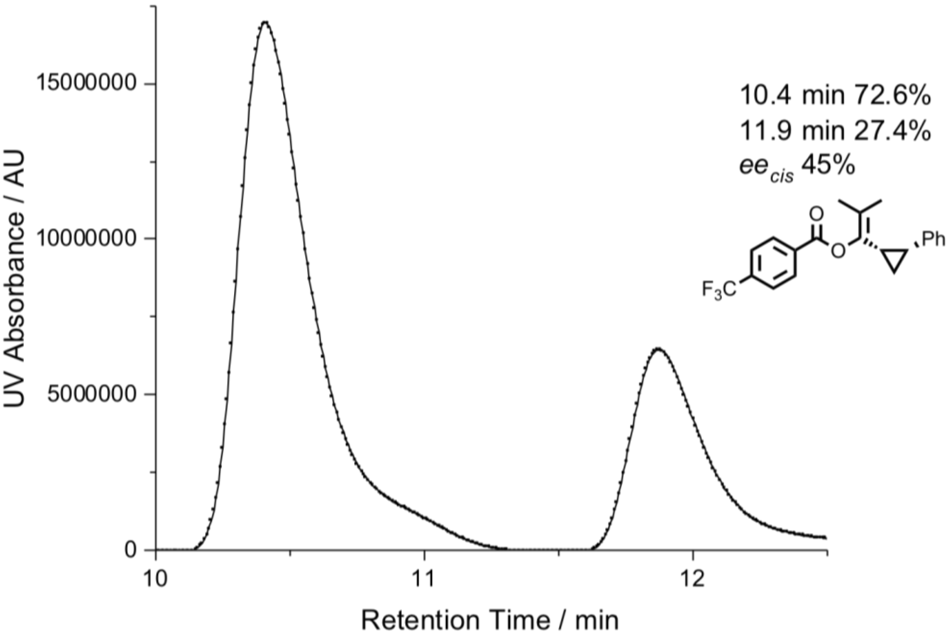

Figure S124: Chiral Stationary Phase HPLC (RegisCell, *n*-hexane-isopropanol 99.9 : 0.1, 303 K, load Et_2O, flowrate 0.75 $mLmin^{-1}$) of 73 : 27 *er cis*-**15**. Retention times (min): 10.4, 11.9. The absolute stereochemistry of the major product was determined to be (1*S*,2*R*)-**15** by reduction with $LiAlH_4$ and comparison with the product of the same reaction with cyclopropane **9** (see **Figure S168**).

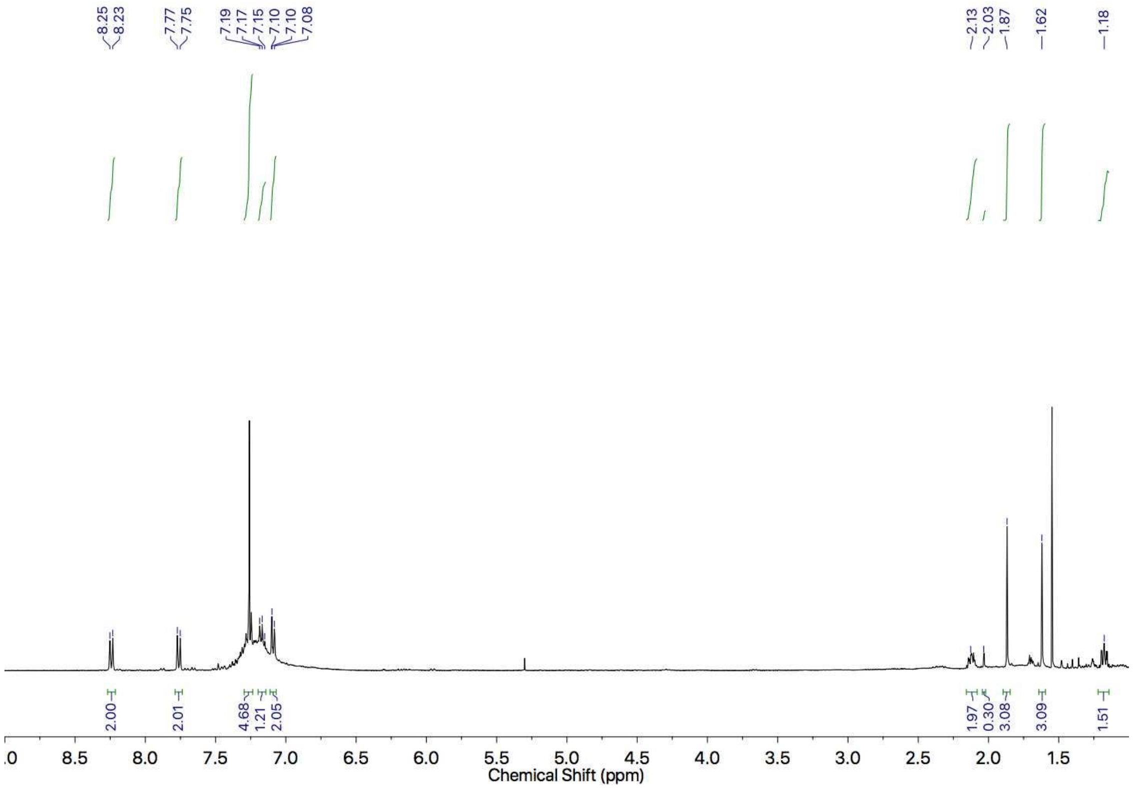

Figure S125: ^{1}H NMR (400 MHz, $CDCl_3$) of *trans*-**15**.

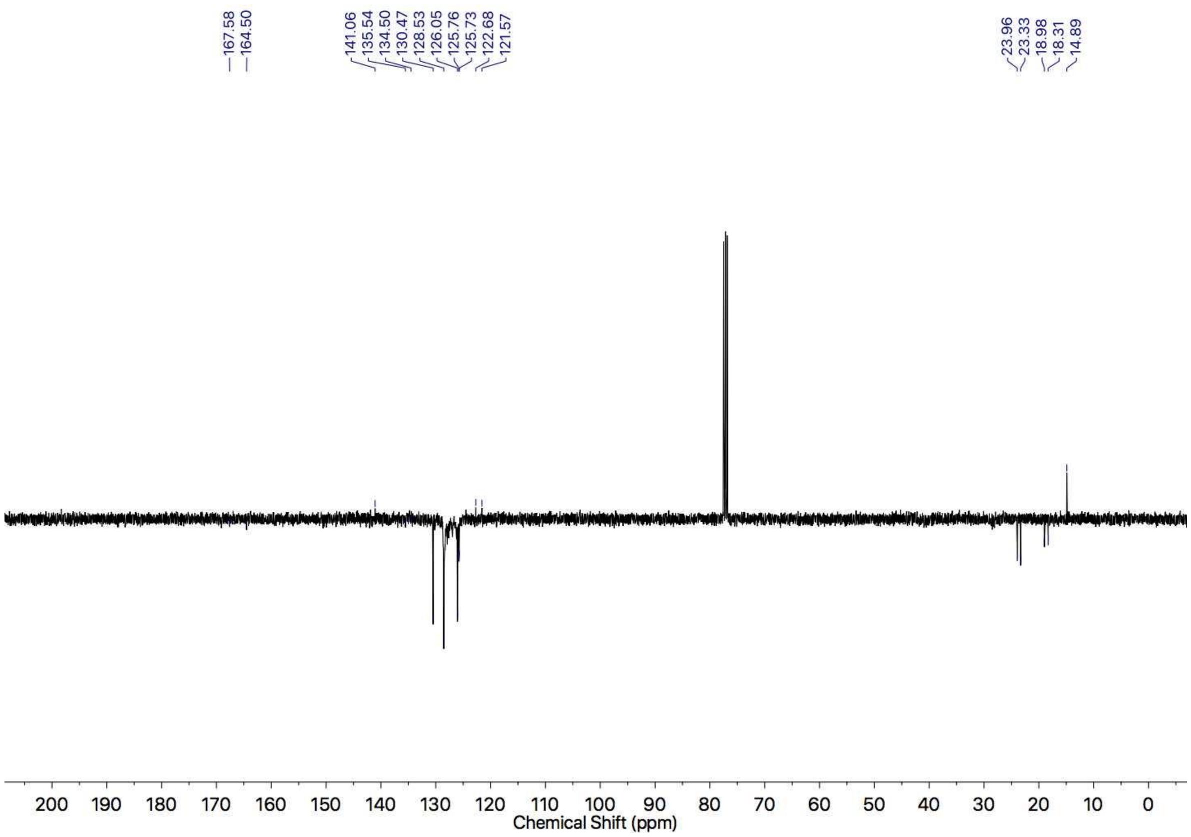

Figure S126: JMOD NMR (101 MHz, $CDCl_3$) of *trans*-**15**.

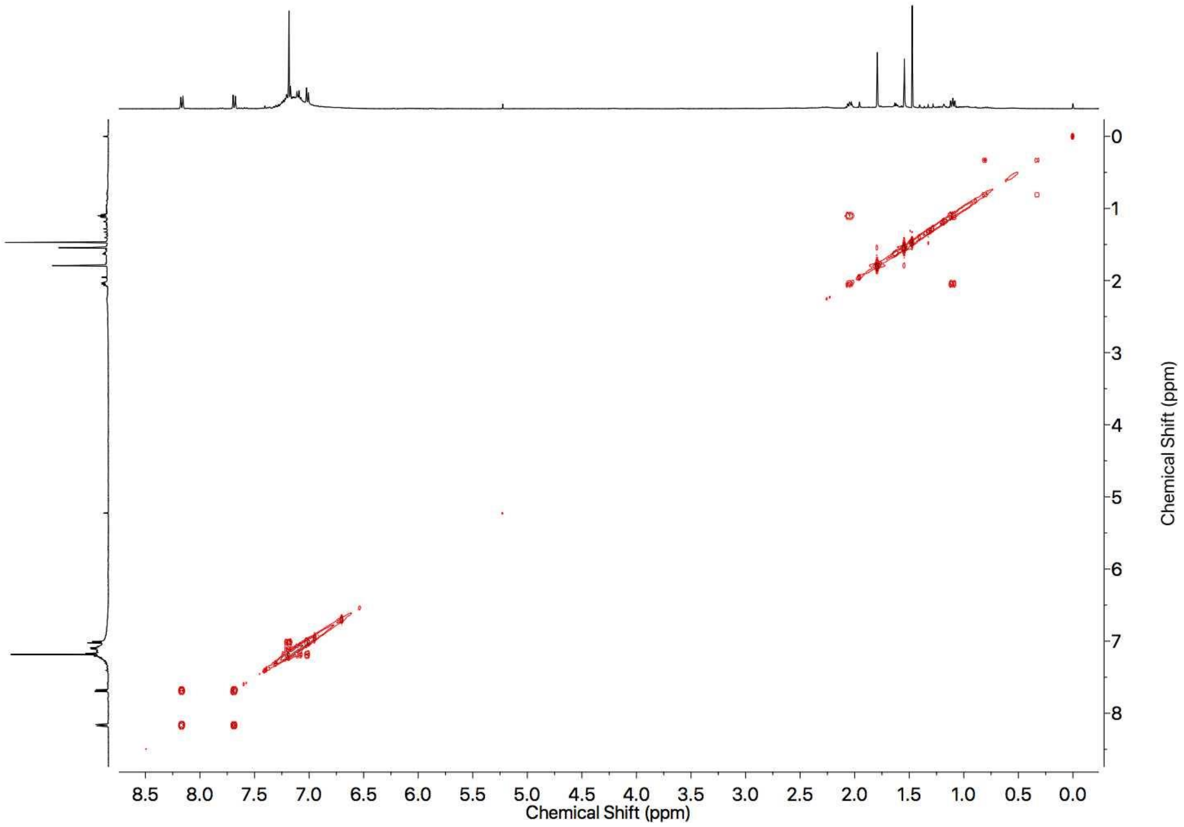

Figure S127: COSY NMR ($CDCl_3$) of *trans*-**15**.

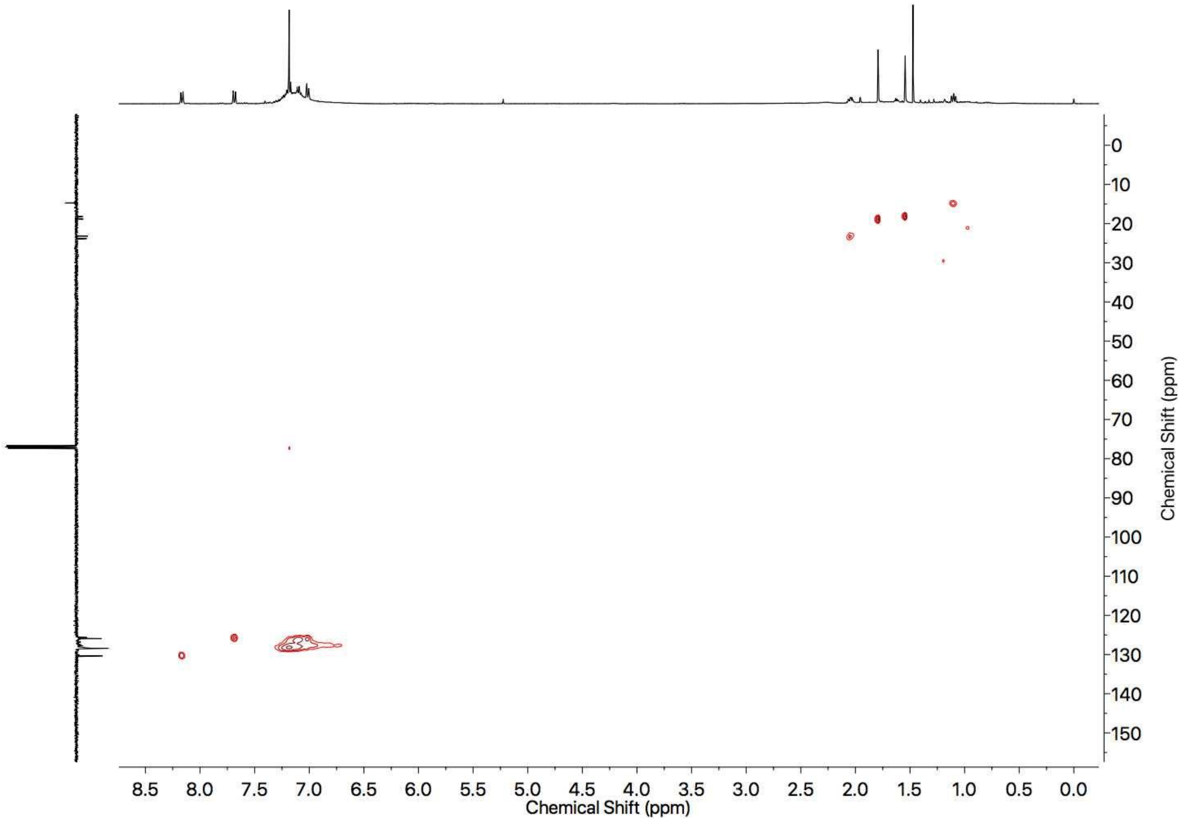

Figure S128: HSQC NMR ($CDCl_3$) of *trans*-**15**.

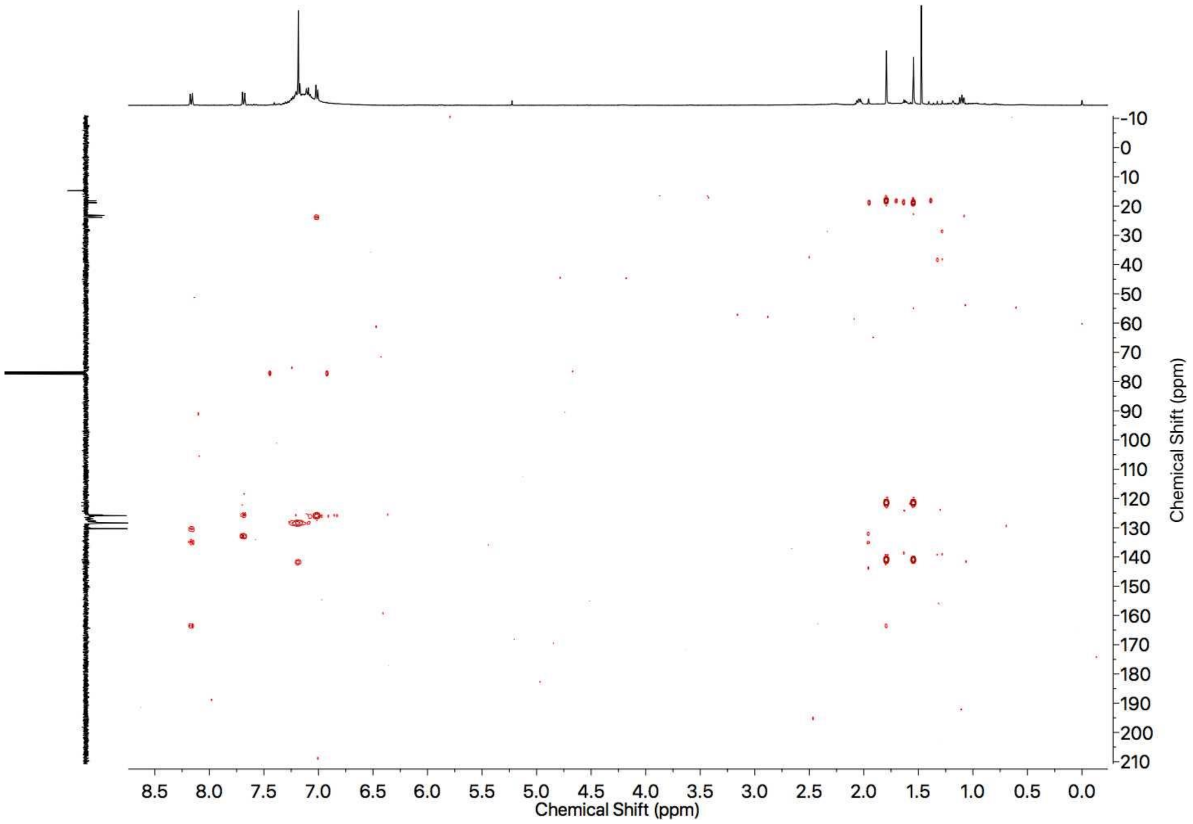

Figure S129: HMBC NMR ($CDCl_3$) of *trans*-**15**.

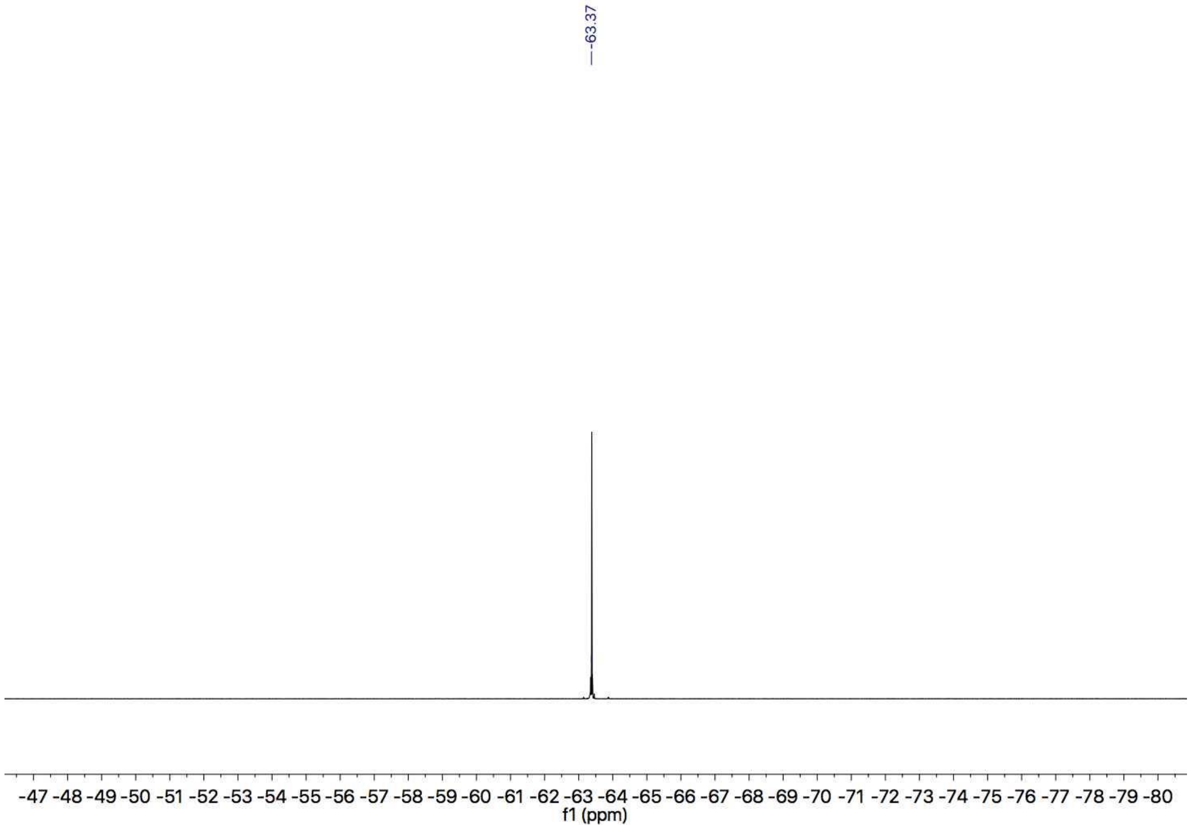

Figure S130: ^{19}F NMR (376 MHz, $CDCl_3$) of *trans*-**15**.

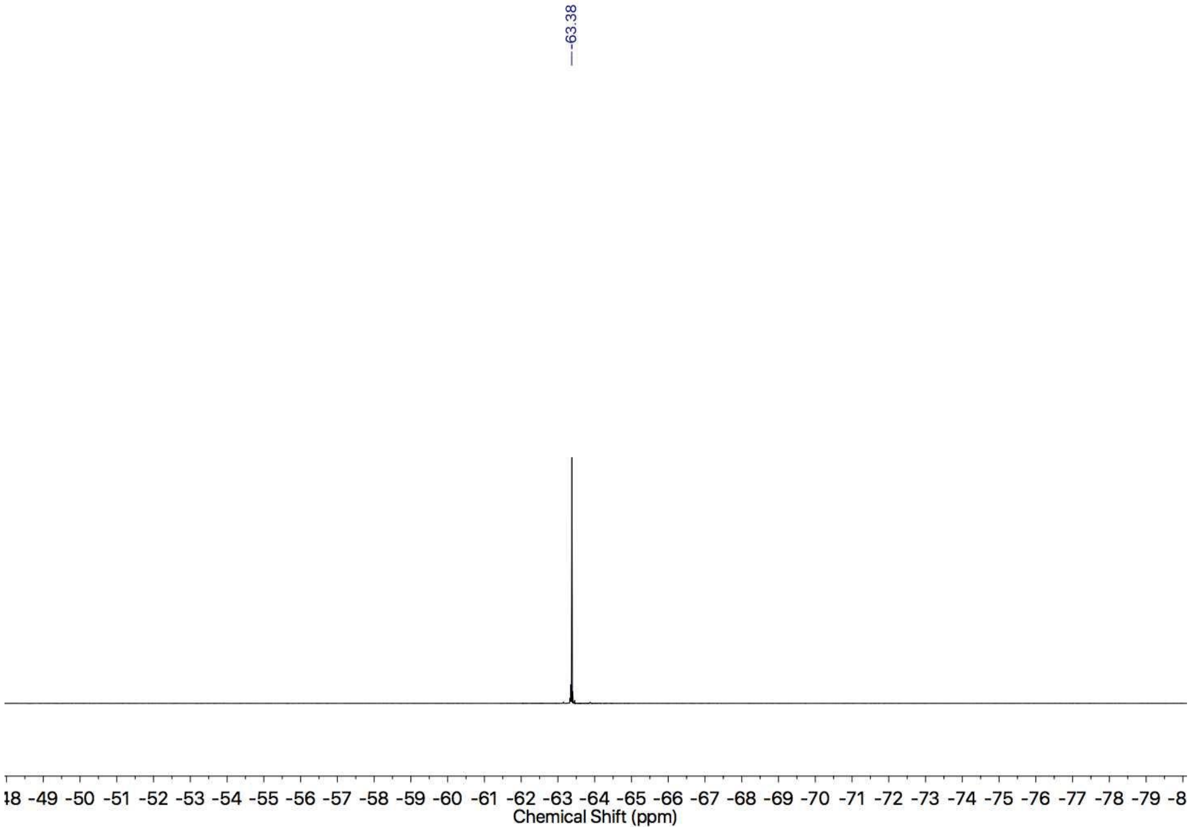

Figure S131: $^{19}F\{^1H\}$ NMR (376 MHz, $CDCl_3$) of *trans*-**15**.

Cyclopropanes **16**

**Catalyst**	**Yield / %**	***dr***	***er*****cis**	***er**trans*
$(Ph_3P)AuCl$[a]	62	90 : 10	1 : 1	1 : 1
[Au((*R*mp)-**6**)(Cl)][b]	48	94 : 6	71 : 29	73 : 27

Table S11. Summary of reactions leading to cyclopropanes **16**.

Cis-**16**

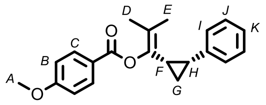

δ_H ($CDCl_3$, 400 MHz) 7.81 (2H, dt, *J* = 9.0, 2.4, ***H***C), 7.24 (2H, br. dd, *J* = 7.7, 7.2, ***H***J), 7.18 (1H, tt, *J* = 7.2, 1.5, ***H***K), 7.10 (2H, dd, *J* = 7.6, 1.6, ***H***I), 6.89 (2H, dt, *J* = 9.0, 2.4, ***H***B), 3.87 (3H, s, ***H***A), 2.40-2.27 (2H, m, ***H***F, ***H***G), 1.63 (3H, s, ***H***E), 1.46 (3H, s, ***H***D), 1.26 (1H, ddd, *J* = 14.2, 8.8, 5.6, ***H***H), 1.09 (1H, q, *J* = 5.9, ***H***H′).

δ_C ($CDCl_3$, 101 MHz) 164.6, 163.6, 139.6, 138.6, 132.1, 127.8, 127.8, 125.7, 123.4, 122.3, 113.7, 55.6, 23.9, 21.6, 18.8, 17.8, 11.8.

HR-EI-MS m/z 322.1556 [$M^{+\cdot}$] (calc. m/z for $C_{21}H_{22}O_3$ 322.1563).

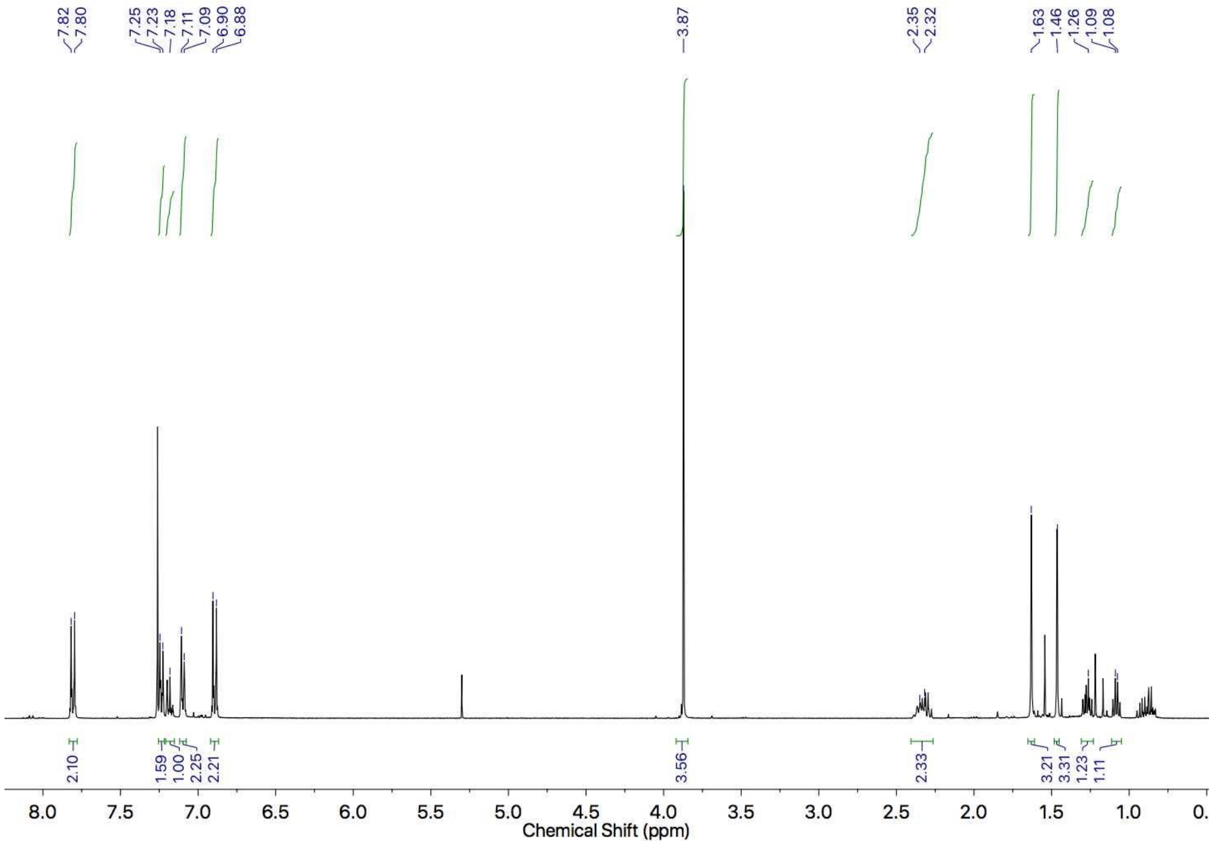

Figure S132: ^{1}H NMR (400 MHz, $CDCl_3$) of *cis*-**16**.

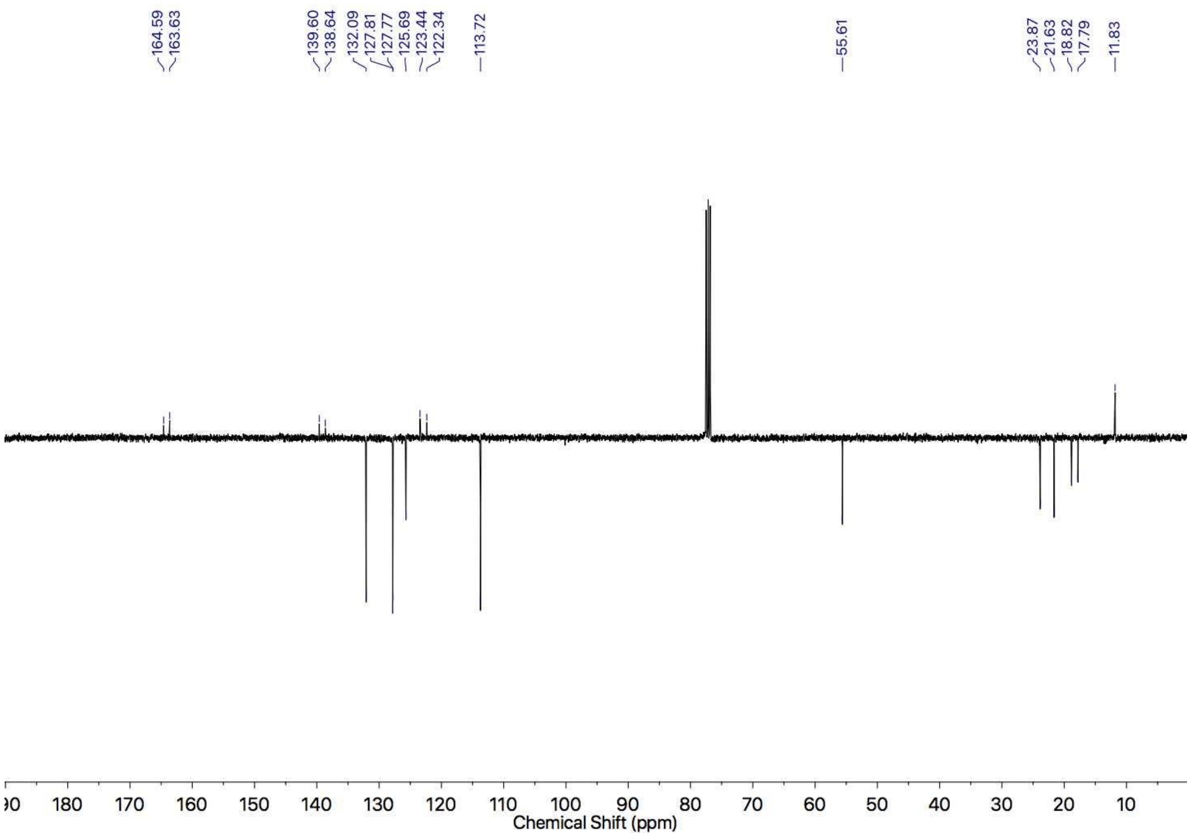

Figure S133: JMOD NMR (101 MHz, $CDCl_3$) of *cis*-**16**.

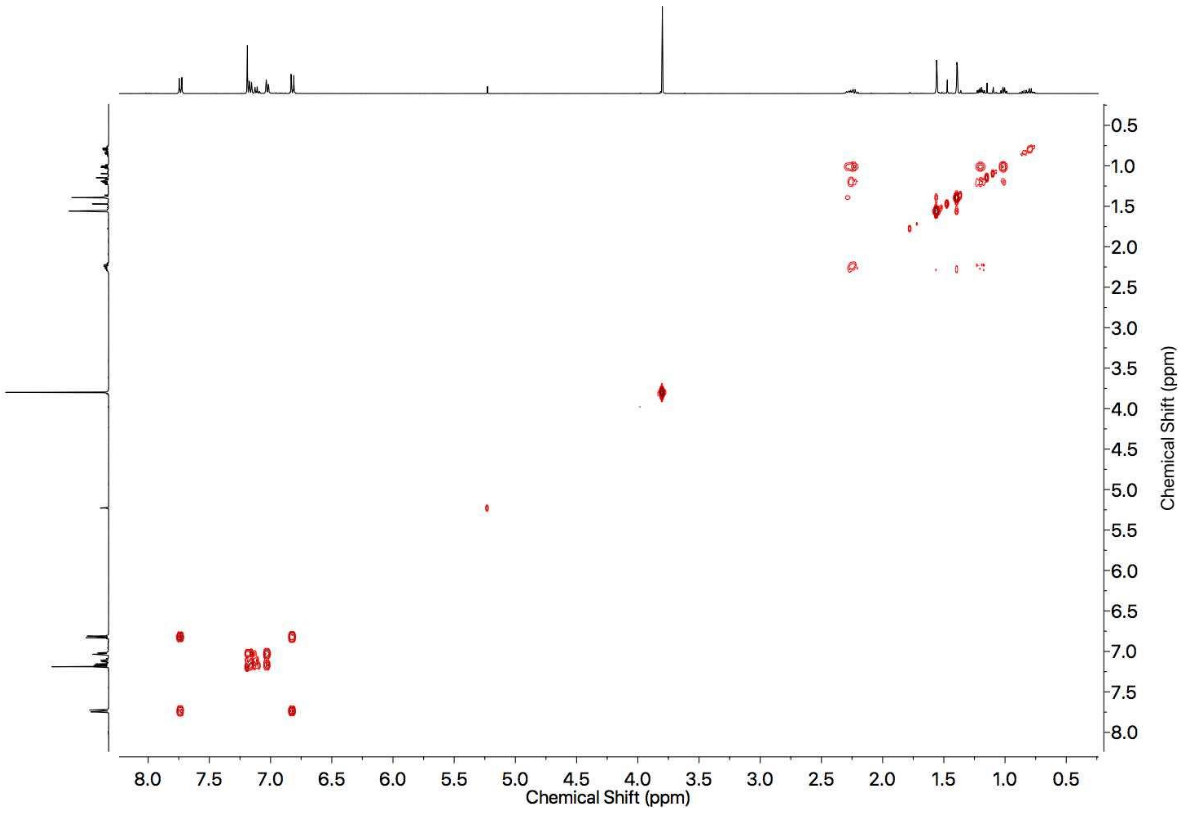

Figure S134: COSY NMR ($CDCl_3$) of *cis*-**16**.

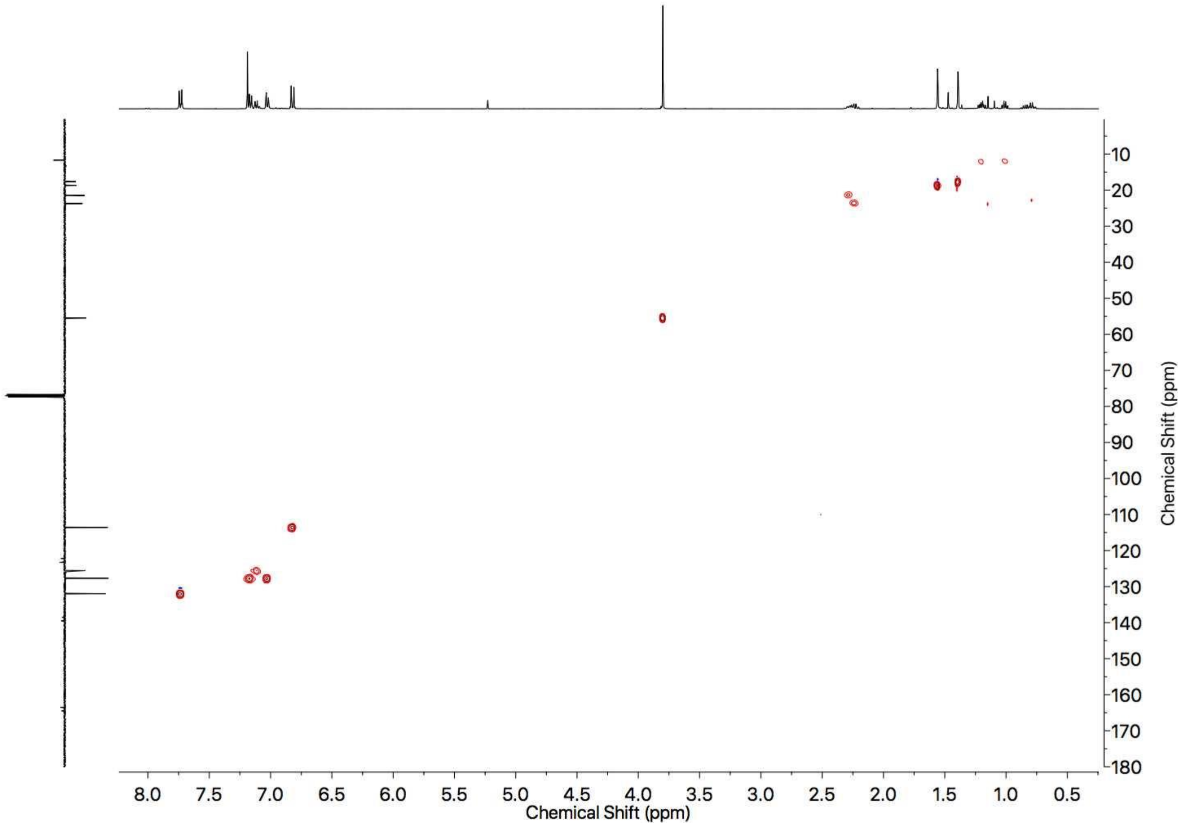

Figure S135: HSQC NMR ($CDCl_3$) of *cis*-**16**.

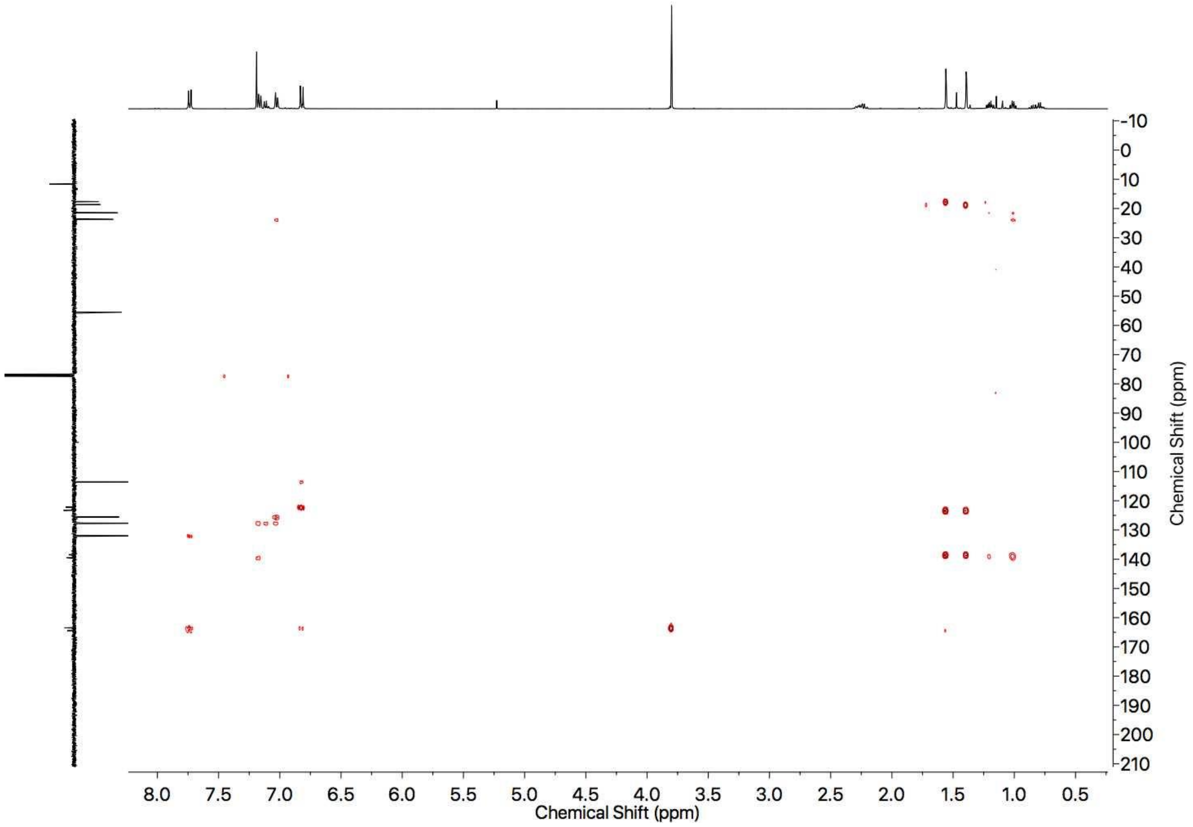

Figure S136: HMBC NMR ($CDCl_3$) of *cis*-**16**.

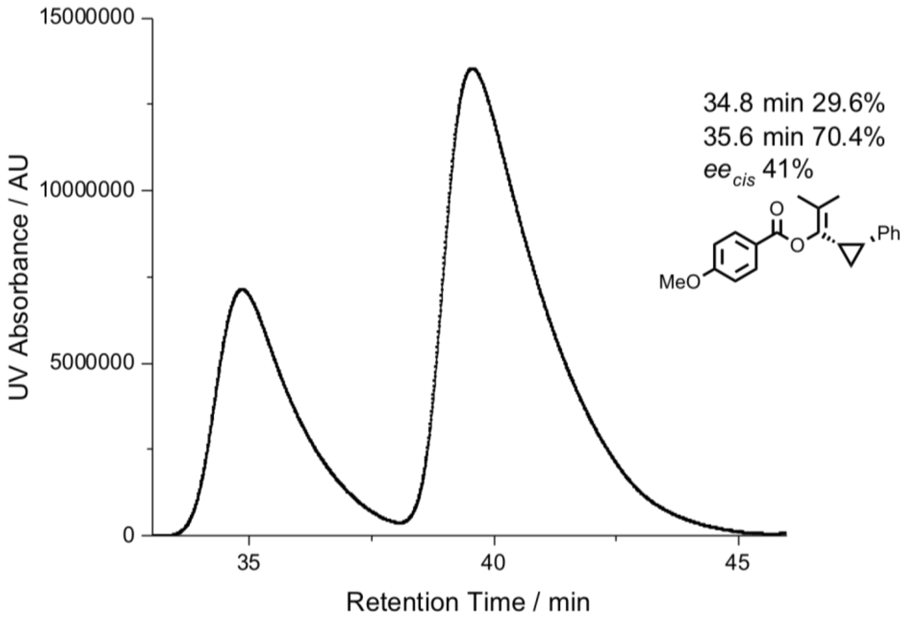

Figure S137: Chiral Stationary Phase HPLC (RegisPack, *n*-hexane-isopropanol 99 : 1, 303 K, load Et_2O, flowrate 0.25 mLmin^{-1}) of 70 : 30 *er cis*-**16**. Retention times (min): 34.8, 35.6. The absolute stereochemistry of the major product was determined to be (1*S*,2*R*)-**16** by reduction with $LiAlH_4$ and comparison with the product of the same reaction with cyclopropane **9** (see **Figure S168**).

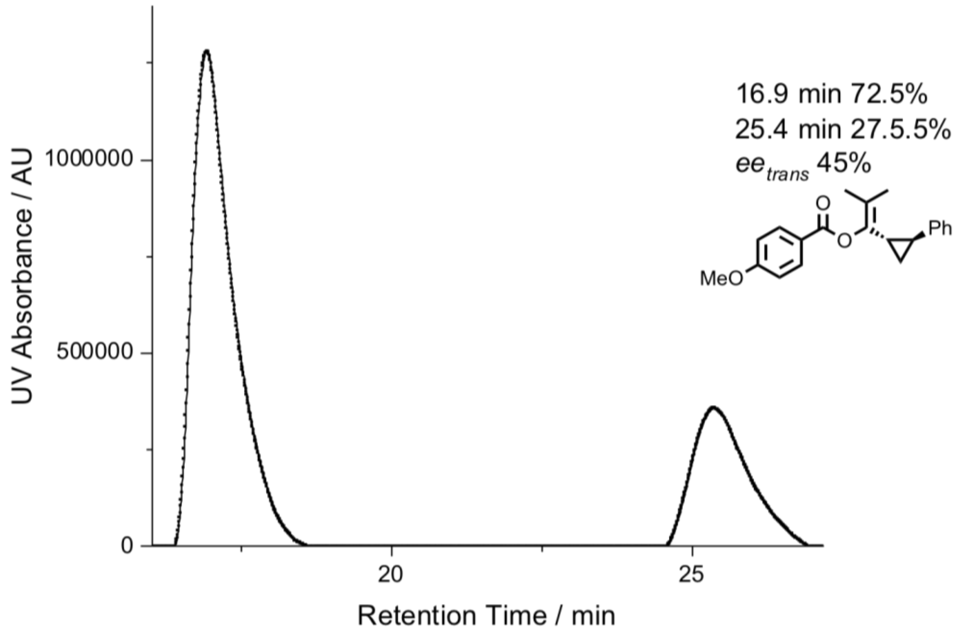

Figure S138: Chiral Stationary Phase HPLC (RegisPack, *n*-hexane-isopropanol 99 : 1, 303 K, load Et_2O, flowrate 0.25 mLmin^{-1}) of 72.5 : 27.5 *er trans*-**16**. Retention times (min): 16.9, 25.4. The absolute stereochemistry of the products was not determined. The (1*S*,2*S*)-**16** isomer is shown for illustrative purposes only.

Cyclopropanes **17**

**Catalyst**	**Yield / %**	***dr***	***er***	

**Catalyst**	**Yield / %**	***dr***	$\boldsymbol{er_{cis}}$	$\boldsymbol{er_{trans}}$
$(Ph_3P)AuCl$	87	95 : 5	1 : 1	1 : 1
[Au((*R*$_{mp}$)-**6**)(Cl)]	79	95 : 5	87 : 13[c]	65 : 35

Table S12. Summary of reactions leading to cyclopropanes **17**.

cis-**17**

Colourless oil (*dr cis-trans* 95 : 5, NMR yield 71 mg, 0.202 mmol, 87%)

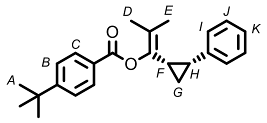

δ_H ($CDCl_3$, 400 MHz) 7.84 (2H, dt, *J* = 9.2, 1.8, **H_C**), 7.45 (2H, dt, *J* = 9.1, 1.8, **H_B**), 7.26 (2H, tt, *J* = 7.1, 1.5, **H_J**), 7.19 (1H, tt, *J* = 7.2, 1.3, **H_K**), 7.12 (2H, dd, *J* = 7.5, 1.7, **H_I**), 2.42-2.28 (2H, m, **H_F**, **H_G**), 1.62 (3H, s, **H_E**), 1.47 (3H, s, **H_D**), 1.36 (9H, s, **H_A**), 1.30-1.24 (1H, m, **H_H**), 1.10 (1H, q, *J* = 5.4, **$H_{H'}$**).

δ_C ($CDCl_3$, 101 MHz) 164.8, 156.9, 139.5, 138.5, 129.9, 127.8, 127.7, 127.2, 125.7, 125.5, 123.5, 35.2, 31.3, 24.0, 21.7, 18.8, 17.7, 11.7.

HR-EI-MS m/z 348.2081 [$M^{+\cdot}$] (calc. m/z for $C_{24}H_{28}O_2$ 348.2084).

Trans-**17**

Colourless oil (*dr cis-trans* 95 : 5, NMR yield 71mg, 0.202 mmol, 87%)

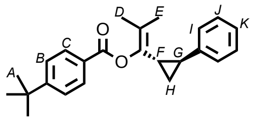

δ_H ($CDCl_3$, 400 MHz) 7.84 (2H, dt, *J* = 8.9, 2.0, **H_C**), 7.50 (2H, dt, *J* = 8.8, 2.0, **H_B**), 7.25 (2H, tt, *J* = 7.4, 1.2, **H_J**), 7.15 (1H, tt, *J* = 7.3, 1.9, **H_K**), 7.09 (2H, dd, *J* = 7.2, 1.5, **H_I**), 2.17-2.06 (2H, m, **H_F**, **H_G**), 1.85 (3H, s, **H_E**) 1.61 (3H, s, **H_D**), 1.36 (9H, s, **H_A**), 1.20 (1H, ddd, *J* = 11.1, 6.0, 5.0, **H_H**), 1.13 (1H, ddd, *J* = 11.1, 5.9, 5.0, **$H_{H'}$**).

δ_C ($CDCl_3$, 101 MHz) 164.9, 157.2, 142.3, 130.0, 128.5, 127.0, 126.1, 125.9, 125.7, 122.8, 120.9, 35.3, 31.3, 23.8, 23.6, 19.0, 18.3, 14.7.

HR-EI-MS m/z 348.2077 [$M^{+\cdot}$] (calc. m/z for $C_{24}H_{28}O_2$ 348.2089).

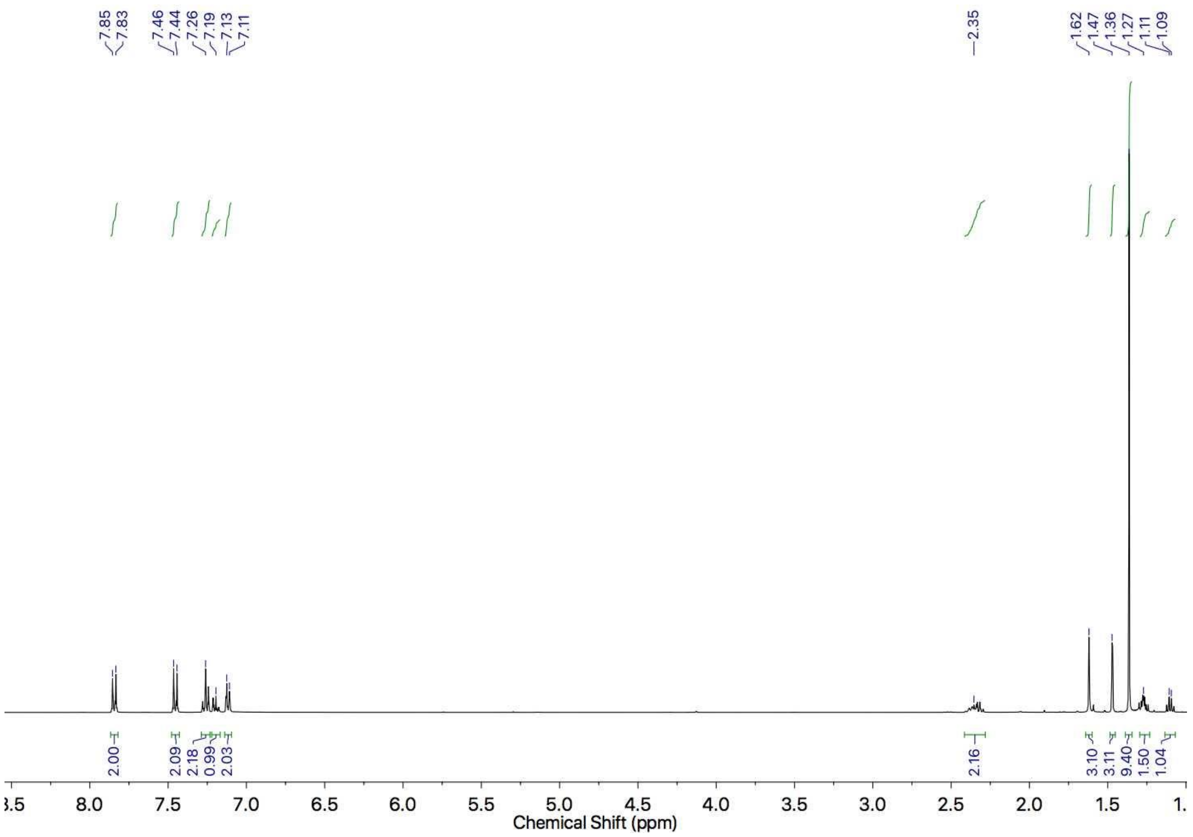

Figure S139: ^{1}H NMR (400 MHz, $CDCl_3$) of *cis*-**17**.

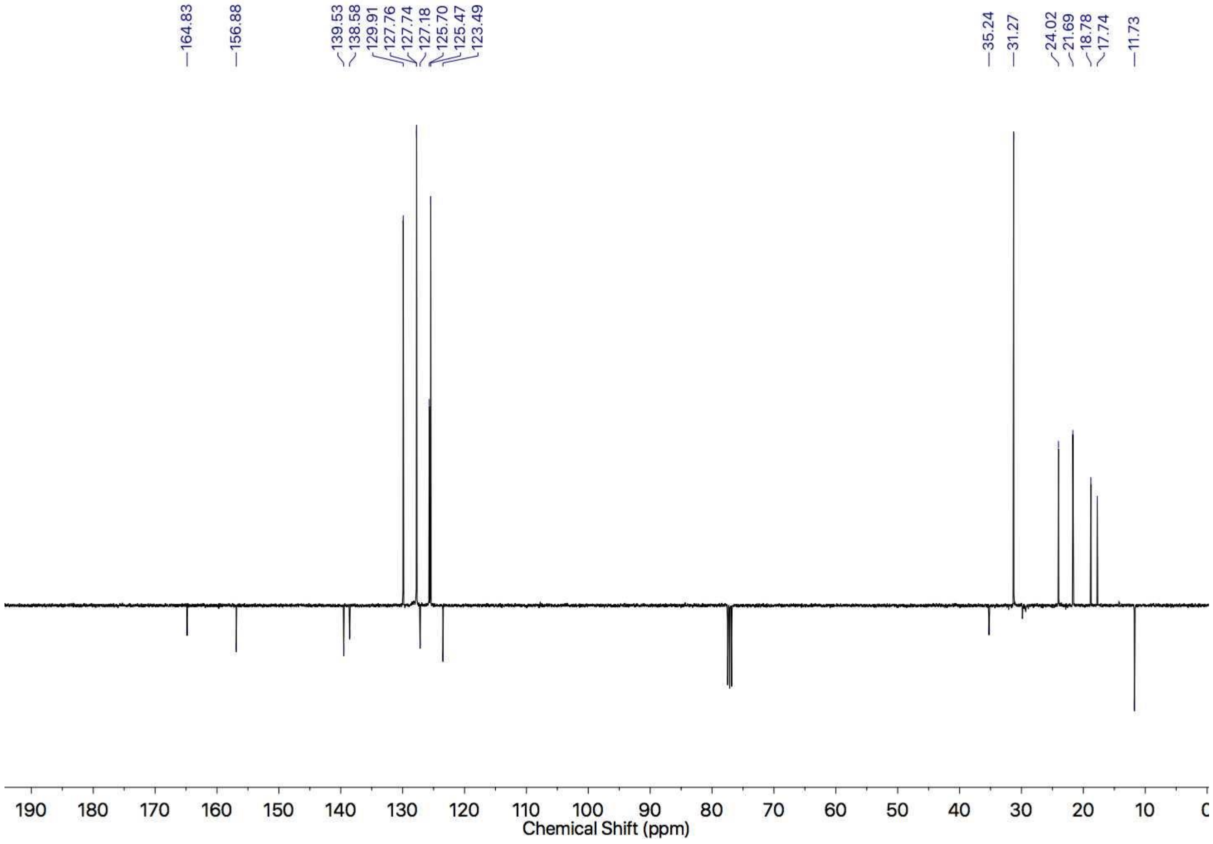

Figure S140: JMOD NMR (101 MHz, $CDCl_3$) of *cis*-**17**.

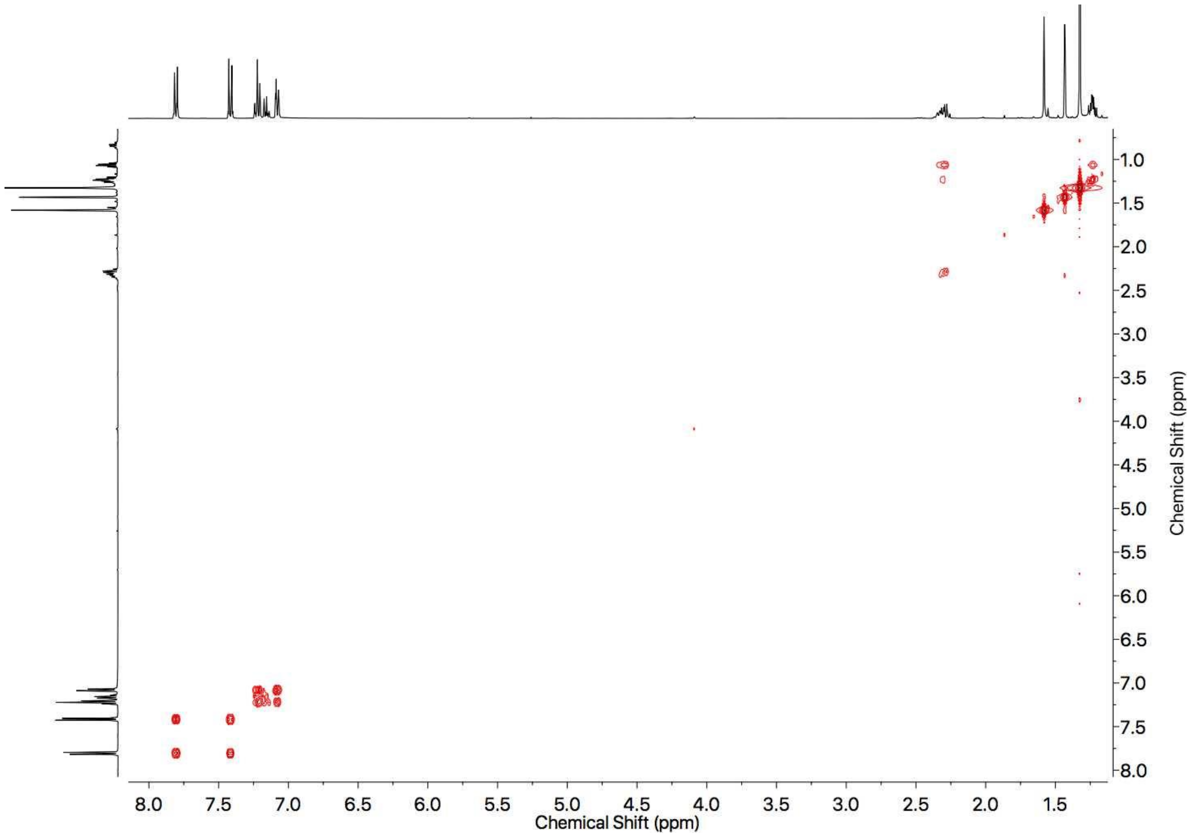

Figure S141: COSY NMR ($CDCl_3$) of *cis*-**17**.

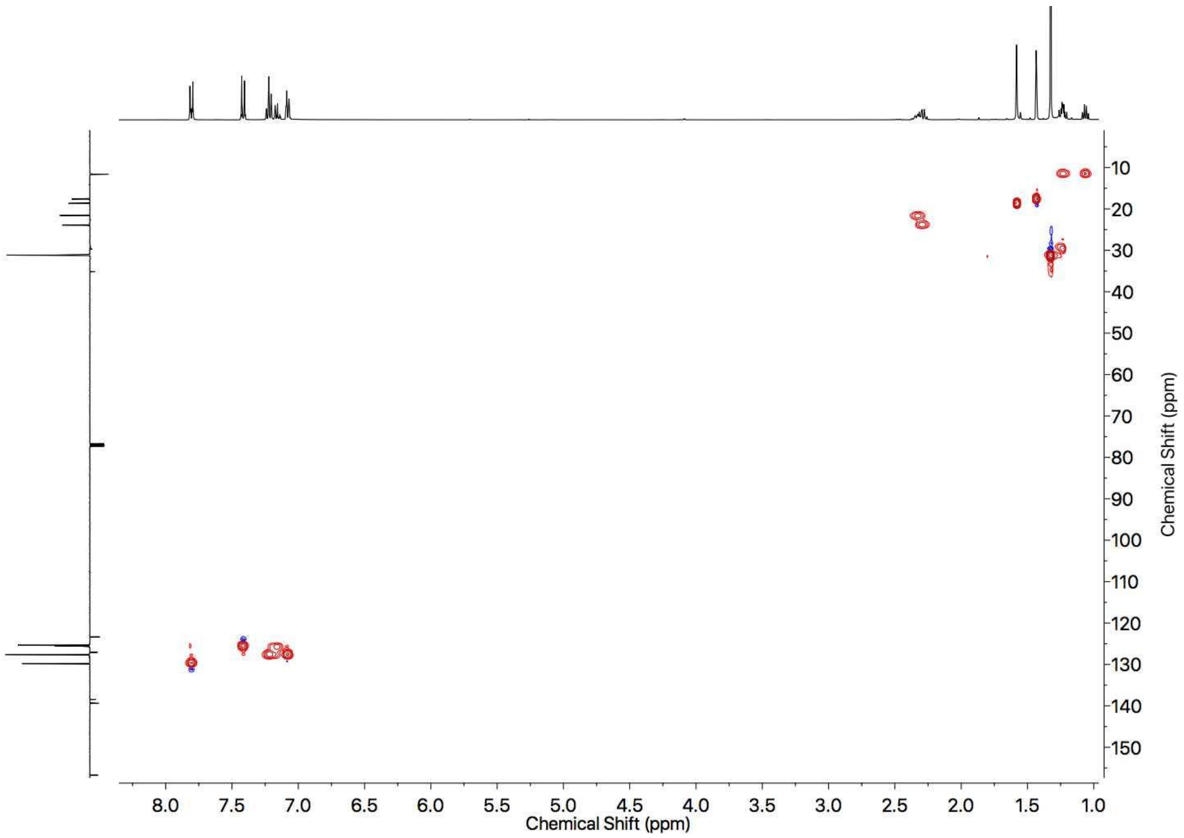

Figure S142: HSQC NMR ($CDCl_3$) of *cis*-**17**.

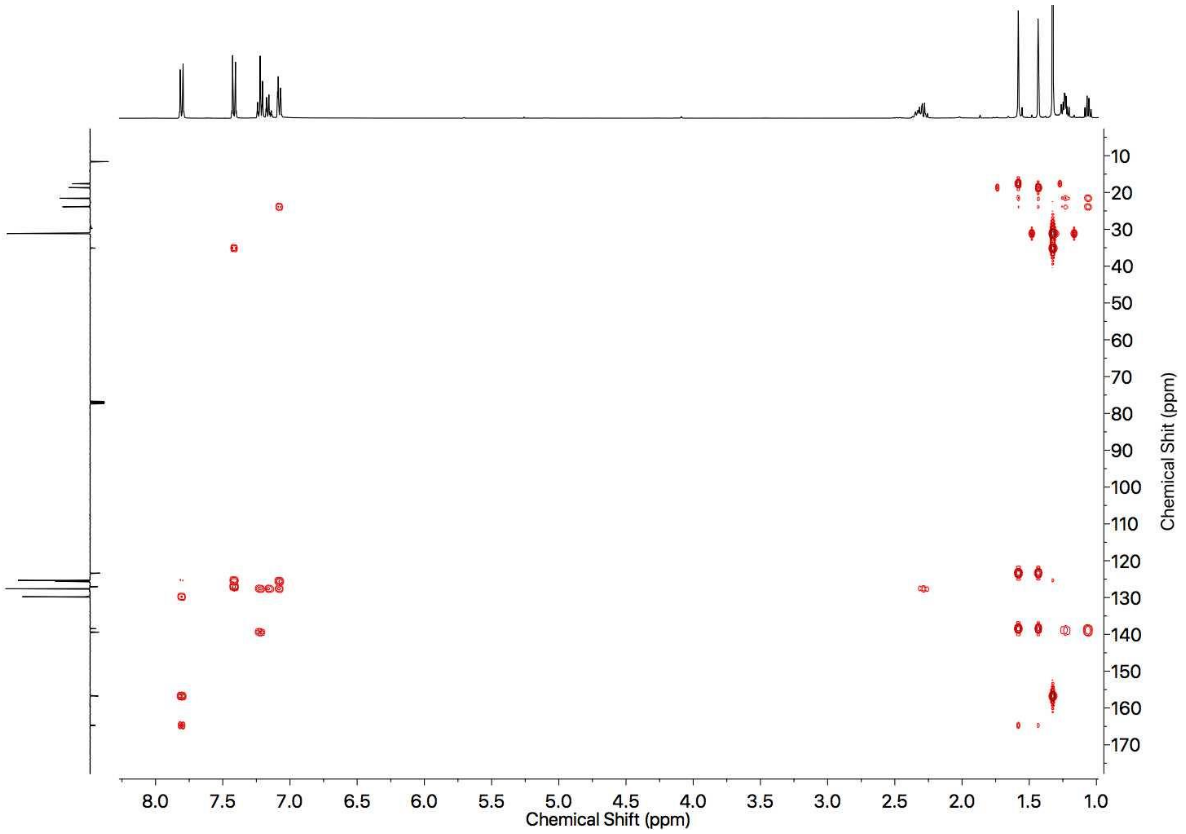

Figure S143: HMBC NMR ($CDCl_3$) of *cis*-**17**.

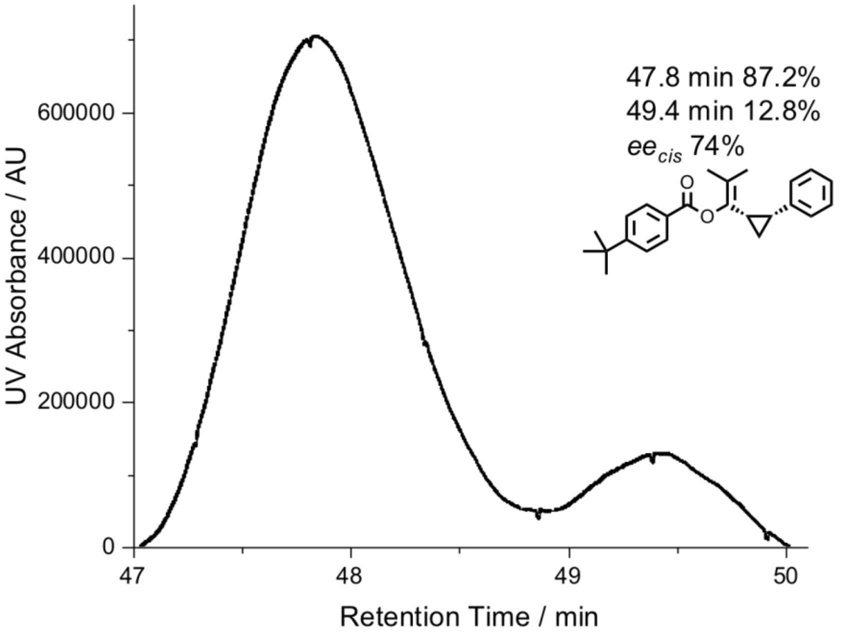

Figure S144: Chiral Stationary Phase HPLC ((S,S)Whelk, *n*-hexane-isopropanol 99.5 : 0.5, 303 K, load Et_2O, flowrate 0.25 $mLmin^{-1}$) of 83 : 17 *er cis*-**S17**. Retention times (min): 47.8, 49.4. The absolute stereochemistry of the products was not determined. The (1*S*,2*R*)-**S17** isomer is shown for illustrative purposes only.

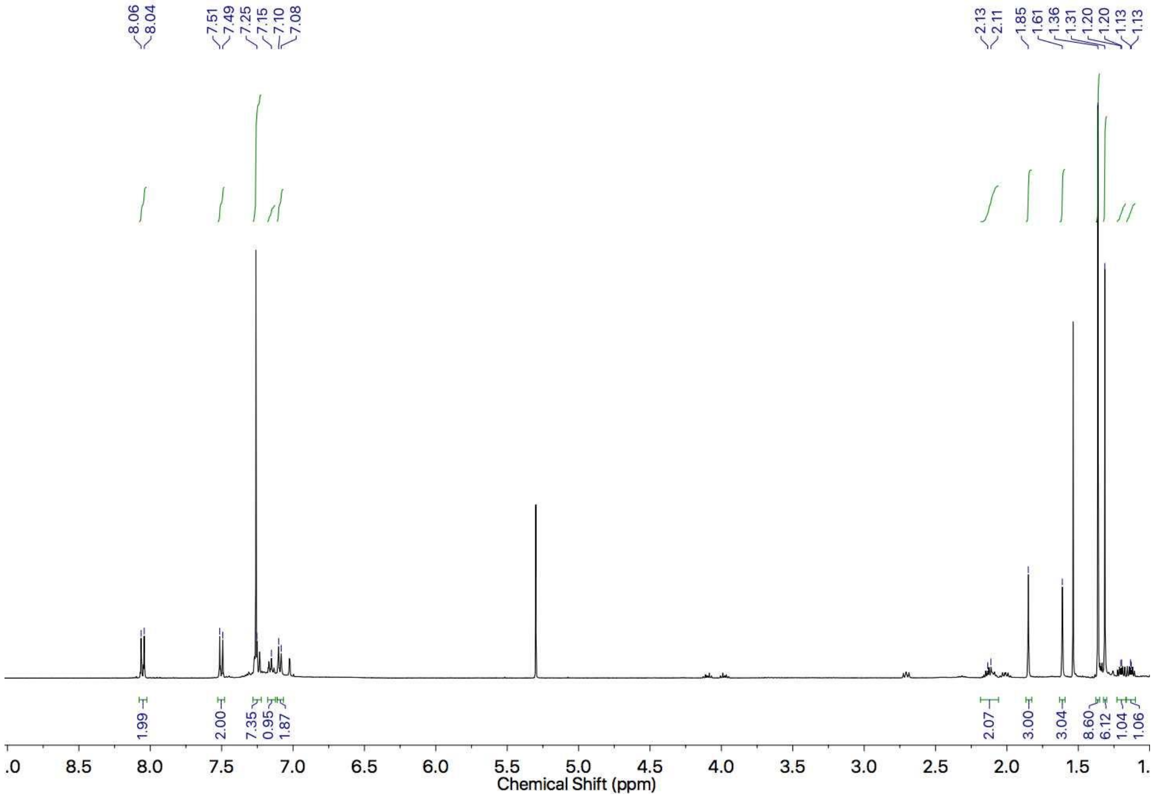

Figure S145: ^{1}H NMR (400 MHz, $CDCl_3$) of *trans*-**17**.

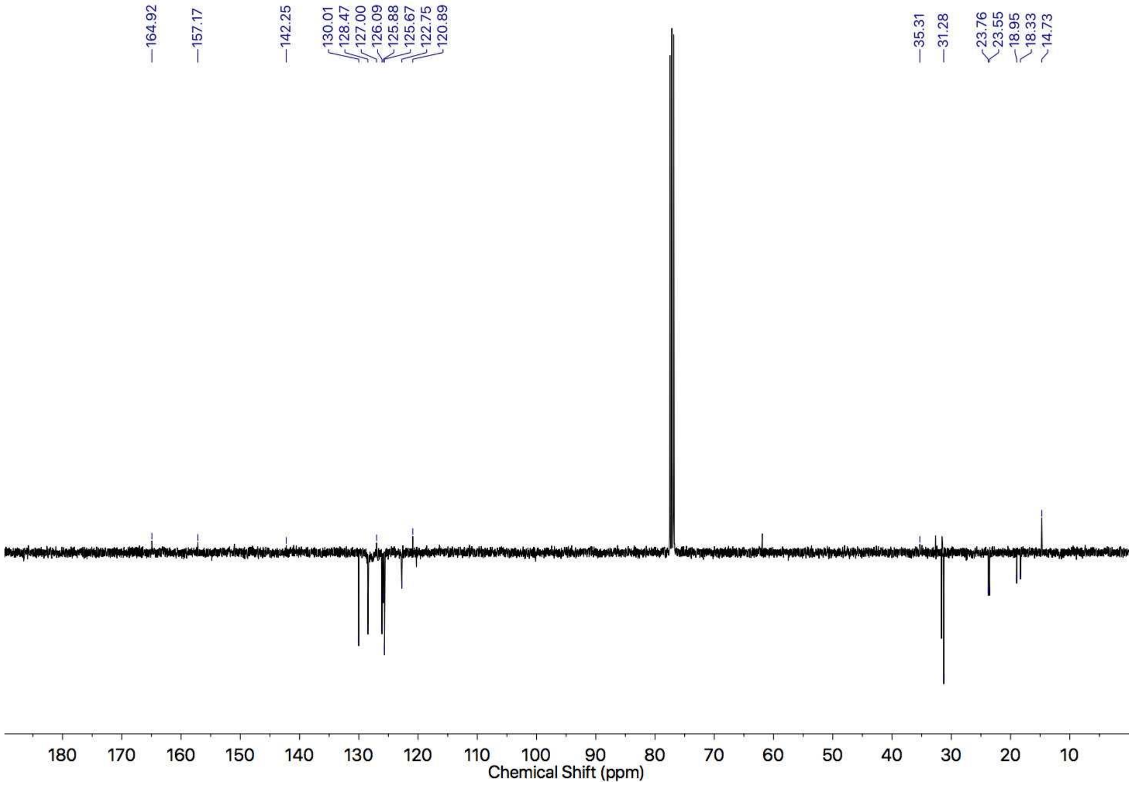

Figure S146: JMOD NMR (101 MHz, $CDCl_3$) of *trans*-**17**.

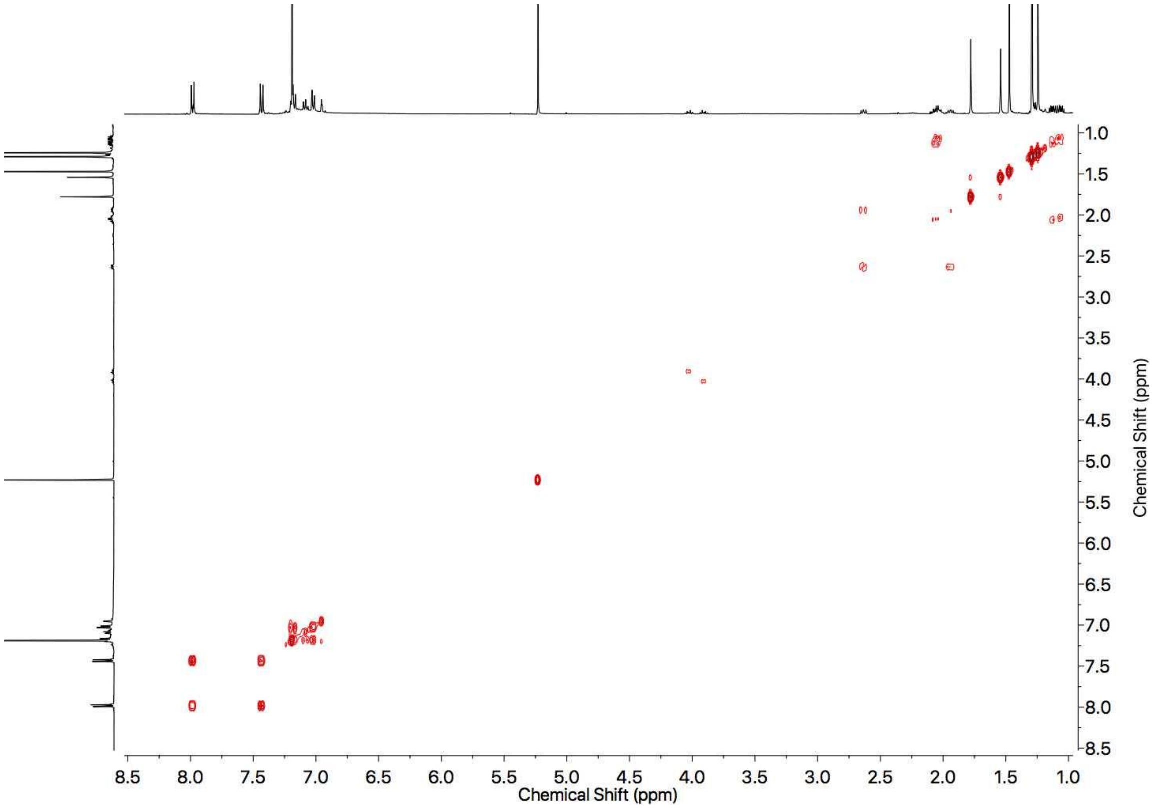

Figure S147: COSY NMR ($CDCl_3$) of *trans*-**17**.

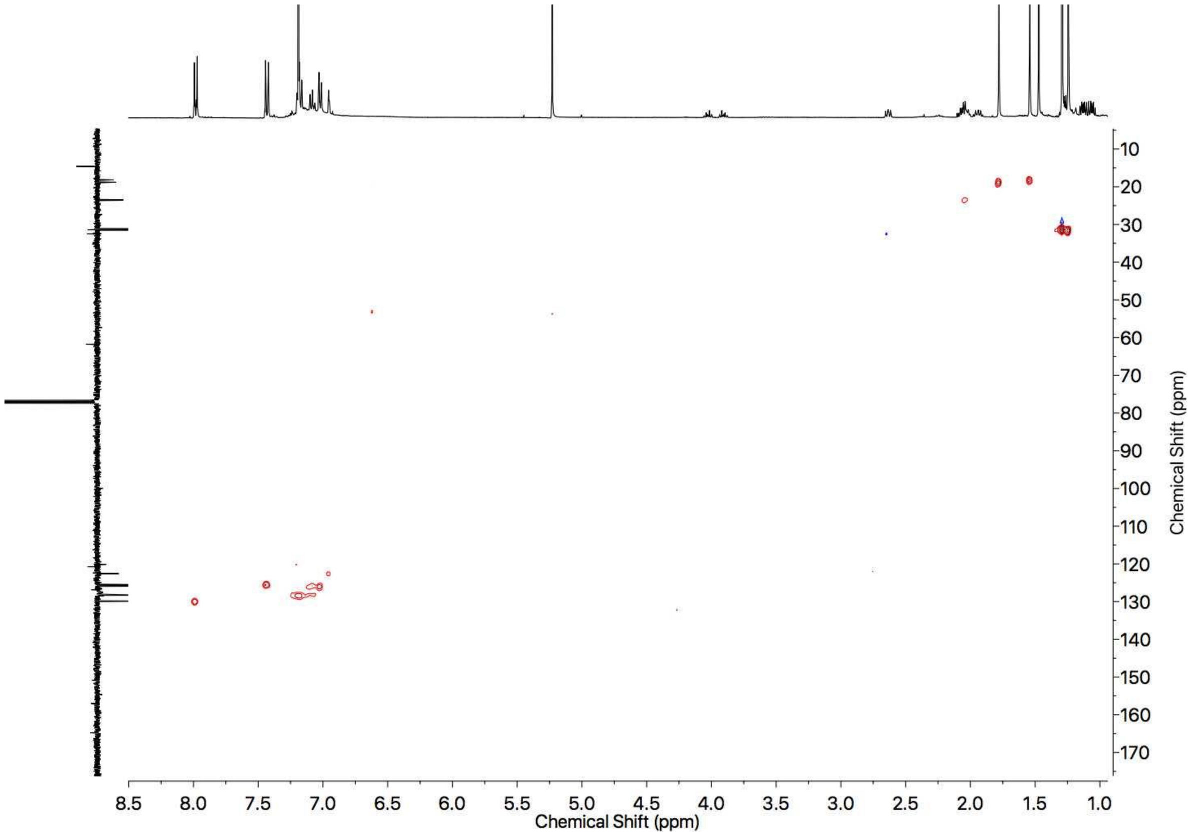

Figure S148: HSQC NMR ($CDCl_3$) of *trans*-**17**.

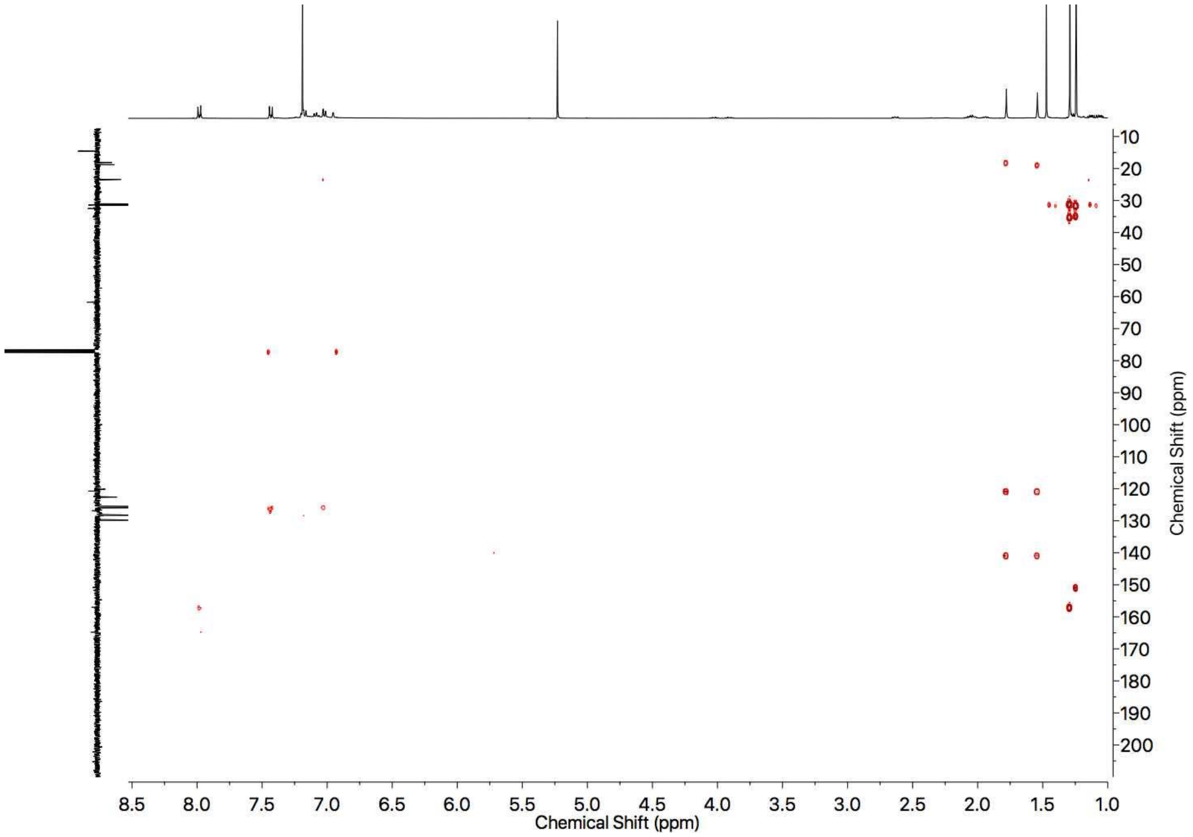

Figure S149: HMBC NMR ($CDCl_3$) of *trans*-**17**.

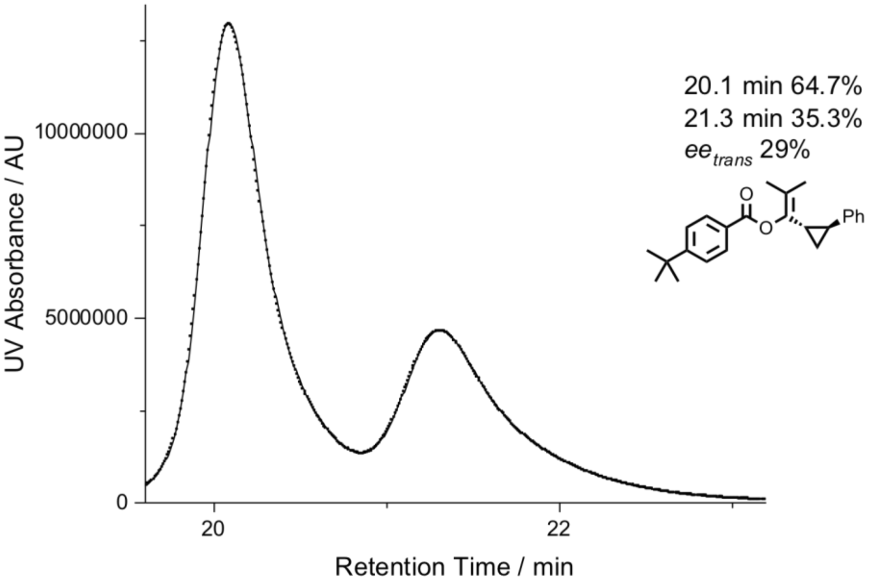

Figure S150: Chiral Stationary Phase HPLC (RegisPack, *n*-hexane-isopropanol 99 : 1, 303 K, load Et_2O, flowrate 0.25 $mLmin^{-1}$) of 65 : 35 *er trans*-**17**. Retention times (min): 20.1, 21.3. The absolute stereochemistry of the products was not determined. The (1*S*,2*S*)-**17** isomer is shown for illustrative purposes only.

Cyclopropanes **18**

**Catalyst**	**Yield / %**	***dr***	***er*cis**	***er*trans**
$(Ph_3P)AuCl$[a]	99	95 : 5	1 : 1	1 : 1
[Au((*R*mp)-**6**)(Cl)][b]	68	96 : 4	89 : 11	70 : 30

Table S13. Summary of reactions leading to cyclopropanes **18**.

Cis-**18**

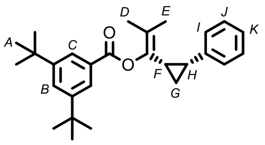

δ_H ($CDCl_3$, 400 MHz) 7.84 (2H, d, *J* = 1.9, ***H***C), 7.66 (1H, t, *J* = 1.9, ***H***B), 7.25 (2H, t, *J* = 7.6, ***H***J), 7.18 (1H, tt, *J* = 7.3, 2.0, ***H***K), 7.15 (2H, d, *J* = 7.1, ***H***I), 2.42-2.29 (2H, m, ***H***F, ***H***G), 1.67 (3H, s, ***H***E), 1.49 (3H, s, ***H***D), 1.37 (18H, s, ***H***A) 1.32-1.26 (1H, m, ***H***H), 1.15 (1H, q, *J* = 6.1, ***H***H).

δ_C ($CDCl_3$, 101 MHz) 165.5, 151.2, 139.5, 138.7, 129.3, 127.8, 127.8, 127.5, 125.8, 124.2, 123.3, 35.1, 31.5, 23.9, 21.8, 18.8, 17.9, 11.8.

HR-EI-MS m/z 404.2703 [$MS^{+\cdot}$] (calc. m/z for $C_{28}H_{36}O_2$ 404.2710).

Trans-**18**

Colourless oil (*dr cis-trans* 95 : 5, NMR yield 94 mg, 0.232 mmol, 99%)

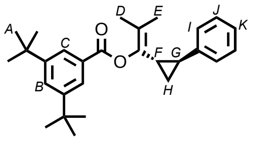

δ_H ($CDCl_3$, 400 MHz) 7.97 (2H, d, *J* = 1.9, ***H***C), 7.68 (1H, t, *J* = 1.9, ***H***B), 7.26 (2H, tt, *J* = 7.5, 1.3, ***H***J), 7.15 (1H, tt, *J* = 7.4, 1.9, ***H***K), 7.10 (2H, dd, *J* = 7.4, 1.5, ***H***I), 2.19-2.07 (2H, m, ***H***F, ***H***G), 1.86 (3H, s, ***H***E), 1.63 (3H, s, ***H***D), 1.38 (18H, s, ***H***A), 1.23 (1H, ddd, *J* = 11.1, 5.1, 6.0, ***H***H), 1.15 (1H, ddd, *J* = 11.0, 5.7, 5.1, ***H***H′).

δ_C ($CDCl_3$, 101 MHz) 165.6, 151.4, 142.0, 141.0, 129.1, 128.5, 127.7, 126.1, 125.9, 124.3, 120.9, 35.1, 31.5, 23.8, 23.7, 19.0, 18.4, 14.8.

HR-EI-MS m/z 404.2711 [$M^{+\cdot}$] (calc. m/z for $C_{28}H_{36}O_2$ 404.2710).

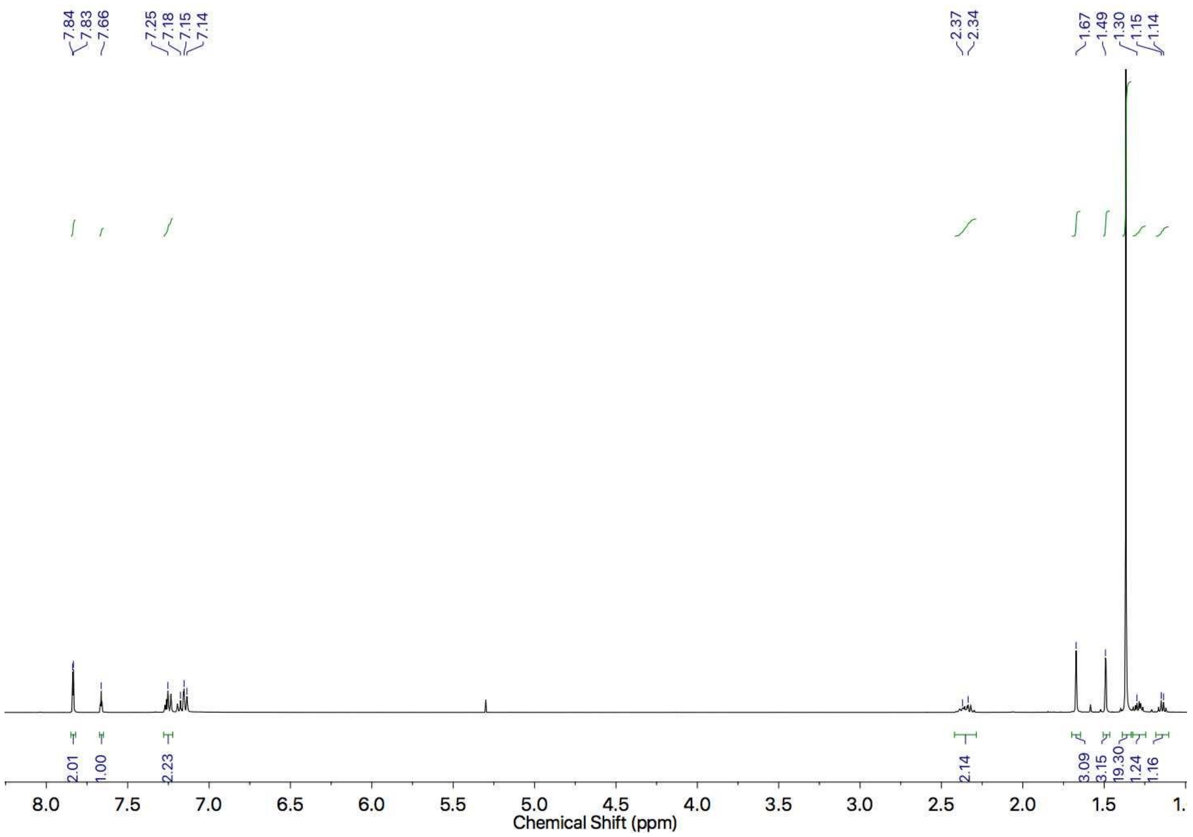

Figure S151: ^{1}H NMR (400 MHz, $CDCl_3$) of *cis*-**18**.

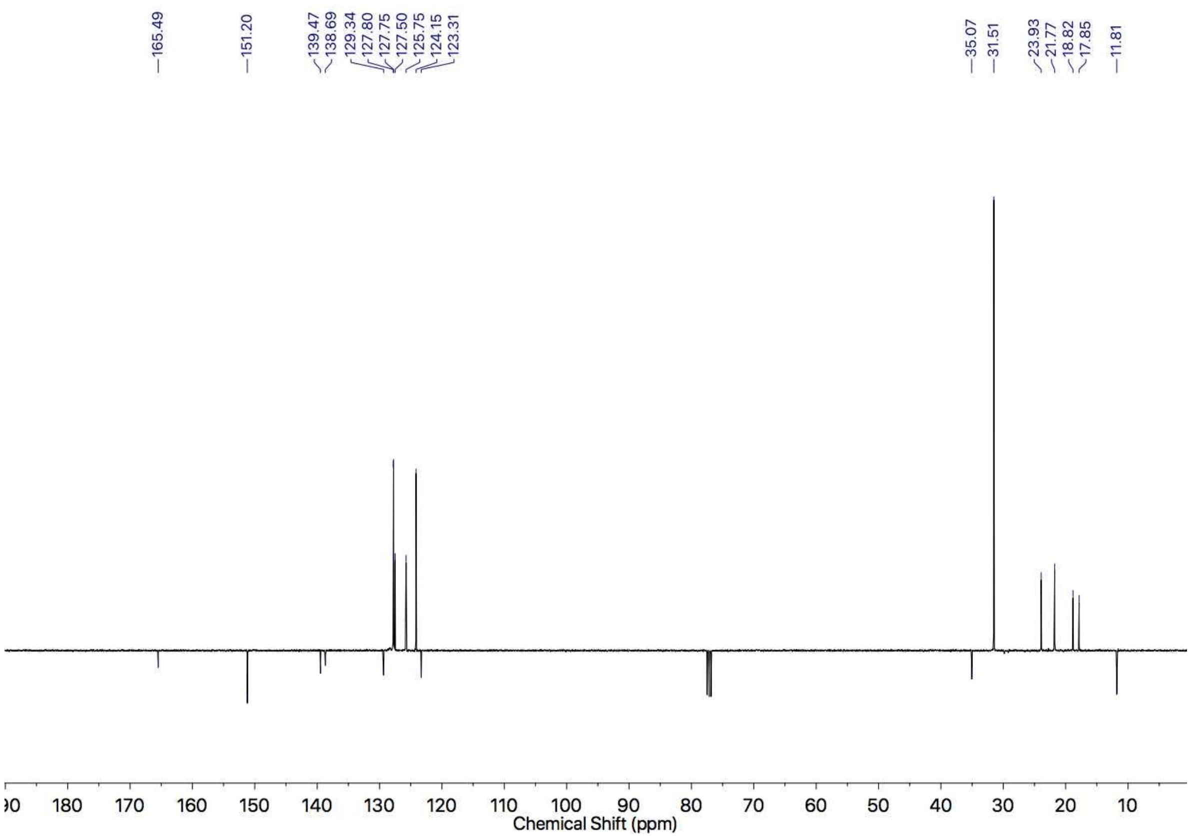

Figure S152: JMOD NMR (101 MHz, $CDCl_3$) of *cis*-**18**.

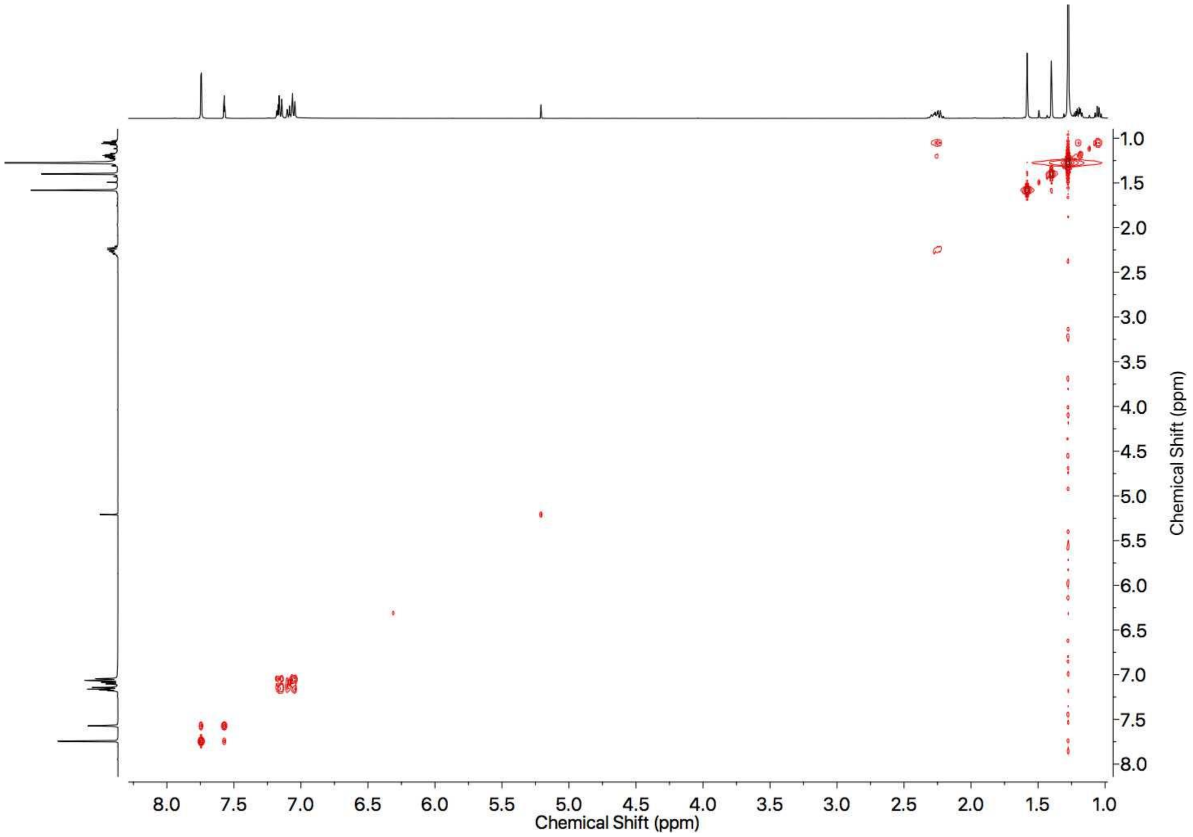

Figure S153: COSY NMR ($CDCl_3$) of *cis*-**18**.

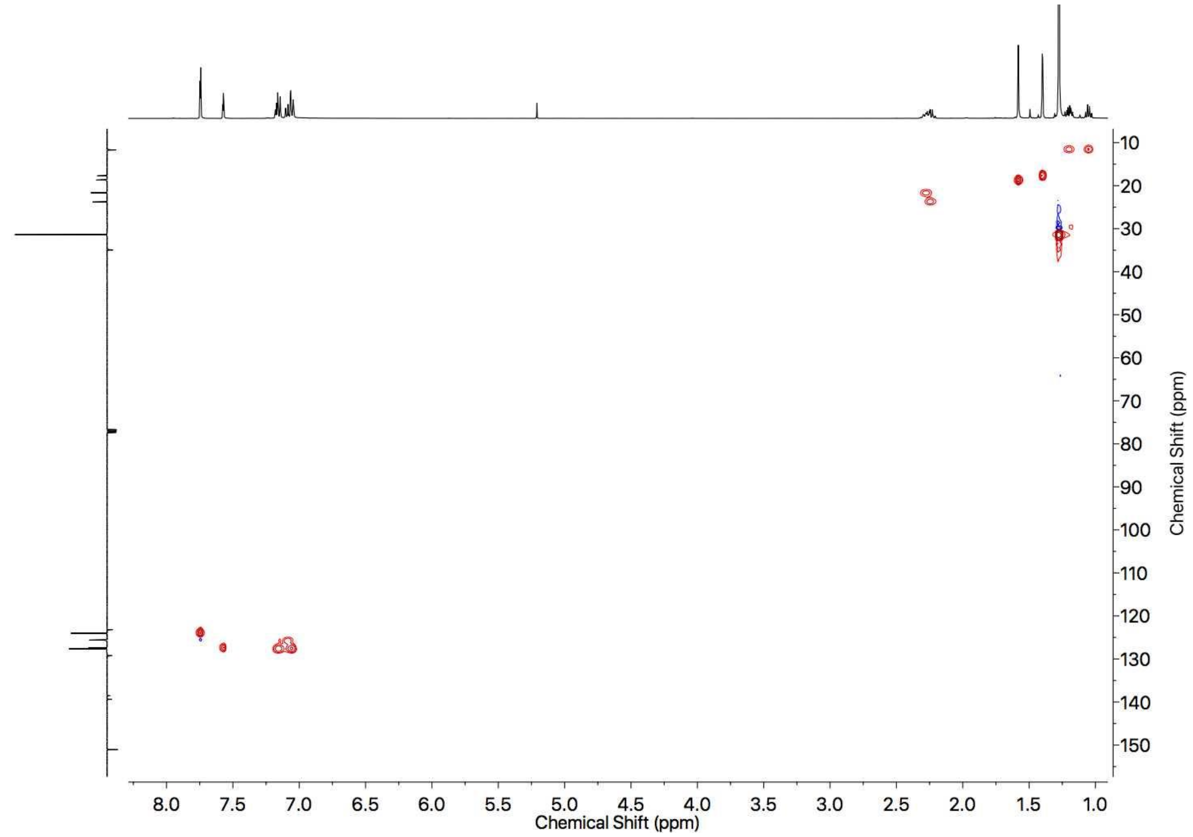

Figure S154: HSQC NMR ($CDCl_3$) of *cis*-**18**.

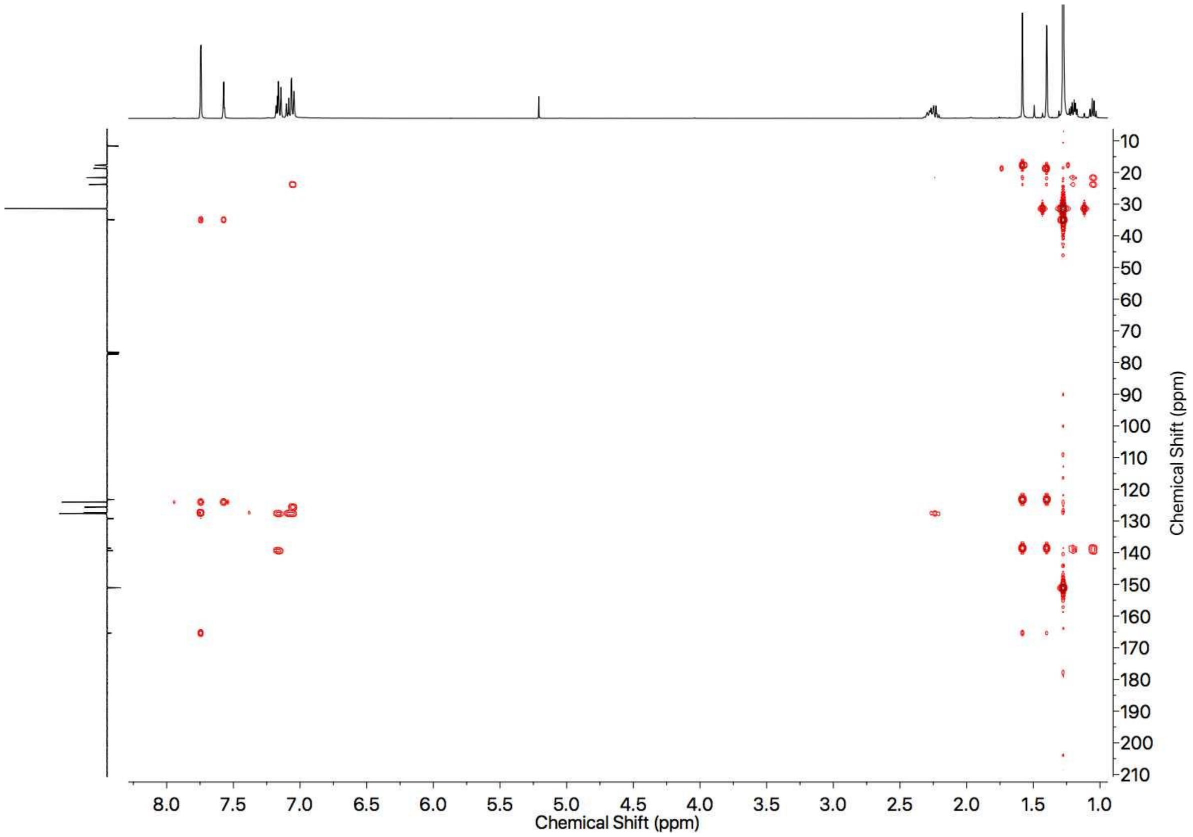

Figure S155: HMBC NMR ($CDCl_3$) of *cis*-**18**.

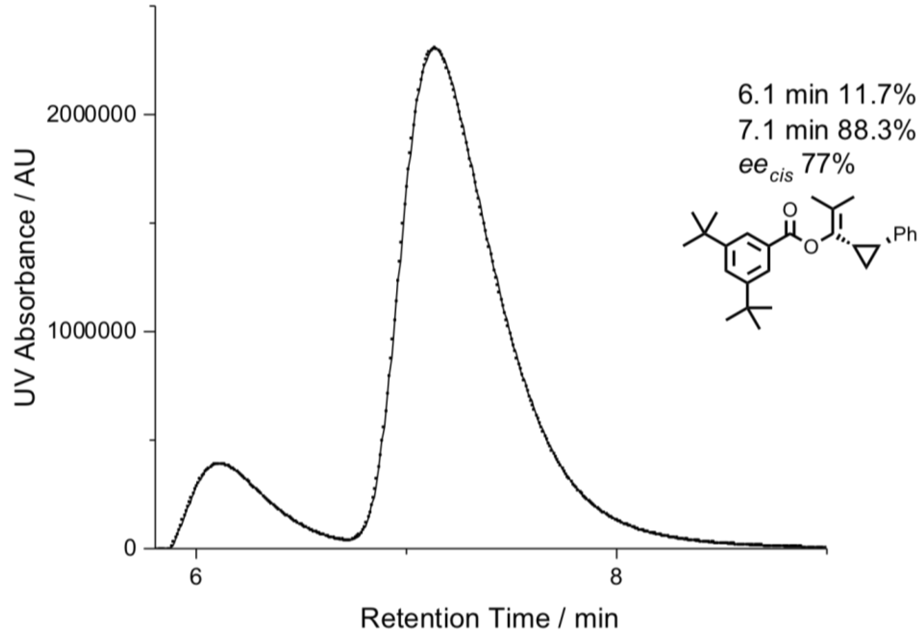

Figure S156: Chiral Stationary Phase HPLC (RegisPack, *n*-hexane-isopropanol 99.8 : 0.2, 303 K, load Et_2O, flowrate 0.75 $mLmin^{-1}$) of 88 : 12 *er cis*-**18**. Retention times (min): 6.1, 7.1. The absolute stereochemistry of the products was not determined. The (1*S*,2*R*)-**18** isomer is shown for illustrative purposes only.

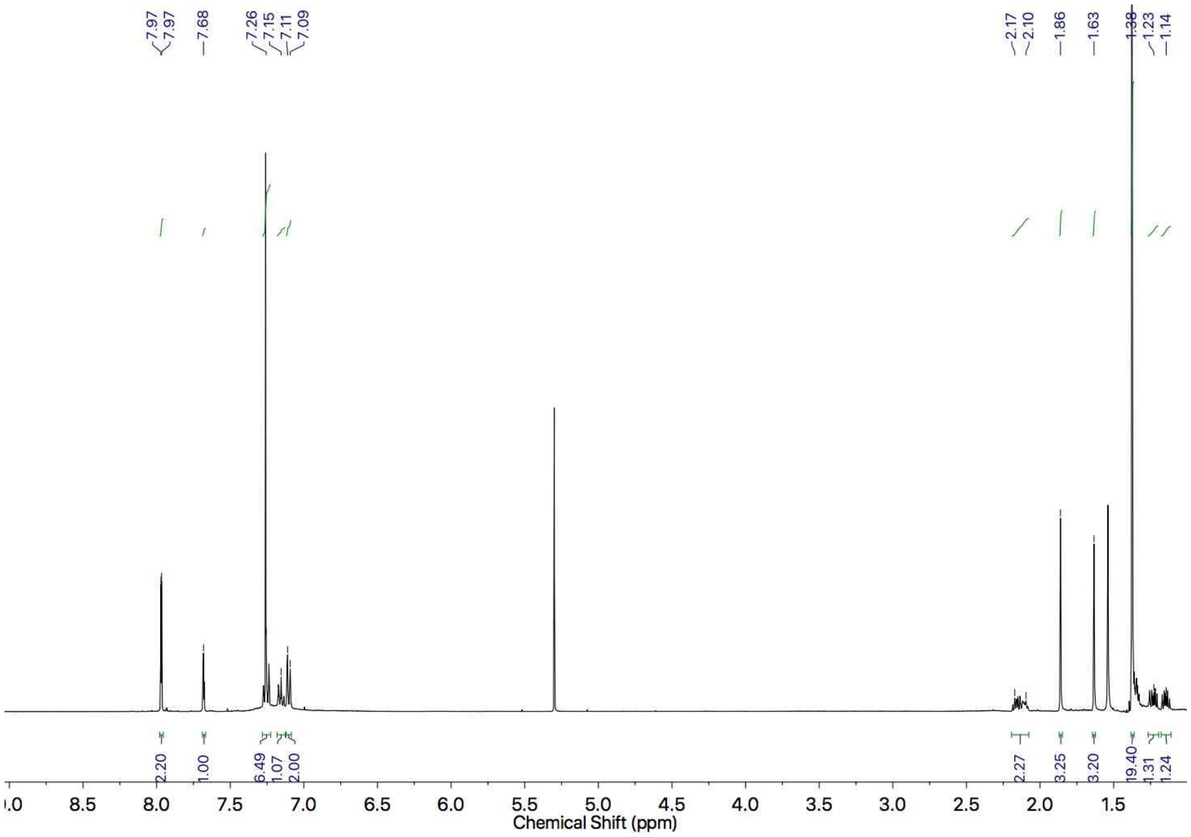

Figure S157: ^{1}H NMR (400 MHz, $CDCl_3$) of *trans*-**18**.

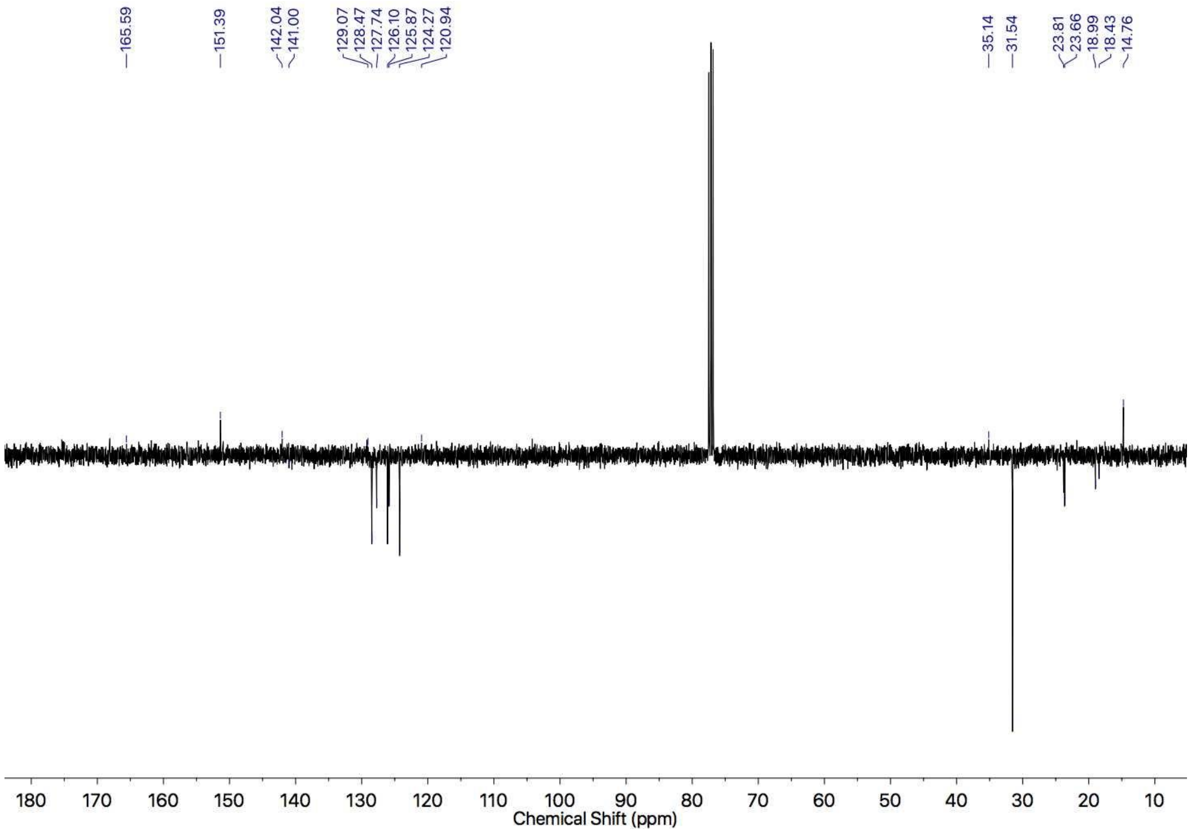

Figure S158: JMOD NMR (101 MHz, $CDCl_3$) of *trans*-**18**.

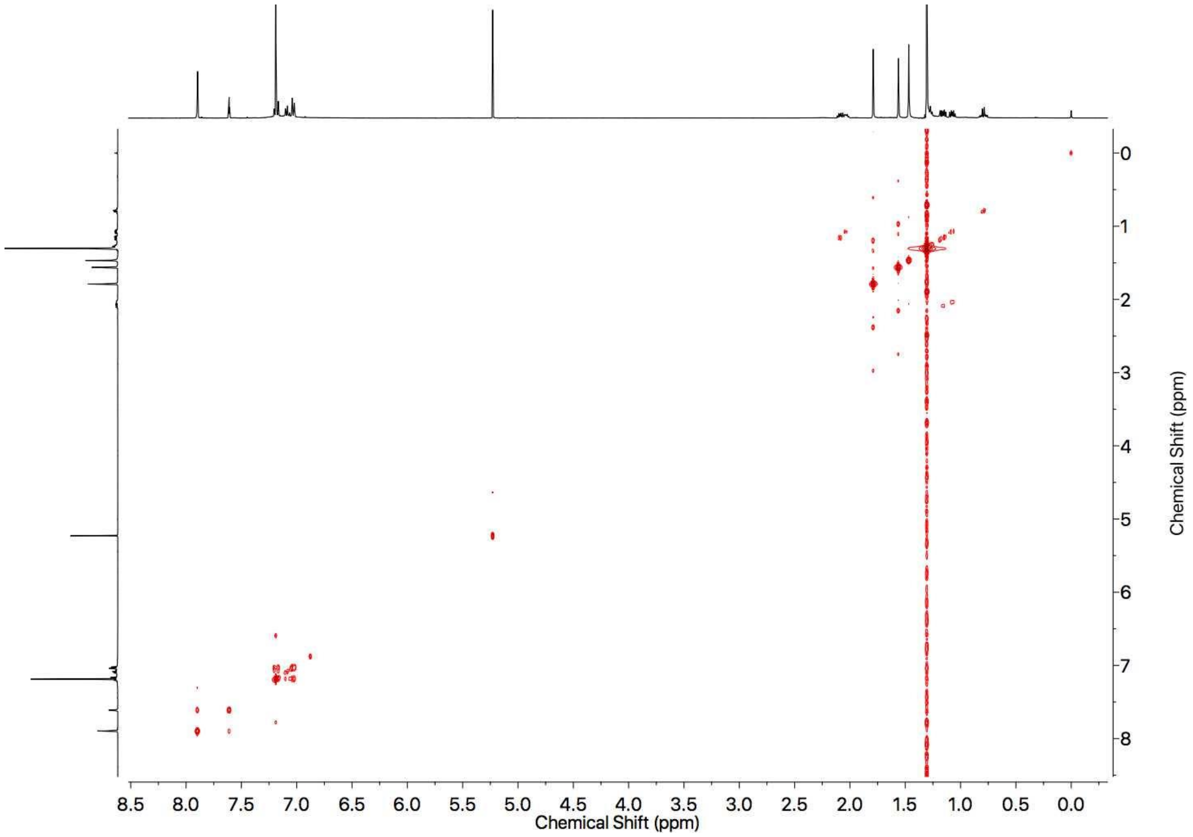

Figure S159: COSY NMR ($CDCl_3$) of *trans*-**18**.

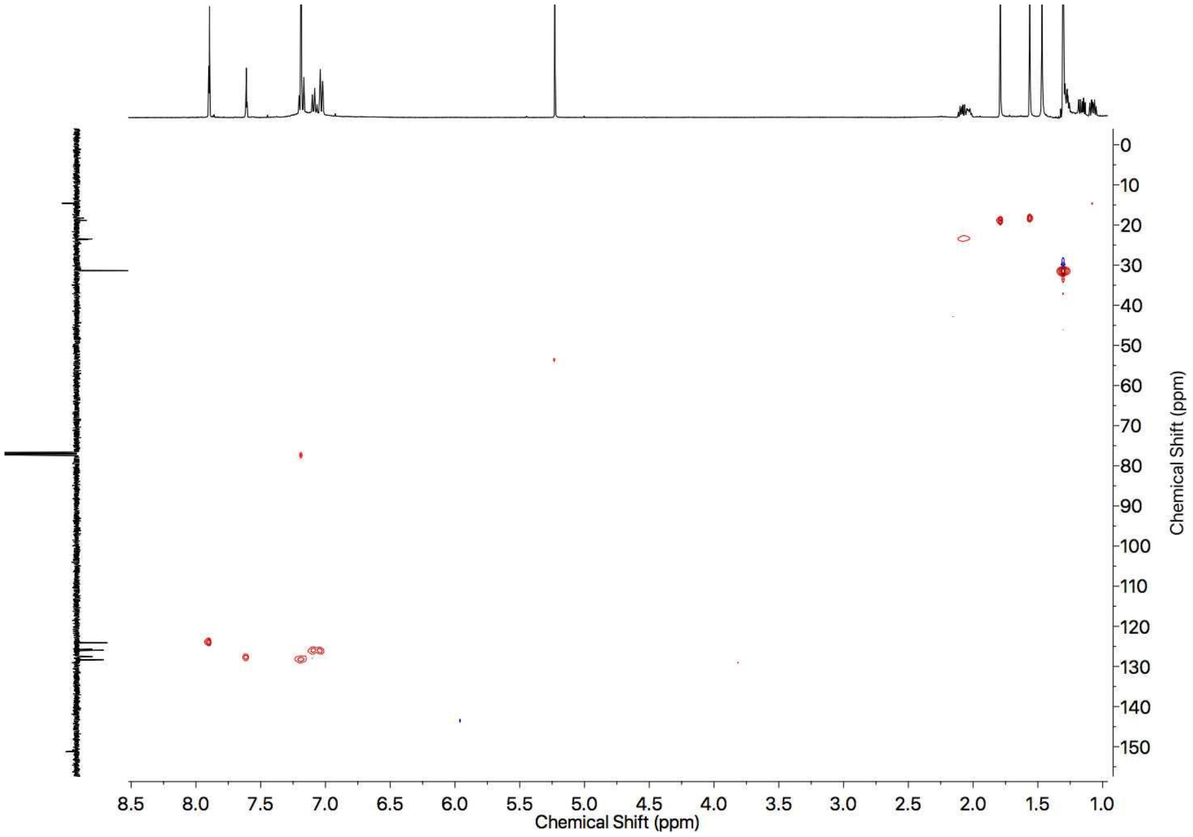

Figure S160: HSQC NMR ($CDCl_3$) of *trans*-**18**.

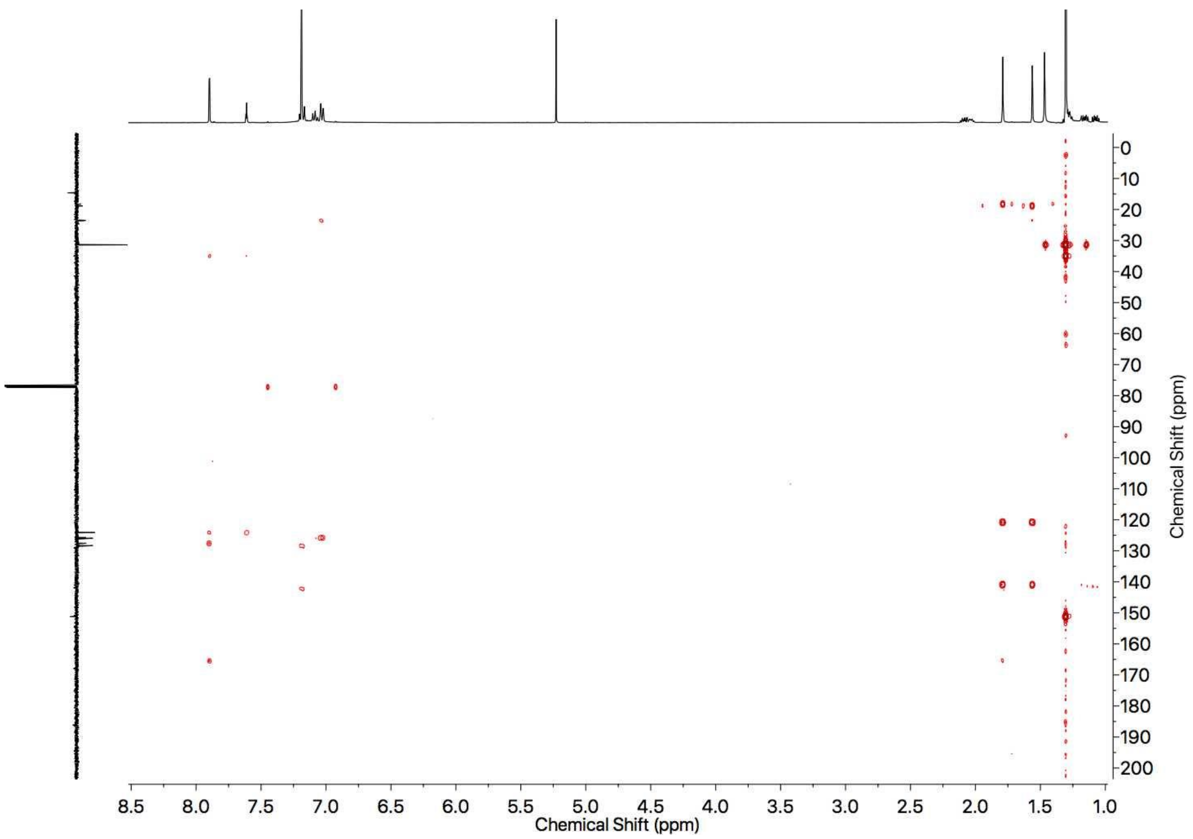

Figure S161: HMBC NMR ($CDCl_3$) of *trans*-**18**.

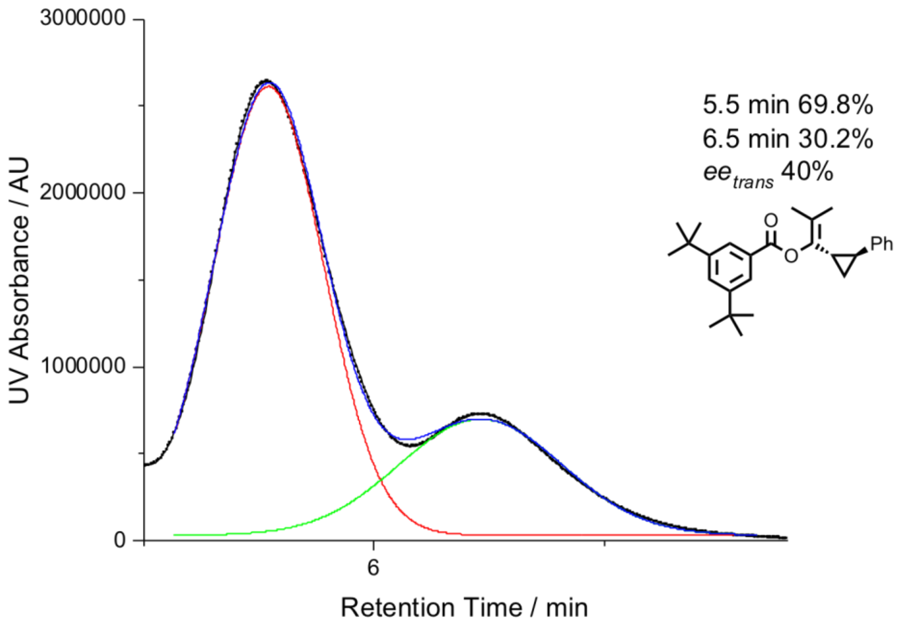

Figure S162: Chiral stationary phase HPLC (RegisPack, hexane-isopropanol 99.8 : 0.2, 303 K, load Et_2O, flowrate 0.75 mLmin^{-1}) of 70 : 30 *er trans*-**18**. Retention times (min): 5.5, 6.5. The absolute stereochemistry of the products was not determined. The (1*S*,2*S*)-**18** isomer is shown for illustrative purposes only.

The absolute configuration of *cis*-**9** was determined by comparison to known selectivity of (*R*)-DTBM-SEGPHOS(AuCl)$_2$ by Toste *et al.* *cis*-**9**, *cis*-**13**, *cis*-**15**, *cis*-**16** were converted to ketone **S9** as per the approach to Toste *et al.*[6] Converging HPLC traces demonstrate the matching absolute stereochemistry for (1*S*,2*R*)-**9**, (1*S*,2*R*)-**15**, and (1*S*,2*R*)-**16**.

cis-**S9**

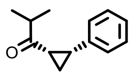

$LiAlH_4$ (50 µL, 50 µmol, 2 eq., 1 M) was added to a solution of *cis*-**17** (8.7 mg, 25 µmol, 1 eq.) in anhydrous THF (0.2 mL) at 0 °C, then stirred at room temperature for 2 h. The reaction was quenched with MeOH, washed with 5% w/v $LiCl_{aq}$ (15 mL) and extracted with CH_2Cl_2 (3 x 15 mL). The combined organic phases were dried ($MgSO_4$) and reduced *in vacuo*. The residue was dissolved in CH_2Cl_2, filtered through a Celite® and the filtrate reduced *in vacuo*, yielding *cis*-**S9** as a colourless oil (2.4 mg, 13 µmol, 51%).

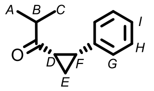

δ_H ($CDCl_3$, 400 MHz) 7.27-7.14 (5H, m, ***H***$_G$, ***H***$_H$, ***H***$_I$), 2.68 (1H, ddd (app q), *J* = 8.3, ***H***$_E$), 2.59 (1H, sept., *J* = 6.9, ***H***$_B$), 2.50 (1H, ddd, *J* = 9.2, 7.5, 5.8, ***H***$_D$), 1.87 (1H, ddd, *J* = 7.6, 5.7, 4.9, ***H***$_F$), 1.31 (1H, ddd, *J* = 8.5, 7.5, 4.8, ***H***$_{F'}$), 0.99 (3H, d, *J* = 6.9, ***H***$_A$), 0.93 (3H, d, *J* = 6.9, ***H***$_C$).

δ_C ($CDCl_3$, 101 MHz) 209.4, 136.2, 129.2, 127.9, 126.7, 41.9, 28.9, 28.3, 17.9, 17.8, 11.7.

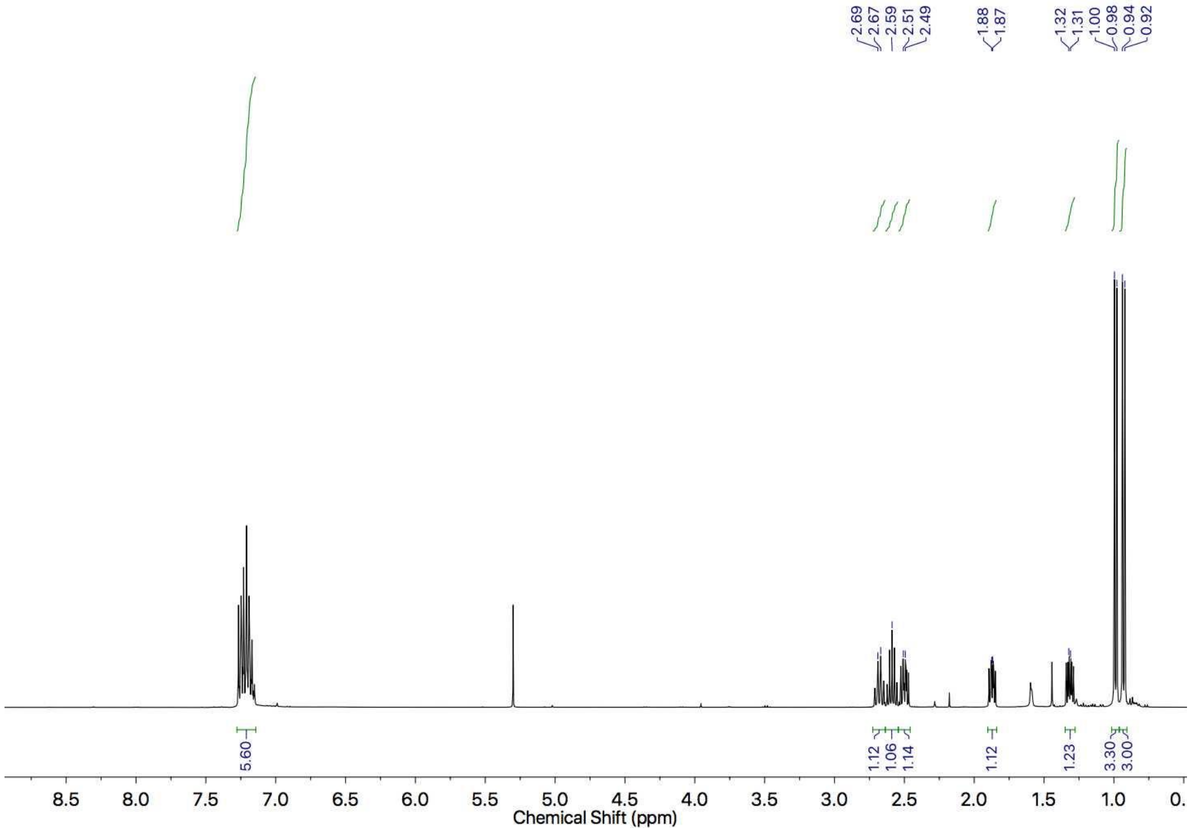

Figure S163: ^{1}H NMR (400 MHz, $CDCl_3$) of *cis*-**S9**.

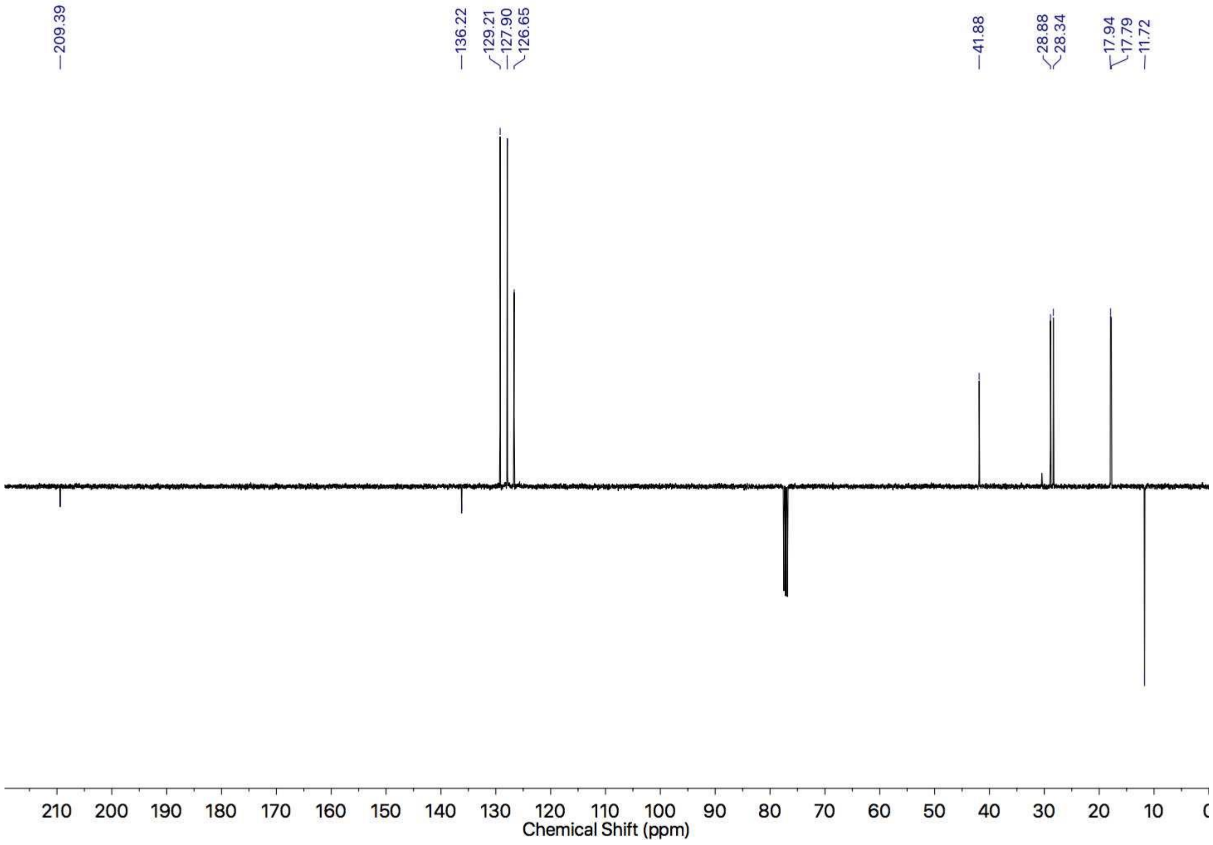

Figure S164: JMOD NMR (101 MHz, $CDCl_3$) of *cis*-**S9**.

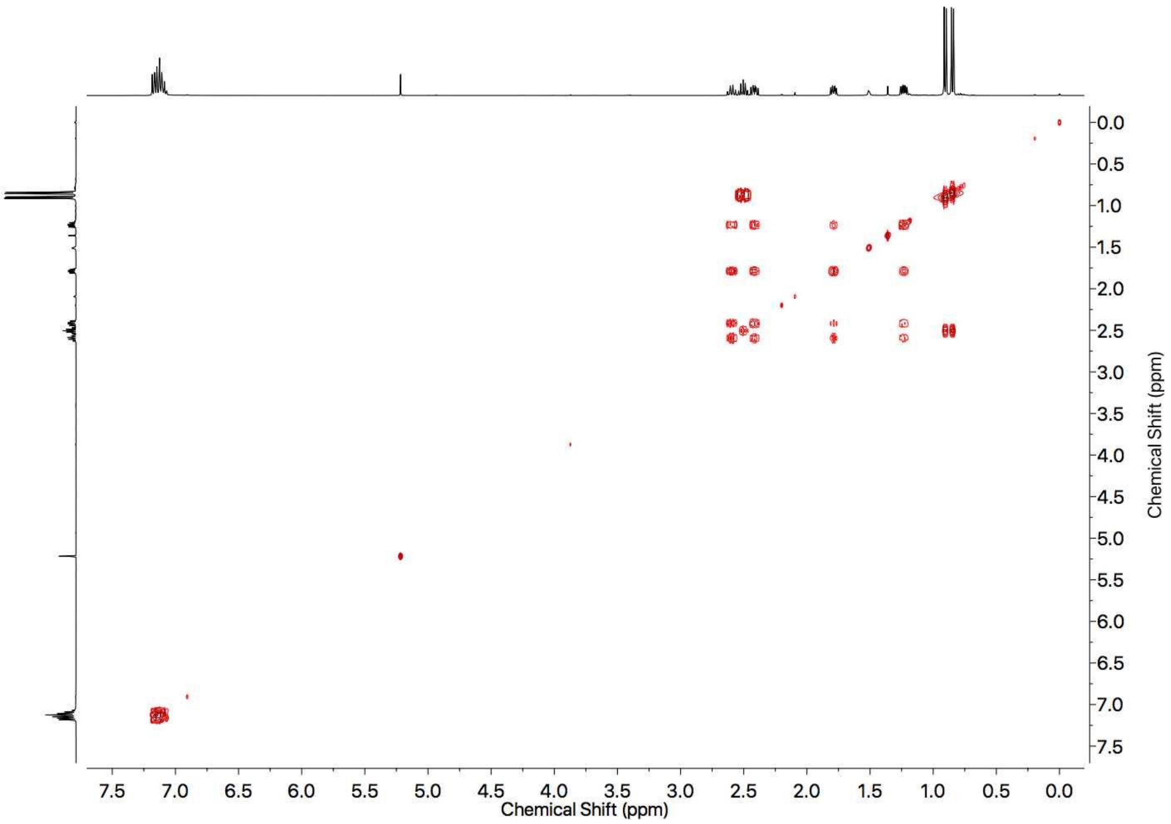

Figure S165: COSY NMR ($CDCl_3$) of *cis*-**S9**.

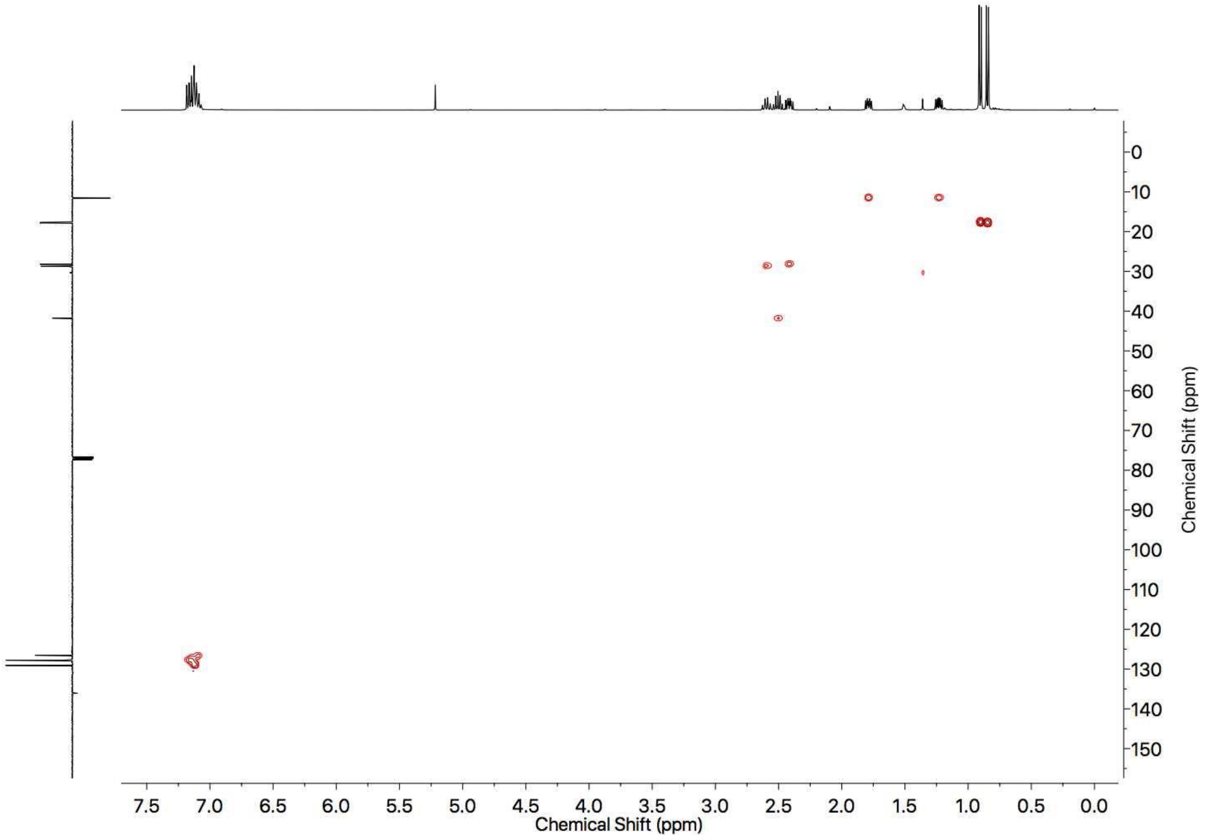

Figure S166: HSQC NMR ($CDCl_3$) of *cis*-**S9**.

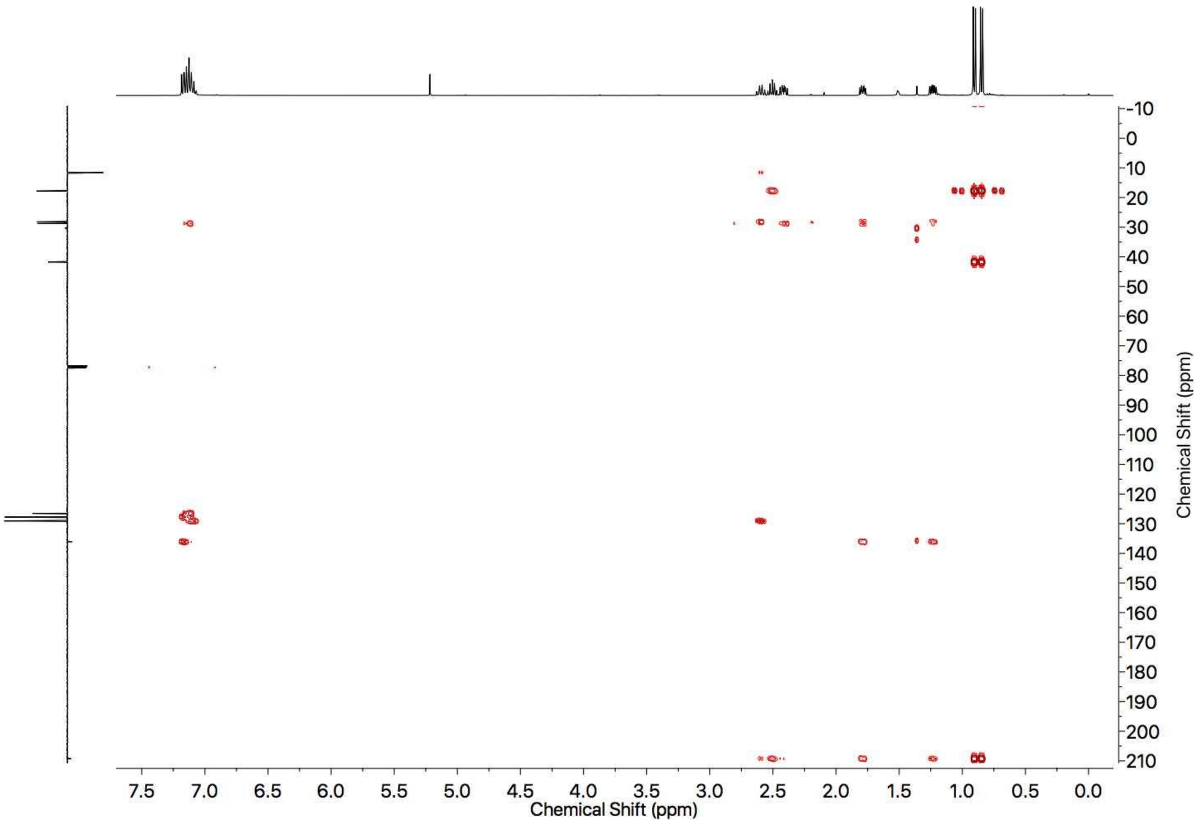

Figure S167: HMBC NMR ($CDCl_3$) of *cis*-**S9**.

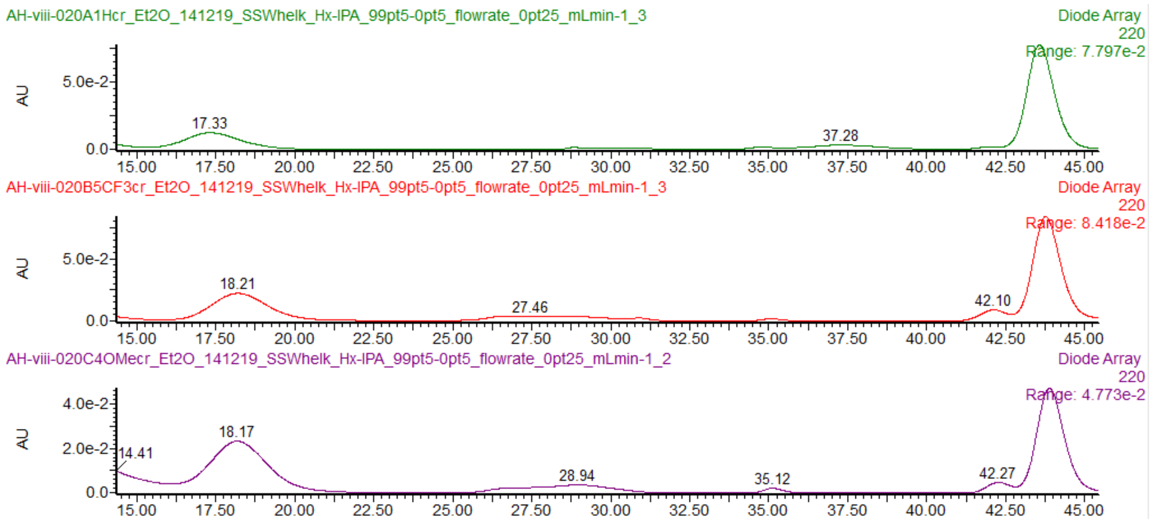

Figure S168: Chiral stationary phase HPLC ((*S*,*S*)Whelk, hexane-isopropanol 99.5 : 0.5, 303 K, load petrol, flowrate 0.25 mLmin^{-1}) of *cis*-**S9** crude reaction mixtures. 220 nm UV absorbance shown. Retention times (min): 18.1, 43.7. (1) Reduction of *cis*-**9** (*er* 22 : 78) to *cis*-**S9** (apparent *er* 22 : 78 integrals 22221 (17.3 min) : 81636 (43.6 min)). (2) Reduction of *cis*-**15** (*er* 27 : 73) to *cis*-**S9** (apparent *er* 29 : 71 integrals 36466 (18.2 min) : 90032 (43.7 min)). (3) Reduction of *cis*-**16** (*er* 30 : 70) to *cis*-**S9** (apparent *er* 39 : 61 integrals 32137 (18.2 min) : 50259 (43.9 min)).

6. Additional Reaction Scope

To investigate the potential of [Au((R_{mp})-**6**)(Cl)] in other reactions we performed two previously reported cycloisomerisation reactions and an intramolecular hydroamination reaction to compare the outcomes to those achieved with (*R*)-DTBM-SEGPHOS®$(AuCl)_2$. In all cases little or no enantioselectivity was observed with [Au((R_{mp})-**6**)(Cl)]. Similar results were obtained for (*R*)-DTBM-SEGPHOS®$(AuCl)_2$ except in the case of the hydroamination reaction (modest enantioselectivity).

Cycloisomerisation 1[13]

Enyne cyclisation substrate **S10** was synthesised according to a literature route.[14]

Compound **S11**

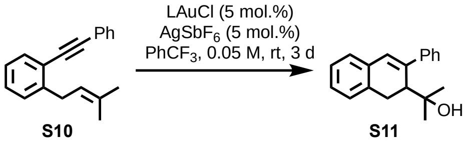

General procedure: A foil covered CEM MW vial was charged with $AgSbF_6$ (1.1 mg, 3.1 µmol, 0.05 eq.) and, in the case of [Au((R_{mp})-**6**)(Cl)], $[Cu(MeCN)_4]PF_6$ (1.2 mg, 3.1 µmol, 0.05 eq.), then purged with N_2. [Au(L)(Cl)] (3.1 µmol, 0.05 eq.) was transferred in $PhCF_3$ (0.50 mL) and stirred for 5 minutes. **S10** (15.3 mg, 0.062 mmol, 1 eq.) in $PhCF_3$ (0.2 mL) and H_2O (2.8 mg, 0.155 mmol, 2.5 eq.) were added. The mixture was stirred at rt for 3 days. After 3 days, the reaction was quenched with 1 drop of NEt_3, and concentrated *in vacuo*. **S11** was purified by column chromatography (SiO_2, petrol-EtOAc 0→20%) yielding a white solid. Enantiomeric excess was evaluated by chiral stationary phase HPLC. Spectra match literature.[13]

δ_H (CD_2Cl_2, 400 MHz) 7.58 – 7.50 (2H, m), 7.43 – 7.34 (2H, m), 7.33 – 7.25 (1H, m), 7.21 – 7.09 (4H, m), 6.86 (1H, s), 3.32 – 3.19 (2H, m), 3.11 (1H, dd, J = 7.1, 2.3 Hz), 1.18 – 1.12 (1H, m), 0.96 (3H, s), 0.88 (3H, s).

δ_C (CD_2Cl_2, 101 MHz) 143.9, 140.9, 136.0, 135.2, 129.1, 128.4, 128.1, 127.8, 127.6, 127.2, 126.9, 126.8, 75.3, 46.0, 31.3, 29.2, 28.1.

LRMS [M – $H_2O^{+\bullet}$] 246.1.

Table S14. Summary of reactions leading to cyclopropanes **S11**.

	**Catalyst**	**Yield / %**	***er***
1	$(Ph_3P)AuCl$	25	50 : 50
2	[Au((R_{mp})-**6**)(Cl)]	18	48 : 52
3	(*R*)-DTBM-SEGPHOS®$(AuCl)_2$	9	57 : 43

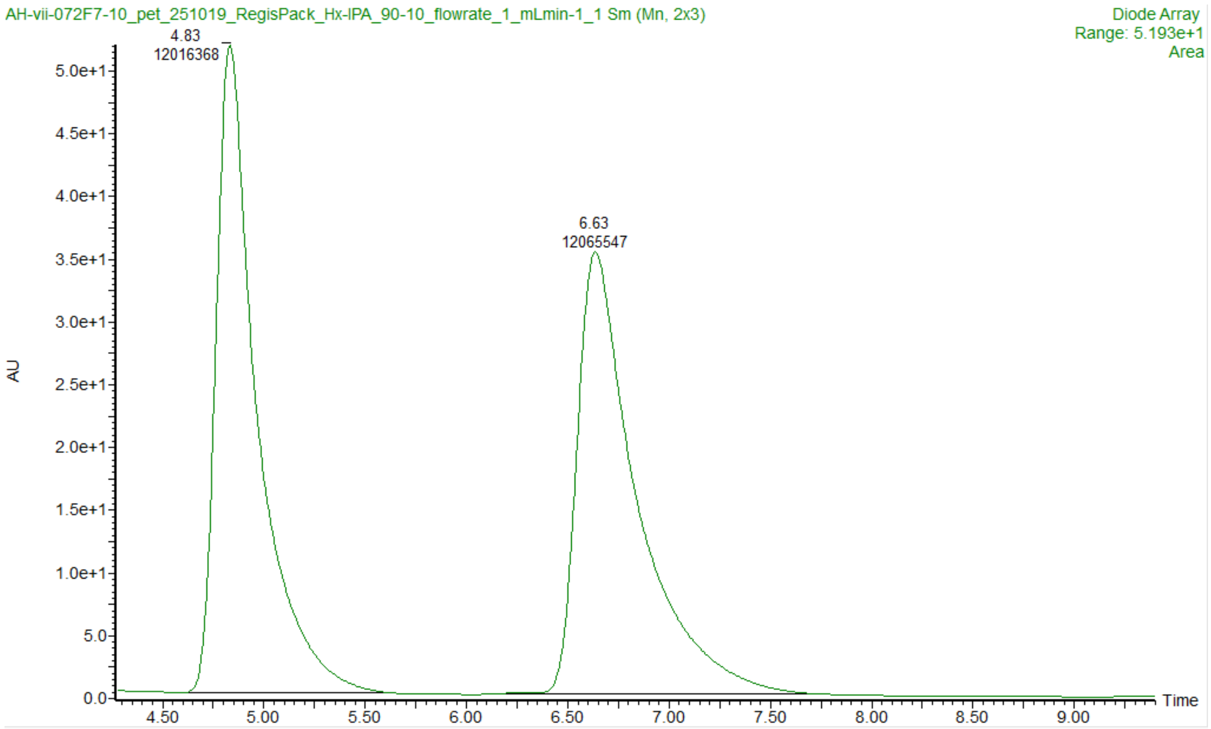

Figure S169: Entry 1. Chiral Stationary Phase HPLC (RegisPack, n-hexane-isopropanol 90 : 10, 303 K, load Et_2O, flowrate 1 mLmin^{-1}) of 50 : 50 *er*. Retention times (min): 4.8, 6.6.

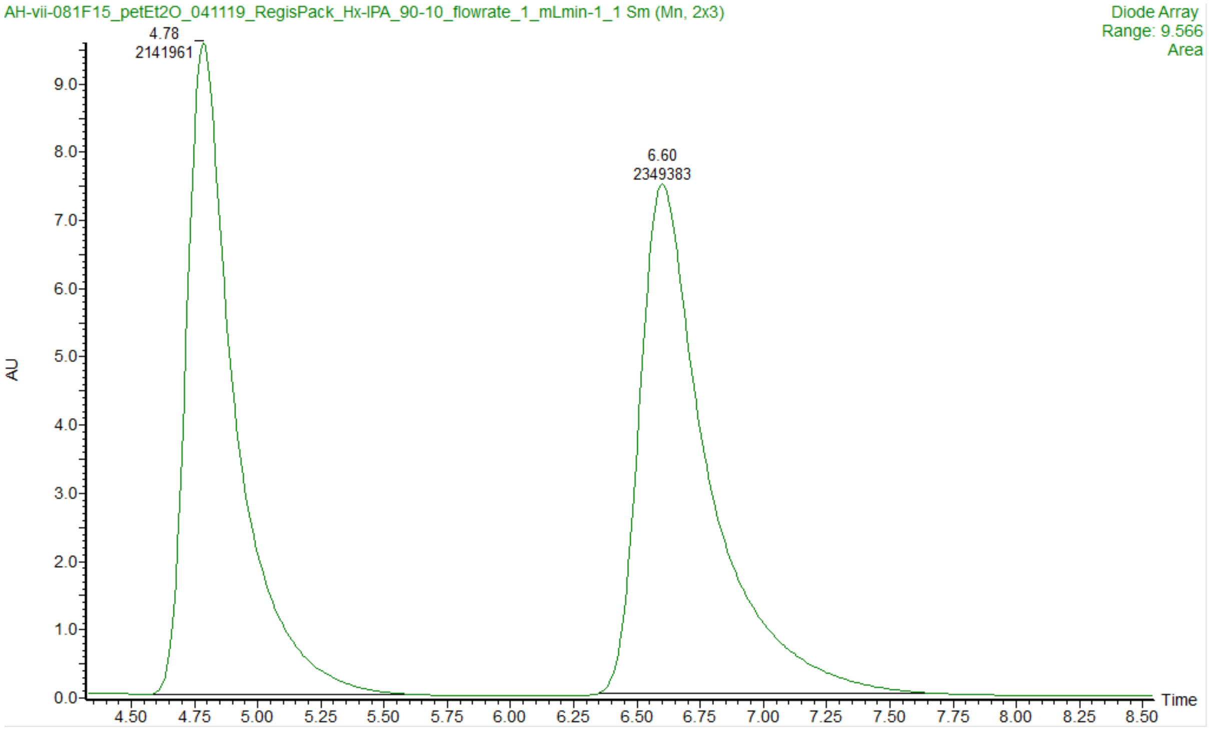

Figure S170: Entry 2. Chiral Stationary Phase HPLC (RegisPack, n-hexane-isopropanol 90 : 10, 303 K, load Et_2O, flowrate 1 mLmin^{-1}) of 48 : 52 *er*. Retention times (min): 4.8, 6.6.

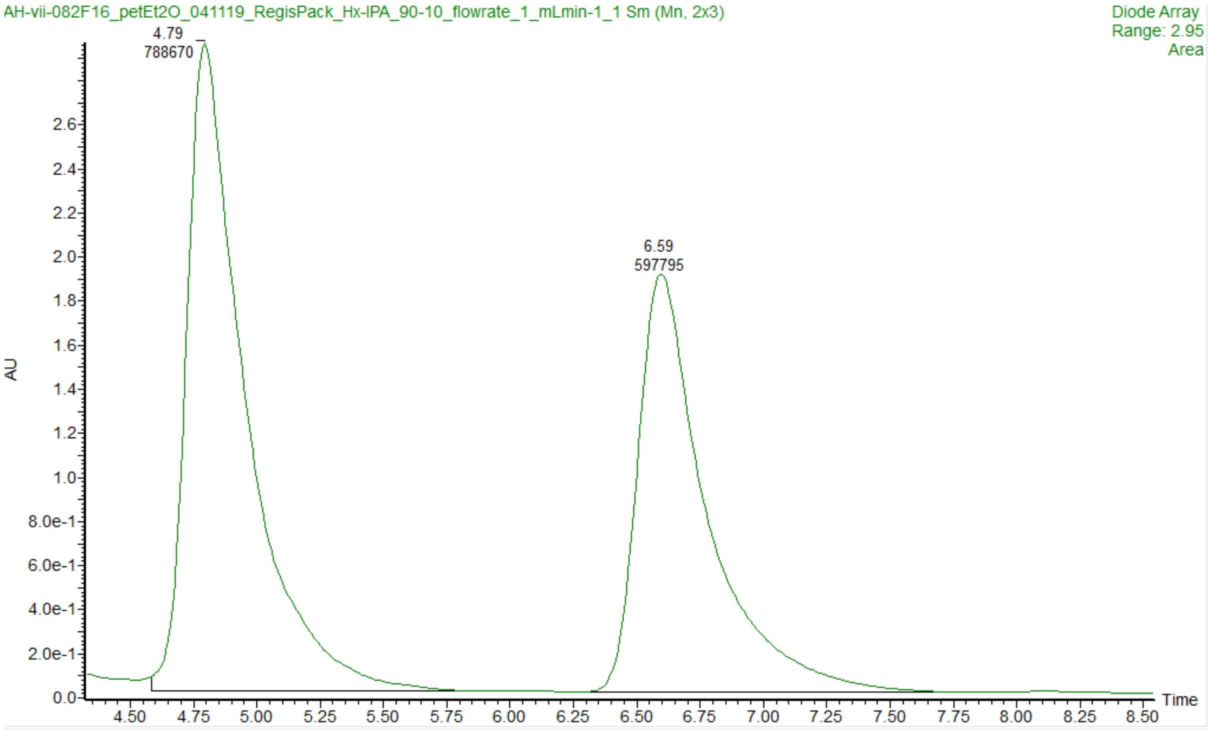

Figure S171: Entry 3. Chiral Stationary Phase HPLC (RegisPack, n-hexane-isopropanol 90 : 10, 303 K, load Et_2O, flowrate 1 $mLmin^{-1}$) of 57 : 43 *er*. Retention times (min): 4.8, 6.6.

Cycloisomerisation 2[15]

Enyne cyclisation substrate **S12** was synthesised according to a literature route.[16]

Compound **S13**

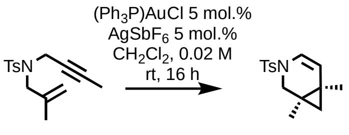

General procedure: A CEM MW vial was charged with [Au(L)(Cl)] (4.4 µmol, 0.05 eq.), $AgSbF_6$ (1.5 mg, 4.4 µmol, 0.05 eq.), and in the case of [Au((R_{mp})-**6**)(Cl)], $[Cu(MeCN)_4]PF_6$ (1.7 mg, 4.4 µmol, 0.05 eq.), and purged with N_2. Alkyne substrate **S12** (24.4 mg, 0.088 mmol, 1.0 eq.) was added in degassed anhydrous CH_2Cl_2 (4.4 mL, 0.02 M) and the reaction stirred at rt for 16 h. The mixture was filtered through Celite© and concentrated *in vacuo*. The residue was purified by column chromatography (SiO_2, isocratic pet-EtOAc 95 : 5). Spectra match literature.[15]

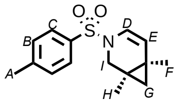

S13

δ_H ($CDCl_3$, 400 MHz) 7.65 (2H, d, J = 8.3, **H**$_C$), 7.31 (2H, d, J = 8.4, **H**$_B$), 6.25 (1H, d, J = 8.0, **H**$_E$), 5.17 (1H, d, J = 8.0, **H**$_D$), 3.77 (1H, d, J = 11.5, **H**$_I$), 3.68 (1H, d, J = 11.5, **H**$_{I'}$), 2.42 (3H, s, **H**$_A$), 1.11 (6H, s, **H**$_F$, **H**$_H$), 0.72 (1H, d, J = 4.3, **H**$_G$), 0.33 (1H, d, J = 4.3, **H**$_{G'}$).

δ_C ($CDCl_3$, 101 MHz) 143.7, 135.2, 129.9, 127.2, 120.2, 118.3, 46.8, 29.5, 26.4, 24.0, 21.7, 18.8, 17.6.

LRMS [$M^{+\cdot}$] 277.0.

Table S15. Summary of reactions leading to cyclopropanes **S13**.

	**Catalyst**	**Yield / %**	***er***
1	$(Ph_3P)AuCl$	76	50 : 50
2	[Au((*R*$_{mp}$)-**6**)(Cl)]	37	51 : 49
3	(*R*)-DTBM-SEGPHOS®$(AuCl)_2$	20	50 : 50

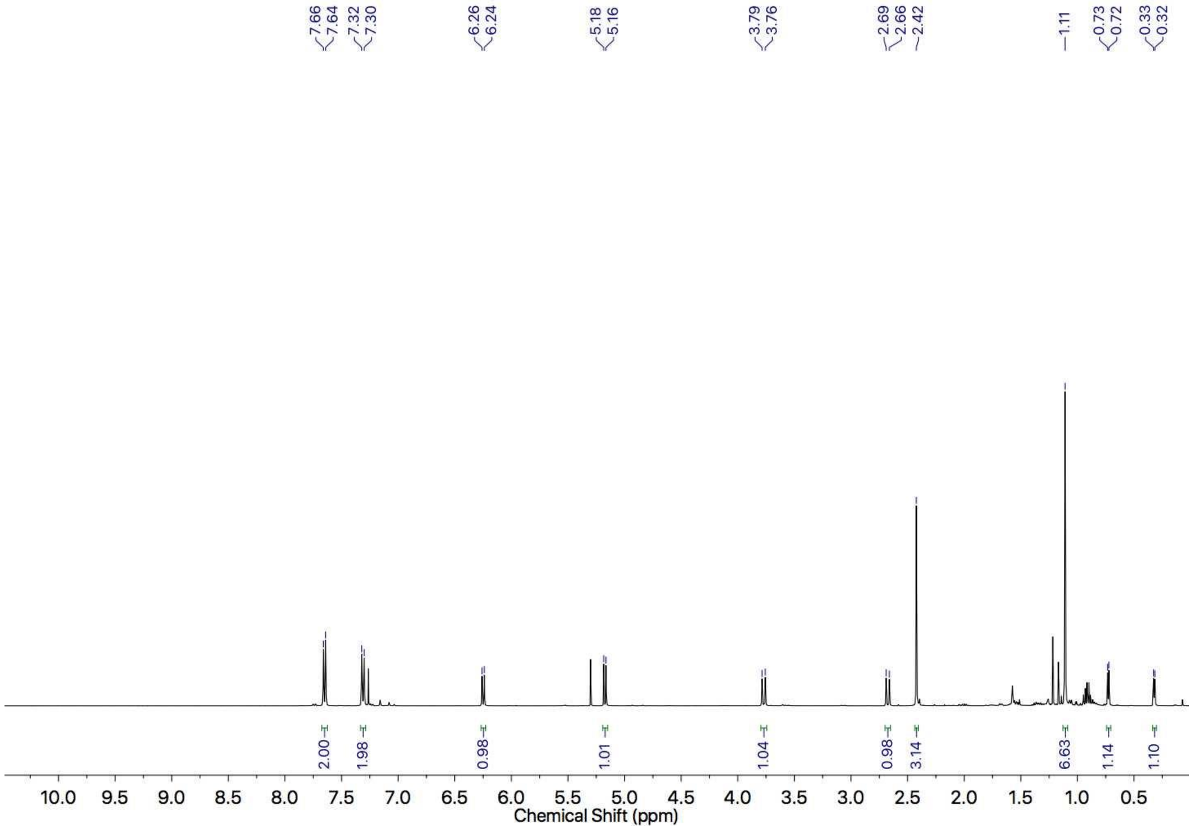

Figure S172: ^{1}H NMR of **S13** ($CDCl_3$, 400 MHz).

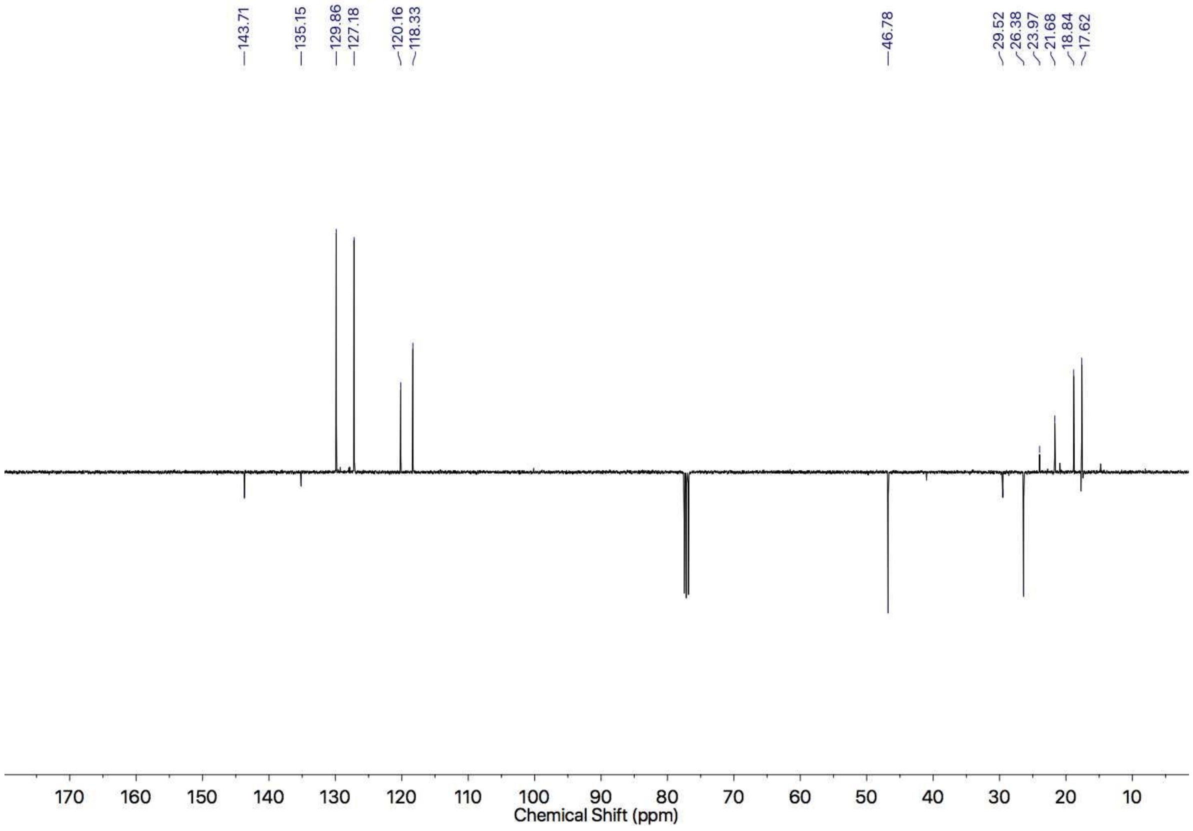

Figure S173: JMOD NMR of **S13** ($CDCl_3$, 101 MHz).

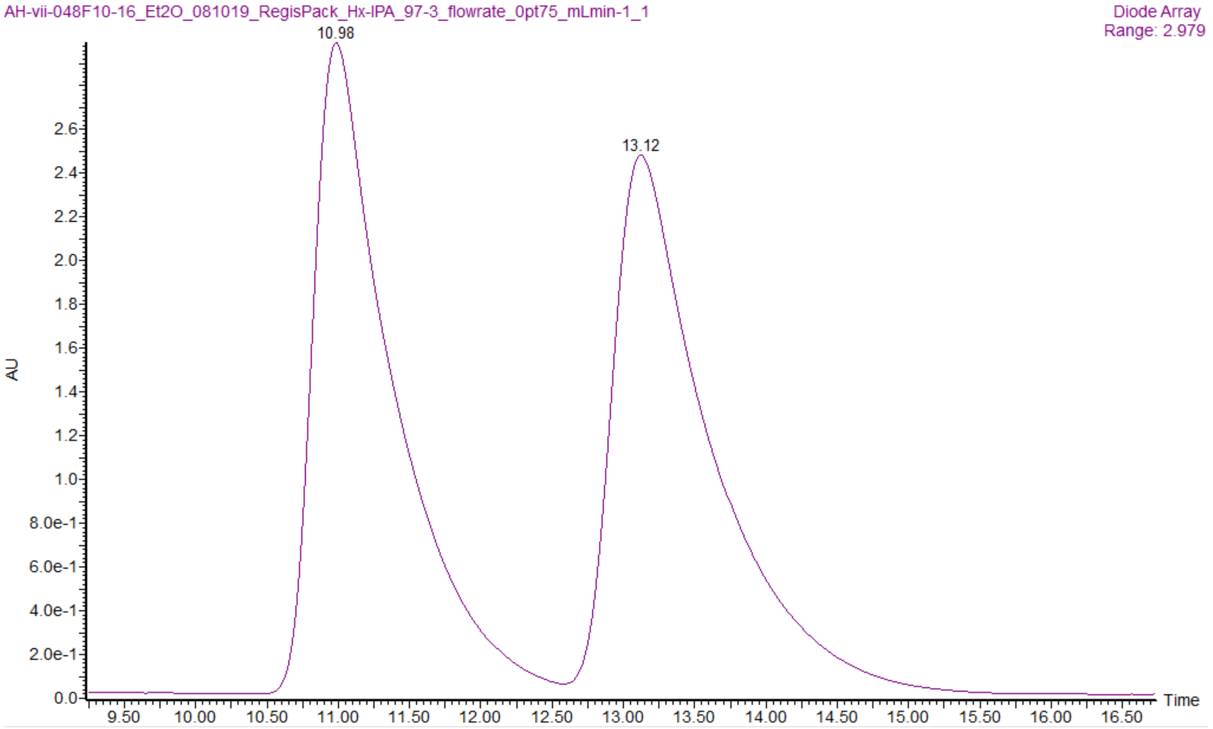

Figure S174: Entry 1. Chiral Stationary Phase HPLC (RegisPack, n-hexane-isopropanol 97 : 3, 303 K, load Et_2O, flowrate 0.75 $mLmin^{-1}$) of 50 : 50 *er*. Retention times (min): 11.0, 13.1.

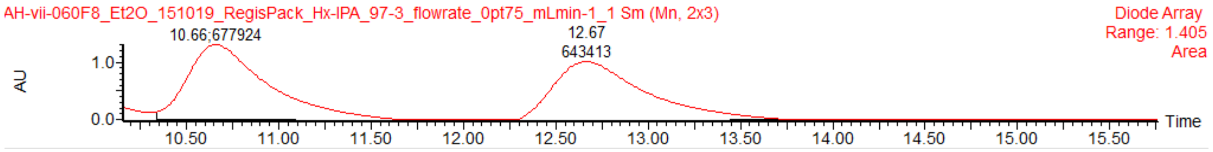

Figure S175: Entry 2. Chiral Stationary Phase HPLC (RegisPack, n-hexane-isopropanol 97 : 3, 303 K, load Et_2O, flowrate 0.75 $mLmin^{-1}$) of 51 : 49 *er*. Retention times (min): 10.7, 12.7.

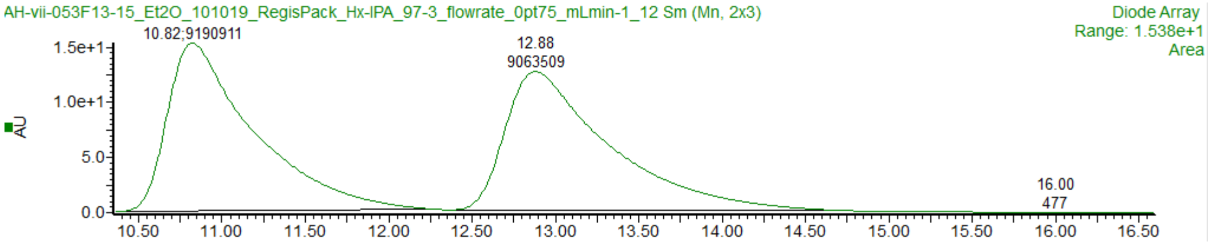

Figure S176: Entry 3. Chiral Stationary Phase HPLC (RegisPack, n-hexane-isopropanol 97 : 3, 303 K, load Et_2O, flowrate 0.75 $mLmin^{-1}$) of 50 : 50 *er*. Retention times (min): 10.8, 12.9.

Intramolecular Hydroamination of Allenes

Compound S14 was synthesised according to a literature route.[17]

Compound **S15**

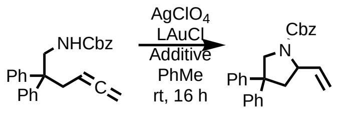

General Procedure: A CEM MW vial was charged with $AgClO_4$ (0.9 mg, 4.5 µmol, 0.05 eq.), LAuCl (4.5 µmol, 0.05 eq.) and in the case of [Au((R_{mp})-**6**)(Cl)], $[Cu(MeCN)_4]PF_6$ (1.7 mg, 4.5 µmol, 0.05 eq.). The flask was purged with N_2, and allene **S14** (34.7 mg, 0.091 mmol, 1.0 eq.) was added in degassed anhydrous PhMe (0.60 mL, 0.15 M) and stirred at rt overnight. The mixture was concentrated *in vacuo*. The residue was purified by column chromatography (SiO_2, pet-Et_2O 0→25%) yielding a colourless oil. Spectra match literature.[17]

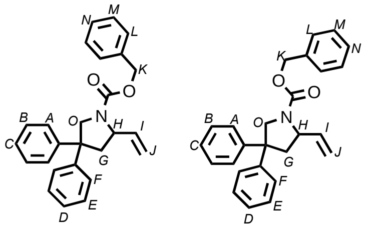

S15

A mixture of carbamate rotamers was observed. δ_H ($CDCl_3$, 400 MHz) 7.48-7.07 (15H, m), [5.78 (td, J = 10.5, 7.1), 5.77 (td, J = 10.5, 7.1), 1:1, 1H], 5.33-5.06 (4H, m), [4.76 (dd, J = 11.6, 1.9), 4.60 (dd, J = 11.5, 1.6) 1:1, 1H], [4.17 (dd, J 16.0, 7.4), 4.11 (dd, J 15.6, 7.3), 1:1, 1H], [3.72 (d, J = 15.6 Hz), 3.71 (d, J = 15.6), 1:1, 1H], 2.88-2.81 (1H, m), [2.47 (dd, J = 12.5, 9.8 Hz), 2.42 (dd, J = 12.5, 9.8 Hz), 1:1, 1 H].

δ_C ($CDCl_3$, 101 MHz) 155.4, 154.8, 145.5, 144.9, 139.3, 138.6, 137.0, 136.8, 128.8, 128.7, 128.4, 128.3, 128.2, 127.8, 127.7, 126.9, 126.7, 126.6, 115.8, 115.2, 67.0, 59.6, 59.1, 56.3, 53.2, 52.8, 45.8, 44.7.

LRMS [M + H^+] m/z 384.3286 (calc. $C_{26}H_{26}N_1O_2$ m/z 384.1964).

Table S16. Summary of reactions leading to cyclopropanes **S15**.

	Catalyst	Yield / %	*er*
1	$(Ph_3P)AuCl$	5	50 : 50
2	[Au((*R*$_{mp}$)-**6**)(Cl)]	51	50 : 50
3	(*R*)-DTBM-SEGPHOS®$(AuCl)_2$	38	67 : 33

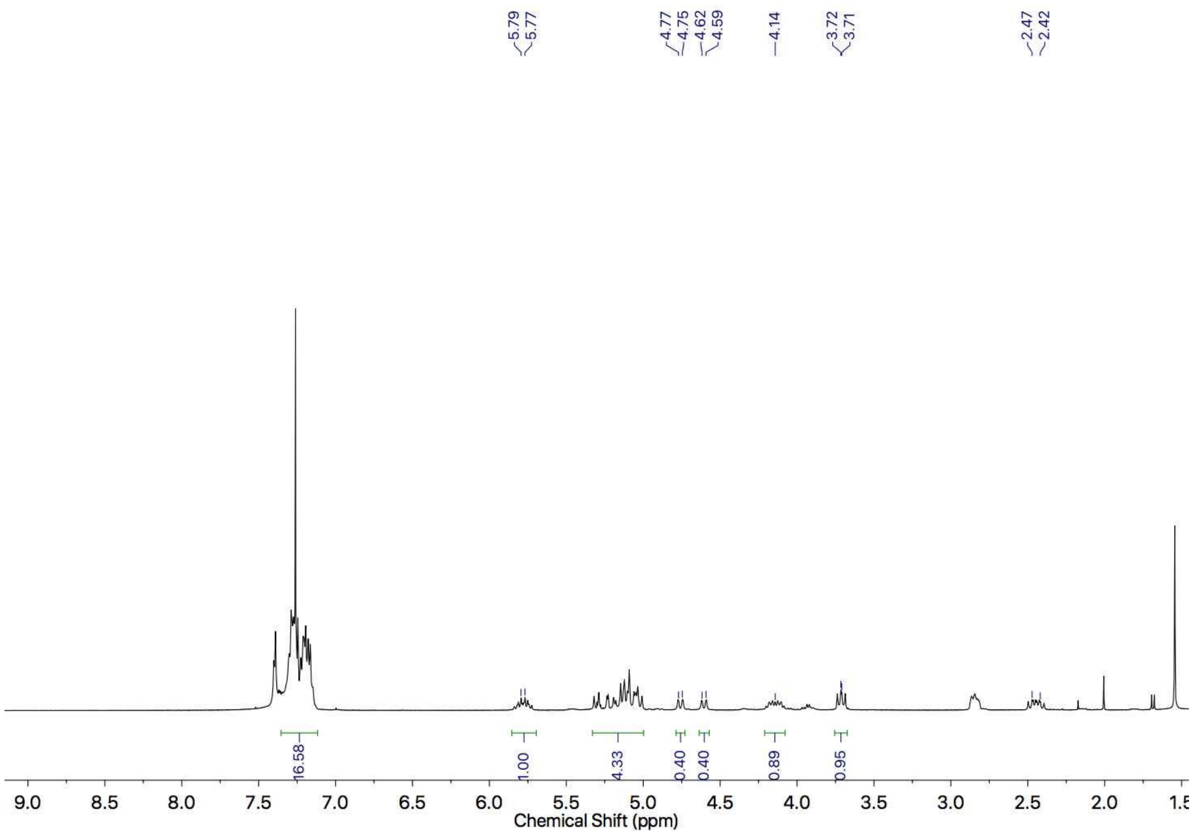

Figure S177: ^{1}H NMR of **S15** ($CDCl_3$, 400 MHz).

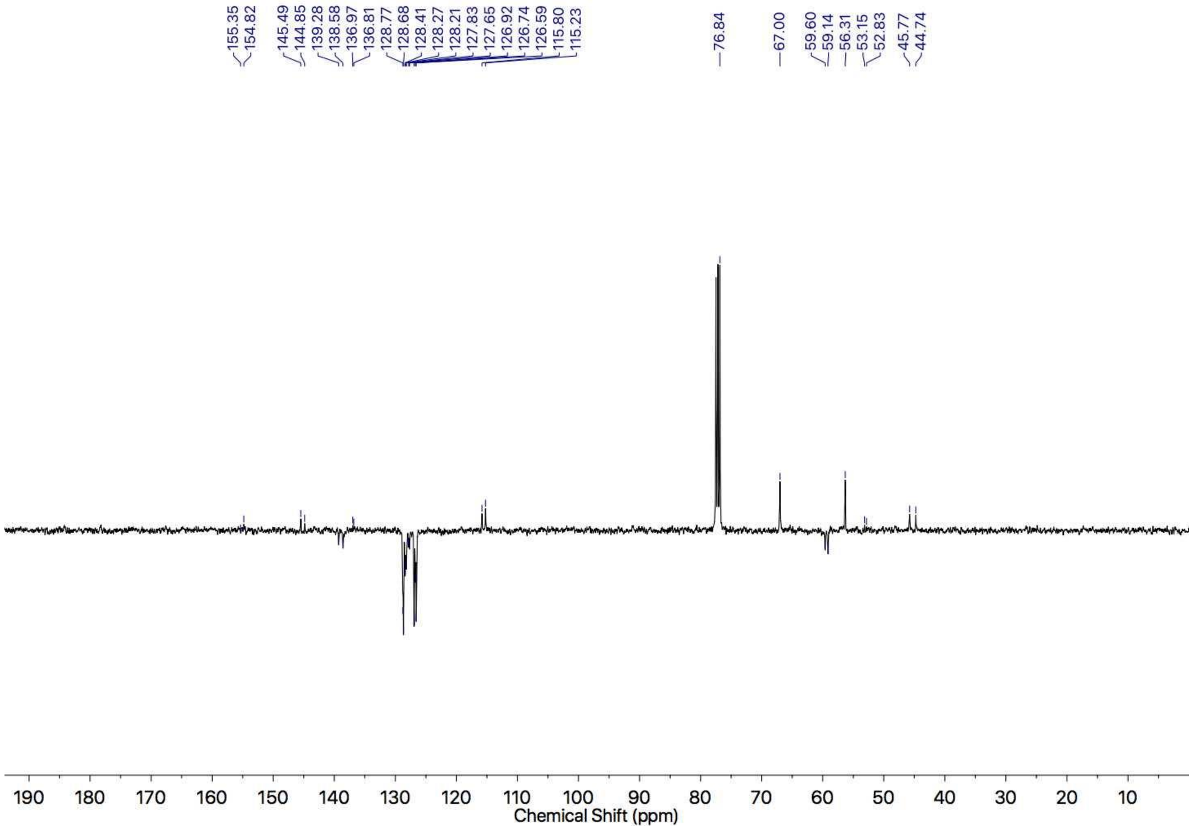

Figure S178: JMOD NMR of **S15** ($CDCl_3$, 101 MHz).

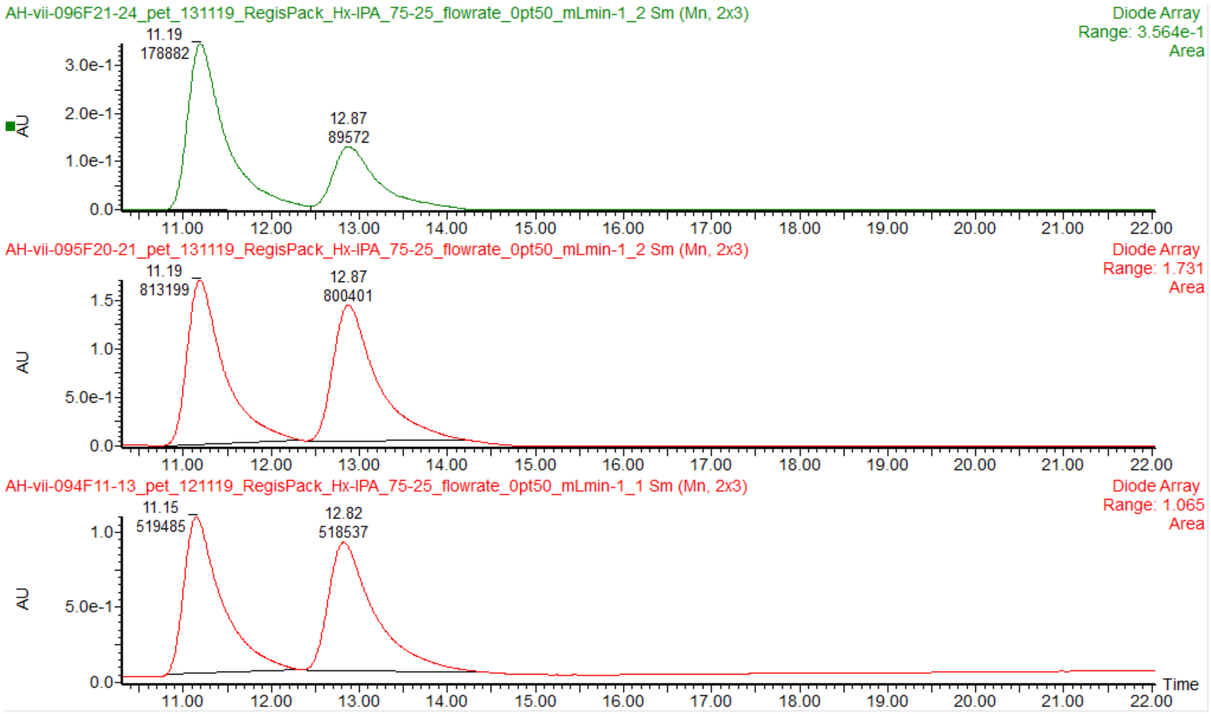

Figure S179: Chiral Stationary Phase HPLC (RegisPack, n-hexane-isopropanol 75 : 25, 303 K, load pet, flowrate 0.50 mLmin^{-1}). Retention times (min): 11.2, 12.9. Top (entry 3) *er* 67 : 33, Middle (entry 2) *er* 50 : 50, Bottom (entry 1) *er* 50 : 50.

7. Molecular Modelling

Models of the reaction of ester **7** and styrene (**8**) mediated by [Au(PPh_3)(Cl)]

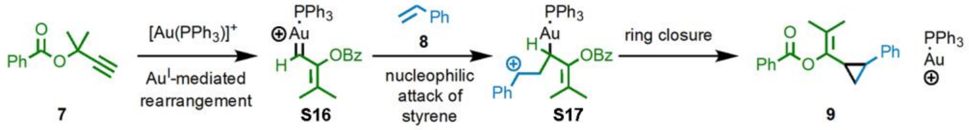

Figure S180. Proposed pathway for the reaction of **7** and **8** mediated by [Au(PPh_3)(Cl)]

We initially investigated the reaction of ester **7** with styrene (**8**) mediated by [Au(PPh_3)(Cl)] (**Figure S180**) to determine a reasonable transition state for the same reaction mediated by rotaxane complex [Au(**6**)(Cl)]. Models of the transition states for the reaction of presumed carbene intermediate **S16** with styrene were constructed based on transition state structures reported by Echavarren and co-workers in a related reaction by modifying the substituents of the phosphine and carbene.[18] Scanning of the distance between the carbenic carbon and the β-position of styrene, followed by transition state optimisation of the structures that corresponded to energetic maxima on the scanned coordinate yielded four transition states **TSI** (**Figure S181**), two leading to *cis*-**9**, two leading to *trans*-**9**, of which *cis*-**TSIb** was found to be more stable by 10.3 kJmol^{-1} than the nearest alternative pathway. All transition states were determined to be first order saddle points with a single imaginary frequency mode.

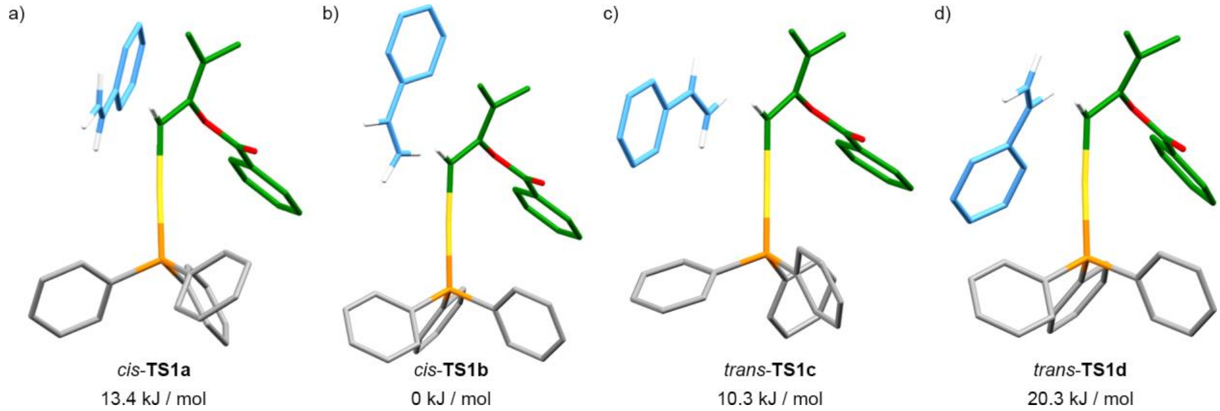

Figure S181. Transition states and their relative energies for the reaction **S16→S17** determined by DFT (CAM-B3LYP, 6-31G*(C,H,O,P)/SDD(Au)). For structures see "Model_AuPPh3Cl_TS1.cif" submitted as electronic supporting information.

It should be noted that, in accordance with Echavarren and co-workers' previous report, in all cases IRC calculations indicate that transition states **TSI** correspond to a stepwise mechanism as shown in **Figure S180**, in which the styrene acts as a nucleophile on the electrophilic carbene to generate α-aryl cations **S17**, that then undergo ring closure with extremely low barrier to give the observed cyclopropane product. Specifically, in the case of *cis*-**TSIb** (**Figure S181**), the reaction of

S16 to produce **S17** was found to proceed with a barrier of 3.0 kJmol^{-1} and to be extremely favourable (-45.2 kJmol^{-1}). Subsequent ring closure was essentially barrierless, passing through *cis*-**TSIIb** (first order saddle point, single imaginary frequency mode) to generate cyclopropane *cis*-**9** initially with the Au^{I} catalyst associated with the carbonyl of the ester. (Note: although **S17** and **TSIIb** were found to be minima and maxima respectively on the potential energy surface by frequency analysis, their structures are related by an extremely small atomic reorganisation and were energetically indistinguishable at this level of theory).

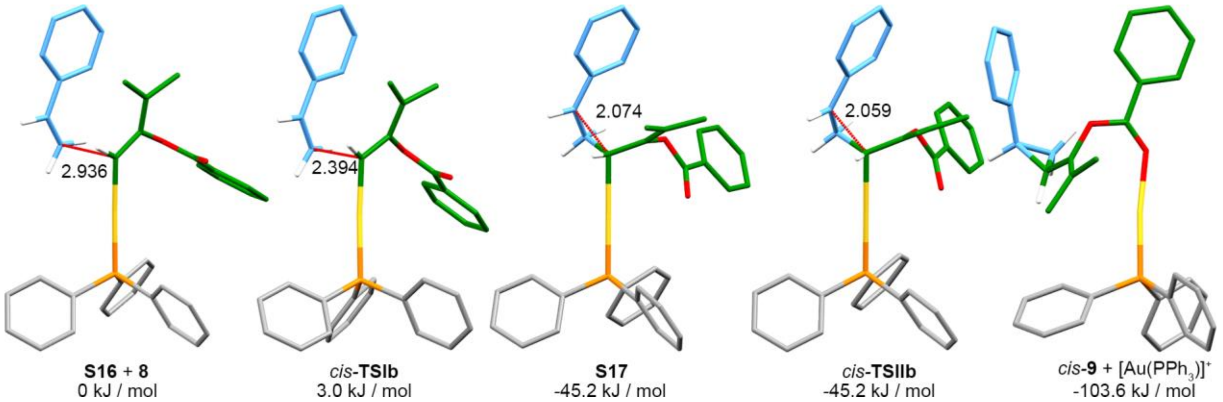

Figure S182. Intermediates and transition states and their relative energies for the reaction from **S16** → cyclopropane **9** determined by DFT (CAM-B3LYP, 6-31G*(C,H,O,P)/SDD(Au)). For structures see "Model_DFT_AuPPh3Cl_full_reaction.cif" submitted as electronic supporting information.

Diastereomeric transition states for the reaction of 7 with 8 mediated by [Au(6)(Cl)]

A model of the transition state for the reaction of **7** with **8** mediated by [Au(**6**)(Cl)] was constructed by joining *cis*-**TSIb** with a model of [Cu((*R*$_{mp}$)-**5**]$^{+}$ by replacing one of the Ph substituents of *cis*-**TSIb** (highlighted) with the rotaxane framework with the highlighted atoms removed (**Figure S183**). The diastereomeric model was generated by using the same rotaxane framework with the enantiomer of *cis*-**TSIb**.

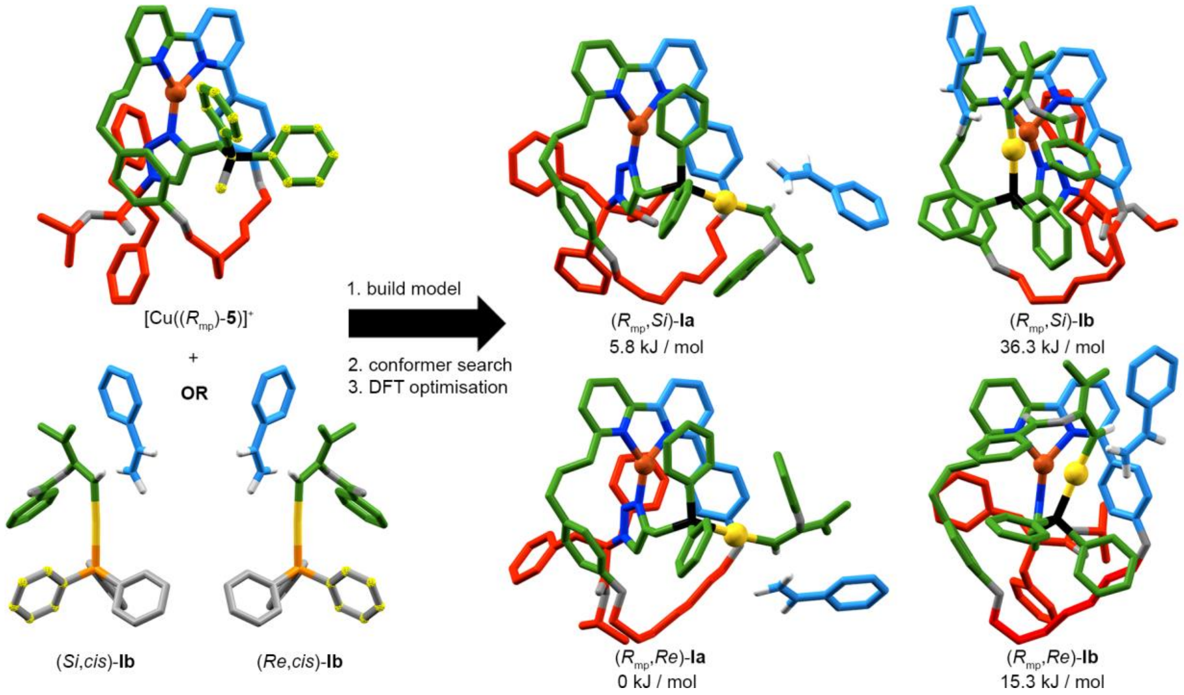

Figure S183. Procedure for the construction of candidate conformations of the transition state for the reaction mediated by [Au(**6**)(Cl)], the four lowest energy conformations found and their energies as evaluated using DFT (CAM-B3LYP, 6-31G(C,H,O,P,N)/SDD(Au,Cu)). For structures of **Ia** and **Ib** see "Model_Rmp_confs_I.cif" submitted as electronic supporting information.

A conformational search was performed using these models of (R_{mp},*Re*)-**Ia** and (R_{mp},*Si*)-**Ia**, where Re and Si refer to the face of the carbene approached by styrene (Note: (R_{mp},*Si*)-**Ia** gives rise to the observed major product (1*S*,2*R*)-**9**). The transition state fragment (carbene unit, styrene moiety, Au and P atoms) was frozen, the Cu ion was restricted to a trigonal planar configuration, and a conformational search was performed (Spartan '10, MMF) by rotating triazole-N^1-C, and the single bonds of the C-C-P fragment in 60 degree increments followed by re-optimisation using the same model with no restriction on these bond angles. This search was repeated several times starting from different initial states and yielded reproducible results; for both diastereomeric structures, two families of low energy conformations were identified, one in which the P-Au bond was projected parallel or nearly parallel to the triazole-N-Cu bond and the other in which the P-Au bond is projected towards the same O substituent of the macrocycle. The two lowest energy conformations included one of each family and were predicted to be more stable than the next lowest energy conformation by > 10 kJ / mol. These conformations were then reoptimized with the transition state fragment frozen using DFT (CAM-B3LYP, 631-G(C,H,O,P,N)/SDD(Au, Cu)) with the result that conformers **Ia** were found to be more stable by >15 kJ / mol (**Figure S183**).

Conformers (R_{mp},*Re*)-**Ia** and (R_{mp},*Si*)-**Ia** were taken forward for transition state optimisations and

the results of these calculations are summarised in **Table S17** A transition state search using an ONIOM method was carried out initially with the transition state fragment (including the Ph substituents of the P atom), in the high level (CAM-B3LYP, 631-G(C,H,O,P)/SDD(Au)) and the rest of the molecule in the low level (UFF) and frozen, except for the methylene linking the different fragments. The outputs of the ONIOM calculations were then subjected to a transition state search using DFT (CAM-B3LYP, 631-G(C,H,O,P,N)/SDD(Au,Cu)) with no frozen or restricted atoms. This yielded two diastereomeric transition state structures **TS1** with a single imaginary mode. These structures were re-optimized in the gas phase (entry 5, CAM-B3LYP, 631-G*(C,H,O,P,N)/SDD(Au,Cu)) and in $CHCl_3$ (entry 4, PCM($CHCl_3$), CAM-B3LYP, 631-G(C,H,O,P,N)/SDD(Au,Cu)), the latter of which was subjected to a single point energy calculation (entry 6, $CHCl_3$, CAM-B3LYP, 631-G*(C,H,O,P,N)/SDD(Au,Cu)). Attempts to directly locate a transition state with these latter parameters proved prohibitively computationally expensive. The structures of Re/Si **TS1-Oniom** (**Table S17**, entry 2), **TS1-631G** (entry 3), **TS1-631GCHCl3** (entry 4) and **TS1-631GG** (entry 5) are provided as supporting information (.xyz).

Examining the computed relative energies of **TS1**, the outcome is strongly dependent on the method used. When evaluated using an Oniom method (entry 2), the calculations predict a very large preference for the (1*S*,2*R*)-**9** major product observed experimentally. Optimisation of the entire structure using DFT with a 6-31G basis set in the gas phase (entry 3) predicts the opposite enantiomer. When solvent is included ($CHCl_3$, entry 4), or a larger basis set employed in the gas phase (entry 5) the correct product is predicted, both with reasonable agreement to the experimental value. Re-subjecting the output of entry 4 to a single point energy calculation with a larger basis set (6-31G*, entry 6) results in an even higher predicted preference for the experimentally observed major product. The results in **Table S17** suggest that although the structures of **TS1** determined using DFT are useful from an illustrative point of view, the relative energies determined are not particularly reliable, especially given the relatively small difference (~3 kJ / mol) predicted from experiment.

Table S17. Comparison of the outcomes of different calculations for **TS1**

Entry	Method	Basis Set	Solvent	Imaginary modes (cm^{-1})	E[*Si* –*Re*] (kJ / mol)	(1*S*,2*R*)-9 *:* (1*R*,2*S*)-9
1	Experiment	-	$CHCl_3$	-	–3.0	79 : 21
2	Oniom (CAM-B3LYP:UFF)	6-31G/SDD(Au)	-	–175.19 (*Si*), –51.23 (*Re*)	–25.4	100 : 0
3	CAM-B3LYP	6-31G/SDD(Au,Cu)	-	–159.89 (*Si*), –118.84 (*Re*)	1.7	32 : 68
4	CAM-B3LYP	6-31G/SDD(Au,Cu)	$CHCl_3$	–176.85 (*Si*), –175.54 (*Re*)	–2.3	74 : 26
5	CAM-B3LYP	6-31G*/SDD(Au,Cu)	-	–149.80 (*Si*), –120.42 (*Re*)	–4.9	90 : 10
6	CAM-B3LYP (single point)	6-31G*/SDD(Au,Cu)	$CHCl_3$	-	–6.4	94 : 6

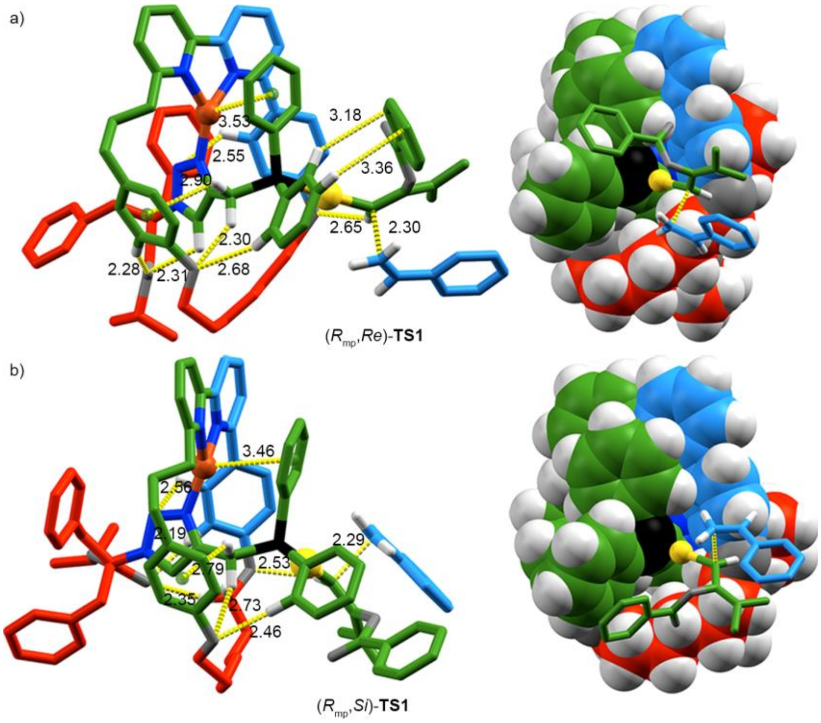

Figure S184. Transition states **TS1** determined for the reaction mediated by [Au(**6**)(Cl)] using DFT (CAM-B3LYP, 6-31G(C,H,O,P,N)/SDD(Au,Cu), PCM($CHCl_3$)) with selected intercomponent interactions and distances indicated. For structures see “Model_Table_S17_structures.cif” submitted as electronic supporting information.

Transition states for the formation of cyclopropanes 13, 15 and 16 mediated by [Au(6)(Cl)]

Transition states for the reactions leading to cyclopropanes **13**, **15** and **16** were constructed by modification of diastereomeric transition states **TS1** (**Table S17**, entry 4), followed by repeating the transition state search, first in the gas phase then in $CHCl_3$ (PCM). The structures obtained are shown in **Figure S185** and the results are summarized in **Table S18**.

Table S18. Comparison of the outcomes of different calculations of **TS1**

Entry	Method	Basis Set	Solvent	Imaginary modes (cm^{-1})	ΔE[*Si* –*Re*] (kJ / mol)	(1*S*,2*R*)-*9* : (1*R*,2*S*)-*9*
			Cyclopropanes **13**			
1	Experiment	-	$CHCl_3$	-	0.4	55 : 45
2	CAM-B3LYP	6-31G/SDD(Au,Cu)	$CHCl_3$	–190.27 (*Si*), –175.54 (*Re*)	3.1	16 : 84
			Cyclopropanes **15**			
3	Experiment	-	$CHCl_3$	-	–2.2	73 : 27
4	CAM-B3LYP	6-31G/SDD(Au,Cu)	$CHCl_3$	–177.19 (*Si*), –160.62 (*Re*)	–3.1	80 : 20
			Cyclopropanes **16**			
5	Experiment	-	$CHCl_3$	-	–2.0	70 : 30
6	CAM-B3LYP	6-31G/SDD(Au,Cu)	$CHCl_3$	–194.11 (*Si*), –170.46 (*Re*)	–3.0	79 : 21

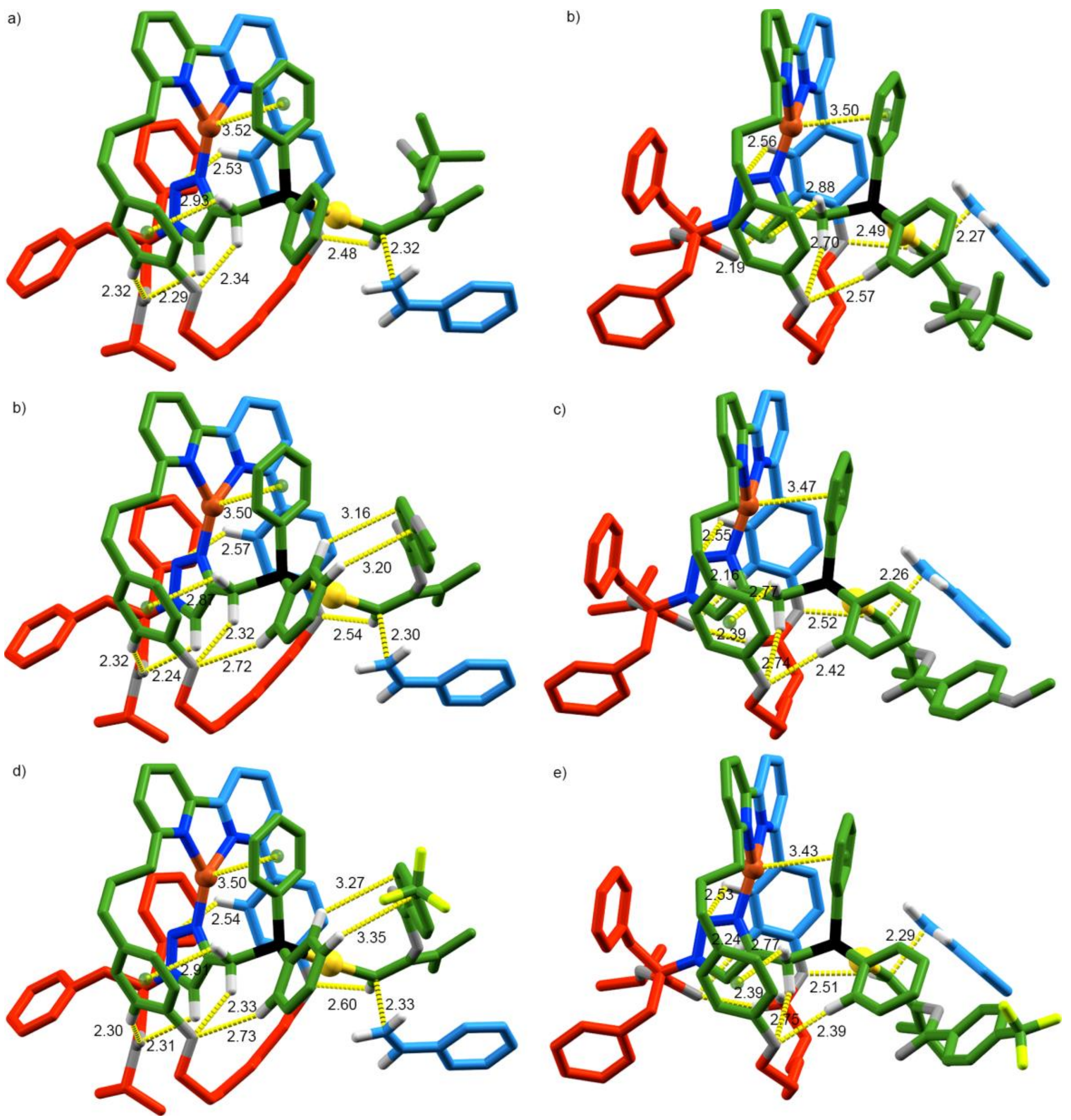

Figure S185. Transition states determined (DFT (CAM-B3LYP, 6-31G(C,H,O,P,N)/SDD(Au,Cu), PCM($CHCl_3$)) for reactions leading to cyclopropanes a) (1*R*,2*S*)-**13**, b) (1*S*,2*R*)-**13**, c) (1*R*,2*S*)-**15**, d) (1*S*,2*R*)-**15**, e) (1*R*,2*S*)-**16**, f) (1*S*,2*R*)-**16**. with selected intercomponent interactions and distances indicated. For structures see "Model_Table_S18_structures.cif" submitted as electronic supporting information.

The results obtained for cyclopropanes **15** and **16** are in reasonable agreement with experiment. The fact that modelling does not predict the correct ranking of *ee* for **9**, **15**, and **16**, is not surprising, given the small difference between the experimental values (~1 kJ / mol). The large discrepancy between the experimental and modelled data for cyclopropanes **13** may indicate that, in the case of pivolyl esters, different/additional conformations of the catalyst-substrate complex may be important in product formation. Indeed, given the potential for other catalytically relevant

conformations, further detailed studies are required to assess their relevance and provide a more detailed framework for discussing the selectivity induced by the mechanically planar chiral stereogenic unit.

8. References

1. Pigorsch, A. & Kockerling, M. (2016). The Crystallization of Extended Niobium-Cluster Framework Compounds: A Novel Approach Using Ionic Liquids. *Cryst.Growth Des.* **16**, 4240–4246.

2. Schuster, E. M., Botoshansky, M. & Gandelman, M. (2008). Pincer click ligands. *Angew. Chem. Int. Ed.* **47**, 4555–4558.

3. Jinks, M. A., de Juan, A., Denis, M., Fletcher, C. J., Galli, M., Jamieson, E. M. G., Modicom, F., Zhang, Z. & Goldup, S. M. (2018). Stereoselective Synthesis of Mechanically Planar Chiral Rotaxanes. *Angew. Chem. Int. Ed.* **57**, 14806–14810.

4. Lewis, J. E. M., Bordoli, R. J., Denis, M., Fletcher, C. J., Galli, M., Neal, E. A., Rochette, E. M. & Goldup, S. M. (2016). High yielding synthesis of 2,2′-bipyridine macrocycles, versatile intermediates in the synthesis of rotaxanes. *Chem. Sci.* **7**, 3154–3161.

5. Jamieson, E. M. G., Modicom, F. & Goldup, S. M. (2018). Chirality in rotaxanes and catenanes. *Chem. Soc. Rev.* **47**, 5266–5311.

6. Johansson, M. J., Gorin, D. J., Staben, S. T. & Toste, F. D. (2005). Gold(I)-Catalyzed Stereoselective Olefin Cyclopropanation. *J. Am. Chem. Soc.* **127**, 18002–18003.

7. Sheldrick, G. M. (2015). Crystal structure refinement with SHELXL. *Acta Crystallogr. Sect. C Struct. Chem.* **71**, 3–8.

8. Wu, H., Guo, W., Daniel, S., Li, Y., Liu, C. & Zeng, Z. (2018). Fluoride-Catalyzed Esterification of Amides. *Chem. E* **24**, 3444–3447.

9. Pagar, V. V., Jadhav, A. M. & Liu, R. (2011). Gold-Catalyzed Formal [3 + 3] and [4 + 2] Cycloaddition Reactions of Nitrosobenzenes with Alkenylgold Carbenoids. *J. Am. Chem. Soc* **133**, 20728–20731.

10. Schiessl, J., Stein, P. M., Stirn, J., Emler, K., Rudolph, M., Rominger, F. & Hashmi, A. S. K. (2019). Strategic Approach on N -Oxides in Gold Catalysis – A Case Study. *Adv. Synth. Catal.* **361**, 725–738.

11. Although compound **S4** has previously been reported (Journal of Organic Chemistry USSR, 1972, vol 8, 884), we were not able to access this article and so characterised the compound in full.

12. Soderberg, B. C., Neil, S. N. O., Chisnell, A. C. & Liu, J. (2000). A [3 . 3] Sigmatropic Rearrangement of a , b - Unsaturated Fischer Chromium Carbenes : Synthesis of Alkynol and Dienol Esters. *Tetrahedron* **56**, 5037–5044.

13. Zuccarello, G., Mayans, J. G., Escofet, I., Scharnagel, D., Kirillova, M. S., Pérez-Jimeno, A. H., Calleja, P., Boothe, J. R. & Echavarren, A. M. (2019). Enantioselective Folding of Enynes by Gold(I) Catalysts with a Remote C 2 -Chiral Element. *J. Am. Chem. Soc.* **141**, 11858–11863.

14. Ref. 13 and Liang, B., Dai, M., Chen, J. & Yang, Z. (2005). Copper-free sonogashira coupling reaction with PdCl2 in water under aerobic conditions. *J. Org. Chem.* **70**, 391–393. Sanjuán, A. M., Martínez, A., García-García, P., Fernández-Rodríguez, M. A. & Sanz, R. (2013). Gold(I)-catalyzed 6-endo hydroxycyclization of 7-substituted-1,6-enynes. *Beilstein J. Org. Chem.* **9**, 2242–2249.

15. Benedetti, E., Simonneau, A., Hours, A., Amouri, H., Penoni, A., Palmisano, G., Malacria, M., Goddard, J. P. & Fensterbank, L. (2011). (Pentamethylcyclopentadienyl)iridium dichloride dimer {[IrCp*Cl 2]2}: A novel efficient catalyst for the cycloisomerizations of homopropargylic diols and N-tethered enynes. *Adv. Synth. Catal.* **353**, 1908–1912.

16. Shibata, T., Kobayashi, Y., Maekawa, S., Toshida, N. & Takagi, K. (2005). Iridium-catalyzed enantioselective cycloisomerization of nitrogen-bridged 1,6-enynes to 3-azabicylo[4.1.0]heptenes. *Tetrahedron* **61**, 9018–9024.

17. Zhang, Z., Bender, C. F. & Widenhoefer, R. A. (2007). Gold(I)-catalyzed enantioselective hydroamination of N-allenyl carbamates. *Org. Lett.* **9**, 2887–2889.

18. Pérez-Galán, P., Herrero-Gómez, E., Hog, D. T., Martin, N. J. A., Maseras, F. & Echavarren, A. M. (2011). Mechanism of the gold-catalyzed cyclopropanation of alkenes with 1,6-enynes. *Chem. Sci.* **2**, 141–149.
