# Supplementary material for: Synthesis of a Mechanically Planar Chiral Rotaxane Ligand for Enantioselective Catalysis
Source: Chem. 2020 Apr 9;6(4):994–1006. doi: 10.1016/j.chempr.2020.02.006 (PMC7153771; doi:10.1016/j.chempr.2020.02.006)
Supplement: Document S2. Article plus Supplemental Information [file mmc7.pdf]

Chem

Cell Press

Article

Synthesis of a Mechanically Planar Chiral Rotaxane Ligand for Enantioselective Catalysis

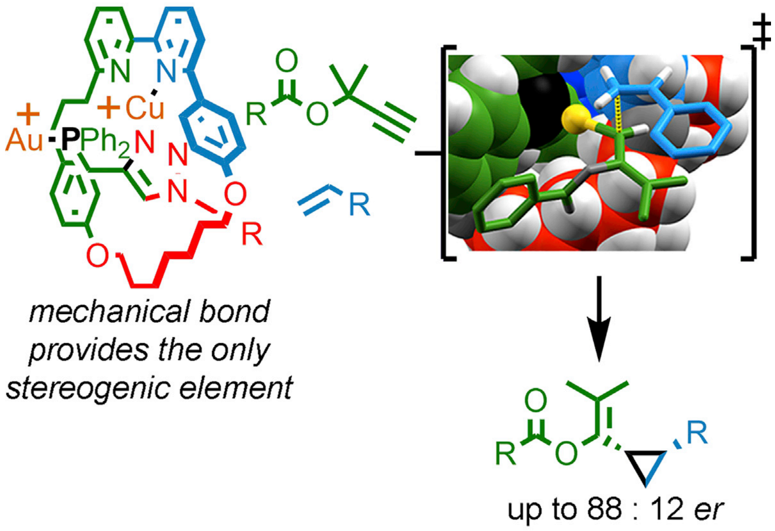

We report an enantioselective catalyst based on a "mechanically chiral" rotaxane. Catalysis with chiral molecules is extremely important in modern chemistry because it is one of the most efficient ways to make chiral molecules for applications in many areas. Our results demonstrate, for the first time, that mechanically chiral molecules are a promising and underexplored platform for generating such catalysts. We achieve enantioselectivities for the Au^{I}-catalyzed Ohe-Uemura cyclopropanation of benzoate esters comparable to previously reported covalent catalysts.

Andrew W. Heard, Stephen M. Goldup

s.goldup@soton.ac.uk

HIGHLIGHTS

We synthesized a mechanically planar chiral rotaxane for catalytically active gold ions

We demonstrate enantioselective catalysis with a mechanically planar chiral rotaxane

Our results suggest that mechanical stereochemistry has untapped potential in enantioselective catalysis

Heard & Goldup, Chem 6, 994–1006
April 9, 2020 © 2020 The Author(s). Published by Elsevier Inc.
https://doi.org/10.1016/j.chempr.2020.02.006

Chem

CellPress

Article

Synthesis of a Mechanically Planar Chiral Rotaxane Ligand for Enantioselective Catalysis

Andrew W. Heard[1] and Stephen M. Goldup[1,2,*]

SUMMARY

Rotaxanes are interlocked molecules in which a molecular ring is trapped on a dumbbell-shaped axle because of its inability to escape over the bulky end groups, resulting in a so-called mechanical bond. Interlocked molecules have mainly been studied as components of molecular machines, but the crowded, flexible environment created by threading one molecule through another has also been explored in catalysis and sensing. However, so far, the applications of one of the most intriguing properties of interlocked molecules, their ability to display stereogenic units that do not rely on the stereochemistry of their covalent subunits, termed "mechanical chirality," have yet to be properly explored, and prototypical demonstration of the applications of mechanically chiral rotaxanes remain scarce. Here, we describe a mechanically planar chiral rotaxane-based Au complex that mediates a cyclopropanation reaction with stereoselectivities that are comparable with the best conventional covalent catalyst reported for this reaction.

The Bigger Picture

Molecules that exist in non-identical mirror image forms are referred to as chiral. Chirality can arise because of various molecular features in which atoms are held in fixed orientations that are themselves chiral, and typically such "stereogenic units" are maintained by direct bonds between atoms. Molecular chirality can also arise by threading a dumbbell-shaped molecule through a molecular ring to generate a rotaxane. However, these molecules have not been investigated significantly because until recently they were extremely hard to make in one mirror image form. Here, we report the first example of a catalyst based on such a "mechanically chiral" rotaxane. Catalysis with chiral molecules is extremely important in modern chemistry because it is one of the most efficient ways to make chiral molecules for applications in healthcare and other areas. Our results demonstrate that mechanically chiral molecules are a promising and underexplored platform for generating such catalysts.

INTRODUCTION

Interlocked molecules such as rotaxanes, in which a dumbbell-shaped axle is threaded through a macrocycle, and catenanes, in which two or more macrocycles are held together in a manner akin to links in a chain,[1] are most commonly investigated as components of molecular machines,[2] building on the pioneering work of Stoddart and Sauvage, who were awarded the Nobel Prize for their efforts in 2016.[3–5] In contrast, one of the most intriguing structural properties of interlocked molecules, their ability to display enantiotopic stereogenic elements that do not rely on covalent stereochemistry,[6] has received much less attention, despite the possibility of such enantiomerism being discussed early in the development of the field.[7,8] Such "mechanical" stereogenic units can arise because of desymmetrization of one of the covalent subunits by the relative position of the other (co-conformational chirality), the combination of subunits with appropriate symmetry properties (conditional mechanical chirality), or the unconditional topology of the mechanical bond itself (Figure 1A).[6,9,10]

The relative paucity of even prototypical applications of mechanically chiral molecules is at least in part because enantiopure samples were historically hard to synthesize, with the pioneering work carried out by Vögtle, Okamoto, and Sauvage,[15,16] requiring the use of chiral stationary phase high-performance liquid chromatography (HPLC) to separate the enantiomeric products from a racemic mixture. Using this approach, Vogtle and co-workers showed that mechanically planar chiral rotaxanes and topologically chiral catenanes displayed strong electronic circular

This is an open access article under the CC BY license (http://creativecommons.org/licenses/by/4.0/).

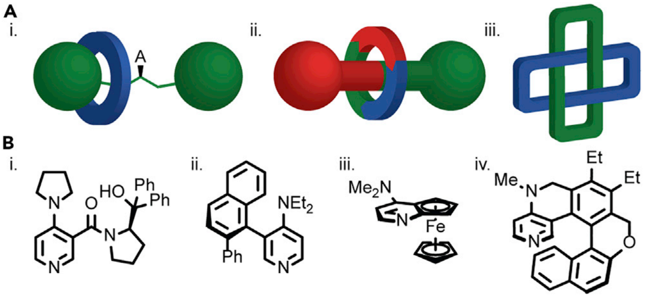

Figure 1. Different Forms of Chirality in Mechanically and Covalently Bonded Molecules

(A) Examples of (i) co-conformational, (ii) conditional mechanical, and (iii) unconditional topological stereogenic units.

(B) Examples of covalently bonded chiral acyl transfer catalysts based on (i) point,[11] (ii) axial,[12] (iii) planar,[13] and (iv) helical[14] stereogenic units.

dichroism (CD),[15] Hirose and co-workers disclosed a mechanically planar chiral rotaxane that selectively binds and senses the enantiomers of small chiral molecules,[17] and Takata and co-workers demonstrated that the mechanically planar chiral stereogenic unit can direct the helical twist of a poly-diacetylene material.[18] More recently, Saito and co-workers demonstrated the separation of co-conformationally mechanically planar chiral rotaxanes and used the link between the rate of racemization and co-conformational motion to determine the energy barrier for shuttling[19] and Credi and co-workers demonstrated a co-conformationally mechanically planar chiral molecule that shuttles between achiral and chiral states, the latter of which could be biased by the binding of a small chiral guest.[20]

However, of these unusual forms of stereochemistry, only co-conformational point chirality has been exploited in catalysis; in 2015, Leigh and co-workers demonstrated an enantioselective co-conformationally covalent point chiral organocatalyst (Figure 1Ai) that mediated enamine and iminium activation.[21,22] (The same authors have developed molecular ratchets and motors based on this stereogenic element.[23,24]) In contrast, the full complement of covalent stereogenic units,[25] including point,[11] axial,[12] planar,[13] and helical[14] chirotopic elements[26] have been applied in the development of new scaffolds to mediate enantioselective processes (Figure 1B) since the Nobel Prize was awarded in 2001 to Noyori, Knowles, and Sharpless for their contributions to the development of enantioselective catalysis.[27–29] Indeed, recent work has aimed at expanding the mechanisms by which stereochemical information is transferred to the reaction space including the use of chiral counterions,[30] chiral-at-metal systems,[31] helical artificial[32] and natural[33,34] polymers, chiral solvents,[35] chiral capsules,[36] and other confined environments.[37]

Building on our recent effort to improve access to mechanically chiral molecules through the use of chiral derivatizing units[38,39] and auxiliaries,[40] here we demonstrate the first example of enantioselective catalysis with a mechanically planar chiral rotaxane, one of the simplest conditional mechanical stereogenic units, which arises when an achiral macrocycle with C_{nh} point group symmetry encircles an achiral axle with C_{nv} point group symmetry.[6,9,10] Our rotaxane catalyst, whose structure was not designed or optimized, displays enantioselectivities in an Au^{I}-mediated cyclopropanation reaction comparable to the best reported covalent catalyst.[41] Our results suggest that mechanical stereochemistry has untapped potential in the development of new enantioselective catalytic systems.

[1]School of Chemistry, University of Southampton, Highfield, Southampton SO17 1BJ, UK

[2]Lead Contact

*Correspondence: s.goldup@soton.ac.uk

https://doi.org/10.1016/j.chempr.2020.02.006

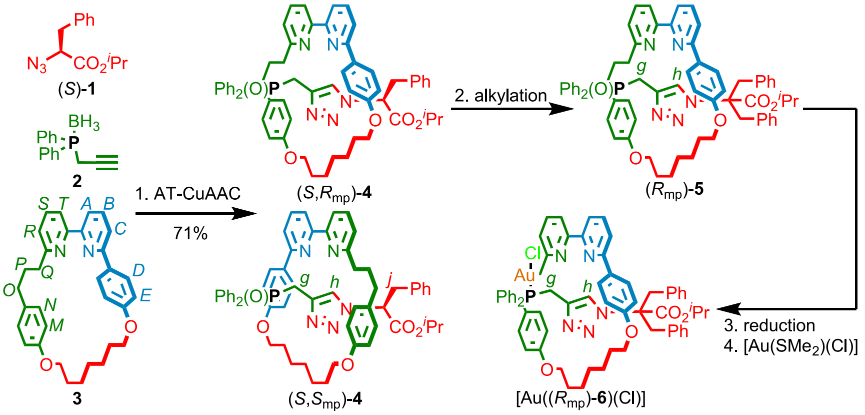

Scheme 1. Synthesis of Mechanically Planar Chiral Rotaxane Precatalysts

Reagents and conditions: (1) (i) $[Cu(MeCN)_4]PF_6$, ^{1}H-sponge, CH_2Cl_2, room temperature (RT), 8 h; (ii) KCN, MeOH-CH_2Cl_2 (1:1), RT, 30 min; (iii) H_2O_2 (35% w/w in H_2O), CH_2Cl_2, RT, 5 min. 72% combined yield over 3 steps prior to separation of diastereomers. (S,R_{mp})-**4**: 30%, 98% *ee*, >99: <1 *dr*; (S,S_{mp})-**4**: 24%, (S,S_{mp})-**4**-(R,S_{mp})-**4**-(S,R_{mp})-**4** = 98.4:1.0:0.6. (2) LiHMDS, tetrahydrofuran, −78°C then, BnI, −78°C to RT, 18 h. (R_{mp})-**5**: 81% (98% *ee*). (S_{mp})-**5** 63% (98% *ee*; data not shown, see Supplemental Information). (3) $HSiCl_3$, NEt_3, PhMe, CH_2Cl_2, 100°C, 3 days. (4) $(Me_2S)AuCl$, CH_2Cl_2, RT, 1 h. (R_{mp})-**5**: 64% yield over two steps (98% *ee*). (S_{mp})-**6**: 62% (98% *ee*; data not shown, see Supplemental Information).

RESULTS AND DISCUSSION

Synthesis and Characterization of Mechanically Planar Chiral Complex [Au(6)(Cl)]

To demonstrate the potential of mechanical stereochemistry in catalysis, we selected a Au^I-mediated reaction for our study. Au^I-mediated reactions are inherently difficult to render enantioselective as a result of the linear coordination chemistry of the metal ion.[42] These challenges are typically overcome through the use of large, monodentate ligands that project substituents into the reaction space or di-Au^I complexes, in which aurophilic interactions pre-organize the complex with one metal ion playing the role of the catalyst and the other of a structural unit,[42,43] although employing secondary interactions in bifunctional catalysts is a promising emerging strategy.[44–46] Given that we have previously shown that the mechanical bond can be used to project steric bulk around an Au^I center, leading to highly diastereoselective catalysis,[47] we proposed that similar effects might be observed in the case of a mechanically chiral derivative, leading to enantioselective catalysis.

Rotaxane Au^I complex [Au(**6**)(Cl)] was synthesized using our small macrocycle modification[48] of Leigh's active template[49] Cu-mediated alkyne-azide cycloaddition reaction (AT-CuAAC),[50,51] employing amino-acid-derived azide **1** as a stereo-differentiating unit,[39] borane-protected propargylic phosphine **2** as the alkyne coupling partner, and readily available C_{1h} (C_s) symmetric macrocycle **3**,[52] as the key mechanical bond forming step. We typically carry out the AT-CuAAC reaction in the presence of excess N^iPr_2Et, which accelerates the reaction by favoring the formation of the key macrocycle-Cu^I-acetylide complex intermediate. However, in this case, N^iPr_2Et was found to cause epimerization of the azide stereocenter, resulting in a mixture of all four possible stereoisomeric products. Replacing N^iPr_2Et with Proton Sponge drastically reduced the epimerization side reaction, allowing the mixture of diastereomeric phosphine oxides **4** to be separated[53] with excellent stereochemical purity after demetallation and oxidative work-up. Using this sequence, we were able to isolate rotaxanes (S,R_{mp})-**4** (98% *ee*, >99: <1 *dr*) and (S,S_{mp})-**4** ((S,S_{mp})-**4**-(R,S_{mp})-**4**-(S,R_{mp})-**4** = 98.4:1.0:0.6, i.e., >98%

CellPress

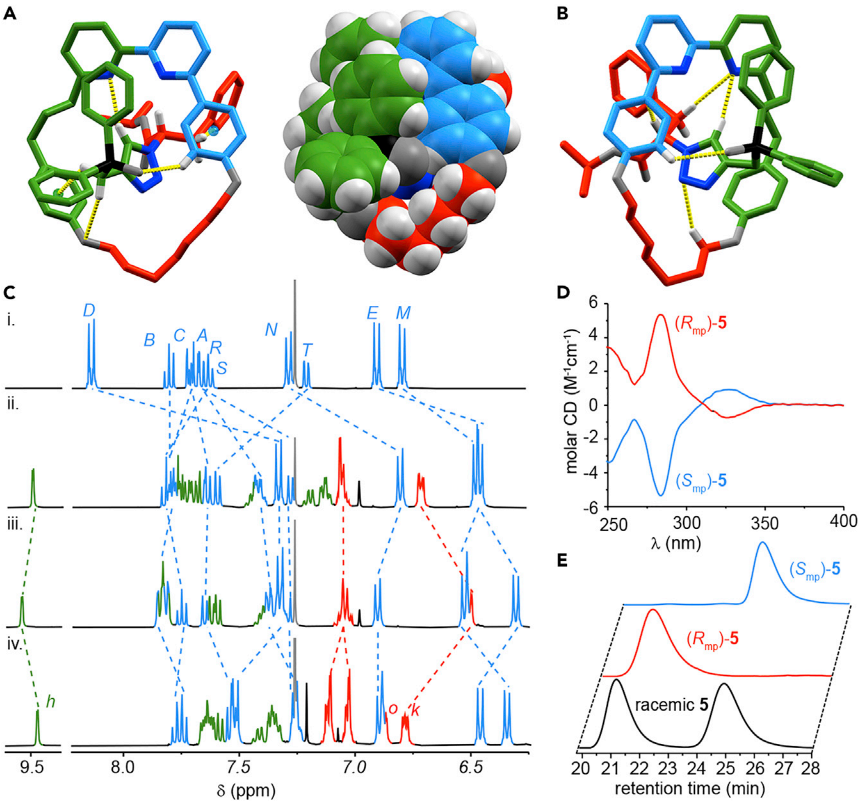

Figure 2. Characterization of Rotaxanes 4 and 5

(A) Solid-state structure of (S,R_{mp})-**4** with selected intercomponent interactions highlighted (atom labels and colors [O, dark gray; N, dark blue] as in Scheme 1, selected distances [Å]: H_g●●●O = 2.4, H_g●●●centroid = 2.6, H_h●●●N = 2.5, H_j●●●centroid = 3.2, and H_E●●●O = 2.5).

(B) Solid-state structure of (S,S_{mp})-**4** with selected intercomponent interactions (atom labels and colors [O, dark gray; N, dark blue] as in Scheme 1, selected distances [Å]: H_h●●●N = 2.4, H_i●●●C = 2.6, H_j●●●N = 2.7, and H_E●●●O = 2.7). It should be noted that the asymmetric unit contains an oxidized derivative of (S,S_{mp})-**4** as a disordered impurity.[54] The figure depicts the component of the unit cell that is unaffected by this disorder.

(C) Partial ^{1}H NMR ($CDCl_3$, 400 MHz, 298 K) of (i) macrocycle **3**, (ii) rotaxane (S,R_{mp})-**4**, (iii) rotaxane (S,S_{mp})-**4**, and (iv) rotaxane (R_{mp})-**5**. Selected signals are assigned and color coded (see Scheme 1 for labels; H_k and H_o, assigned arbitrarily, are the *ortho* protons of the diastereotopic axle benzyl groups). Signals corresponding to macrocycle **3** are all shown in blue for clarity.

ee in the mechanical stereogenic unit) in an acceptable combined yield of 54%. Alkylation of diastereomer (S,R_{mp})-**4** with BnI erased the covalent stereogenic unit to produce rotaxane (R_{mp})-**5**, in which the mechanical bond provides the sole stereogenic unit in excellent yield and enantiopurity (81% and 98% *ee*). Subsequent reduction of the phosphine oxide moiety and coordination of AuCl produced the Au^I precatalyst [Au((R_{mp})–**6**)(Cl)], the enantiopurity of which was assumed to be the same as that of (R_{mp})-**5** (98% *ee*) as the mechanical bond is configurationally stable. The same procedures starting from (S,S_{mp})-**4** produced [Au((S_{mp})–**6**)(Cl)] (98% *ee*).

Rotaxanes **4**, **5**, and [Au(**6**)(Cl)] were isolated and characterized in full by NMR, mass spectrometry (MS), HPLC (**4** and **5**), and CD (see Supplemental Information for full details). The absolute stereochemistries of phosphine oxides (S,R_{mp})-**4** and (S,S_{mp})-**4**[54] were assigned by single-crystal X-ray diffraction (SC-XRD)[55] (Figures 2A and 2B); the internal stereochemical reference provided by the azide-derived unit allowed the

orientation of the macrocycle to be determined unambiguously and the stereochemical labels were assigned using our established approach (see Supplemental Information for details).[6,9,10] The absolute stereochemistry of rotaxanes **5** and [Au(**6**)(Cl)] were inferred by noting that the mechanical stereochemistry of the corresponding diastereomeric starting materials cannot be altered in subsequent reactions.

The ^{1}H NMR spectra of diastereomers (S,R_{mp})-**4** and (S,S_{mp})-**4** (Figures 2Cii and 2Ciii, respectively) display the typical features of such interlocked molecules;[48] many of the signals corresponding to the axle and macrocycle components, including H_D, H_E, H_M, and H_N are shielded relative to the non-interlocked macrocycle (Figure 2Ci), and triazole proton H_h appears at a high chemical shift due to the formation of an intercomponent C–H●●●N hydrogen bond with the bipyridine, as observed in the solid-state structures (Figures 2A and 2B). However, their ^{1}H NMR spectra are clearly distinct, in keeping with the diastereomeric relationship between the two products, as are their CD spectra (see Supplemental Information). Alkylation of rotaxanes **4** to give rotaxanes **5**, produced materials with identical ^{1}H NMR spectra (Figure 2Biv) but mirror image CD spectra (Figure 2D), in keeping with the enantiomeric relationship between these products. Strikingly, in addition to the expected shielding/deshielding of signals, the aromatic protons corresponding to the diastereotopic benzylic units of the axle in rotaxanes **5** are clearly distinct (e.g., benzylic *ortho* protons H_k and H_o), suggesting that the stereochemistry of the mechanical bond is well expressed onto the axle.

Enantioselective Cyclopropanation Reactions Mediated by Rotaxane [Au((R_{mp})−6)(Cl)]

With the precatalyst [Au((R_{mp})−**6**)(Cl)] in hand, we investigated its behavior in the enantioselective Au^I-mediated variant of the Ohe-Uemura[56] cyclopropanation of alkenes by propargylic esters originally reported by Toste and co-workers using (R)-DTBM-SEGPHOS$(AuCl)_2$ and resulting in stereoselectivities from 60% to 94% *ee*.[41] More recently, Fuerstner and co-workers reported a mono-dentate 1,1-bi-2-naphthol-derived phosphoramite ligand for the same reaction,[57] and Toste and co-workers reported a reaction system that employs Au nanoclusters embedded in a chiral self-assembled monolayer.[58]

Under conditions previously optimized for an analogous achiral rotaxane-based catalyst,[47] [Au((R_{mp})−**6**)(Cl)] mediated the reaction of benzoyl ester **7** with styrene (**8**) to produce cyclopropanes **9** in excellent selectivity for the *cis* diastereomer (Table 1, entry 1). The role of the Cu^I additive is to bind to the bipyridine moiety, preventing the Lewis base inhibition of the Au^I center; reactions in the absence of Cu^I were unsuccessful (entry 2).[47] Other cationic additives failed to activate the catalyst (see Supplemental Information). Analysis of the purified major diastereomer by chiral stationary phase HPLC revealed reasonable enantioselectivity for (1S,2R)-**9** (*er* = 72:28).[59] As expected, replacing [Au((R_{mp})−**6**)(Cl)] with [Au((S_{mp})−**6**)(Cl)] produced **9** with opposite enantioselectivity (entry 3). Variation of the solvent led to changes in the observed *er* of *cis*-**9** but no significant improvement (entries 4–7). Cooling the reaction to 0°C improved the *er* of the major diastereomer to 79:21 (entry 8). Cooling the reaction mixture further led to no significant improvement and slowed the process considerably (entry 9). For comparison, the same reaction mediated by (R)-DTBM-SEGPHOS$(AuCl)_2$ reported by Toste and co-workers produces cyclopropanes **9** in moderately higher and opposite stereoselectivity (entry 10).

With suitable conditions in hand (Table 1, entry 8), we performed a brief investigation of the effect of substrate on the stereoselectivity of reactions mediated by [Au((R_{mp})−**6**)(Cl)]

Table 1. Optimization of an Enantioselective Cyclopropanation Reaction Mediated by [Au(6)(Cl)][a]

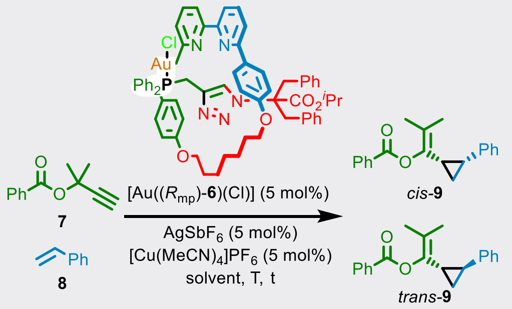

Entry[a]	Solvent	Temperature (°C)	Time (h)	*cis:trans*[b]	*er$_{cis}$*[c]	*er$_{trans}$*[c]
1	$CDCl_3$	25	1	95:5	72:28	58:42
2[d]	$CDCl_3$	25	1	n.r.	–	–
3[e]	$CDCl_3$	25	1	95:5	29:71	42:58
4	$MeNO_2$	25	1	87:13	53:47	65:35
5	CD_2Cl_2	25	1	83:17	64:36	66:34
6	CCl_4	25	1	86:14	71:29	58:42
7	PhMe	25	1	85:15	69:31	56:44
8[f]	$CDCl_3$	0	6	94:6	79:21	62:38
9	$CDCl_3$	−35	24	96:4	79:21	61:39
10[g]	$MeNO_2$	25	0.5	>20:<1	16:84	–

[a][Au(**6**)(Cl)] with 84% *ee* was used for screening experiments unless otherwise stated.
[b]Determined by ^{1}H NMR analysis of the crude reaction product using $C_2Cl_4H_2$ as an internal standard (yield determination).
[c]Determined by HPLC.
[d]Reaction was conducted without the Cu^I additive.
[e]Reaction conducted with [Au((S_{mp})−6)(Cl)].
[f]Reaction conducted with [Au(**6**)(Cl)] with *er* = 99:1 stereopurity.
[g]Reaction outcome reported by Toste and co-workers for (*R*)-DTBM-SEGPHOS$(AuCl)_2$.[41]

(Figure 3). Variation of the styrene component in the reaction of benzoate ester **7** gave cyclopropanes **10** and **11** in similar *ee* and *de* to **9**, although the yield of the reaction was much lower in the case of 2-Me-substituted product **11**. Replacing styrene with allyl benzene gave cyclopropane **12** in reasonable enantioselectivity but lower diastereoselectivity, as has previously been observed for aliphatic alkenes.[41] Conversely, variation of the propargylic ester component had a significant effect on the reaction stereoselectivity. Whereas (*R*)-DTBM-SEGPHOS$(AuCl)_2$ is reported to deliver higher stereoselectivity with the pivaloyl derivative of propargyl ester **7**, in the case of [Au((R_{mp})−**6**)(Cl)], cyclopropane **13** was produced with almost no enantioselectivity. Pleasingly, phenylacetate ester-derived cyclopropane **14** was produced in comparable selectivity to **9**, confirming that α-alkyl esters are tolerated by [Au(**6**)(Cl)] and suggesting that the steric bulk of the pivolyl moiety is responsible for the loss of stereoselectivity in the case of **13**. Variation of the benzoyl moiety to introduce strongly electron-withdrawing or -donating groups (cyclopropanes **15** and **16**, respectively) led to a reduction in reaction enantioselectivity. In contrast, bulky alkyl groups on the benzoate moiety increased the reaction *ee*; *p*-^{t}Bu benzoyl cyclopropane **17** and 3,5-di-^{t}Bu-substituted cyclopropane **18** were produced in good yield and enantioselectivity. Cyclopropanes **9–18** were isolated by flash chromatography prior to HPLC analysis; the catalyst and any associated decomposition products were readily removed from the product mixture.

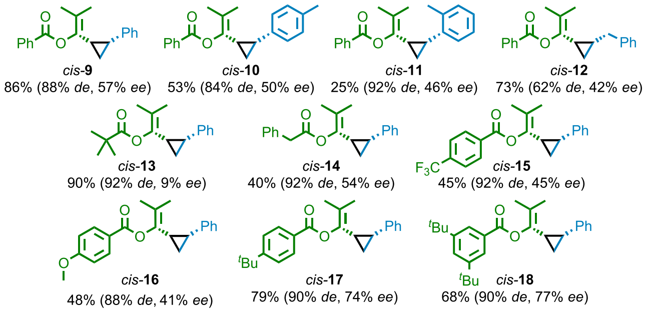

Figure 3. Cyclopropane Products Synthesized Using [Au((R_{mp})−6)(Cl)]

All reactions carried out under the conditions shown in Table 1, entry 8. Combined yields of cyclopropanes and *de* were determined by ^{1}H NMR analysis of the crude reaction product using $C_2Cl_4H_2$ as an internal standard. *ee* of the major *cis* diastereomer determined by HPLC analysis of purified samples.[59]

Modeling of the Au^{I}-Mediated Cyclopropanation of Styrene

Detailed modeling of interlocked molecules is challenging, given both their size and flexibility. Previously, the catalytic behavior of an interlocked catenane organocatalyst was studied computationally by considering the catalytic fragment alone on the assumption that the rest of the structure did not play a direct role in the reaction.[60] In the case of [Au(**6**)(Cl)], this clearly would not be a reasonable assumption as the mechanical bond is the sole source of stereochemistry. Also, the implied difference in activation barrier, even for the most selective example reported above (**18**) is only ~4.5 kJmol^{-1}, a relatively small value for such a complex system where multiple conformations of the catalyst may be mechanistically relevant. These caveats notwithstanding, in order to gain some qualitative insight into how interactions between the reacting substrates and the rotaxane structure might influence the stereoselectivity of the reaction, we conducted preliminary computational modeling of the reaction of propargylic ester **7** and styrene (**8**) mediated by [Au(R_{mp}-**6**)(Cl)].

In brief (for full details see Supplemental Information), we began by locating the lowest energy transition state (CAM-B3LYP/6-31G*/SDD(Au)) for the reaction of **7** with **8** mediated by [Au(PPh_3)(Cl)], building on previous work by Echavarren and co-workers.[61] In keeping with this previous report, the reaction of the carbene derived from **7** with **8** was found to be a two-step process. We thus assumed a similar pathway for the reaction mediated by [Au(**6**)(Cl)] (Figure 4A); coordination of Cu^{I} and abstraction of the Cl ligand gives rise to the proposed active catalyst [AuCu(**6**)]$^{2+}$, which coordinates to alkyne **7** to give complex **I** that undergoes a rearrangement to produce key carbene intermediate **II**. Addition of styrene to **II** produces carbocation **III** via key transition state **TSI**, in the process setting the stereochemistry of C^{1} of the cyclopropane product. Subsequent rapid ring closure gives rise to cyclopropane **9** and regenerates the catalyst.

In order to investigate the reaction mediated by rotaxane [Au(**6**)(Cl)], the transition state model found for the reaction mediated by [Au(PPh_3)(Cl)] was modified by attachment to the Cu^{I}-coordinated metallorotaxane.[62–64] A conformational search (Spartan '10, MMF)[65] with the transition state fragment frozen yielded low-energy conformers for each diastereomeric complex, which were optimized using density functional theory (DFT) (Gaussian '09,[66] CAM-B3LYP, 6–31G/SDD(Cu,Au)), again with the transition state fragment frozen, to identify the lowest energy conformation. Transition state optimization, first using an ONIOM method (CAM-B3LYP:UFF,

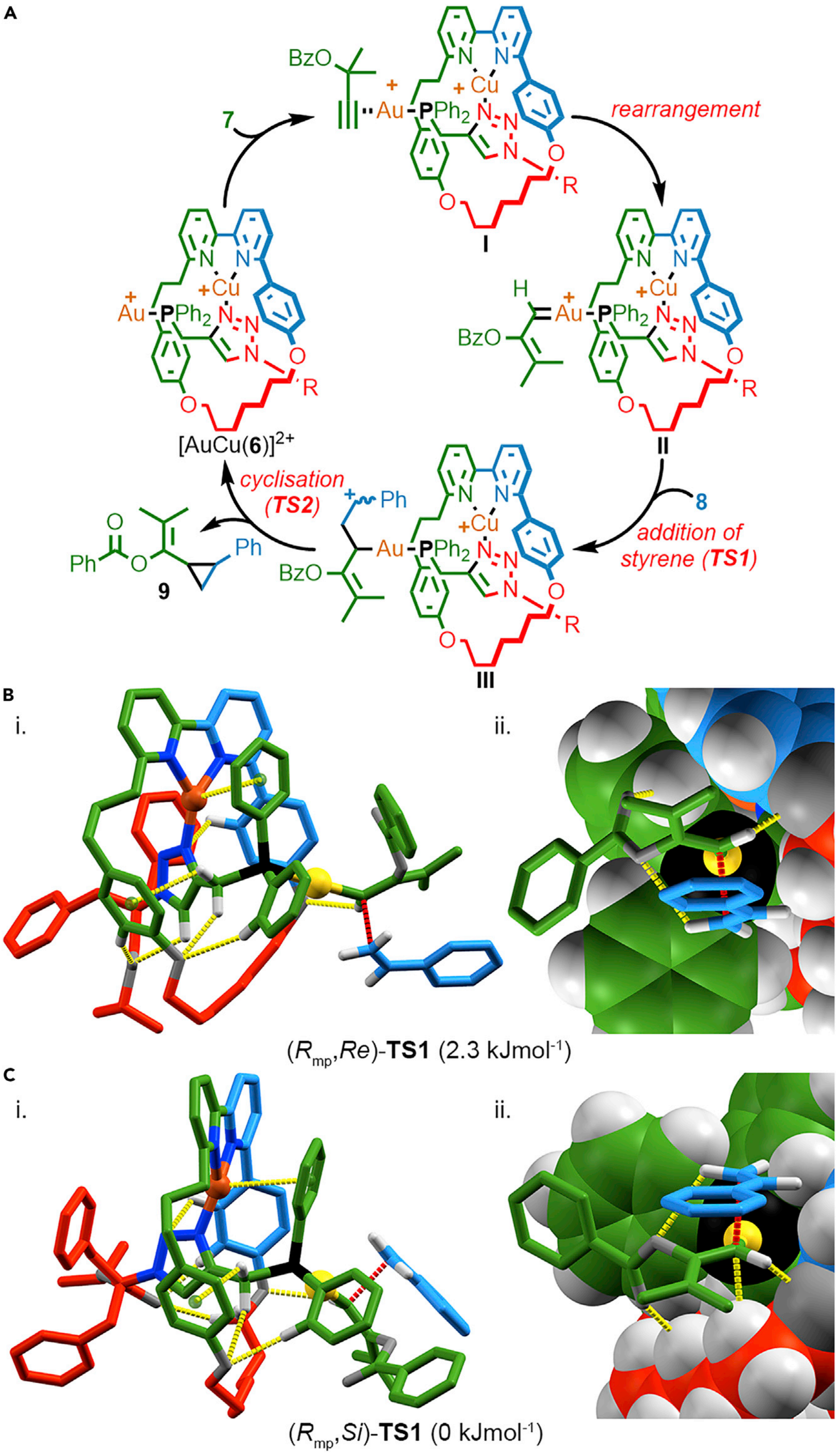

Figure 4. Reaction Pathway and Modeled Transition State Structures

(A) Reaction pathway presumed for the reaction of [Au(**6**)(Cl)] based on molecular modeling (Gaussian '09, CAM-B3LYP, 6-31G*/SDD(Au)) of the reaction of **7** and **8** mediated by [Au(PPh_3)(Cl)]. R = $C(Bn)_2CO_2{}^iPr$.

(B) Modeled ($CHCl_3$, CAM-B3LYP, 6-31G/SDD) structure of **TS1** leading to (1*R*,2*S*)-**9** for the reaction of **7** with **8** mediated by $[AuCu((R_{mp})\text{-}\mathbf{6})]^{2+}$ in (i) sticks representation and (ii) close-up of the

Figure 4. *Continued*

transition state fragment in mixed space-filling and sticks representation. Selected intercomponent interactions and the carbene-styrene interaction associated with the reaction coordinate are highlighted in yellow and red, respectively.

(C) Modeled ($CHCl_3$, CAM-B3LYP, 6-31G/SDD) structure of **TS1** leading to (1*S*,2*R*)-**9** for the reaction of **7** with **8** mediated by $[AuCu((R_{mp})\text{-}\mathbf{6})]^{2+}$ in (i) sticks representation and (ii) close-up of the transition state fragment in mixed space-filling and sticks representation. Selected intercomponent interactions and the carbene-styrene interaction associated with the reaction coordinate are highlighted in yellow and red, respectively.

6–31G/SDD(Au)), followed by a full DFT optimization (CAM-B3LYP, 631G/SDD(Cu,Au)) first in the gas phase then in solvent ($CHCl_3$, polarizable continuum model) yielded transition state models (R_{mp},*Re*)-**TS1** and (R_{mp},*Si*)-**TS1** (Figures 4B and 4C, respectively) that were determined to be first order saddle points with a single imaginary frequency.

Examining the models of (R_{mp},*Re*)-**TS1** and (R_{mp},*Si*)-**TS1** (Figures 4B and 4C, respectively) reveals that, in spite of their size and large number of rotatable bonds, the modeled catalyst structure is actually relatively rigid because of steric crowding combined with the coordination of the Cu^{I} ion. A complex network of short intra- and intercomponent contacts including CH hydrogen bonds, CH-π interactions, and cation-π interaction between the Cu^{I} ion and one of the Ph rings of the phosphine ligand are predicted to stabilize the system further and project the Au^{I} center bearing the reactive carbene moiety toward the macrocycle, into the space around one of the phenoxy ether moieties. It is perhaps noteworthy that the optimized structures are similar to the solid-state structures of rotaxanes **4** determined by X-ray diffraction in which the phosphine substituent (O) is also projected toward the same aryl ether moiety. Crowding around the Au^{I} carbene moiety due to the mechanical bond is clearly seen in the space-filling models of (R_{mp},*Re*)-**TS1** and (R_{mp},*Si*)-**TS1** (Figures 4Bii and 4Cii, respectively); the macrocycle provides a sterically crowded environment that shields one face of the carbene unit and restricts the rotation of the substrate around the Au-P axis. The substrates are stabilized in the rotaxane environment through a number of non-covalent interactions, in particular a C(carbene)H–O interaction in both structures and a CH-C(carbene) interaction in the case of (R_{mp},*Si*)-**TS1**. Thus, the modeling suggests that a mechanically bonded structure provides a well-expressed chiral environment for the catalysis to take place within, which is consistent with the reasonable enantioselectivities achieved experimentally.

Finally, comparison of the calculated relative energies of (R_{mp},*Re*)-**TS1** and (R_{mp},*Si*)-**TS1** revealed remarkable agreement, given the size of the system, between experiment and theory; (R_{mp},*Si*)-**TS1** was found to be favored by $\sim$2.3 $kJmol^{-1}$, corresponding to a stereoselectivity of 74:26 in favor of the major observed product (1*S*,2*R*)-**9**. However, caution should be taken when interpreting this level of agreement; modeling in the gas phase (6–31G/SDD) predicted the opposite stereoselectivity ((R_{mp},*Re*)-**TS1** favored by $\sim$1.7 $kJmol^{-1}$). Conversely, re-optimization of **TS1** with the larger 6–31G* basis set in the gas phase or in $CHCl_3$ (single point calculation)[67] resulted in a predicted selectivity for the correct diastereomer that exceeds what is observed experimentally, demonstrating the uncertainty in the absolute values generated in such complex systems. Furthermore, although extending the modeling to the reactions leading to cyclopropanes **15** and **16** revealed reasonable agreement with experiment, the same calculations for the reaction leading to cyclopropanes **13** predicted a high selectivity, in contrast to the low selectivity observed experimentally (see Supplemental Information for details).

Thus, the molecular models of (R_{mp},*Re*)-**TS1** and (R_{mp},*Si*)-**TS1** should be considered qualitative, providing some insight into the potential interactions and a pictorial representation of the chiral environment created by the mechanical bond around the reacting Au^I carbene. A more detailed study, combined with many more comparisons between experiment and theory, would be required to determine the details of the key intermolecular interactions that lead to the observed stereoselectivity.

Conclusions

Although the first enantiopure mechanically planar chiral rotaxane was reported over two decades ago,[15] this is, to our knowledge, the first time that this stereogenic unit has been applied in catalysis. The results presented clearly demonstrate that the mechanically planar chiral stereogenic unit can direct enantioselective catalysis. The results are particularly pleasing given that rotaxane **6** was not explicitly designed or optimized for the reaction presented and yet achieves stereoselectivities with benzoate esters of 42%–77% *ee*, comparable to a similar reaction mediated by optimized covalent catalyst (*R*)-DTBM-SEGPHOS$(AuCl)_2$ (68% *ee*). By extension, our results suggest that other mechanical stereogenic units[6] such as the axial and topological chiral units in catenanes have unexplored potential in catalytic applications.

However, the stereoselectivities observed in this cyclopropanation reaction are lower than those reported when pivloate esters, which are not tolerated by [Au(**6**)(Cl)], were employed with the best covalent catalysts (76% to 94% *ee*),[41] clearly demonstrating that challenges remain to be overcome for mechanically chiral rotaxanes to become useful tools in organic synthesis. It should also be noted that preliminary attempts to apply [Au(**6**)(Cl)] to other Au^I-mediated reactions were unsuccessful (see Supplemental Information), suggesting that our success in this one reaction is serendipitous rather than an indication that the mechanically planar chiral stereogenic unit is somehow a "magic bullet" for enantioselective gold catalysis. Indeed, this is consistent with results with covalent catalysts (e.g., (*R*)-DTBM-SEGPHOS$(AuCl)_2$—see Supplemental Information) that have been optimized for one Au^I-mediated reaction but often perform poorly in others.[45] Furthermore, despite recent progress in the area,[38–40] the synthesis of mechanically interlocked molecules is still challenging, in the example presented, specifically due to the low stereoselectivity observed in the mechanical bond forming step and epimerization of the stereodirecting unit derived from the α-chiral azide that complicates the purification. This synthetic challenge clearly complicates the optimization of catalyst frameworks to deliver enhanced enantioselectivity. However, recent progress in the development of new methodologies to access enantiopure mechanically chiral molecules suggests that this synthetic challenge can and is being addressed, and pleasingly, based on the preliminary molecular modeling presented, it seems that modern computational chemistry may well be able to aid the design process.

Thus, in the future, we see a place for mechanical chirality in catalysis, particularly where it is otherwise challenging to project chiral information into the reaction space, as in the Au^I-mediated reaction presented here; the crowded, three-dimensional[68,69] nature of the mechanical bond appears to be well suited to generating a chiral pocket for chemical reactions to take place within, similar in some ways to enzymatic active sites with their combination of steric hindrance and weak attractive interactions with the substrate. Furthermore, combining chiral mechanical stereogenic units with the well-developed chemistry of interlocked molecular shuttles[2,70–73] should allow the influence of the stereogenic mechanical bond to be modulated[74] in a stimuli-responsive manner in order to develop switchable chiral

catalysts, for instance, to produce both hands of a given chiral product in high enantioselectivity[75,76] Indeed, during the preparation of this manuscript, this principle was demonstrated in the context of co-conformational covalent point chirality.[77] The same principles may also hold in the development of enantioselective sensors for chiral molecules. What is clear, based on these results, is that the chemical applications[78,79] of mechanically chiral interlocked molecules deserve further investigation.

DATA AND CODE AVAILABILITY

The accession numbers for the solid-state structures of (S,R_{mp})-**4** and (S,S_{mp})-**4** reported in this paper are CCDC: 1950723, 1980985, respectively. Processed compound characterization data are available freely from the University of Southampton repository (https://doi.org/10.5258/SOTON/D1223).

SUPPLEMENTAL INFORMATION

Supplemental Information can be found online at https://doi.org/10.1016/j.chempr.2020.02.006.

ACKNOWLEDGMENTS

S.M.G. thanks the European Research Council (consolidator grant agreement no. 724987) and Leverhulme Trust (ORPG-2733) for funding and the Royal Society for a Research Fellowship. S.M.G. is a Royal Society Wolfson Research Fellow. The authors would like to thank Dr. Marzia Galli and Dr. Jorge Meijide Suarez for helpful discussions and the latter for preparation of starting material S12. The authors thank Dr. Graham Tizzard of the National Crystallographic Service for helpful discussions around the SC-XRD data. The authors acknowledge the use of the IRIDIS High Performance Computing Facility and associated support services at the University of Southampton, in the completion of this work.

AUTHOR CONTRIBUTIONS

S.M.G. conceived the project and secured the project funding. A.W.H. contributed to the design of the experiments and methodology and executed all of the experimental procedures. S.M.G. carried out the computational modeling. S.M.G. wrote the manuscript with input from A.W.H. Both authors contributed to the reviewing and editing of the manuscript.

DECLARATION OF INTERESTS

The authors declare no competing interests.

Received: September 1, 2019
Revised: October 1, 2019
Accepted: February 10, 2020
Published: March 9, 2020

REFERENCES

1. Bruns, C.J., and Stoddart, J.F. (2016). The Nature of the Mechanical Bond: From Molecules to Machines (Wiley).
2. Erbas-Cakmak, S., Leigh, D.A., McTernan, C.T., and Nussbaumer, A.L. (2015). Artificial molecular machines. Chem. Rev. *115*, 10081–10206.
3. Stoddart, J.F. (2017). Mechanically interlocked molecules (MIMs)-molecular shuttles, switches, and machines (nobel lecture). Angew. Chem. Int. Ed. *56*, 11094–11125.
4. Sauvage, J.P. (2017). From chemical topology to molecular machines (nobel lecture). Angew. Chem. Int. Ed. *56*, 11080–11093.
5. Feringa, B.L. (2017). The art of building small: from molecular switches to motors (nobel lecture). Angew. Chem. Int. Ed. *56*, 11060–11078.
6. Jamieson, E.M.G., Modicom, F., and Goldup, S.M. (2018). Chirality in rotaxanes and catenanes. Chem. Soc. Rev. *47*, 5266–5311.

7. Frisch, H.L., and Wasserman, E. (1961). Chemical topology[1]. J. Am. Chem. Soc. *83*, 3789–3795.

8. Schill, G. (1971). Catenanes, Rotaxanes and Knots (Academic Press).

9. Pairault, N., and Niemeyer, J. (2018). Chiral mechanically interlocked molecules – applications of rotaxanes, catenanes and molecular knots in stereoselective chemosensing and catalysis. Synlett *29*, 689–698.

10. Evans, N.H. (2018). Chiral catenanes and rotaxanes: fundamentals and emerging applications. Chemistry *24*, 3101–3112.

11. Dálaigh, C.O., Hynes, S.J., Maher, D.J., and Connon, S.J. (2005). Kinetic resolution of sec-alcohols using a new class of readily assembled (S)-proline-derived 4-(pyrrolidino)-pyridine analogues. Org. Biomol. Chem. *3*, 981–984.

12. Spivey, A.C., Zhu, F., Mitchell, M.B., Davey, S.G., and Jarvest, R.L. (2003). Concise synthesis, preparative resolution, absolute configuration determination, and applications of an atropisomeric biaryl catalyst for asymmetric acylation. J. Org. Chem. *68*, 7379–7385.

13. Ruble, J.C., Latham, H.A., and Fu, G.C. (1997). Effective kinetic resolution of secondary alcohols with a planar-chiral analogue of 4-(dimethylamino)pyridine. Use of the $Fe(C_5Ph_5)$ group in asymmetric catalysis. J. Am. Chem. Soc. *119*, 1492–1493.

14. Crittall, M.R., Rzepa, H.S., and Carbery, D.R. (2011). Design, synthesis, and evaluation of a helicenoidal DMAP lewis base catalyst. Org. Lett. *13*, 1250–1253.

15. Yamamoto, C., Okamoto, Y., Schmidt, T., Jäger, R., and Vögtle, F. (1997). Enantiomeric resolution of cycloenantiomeric rotaxane, topologically chiral catenane, and pretzel-shaped molecules: observation of pronounced circular dichroism. J. Am. Chem. Soc. *119*, 10547–10548.

16. Kaida, Y., Okamoto, Y., Chambron, J.-C., Mitchell, D.K., and Sauvage, J.-P. (1993). The separation of optically active copper (I) catenates. Tetrahedron Lett. *34*, 1019–1022.

17. Hirose, K., Ukimi, M., Ueda, S., Onoda, C., Kano, R., Tsuda, K., Hinohara, Y., and Tobe, Y. (2018). The asymmetry is derived from mechanical interlocking of achiral axle and achiral ring components –syntheses and properties of optically pure [2]rotaxanes–. Symmetry *10*, 20.

18. Ishiwari, F., Nakazono, K., Koyama, Y., and Takata, T. (2017). Induction of Single-Handed helicity of polyacetylenes using mechanically chiral rotaxanes as chiral sources. Angew. Chem. Int. Ed. *56*, 14858–14862.

19. Mochizuki, Y., Ikeyatsu, K., Mutoh, Y., Hosoya, S., and Saito, S. (2017). Synthesis of mechanically planar chiral rac- [2]rotaxanes by partitioning of an achiral [2]rotaxane: stereoinversion induced by shuttling. Org. Lett. *19*, 4347–4350.

20. Corra, S., de Vet, C., Groppi, J., La Rosa, M., Silvi, S., Baroncini, M., and Credi, A. (2019). Chemical on/off switching of mechanically planar chirality and chiral anion recognition in a [2]rotaxane molecular shuttle. J. Am. Chem. Soc. *141*, 9129–9133.

21. Cakmak, Y., Erbas-Cakmak, S., and Leigh, D.A. (2016). Asymmetric catalysis with a mechanically point-chiral rotaxane. J. Am. Chem. Soc. *138*, 1749–1751.

22. Goldup, S.M. (2016). Mechanical chirality: a chiral catalyst with a ring to it. Nat. Chem. *8*, 404–406.

23. Alvarez-Pérez, M., Goldup, S.M., Leigh, D.A., and Slawin, A.M.Z. (2008). A chemically-driven molecular information ratchet. J. Am. Chem. Soc. *130*, 1836–1838.

24. Carlone, A., Goldup, S.M., Lebrasseur, N., Leigh, D.A., and Wilson, A. (2012). A three-compartment chemically-driven molecular information ratchet. J. Am. Chem. Soc. *134*, 8321–8323.

25. Eliel, E., Wilen, S., and Mander, L. (1994). Stereochemistry of Organic Compounds (John Wiley and Sons, Inc)).

26. By chirotopic elements, we mean stereogenic units around that are locally chiral (see: Mislow, K., and Siegel, J. (1984). Stereoisomerism and local chirality J. Am. Chem. Soc. *106*, 3319–3328, Similarly, when we describe a molecule as "mechanically planar chiral" (or similar) this is shorthand for a molecule that is chiral as a consequence of containing the mechanical planar chiral stereogenic unit.

27. Noyori, R. (2002). Asymmetric catalysis: science and opportunities (nobel lecture). Angew. Chem. Int. Ed. *41*, 2008–2022.

28. Knowles, W.S. (2002). Asymmetric hydrogenations (nobel lecture). Angew. Chem. Int. Ed. *41*, 1999–2007.

29. Sharpless, K.B. (2002). Searching for new reactivity (nobel lecture). Angew. Chem. Int. Ed. *41*, 2024–2032.

30. Phipps, R.J., Hamilton, G.L., and Toste, F.D. (2012). The progression of chiral anions from concepts to applications in asymmetric catalysis. Nat. Chem. *4*, 603–614.

31. Hong, Y., Jarrige, L., Harms, K., and Meggers, E. (2019). Chiral-at-iron catalyst: expanding the chemical space for asymmetric earth-abundant metal catalysis. J. Am. Chem. Soc. *141*, 4569–4572.

32. Yashima, E., Maeda, K., Iida, H., Furusho, Y., and Nagai, K. (2009). Helical polymers: synthesis, structures, and functions. Chem. Rev. *109*, 6102–6211.

33. Boersma, A.J., Megens, R.P., Feringa, B.L., and Roelfes, G. (2010). DNA-based asymmetric catalysis. Chem. Soc. Rev. *39*, 2083–2092.

34. Silverman, S.K. (2010). DNA as a versatile chemical component for catalysis, encoding, and stereocontrol. Angew. Chem. Int. Ed. *49*, 7180–7201.

35. Nagata, Y., Takeda, R., and Suginome, M. (2019). Asymmetric catalysis in chiral solvents: chirality transfer with amplification of homochirality through a helical macromolecular scaffold. ACS Cent. Sci. *5*, 1235–1240.

36. Tan, C., Chu, D., Tang, X., Liu, Y., Xuan, W., and Cui, Y. (2019). Supramolecular coordination cages for asymmetric catalysis. Chemistry *25*, 662–672.

37. Zhang, P., Tugny, C., Meijide Suárez, J., Guitet, M., Derat, E., Vanthuyne, N., Zhang, Y., Bistri, O., Mouriès-Mansuy, V., Ménand, M., et al. (2017). Artificial chiral metallo-pockets including a single metal serving as structural probe and catalytic center. Chem *3*, 174–191.

38. Bordoli, R.J., and Goldup, S.M. (2014). An efficient approach to mechanically planar chiral rotaxanes. J. Am. Chem. Soc. *136*, 4817–4820.

39. Jinks, M.A., de Juan, A., Denis, M., Fletcher, C.J., Galli, M., Jamieson, E.M.G., Modicom, F., Zhang, Z., and Goldup, S.M. (2018). Stereoselective synthesis of mechanically planar chiral rotaxanes. Angew. Chem. Int. Ed. *57*, 14806–14810.

40. Denis, M., Lewis, J.E.M., Modicom, F., and Goldup, S.M. (2019). An auxiliary approach for the stereoselective synthesis of topologically chiral catenanes. Chem *5*, 1512–1520.

41. Johansson, M.J., Gorin, D.J., Staben, S.T., and Toste, F.D. (2005). Gold(I)-catalyzed stereoselective olefin cyclopropanation. J. Am. Chem. Soc. *127*, 18002–18003.

42. Wang, Y.M., Lackner, A.D., and Toste, F.D. (2014). Development of catalysts and ligands for enantioselective gold catalysis. Acc. Chem. Res. *47*, 889–901.

43. Zi, W., and Dean Toste, F. (2016). Recent advances in enantioselective gold catalysis. Chem. Soc. Rev. *45*, 4567–4589.

44. Wang, Z., Nicolini, C., Hervieu, C., Wong, Y.F., Zanoni, G., and Zhang, L. (2017). Remote cooperative group strategy enables ligands for accelerative asymmetric gold catalysis. J. Am. Chem. Soc. *139*, 16064–16067.

45. For a recent example of an elegant design strategy to control the enantioselective folding of an enyne substrate on a π-surface, as well as where a very large number of previously successful ligands for Au^{I} enantioselective catalysis were screened (often with poor results) in a new reaction, see: Zuccarello, G., Mayans, J.G., Escofet, I., Scharnagel, D., Kirillova, M.S., Pérez-Jimeno, A.H., Calleja, P., Boothe, J.R., and Echavarren, A.M. (2019). Enantioselective folding of enynes by gold(I) catalysts with a remote C_2 -chiral element J. Am. Chem. Soc. *141*, 11858–11863.

46. Wang, Y., Zhang, P., Di, X., Dai, Q., Zhang, Z.M., and Zhang, J. (2017). Gold-catalyzed asymmetric intramolecular cyclization of N-Allenamides for the synthesis of chiral Tetrahydrocarbolines. Angew. Chem. Int. Ed. *56*, 15905–15909.

47. Galli, M., Lewis, J.E.M., and Goldup, S.M. (2015). A stimuli-responsive Rotaxane-gold catalyst: regulation of activity and diastereoselectivity. Angew. Chem. Int. Ed. *54*, 13545–13549.

48. Lahlali, H., Jobe, K., Watkinson, M., and Goldup, S.M. (2011). Macrocycle size matters: "small" functionalized rotaxanes in excellent yield using the CuAAC active template approach. Angew. Chem. Int. Ed. *50*, 4151–4155.

49. Denis, M., and Goldup, S.M. (2017). The active template approach to interlocked molecules. Nat. Rev. Chem. *1*, 0061.

50. Aucagne, V., Hänni, K.D., Leigh, D.A., Lusby, P.J., and Walker, D.B. (2006). Catalytic "click" rotaxanes: a substoichiometric metal-template pathway to mechanically interlocked architectures. J. Am. Chem. Soc. *128*, 2186–2187.

51. Aucagne, V., Berna, J., Crowley, J.D., Goldup, S.M., Hänni, K.D., Leigh, D.A., Lusby, P.J., Ronaldson, V.E., Slawin, A.M.Z., Viterisi, A., and Walker, D.B. (2007). Catalytic 'active-metal' template synthesis of [2]rotaxanes, [3] rotaxanes, and molecular shuttles, and some observations on the mechanism of the cu(i)-catalyzed azide-alkyne 1,3-cycloaddition. J. Am. Chem. Soc. *129*, 11950–11963.

52. Lewis, J.E.M., Bordoli, R.J., Denis, M., Fletcher, C.J., Galli, M., Neal, E.A., Rochette, E.M., and Goldup, S.M. (2016). High yielding synthesis of 2,2′-bipyridine macrocycles, versatile intermediates in the synthesis of rotaxanes. Chem. Sci. *7*, 3154–3161.

53. Care must be taken in the separation of diastereomers **4** as the covalent stereogenic center is prone to epimerization. See Supplemental Information for full details

54. Although the SC-XRD-derived solid state structure of (S,Rmp)-**4** is of high quality, the single-crystal of (S,Smp)-**4** appears to be contaminated with an oxidized derivative of the rotaxane. The structure of (S,Rmp)-**4** alone is sufficient to assign the relative and, using the known stereochemistry of the azide derived component, the absolute stereochemistry of both diastereomers. However, the SC-XRD-derived structure of (S,Smp)-**4** is of good quality once the impurity is taken into account and, importantly, the relative stereochemistry observed is, as expected, epimeric with that determined for (S,Rmp)-**4**. For full details, see Supplemental Information.

55. SC-XRD allows the relative stereochemistry of the mechanical bond and covalent stereogenic unit to be determined unambiguously for the diastereomeric rotaxanes. This information, combined with the known configuration of the azide-derived stereogenic unit, allows the absolute stereochemistry of rotaxanes **4**, and their derivatives, to be determined

56. Miki, K., Ohe, K., and Uemura, S. (2003). A new ruthenium-catalyzed cyclopropanation of alkenes using propargylic acetates as a precursor of vinylcarbenoids. Tetrahedron Lett. *44*, 2019–2022.

57. Teller, H., Flügge, S., Goddard, R., and Fürstner, A. (2010). Enantioselective gold catalysis: opportunities provided by monodentate phosphoramidite ligands with an acyclic TADDOL backbone. Angew. Chem. Int. Ed. *49*, 1949–1953.

58. Gross, E., Liu, J.H., Alayoglu, S., Marcus, M.A., Fakra, S.C., Toste, F.D., and Somorjai, G.A. (2013). Asymmetric catalysis at the mesoscale: gold nanoclusters embedded in chiral self-assembled monolayer as heterogeneous catalyst for asymmetric reactions. J. Am. Chem. Soc. *135*, 3881–3886.

59. The absolute stereochemistry of (1*S*,2*R*)-**9** was assigned by comparison with the product of the reaction mediated by (*R*)-DTBM-SEGPHOS®$(AuCl)_2$, the stereochemical outcome of which is known.[41] The absolute configurations of cis-**15**, *cis*-**16**, and *cis*-**18** were determined to be (1S,2R) by convergence to the same reduction product as that produced by *cis*-**9** (see Supplemental Information for details). The absolute stereochemistry of all other cyclopropane products was not determined. The (1S,2R) products are shown but this assignment is arbitrary.

60. Mitra, R., Zhu, H., Grimme, S., and Niemeyer, J. (2017). Functional mechanically interlocked molecules: asymmetric organocatalysis with a catenated bifunctional brønsted acid. Angew. Chem. Int. Ed. *56*, 11456–11459.

61. Pérez-Galán, P., Herrero-Gómez, E., Hog, D.T., Martin, N.J.A., Maseras, F., and Echavarren, A.M. (2011). Mechanism of the gold-catalyzed cyclopropanation of alkenes with 1,6-enynes. Chem. Sci. *2*, 141–149.

62. It is an open question whether an interlocked molecule in which the components are bridged in this manner are true rotaxanes or if the metal-ligand interactions constitute a covalent link, rendering them entangled but not mechanically bonded, strictly speaking. Sauvage employed the term "catenate" to denote such complexes in the context of catenanes,[16] but the equivalent noun "rotaxanate" is more commonly used as a verb meaning "to make a rotaxane." For examples where rotaxanate is used as a noun, see: Furusho, Y., Matsuyama, T., Takata, T., Moriuchi, T., and Hirao, T. (2004). Synthesis of novel interlocked systems utilizing a palladium complex with 2,6-pyridinedicarboxamide-based tridentate macrocyclic ligand Tetrahedron Lett. *45*, 9593–9597.

63. Mateo-Alonso, A. (2010). Mechanically interlocked molecular architectures functionalised with fullerenes. Chem. Commun. (Camb.) *46*, 9089–9099.

64. Miyagawa, N., Watanabe, M., Matsuyama, T., Koyama, Y., Moriuchi, T., Hirao, T., Furusho, Y., and Takata, T. (2010). Successive catalytic reactions specific to Pd-based rotaxane complexes as a result of wheel translation along the axle. Chem. Commun. (Camb.) *46*, 1920–1922, Here we use the term "metallorotaxane," which has seen some use to denote the mechanically chelated complex, rather than "rotaxanate," to avoid confusion.[1].

65. Wavefunction Inc. and Q-Chem (2010). Spartan'10 (Wavefunction Inc).

66. Frisch, M.J., Trucks, G.W., Schlegel, H.B., Scuseria, G.E., Robb, M.A., Cheeseman, J.R., Scalmani, G., Barone, V., Mennucci, B., Petersson, G.A., et al. (2016). Gaussian 09, Revision A.02 (Gaussian, Inc.).

67. Single point energy calculation based on the gas phase 6–31G* structure; transition state optimization using the 6–31G* basis set in solvent proved prohibitively computationally expensive

68. For examples in which covalent chiral information is transferred through the mechanical bond, see: Tachibana, Y., Kihara, N., and Takata, T. (2004). Asymmetric benzoin condensation catalyzed by chiral rotaxanes tethering a thiazolium salt moiety via the cooperation of the component: can rotaxane be an effective reaction field? J. Am. Chem. Soc. *126*, 3438–3439.

69. Xu, K., Nakazono, K., and Takata, T. (2016). Design of Rotaxane Catalyst for *O* -Acylative Asymmetric Desymmetrization of meso -1,2-diol Utilizing the Cooperative Effect of the Components. Chem. Lett. *45*, 1274–1276.

70. For examples of switchable catalysts based on rotaxane molecular shuttles, see: Blanco, V., Carlone, A., Hänni, K.D., Leigh, D.A., and Lewandowski, B. (2012). A rotaxane-based switchable organocatalyst Angew. Chem. Int. Ed. *51*, 5166–5169.

71. Blanco, V., Leigh, D.A., Lewandowska, U., Lewandowski, B., and Marcos, V. (2014). Exploring the activation modes of a Rotaxane-based switchable organocatalyst. J. Am. Chem. Soc. *136*, 15775–15780.

72. Eichstaedt, K., Jaramillo-Garcia, J., Leigh, D.A., Marcos, V., Pisano, S., and Singleton, T.A. (2017). Switching between anion-binding catalysis and aminocatalysis with a rotaxane dual-function catalyst. J. Am. Chem. Soc. *139*, 9376–9381.

73. Blanco, V., Leigh, D.A., Marcos, V., Morales-Serna, J.A., and Nussbaumer, A.L. (2014). A switchable [2]rotaxane asymmetric organocatalyst that utilizes an acyclic chiral secondary amine. J. Am. Chem. Soc. *136*, 4905–4908.

74. Suzuki, S., Ishiwari, F., Nakazono, K., and Takata, T. (2012). Reversible helix–random coil transition of poly(m-phenylenediethynylene) by a rotaxane switch. Chem. Commun. (Camb.) *48*, 6478–6480.

75. For examples of non-interlocked molecular machines that control the stereoselective synthesis of molecules, see: Wang, J., and Feringa, B.L. (2011). Dynamic control of chiral space in a catalytic asymmetric reaction using a molecular motor Science *331*, 1429–1432.

76. Kassem, S., Lee, A.T.L., Leigh, D.A., Marcos, V., Palmer, L.I., and Pisano, S. (2017). Stereodivergent synthesis with a programmable molecular machine. Nature *549*, 374–378.

77. Dommaschk, M., Echavarren, J., Leigh, D.A., Marcos, V., and Singleton, T.A. (2019). Dynamic control of chiral space Through local symmetry breaking in a rotaxane organocatalyst. Angew. Chem. Int. Ed. *58*, 14955–14958.

78. Sluysmans, D., and Stoddart, J.F. (2019). The burgeoning of mechanically interlocked molecules in chemistry. Trends Chem. *1*, 185–197.

79. Heard, A.W., and Goldup, S.M. (2020). Simplicity in the design, operation and applications of mechanically interlocked molecular machines. ACS Cent. Sci.

Chem, Volume *6*

Supplemental Information

Synthesis of a Mechanically Planar Chiral Rotaxane Ligand for Enantioselective Catalysis

Andrew W. Heard and Stephen M. Goldup

1. General Experimental Information ... *3*
2. Synthesis and Characterisation of Catalysts [Au(6)(Cl)] ... *5*
Rotaxanes (S,R_{mp})-4 and (S,S_{mp})-4 ... 5
Rotaxane (R_{mp})-5 ... 19
Rotaxane (S_{mp})-5 ... 28
Rotaxane [Au((R_{mp})-6)(Cl)] ... 32
Rotaxane [Au((S_{mp})-6)(Cl)] ... 40
^{1}H NMR Stack Plot Demonstrating the binding of Cu^I to the Rotaxane Framework ... 42
3. Crystallographic Data: Assignment of Relative and Absolute Stereochemistry of (S,R_{mp})-4 and (S,S_{mp})-4 ... *43*
Rotaxane (S,R_{mp})-4 SCXRD Data (XRAY_ SRmp4.cif) ... 43
Rotaxane (S,S_{mp})-4 SCXRD Data (XRAY_ SSmp4.cif) ... 46
4. Synthesis of Cyclopropanation Substrates ... *51*
Substituted 2-methyl-3-butyn-2-yl benzoate general procedure ... 51
2-methyl-3-butyn-2-yl benzoate, 7 ... 51
2-methyl-3-butyn-2-yl pivaloate, S3' ... 53
2-methyl-3-butyn-2-yl phenylacetate, S4 ... 54
2-methyl-3-butyn-2-yl 4-trifluoromethylbenzoate, S5 ... 56
2-methyl-3-butyn-2-yl 4-methoxybenzoate, S6 ... 58
2-methyl-3-butyn-2-yl 4-tertbutylbenzoate, S7 ... 59
2-methyl-3-butyn-2-yl 3,5-ditertbutylbenzoate, S8 ... 61
5. Cyclopropanation Reactions ... *63*
Screening of reaction conditions ... 63
Determination of the absolute stereochemistry of cyclopropanes 9 ... 64
Cyclopropanation General Procedures ... 65
Cyclopropanes 9[6] ... 66
Cyclopropane 10 ... 72
Cyclopropanes 12 ... 80
Cyclopropanes 13[6] ... 86
Cyclopropanes 14 ... 90
Cyclopropanes 15 ... 94
Cyclopropanes 16 ... 102
Cyclopropanes 17 ... 106
Cyclopropanes 18 ... 113
6. Additional Reaction Scope ... *124*

Cycloisomerisation 1 .... **124**

Cycloisomerisation 2 .... **127**

Intramolecular Hydroamination of Allenes **130**

7. Molecular Modelling ***133***

Models of the reaction of ester 7 and styrene (8) mediated by [Au(PPh_3)(Cl)]............ **133**

Diastereomeric transition states for the reaction of 7 with 8 mediated by [Au(6)(Cl)] **134**

Transition states for the formation of cyclopropanes 13, 15 and 16 mediated by [Au(6)(Cl)] **137**

8. References ***139***

1. General Experimental Information

Unless otherwise stated, all reagents were purchased from commercial sources (Acros Organics, Alfa Aesar, Fisher Scientific, FluoroChem, Sigma Aldrich and VWR) and used without further purification. Styrene was purified by vacuum distillation prior to usage and stored under inert N_2 atmosphere. $[Cu(MeCN)_4]PF_6$ was prepared as described by Pigorsch and Köckerling.[1] Anhydrous solvents were purchased from Acros Organics. Petrol refers to the fraction of petroleum ether boiling in the range 40-60 °C. IPA refers to isopropanol. THF refers to tetrahydrofuran. EDTA-NH_3 solution refers to an aqueous solution of NH_3 (17% w/w) saturated with sodium-ethylenediaminetetraacetate. $CDCl_3$ (without stabilising agent) was distilled over $CaCl_2$ and K_2CO_3 prior to use. Compounds **1**,[2] **2**,[3] and **3**[4] were synthesised according to literature procedure. Unless otherwise stated, all reactions were performed in oven dried glassware under an inert N_2 atmosphere with purchased anhydrous solvents. Unless otherwise stated experiments carried out in sealed vessels were performed in CEM microwave vials, with crimped aluminium caps and PTFE septa.

Flash column chromatography was performed using Biotage Isolera-4 or Isolera-1 automated chromatography system. SiO_2 cartridges were purchased commercially from Teledyne Technologies, or Biotage (SNAP or ZIP, 50 µm irregular silica, default flow rates). Neutralised SiO_2 refers to ZIP cartridges which were eluted with petrol-NEt_3 (99 : 1, 5 column volumes), followed by petrol (5 column volumes). H_2O saturated SiO_2 refers to ZIP cartridges first eluted with H_2O saturated petrol-Et_2O-EtOAc (5 : 3 : 2 shaken with H_2O and the layers separated) followed by petrol-Et_2O-EtOAc (5 : 3 : 2) before loading the compound. Analytical TLC was performed on pre-coated silica gel plates on aluminum (0.25 mm thick, 60F254, Merck, Germany) and observed under UV light (254 nm) or visualised with $KMnO_4$ stain.

All melting points were determined using a Griffin apparatus and are uncorrected. NMR spectra were recorded on Bruker AV400 or AV500 instrument, at a constant temperature of 298 K. Chemical shifts are reported in parts per million from low to high field and referenced to residual solvent. Coupling constants (*J*) are reported in Hertz (Hz). Standard abbreviations indicating multiplicity were used as follows: m = multiplet, quint = quintet, q = quartet, t = triplet, d = doublet, s = singlet, app. = apparent, br = broad, sept = septet. Signal assignment was carried out using 2D NMR methods (COSY, NOESY, TOCSY, HSQC, HMBC or ^{31}P-^{1}H HMBC) where necessary. In some cases, complex multiplets with multiple contributing proton signals, exact assignment was not possible. In interlocked compounds, all proton signals corresponding to axle

components are in lower case, and all proton signals corresponding to the macrocycle components are in upper case. For mixtures of diastereomeric cyclopropanes, upper case is used to denote the major diastereoisomer, and lower case is used to denote the minor diastereoisomer. Low resolution mass spectrometry was carried out by the mass spectrometry services at University of Southampton (Waters TQD mass spectrometer equipped with a triple quadrupole analyser with UHPLC injection [BEH C18 column; MeCN-H_2O gradient {0.2% formic acid}]). High resolution mass spectrometry was carried out either by the mass spectrometry service at the University of Edinburgh (ThermoElectron MAT 900) or by the mass spectrometry services at the University of Southampton (MaXis, Bruker Daltonics, with a Time of Flight (TOF) analyser; samples were introduced to the mass spectrometer via a Dionex Ultimate 3000 autosampler and uHPLC pump in a gradient of 20% MeCN in hexane to 100% acetonitrile (0.2% formic acid) over 5-10 min at 0.6 mL/min; column: Acquity UPLC BEH C18 (Waters) 1.7 micron 50 × 2.1mm). Circular dichroism spectra were acquired on an Applied Photo-physics Chirascan spectropolarimeter, recorded using Applied Photophysics software Ver. 4.2.0 in dried spectroscopic grade $CHCl_3$, following overnight desiccation of the sample, at a concentration range of 0.1-1 10^{-4} M, in a quartz cell of 1 cm path length, at a temperature of 293 K.

Stereochemical purity was determined by Chiral Stationary Phase HPLC on a Waters Acquity Arc Instrument at 303 K, with *n*-hexane-isopropanol isocratic eluents. Regis Technologies (*S*,*S*)-Whelk-O1 (1-(3,5-dinitrobenzamido)-1,2,3,4-tetrahydrophenanthrene stationary phase), RegisPack (tris-(3,5-dimethylphenyl) carbamoyl amylose stationary phase) and RegisCell (tris-(3,5-dimethylphenyl) carbamoyl cellulose stationary phase) columns were used throughout (5 micron, column dimensions 25 cm x 4.6 mm). The absolute stereochemistry of mechanically planar chiral rotaxanes **4**-**6** was determined by X-ray crystallography of rotaxanes **4**, with the mechanical stereogenic unit assumed to be invariant through subsequent steps. Stereochemical labels were assigned using our established approach.[5] The absolute stereochemistry of cyclopropanes **9** was determined by comparison with the known stereochemical outcome of the reaction mediated by (*R*)-DTBM-SEGPHOS®$(AuCl)_2$.[6]

2. Synthesis and Characterisation of Catalysts [Au(6)(Cl)]

Rotaxanes (S,R_{mp})-**4** and (S,S_{mp})-**4**

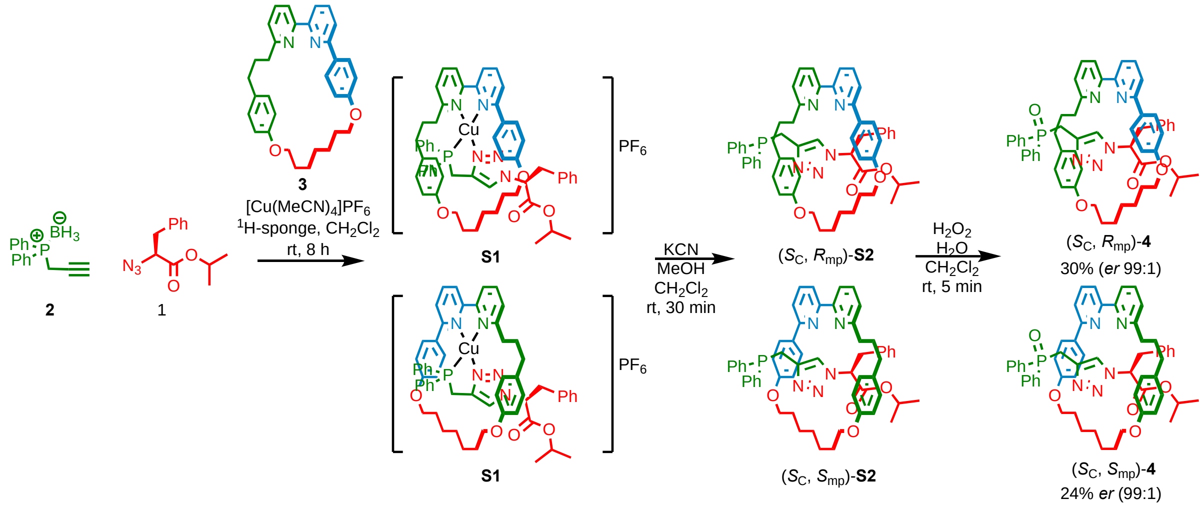

Scheme S1: Synthesis of (S,R_{mp})-**4** and (S,S_{mp})-**4**.

3 (200 mg, 0.418 mmol, 1.0 eq.), **2** (298 mg, 1.25 mmol, 3.0 eq.), **1** (292 mg, 1.25 mmol, 3.0 eq.), $[Cu(MeCN)_4]PF_6$ (150 mg, 0.401 mmol, 0.96 eq.) and ^{1}H-sponge (89.5 mg, 0.418 mmol, 1.0 eq.) were stirred in anhydrous CH_2Cl_2 (10 mL) at rt for 8 h under N_2. The orange solution was diluted with CH_2Cl_2 (10 mL), washed with sat. EDTA-NH_3 (25 mL) and then brine (25 mL). The combined aqueous washes were extracted with CH_2Cl_2 (3 x 25 mL). The combined organic extracts were dried ($MgSO_4$), and the solvent removed *in vacuo*. The residue was purified by column chromatography (neutralised SiO_2 (see general experimental), petrol-CH_2Cl_2 1 : 1 with 0→5% MeOH gradient), yielding an orange foam containing both diastereoisomers of **S1** (393 mg, 0.343 mmol, *dr* 1.0 : 1.1, 82%).

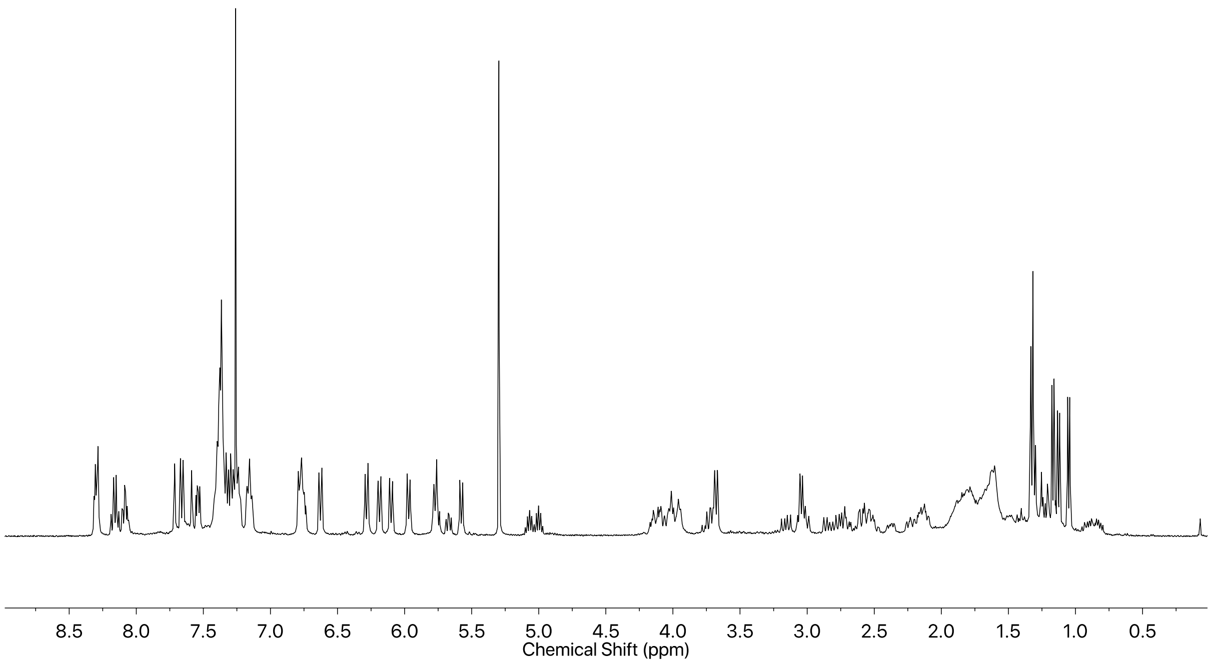

Figure S1: ^{1}H NMR (400 MHz, $CDCl_3$) of the diastereomeric mixture of Cu complexes **S1**.

The diastereomeric mixture of **S1** (393 mg, 0.343 mmol, 1.0 eq.) in CH_2Cl_2 (2.0 mL) was added to a solution of KCN (100 mg, 1.54 mmol, 4.5 eq.) in MeOH (2.0 mL), and the reaction mixture stirred for 30 min at rt. The solution was washed with brine (15 mL), aqueous H_2O_2 (35% w/w, 20 mL), brine (20 mL), and the combined aqueous washes were extracted with CH_2Cl_2 (3 x 15 mL). The combined organic phases were dried ($MgSO_4$) and the solvent removed *in vacuo* yielding a yellow foam (S,R_{mp})-**4**:(S,S_{mp})-**4** (295 mg, 0.309 mmol, *dr* 1.0 : 1.1, 90%). (S,R_{mp})-**4** and (S,S_{mp})-**4** were separated by column chromatography (H_2O saturated SiO_2, isocratic petrol-Et_2O-EtOAc 5 : 3 : 2) to give (S,R_{mp})-**4** (121 mg, 0.127 mmol, *er* = 99 : 1) and (S,S_{mp})-**4** (97 mg, 0.102 mmol, (S,R_{mp})-**4** : (R,S_{mp})-**4** : (S,S_{mp})-**4** = 0.6 : 1.0 : 98.4).

The absolute stereochemistry of the products was determined by SC-XRD (*vide infra* for a full discussion) and the stereolabels assigned based on our established approach using the priority atoms A-D indicated below. Stereochemical purity was determined by HPLC using racemic samples of rotaxanes **4** (synthesised as above) for comparison (**Figure S9**, **Figure S10**). The stereochemical purity of (S,R_{mp})-**4** was ultimately confirmed by analysis of its derivative, (R_{mp})-**5** (**Figure S32**), due to better HPLC peak separation, although it should be noted that direct HPLC analysis of (S,R_{mp})-**4** (**Figure S11**) is consistent with this value.

(S,R_{mp})-**4**

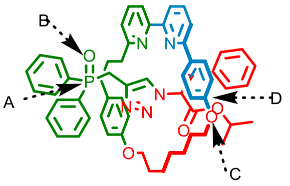

(S,R_{mp})-**4**

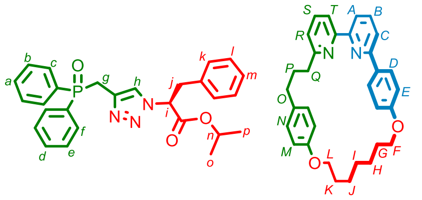

(S,R_{mp})-**4**

δ_{H} ($CDCl_3$, 400 MHz) 9.49 (1H, d, J = 2.0, $\boldsymbol{H}_{\mathrm{h}}$), 7.81 (1H, t, J = 7.8, $\boldsymbol{H}_{\mathrm{S}}$), 7.77 (2H, td, J = 9.0, 1.4, $\boldsymbol{H}_{\mathrm{f}}$), 7.76 (1H, t, J = 7.8, $\boldsymbol{H}_{\mathrm{B}}$), 7.71 (2H, ddd, J = 11.4, 7.4, 1.5, $\boldsymbol{H}_{\mathrm{c}}$), 7.63 (1H, d, J = 7.8, $\boldsymbol{H}_{\mathrm{R}}$), 7.59 (1H, d, J = 7.8, $\boldsymbol{H}_{\mathrm{T}}$), 7.44 (2H, ddd, J = 8.3, 7.4, 1.4, $\boldsymbol{H}_{\mathrm{e}}$), 7.41 (1H, d, J = 7.7, $\boldsymbol{H}_{\mathrm{C}}$), 7.38 (1H, m, $\boldsymbol{H}_{\mathrm{d}}$), 7.32 (2H, dt, J = 8.8, 2.5, $\boldsymbol{H}_{\mathrm{D}}$), 7.28 (1H, d, J = 7.8, $\boldsymbol{H}_{\mathrm{A}}$), 7.20 (1H, td, J = 7.4, 1.4, $\boldsymbol{H}_{\mathrm{a}}$), 7.12 (2H, td, J = 7.4, 2.6, $\boldsymbol{H}_{\mathrm{b}}$), 6.80 (2H, dt, J = 8.7, 2.3, $\boldsymbol{H}_{\mathrm{N}}$), 7.08-7.02 (3H, m, $\boldsymbol{H}_{\mathrm{l}}$, $\boldsymbol{H}_{\mathrm{m}}$), 6.72 (2H, dd, J = 7.0, 2.4, $\boldsymbol{H}_{\mathrm{k}}$), 6.48 (2H, dt, J = 8.6, 2.0, $\boldsymbol{H}_{\mathrm{M}}$), 6.47 (2H, dt, J = 8.8, 2.4, $\boldsymbol{H}_{\mathrm{E}}$), 4.73 (1H, dd, J = 12.4, 4.7, $\boldsymbol{H}_{\mathrm{i}}$), 4.51 (1H, sept., J = 6.2, $\boldsymbol{H}_{\mathrm{n}}$), 4.32 (1H, app. q (ddd), J = 7.4, $\boldsymbol{H}_{\mathrm{Q}}$), 3.90 (2H, m, $\boldsymbol{H}_{\mathrm{F}}$, $\boldsymbol{H}_{\mathrm{F'}}$), 3.82 (1H, dt, J = 7.8, 4.1, $\boldsymbol{H}_{\mathrm{Q'}}$), 3.57 (1H, dd, J = 15.9, 13.6, $\boldsymbol{H}_{\mathrm{g}}$), 3.13 (1H, t(dd), J = 12.8, $\boldsymbol{H}_{\mathrm{j}}$), 2.99 (1H, dd, J = 15.9, 11.1, $\boldsymbol{H}_{\mathrm{g'}}$), 2.80-2.71 (2H, m, $\boldsymbol{H}_{\mathrm{O}}$, $\boldsymbol{H}_{\mathrm{O'}}$), 2.64 (2H, td, J = 8.6, 3.8, $\boldsymbol{H}_{\mathrm{L}}$, $\boldsymbol{H}_{\mathrm{L'}}$), 2.22 (1H, dd, J = 13.5, 4.7, $\boldsymbol{H}_{\mathrm{j'}}$), 2.07-1.95 (2H, m, $\boldsymbol{H}_{\mathrm{P}}$, $\boldsymbol{H}_{\mathrm{K}}$), 1.92-1.73 (4H, m, $\boldsymbol{H}_{\mathrm{P'}}$, $\boldsymbol{H}_{\mathrm{K'}}$, $\boldsymbol{H}_{\mathrm{J}}$, $\boldsymbol{H}_{\mathrm{J'}}$), 1.65-1.42 (2H, m, $\boldsymbol{H}_{\mathrm{G}}$, $\boldsymbol{H}_{\mathrm{G'}}$), 0.95-0.70 (4H, m, $\boldsymbol{H}_{\mathrm{H}}$, $\boldsymbol{H}_{\mathrm{H'}}$, $\boldsymbol{H}_{\mathrm{I}}$, $\boldsymbol{H}_{\mathrm{I'}}$), 0.69 (3H, d, J = 6.3, $\boldsymbol{H}_{\mathrm{p}}$), 0.44 (3H, d, J = 6.3, $\boldsymbol{H}_{\mathrm{o}}$).

δ_{C} ($CDCl_3$, 101 MHz) 169.0, 163.0, 159.2, 158.8, 157.7, 157.5, 157.0, 137.2, 137.0, 136.0, 135.6 (d, J = 7.2), 134.5 (d, J = 79.0), 132.5 (d, J = 78.8), 131.8, 131.5 (d, J = 2.6), 131.3 (d, J = 2.6), 131.2 (d, J = 3.4), 131.1 (d, J = 3.3), 130.6, 129.4, 129.3, 128.9, 128.7 (d, J = 11.6), 128.4 (d, J = 11.6), 128.1, 127.4 (d, J = 5.3), 126.5, 121.9, 120.3, 120.0, 119.5, 115.1, 114.3, 68.6, 67.9, 66.2, 62.3, 37.1, 36.4, 35.1, 31.3, 28.8, 28.7, 28.0 (d, J = 69.1), 25.5, 25.1, 24.0, 21.2, 20.9.

$\delta_{\mathrm{31P\{1H\}}}$ ($CDCl_3$, 202 MHz) 28.7.

HR-ESI-MS m/z = 952.4564 $[M + H]^+$ (calc. m/z for $C_{59}H_{62}N_5O_5P$ 952.4561).

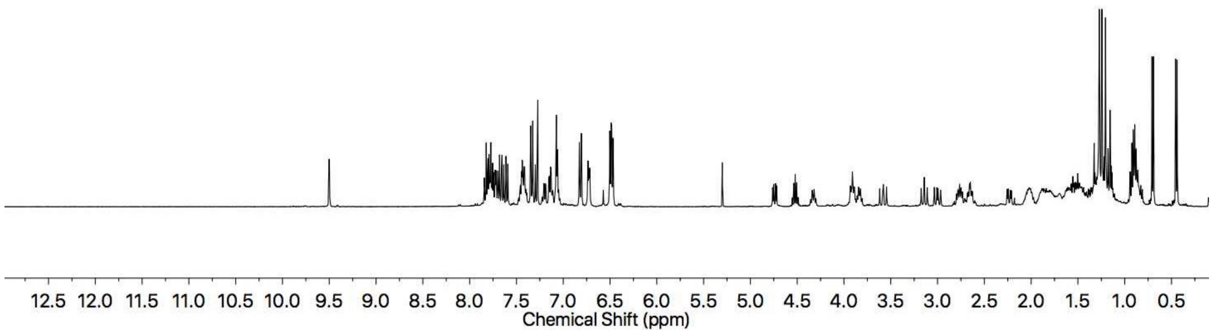

Figure S2: ^{1}H NMR (400 MHz, $CDCl_3$) of (S,R_{mp})-**4**.

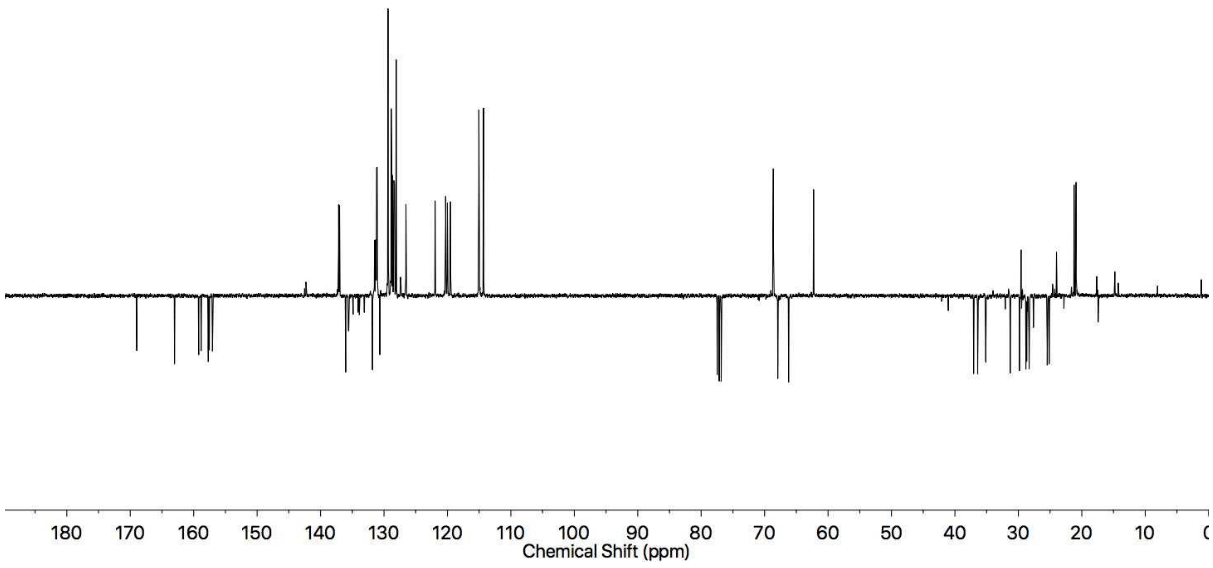

Figure S3: JMOD NMR (101 MHz, $CDCl_3$) of (S,R_{mp})-**4**.

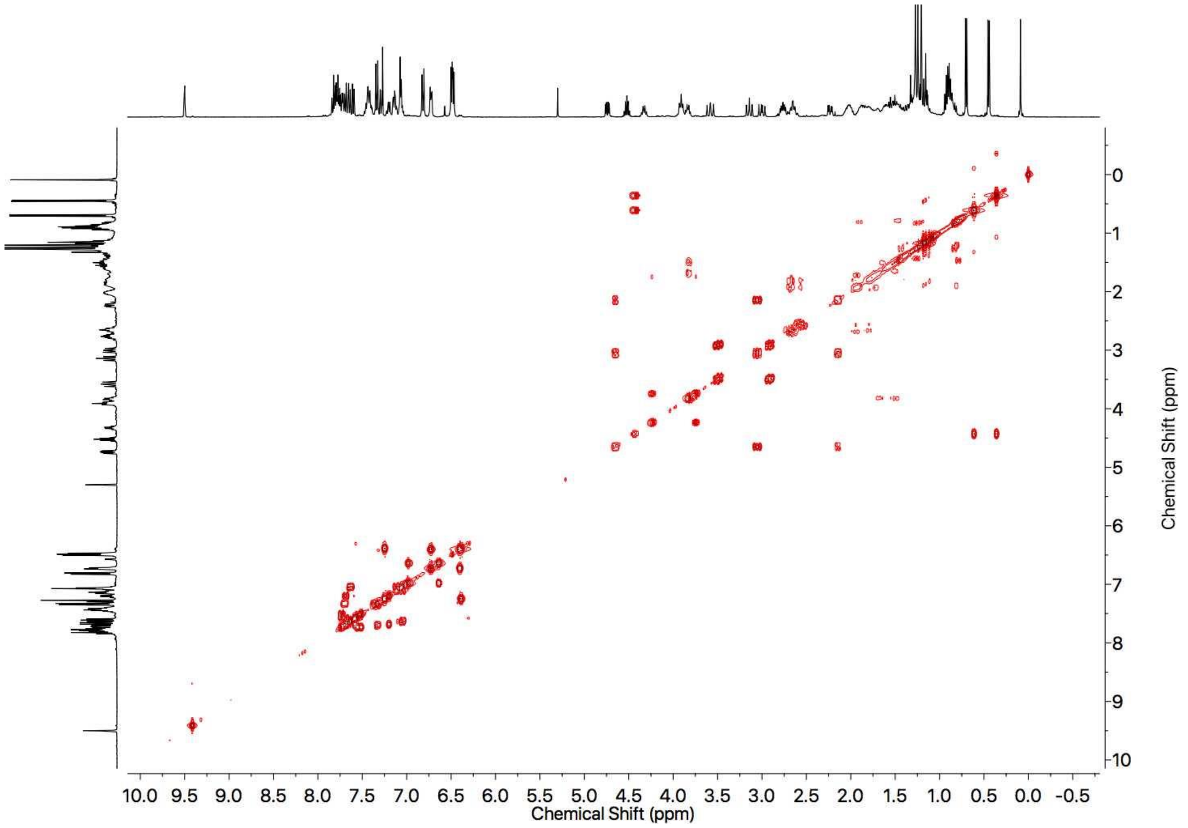

Figure S4: COSY NMR ($CDCl_3$) of (*S*,*R*$_{mp}$)-**4**.

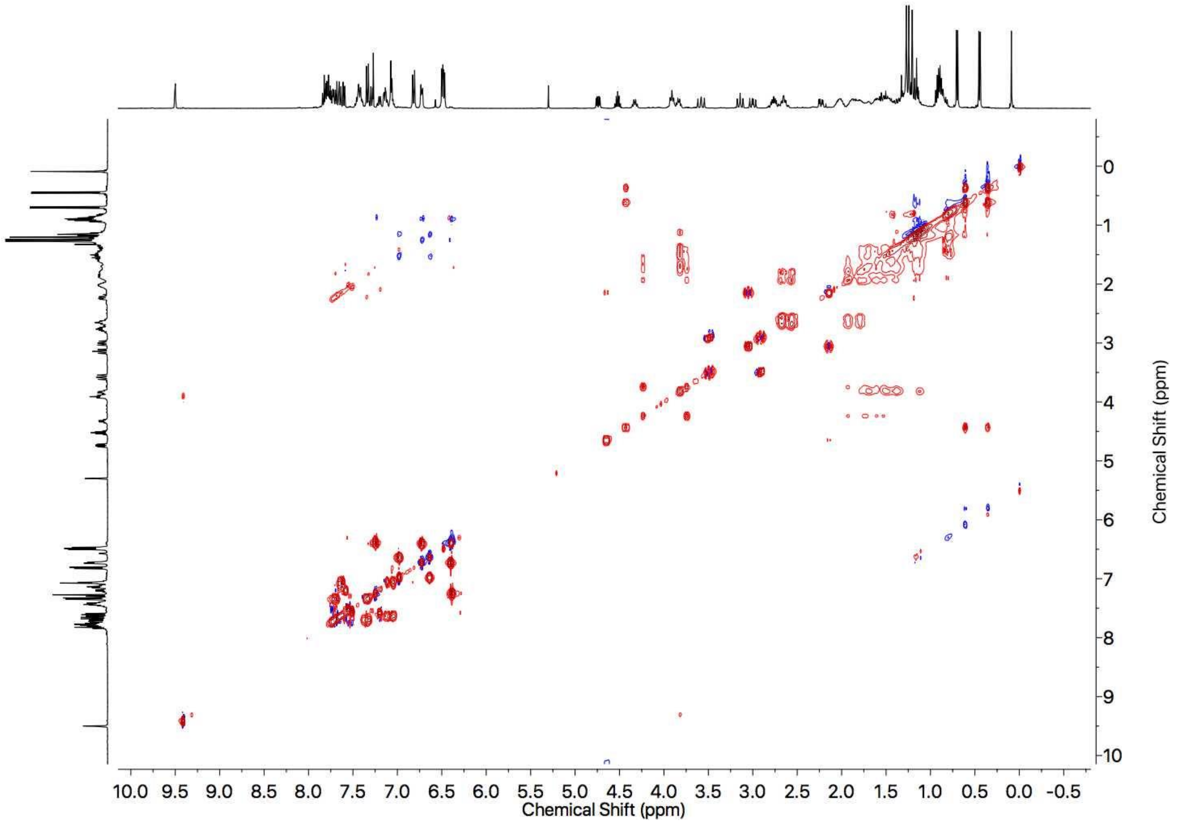

Figure S5: TOCSY NMR ($CDCl_3$) of (*S*,*R*$_{mp}$)-**4**.

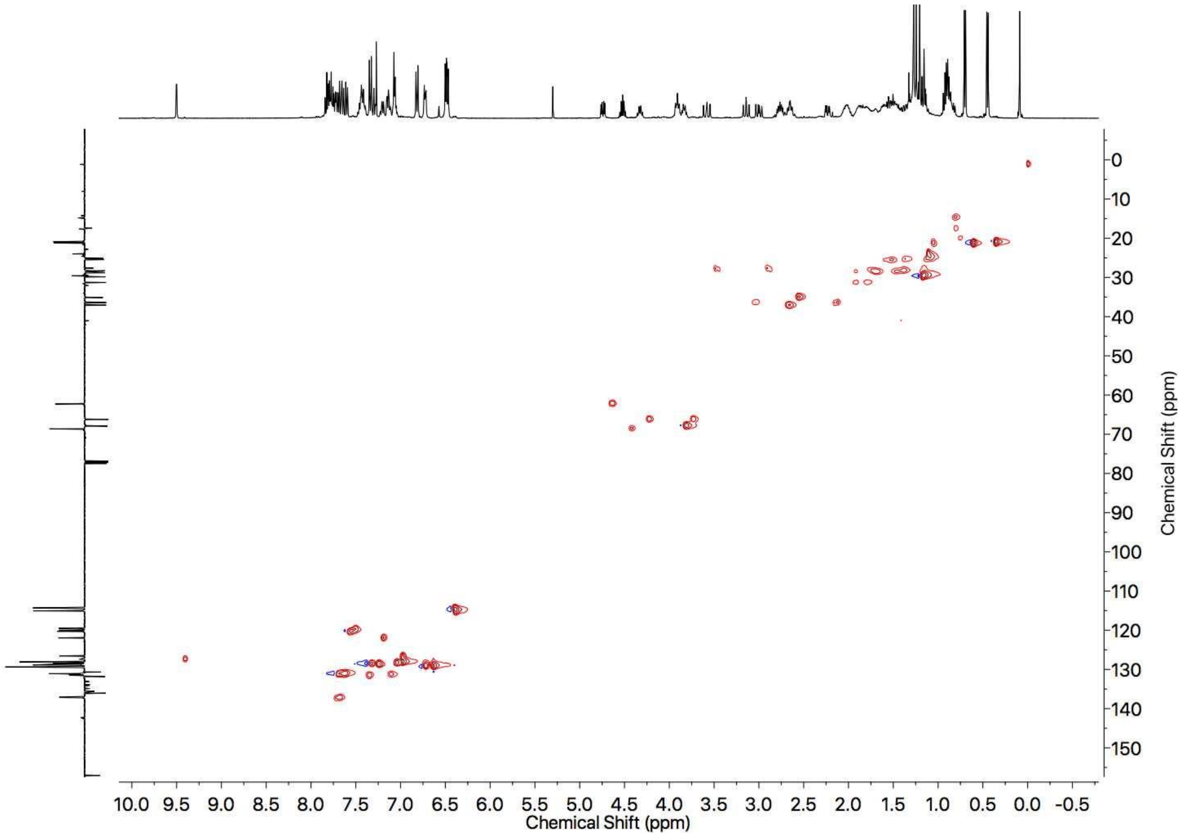

Figure S6: HSQC NMR ($CDCl_3$) of (*S*,*R*$_{mp}$)-**4**.

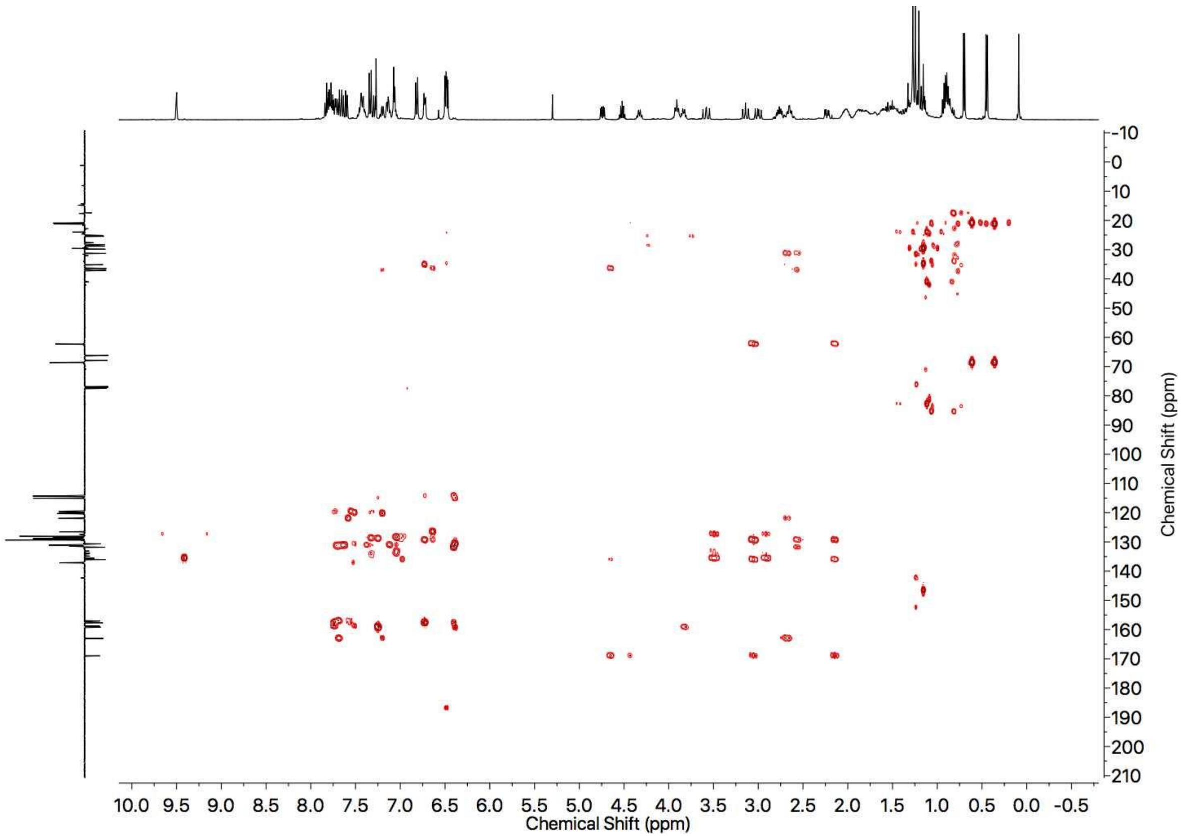

Figure S7: HMBC NMR ($CDCl_3$) of (*S*,*R*$_{mp}$)-**4**.

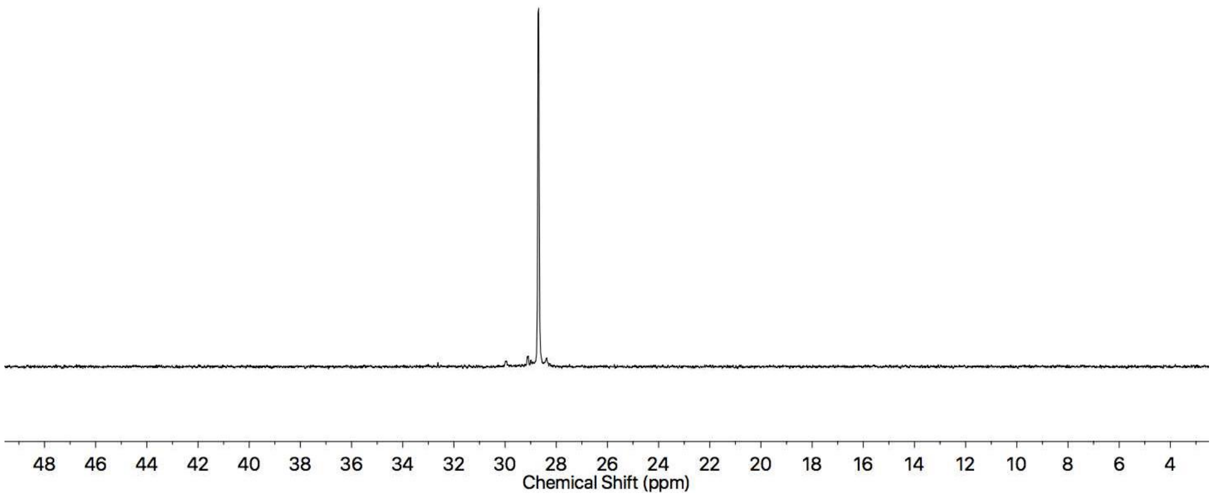

Figure S8: $^{31}P\{^{1}H\}$ NMR (202 MHz, $CDCl_3$) of (*S*,*R*$_{mp}$)-**4**.

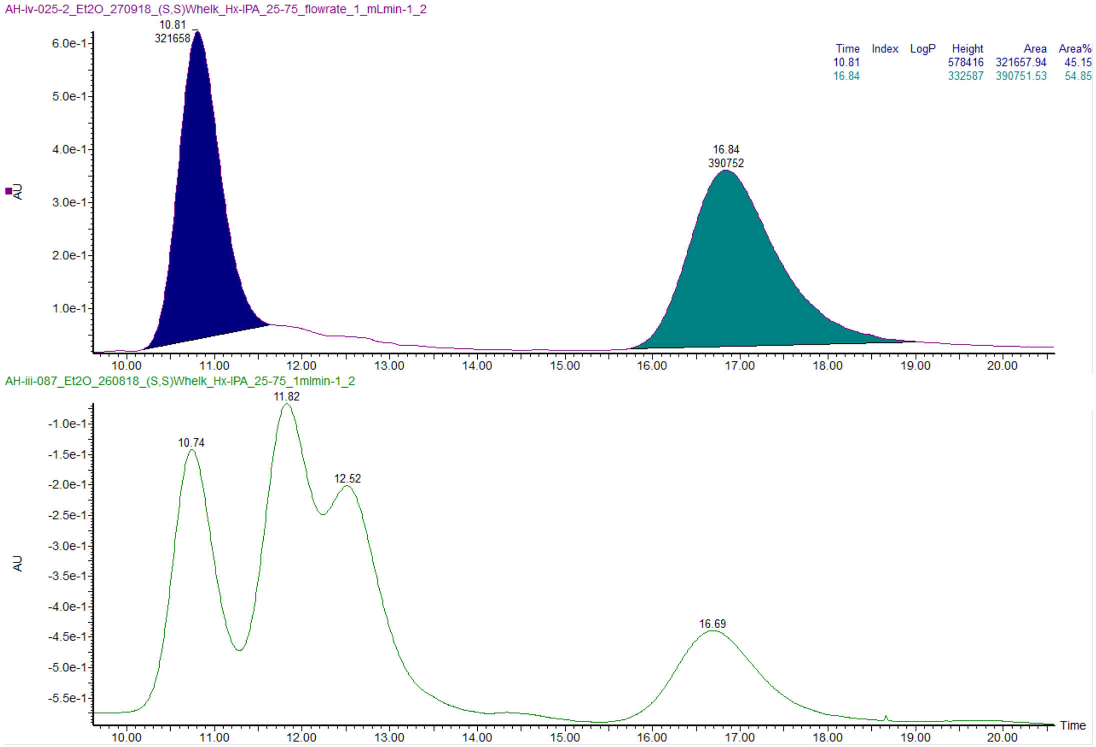

Figure S9: Chiral Stationary Phase HPLC ((*S*,*S*)Whelk, isocratic *n*-hexane-isopropanol 25 : 75, 303 K, load solvent Et_2O, 5 µL injection, flowrate 1 mLmin^{-1}) of the crude mixture of rotaxanes **4** (top) and the equivalent racemate (bottom). Retention times (min): (*S*,*R*$_{mp}$)-**4** 10.7, (*R*,*S*$_{mp}$)-**4** 11.8, (*R*,*R*$_{mp}$)-**4** 12.5, (*S*,*S*$_{mp}$)-**4** 16.7.

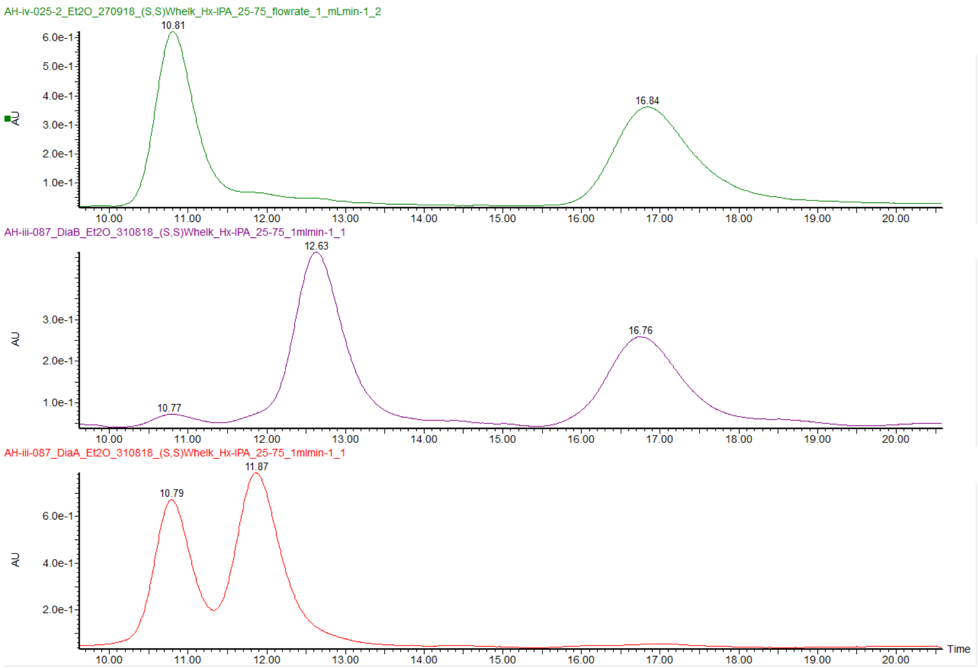

Figure S10: Chiral Stationary Phase HPLC ((*S*,*S*)Whelk, isocratic *n*-hexane-isopropanol 25 : 75, 303 K, load solvent Et_2O, 5 µL injection, flowrate 1 $mLmin^{-1}$) of the crude mixture of rotaxanes **4** (top) and the racemates of highly diastereomerically enriched samples (middle and bottom). Retention times (min): (*S*,R_{mp})-**4** 10.7, (*R*,S_{mp})-**4** 11.8, (*R*,R_{mp})-**4** 12.5, (*S*,S_{mp})-**4** 16.7.

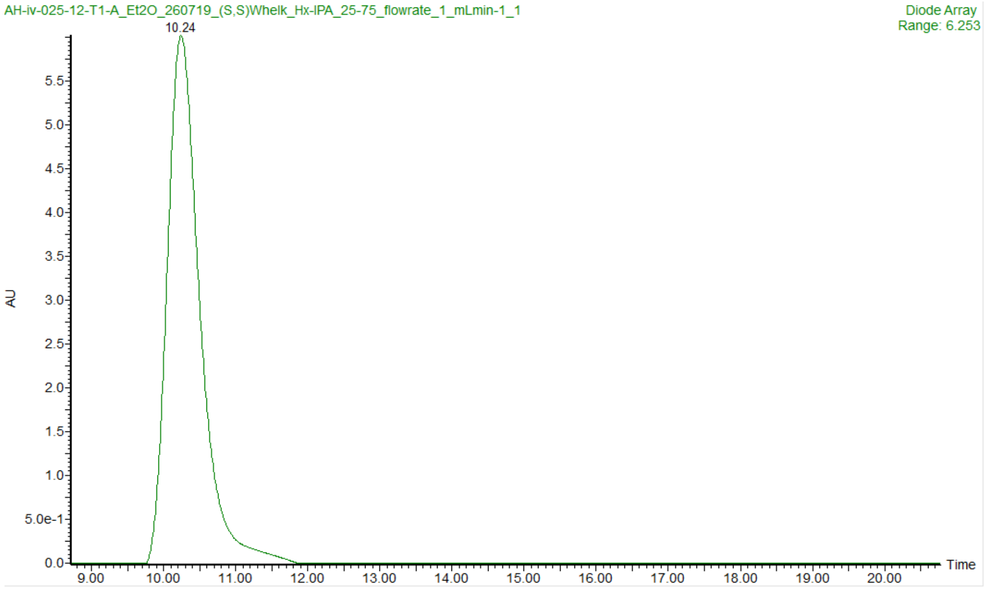

Figure S11: Chiral Stationary Phase HPLC ((*S*,*S*)Whelk, isocratic *n*-hexane-isopropanol 25 : 75, 303 K, load solvent Et_2O, 5 µL injection, flowrate 1 $mLmin^{-1}$) of 99 : 1 *er* (*S*,R_{mp})-**4.** Due to overlap of the peaks, the stereochemical purity is inferred from analysis of (*S*,R_{mp})-**5**. The HPLC trace shown here is consistent with this.

(S,S_{mp})-**4**

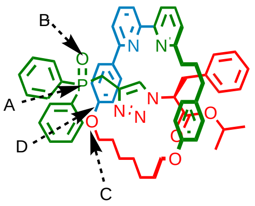

(S,S_{mp})-**4**

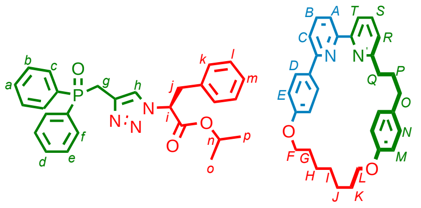

(S,S_{mp})-**4**

δ_H ($CDCl_3$, 400 MHz) 9.54 (1H, d, J = 1.8, $\boldsymbol{H}_h$), 7.87-7.79 (4H, m, $\boldsymbol{H}_b$, $\boldsymbol{H}_e$), 7.83 (1H, t, J = 7.6, $\boldsymbol{H}_B$), 7.75 (1H, t, J = 7.6, $\boldsymbol{H}_S$), 7.65 (1H, d, J = 7.6, $\boldsymbol{H}_A$), 7.62 (1H, d, J = 7.6, $\boldsymbol{H}_T$), 7.59 (1H, d, J = 8.0, $\boldsymbol{H}_a$ or $\boldsymbol{H}_d$), 7.44-7.35 (4H, m, $\boldsymbol{H}_c$, $\boldsymbol{H}_f$), 7.33 (1H, d, J = 7.7, $\boldsymbol{H}_C$), 7.32 (2H, dt, J = 8.9, 2.4, $\boldsymbol{H}_D$), 7.31-7.29 (1H, m, $\boldsymbol{H}_a$ or $\boldsymbol{H}_d$), 7.27 (1H, d, J = 7.6, $\boldsymbol{H}_R$), 6.91 (2H, d, J = 8.8, $\boldsymbol{H}_N$), 7.08-7.01 (3H, m, $\boldsymbol{H}_l$, $\boldsymbol{H}_m$), 6.53 (2H, d, J = 8.9, $\boldsymbol{H}_M$), 6.52 (2H, dd, J = 7.5, 1.9, $\boldsymbol{H}_k$), 6.31 (2H, dt, J = 9.1, 2.2, $\boldsymbol{H}_E$), 4.64 (1H, sept., J = 6.2, $\boldsymbol{H}_n$), 3.96 (1H, app. q., J = 6.7, $\boldsymbol{H}_F$), 3.82 (2H, t, J = 7.6, $\boldsymbol{H}_L$), 3.74 (1H, t, J = 6.6, $\boldsymbol{H}_{F'}$), 3.71 (1H, dd, J = 11.9, 4.3, $\boldsymbol{H}_i$), 3.56 (1H, dd, J = 15.1, 12.0, $\boldsymbol{H}_g$), 3.52 (1H, dd, J = 11.9, 4.3, $\boldsymbol{H}_j$), 3.18 (1H, t, J = 15.1, $\boldsymbol{H}_{g'}$), 2.92 (1H, t, J = 11.9, $\boldsymbol{H}_{j'}$), 2.80 (2H, dt, J = 14.0, 3.6, $\boldsymbol{H}_Q$, $\boldsymbol{H}_{Q'}$), 2.73 (1H, dt, J = 13.6, 5.0, $\boldsymbol{H}_O$), 2.58 (1H, dt, J = 13.6, 6.8, $\boldsymbol{H}_O$'), 2.00-1.87 (3H, m, $\boldsymbol{H}_P$, $\boldsymbol{H}_{P'}$, $\boldsymbol{H}_G$), 1.80-1.46 (5H, m, $\boldsymbol{H}_{G'}$, $\boldsymbol{H}_J$, $\boldsymbol{H}_{J'}$), 1.31-1.10 (2H, m, $\boldsymbol{H}_K$, $\boldsymbol{H}_{K'}$), 0.94-0.79 (4H, m, $\boldsymbol{H}_H$, $\boldsymbol{H}_{H'}$, $\boldsymbol{H}_I$, $\boldsymbol{H}_{I'}$),0.99 (3H, d, J = 6.2, $\boldsymbol{H}_p$), 0.63 (3H, d, J = 6.3, $\boldsymbol{H}_o$).

δ_C ($CDCl_3$, 101 MHz) 167.8, 163.1, 159.4, 158.9, 157.6, 157.2, 137.1 (d, J = 3.4), 135.7 (d, J = 7.4), 135.5, 134.3 (d, J = 11.8), 133.3 (d, J = 12.5), 131.5, 131.4, 131.3 (d, J = 2.7) 131.3, 131.1 (d, J = 9.8), 130.9, 129.1, 129.1, 128.5 (d, J = 7.4), 128.4 (d, J = 7.4), 128.3, 128.1, 126.5, 126.2 (d, J = 6.3), 122.5, 120.2 (d, J = 17.6), 119.4, 115.6, 114.0, 68.7, 68.3, 66.0, 63.3, 37.3, 37.3, 35.1, 32.6, 29.7, 29.4, 29.3, 28.5, 28.3 (d, J = 22.5), 27.8, 25.0, 24.7, 23.9, 21.5, 21.1.

$\delta_{31P\{1H\}}$ ($CDCl_3$, 202 MHz) 29.8.

HR-ESI-MS m/z = 952.4564 $[M + H]^+$ (calc. m/z for $C_{59}H_{62}N_5O_5P$ 952.4561).

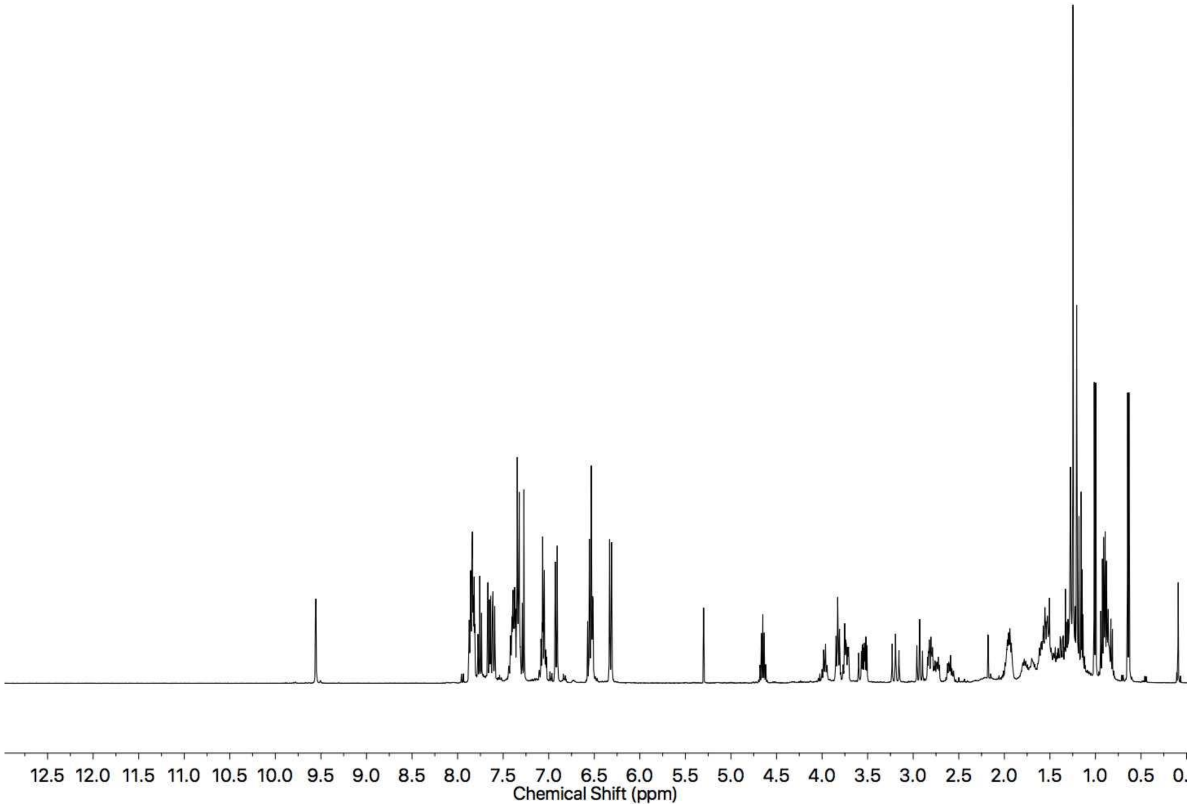

Figure S12: ^{1}H NMR (400 MHz, $CDCl_3$) of (S,S_{mp})-**4**.

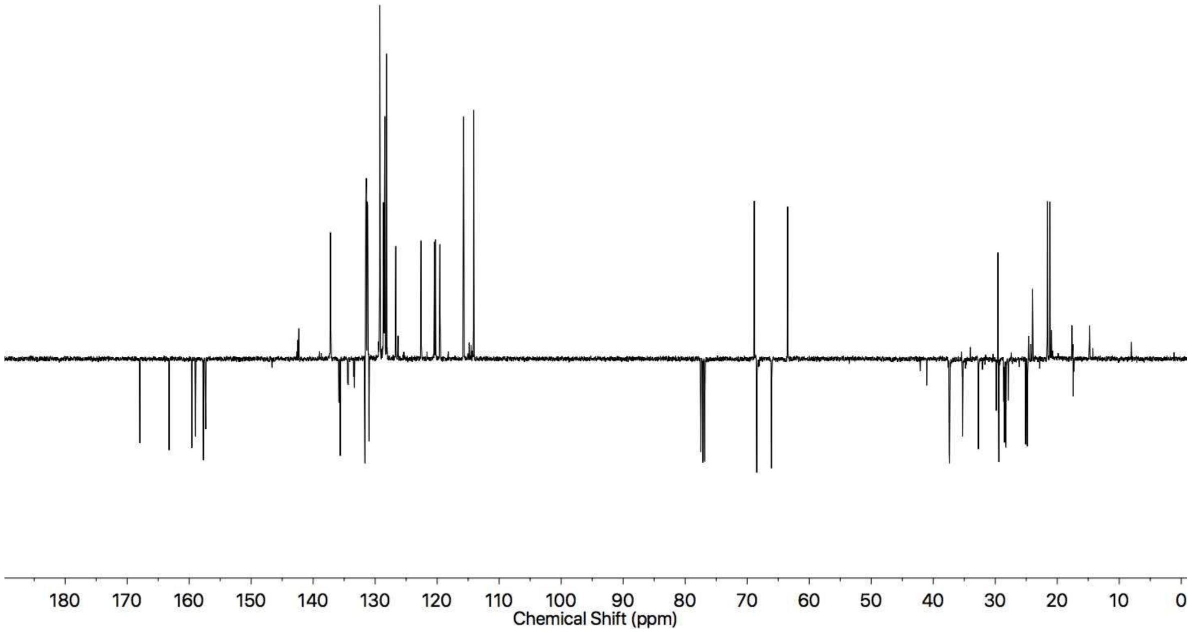

Figure S13: JMOD NMR (101 MHz, $CDCl_3$) of (S,S_{mp})-**4**.

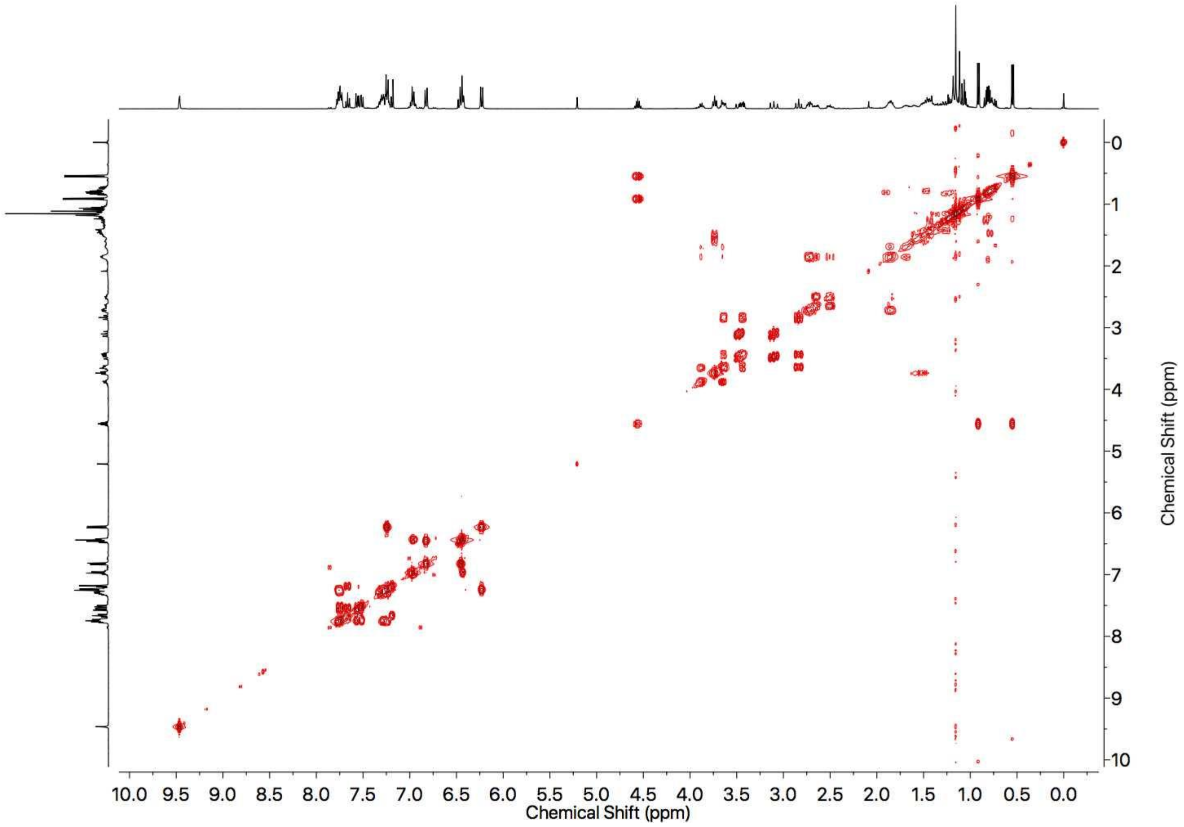

Figure S14: COSY NMR ($CDCl_3$) of (*S*,*S*mp)-**4**.

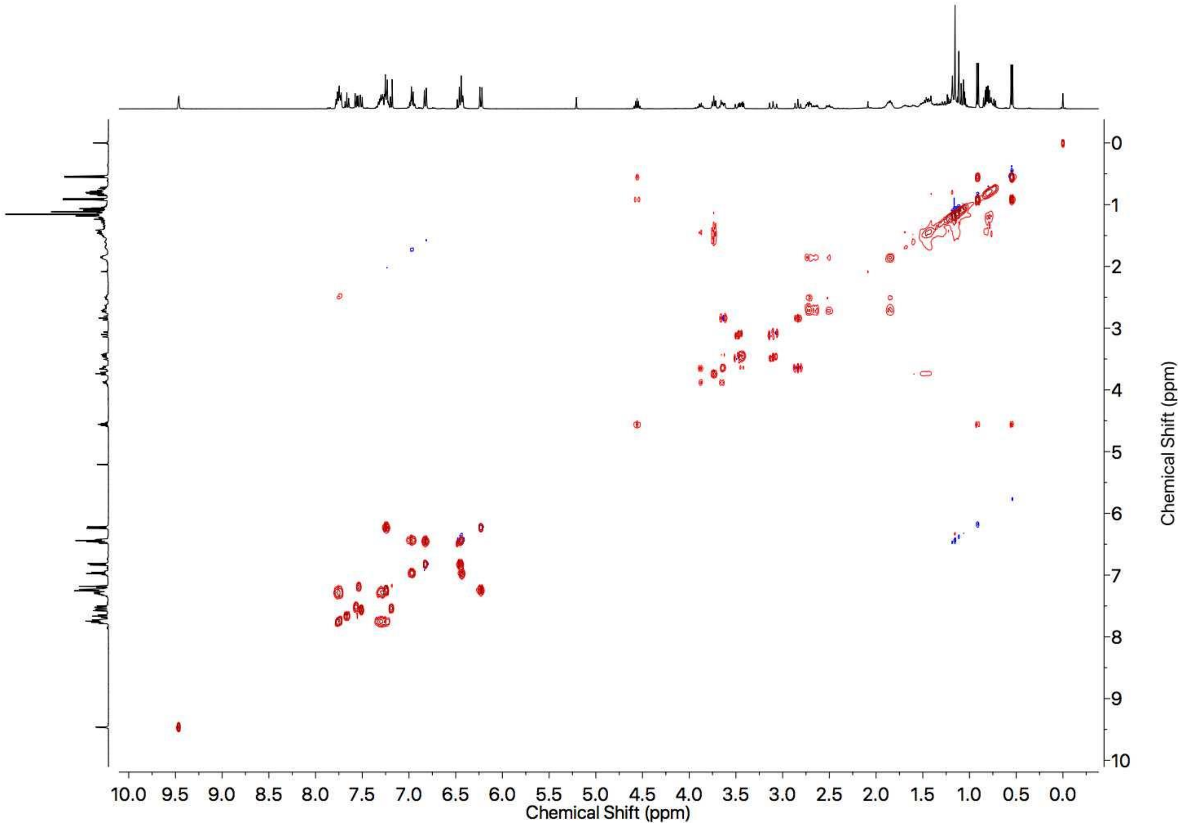

Figure S15: TOCSY NMR ($CDCl_3$) of (*S*,*S*mp)-**4**.

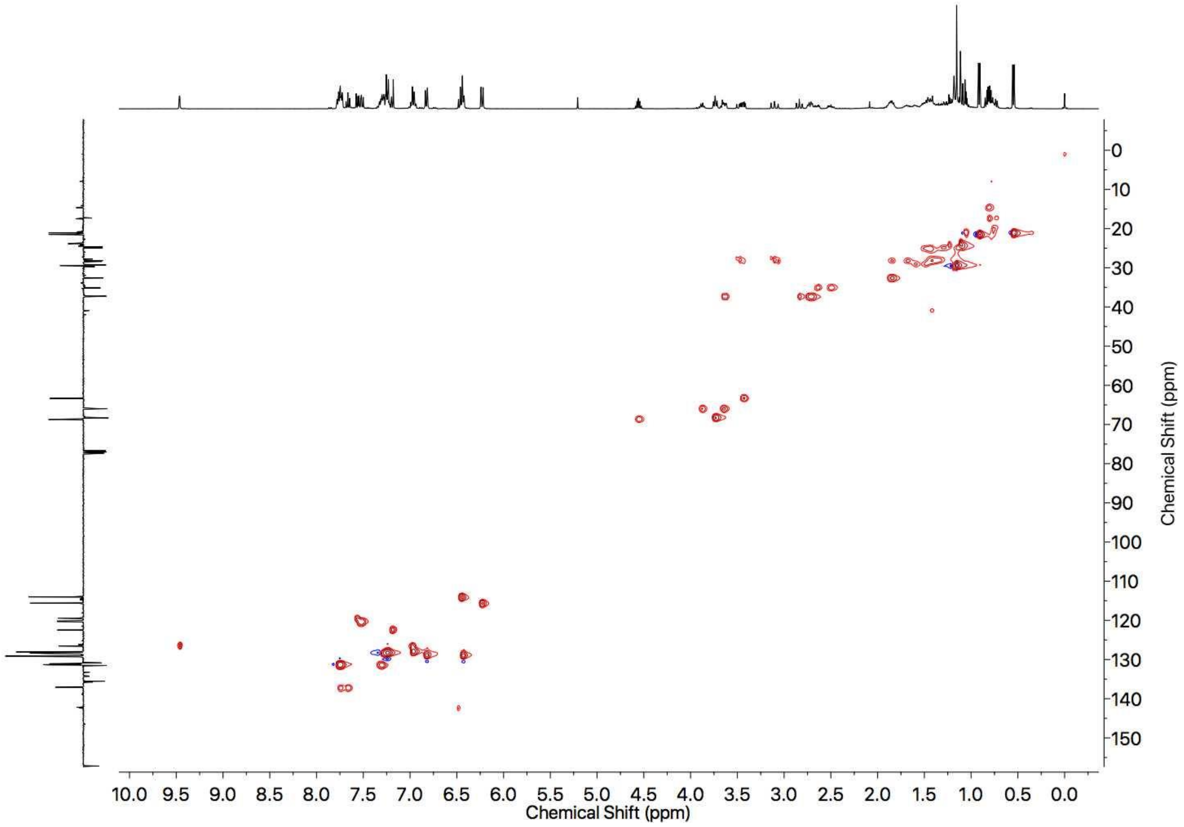

Figure S16: HSQC NMR ($CDCl_3$) of (*S*,*S*$_{mp}$)-**4**.

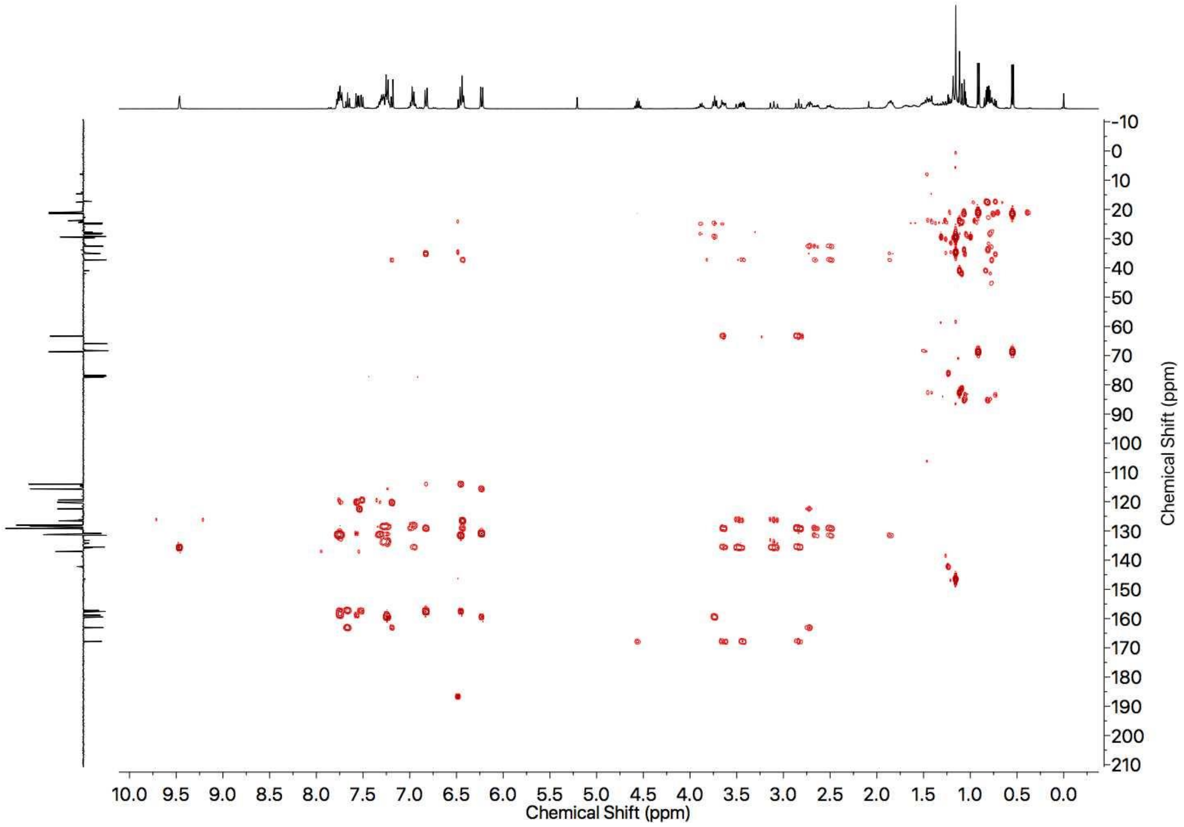

Figure S17: HMBC NMR ($CDCl_3$) of (*S*,*S*$_{mp}$)-**4**.

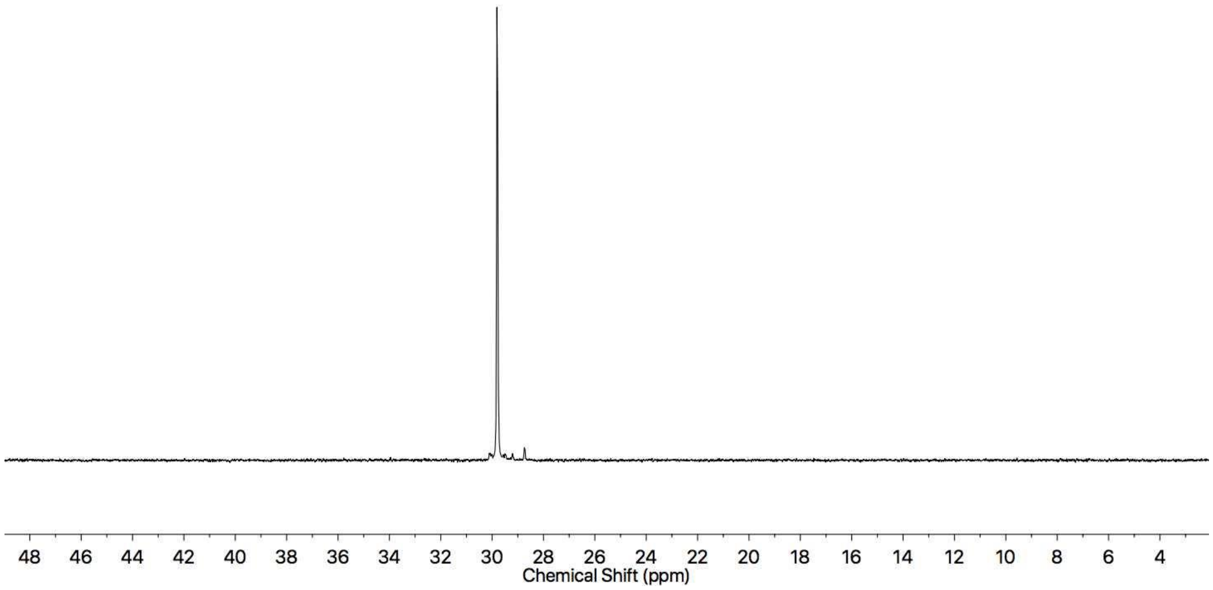

Figure S18: $^{31}P\{^{1}H\}$ NMR (202 MHz, $CDCl_3$) of (*S*,*S*$_{mp}$)-**4**.

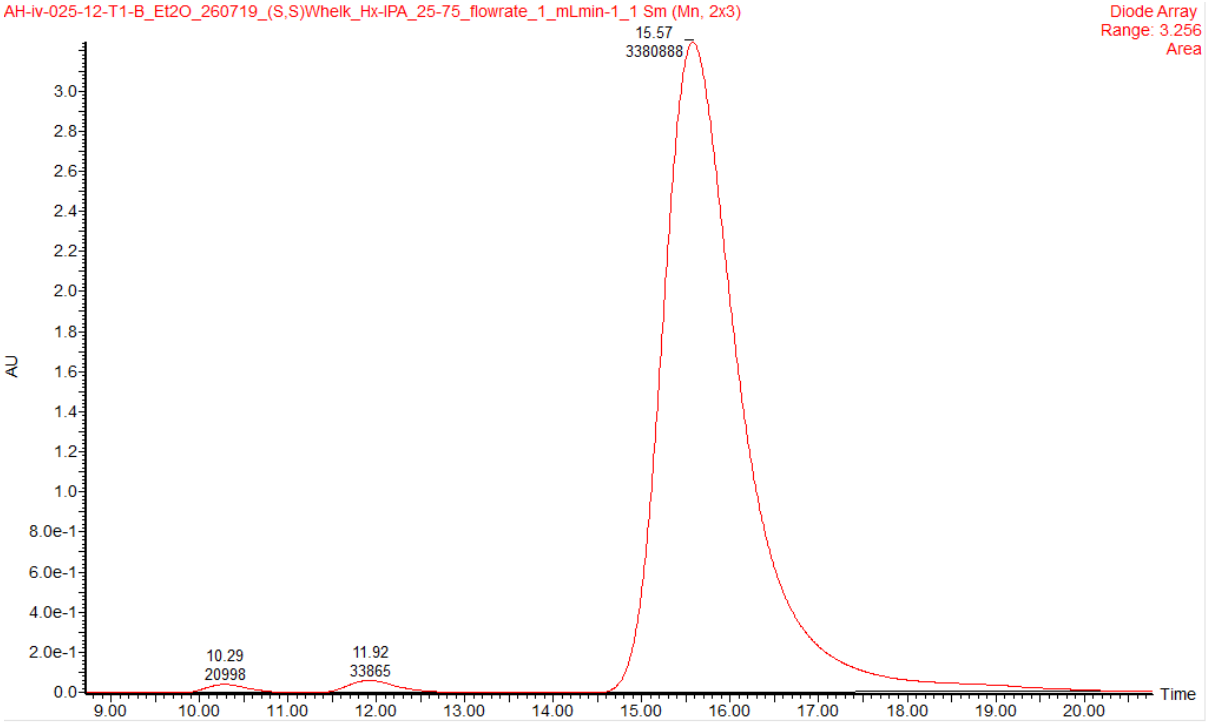

Figure S19: Chiral Stationary Phase HPLC ((*S*,*S*)Whelk, isocratic *n*-hexane-isopropanol 25 : 75, 303 K, load solvent Et_2O, 5 µL injection, flowrate 1 mLmin^{-1}). (*S*,*R*$_{mp}$)-**4** : (*R*,*S*$_{mp}$)-**4** : (*S*,*S*$_{mp}$)-**4**, 0.6 : 1.0 : 98.4. Mechanically planar stereogenic element ratio (*R*$_{mp}$) : (*S*$_{mp}$) 1 : 99.

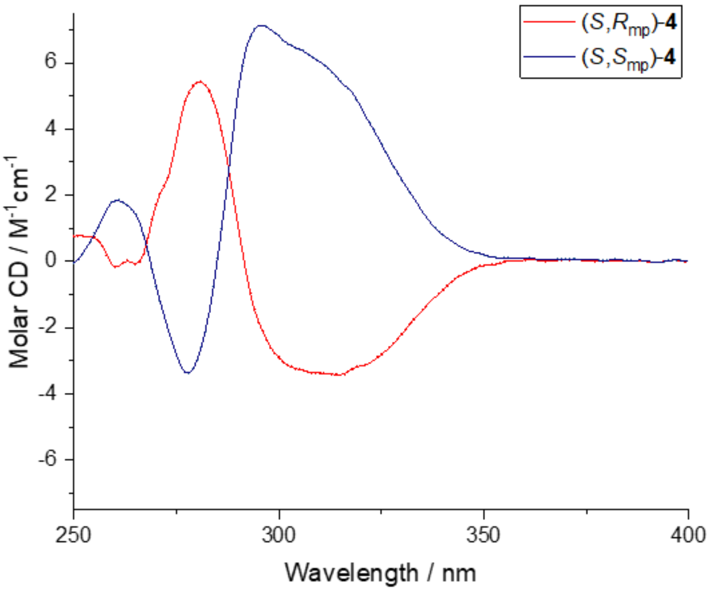

Figure S20: Circular Dichroism spectra of (*S*,*R*$_{mp}$)-**4** (53.4 µM, 99 : 1 *er*) and (*S*,*S*$_{mp}$)-**4** (58.8 µM, (*S*,*S*$_{mp}$)-**4** : (*R*,*S*$_{mp}$)-**4** : (*S*,*R*$_{mp}$)-**4**, 98.4 : 1.0 : 0.6) at 293 K in $CHCl_3$.

Rotaxane (R_{mp})-**5**

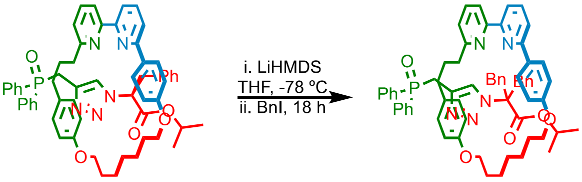

(S_C, R_{mp})-**4** (R_{mp})-**5** 81%, *ee* 98%

Scheme S2: Synthesis of (R_{mp})-**5**.

(S,R_{mp})-**4** (61.4 mg, 0.064 mmol, 1.0 eq.) was dissolved in anhydrous THF (3 mL) and transferred into a dry CEM MW vial under N_2. The solution was cooled to -78 °C and stirred for 20 min. Lithium bis(trimethylsilyl)amide (1 M in THF, 0.32 mL, 0.32 mmol, 5.0 eq.) was added to the reaction mixture and stirred for 10 min. Benzyl iodide (1 M in THF, 0.65 mL, 0.65 mmol, 10.0 eq.) was added. The reaction was allowed to warm to rt and stirred for 18 h. The reaction mixture was diluted with saturated NH_4Cl (30 mL) and extracted with CH_2Cl_2 (3 x 20 mL). The combined organic extracts were dried ($MgSO_4$) and solvent removed *in vacuo*. The residue was purified by column chromatography (SiO_2, petrol-EtOAc 0→50%) to give a yellow foam product (R_{mp})-**5** (53.7 mg, 0.051 mmol, 81%, *er* 99 : 1). *Enantiopurity was assessed by chiral stationary phase HPLC*. The absolute mechanical stereochemistry was inferred from that of the starting materials and the sterolabels assigned based on our established approach[5] using the priority atoms indicated below.

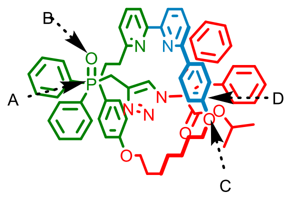

(R_{mp})-**5**

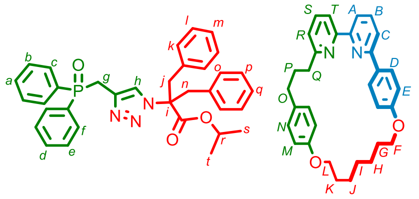

(R_{mp})-**5**

δ_H ($CDCl_3$, 400 MHz) 9.48 (1H, d, J = 1.6, H_h), 7.77 (1H, t, J = 8.1, H_S), 7.74 (1H, t, J = 8.1, H_B), 7.64 (4H, ddd, J = 8.2, 7.7, 2.3, H_c, H_f), 7.58 (1H, d, J = 8.1, H_T), 7.54 (1H, d, J = 7.8, H_A), 7.52 (2H, d, J = 8.5, H_D), 7.36 (4H, td, J = 7.6, 2.7, H_b, H_e), 7.29-7.23 (4H, m, H_a, H_d, H_R, H_C), 7.14-7.09 (3H, m, H_l, -H_m), 7.06-7.01 (3H, m, H_p, H_q), 6.90 (2H, d, J = 8.5, H_N), 6.89 (2H, dd, J = 7.2, 2.1, H_k), 6.79 (2H, dd, J = 7.1, 2.6, H_o), 6.49 (2H, d, J = 8.5, H_E), 6.35 (2H, d, J = 8.3, H_M), 4.60 (1H, sept., J = 6.2, H_r), 3.45 (1H, d, J = 15.0, H_g), 3.93-3.81 (4H, m, H_L, $H_{L'}$, H_F, $H_{F'}$), 3.39 (1H, d, J = 14.8, H_j), 3.09-2.97 (2H, m, H_Q, $H_{Q'}$), 2.92 (1H, d, J = 14.7, H_n), 2.74 (1H, d, J = 15.2, H_g), 2.68 (1H, br. d, J = 14.8, H_O), 2.63-2.54 (1H, m, $H_{j'}$), 2.38 (1H, d, J = 14.7, $H_{n'}$), 2.02-1.89 (3H, m, H_P, $H_{P'}$, $H_{O'}$), 1.87-1.77 (2H, m, H_J, $H_{J'}$), 1.75-1.62 (2H, m, H_G, $H_{G'}$), 1.61-1.48 (2H, m, H_K, $H_{K'}$), 1.47-1.36 (2H, m, H_I, $H_{I'}$), 0.94-0.86 (2H, m, H_H, $H_{H'}$), 0.71 (3H, d, J = 6.3, H_s), 0.66 (3H, d, J = 6.3, H_t).

δ_C ($CDCl_3$, 101 MHz) 169.1, 163.1, 159.4, 158.5, 158.1, 157.8, 157.7, 137.9 (d, J = 7.2), 137.0, 136.9, 136.5 (2C, d, J = 48.6), 132.0, 131.5, 131.2, 131.2, 131.1 (2C, d, J = 9.3), 130.6, 130.0, 129.9, 129.4, 128.4 (2C, d, J = 8.4), 128.3. (2C, d, J = 8.4), 127.7 (d, J = 6.4), 126.4, 126.1, 120.6, 120.4, 120.1, 119.0, 115.0, 114.6, 70.8, 69.7, 68.5, 66.9, 44.3, 42.7, 36.5, 34.7, 29.5, 28.9, 28.6, 28.1, 26.8 (d, J = 72.6), 25.5, 24.9, 24.0, 21.1, 21.0.

$\delta_{31P\{1H\}}$ ($CDCl_3$, 202 MHz) 28.1.

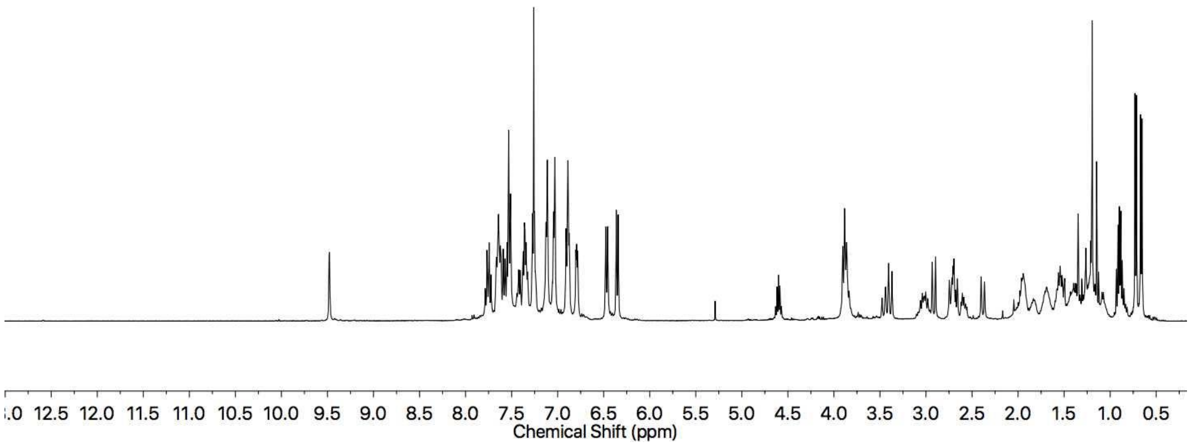

Figure S21: ^{1}H NMR (400 MHz, $CDCl_3$) of (R_{mp})-**5**.

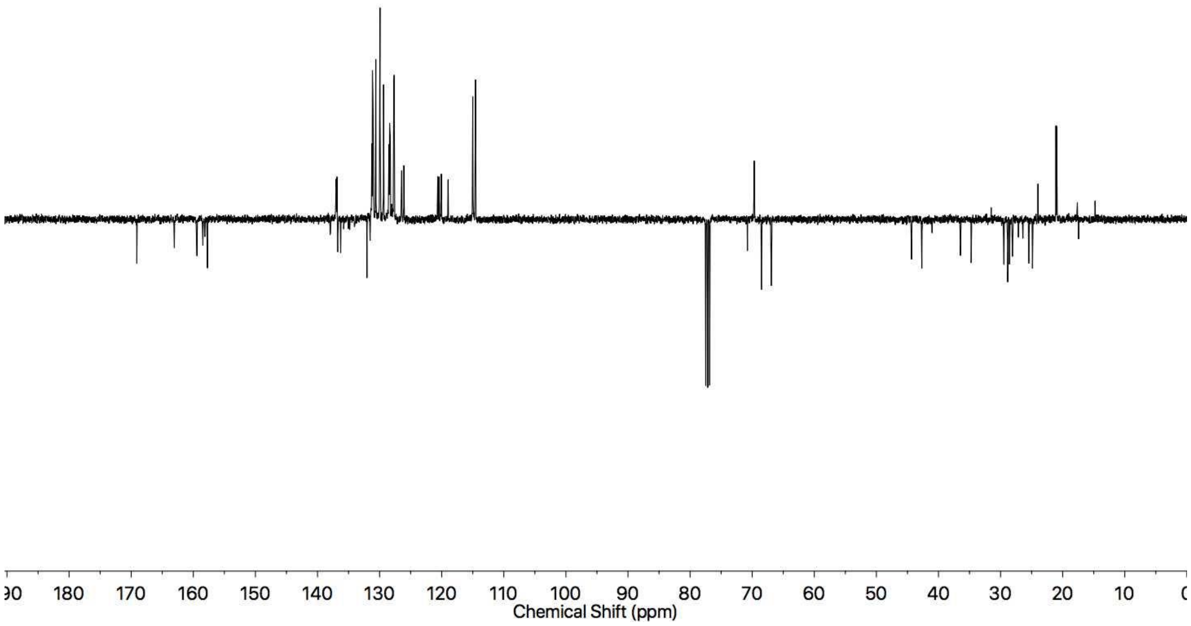

Figure S22: JMOD NMR (101 MHz, $CDCl_3$) of (R_{mp})-**5**.

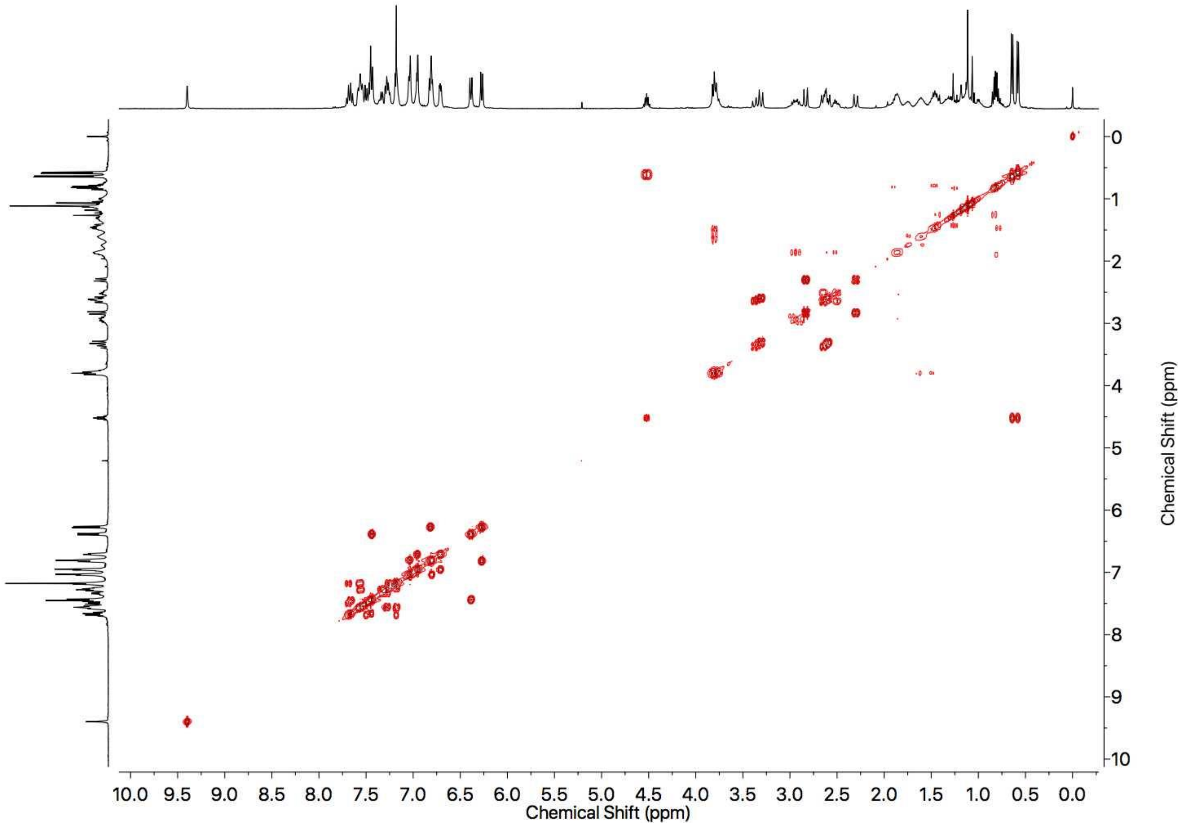

Figure S23: COSY NMR ($CDCl_3$) of (R_{mp})-**5**.

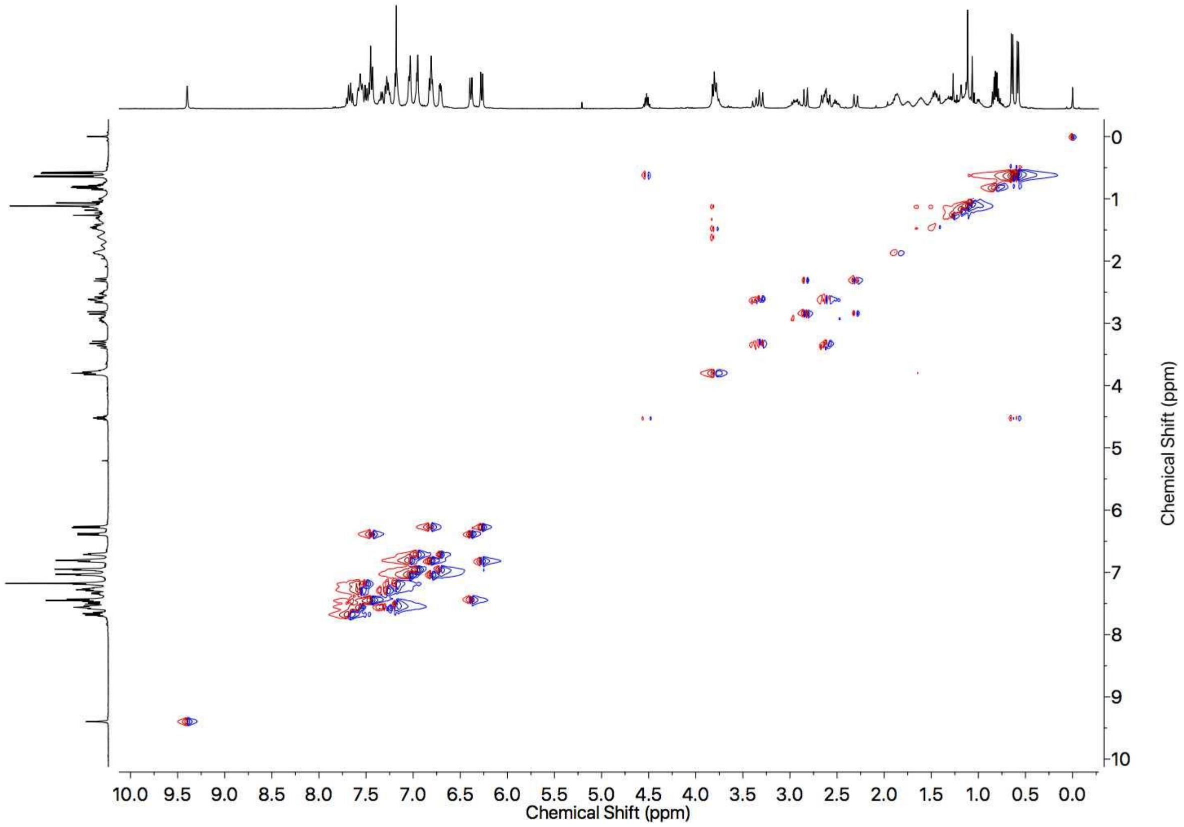

Figure S24: TOCSY NMR ($CDCl_3$) of (R_{mp})-**5**.

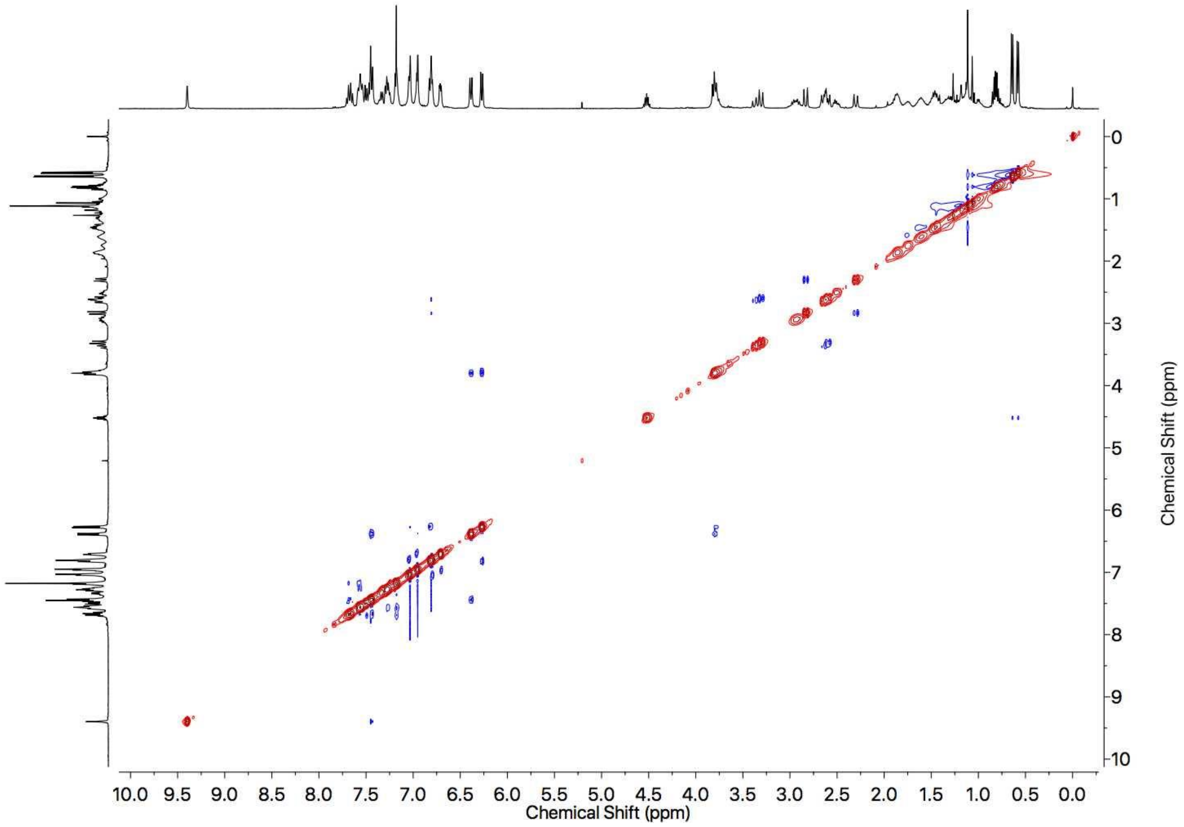

Figure S25: NOESY NMR ($CDCl_3$) of (R_{mp})-**5**.

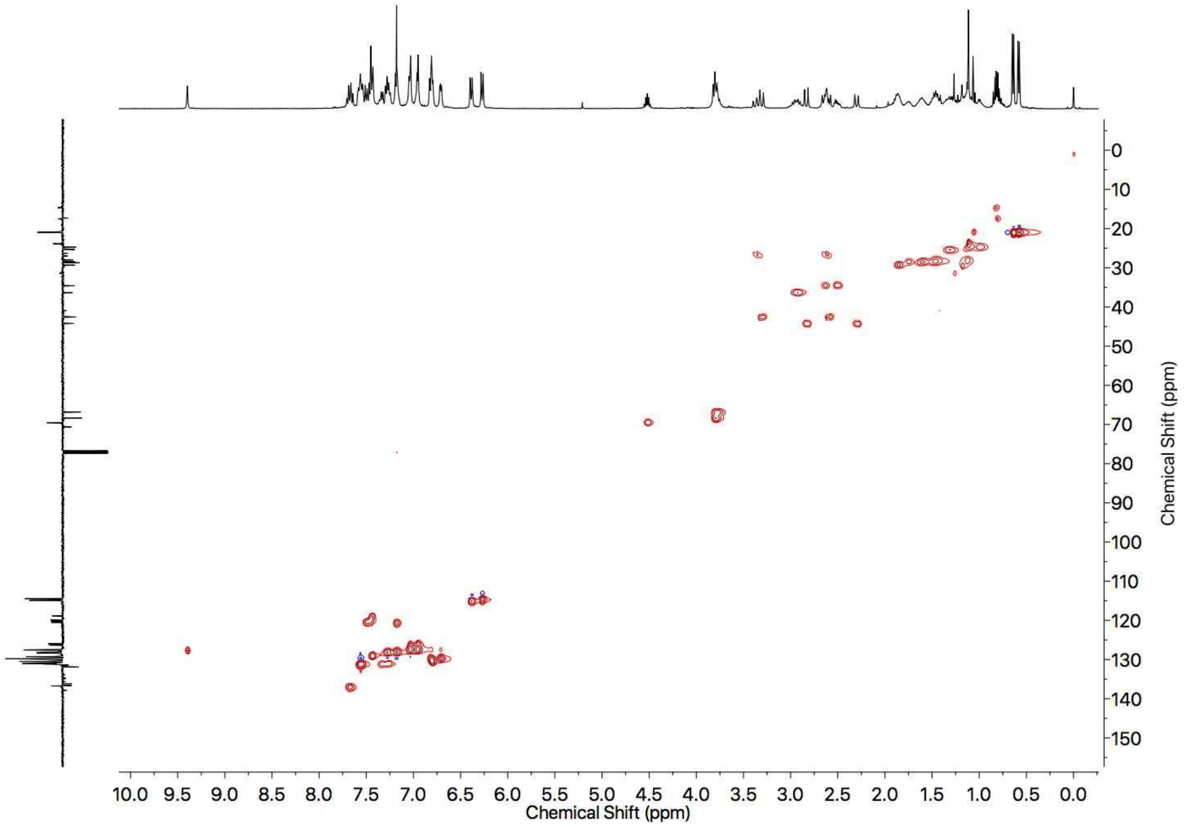

Figure S26: HSQC NMR ($CDCl_3$) of (R_{mp})-**5**.

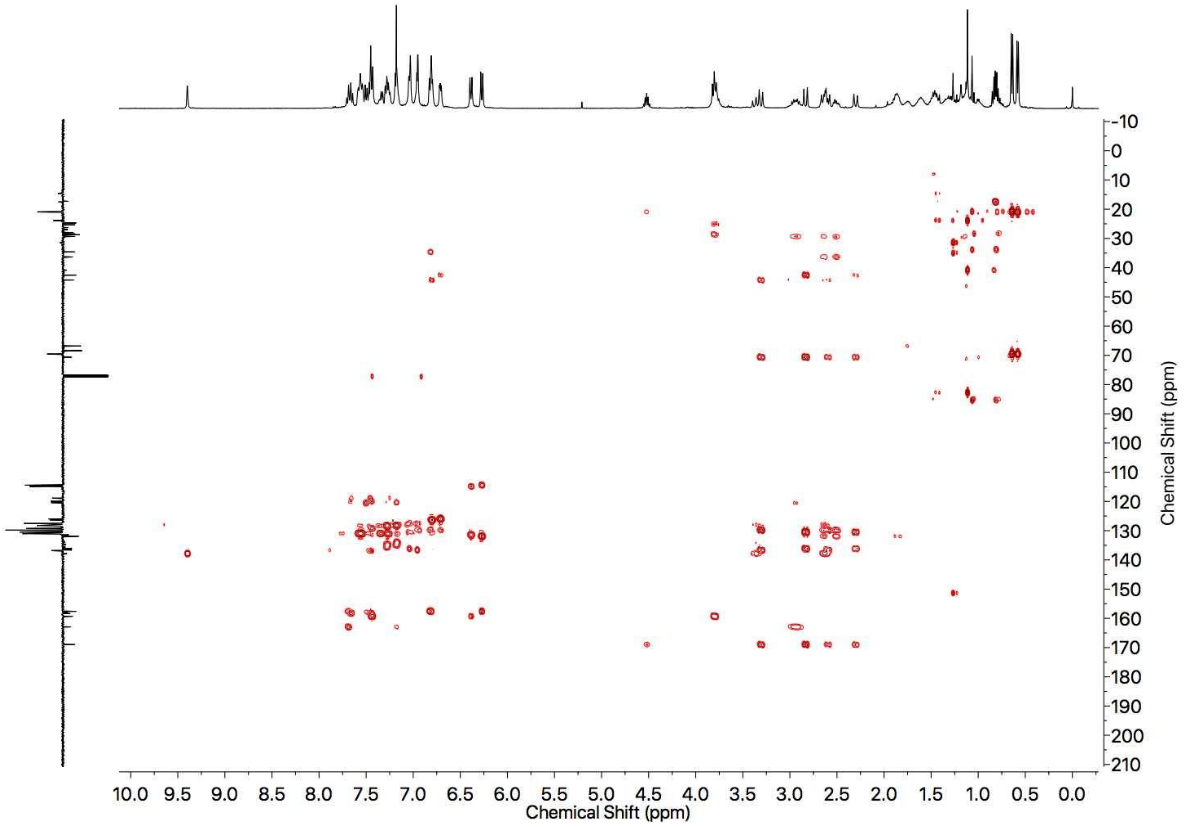

Figure S27: HMBC NMR ($CDCl_3$) of (R_{mp})-**5**.

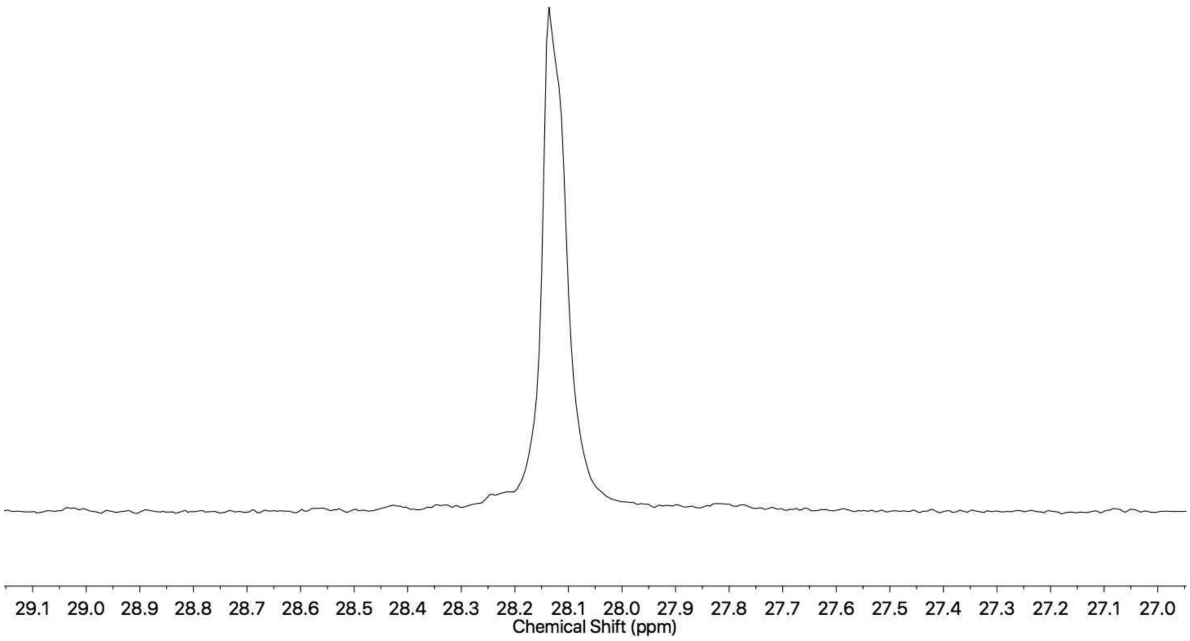

Figure S28: $^{31}P\{^1H\}$ NMR (202 MHz, $CDCl_3$) of (R_{mp})-**5**.

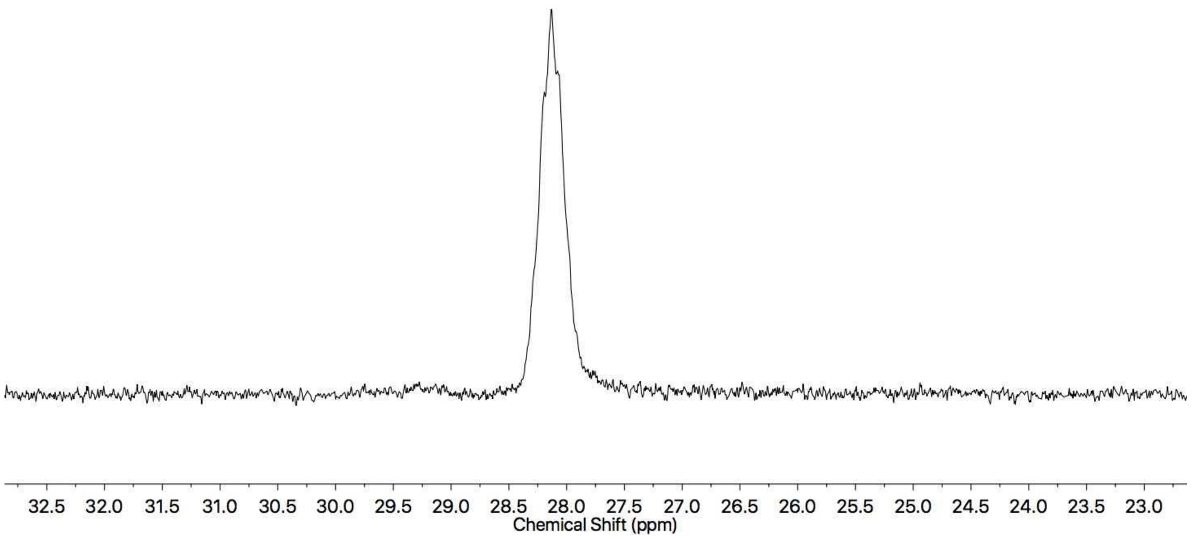

Figure S29: ^{31}P NMR (202 MHz, $CDCl_3$) of (R_{mp})-**5**.

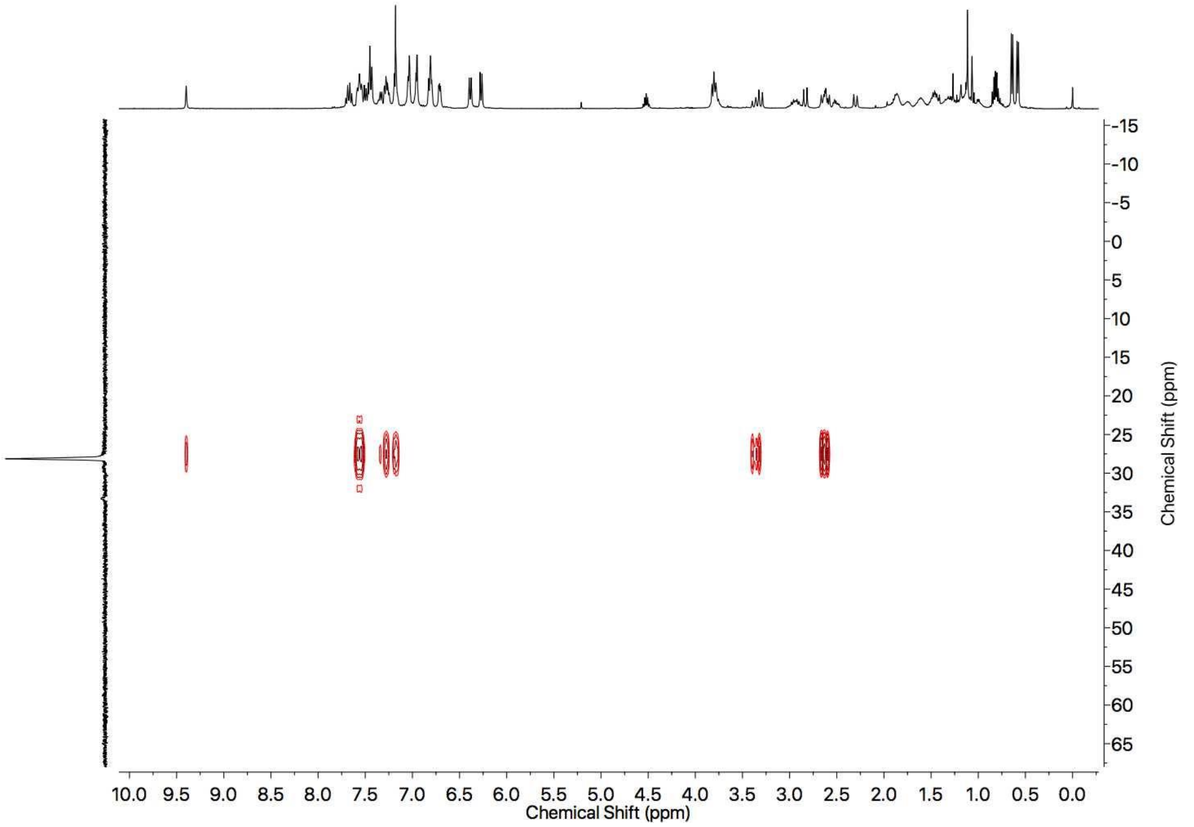

Figure S30: ^{31}P-^{1}H HMBC NMR ($CDCl_3$) of (R_{mp})-**5**.

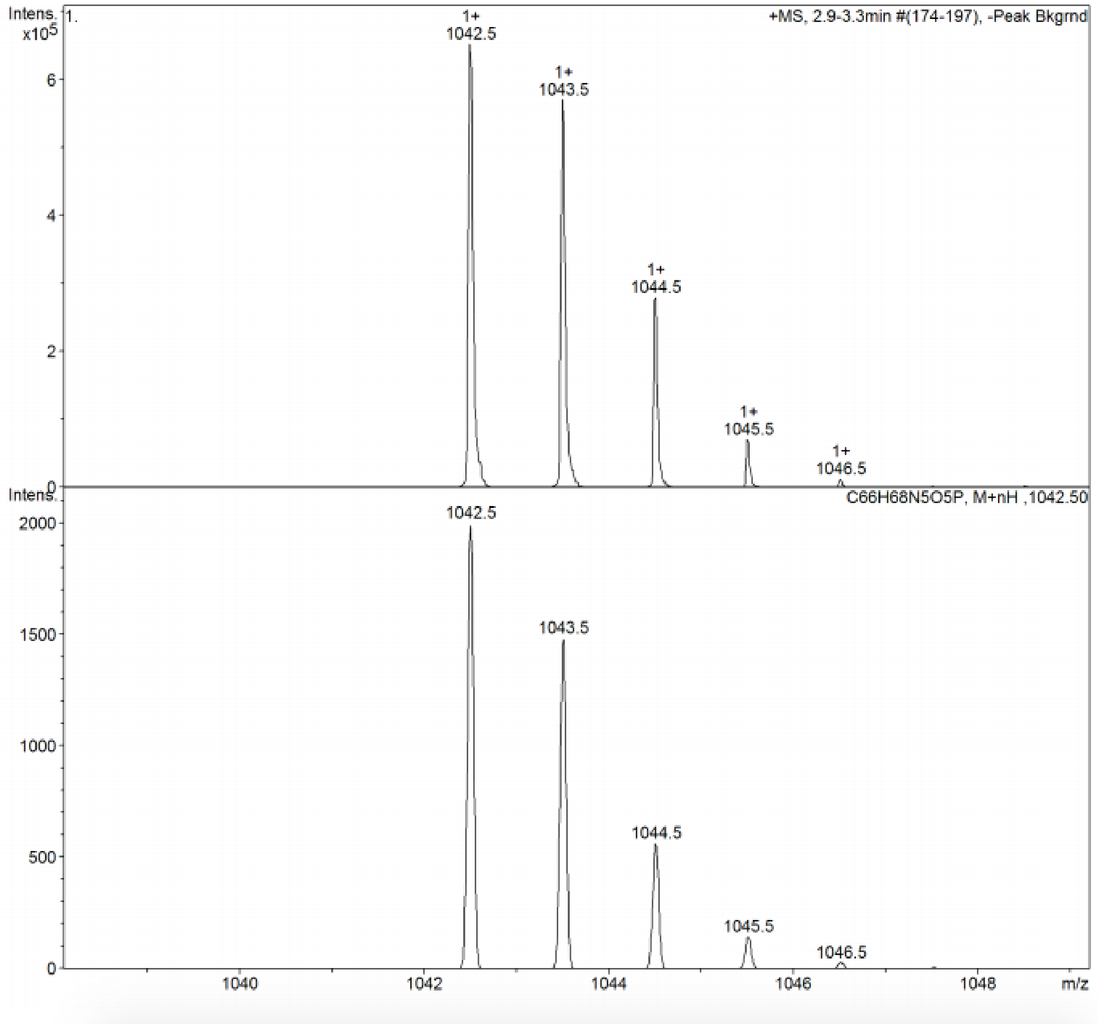

Figure S31: HR-ESI-MS of (R_{mp})-**5**. m/z 1042.5 $[M + H]^+$ (calc. m/z for $C_{66}H_{69}N_5O_5P$ 1042.5).

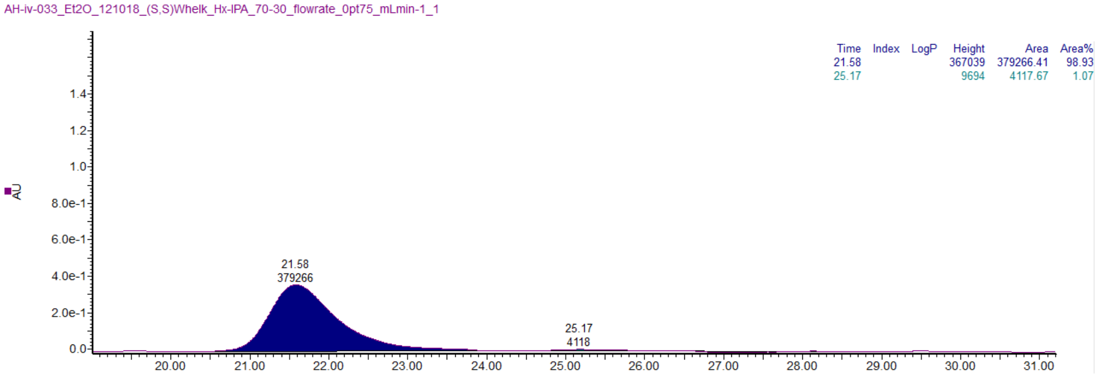

Figure S32: Chiral Stationary Phase HPLC ((*S*,*S*)Whelk, isocratic *n*-hexane-isopropanol 70 : 30, 303 K, load solvent Et_2O, 5 µL injection, flowrate 0.75 $mLmin^{-1}$) of 99 : 1 *er* (R_{mp})-**5**. Retention times (min): (R_{mp})-**5** 21.6, (S_{mp})-**5** 25.2.

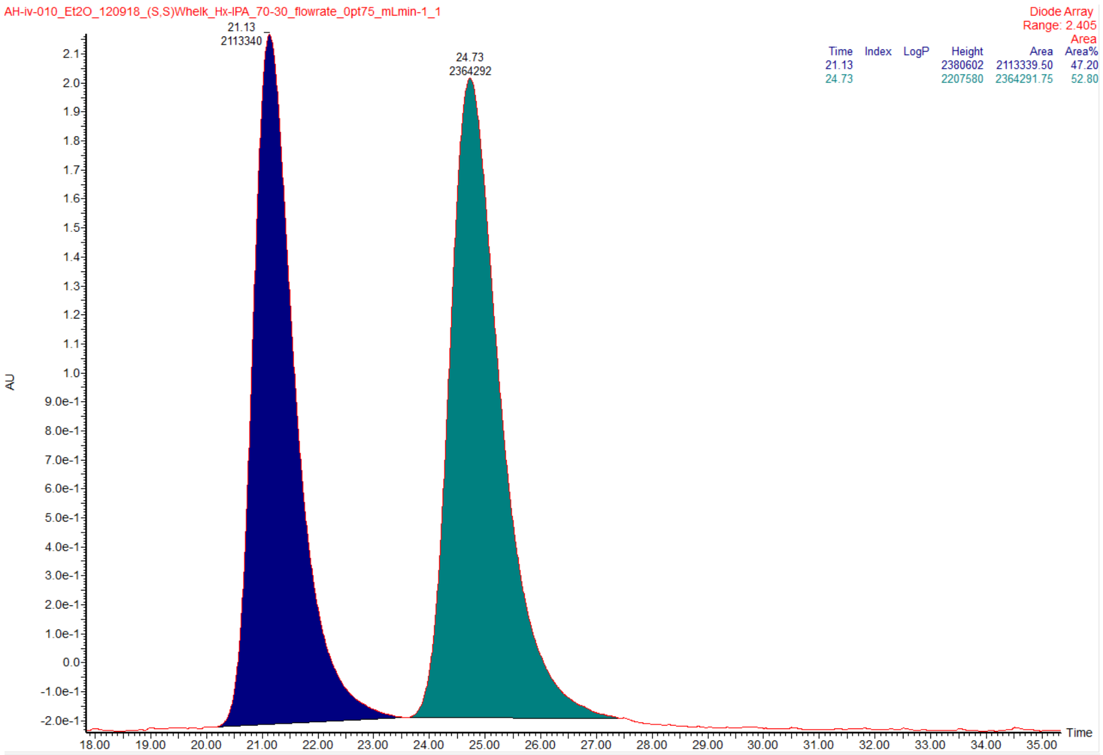

Figure S33: Chiral Stationary Phase HPLC ((*S*,*S*)Whelk, isocratic *n*-hexane-isopropanol 70 : 30, 303 K, load solvent Et_2O, 5 µL injection, flowrate 0.75 $mLmin^{-1}$) of racemate **5**. Retention times (min): (R_{mp})-**5** 21.1, (S_{mp})-**5** 24.7.

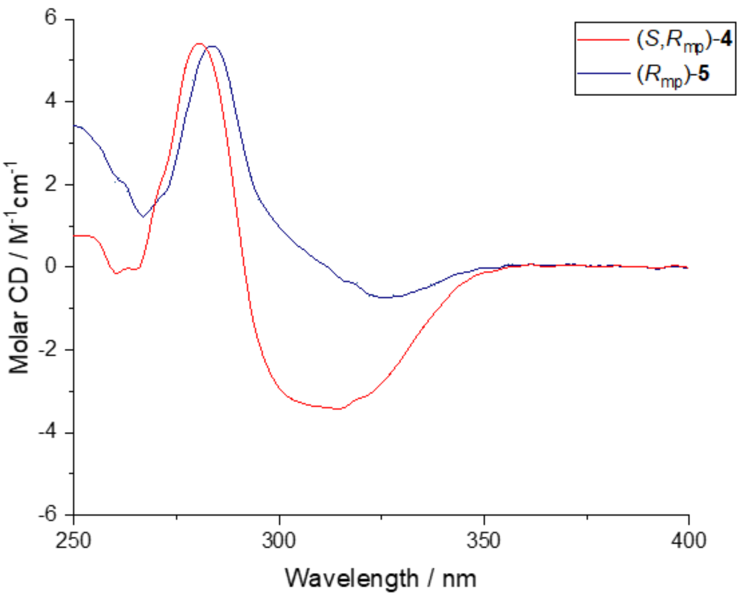

Figure S34: Circular Dichroism spectra of (S,R_{mp})-**4** (53.4 µM, 99 : 1 *er*) and (R_{mp})-**5** (66.2 µM, 99 : 1 *er*) at 293 K in $CHCl_3$.

Rotaxane (S_{mp})-**5**

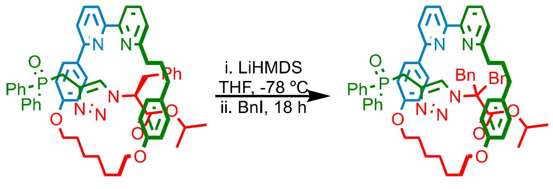

Scheme S3: Synthesis of (S_{mp})-**5**.

(S,S_{mp})-**4** (55.9 mg, 0.059 mmol, 1.0 eq.) was dissolved in anhydrous THF (3 mL) and transferred into a dry CEM MW vial under N_2. The solution was cooled to -78 °C and stirred for 20 min. Lithium bis(trimethylsilyl)amide (1 M in THF, 0.29 mL, 0.29 mmol, 5.0 eq.) was added to the reaction mixture and stirred for 10 min. Benzyl iodide (1 M in THF, 0.59 mL, 0.59 mmol, 10.0 eq.) was added. The reaction was allowed to warm to rt and stirred for 18 h. The reaction mixture was diluted with saturated NH_4Cl (30 mL) and extracted with CH_2Cl_2 (3 x 20 mL). The combined organic extracts were dried ($MgSO_4$) and solvent removed *in vacuo*. The reidue was purified by column chromatography (SiO_2, petrol-EtOAc 0→50%) to give a yellow foam product (S_{mp})-**5** (38.4 mg, 0.037 mmol, 63%, 1 : 99 *er*). Enantiopurity was assessed by chiral stationary phase HPLC. The absolute mechanical stereochemistry was inferred from that of the starting material and the stereolabel assigned based on our established approach[5] using the priority atoms indicated below.

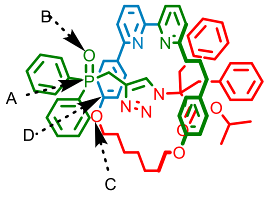

(S_{mp})-**5**

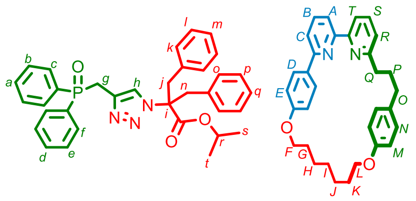

(S_{mp})-**5**

(S_{mp})-**5** same as (R_{mp})-**5** above

δ_H ($CDCl_3$, 400 MHz) 9.48 (1H, d, J = 1.6, H_h), 7.77 (1H, t, J = 8.1, H_S), 7.74 (1H, t, J = 8.1, H_B), 7.64 (4H, ddd, J = 8.2, 7.7, 2.3, H_c, H_f), 7.58 (1H, d, J = 8.1, H_T), 7.54 (1H, d, J = 7.8, H_A), 7.52 (2H, d, J = 8.5, H_D), 7.36 (4H, td, J = 7.6, 2.7, H_b, H_e), 7.29-7.23 (4H, m, H_a, H_d, H_R, H_C), 7.14-7.09 (3H, m, H_l, - H_m), 7.06-7.01 (3H, m, H_p, H_q), 6.90 (2H, d, J = 8.5, H_N), 6.89 (2H, dd, J = 7.2, 2.1, H_k), 6.79 (2H, dd, J = 7.1, 2.6, H_o), 6.49 (2H, d, J = 8.5, H_E), 6.35 (2H, d, J = 8.3, H_M), 4.60 (1H, sept., J = 6.2, H_r), 3.45 (1H, d, J = 15.0, H_g), 3.93-3.81 (4H, m, H_L, $H_{L'}$, H_F, $H_{F'}$), 3.39 (1H, d, J = 14.8, H_j), 3.09-2.97 (2H, m, H_Q, $H_{Q'}$), 2.92 (1H, d, J = 14.7, H_n), 2.74 (1H, d, J = 15.2, H_g), 2.68 (1H, br. d, J = 14.8, H_O), 2.63-2.54 (1H, m, $H_{j'}$), 2.38 (1H, d, J = 14.7, $H_{n'}$), 2.02-1.89 (3H, m, H_P, $H_{P'}$, $H_{O'}$), 1.87-1.77 (2H, m, H_J, $H_{J'}$), 1.75-1.62 (2H, m, H_G, $H_{G'}$), 1.61-1.48 (2H, m, H_K, $H_{K'}$), 1.47-1.36 (2H, m, H_I, $H_{I'}$), 0.94-0.86 (2H, m, H_H, $H_{H'}$), 0.71 (3H, d, J = 6.3, H_s), 0.66 (3H, d, J = 6.3, H_t).

δ_C ($CDCl_3$, 101 MHz) 169.1, 163.1, 159.4, 158.5, 158.1, 157.8, 157.7, 137.9 (d, J = 7.2), 137.0, 136.9, 136.5 (2C, d, J = 48.6), 132.0, 131.5, 131.2, 131.2, 131.1 (2C, d, J = 9.3), 130.6, 130.0, 129.9, 129.4, 128.4 (2C, d, J = 8.4), 128.3. (2C, d, J = 8.4), 127.7 (d, J = 6.4), 126.4, 126.1, 120.6, 120.4, 120.1, 119.0, 115.0, 114.6, 70.8, 69.7, 68.5, 66.9, 44.3, 42.7, 36.5, 34.7, 29.5, 28.9, 28.6, 28.1, 26.8 (d, J = 72.6), 25.5, 24.9, 24.0, 21.1, 21.0.

$\delta_{31P\{1H\}}$ ($CDCl_3$, 202 MHz) 28.1.

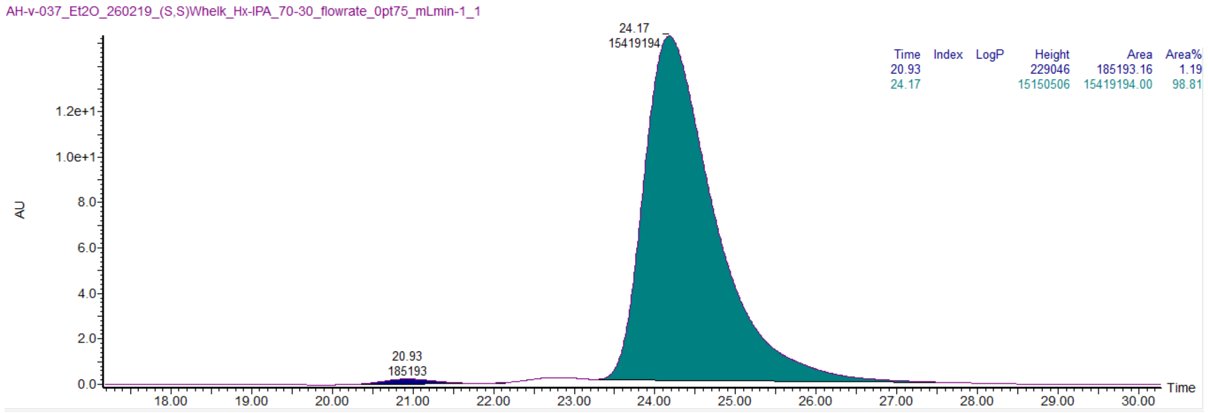

Figure S35: Chiral Stationary Phase HPLC ((*S*,*S*)Whelk, isocratic *n*-hexane-isopropanol 70 : 30, 303 K, load solvent Et_2O, 5 µL injection, flowrate 0.75 mLmin^{-1}) of 1 : 99 *er* (S_{mp})-**5**. Retention times (min): (R_{mp})-**5** 20.9, (S_{mp})-**5** 24.2.

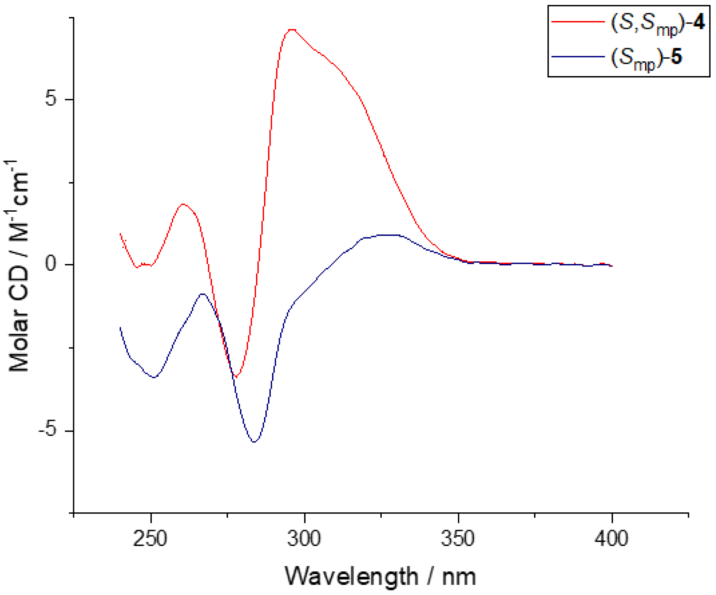

Figure S36: Circular Dichroism spectra of (*S*,S_{mp})-**4** (58.8 µM, (*S*,S_{mp})-**4** : (*R*,S_{mp})-**4** : (*S*,R_{mp})-**4**, 98.4 : 1.0 : 0.6) and (S_{mp})-**5** (54.7 µM, 99 : 1 *er*) at 293 K in $CHCl_3$.

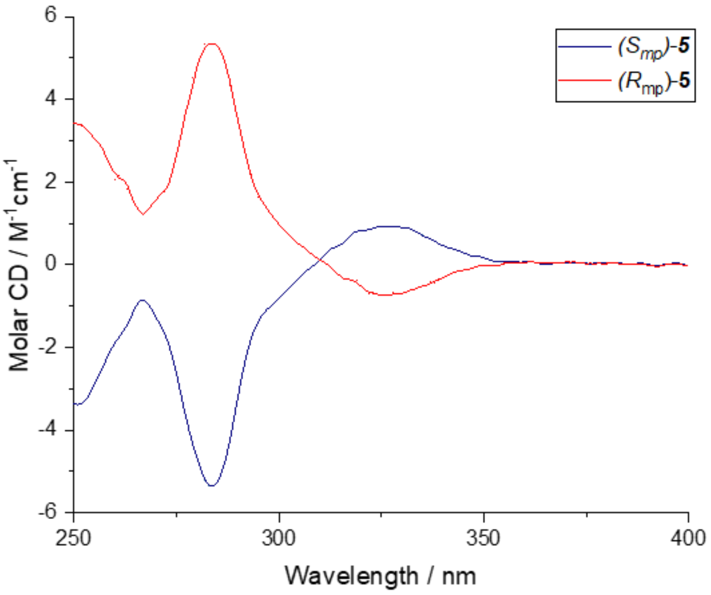

Figure S37: Circular Dichroism spectra of (R_{mp})-**5** (66.2 µM, 99 : 1 *er*) and (S_{mp})-**5** (54.7 µM, 99 : 1 *er*) at 293 K in $CHCl_3$.

Rotaxane [Au((R_{mp})-**6**)(Cl)]

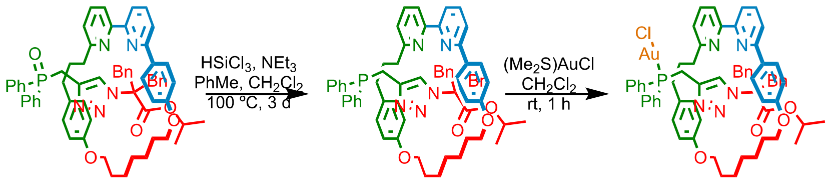

Scheme S4: Synthesis of [Au((R_{mp})-**6**)(Cl)].

A Young's tube was dried under reduced pressure with a heat gun three times and filled with nitrogen in-between each period, as per Schlenk technique. Under a high flow of nitrogen, anhydrous NEt_3 (3.0 mL, 21.5 mmol, 200 eq.) was added, followed by $HSiCl_3$ (1.1 mL, 10.7 mmol, 100 eq.) slowly. After 5 minutes, (R_{mp})-**5** (112 mg, 0.107 mmol, 1eq.) was transferred in anhydrous PhMe (5.5 mL) and then anhydrous CH_2Cl_2 (1.1 mL). The vessel was sealed and stirred at 100 ^{o}C for 3 days. After 3 days the solution was cooled to rt, washed with NaOH (1 M, 40 mL) and extracted in CH_2Cl_2 (4 x 30 mL). The combined organic phases were dried ($MgSO_4$), and the solvent removed *in vacuo*. This work up was repeated 4 times until NEt_3.HCl was absent from the ^{1}H NMR, yielding an orange oil (R_{mp})**-6** (76.2 mg, 0.074 mmol, 69%).

A dry vial was charged with (Me_2S)AuCl (22.1 mg, 0.074 mmol, 1.0 eq.) and (R_{mp})-**6** was transferred in anhydrous CH_2Cl_2 (1.5 mL). The solution was stirred at rt for 1 h. The solution was filtered through Celite® and eluted with Et_2O. The solvent was removed *in vacuo*. The residue was purified by column chromatography (SiO_2, petrol-Et_2O 0→100%) to give [Au((R_{mp})-**6**)(Cl)] as a white foam (86.2 mg, 0.068 mmol, 93%), the enantiopurity of which (*er* = 99 : 1) was inferred from the enantiopurity of the (R_{mp})-**5** starting material and the stereolabel assigned based on our established approach[5] using the priority atoms indicated below.

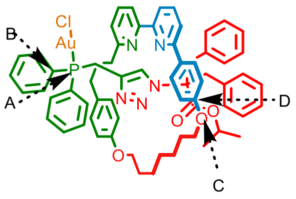

[Au((R_{mp})-**6**)Cl]

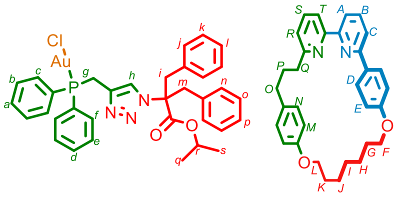

[Au((R_{mp})-**6**)Cl]

δ_H ($CDCl_3$, 400 MHz) 9.82 (1H, d, *J* = 2.3, ***H***$_h$), 7.86 (1H, t, *J* = 7.8, ***H***$_S$), 7.77 (1H, t, *J* = 7.7, ***H***$_B$), 7.74 (2H, dd, *J* = 11.9, 5.0, ***H***$_f$), 7.69 (1H, d, *J* = 7.7, ***H***$_T$), 7.57 (1H, dd, *J* = 7.5, 0.7, ***H***$_A$), 7.52 (1H, dd, *J* = 7.9, 0.7, ***H***$_C$), 7.49 (1H, ddt (dq), *J* = 7.2, 1.3, ***H***$_d$), 7.44 (2H, td, *J* = 7.9, 2.5, ***H***$_e$), 7.37 (2H, ddd, *J* = 12.7, 7.9, 1.5, ***H***$_c$) 7.33 (2H, d, *J* = 8.9, ***H***$_D$), 7.31 (1H, d, *J* = 7.7, ***H***$_R$), 7.29-7.27 (1H, m, ***H***$_a$), 7.19 (2H, td, *J* = 7.9, 2.6, ***H***$_b$), 7.16-7.10 (3H, m, ***H***$_o$, ***H***$_p$), 7.07-7.03 (3H, m, ***H***$_j$, ***H***$_l$), 6.94 (2H, dd, *J* = 7.5, 1.7, ***H***$_M$), 6.76-6.71 (2H, m, ***H***$_k$), 6.61 (2H, d, *J* = 8.4, ***H***$_N$), 6.31 (2H, d, *J* = 8.4, ***H***$_n$), 6.15 (2H, d, *J* = 8.9, ***H***$_E$), 4.60 (1H, sept., *J* = 6.3, ***H***$_r$), 3.17 (1H, d, *J* = 14.8, ***H***$_i$), 3.04 (1H, d, *J* = 14.8, ***H***$_m$), 3.01 (1H, dd, *J* = 14.8, 13.3, ***H***$_g$), 2.94-2.85 (1H), 2.79 (1H, dd, *J* = 14.8, 12.0, ***H***$_{g'}$), 2.75-2.65 (1H), 2.59 (1H, d, *J* = 14.8, ***H***$_{i'}$), 2.54-2.44 (1H), 2.28 (1H, d, *J* = 14.8, ***H***$_{m'}$), 2.18-1.88 (3H), 1.78-1.03 (14H), 0.68 (3H, d, *J* = 6.4, ***H***$_q$), 0.63 (3H, d, *J* = 6.4, ***H***$_s$). It was not possible to unambiguously assign alkyl region of macrocycle (***H***$_F$-***H***$_L$, ***H***$_O$-***H***$_Q$).

δ_C ($CDCl_3$, 101 MHz) 169.0, 162.3, 158.7, 158.5, 158.4, 157.9, 157.4, 137.9, 137.8, 137.1, 137.0, 136.6, 136.1, 134.4, 134.2, 133.3, 133.2, 132.0, 131.5, 131.5, 131.2, 131.1, 131.0, 130.9, 130.2, 129.9, 129.8, 129.6, 128.9, 128.8, 128.7, 128.6, 128.6, 127.7, 127.6, 126.2, 126.2, 121.8, 120.7, 120.4, 119.0, 114.5, 114.2, 70.6, 69.6, 68.0, 57.2, 65.9, 44.0, 42.8, 35.7, 34.2, 30.3, 29.5, 28.4, 28.2, 27.9, 25.4, 25.3, 25.0, 24.8, 20.8, 20.8, 15.3.

64 signals corresponding to all 53 environments (11 doublets coupling to ^{31}P)

$\delta_{31P\{1H\}}$ ($CDCl_3$, 202 MHz) 26.2.

δ_{31P} ($CDCl_3$, 202 MHz) 26.2 (1P, broad quin.d, *J* 12.6, 3.6).

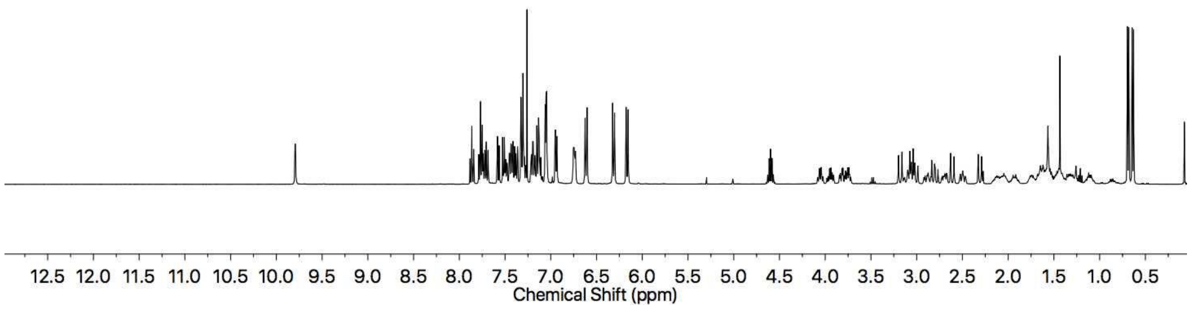

Figure S38: ^{1}H NMR (400 MHz, $CDCl_3$) of [Au((*R*$_{mp}$)-**6**)(Cl)].

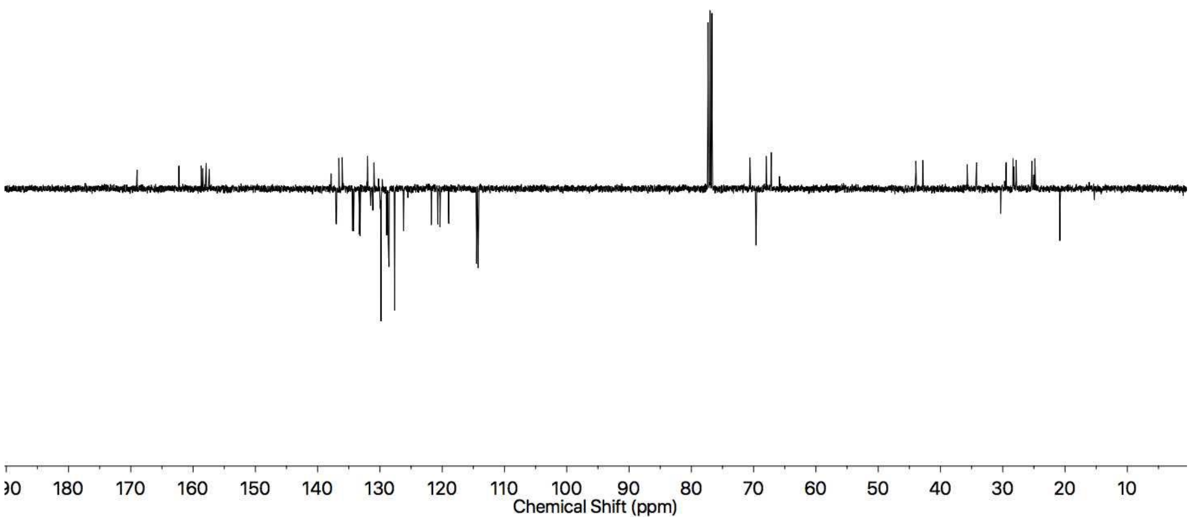

Figure S39: JMOD NMR (101 MHz, $CDCl_3$) of [Au((*R*$_{mp}$)-**6**)(Cl)].

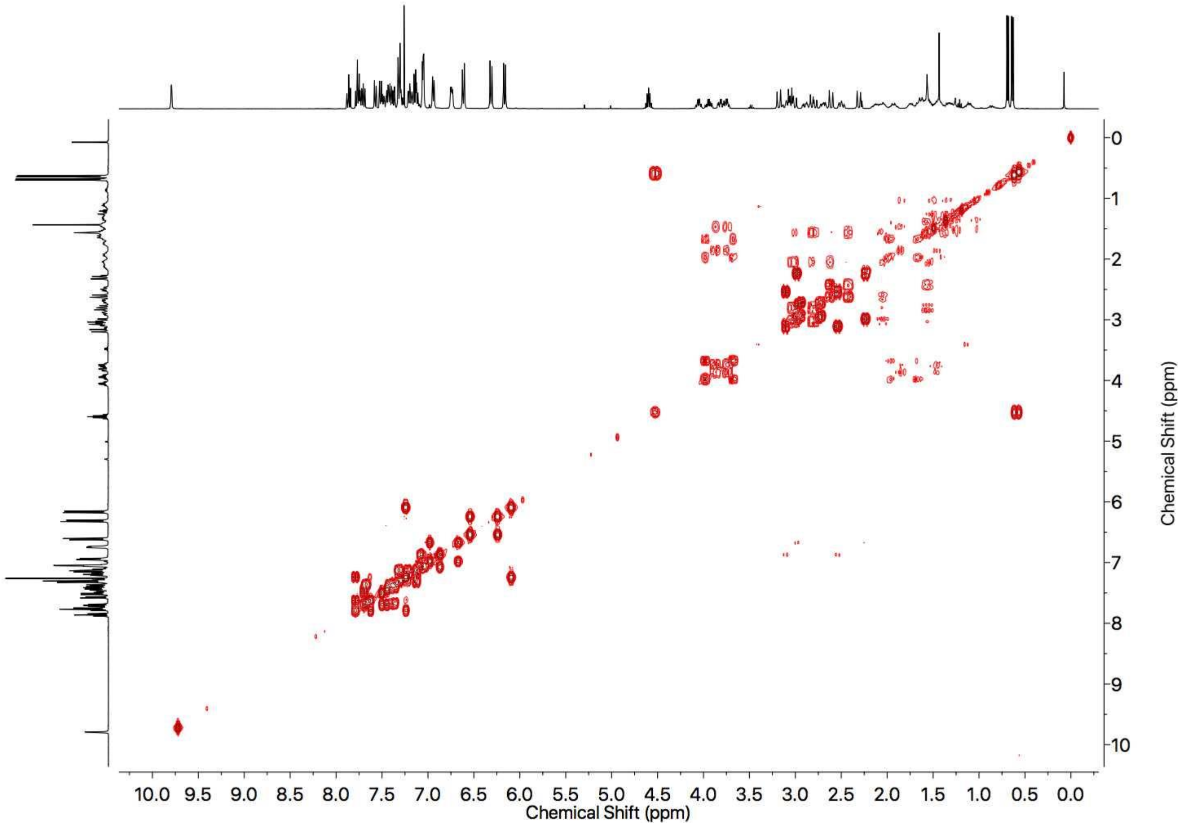

Figure S40: COSY NMR ($CDCl_3$) of [Au((R_{mp})-**6**)(Cl)].

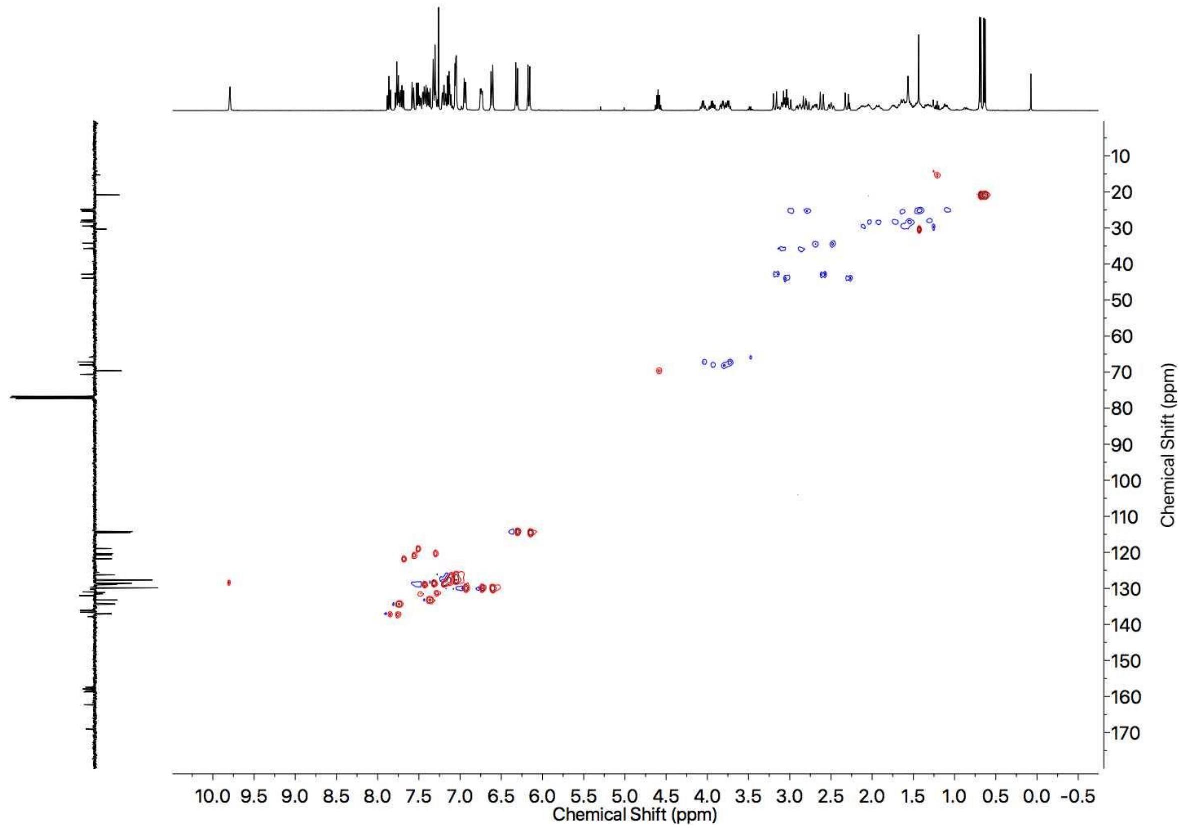

Figure S41: HSQC NMR ($CDCl_3$) of [Au((R_{mp})-**6**)(Cl)].

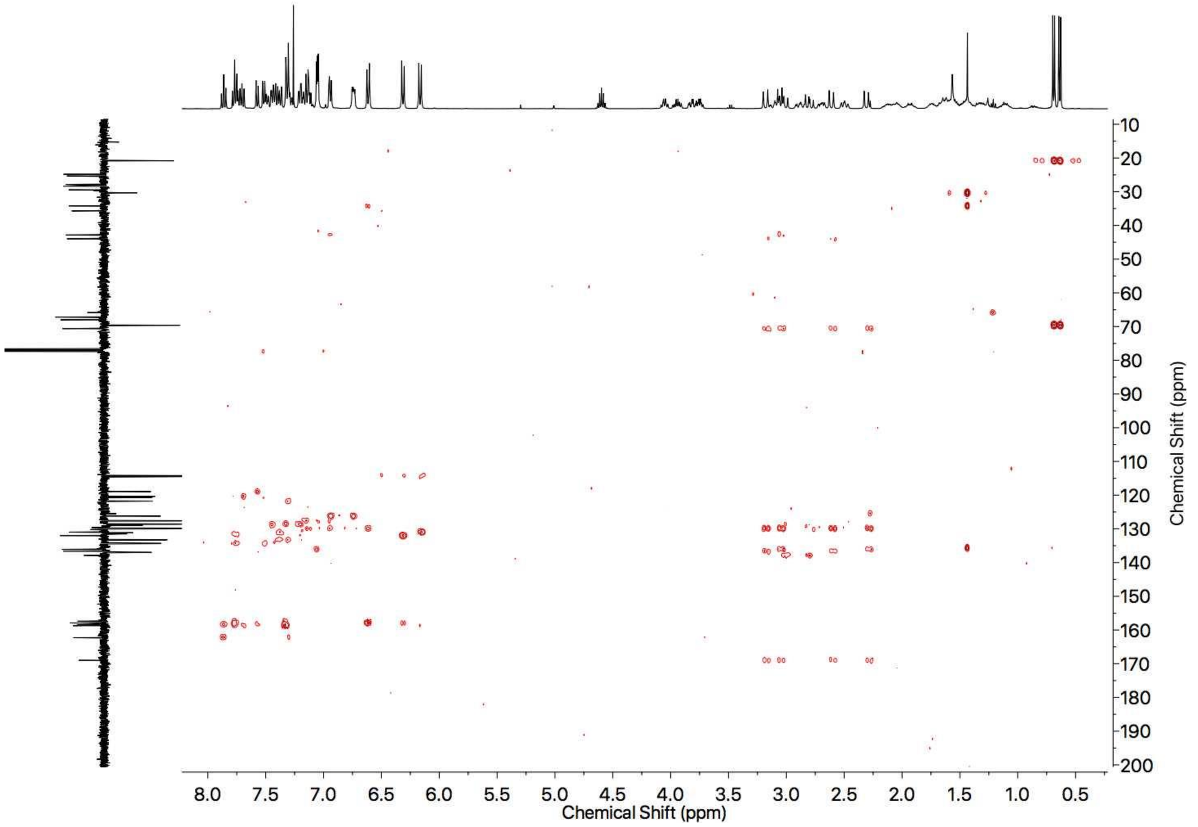

Figure S42: HMBC NMR ($CDCl_3$) of [Au((R_{mp})-**6**)(Cl)].

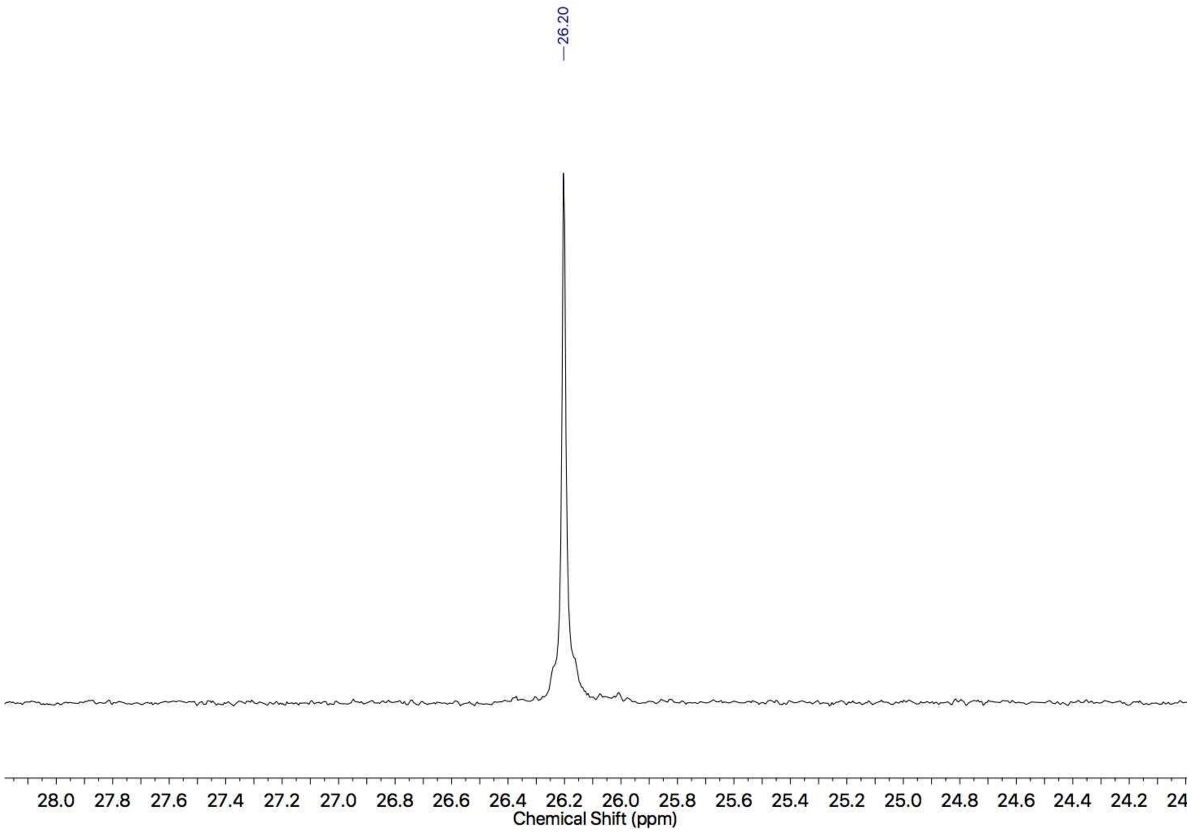

Figure S43: $^{31}P\{^1H\}$ NMR (202 MHz, $CDCl_3$) of [Au((R_{mp})-**6**)(Cl)].

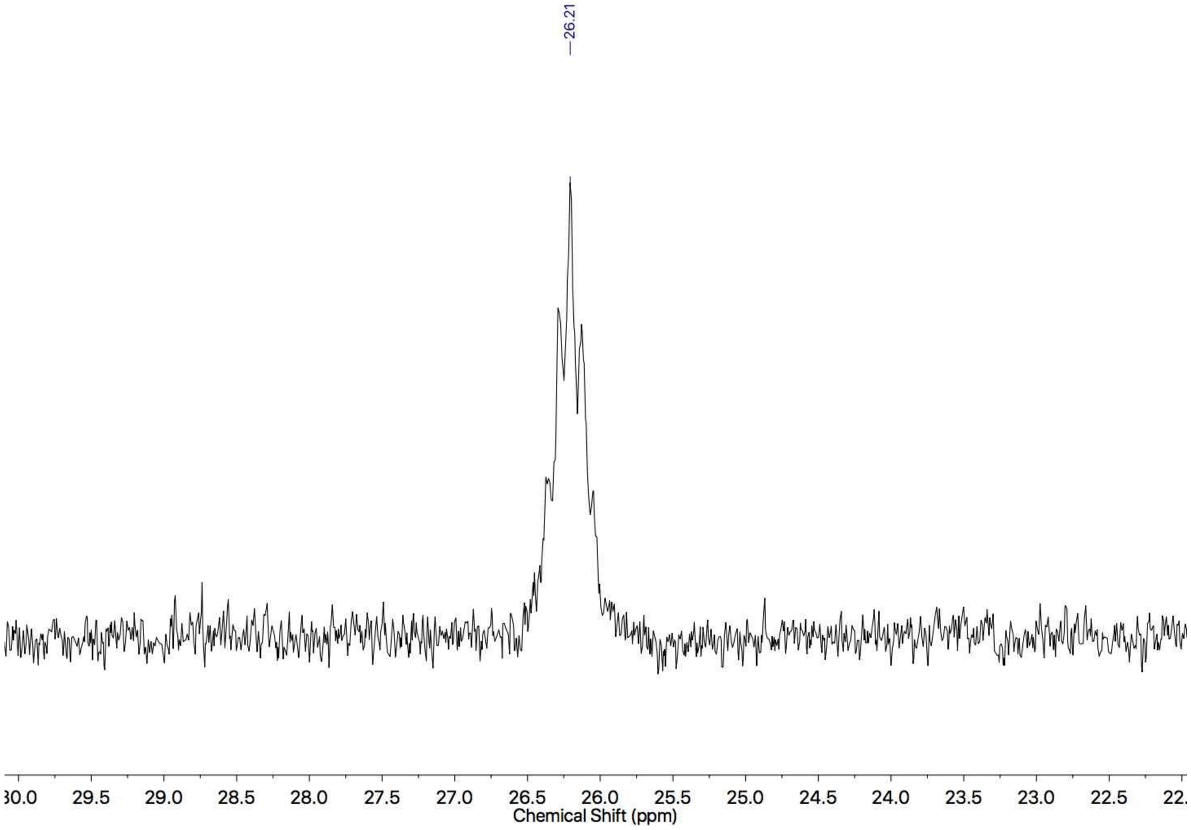

Figure S44: ^{31}P NMR (202 MHz, $CDCl_3$) of [Au((R_{mp})-**6**)(Cl)].

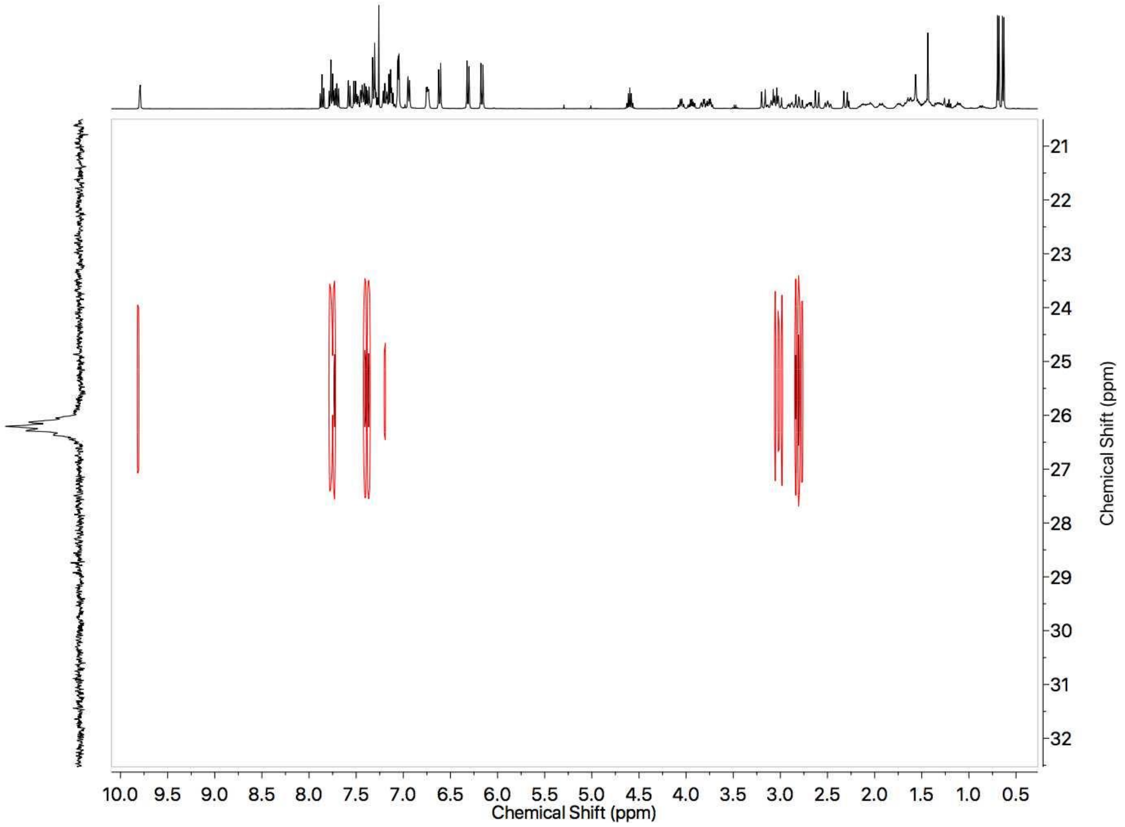

Figure S45: ^{1}H-^{31}P HMBC NMR ($CDCl_3$) of [Au((R_{mp})-**6**)(Cl)].

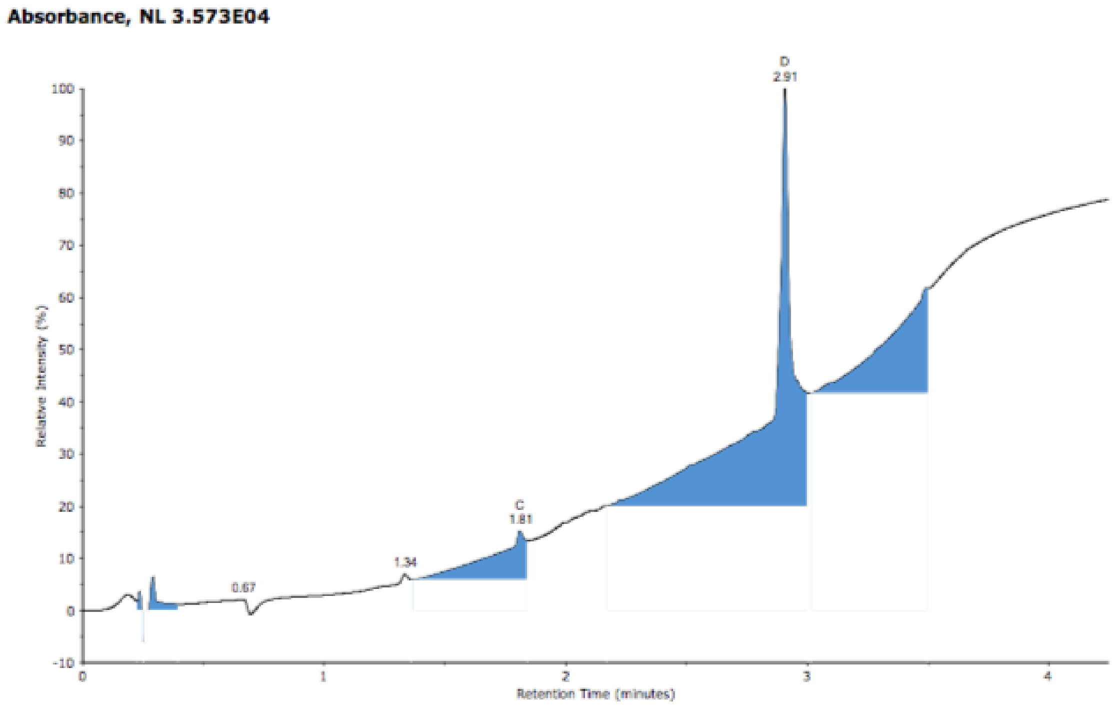

Figure S46: LC-ESI-MS chromatography of [Au((R_{mp})-**6**)(Cl)].

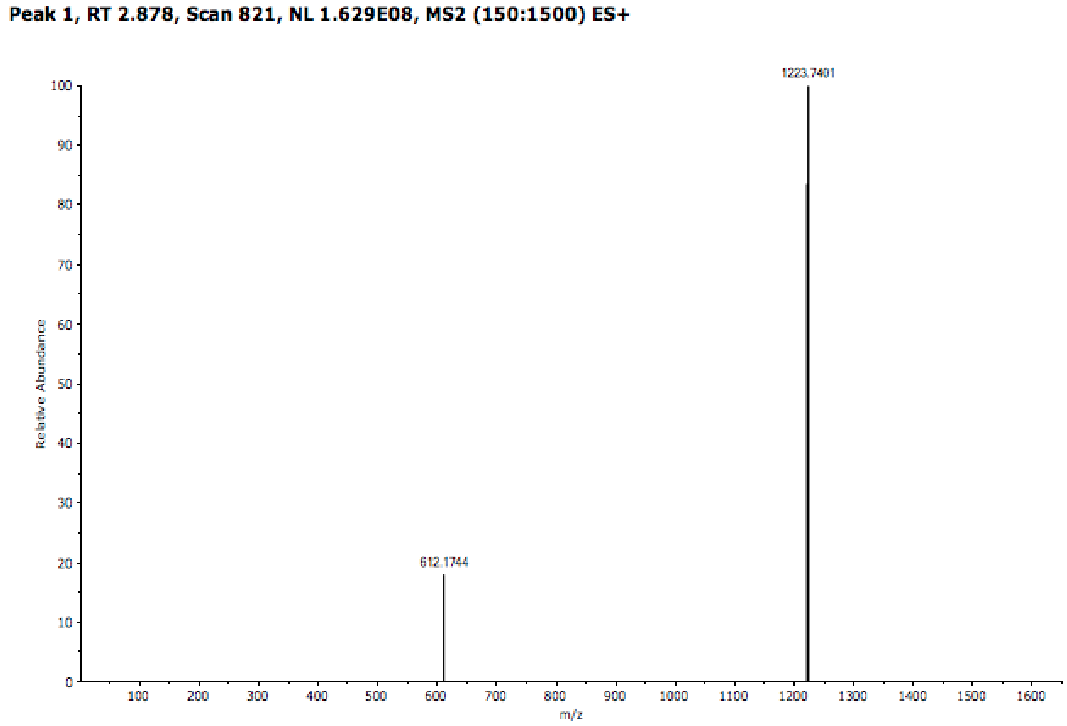

Figure S47: Low resolution LC-ESI-MS spectrum of [Au((R_{mp})-**6**)(Cl)].

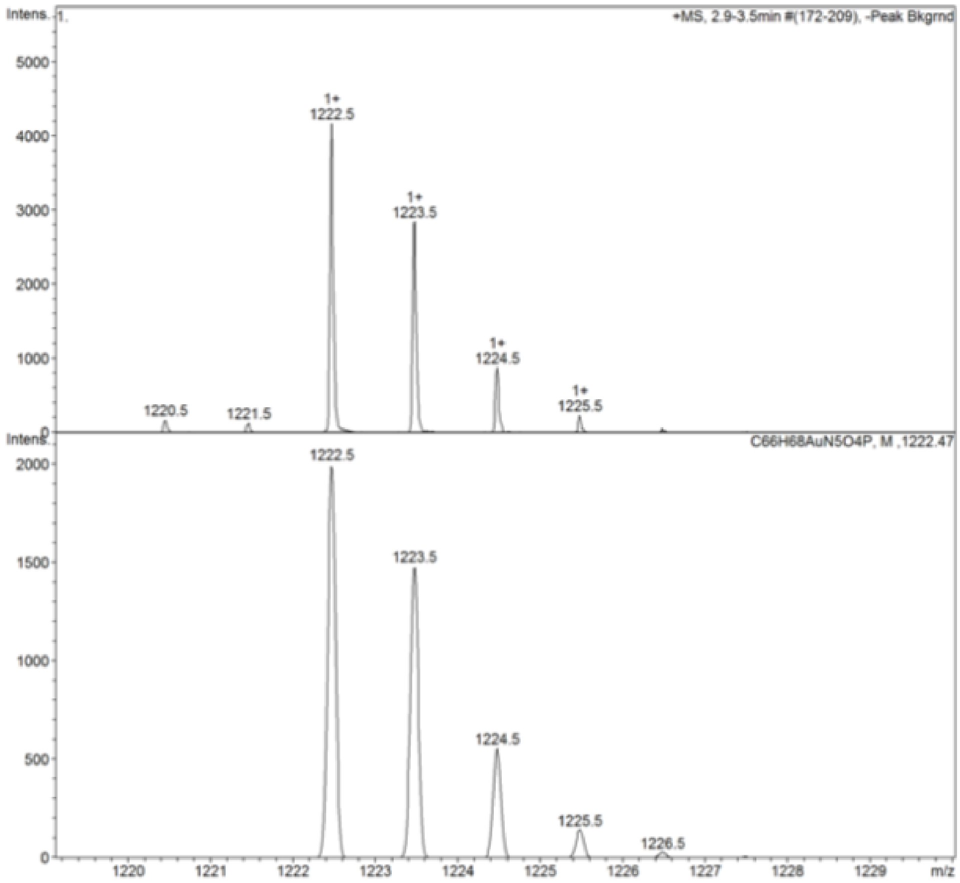

Figure S48: High resolution ESI-MS spectrum of [Au((*R*$_{mp}$)-**6**)(Cl)]. [M - Cl]$^+$ 1222.5 (calc. m/z for $C_{66}H_{68}N_5O_4PAu$ 1222.47), [M + H - Cl]$^{2+}$ 611.7 (calc. m/z for $C_{66}H_{68}N_5O_4PAu$ 611.74).

Rotaxane [Au((S_{mp})-**6**)(Cl)]

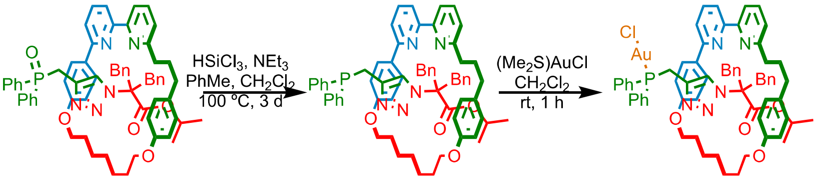

Scheme S5: Synthesis of [Au((S_{mp})-**6**)(Cl)].

A Young's tube was dried under reduced pressure with a heat gun three times and filled with nitrogen in-between each period, as per Schlenk technique. Under a high flow of nitrogen, anhydrous NEt_3 (2.5 mL, 17.9 mmol, 200 eq.) was added, followed by $HSiCl_3$ (0.9 mL, 8.95 mmol, 100 eq.) slowly. After 5 minutes, (S_{mp})-**5** (93.3 mg, 0.090 mmol, 1eq.) was transferred in anhydrous PhMe (4.6 mL) and then anhydrous CH_2Cl_2 (0.9 mL). The vessel was sealed and stirred at 100 °C for 3 days. After 3 days the solution was cooled to rt, washed with NaOH (1 M, 40 mL) and extracted in CH_2Cl_2 (4 x 30 mL). The combined organic phases were dried ($MgSO_4$), and the solvent removed *in vacuo*. This work up was repeated 4 times until NEt_3.HCl was absent from the ^{1}H NMR, yielding an orange oil (S_{mp})-**6** (85.3 mg, 0.083 mmol, 92%).

A dry vial was charged with (Me_2S)AuCl (24.4 mg, 0.083 mmol, 1.0 eq.) and (S_{mp})-**6** was transferred in anhydrous CH_2Cl_2 (2.0 mL). The solution was stirred at rt for 1 h. The solution was filtered through Celite® and eluted with Et_2O. The solvent was removed *in vacuo*. The residue was purified by column chromatography (SiO_2, petrol-Et_2O 0→100%) to give [Au((S_{mp})-**6**)(Cl)] as a white foam (70.5 mg, 0.056 mmol, 67%, 62% yield over two steps), the enantiopurity of which (*er* = 99 : 1) was inferred from the enantiopurity of the (S_{mp})-**5** starting material and the stereolabel assigned based on our established approach[5] using the priority atoms indicated below.

[Au((S_{mp})-**6**)(Cl)] same as [Au((R_{mp})-**6**)(Cl)] above

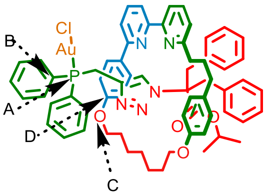
[Au((S_{mp})-**6**)Cl]

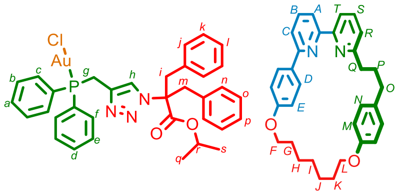
[Au((S_{mp})-**6**)Cl]

δ_H ($CDCl_3$, 400 MHz) 9.82 (1H, d, J = 2.3, H_h), 7.86 (1H, t, J = 7.8, H_S), 7.77 (1H, t, J = 7.7, H_B), 7.74 (2H, dd, J = 11.9, 5.0, H_f), 7.69 (1H, d, J = 7.7, H_T), 7.57 (1H, dd, J = 7.5, 0.7, H_A), 7.52 (1H, dd, J = 7.9, 0.7, H_C), 7.49 (1H, ddt (dq), J = 7.2, 1.3, H_d), 7.44 (2H, td, J = 7.9, 2.5, H_e), 7.37 (2H, ddd, J = 12.7, 7.9, 1.5, H_c) 7.33 (2H, d, J = 8.9, H_D), 7.31 (1H, d, J = 7.7, H_R), 7.29-7.27 (1H, m, H_a), 7.19 (2H, td, J = 7.9, 2.6, H_b), 7.16-7.10 (3H, m, H_o, H_p), 7.07-7.03 (3H, m, H_j, H_l), 6.94 (2H, dd, J = 7.5, 1.7, H_M), 6.76-6.71 (2H, m, H_k), 6.61 (2H, d, J = 8.4, H_N), 6.31 (2H, d, J = 8.4, H_n), 6.15 (2H, d, J = 8.9, H_E), 4.60 (1H, sept., J = 6.3, H_r), 3.17 (1H, d, J = 14.8, H_i), 3.04 (1H, d, J = 14.8, H_m), 3.01 (1H, dd, J = 14.8, 13.3, H_g), 2.94-2.85 (1H), 2.79 (1H, dd, J = 14.8, 12.0, $H_{g'}$), 2.75-2.65 (1H), 2.59 (1H, d, J = 14.8, $H_{i'}$), 2.54-2.44 (1H), 2.28 (1H, d, J = 14.8, $H_{m'}$), 2.18-1.88 (3H), 1.78-1.03 (14H), 0.68 (3H, d, J = 6.4, H_q), 0.63 (3H, d, J = 6.4, H_s). It was not possible to unambiguously assign the alkyl region of macrocycle (H_F-H_L, H_O-H_Q).

δ_C ($CDCl_3$, 101 MHz) 169.0, 162.3, 158.7, 158.5, 158.4, 157.9, 157.4, 137.9, 137.8, 137.1, 137.0, 136.6, 136.1, 134.4, 134.2, 133.3, 133.2, 132.0, 131.5, 131.5, 131.2, 131.1, 131.0, 130.9, 130.2, 129.9, 129.8, 129.6, 128.9, 128.8, 128.7, 128.6, 128.6, 127.7, 127.6, 126.2, 126.2, 121.8, 120.7, 120.4, 119.0, 114.5, 114.2, 70.6, 69.6, 68.0, 57.2, 65.9, 44.0, 42.8, 35.7, 34.2, 30.3, 29.5, 28.4, 28.2, 27.9, 25.4, 25.3, 25.0, 24.8, 20.8, 20.8, 15.3.

64 signals corresponding to all 53 environments (11 doublets coupling to ^{31}P).

$\delta_{31P\{1H\}}$ ($CDCl_3$, 202 MHz) 26.2.

δ_{31P} ($CDCl_3$, 202 MHz) 26.2 (1P, broad quin.d, J 12.6, 3.6).

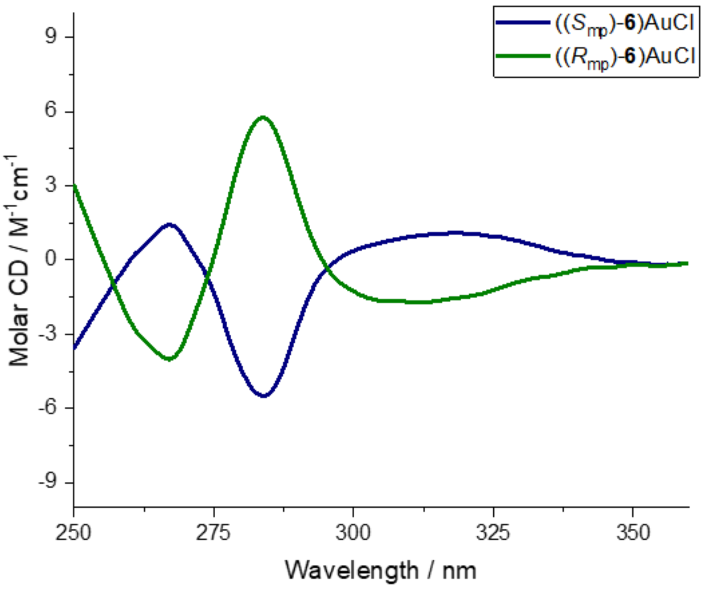

Figure S49: Circular Dichroism spectra of [Au((*R*$_{mp}$)-**6**)(Cl)] (32.1 µM) and [Au((*S*$_{mp}$)-**6**)(Cl)] (55.6 µM) at 293 K in $CHCl_3$. Both samples were 99 : 1 *er*.

^{1}H NMR Stack Plot Demonstrating the binding of Cu^I to the Rotaxane Framework

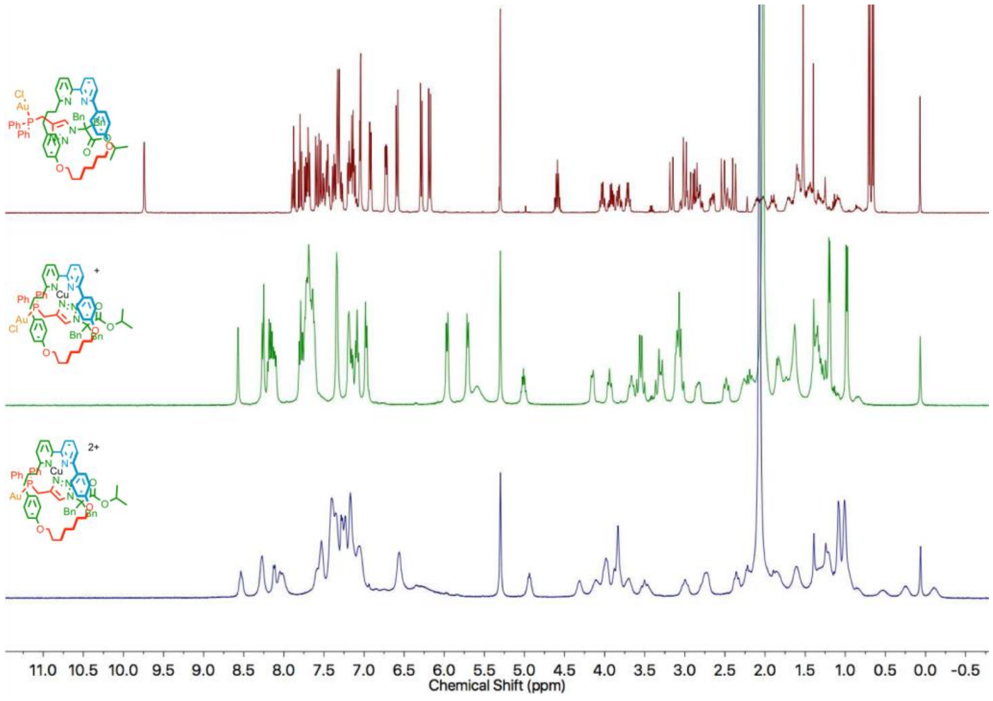

Figure S50: ^{1}H NMR stack plot ($CDCl_3$, 400MHz) of pre-catalyst [Au((*R*$_{mp}$)-**6**)(Cl)] (top) showing triazole ^{1}H shift on addition of $[Cu(MeCN)_4]PF_6$ (middle), and further shift upon chloride abstraction on addition of $AgSbF_6$ to form the active catalyst (bottom). Counter-ions omitted for clarity.

3. Crystallographic Data: Assignment of Relative and Absolute Stereochemistry of (*S*,*R*$_{mp}$)-4 and (*S*,*S*$_{mp}$)-4

Rotaxane (*S*,*R*$_{mp}$)-4 SCXRD Data (XRAY_ SRmp4.cif)

Enantiopure single crystals of (*S*,*R*$_{mp}$)-**4** were grown from (*S*,*R*$_{mp}$)-**4** (diastereomerically pure, *er* 99 : 1, see **Figure S52**, **Figure S53**) by vapor diffusion of *n*-pentane into a saturated solution in CH_2Cl_2. Data was collected at 100 K using a Rigaku 007 HF diffractometer equipped with a HYPix6000 enhanced sensitivity detector. Cell determination, data collection, data reduction, cell refinement and absorption correction were performed with CrysAlisPro. The crystal structure was solved using Olex2 with SHELXT dual methods and refined against F^2 with SHELXL refinement package using anisotropic thermal displacement parameters for all non-hydrogen atoms. H atoms were placed in calculated position and refined using a riding model.[7]

Note on the assignment of the absolute stereochemistry of (*S*,*R*$_{mp}$)-4: SC-XRD analysis of (*S*,*R*$_{mp}$)-**4** allows the relative covalent and mechanical stereochemistry to be directly determined as (*S**,*R**$_{mp}$). Furthermore, the absolute stereochemistry of the azide-derived stereocenter is known to be (*S*) due to the known stereochemistry of the starting material, (S)-**1**. This allows us to unambiguously assign the absolute stereochemistry of the sample to be (*S*,*R*$_{mp}$)-**4**.

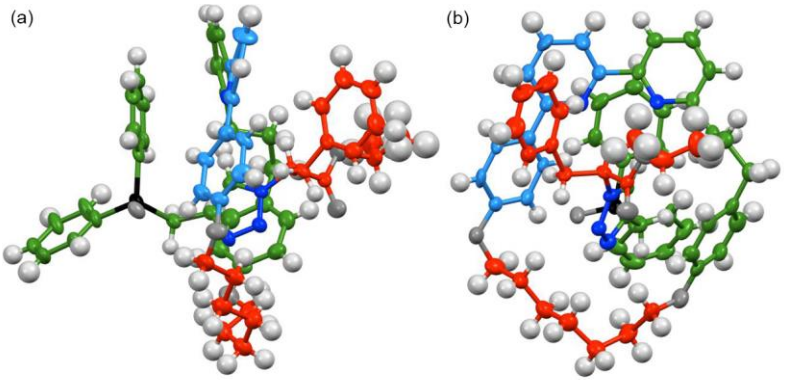

Figure S51: The SCXRD derived asymmetric unit of (*S*,*R*$_{mp}$)-**4** in thermal elipsoid (50%) representation from the (a) side and (b) the front.

Table S1. Crystal Structure Parameters for (S,R_{mp})-**4**.

Compound	(S,R_{mp})-**4**
Empirical formula	$C_{59}H_{62}N_5O_5P$
Formula weight	952.10
Temperature/K	100(2)
Crystal system	orthorhombic
Space group	$P2_12_12_1$
a/Å	12.69420(10)
b/Å	19.2864(2)
c/Å	20.6222(2)
α/°	90
β/°	90
γ/°	90
Volume/Å^3	5048.84(8)
Z	4
ρ_{calc}g/cm^3	1.253
μ/mm^{-1}	0.920
F(000)	2024.0
Crystal size/mm^3	0.05 × 0.05 × 0.05
Radiation	CuKα (λ = 1.54184)
2Θ range for data collection/°	6.274 to 140.93
Index ranges	-15 ≤ h ≤ 15, -23 ≤ k ≤ 23, -25 ≤ l ≤ 24
Reflections collected	96525
Independent reflections	9590 [R_{int} = 0.0620, R_{sigma} = 0.0295]
Data/restraints/parameters	9590/3/633
Goodness-of-fit on F^2	1.083
Final R indexes [I>=2σ (I)]	R_1 = 0.0462, wR_2 = 0.1146
Final R indexes [all data]	R_1 = 0.0495, wR_2 = 0.1166
Largest diff. peak/hole / e Å^{-3}	0.37/-0.25
Flack parameter	-0.008(8)

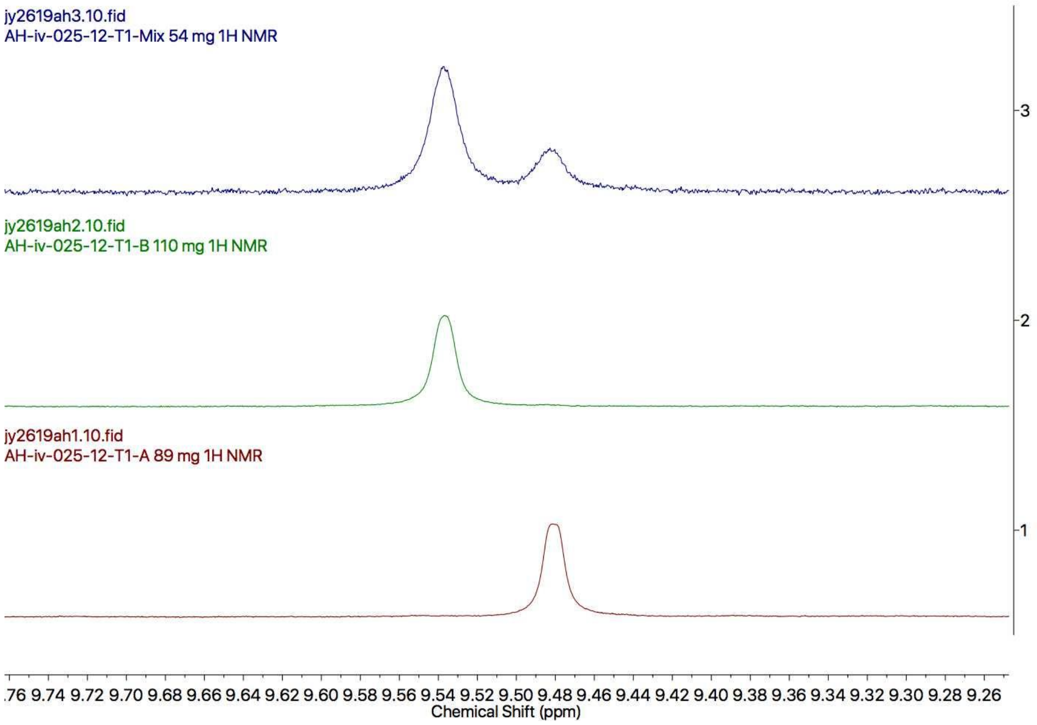

Figure S52: Partial ^{1}H NMR (400 MHz, $CDCl_3$) of a mixture of diastereomeric rotaxanes **4** (top), (*S*,*S*$_{mp}$)-**4** (middle) and the sample of (*S*,*R*$_{mp}$)-**4** (bottom) used for crystallisation to demonstrate the diastereomeric purity of the sample.

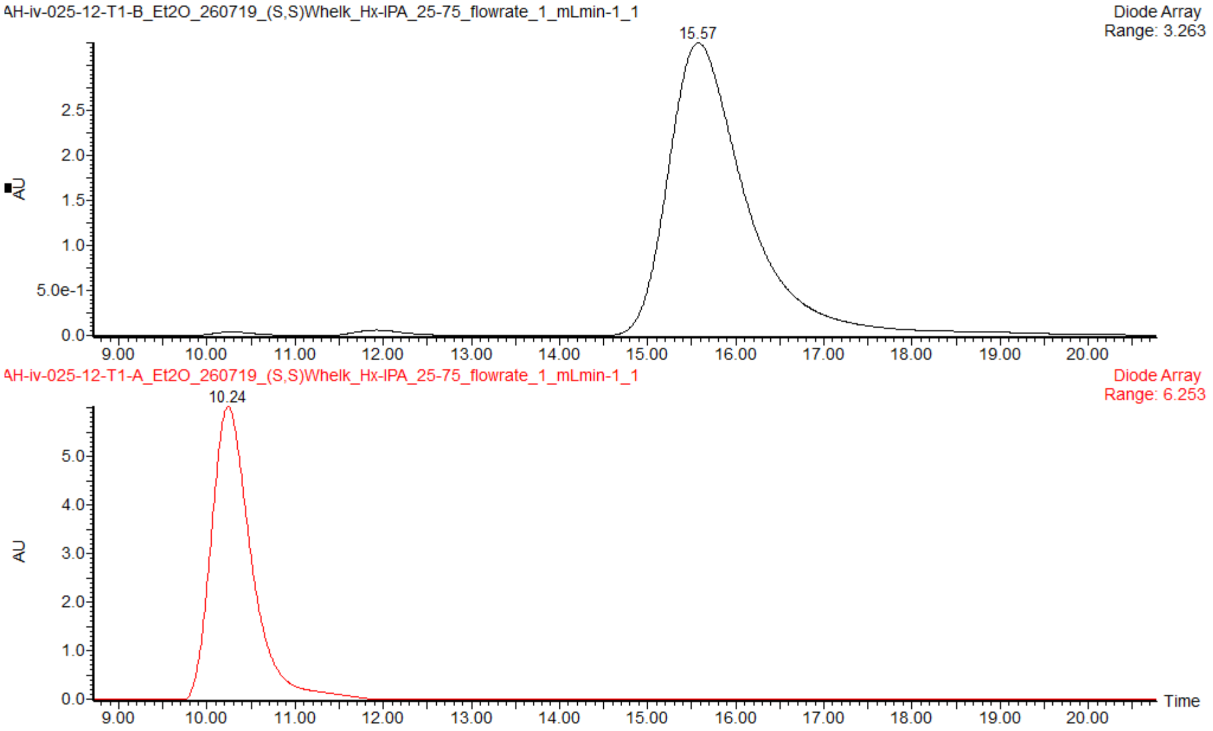

Figure S53: Chiral Stationary Phase HPLC ((*S*,*S*)Whelk column, isocratic *n*-hexane-isopropanol 25 : 75, 303 K, load solvent Et_2O, flowrate: 1.0 mLmin^{-1}) of (*S*,*S*$_{mp}$)-**4** (top) and the sample of (*S*,*R*$_{mp}$)-**4** (bottom) used for crystallisation, demonstrating the diastereo- and enantiopurity of the sample. Retention times (min): (*S*,*R*$_{mp}$)-**4** 10.2, (*R*,*S*$_{mp}$)-**4** 11.4, (*R*,*R*$_{mp}$)-**4** 12.1, (*S*,*S*$_{mp}$)-**4** 15.6.

Rotaxane (*S*,S_{mp})-4 SCXRD Data (XRAY_ SSmp4.cif)

Racemic single crystals of (*S**,$S*_{mp}$)-**4** were grown from a diastereomerically pure, scalemic sample of (*S*,S_{mp})-**4** (*er* 88 : 12; **Figure S55**, **Figure S56**, **Figure S57**) by vapor diffusion of *n*-pentane into a saturated solution in Et_2O. Data was collected at 100 K using a FRE+ HF diffractometer equipped with a Saturn 724+ enhanced sensitivity detector. Cell determination, data collection, data reduction, cell refinement and absorption correction were performed with CrysAlisPro. The crystal structure was solved using Olex2 with SHELXT dual methods and refined against F^2 with SHELXL refinement package using anisotropic thermal displacement parameters for all non-hydrogen atoms. H atoms were placed in calculated position and refined using a riding model.[7]

Important Note: Although the bulk sample of (*S*,S_{mp})-**4** was of high purity, as judged by NMR and HPLC, SC-XRD analysis revealed additional electron density that could not be accounted for by solvent or other impurities. Ultimately, we found the data was consistent with 25% of the molecules in the unit cell being peroxide oxidation product **X**; modelling a 50% occupancy of an O_2 unit in which one O atom is situated 1.32 Å from the benzylic position of one of the two molecules in the asymmetric unit produced a solution that agrees well with the diffraction data. Importantly, this solution is also chemically reasonable – the position found to be oxidized is activated to radical pathways, although it is not clear if the oxidation takes place during the peroxide work up (*vide supra*), or during crystallization due to adventitious oxygen (**Scheme S6**). Regardless, oxidation product **X** is not observed as an impurity by HPLC, NMR or MS analysis of bulk samples of either diastereomer of **4**, or its derivatives, suggesting it is present only in trace quantities. Thus, it appears that **X** is incorporated selectively during the crystallization process, resulting in enrichment of the crystal obtained in this minor species. Although this complicates the SC-XRD analysis of the sample of (*S**,$S*_{mp}$)-**4**, once this element of disorder is taken into consideration, the structure solution is of reasonable quality. Importantly, the data is consistent with the relative stereochemistry of the (*S**,$S*_{mp}$)-**4** diastereomer being epimeric with that determined by SC-XRD for (*S*,R_{mp})-**4** (*vide supra*). Finally, the major enantiomer in the scalemic sample could be determined to be (*S*,S_{mp})-**4** based on the known covalent stereochemistry of azide (*S*)-**1** from which it is derived.

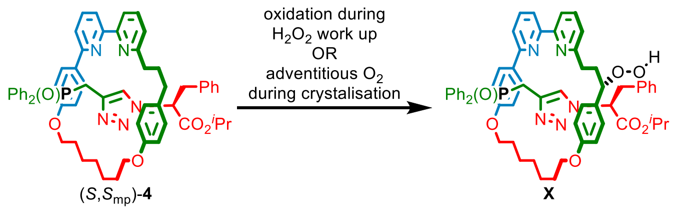

Scheme S6: Oxidation of (*S*,S_{mp})-**4** to give peroxide **X**, which is observed as an impurity by SC-XRD

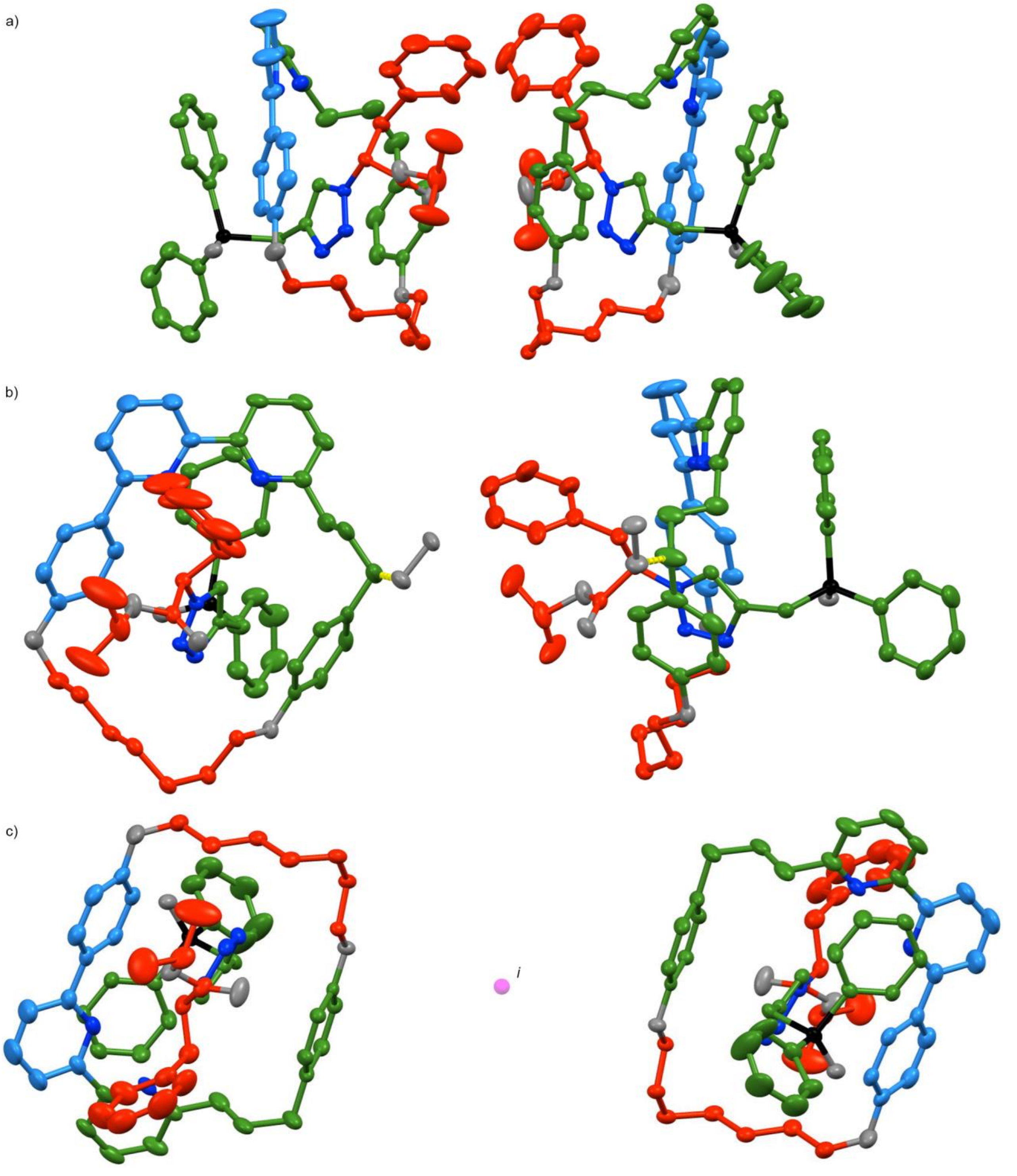

Figure S54: SCXRD structures of (*S**,S^*_{mp})-**4** (crystallised from 88 : 12 *er* mixture, *n*-pentane vapour diffusion into Et_2O) in ellipsoid representation (50% probability, H atoms omitted for clarity). a) The asymmetric unit showing (*S**,S^*_{mp})-**4** only (left hand molecule has 50% occupancy with peroxide **X**). b) Front and side views showing both (*S**,S^*_{mp})-**4** and **X**, the latter being represented by the presence of an O_2 unit 1.32 Å from the benzylic C. c) An enantiomeric pair of (*S**,S^*_{mp})-**4** related by an inversion centre, *i*, in the unit cell.

Table S2. X-ray diffraction data for (*S**,*S**$_{mp}$)-**4**

Compound	(S^*,S^*_{mp})-4
Empirical formula	$C_{118}H_{124}N_{10}O_{11}P_2$
Formula weight	1920.20
Temperature/K	100.0(2)
Crystal system	monoclinic
Space group	$P2_1/n$
a/Å	21.0723(2)
b/Å	18.6958(2)
c/Å	26.7361(2)
α/°	90
β/°	90.9780(10)
γ/°	90
Volume/Å^3	10531.51(17)
Z	4
ρ_{calc}g/cm^3	1.211
μ/mm^{-1}	0.107
F(000)	4080.0
Crystal size/mm^3	0.4 × 0.04 × 0.04
Radiation	Mo Kα (λ = 0.71075)
2Θ range for data collection/°	3.272 to 54.968
Index ranges	$-27 \le h \le 27$, $-24 \le k \le 24$, $-34 \le l \le 33$
Reflections collected	236269
Independent reflections	24147 [R_{int} = 0.0733, R_{sigma} = 0.0432]
Data/restraints/parameters	24147/207/1330
Goodness-of-fit on F^2	1.071
Final R indexes [I>=2σ (I)]	R_1 = 0.0770, wR_2 = 0.1780
Final R indexes [all data]	R_1 = 0.1072, wR_2 = 0.1916
Largest diff. peak/hole / e Å^{-3}	0.69/-0.51

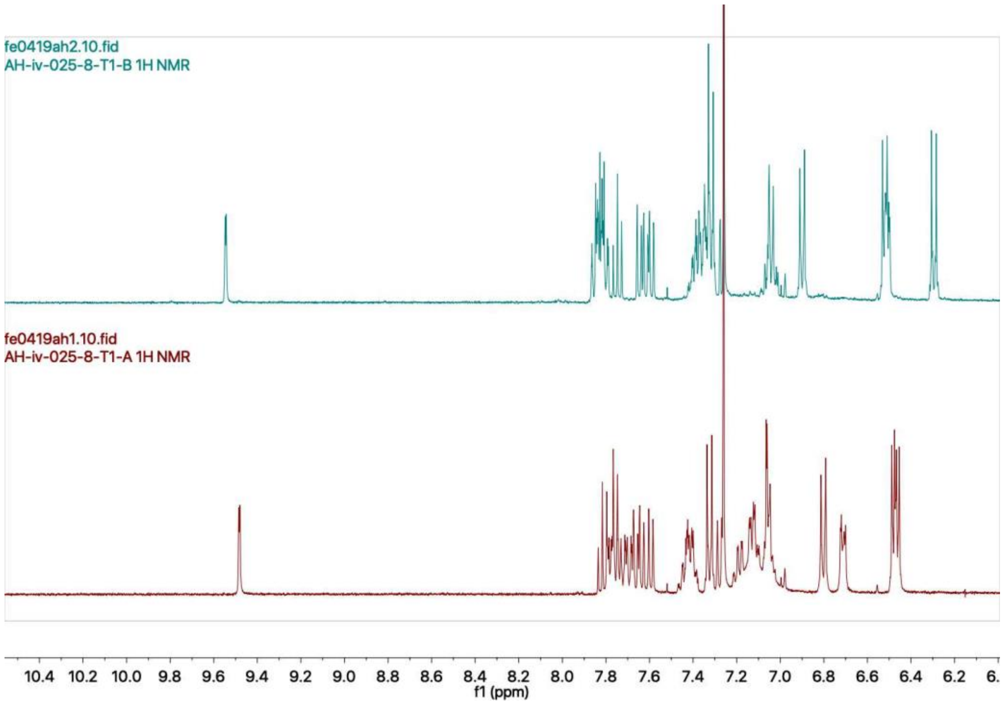

Figure S55: ^{1}H NMR (400 MHz, $CDCl_3$) of (S,R_{mp})-**4** (bottom) and the sample of (S,S_{mp})-**4** (top) used for crystallisation, demonstrating the diastereopurity of the sample.

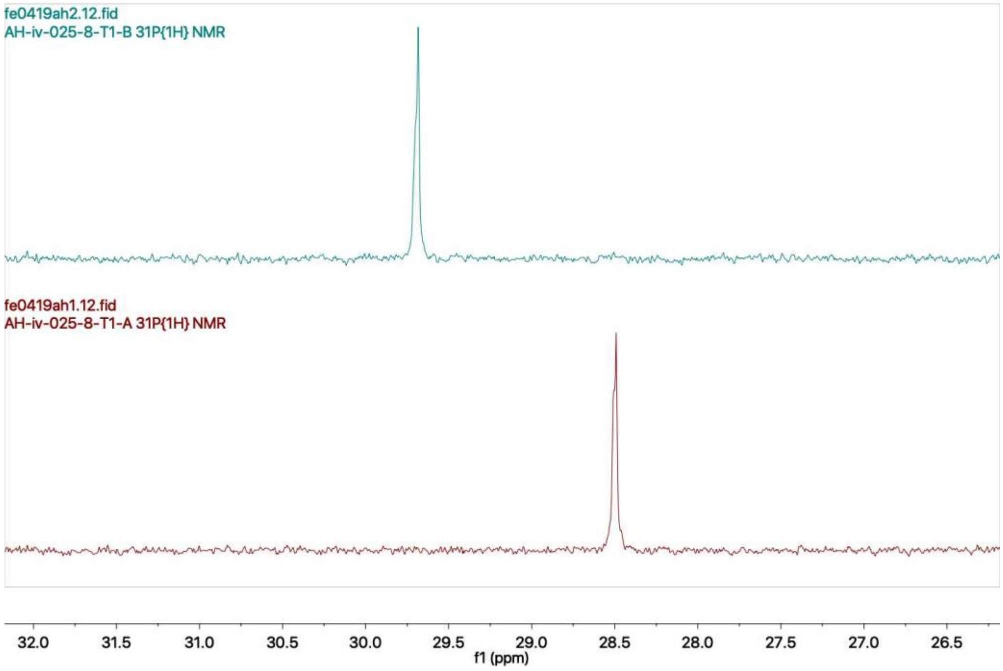

Figure S56: $^{31}P\{^1H\}$ (202 MHz, $CDCl_3$) NMR of (S,R_{mp})-**4** (bottom) and the sample of (S,S_{mp})-**4** (top) used for crystallisation, demonstrating the diastereopurity of the sample

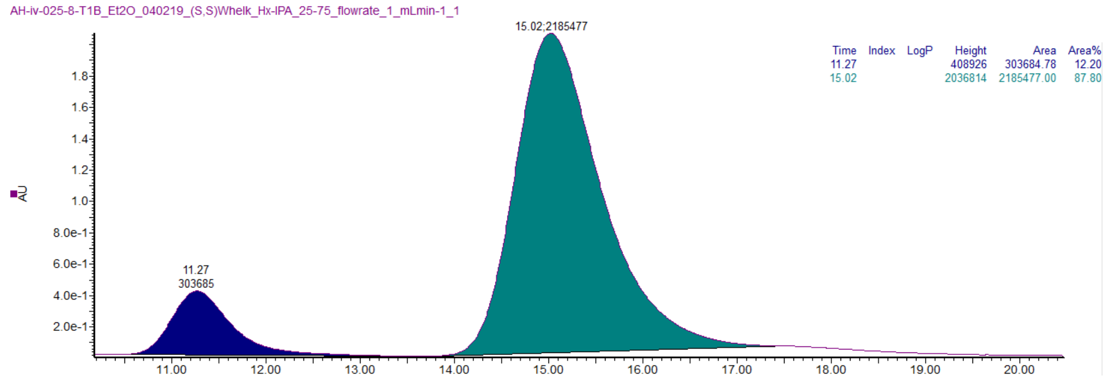

Figure S57: Chiral Stationary Phase HPLC ((*S*,*S*)Whelk column, isocratic *n*-hexane-isopropanol 25 : 75, 303 K, load solvent Et_2O, flowrate: 1.0 mLmin^{-1}) of the sample of (*S*,S_{mp})-**4** used for crystallisation demonstrating the enantiopurity of the sample to be 88 : 12 *er*. Retention times (min): (*R*,R_{mp})-**4** 11.3, (*S*,S_{mp})-**4** 15.0.

4. Synthesis of Cyclopropanation Substrates

Substituted 2-methyl-3-butyn-2-yl benzoate general procedure

Carboxylic acid (1.0 mmol, 1.0 eq.) was heated under reflux at 80 °C, under inert atmosphere, with $SOCl_2$ (0.58 mL, 8.0 mmol, 8.0 eq.) for 3 h. The reaction was cooled and excess $SOCl_2$ was removed *in vacuo*. The residue was cooled to 0 ˚C, and a solution of 2-methyl-3-butyn-2-ol (0.10 mL, 1.0 mmol, 1.0 eq.) and 4-(dimethylamino)pyridine (0.244 g, 2.0 mmol, 2.0 eq) in $CDCl_3$ (2.0 mL, 0.5 M concentration of 2-methyl-3-butyn-2-ol) was added dropwise. The resulting solution was stirred for 17 h at 35 ˚C. Solvent was removed under reduced pressure, and the residue was purified by column chromatography (SiO_2, petrol-Et_2O 0→5%).

2-methyl-3-butyn-2-yl benzoate, **7**[8]

Colourless oil (0.120 g, 0.63 mmol, 63%)

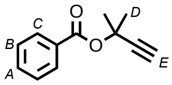

δ_H ($CDCl_3$, 400 MHz) 8.02 (2H, dd, *J* = 7.0, 2.0, $\boldsymbol{H}_C$), 7.55 (1H, t, *J* = 7.5, $\boldsymbol{H}_A$), 7.43 (2H, t, *J* = 7.0, $\boldsymbol{H}_B$), 2.58 (1H, s, $\boldsymbol{H}_E$), 1.83 (6H, s, $\boldsymbol{H}_D$).

δ_C ($CDCl_3$, 101 MHz) 165.0, 133.0, 131.0, 129.7, 128.4, 84.8, 72.7, 72.4, 29.2.

GCMS [$M^{+\cdot}$] m/z 188.2.

HR-EI-MS m/z 188.0831 $[M]^{+\cdot}$ (calc. m/z for $C_{12}H_{12}O_2$ 188.0832).

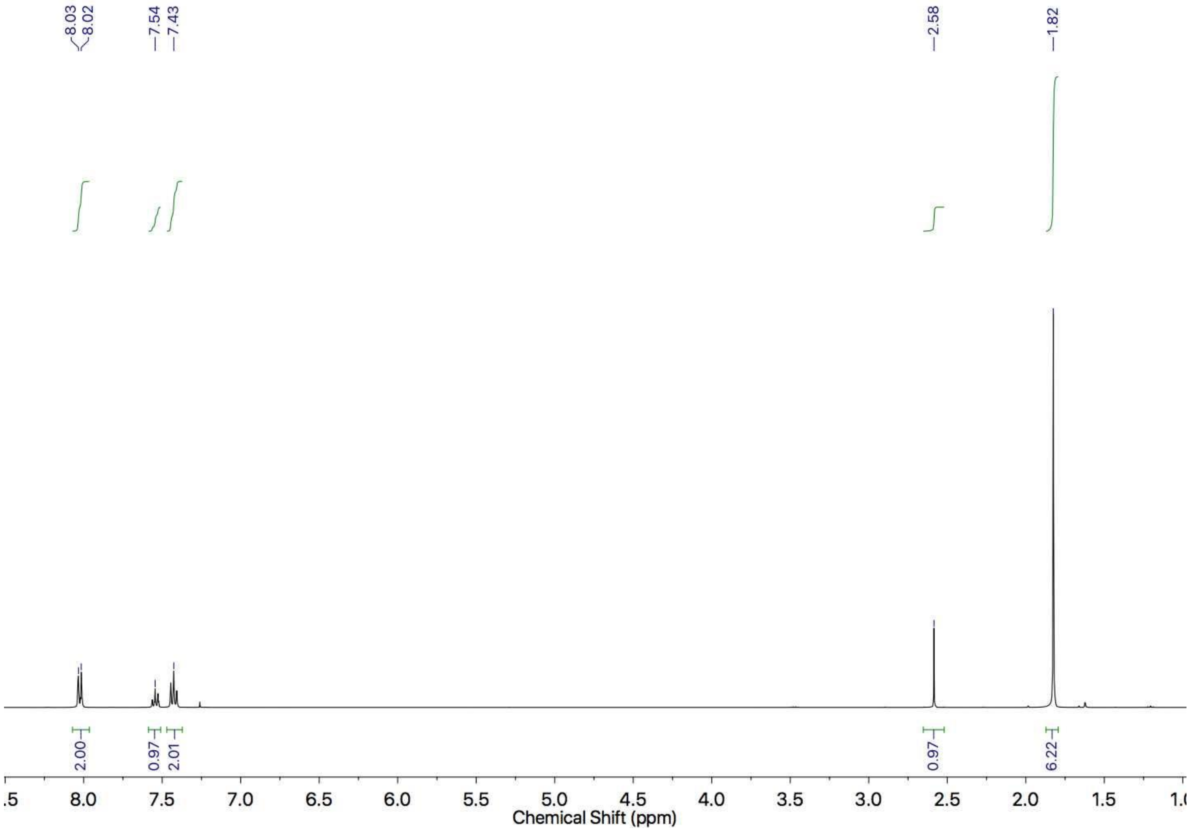

Figure S58: ^{1}H NMR (400 MHz, $CDCl_3$) of **7**.

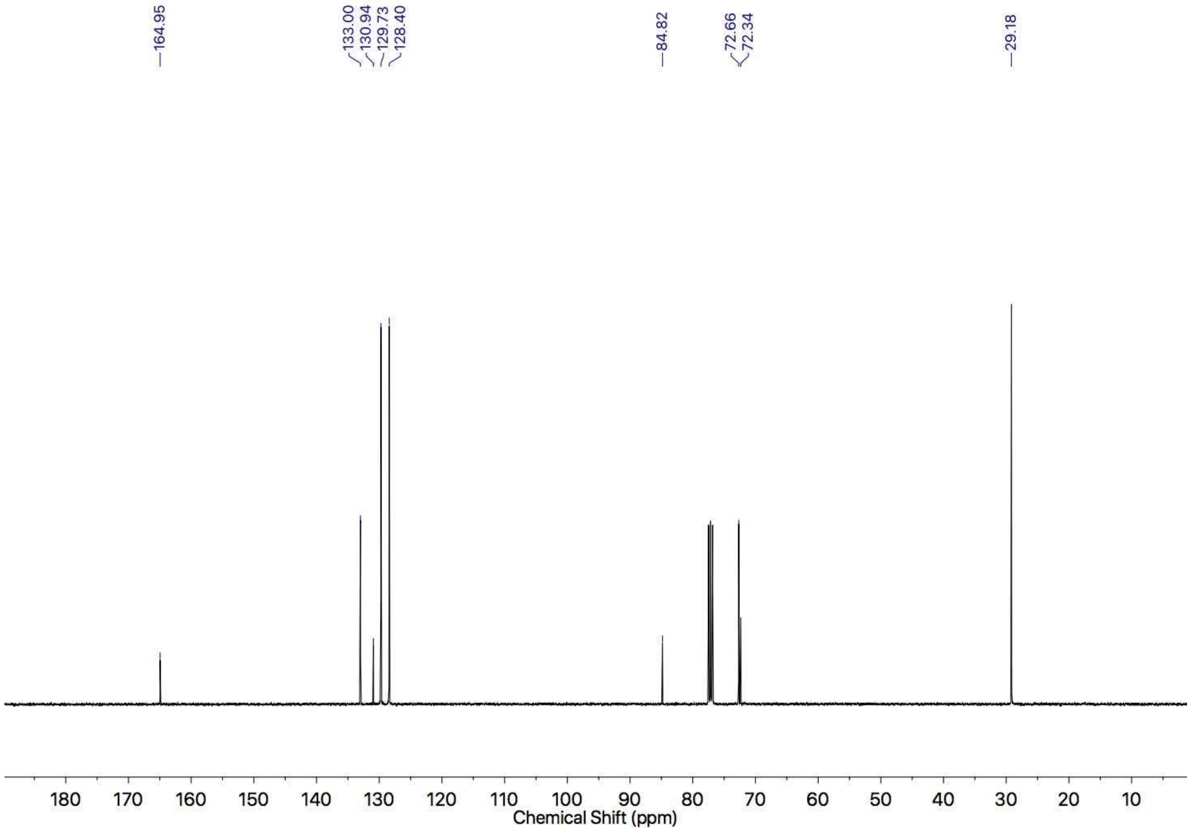

Figure S59: ^{13}C NMR (101 MHz, $CDCl_3$) of **7**.

2-methyl-3-butyn-2-yl pivaloate, **S3**[9,10]

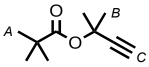

A solution of trimethylacetyl chloride (1.23 g, 10.2 mmol, 1.0 eq.) (in 6.0 mL CH_2Cl_2), was added dropwise to a solution of 2-methyl-3-butyn-2-ol (0.85 g, 10.2 mmol, 1.0 eq.) and 4-(dimethylamino)pyridine (1.87 g, 15.3 mmol, 1.5 eq.) in $CHCl_3$ (6.0 mL) at 0 ˚C. The resulting solution was stirred at 30 ˚C for 16 h. The reaction mixture was diluted with CH_2Cl_2, washed with $NaHCO_3$, and extracted in CH_2Cl_2 and Et_2O. The combined organic phases were dried ($MgSO_4$) and the solvent was removed *in vacuo*. The residue was purified by column chromatography (SiO_2, petrol-Et_2O 0→50%), yielding a pale colourless oil (0.996 g, 6.65 mmol, 58%).

δ_H ($CDCl_3$, 400 MHz) 2.48 (1H, s, $\boldsymbol{H}_C$), 1.64 (6H, s, $\boldsymbol{H}_B$), 1.16 (9H, s, $\boldsymbol{H}_A$).

δ_C ($CDCl_3$, 101 MHz) 176.8, 85.0, 72.0, 71.2, 39.1, 28.9, 27.1.

GC-EI-MS m/z 168.1 [$M^{+\cdot}$].

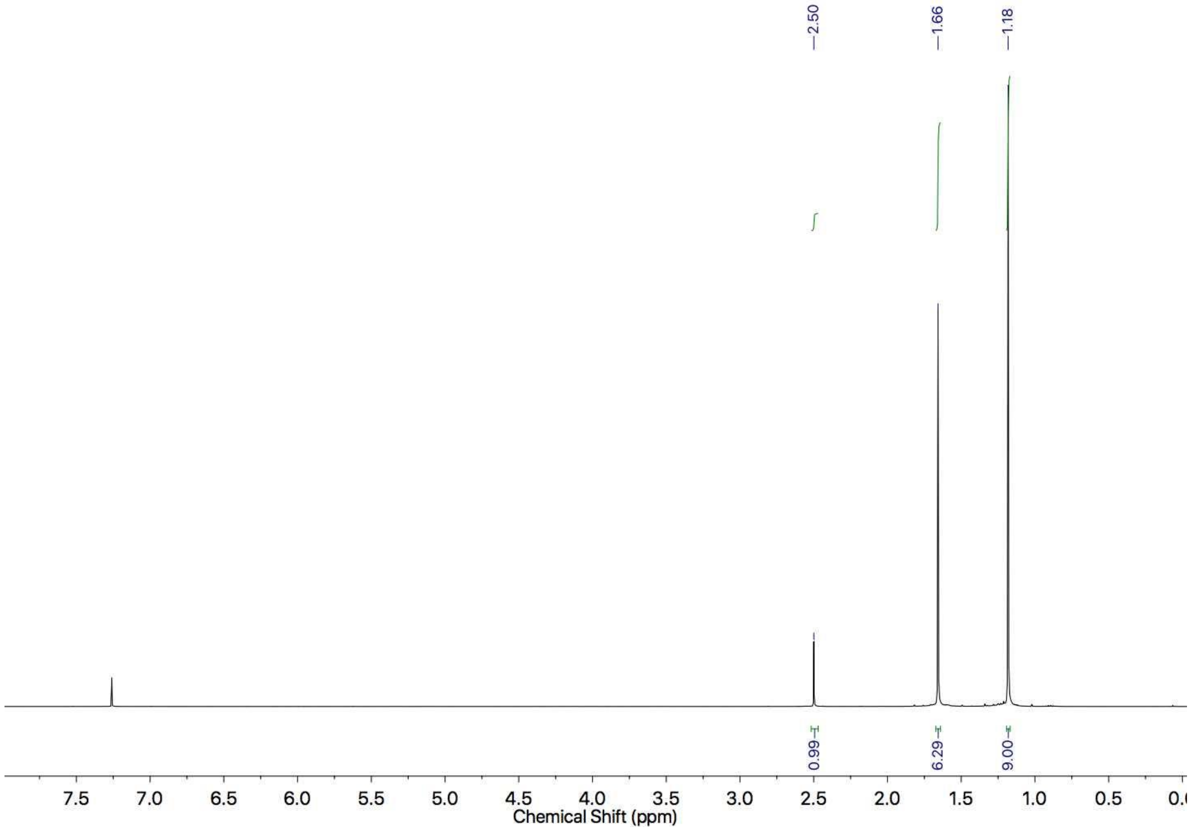

Figure S60: ^{1}H NMR (400 MHz, $CDCl_3$) of **S3**.

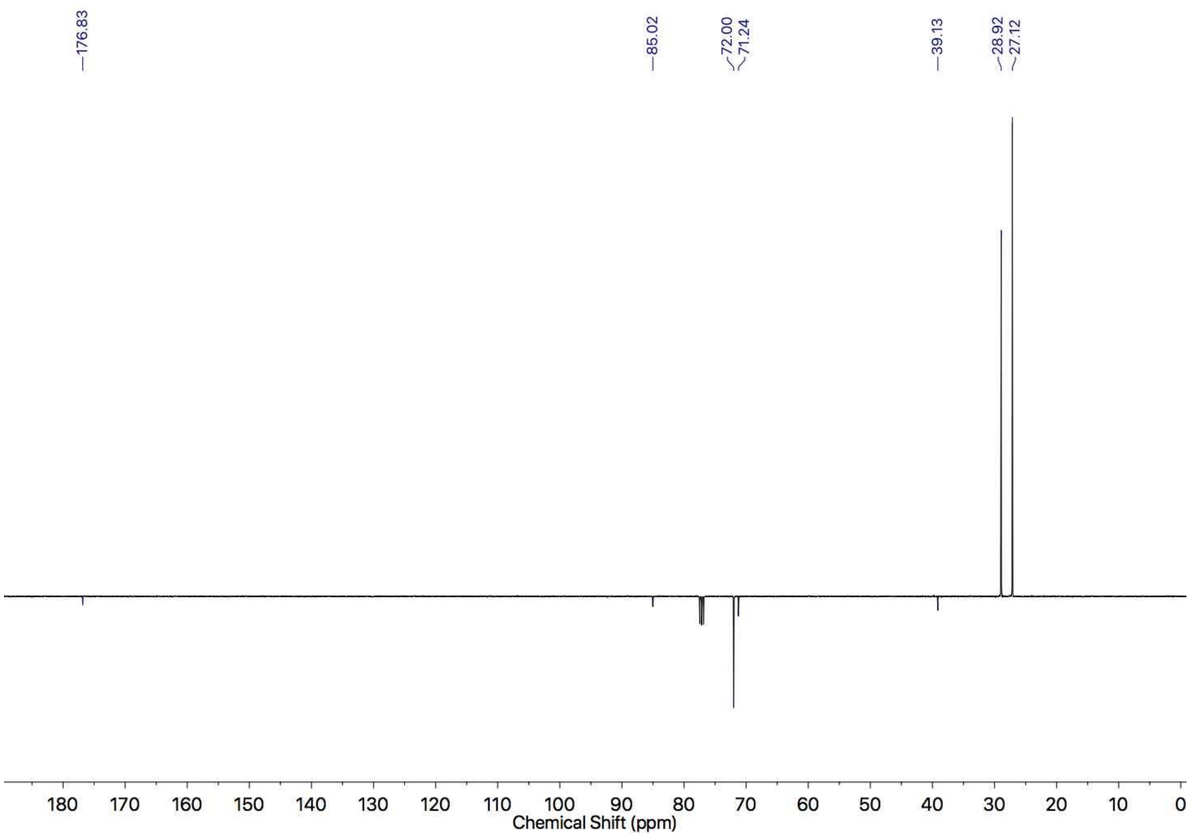

Figure S61: JMOD NMR (101 MHz, $CDCl_3$) of **S3**.

2-methyl-3-butyn-2-yl phenylacetate, **S4**[11]

Phenylacetic acid (0.554 g, 4.0 mmol, 1.0 eq.) was heated under reflux at 80 °C, under inert atmosphere, with $SOCl_2$ (2.32 mL, 32.0 mmol, 8.0 eq.) for 3 h. The reaction was cooled and excess $SOCl_2$ was removed *in vacuo*. The residue was cooled to 0 ˚C, and a solution of 2-methyl-3-butyn-2-ol (0.44 mL, 4.4 mmol, 1.1 eq.) and 4-(dimethylamino)pyridine (0.244 g, 2.0 mmol, 0.5 eq) in $CDCl_3$ (4.0 mL) was added dropwise. The resulting solution was stirred for 16 h at rt. The solvent was removed *in vacuo*, and the residue was purified by column chromatography (SiO_2, petrol-Et_2O 10→30%) yielding a colourless oil (200 mg, 0.989 mmol, 25%).

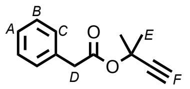

δ_H ($CDCl_3$, 400 MHz) 7.35-7.24 (5H, m, ***H***$_A$, ***H***$_B$, ***H***$_C$), 3.60 (2H, s, ***H***$_D$), 2.52 (1H, s, ***H***$_F$), 1.67 (6H, s, ***H***$_E$).

δ_C ($CDCl_3$, 101 MHz) 169.9, 134.1, 129.4, 128.6, 127.1, 84.7, 72.6, 72.3, 42.1, 29.0.

HR-EI-MS m/z 202.1217 [$M^{+\cdot}$] (calc. m/z for $C_{13}H_{14}O_2$ 202.0994).

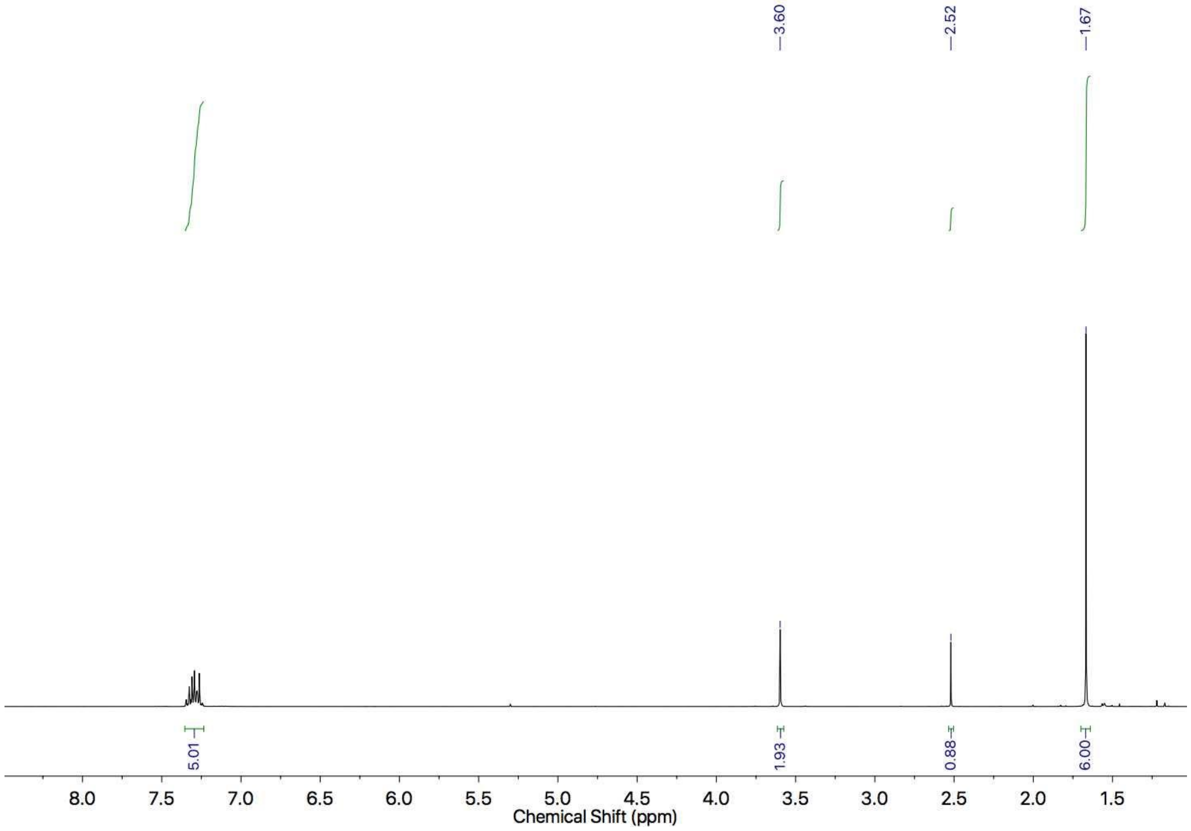

Figure S62: ^{1}H NMR (400 MHz, $CDCl_3$) of **S4**.

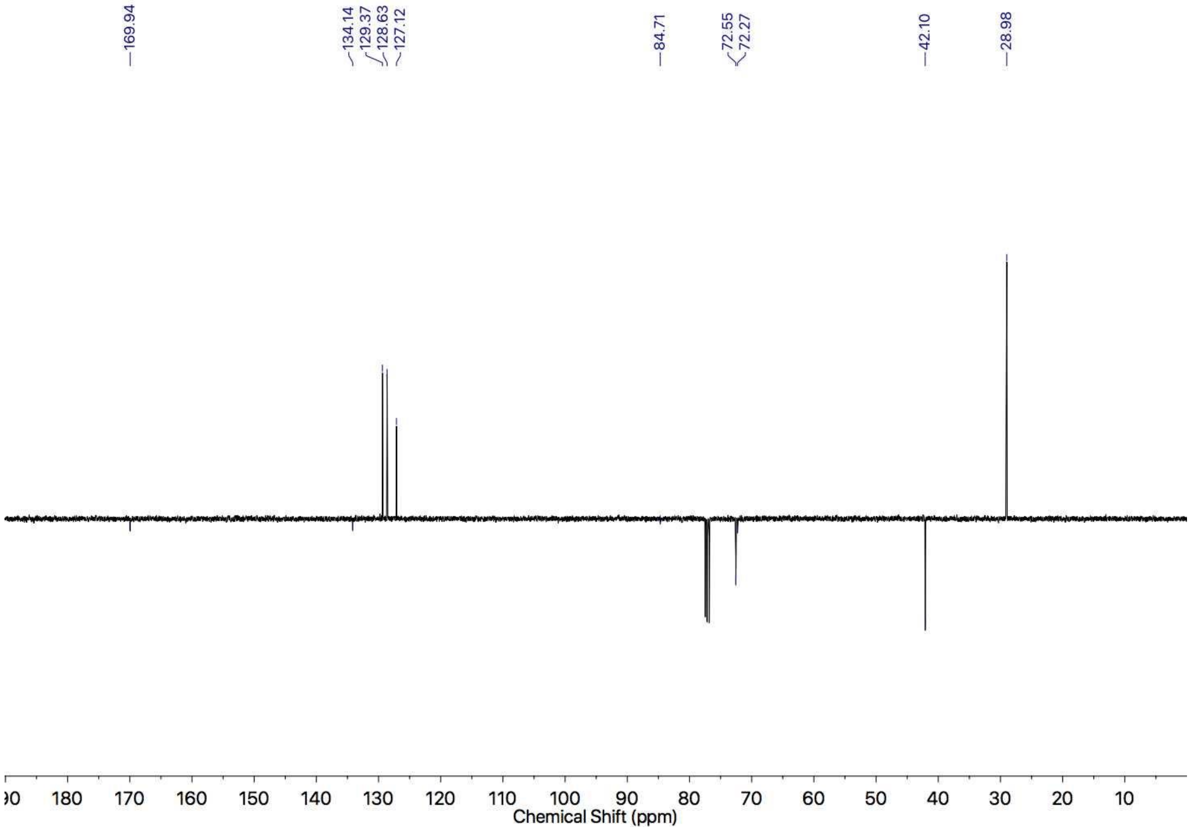

Figure S63: JMOD NMR (101 MHz, $CDCl_3$) of **S4**.

2-methyl-3-butyn-2-yl 4-trifluoromethylbenzoate, **S5**

White solid (128 mg, 0.50 mmol, 50%) m.p. 32-34 °C

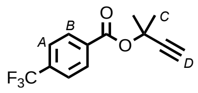

δ_H ($CDCl_3$, 400 MHz) 8.11 (2H, d, *J* = 8.1, $\boldsymbol{H}_B$), 7.70 (2H, d, *J* = 8.2, $\boldsymbol{H}_A$), 2.61 (1H, s, $\boldsymbol{H}_D$), 1.84 (6H, s, $\boldsymbol{H}_C$).

δ_C ($CDCl_3$, 101 MHz) 163.7, 134.5 (q, *J* = 32.7), 134.3 (q, *J* = 18.2), 130.1, 125.4 (q, *J* = 3.7), 122.4, 84.4, 73.1, 73.2, 29.1.

δ_F ($CDCl_3$, 376 MHz) 63.3 (s).

$\delta_{F\{H\}}$ ($CDCl_3$, 376 MHz) 63.3.

HR-EI-MS m/z 256.0701 [$M^{+\cdot}$] (calc. m/z for $C_{13}H_{11}O_2F_3$ 256.0706).

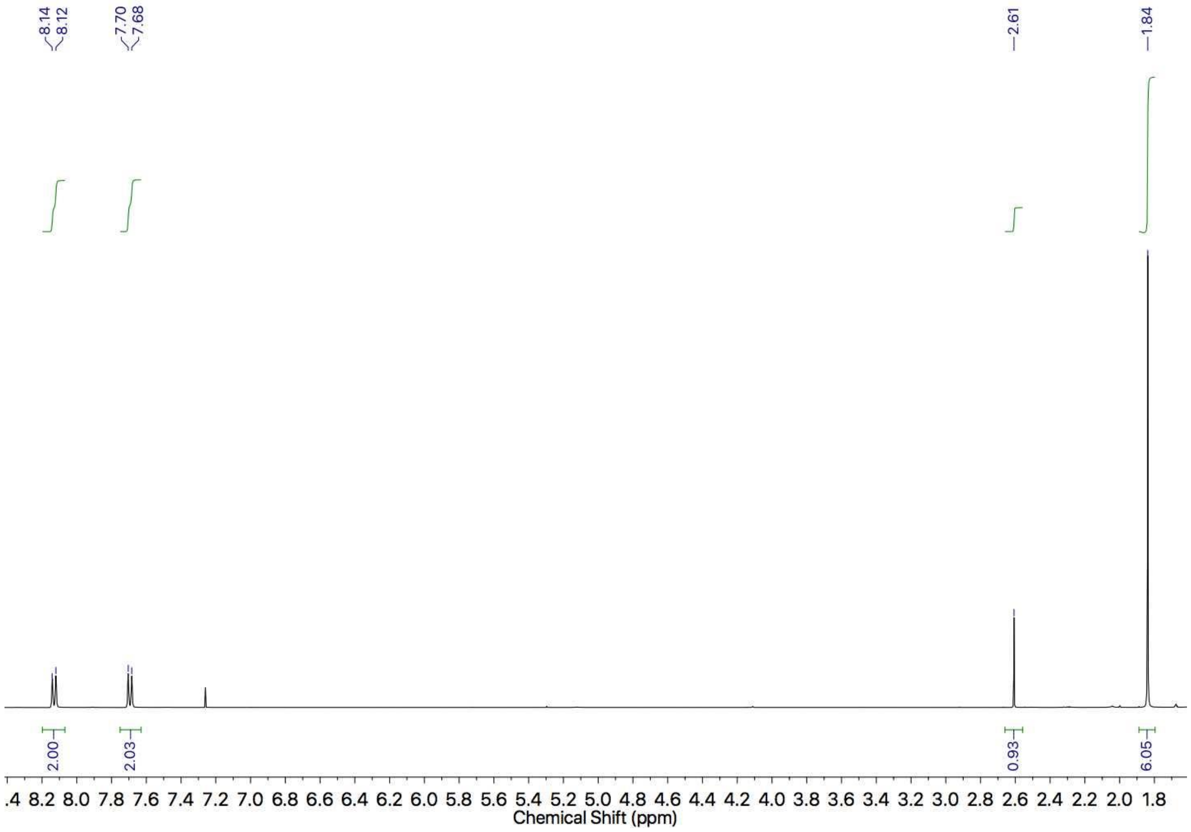

Figure S64: ^{1}H NMR (400 MHz, $CDCl_3$) of **S5**.

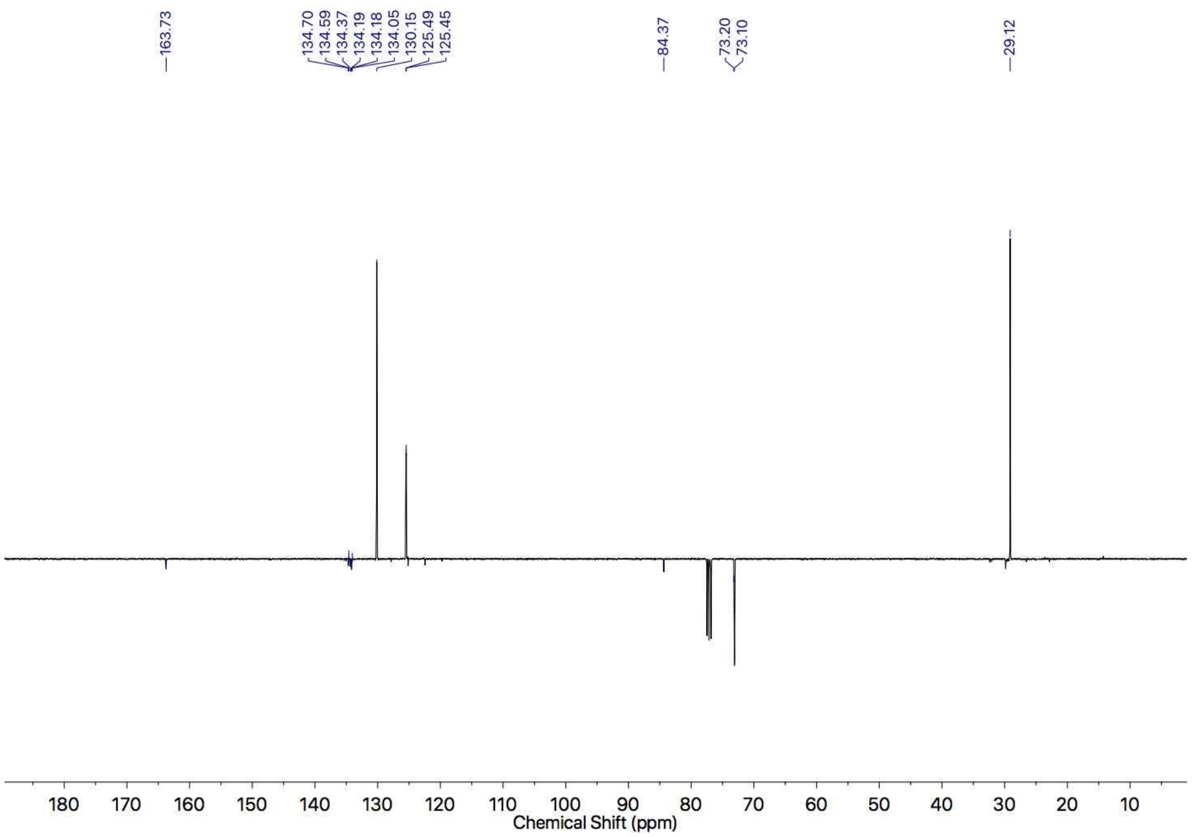

Figure S65: JMOD NMR (101 MHz, $CDCl_3$) of **S5**.

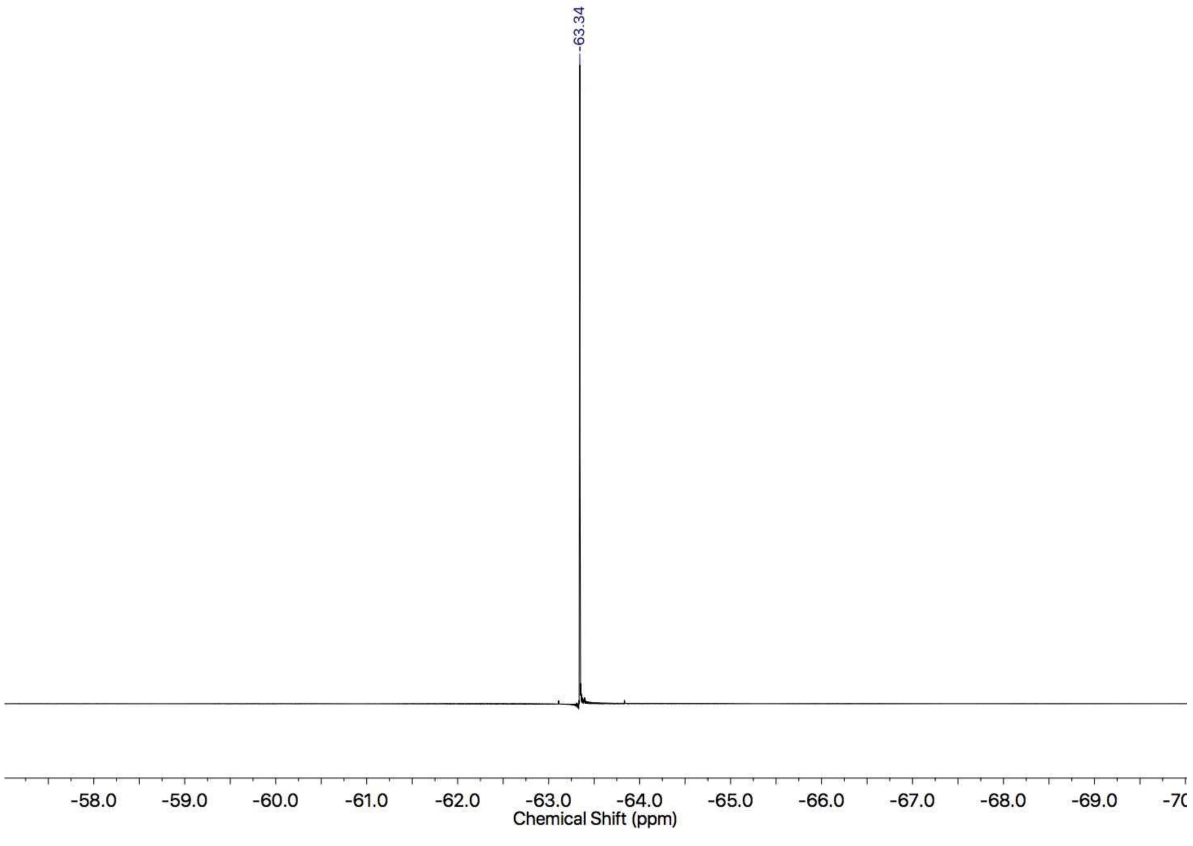

Figure S66: $^{19}F\{^1H\}$ NMR (376 MHz, $CDCl_3$) of **S5**.

2-methyl-3-butyn-2-yl 4-methoxybenzoate, **S6**[12]

Colourless oil (209 mg, 0.96 mmol, 96%)

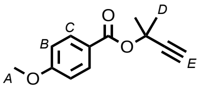

δ_H ($CDCl_3$, 400 MHz) 7.98 (2H, dt, *J* = 9.1, 2.4, ***H***C), 6.91 (2H, dt, *J* = 9.1, 2.4, ***H***B), 3.86 (3H, s, ***H***A), 2.57 (1H, s, ***H***E), 1.81 (6H, s, ***H***D).

δ_C ($CDCl_3$, 101 MHz) 164.8, 163.5, 131.8, 123.4, 113.7, 85.1, 72.5, 72.0, 55.6, 29.2.

HR-EI-MS m/z 318.0933 $[M]^{+\cdot}$ (calc. m/z for $C_{13}H_{14}O_3$ 218.0937).

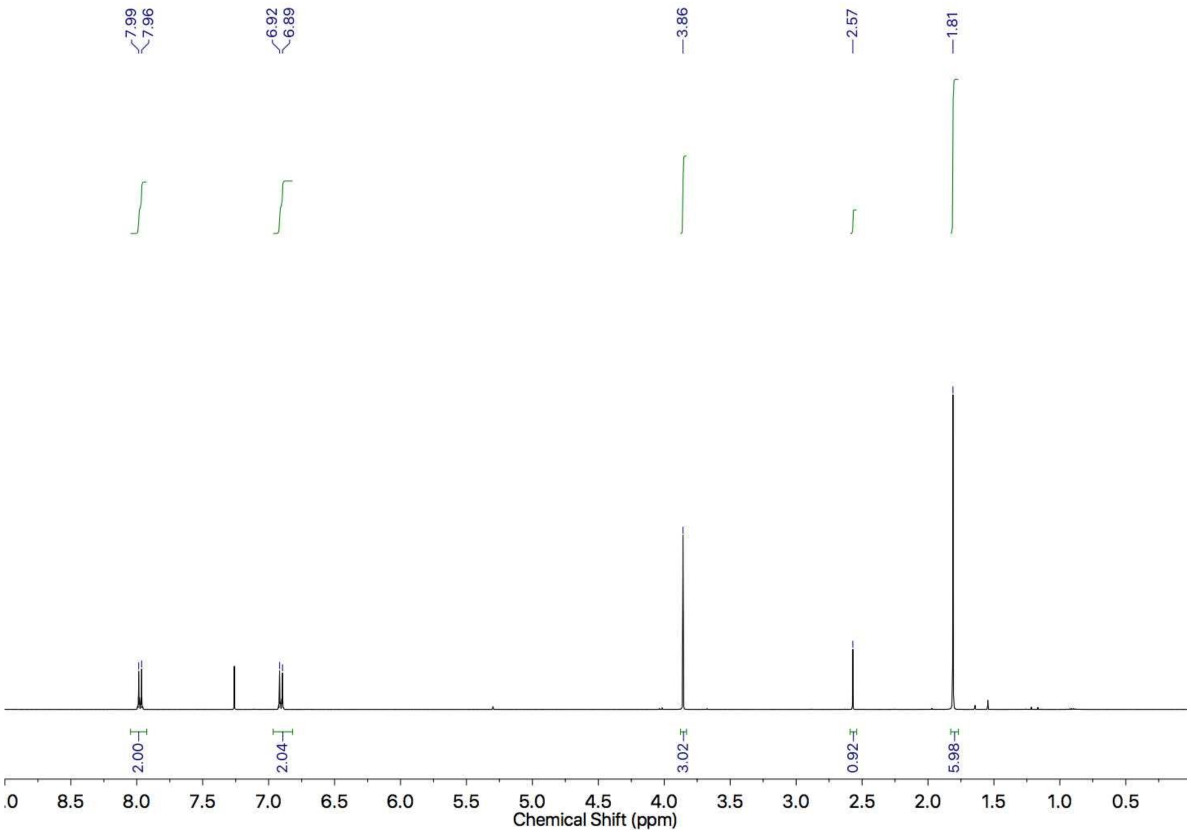

Figure S67: ^{1}H NMR (400 MHz, $CDCl_3$) of **S6**.

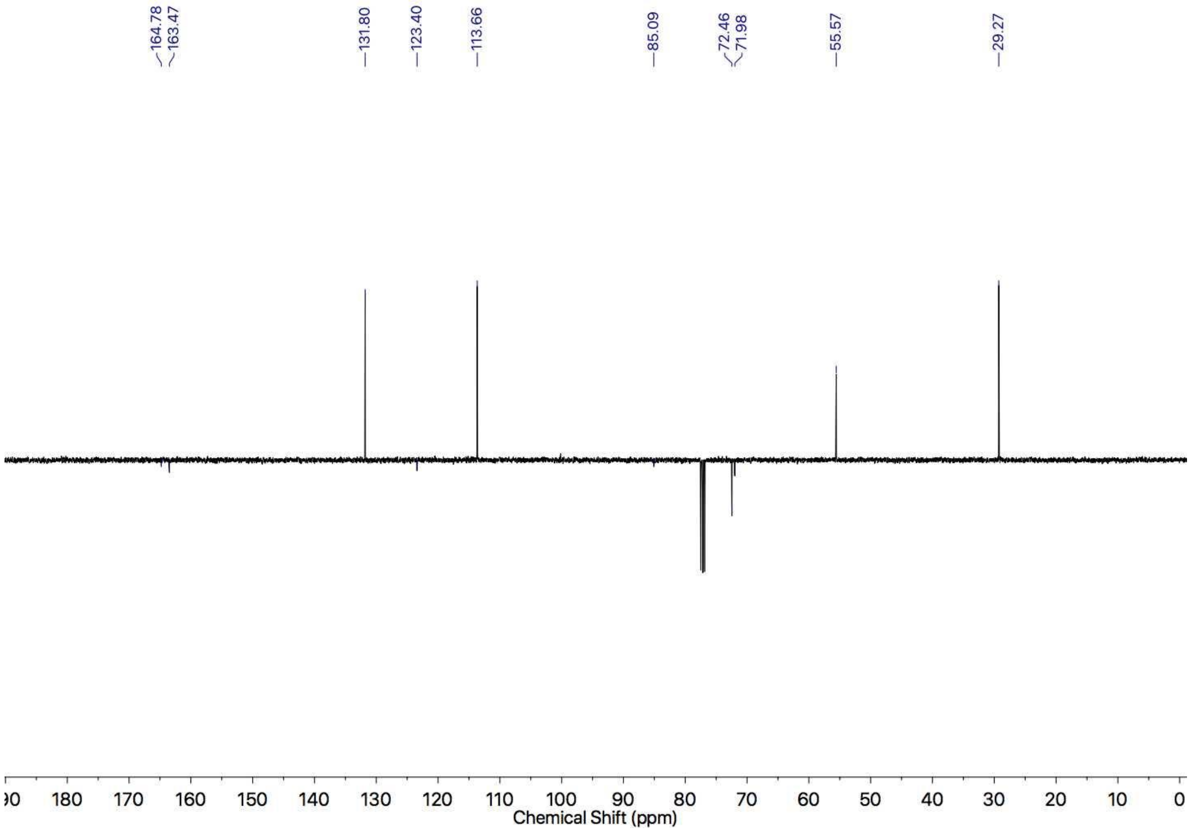

Figure S68: JMOD NMR (101 MHz, $CDCl_3$) of **S6**.

2-methyl-3-butyn-2-yl 4-tertbutylbenzoate, **S7**

Colourless oil (240 mg, 0.982 mmol, 98%)

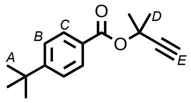

δ_H ($CDCl_3$, 400 MHz) 7.95 (2H, dt, *J* = 8.8, 2.1, ***H***C), 7.45 (2H, dt, *J* = 8.8, 2.1, ***H***B), 2.57 (1H, s, ***H***E), 1.81 (6H, s, ***H***D), 1.33 (18H, s, ***H***A).

δ_C ($CDCl_3$, 101 MHz) 165.0, 156.7, 129.6, 128.2, 125.4, 85.0, 72.5, 72.1, 35.2, 31.3, 29.2.

HR-EI-MS m/z 244.1457 $[M]^{+\cdot}$ (calc. m/z for $C_{16}H_{20}O_2$ 244.1458).

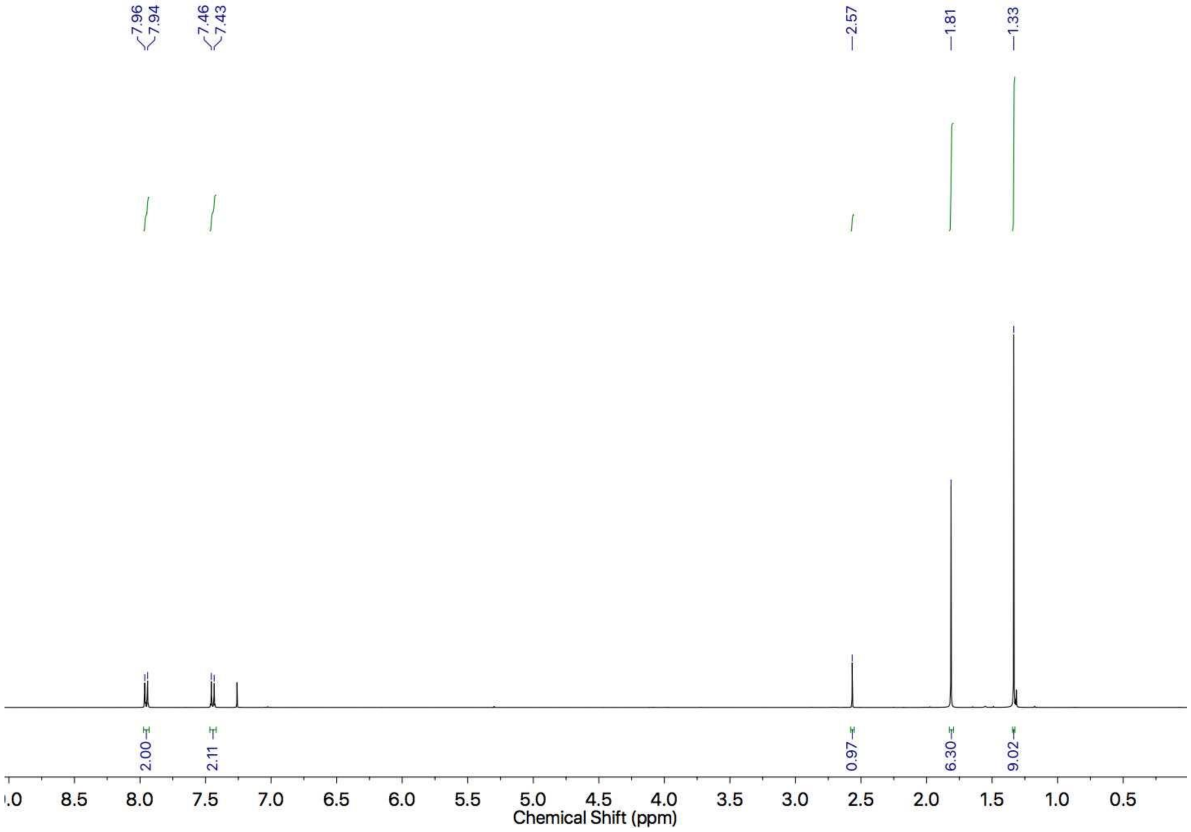

Figure S69: ^{1}H NMR (400 MHz, $CDCl_3$) of **S7**.

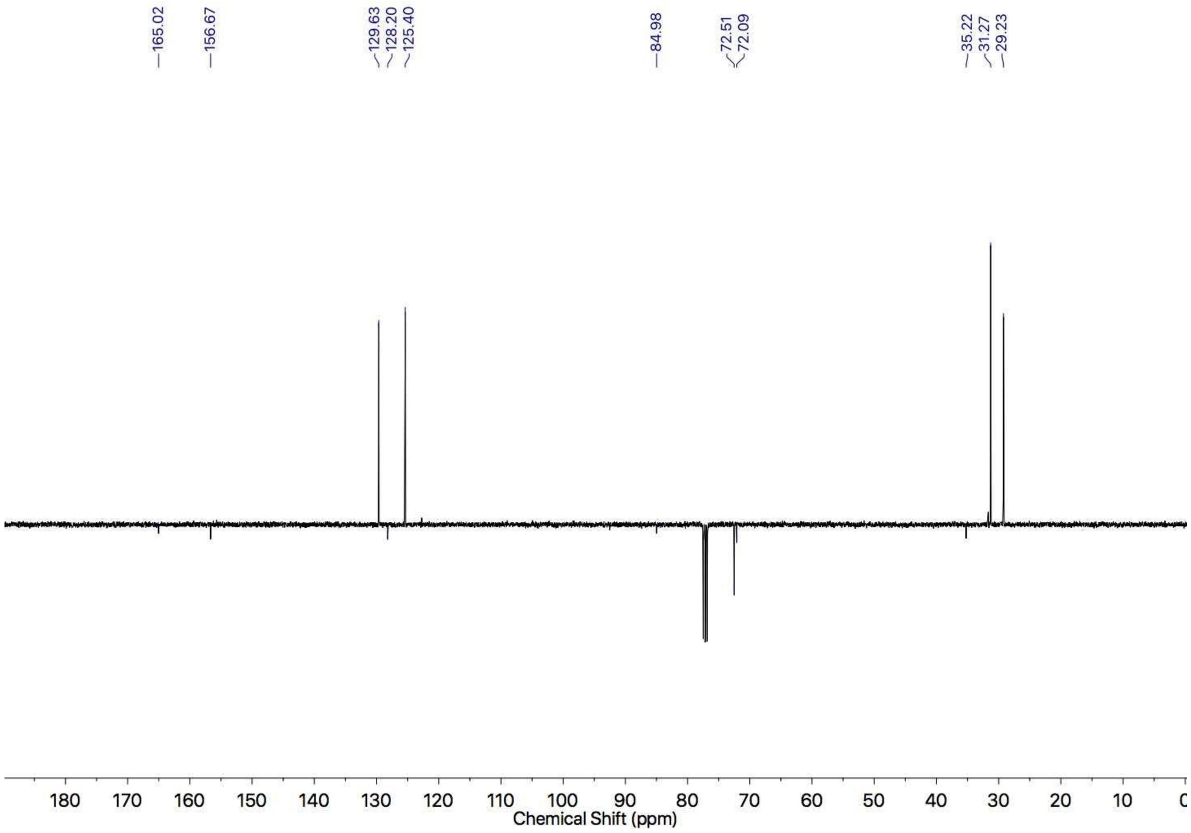

Figure S70: JMOD NMR (101 MHz, $CDCl_3$) of **S7**.

2-methyl-3-butyn-2-yl 3,5-ditertbutylbenzoate, **S8**

Colourless oil (188 mg, 0.626 mmol, 63%)

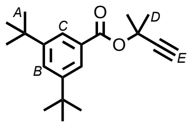

δ$_H$ ($CDCl_3$, 400 MHz) 7.88 (2H, d, *J* = 1.8, ***H***$_C$), 7.62 (1H, t, *J* = 1.8, ***H***$_B$), 2.58 (1H, s, ***H***$_E$), 1.83 (6H, s, ***H***$_D$), 1.34 (18H, s, ***H***$_A$).

δ$_C$ ($CDCl_3$, 101 MHz) 165.8, 151.1, 130.2, 127.3, 123.9, 85.1, 72.5, 72.1, 35.1, 31.5, 29.2.

HR-EI-MS m/z 300.2080 $[M]^{+\cdot}$ (calc. m/z for $C_{20}H_{28}O_2$ 300.2084).

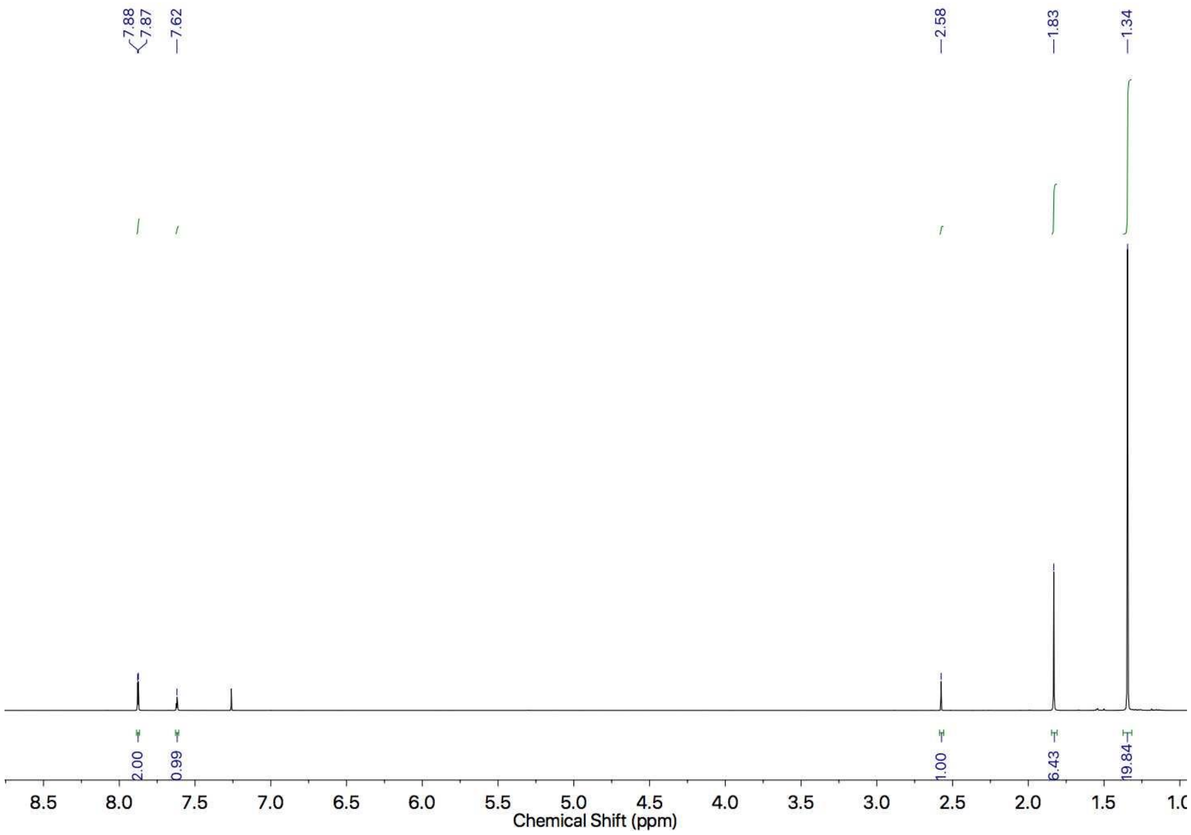

Figure S71: ^{1}H NMR (400 MHz, $CDCl_3$) of **S8**.

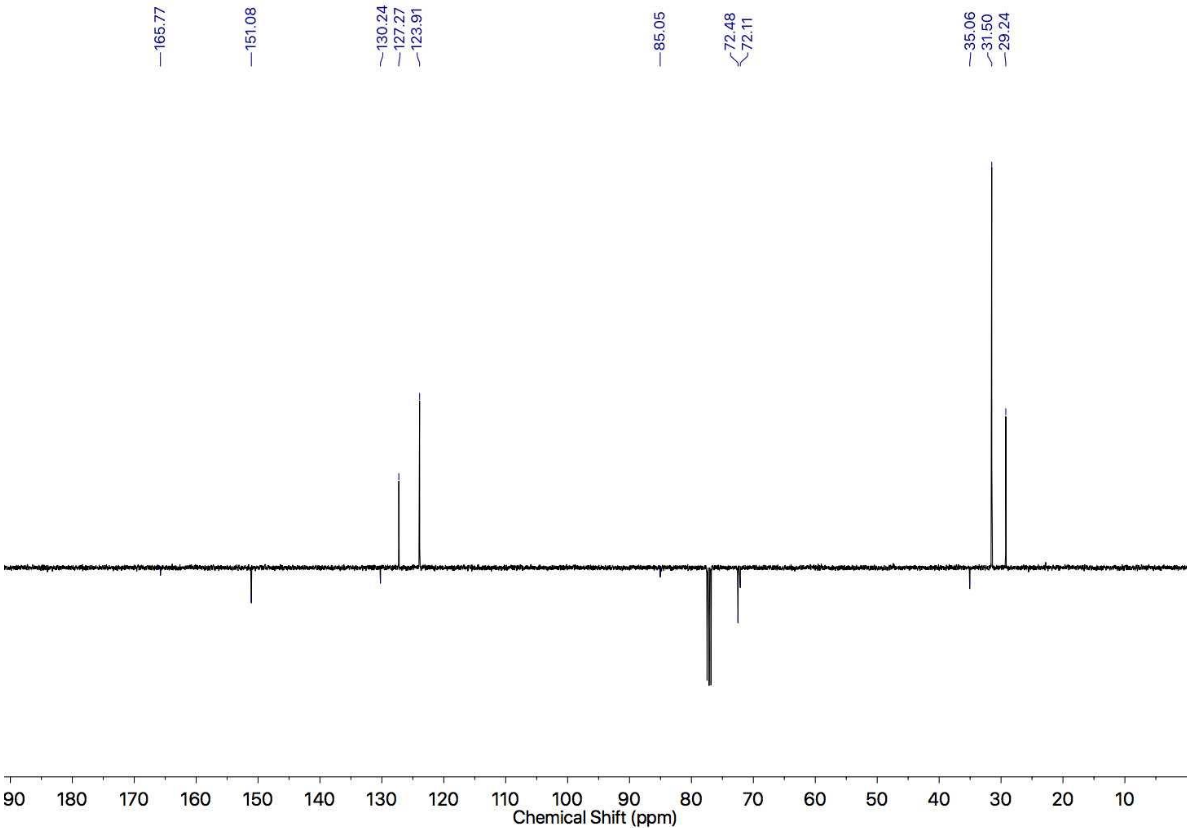

Figure S72: JMOD NMR (101 MHz, $CDCl_3$) of **S8**.

5. Cyclopropanation Reactions

Screening of reaction conditions

Optimum catalytic conditions were determined by screening the reaction of **7** with **8** mediated by [Au(**6**)(Cl)] for a range of solvents, additives and temperatures (**Table S3**). Unless otherwise stated, the stereopurity of the rotaxane catalyst used was 97 : 3 *er*. The best conditions (entry 9) obtained are given in the general procedure (*vide infra*) and were used throughout the experiments that follow.

Table S3. Screening of conditions for the reaction of 7 and 8 mediated by [Au(6)(Cl)]

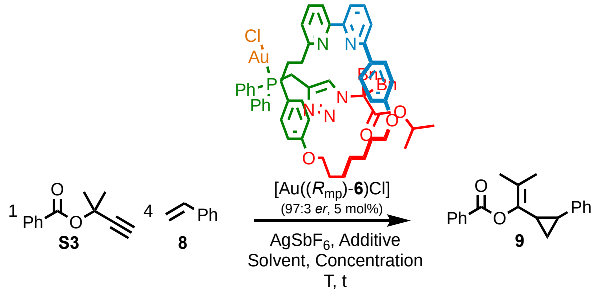

Entry[a]	Solvent	T (°C)	Additive	t (h)	Yield 9 (%) (*cis* : *trans*[b])	*e.r.*$_{cis}$[c]	*e.r.*$_{trans}$[c]
1	$CDCl_3$	25	-	1	-	-	-
2	$CDCl_3$	25	Cu^I	1	42% (95 : 5)	72 : 28	58 : 42
3[b]	$CDCl_3$	25	Cu^I	1	35% (95 : 5)	29 : 71	42 : 58
4	$MeNO_2$	25	Cu^I	1	69% (87 : 13)	53 : 47	65 : 35
5	CD_2Cl_2	25	Cu^I	1	13% (83 : 17)	64 : 36	66 : 34
6	CCl_4	25	Cu^I	1	46% (86 : 14)	71 : 29	58 : 42
7	PhMe	25	Cu^I	1	17% (85 : 15)	69 : 31	56 : 44
8[e]	$CDCl_3$	0	-	6	-	-	-
9[e]	$CDCl_3$	0	Cu^I	6	86% (94 : 6)	79 : 21	62 : 38
10[e]	$CDCl_3$	0	Zn^{II}	6	0% (-)	-	-
11[e]	$CDCl_3$	0	H^+	6	1% (-)	-	-
12[e]	$CDCl_3$	0	-	6	0% (-)	-	-
13	$CDCl_3$	-35	Cu^I	24	25% (96 : 4)	79 : 21	61 : 39
14[f]	$MeNO_2$	25	-	0.5	73% (>20 : 1)	16 : 84	-

[a][Au((***R***$_{mp}$)-**6**)(Cl)] with e.r. = 97 : 3 stereopurity was used for screening experiments unless otherwise stated. [b]Determined by ^1H NMR analysis of the crude reaction product using $C_2H_2Cl_4$ as an internal standard for yield determination. [c]Determined by HPLC. [d]Reaction conducted with [Au((*S*$_{mp}$)-**6**)(Cl)] of 3 : 97 ***er***. [e]Reaction conducted with [Au(**6**)(Cl)] with e.r. = 99 : 1 stereopurity. [a] Cu^I refers to $[Cu(MeCN)_4]PF_6$, Zn^{II} refers to $Zn(OTf)_2$, H^+ refers to HOTs.H_2O, Ag^I refers to $AgSbF_6$. [f]Reaction outcome reported by Toste and co-workers for (*R*)-DTBM-SEGPHOS®(AuCl)$_2$.[6]

Determination of the absolute stereochemistry of cyclopropanes 9

The absolute stereochemistry of cyclopropanes **9** was determined by comparing the HPLC chromatogram of the major *cis* diastereomer produced by [Au(**6**)(Cl)], with that produced using (*R*)-DTBM-SEGPHOS®$(AuCl)_2$ under conditions reported by Toste and co-workers, the stereochemical outcome of which is known.[6] Using this approach, the reactions mediated by [Au((R_{mp})-**6**)Cl] and [Au((S_{mp})-**6**)Cl] (**Table S3**, entries 2 and 3) were shown to yield (1*S*,2*R*)-**9** and (1*R*,2*S*)-**9** respectively as their major products.

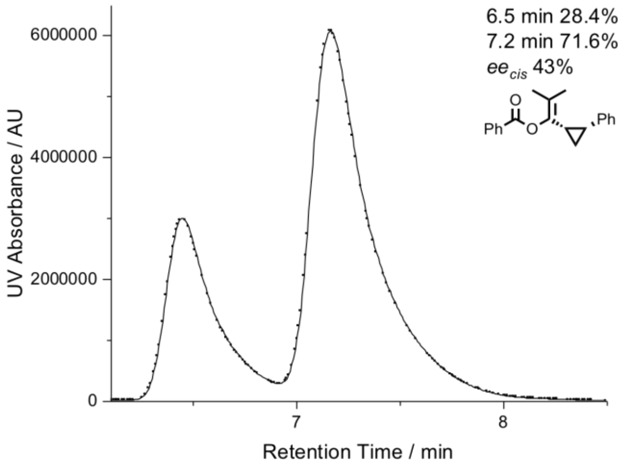

Figure S73: Chiral Stationary Phase HPLC (RegisPack, *n*-hexane-isopropanol 99 : 1, 303 K, load Et_2O, flowrate 0.75 $mLmin^{-1}$) of 72 : 28 *er cis*-**9** produced using [Au((R_{mp})-**6**)Cl] (97 : 3 *er*). Retention times (min): (1*R*,2*S*)-**9** 6.6, (1*S*,2*R*)-**9** 7.4.

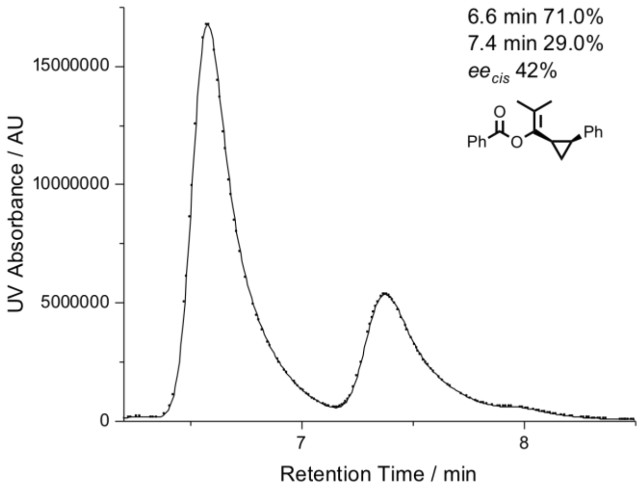

Figure S74: Chiral Stationary Phase HPLC (RegisPack, *n*-hexane-isopropanol 99 : 1, 303 K, load Et_2O, flowrate 0.75 $mLmin^{-1}$) of 29 : 71 *er cis*-**9** produced using [Au((S_{mp})-**6**)Cl] (3 : 97 *er*). Retention times (min): (1*R*,2*S*)-**9** 6.6, (1*S*,2*R*)-**9** 7.4.

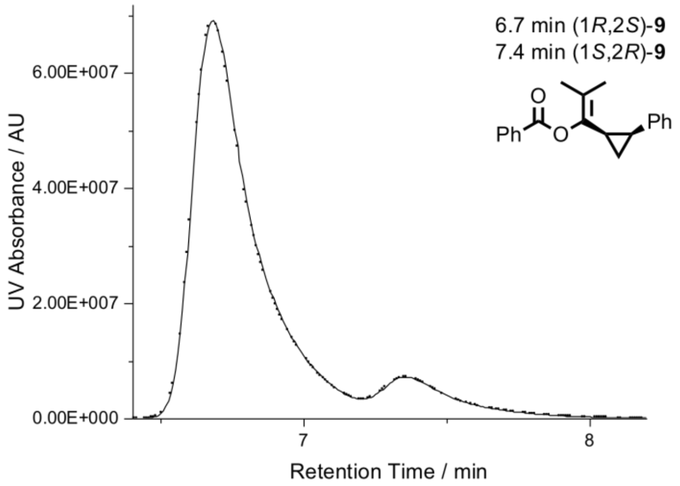

Figure S75: Chiral Stationary Phase HPLC (RegisPack, *n*-hexane-isopropanol 99 : 1, 303 K, load Et_2O, flowrate 0.75 mLmin^{-1}) of *cis*-**9** produced using (*R*)-DTBM-SEGPHOS®$(AuCl)_2$ in according to literature conditions to determine absolute stereochemistry.[6] Retention times (min): (1*R*,2*S*)-**9** 6.6, (1*S*,2*R*)-**9** 7.4.

Cyclopropanation General Procedures

Catalyst [Au(6)(Cl)]: A foil-covered, CEM MW vial was charged with $AgSbF_6$ (1.0 mg, 2.97 µmol, 0.05 eq.) and $[Cu(MeCN)_4]PF_6$ (1.1 mg, 3.0 µmol, 0.05 eq.), then purged with N_2. LAuCl was added as solution in $CDCl_3$ (0.059 M, 50 µL 3.0 µmol, 0.05 eq.) and the solution cooled to 0 ˚C. After 5 minutes, 2-methyl-3-butyn-2-yl ester (59.4 µmol, 1.0 eq.) and alkene (238 µmol, 4.0 eq.) were added in $CDCl_3$ (0.54 mL) (0.1 M with respect to the alkyne), and the solution stirred for 6 h at 0 ˚C . After 6 h, $C_2H_2Cl_4$ (20.0 µL, 0.189 mmol) was added, the solution was filtered through Celite®, and the yield and *dr* was determined by ^{1}H NMR. The reaction mixture was purified by column chromatography (SiO_2, petrol-Et_2O 0→10%) yielding isolated *cis* and *trans* cyclopropanes. Where possible, the enantiopurity of both diastereoisomers was determined by chiral stationary phase HPLC.

$[Au(PPh_3)(Cl)]$: Reactions with $[Au(PPh_3)(Cl)]$ were performed as per the general procedure at rt for 2 h in the absence of $[Cu(MeCN)_4]PF_6$ to provide racemic samples for HPLC method development.

(*R*)-DTBM-SEGPHOS®$(AuCl)_2$: Performed as per literature procedure (rt, $MeNO_2$, 2.5 mol% [Au(L)(Cl) (2.5 mol%), $AgSbF_6$ (5 mol%), benzoyl ester (1 eq., 0.05 M), alkene (4 eq.), $MeNO_2$, rt, 2 h).[6]

Cyclopropanes **9**[6]

**Catalyst**	**Yield / %**	***dr***	***er*$_{cis}$**	***er*$_{trans}$**
(Ph_3P)AuCl	96	89 : 11	1 : 1	1 : 1
[Au((R_{mp})-**6**)(Cl)]	86	94 : 6	78.5 : 21.5	62 : 38
(*R*)-DTBM-SEGPHOS®$(AuCl)_2$	73	>20 : 1	16 : 84	-

Table S4. Summary of reactions leading to cyclopropanes **9**.

cis-**9**

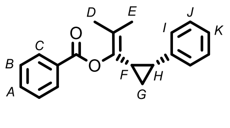

δ_H ($CDCl_3$, 400 MHz) 7.88 (2H, d, *J* = 7.5, ***H***$_C$), 7.58 (1H, tt, *J* = 7.5, 1.5, ***H***$_A$), 7.43 (2H, tt, *J* = 7.5, 1.0, ***H***$_B$), 7.26 (2H, dd, *J* = 7.0, 6.5, ***H***$_J$), 7.20 (1H, tt, *J* = 7.0, 1.5, ***H***$_K$), 7.12 (2H, dd, *J* = 7.5, 1.5, ***H***$_I$), 2.43-2.28 (2H, m, ***H***$_H$, ***H***$_F$), 1.65 (3H, s, ***H***$_F$), 1.49 (3H, s, ***H***$_D$), 1.29 (1H, ddd (td), *J* = 9.0, 5.5, ***H***$_G$), 1.11 (1H, dt (ddd), *J* = 5.5, 5.5, ***H***$_{G'}$).

δ_C ($CDCl_3$, 101 MHz) 164.8, 139.5, 138.7, 133.2, 130.0, 129.9, 128.5, 127.8, 127.8, 125.7, 123.6, 23.9, 21.6, 18.8, 17.8, 11.9.

HR-EI-MS m/z 292.1455 [$M^{+\cdot}$] (calc. m/z for $C_{20}H_{20}O_2$ 292.1458).

trans-**9**

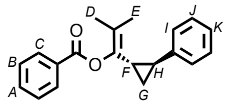

δ_H ($CDCl_3$, 400 MHz) 8.13 (2H, dd, *J* = 8.1, 1.4, ***H***$_C$), 7.61 (1H, tt, *J* = 7.4, 2.7, ***H***$_A$), 7.49 (2H, app. t, *J* = 7.6, ***H***$_B$), 7.26 (2H, br. t, *J* = 7.5, ***H***$_J$), 7.16 (1H, tt, *J* = 7.4, 1.3, ***H***$_K$), 7.10 (2H, dd, *J* = 7.7, 1.5, ***H***$_I$), 7.28-7.18 (2H, m, ***H***$_F$, ***H***$_H$), 1.86 (3H, s, ***H***$_E$), 1.63 (3H, s, ***H***$_D$), 1.21 (1H, ddd, *J* = 8.7, 6.2, 4.9, ***H***$_G$), 1.16 (1H, ddd, *J* = 8.9, 6.0, 5.1, ***H***$_{G'}$).

δ_C ($CDCl_3$, 101 MHz) 164.9, 142.2, 141.0, 133.4, 130.1, 129,8, 128.7, 128.5, 126.1, 125.9, 121.1, 23.8, 23.5, 19.0, 18.3, 14.8.

HR-EI-MS m/z 292.1455 [$M^{+\cdot}$] (calc. m/z for $C_{20}H_{20}O_2$ 292.1458).

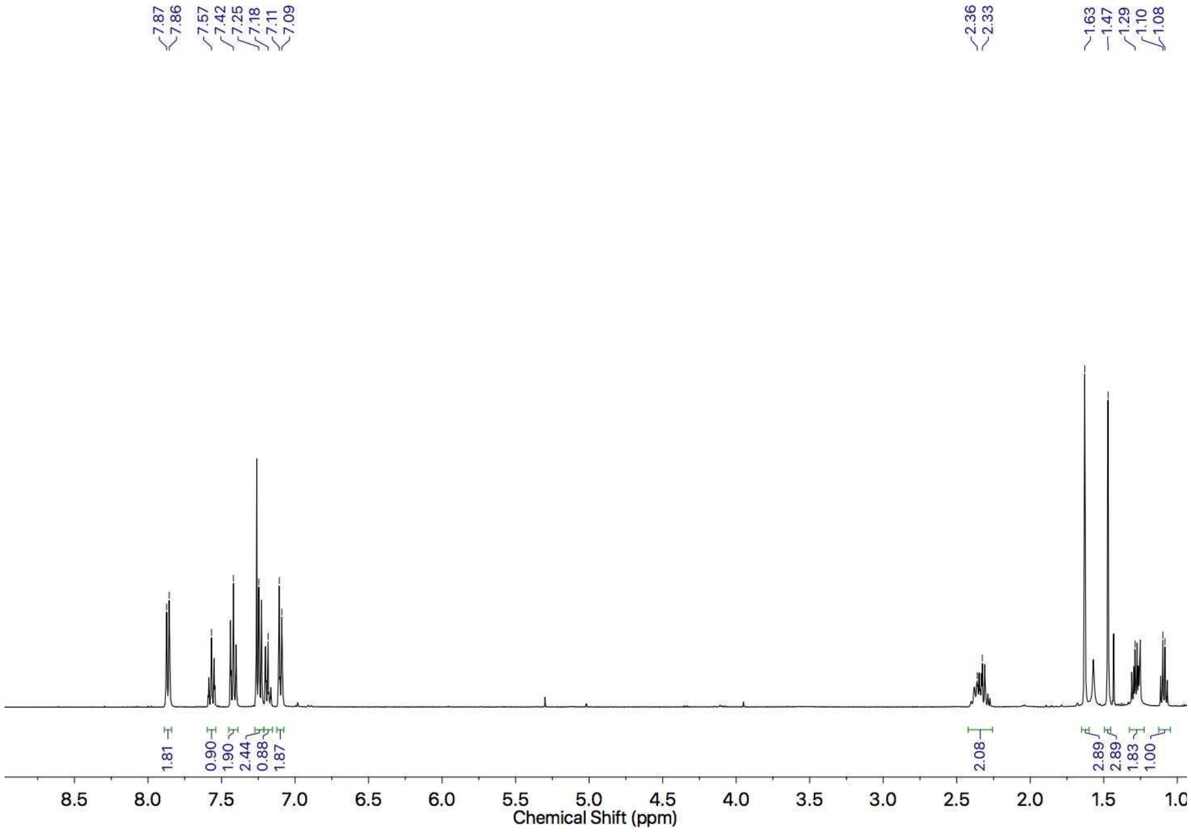

Figure S76: ^{1}H NMR (400 MHz, $CDCl_3$) of *cis*-**9**.

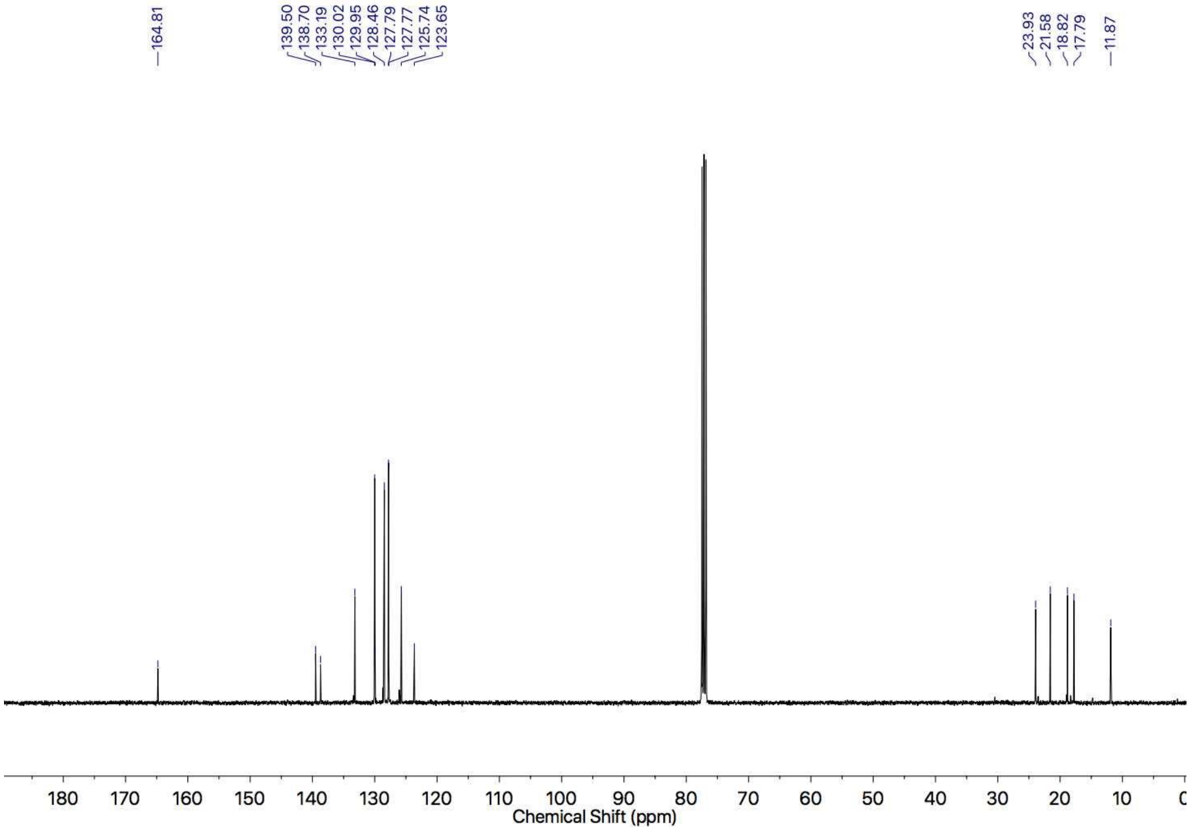

Figure S77: ^{13}C NMR (101 MHz, $CDCl_3$) of *cis*-**9**.

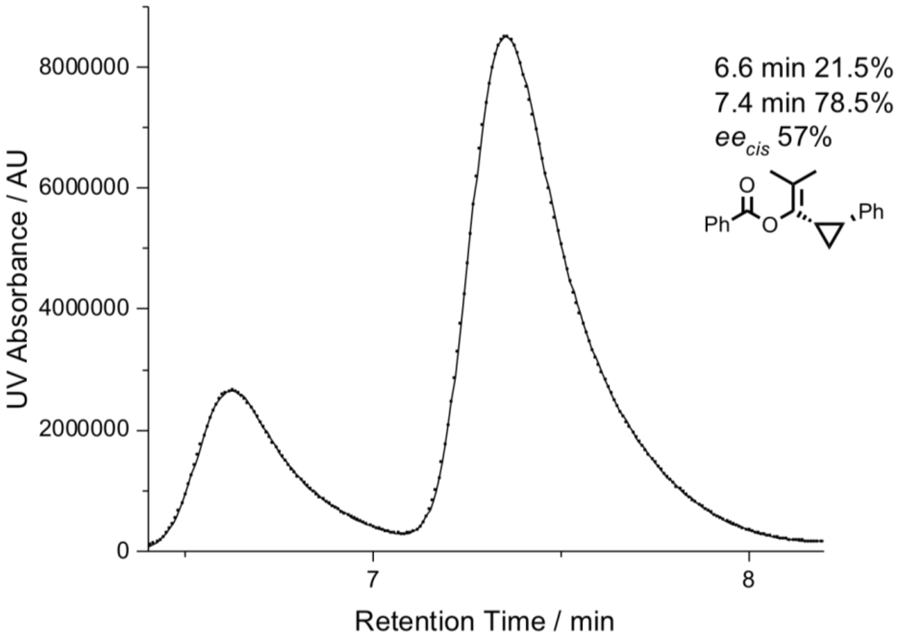

Figure S78: Chiral Stationary Phase HPLC (RegisPack, *n*-hexane-isopropanol 99 : 1, 303 K, load Et_2O, flowrate 0.75 $mLmin^{-1}$) of 78.5 : 21.5 *er cis*-**9**. Retention times (min): (1*R*,2*S*)-**9** 6.6, (1*S*,2*R*)-**9** 7.4.

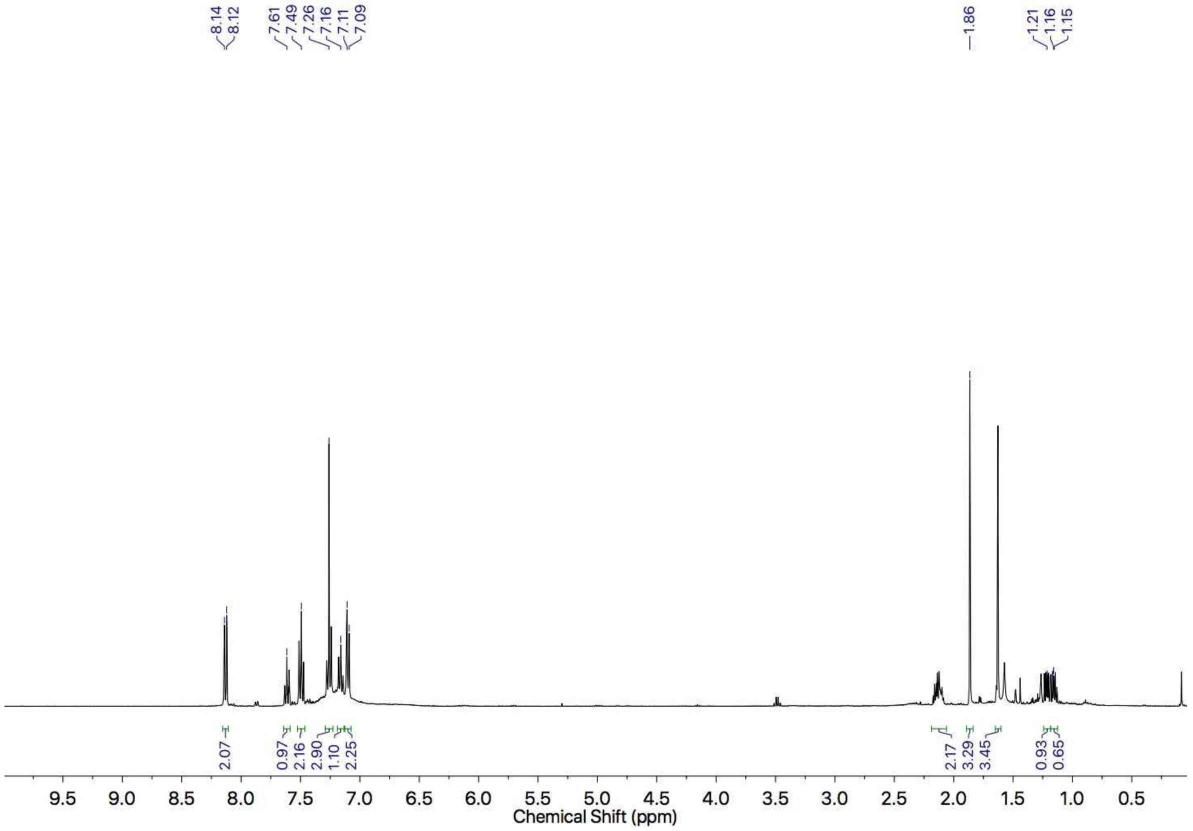

Figure S79: ^1H NMR (400 MHz, $CDCl_3$) of *trans*-**9**.

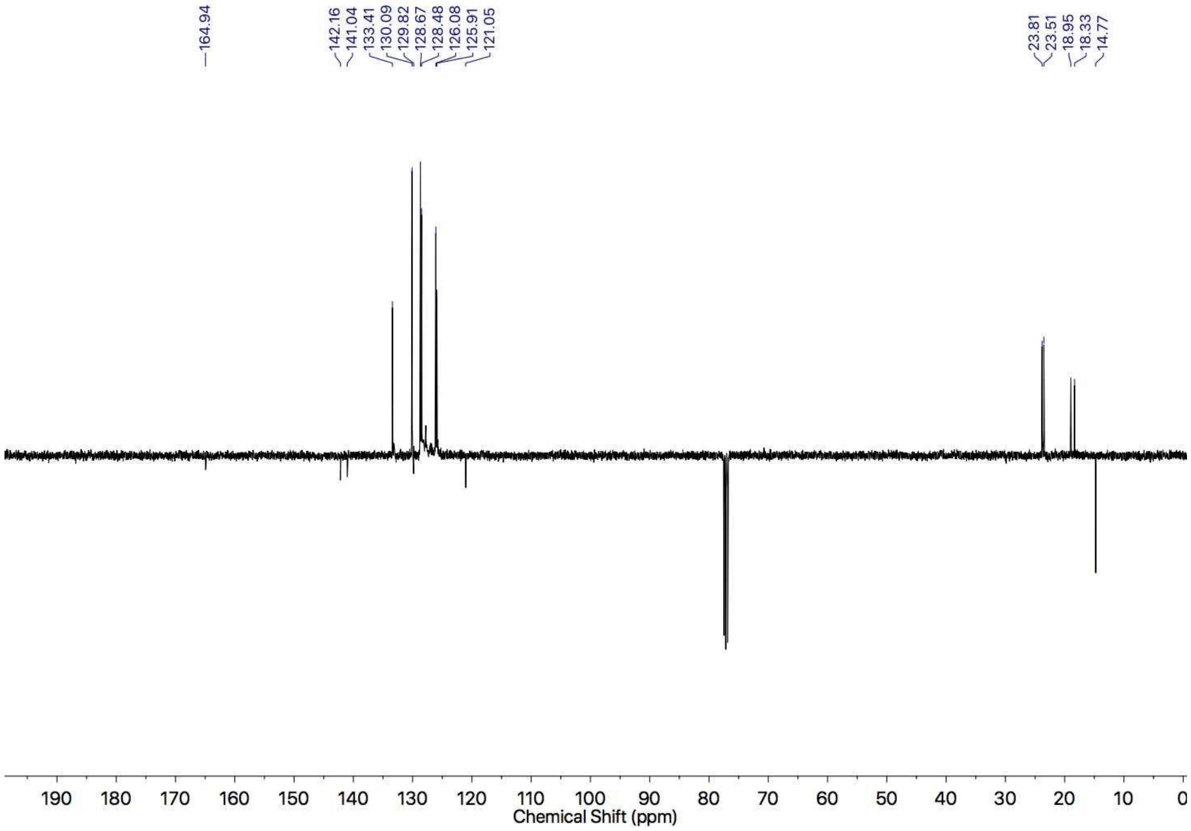

Figure S80: JMOD NMR (101 MHz, $CDCl_3$) of *trans*-**9**.

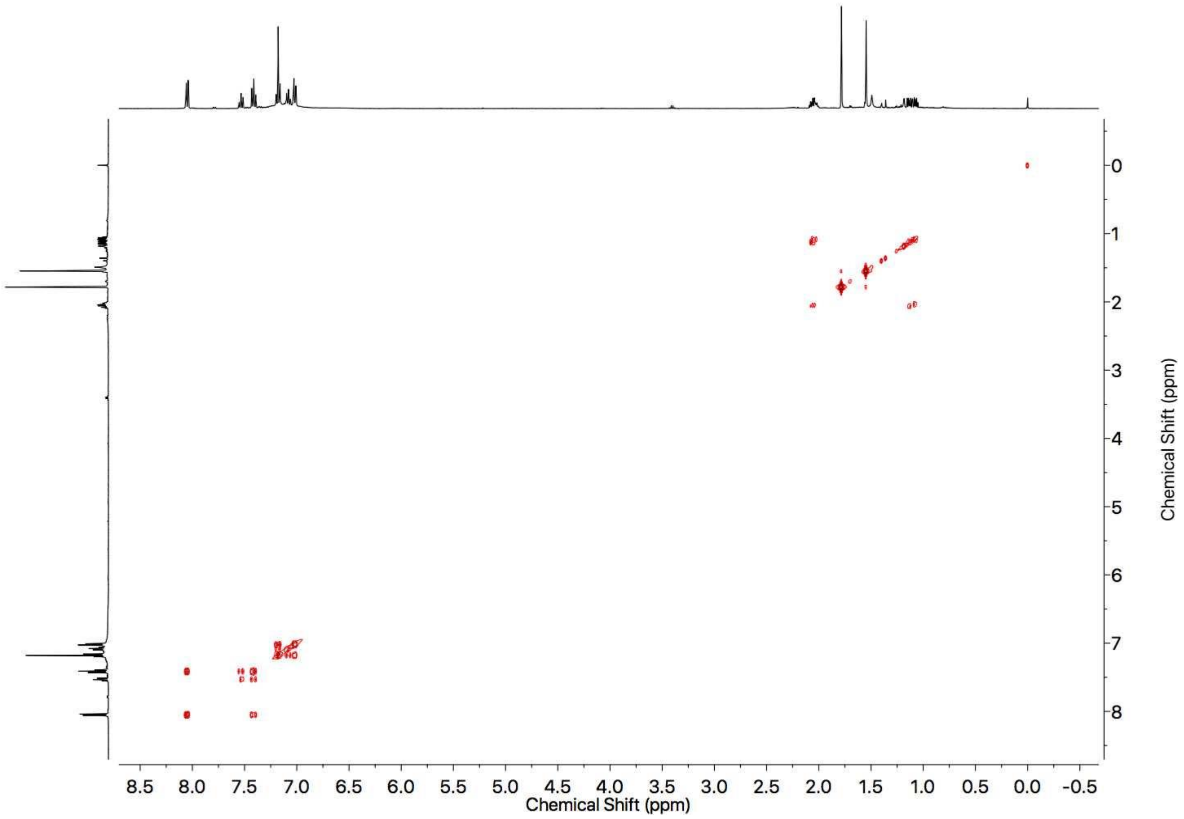

Figure S81: COSY NMR ($CDCl_3$) of *trans*-**9**.

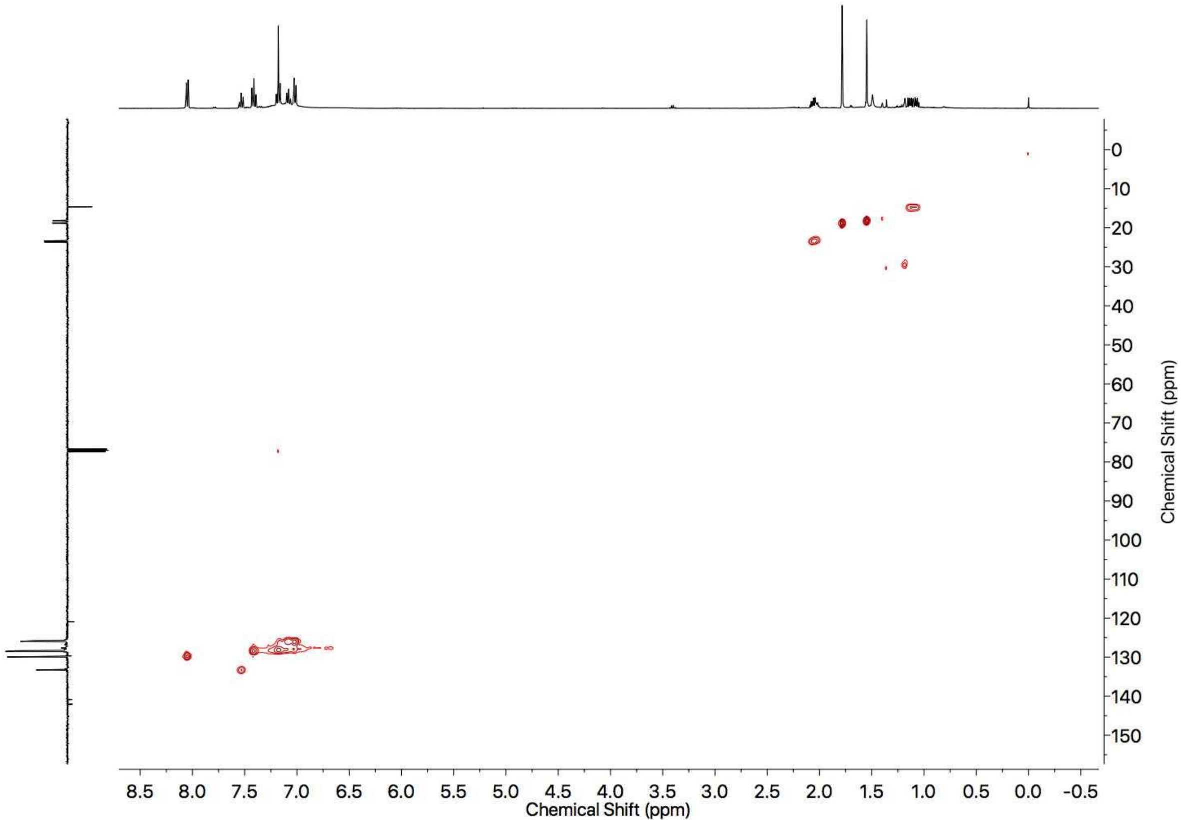

Figure S82: HSQC NMR ($CDCl_3$) of *trans*-**9**.

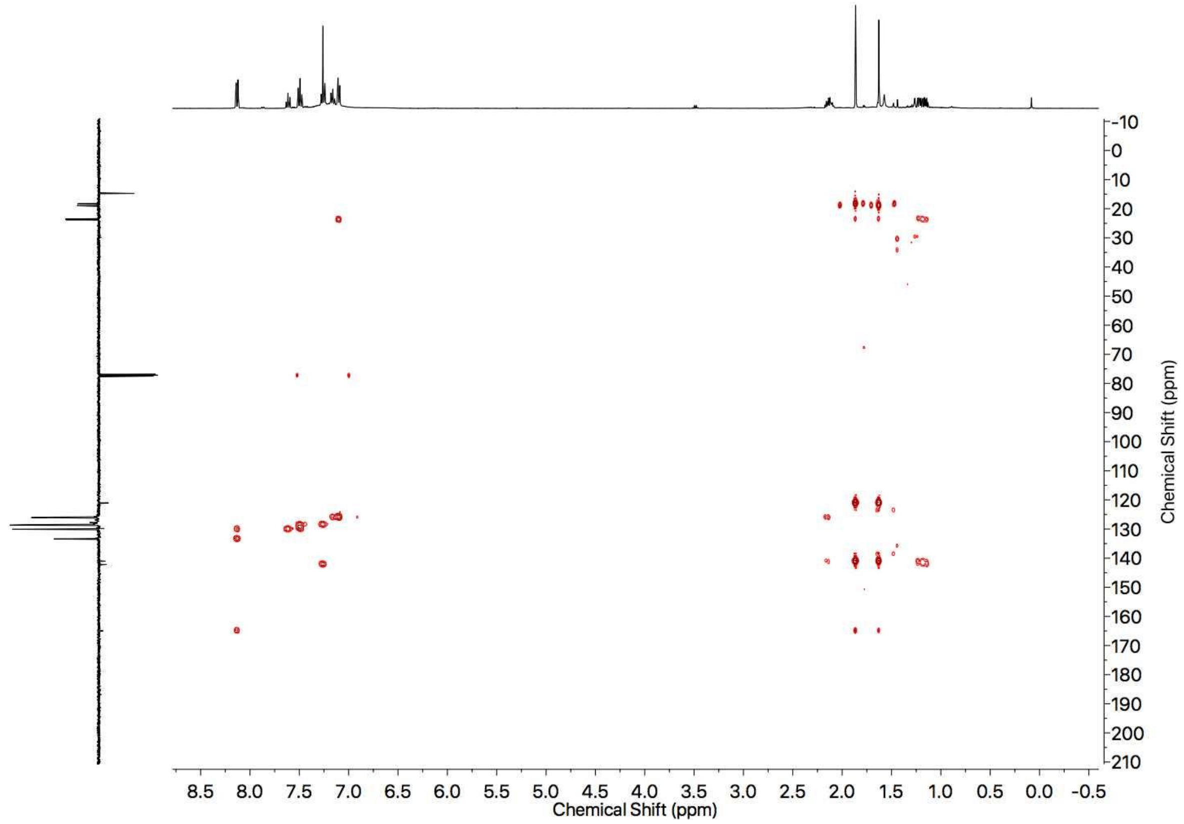

Figure S83: HMBC NMR ($CDCl_3$) of *trans*-**9**.

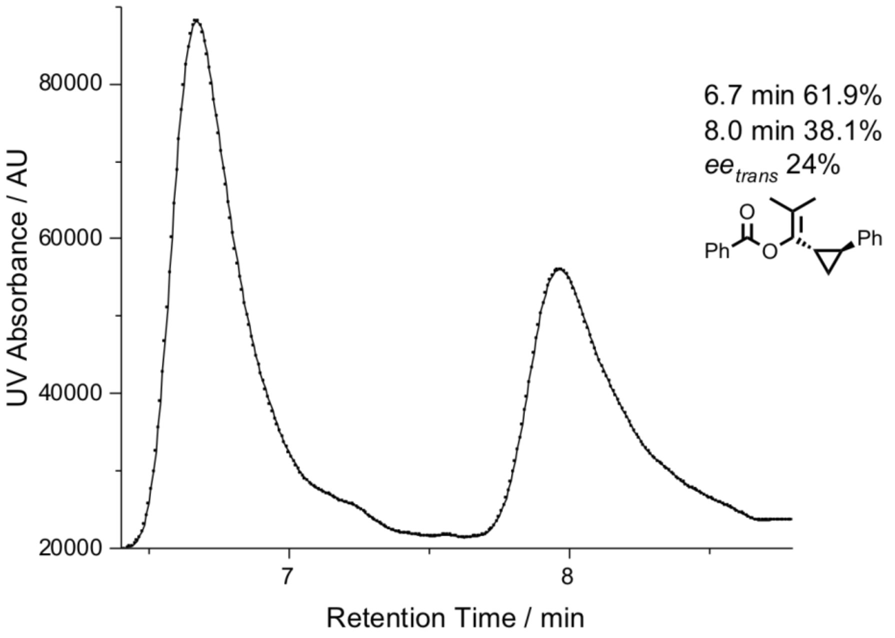

Figure S84: Chiral Stationary Phase HPLC (RegisPack, *n*-hexane-isopropanol 99 : 1, 303 K, load Et_2O, flowrate 0.75 $mLmin^{-1}$) of 62 : 38 *er trans*-**9**. Retention times (min): 6.7, 8.0.

Cyclopropane **10**

**Catalyst**	**Yield / %**	***dr***	***er*cis**
$(Ph_3P)AuCl$[a]	86	4.4 : 1	1 : 1
[Au((*R*mp)-**6**)(Cl)][b]	53	92 : 8	75 : 25

Table S5. Summary of reactions leading to cyclopropanes **10**.

cis-**10**

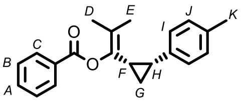

δ_H ($CDCl_3$, 400 MHz) 7.88 (2H, dd, *J* = 7.9, 1.1, ***H***C), 7.57 (1H, tt, *J* = 7.4, 1.3, ***H***A), 7.42 (2H, tt, *J* = 7.8, 1.5, ***H***B), 7.06 (2H, d, *J* = 8.1, ***H***J), 7.00 (2H, d, *J* = 8.1, ***H***I), 2.35 (3H, s, ***H***K), 2.34-2.25 (2H, m, ***H***F, ***H***G), 1.66 (3H, s, ***H***E), 1.49 (3H, s, ***H***D), 1.26 (1H, td, *J* = 8.9, 5.4, ***H***H), 1.07 (1H, dt, *J* = 6.7, 5.9, ***H***H').

δ_C ($CDCl_3$, 101 MHz) 164.8, 138.9, 136.3, 135.1, 133.1, 130.0, 130.0, 128.5, 128.4, 127.7, 123.4, 23.6, 21.3, 21.2, 18.8, 17.8, 11.7.

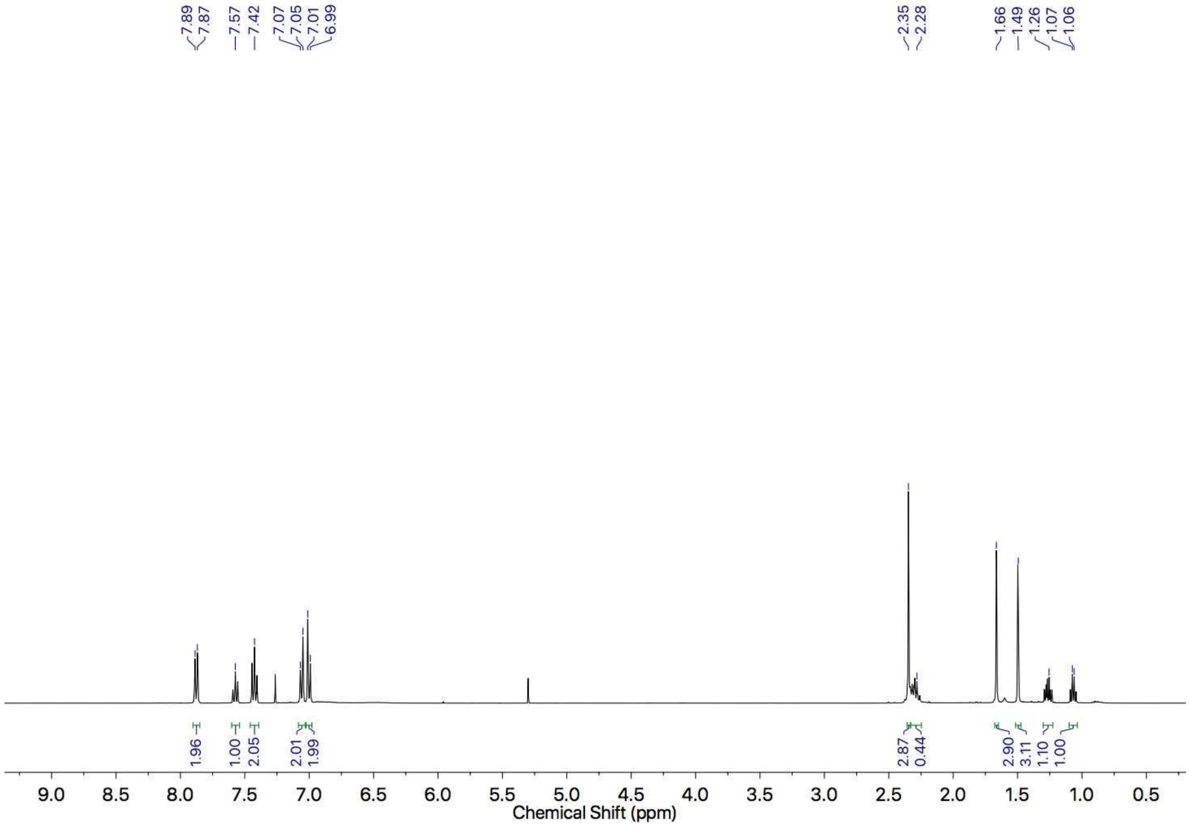

Figure S85: ^1H NMR (400 MHz, $CDCl_3$) of *cis*-**10**.

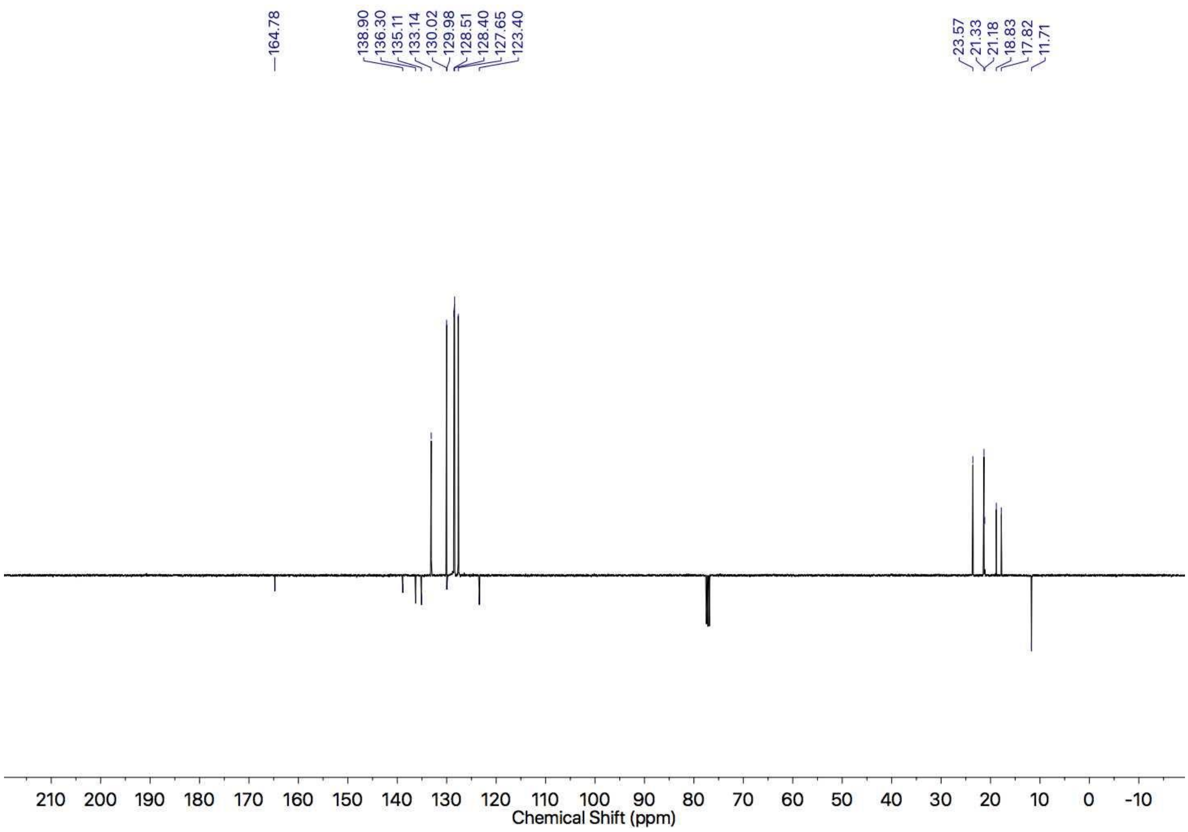

Figure S86: JMOD NMR (101 MHz, $CDCl_3$) of *cis*-**10**.

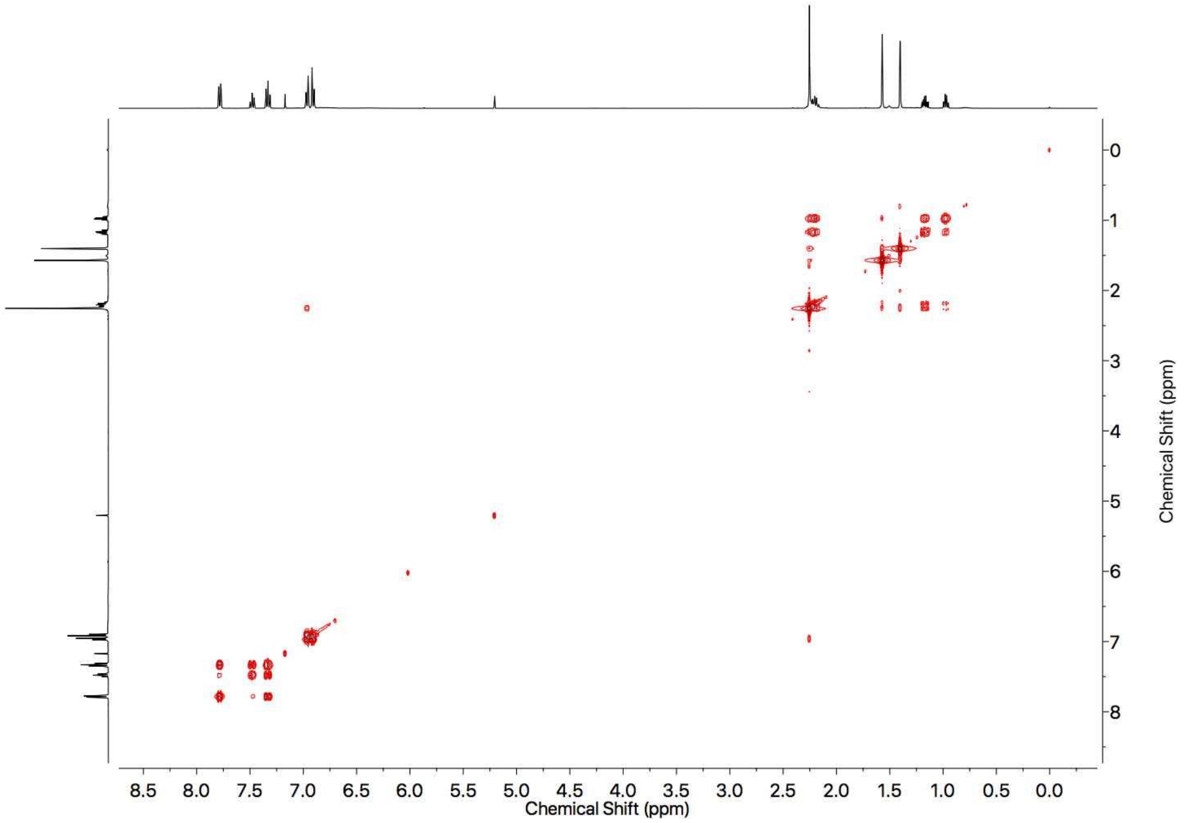

Figure S87: COSY NMR ($CDCl_3$) of *cis*-**10**.

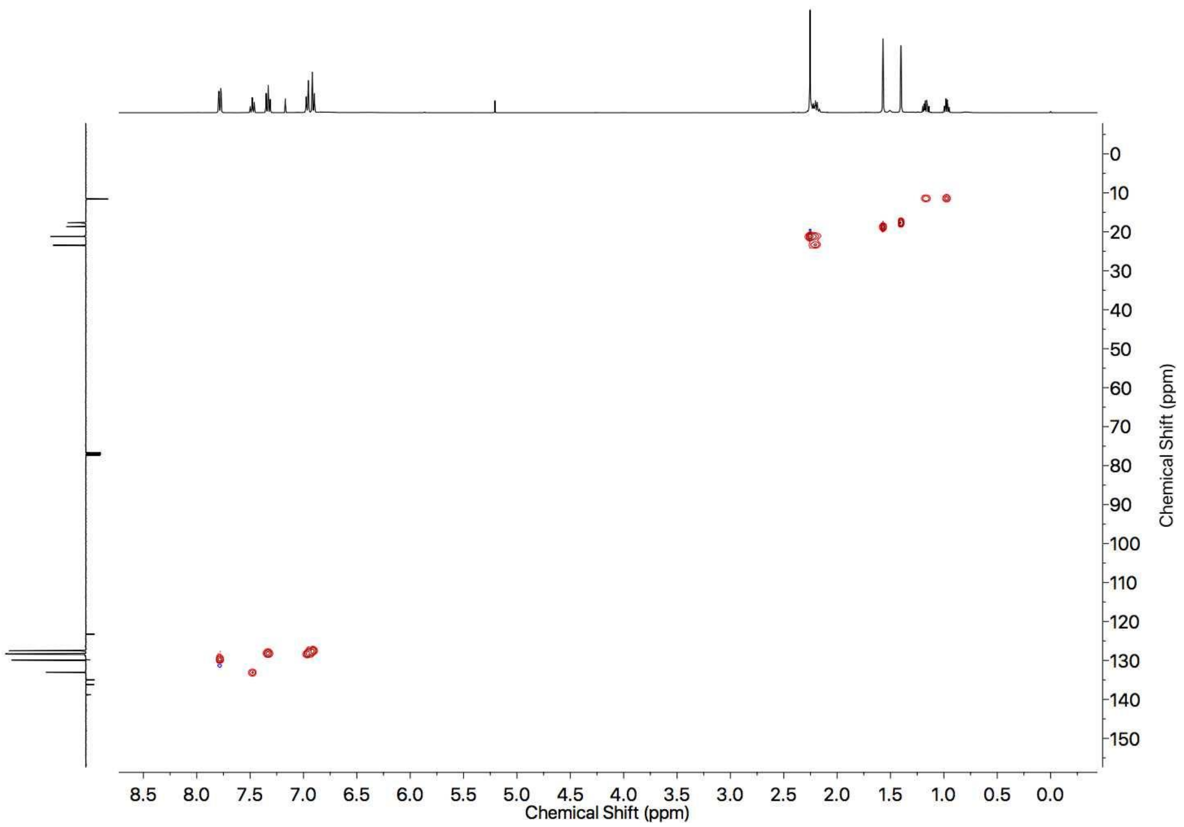

Figure S88: HSQC NMR ($CDCl_3$) of *cis*-**10**.

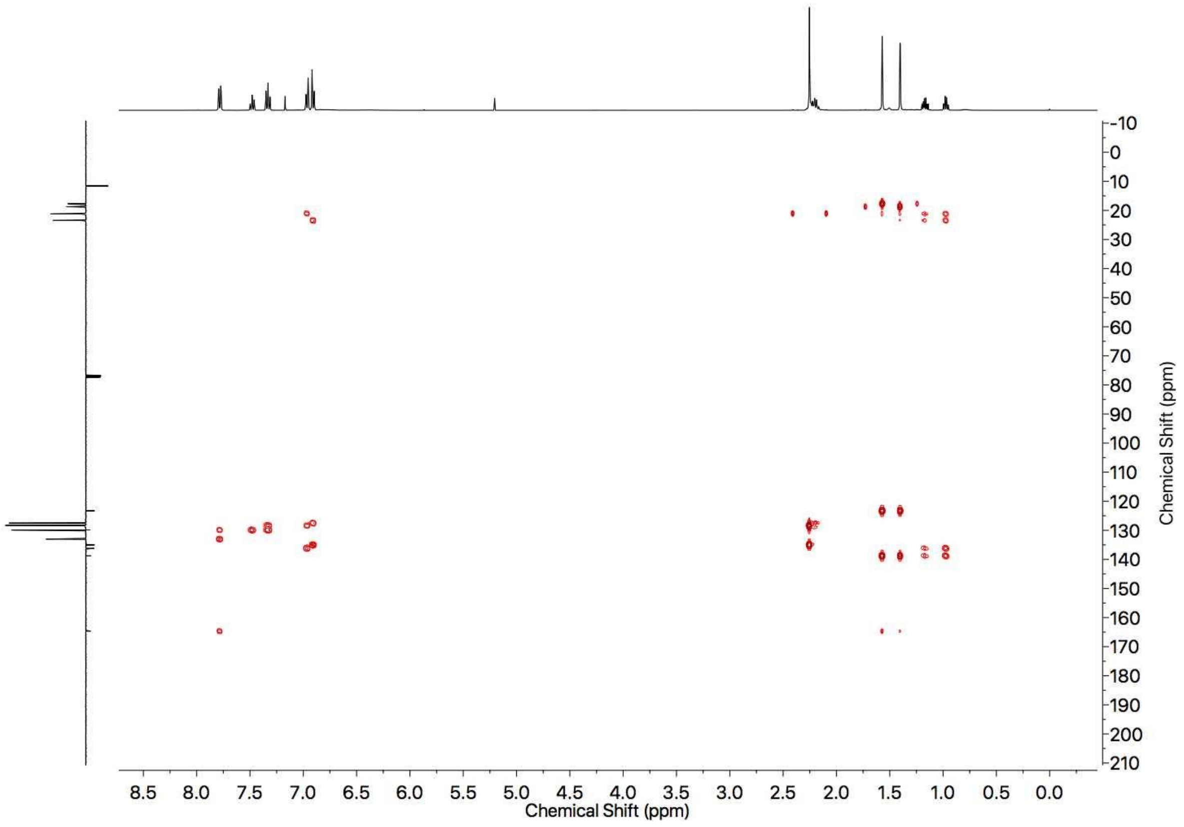

Figure S89: HMBC NMR ($CDCl_3$) of *cis*-**10**.

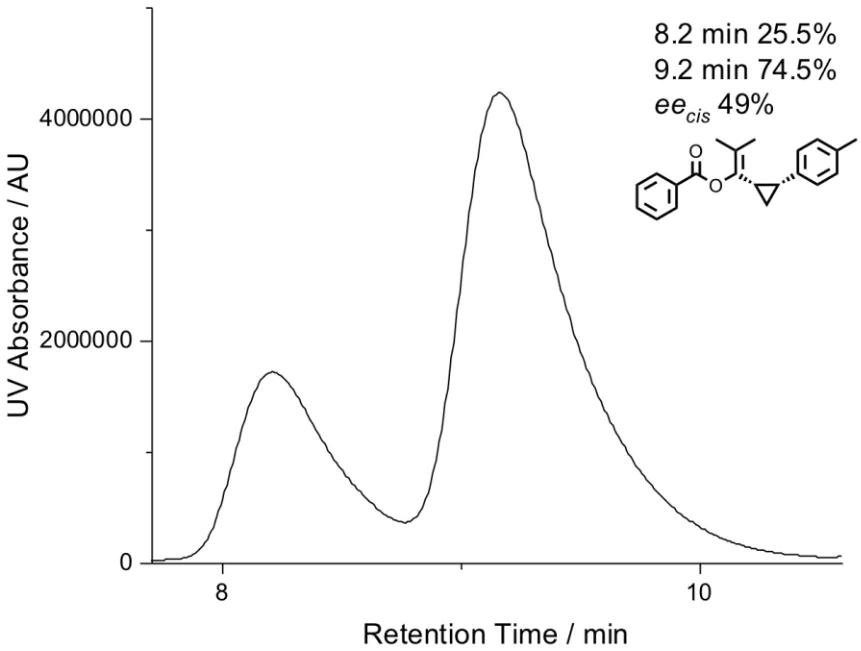

Figure S90: Chiral Stationary Phase HPLC (RegisPack, *n*-hexane-isopropanol 99.5 : 0.5, 303 K, load petrol, flowrate 0.75 mLmin^{-1}) of 25 : 75 *er cis*-**10**. Retention times (min): 8.2, 9.2. The absolute stereochemistry of the products was not determined. The (1*S*,2*R*)-**10** isomer is shown for illustrative purposes only.

Cyclopropane **11**

**Catalyst**	**Yield / %**	***dr***	***er*cis**
$(Ph_3P)AuCl$[a]	66	4.5 : 1	1 : 1
[Au((*R*mp)-**6**)(Cl)][b]	25	25 : 1	73 : 27

Table S6. Summary of reactions leading to cyclopropanes **11**.

cis-**11**

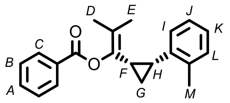

δ_H ($CDCl_3$, 400 MHz) 7.68 (2H, dd, *J* = 8.3, 1.2, ***H***C), 7.54 (1H, tt, *J* = 7.4, 1.2, ***H***A), 7.37 (2H, dd, *J* = 7.9, 7.6, ***H***B), 7.14-7.11 (2H, m, ***H***L, ***H***K), 7.07 (1H, dt, *J* = 8.9, 3.9, ***H***J), 6.96 (2H, d, *J* = 7.4, ***H***I), 2.43 (1H, td, *J* = 8.9, 5.7, ***H***F), 2.34 (3H, s, ***H***M), 2.28 (1H, td, *J* = 8.6, 6.8, ***H***G), 1.83 (3H, s, ***H***E), 1.44 (3H, s, ***H***D), 1.29 (1H, td, *J* = 8.9, 5.4, ***H***H), 1.18 (1H, dt, *J* = 6.2, 5.9, ***H***H′).

δ_C ($CDCl_3$, 101 MHz) 164.4, 139.4, 138.3, 137.2, 133.1, 130.2, 129.7, 129.6, 128.3, 127.1, 125.9, 125.4, 122.1, 21.2, 20.2, 19.8, 19.0, 18.2, 10.3.

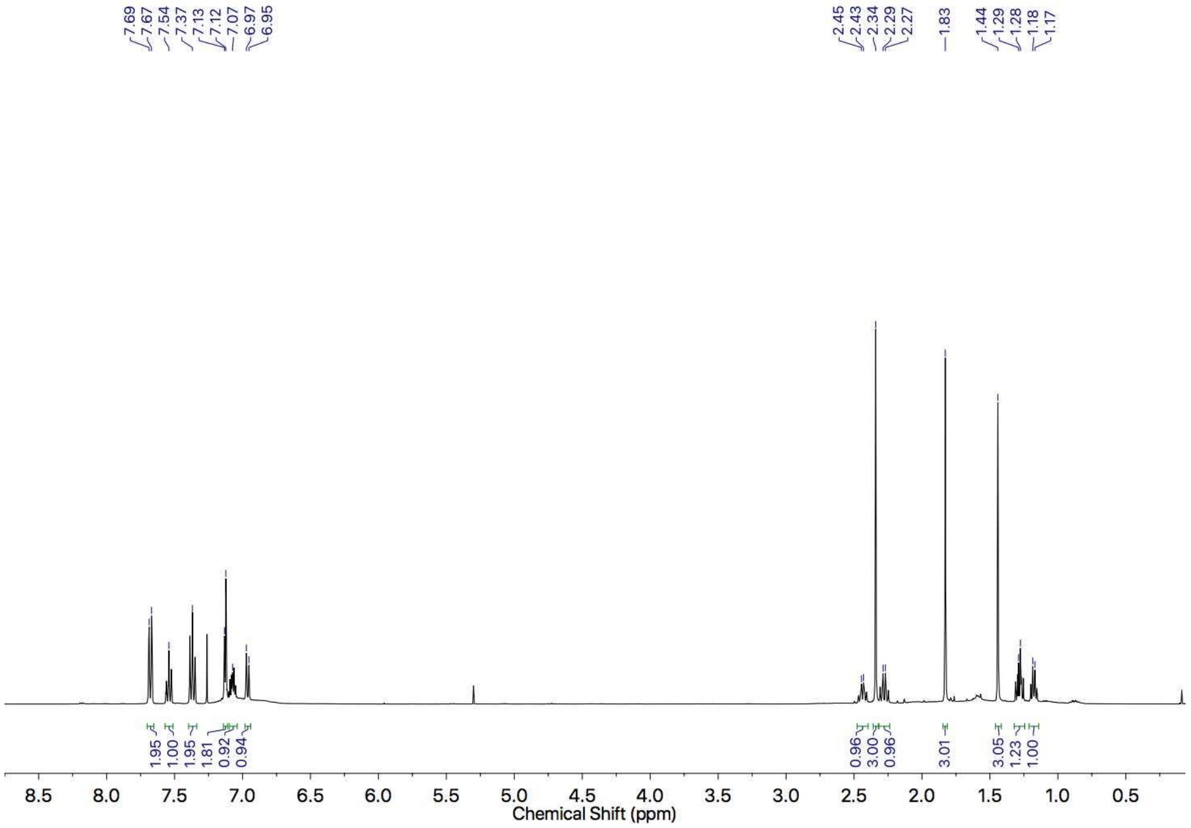

Figure S91: ^1H NMR (400 MHz, $CDCl_3$) of *cis*-**11**.

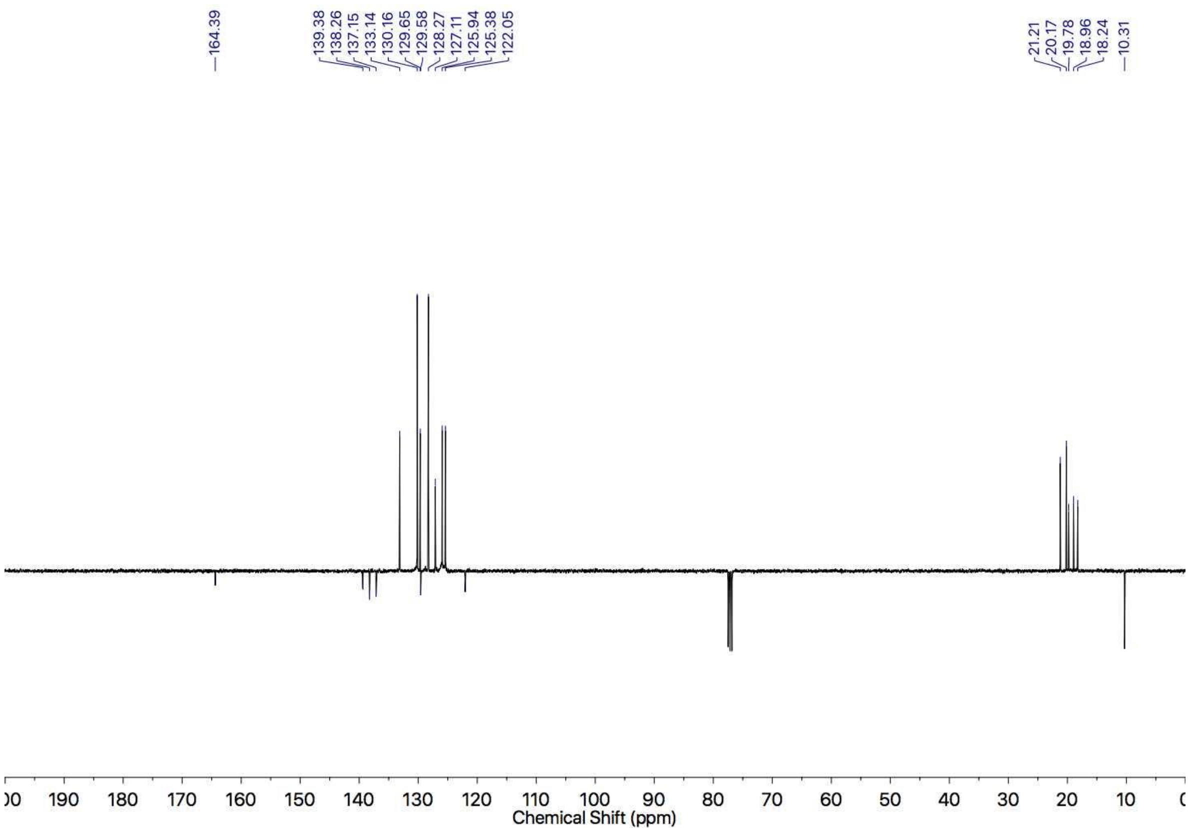

Figure S92: JMOD NMR (101 MHz, $CDCl_3$) of *cis*-**11**.

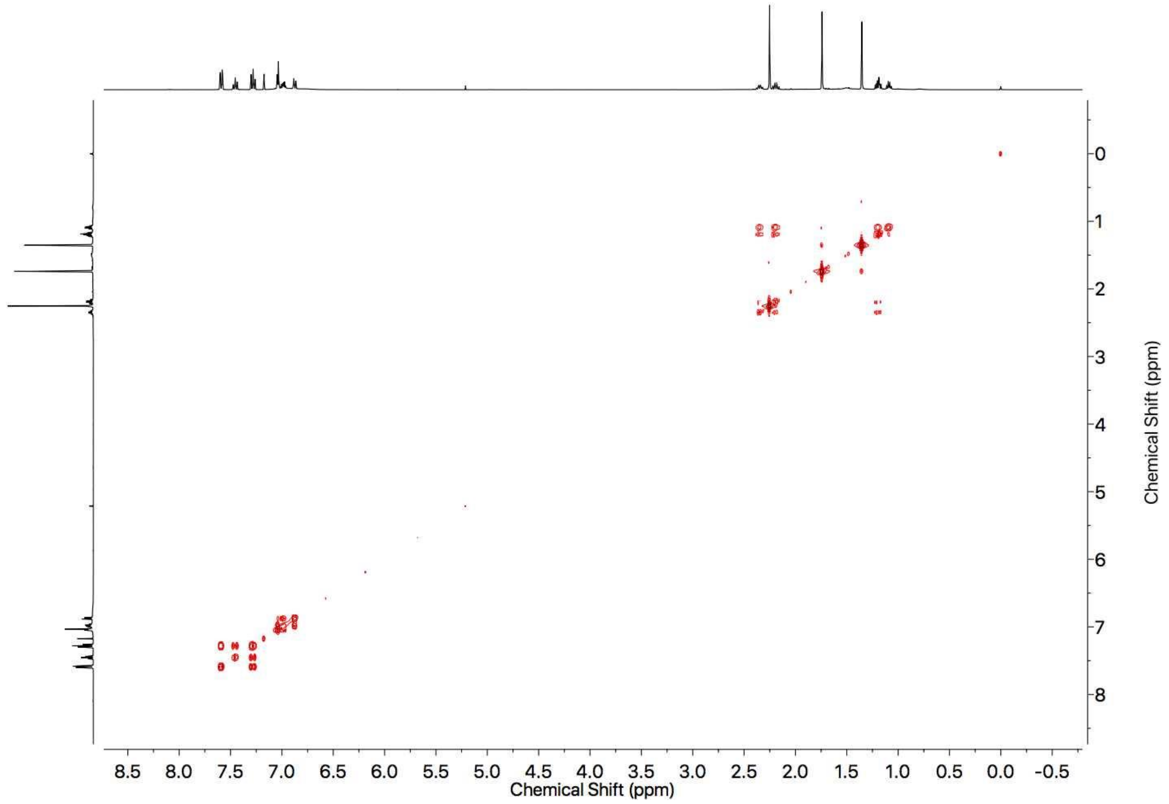

Figure S93: COSY NMR ($CDCl_3$) of *cis*-**11**.

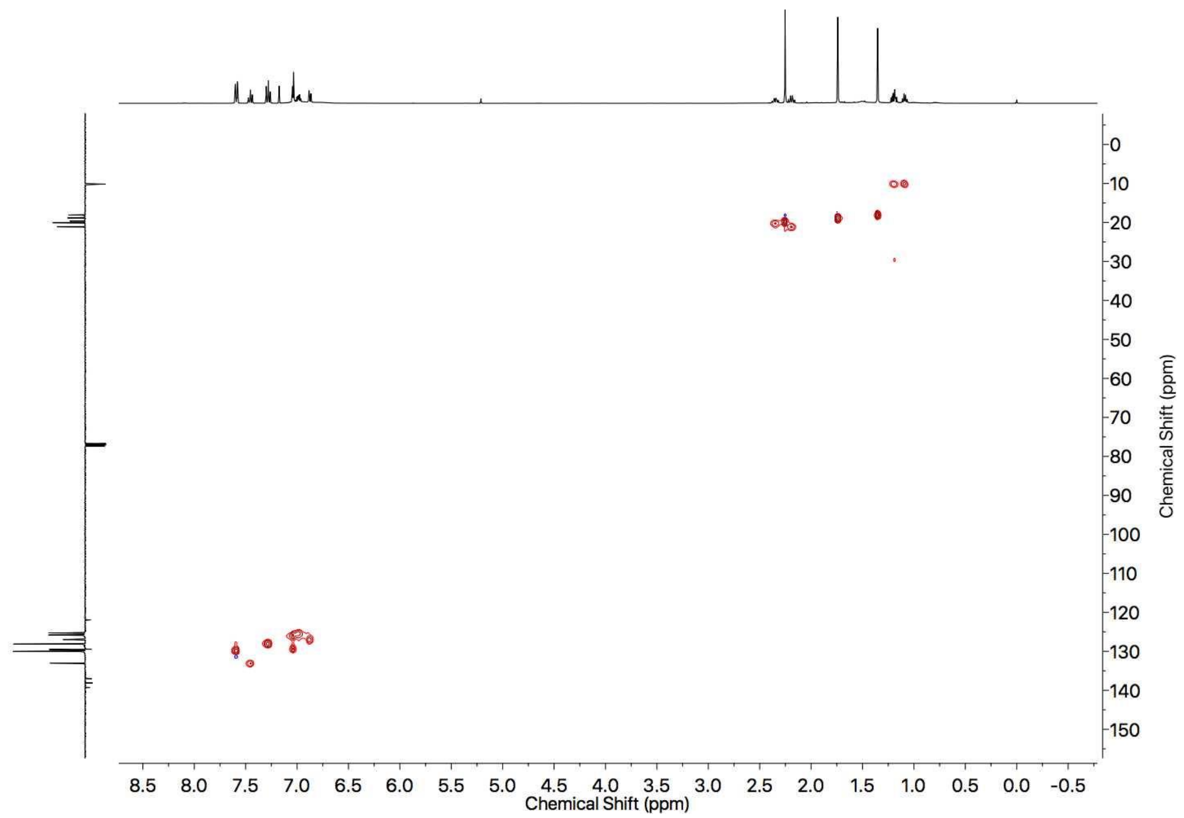

Figure S94: HSQC NMR ($CDCl_3$) of *cis*-**11**.

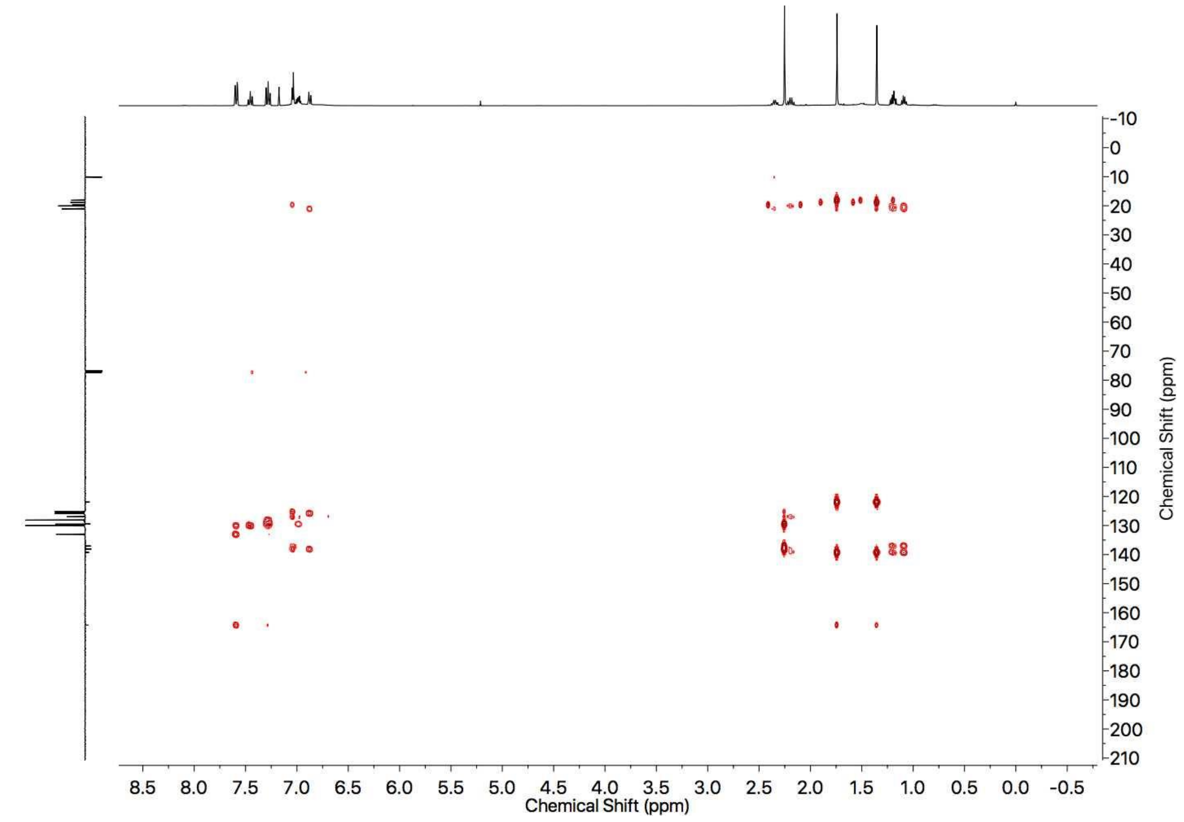

Figure S95: HMBC NMR ($CDCl_3$) of *cis*-**11**.

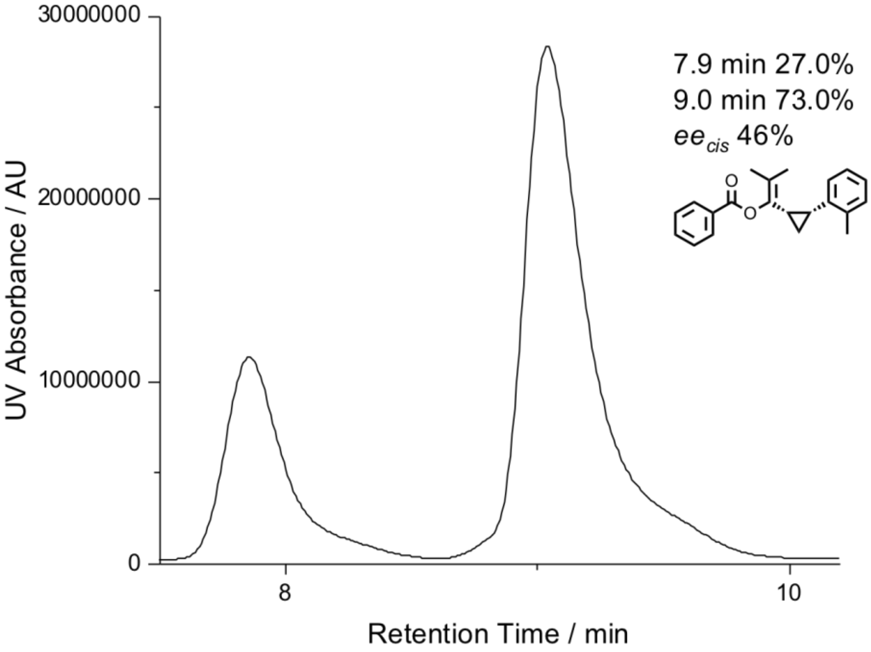

Figure S96: Chiral Stationary Phase HPLC (RegisCell, *n*-hexane-isopropanol 99.5 : 0.5, 303 K, load petrol, flowrate 0.75 mLmin^{-1}) of 27 : 73 *er cis*-**11**. Retention times (min): 7.9, 9.0. The absolute stereochemistry of the products was not determined. The (1*S*,2*R*)-**11** isomer is shown for illustrative purposes only.

Cyclopropanes **12**

**Catalyst**	**Yield / %**	***dr***	***er*cis**	***er*trans**
$(Ph_3P)AuCl$[a]	77	56 : 44	1 : 1	1 : 1
$[Au((R_{mp})$-**6**$)(Cl)]$[b]	73	82 : 18	71 : 29	53 : 47

Table S7. Summary of reactions leading to cyclopropanes **12**.

cis-**12** and *trans*-**12** were not fully separated

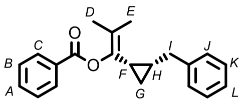

δ_H ($CDCl_3$, 400 MHz) 8.04 (2H, dd, J = 8.5, 1.4, H_C), 7.63-7.55 (1H, m, H_A), 7.50-7.42 (1H, m, H_B), 7.31-7.12 (5H, m, H_J, H_K, H_L), 2.71 (1H, dd, J = 14.8, 6.5, H_I), 2.59 (1H, dd, J = 14.7, 7.3, $H_{I'}$), 1.82 (3H, s, H_E), 1.76-1.70 (1H, m, H_F), 1.58 (3H, s, H_D), 1.31-1.25 (1H, m, H_G), 0.84 (1H, dt, J = 8.5, 5.1, H_H), 0.67 (1H, dt, J = 8.5, 5.1, $H_{H'}$).

δ_C ($CDCl_3$, 101 MHz) 164.8, 141.6, 141.2, 133.2, 130.0, 129.9, 128.5, 128.4, 126.1, 122.7, 39.6, 19.8, 18.9, 18.8, 18.7, 18.3, 12.5.

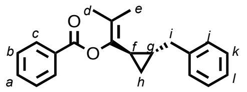

δ_H ($CDCl_3$, 400 MHz) 8.11 (2H, dd, J = 8.4, 1.31, H_c), 7.63-7.55 (1H, m, H_a), 7.50-7.42 (1H, m, H_b), 7.31-7.12 (5H, m, H_j, H_k, H_l), 2.85 (1H, dd, J = 14.8, 6.1, H_i), 2.50 (1H, dd, J = 15.0, 8.6, $H_{i'}$), 2.07-1.99 (1H, m, H_f), 1.79 (3H, s, H_e), 1.66 (3H, s, H_d), 1.40-1.31 (1H, m, H_g), 0.96 (1H, ddd, J = 13.5, 8.8, 5.0, H_h), 0.47 (1H, q, J = 5.6, $H_{h'}$).

δ_C ($CDCl_3$, 101 MHz) 164.8, 142.1, 140.2, 133.3, 130.0, 129.9, 128.6, 128.5, 128.4, 125.9, 120.1, 35.9, 19.5, 18.7, 18.1, 17.4, 11.5. (C_h, C_j, C_k, C_l in 8 signal in the range 128.6-125.9).

HR-EI-MS m/z 306.1607 $[M^{+\cdot}]$ (calc. m/z for $C_{21}H_{22}O_2$ 306.1620).

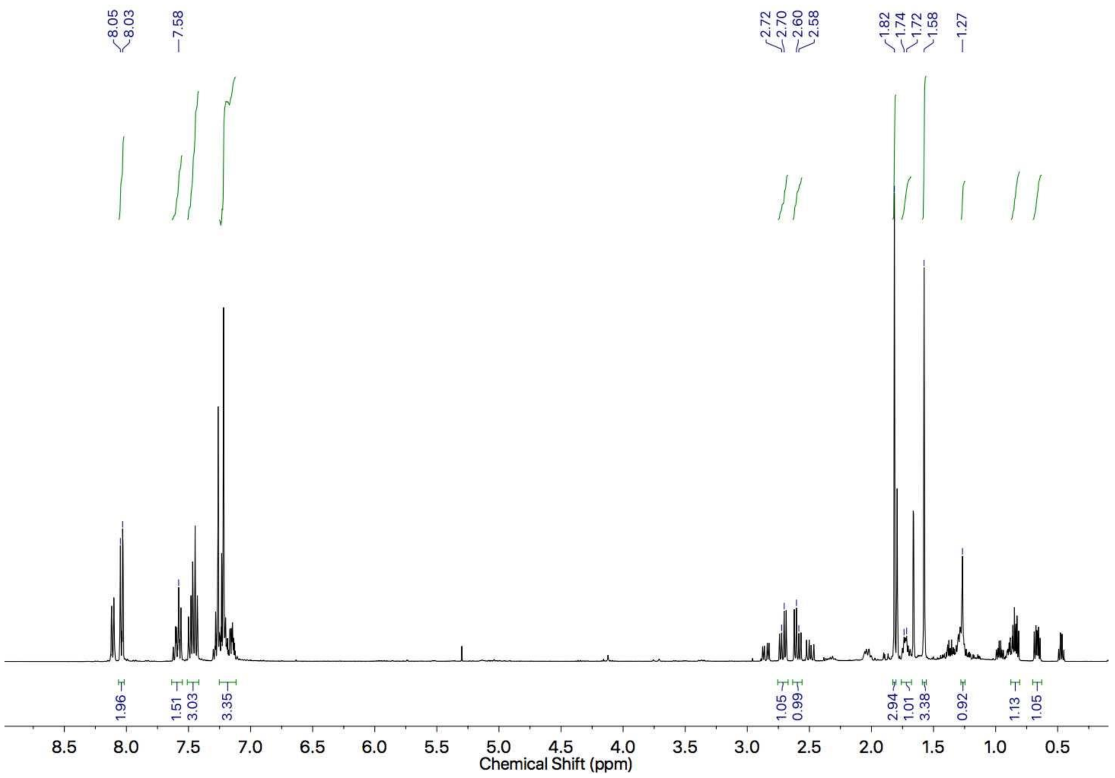

Figure S97: ^{1}H NMR (400 MHz, $CDCl_3$) of the mixture of *cis*-**12** and *trans*-**12** focussing on the major *cis* isomer.

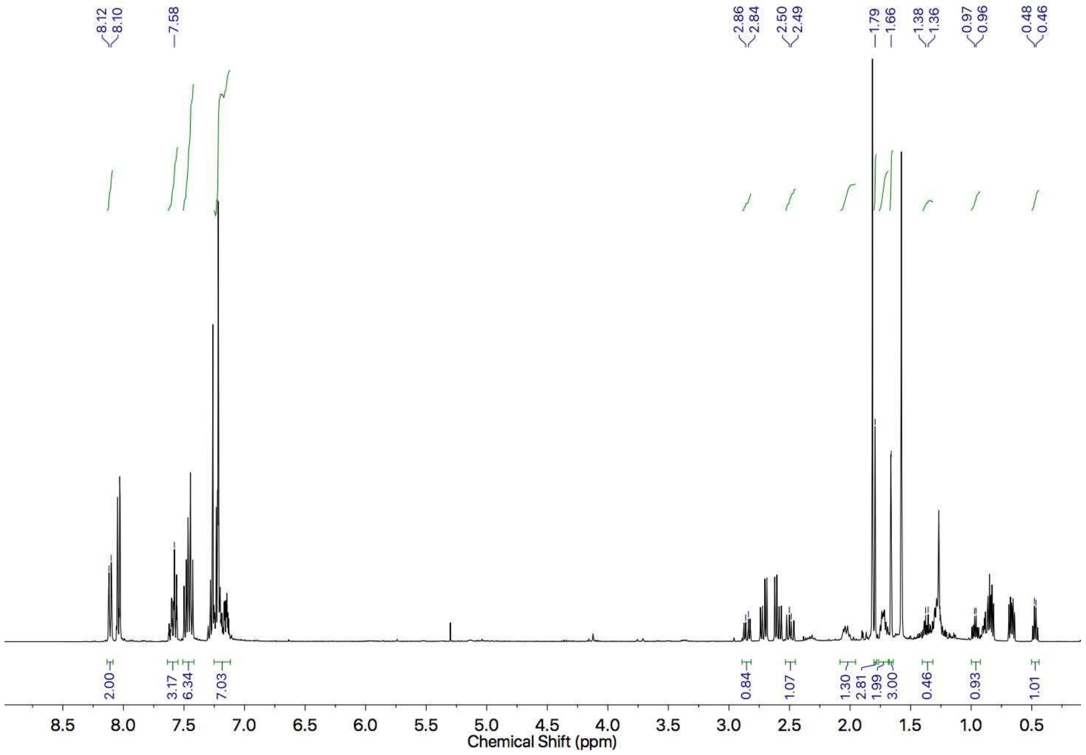

Figure S98: ^{1}H NMR (400 MHz, $CDCl_3$) of the mixture of *cis*-**12** and *trans*-**12** focussing on the minor *trans* isomer.

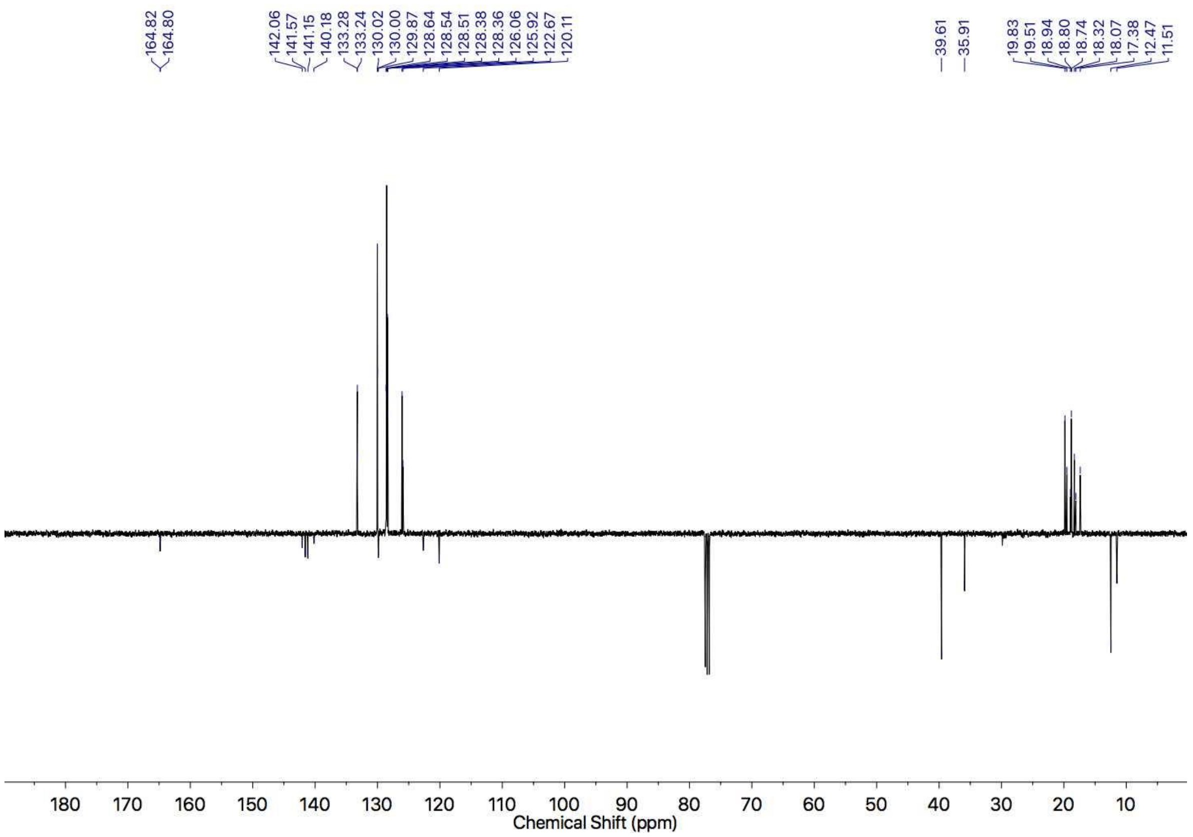

Figure S99: JMOD NMR (101 MHz, $CDCl_3$) of cyclopropanes **12**.

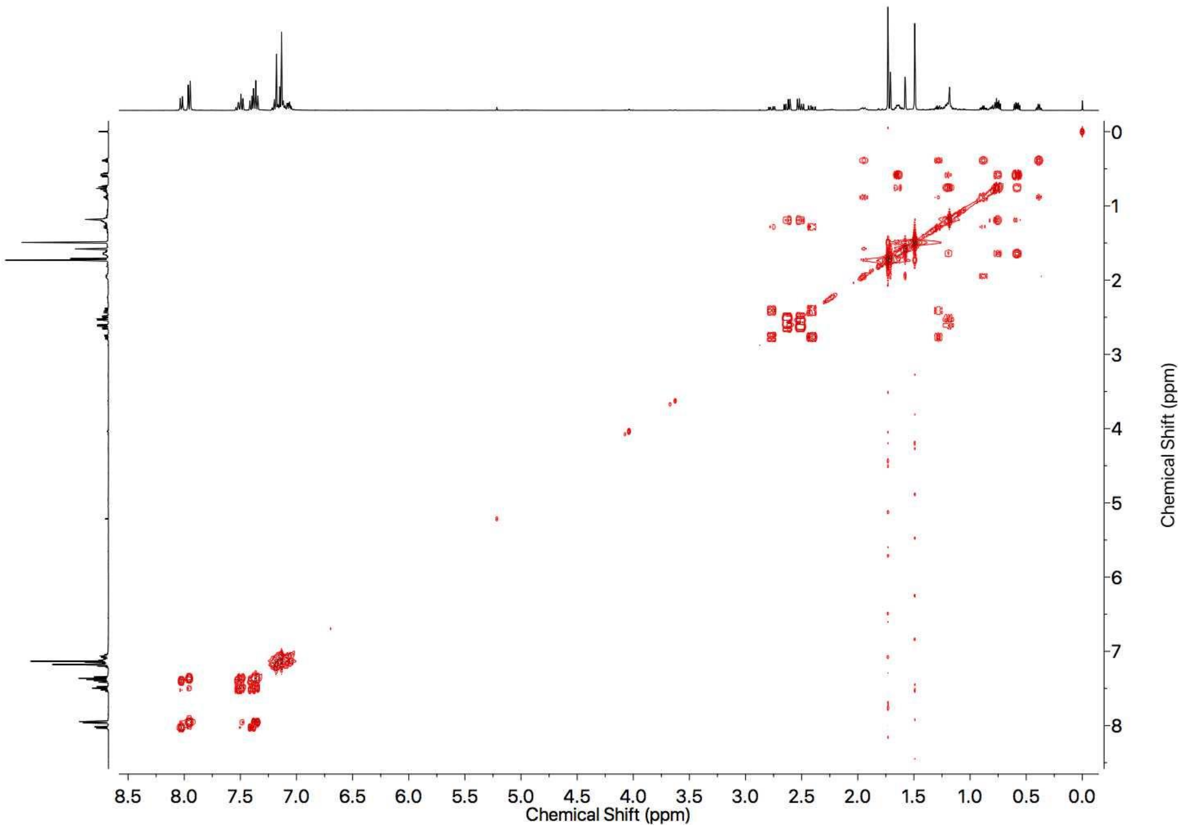

Figure S100: COSY NMR ($CDCl_3$) of cyclopropanes **12**.

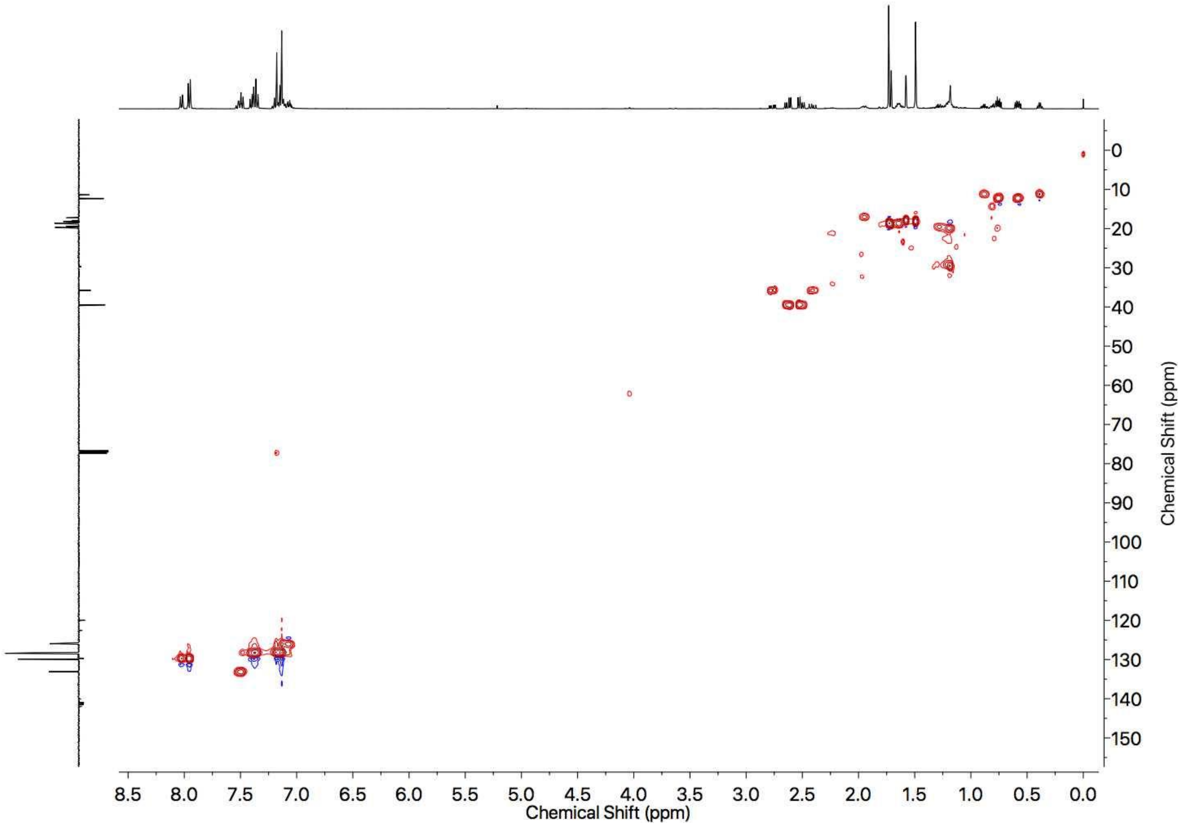

Figure S101: HSQC NMR ($CDCl_3$) of cyclopropanes **12**.

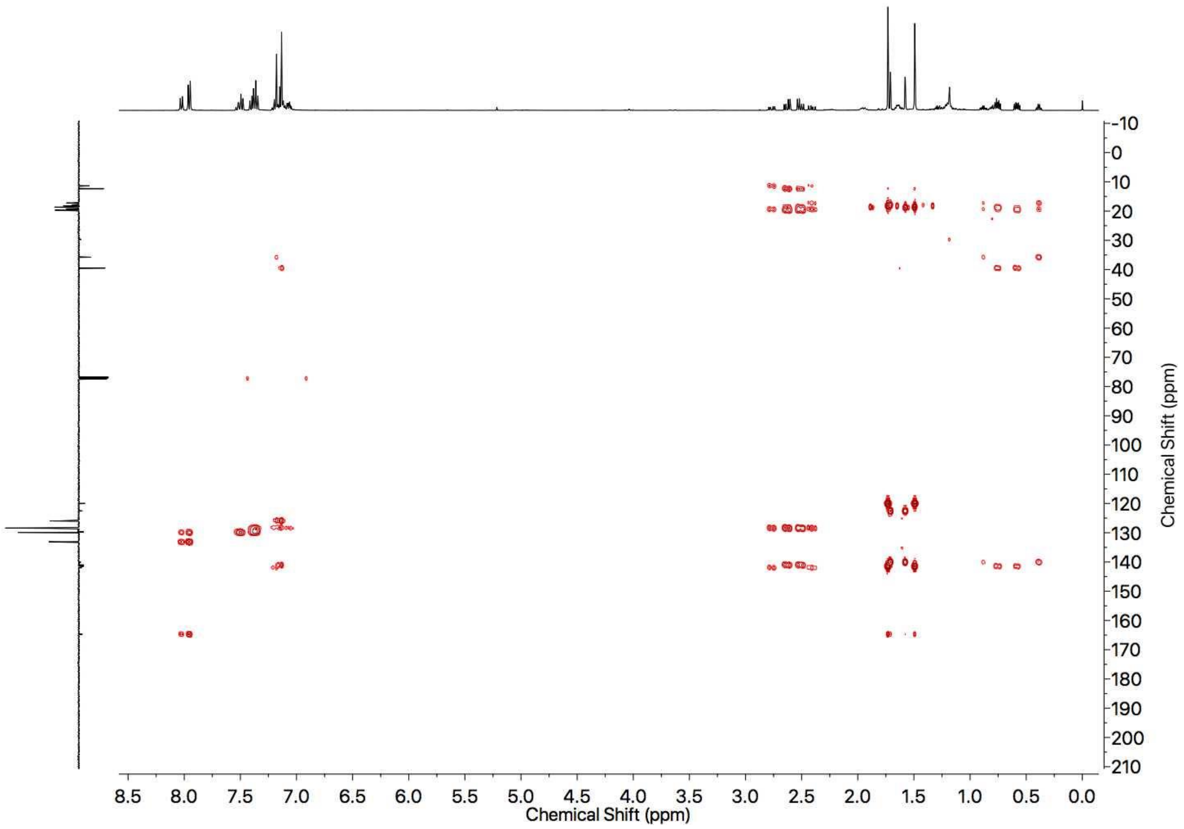

Figure S102: HMBC NMR ($CDCl_3$) of cyclopropanes **12**.

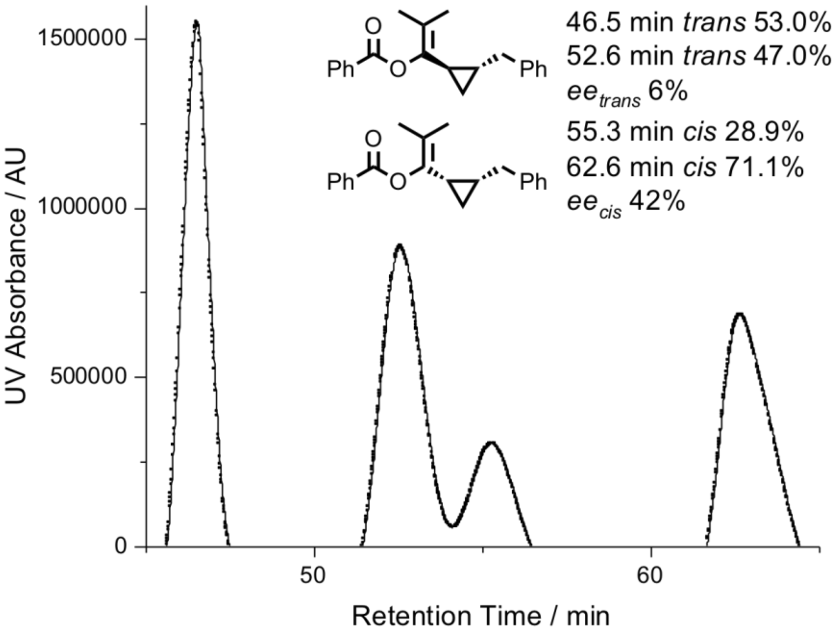

Figure S103: Chiral Stationary Phase HPLC ((S,S)Whelk, isocratic *n*-hexane-isopropanol 99.9 : 0.1, 303 K, loading solvent petrol, 5 µL injection, flowrate 0.25 mLmin^{-1}) 1 : 1 *er trans*-**12** and 64 : 36 *er cis*-**12** (*dr trans-cis* 1.8 : 1.0, not representative of the crude reaction product analysed by ^{1}H NMR). Retention times (min): *trans*-**12** 46.5, *trans*-**12** 52.6, (1*R*,2*R*)-*cis*-**12** 55.3, (1*S*,2*S*)-*cis*-**12** 62.6. The absolute stereochemistry of the products was not determined. (1*S*,2*R*)-**12** is shown for illustrative purposes only.

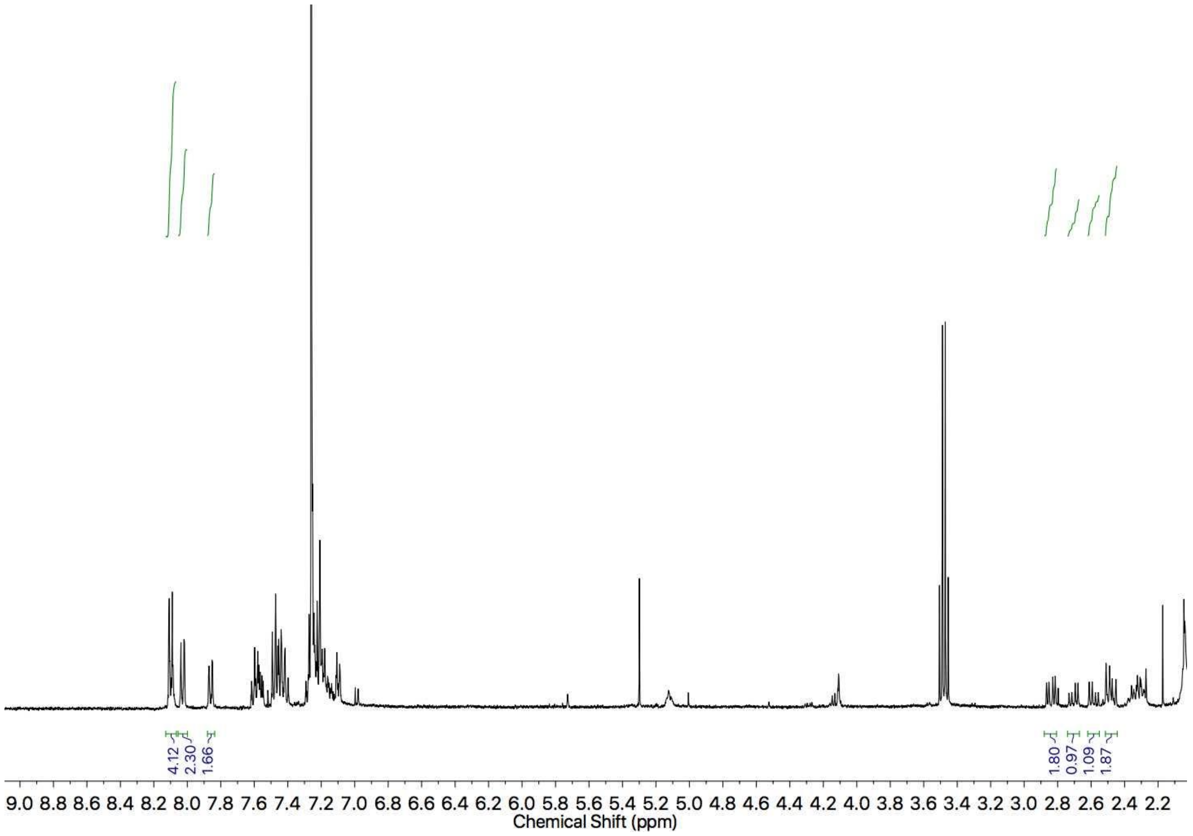

Figure S104: ^{1}H NMR (400 MHz, $CDCl_3$) of *trans*-**12** and *cis*-**12** diastereomer mixture (*dr* 1.8 : 1.0) from column chromatography fraction used for chiral HPLC.

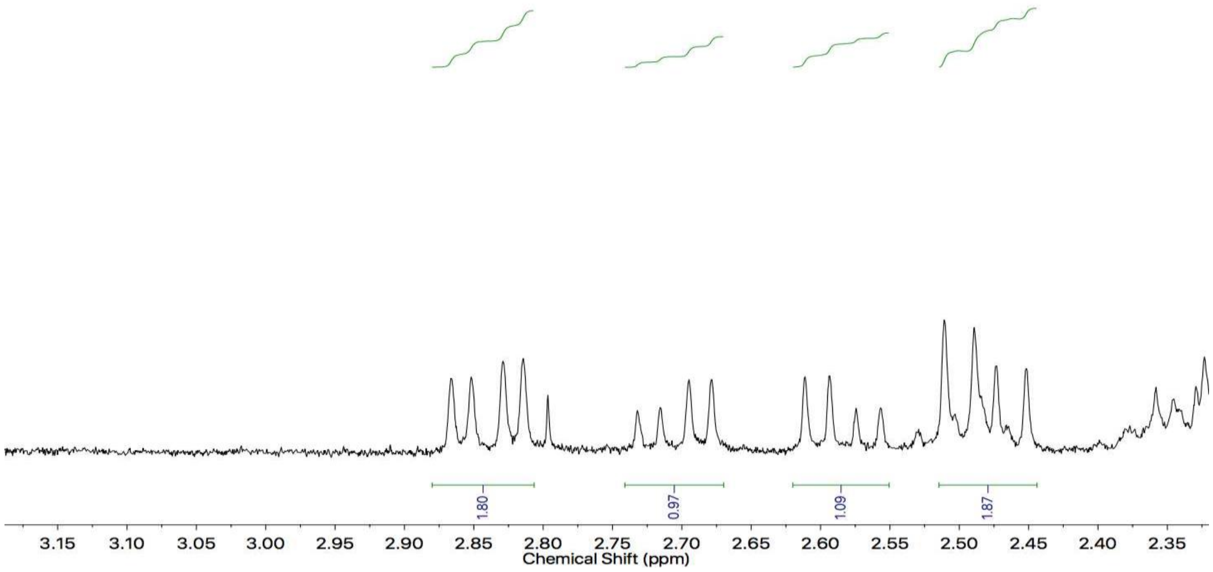

Figure S105: ^{1}H NMR (400 MHz, $CDCl_3$) of *trans*-**12** and *cis*-**12** diastereomer mixture (*dr* 1.8 : 1.0) from column chromatography fraction used for chiral HPLC.

Cyclopropanes **13**[6]

**Catalyst**	**Yield / %**	***dr***	***er*cis**	***ertrans***
$(Ph_3P)AuCl$	44	92 : 8	1 : 1	-
[Au((*R*mp)-**6**)(Cl)]	90	97 : 3	55 : 45	-
(*R*)-DTBM-SEGPHOS®$(AuCl)_2$	70	>20 : 1	91 : 9	-

Table S8. Summary of reactions leading to cyclopropanes **13**.

Cis-**13**

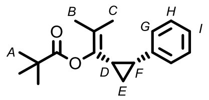

δ_H ($CDCl_3$, 400 MHz) 7.21 (2H, tt, *J* = 7.5, 1.6, H_H), 7.14 (1H, tt, *J* = 7.4, 1.3, H_I), 7.03 (2H, dd, *J* = 7.5, 1.3, H_G), 2.28 (1H, d, *J* = 6.5, H_E), 2.26 (1H, d, *J* = 6.5, H_D), 1.48 (3H, s, H_C), 1.41 (3H, s, H_B), 1.26 (1H, m, H_F), 1.22 (9H, s, H_A), 1.00 (1H, dt, *J* = 6.0, 6.0, $H_{F'}$).

δ_C ($CDCl_3$, 101 MHz) 176.9, 139.6, 138.2, 127.6, 127.4, 125.6, 123.2, 39.0, 27.4, 24.1, 21.9, 18.7, 17.4, 11.9.

HR-EI-MS m/z 272.1766 [$M^{+\cdot}$] (calc. m/z for $C_{18}H_{24}O_2$ 272.1771).

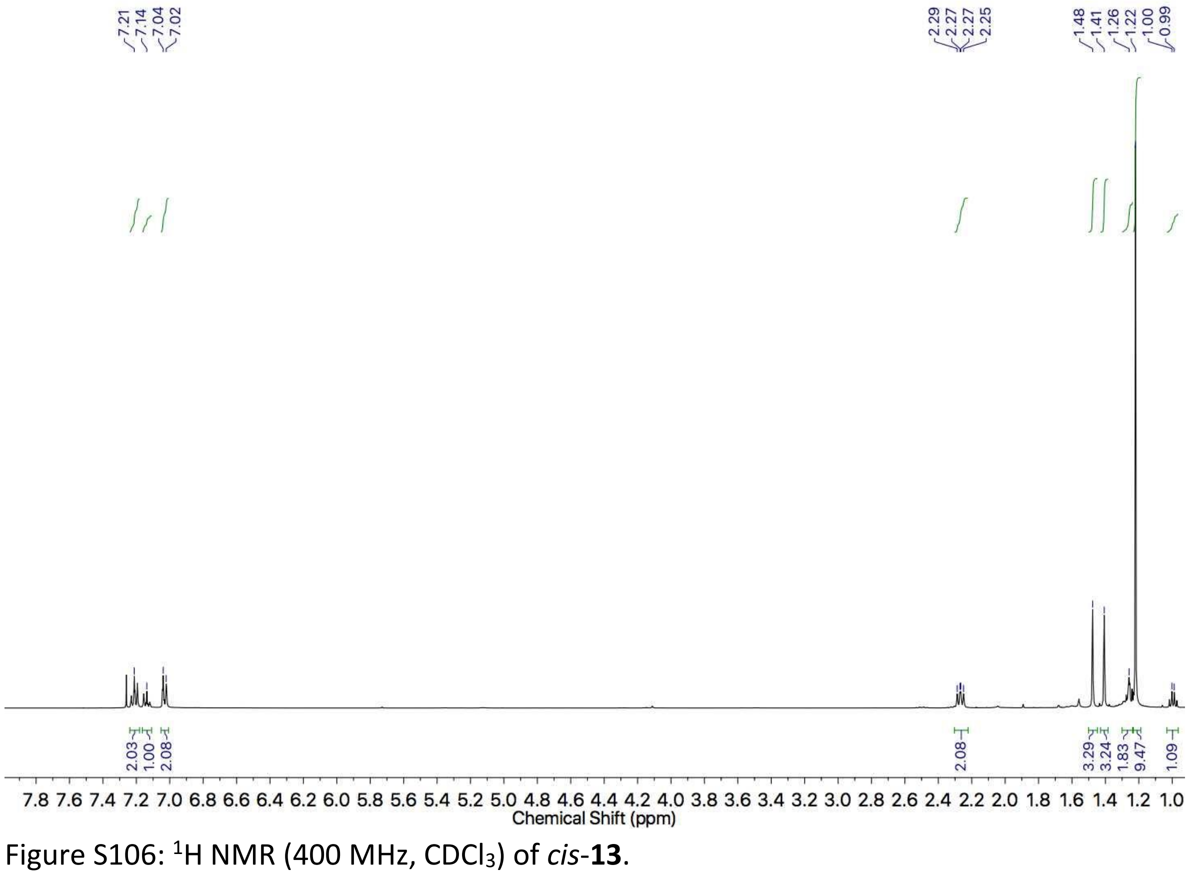

Figure S106: ^{1}H NMR (400 MHz, $CDCl_3$) of *cis*-**13**.

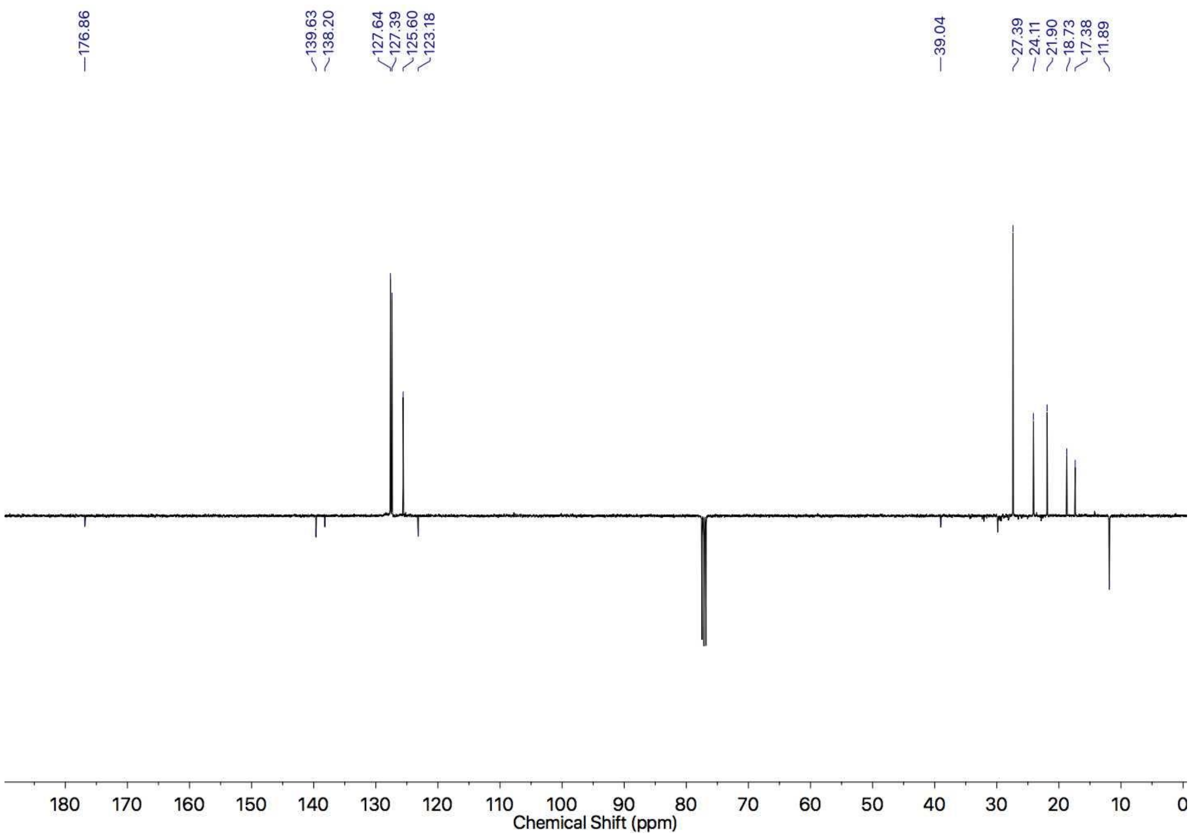

Figure S107: JMOD NMR (101 MHz, $CDCl_3$) of *cis*-**13**.

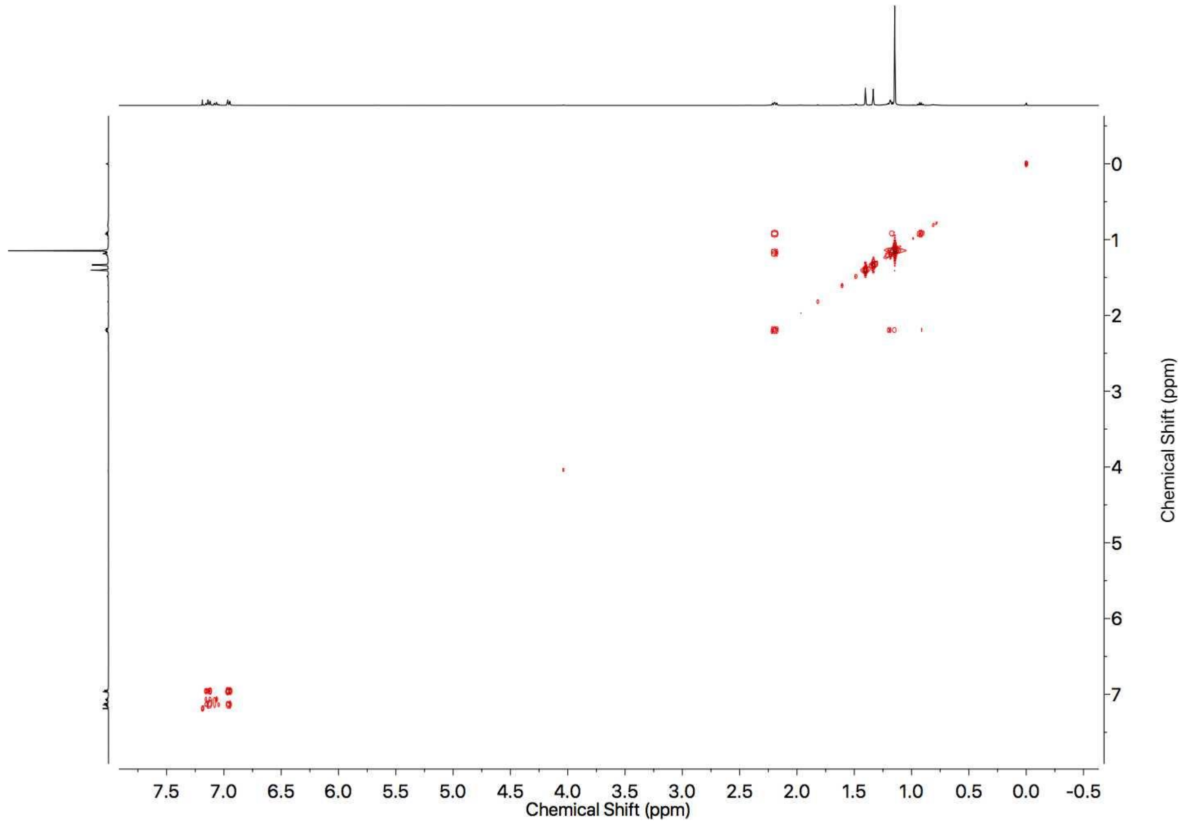

Figure S108: COSY NMR ($CDCl_3$) of *cis*-**13**.

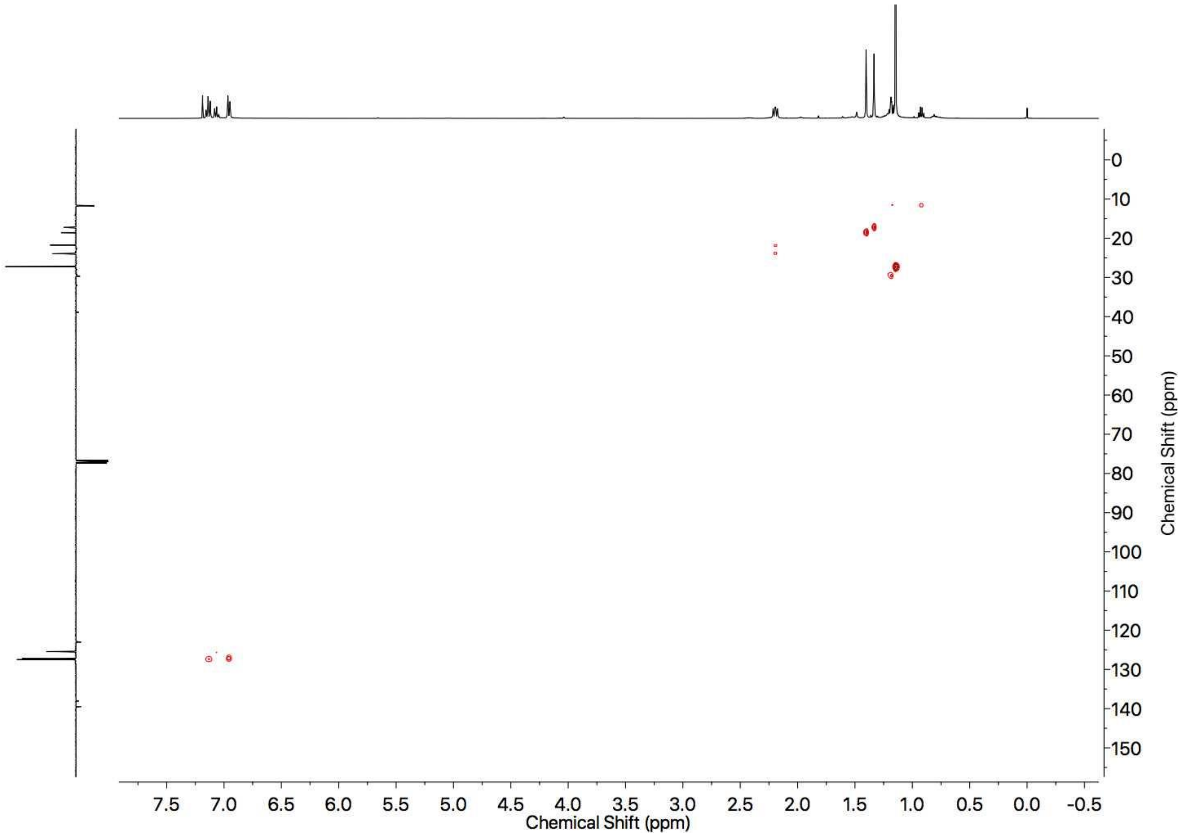

Figure S109: HSQC NMR ($CDCl_3$) of *cis*-**13**.

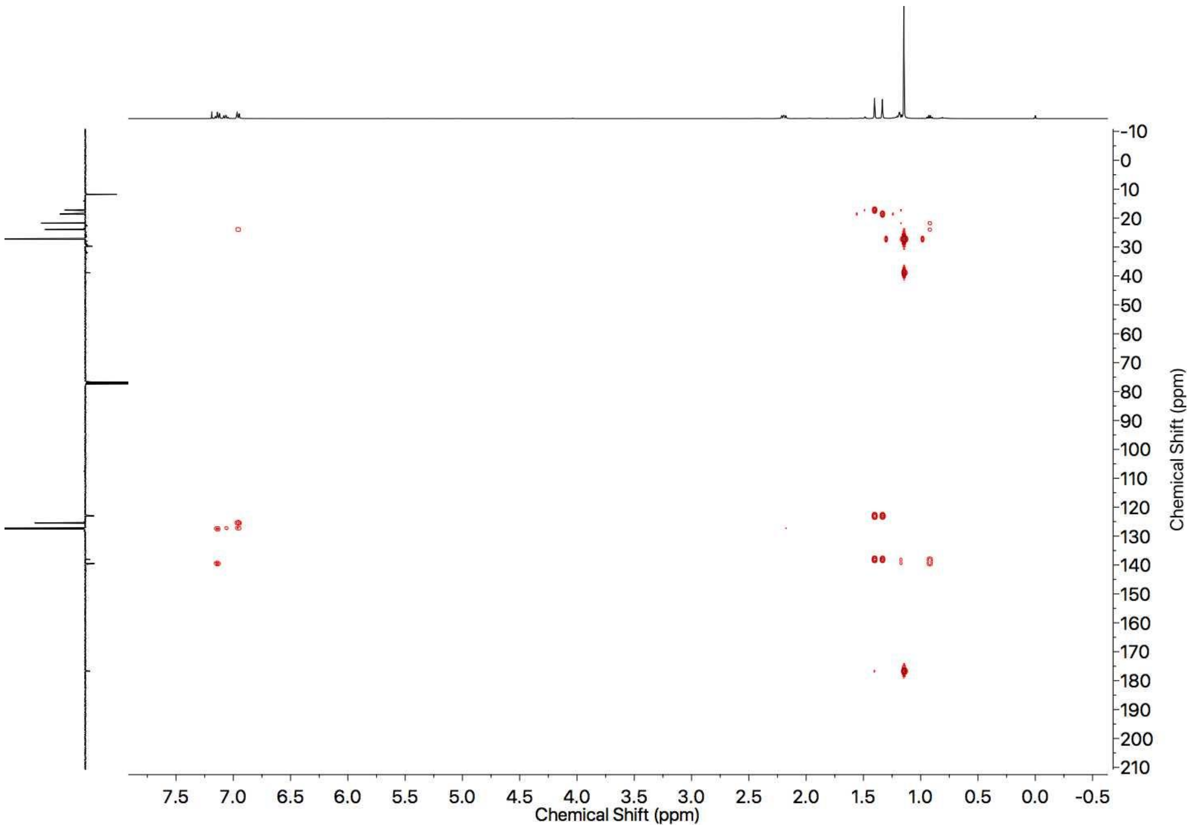

Figure S110: HMBC NMR ($CDCl_3$) of *cis*-**13**.

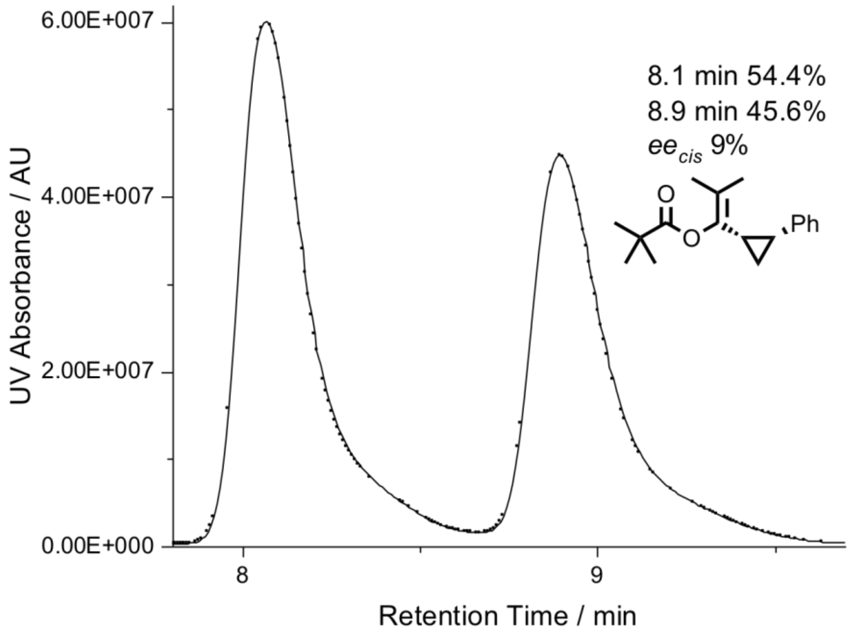

Figure S111: Chiral Stationary Phase HPLC (RegisCell, *n*-hexane-isopropanol 99.5 : 0.5, 303 K, load solvent Et_2O, flowrate 0.50 $mLmin^{-1}$) of 54 : 46 *er cis*-**13**. Retention times (min): 8.1, 8.9. The absolute stereochemistry of the products was not determined. The (1*S*,2*R*)-**13** isomer is shown for illustrative purposes only.

Cyclopropanes **14**

**Catalyst**	**Yield / %**	***dr***	***er*** cis	***er*** *trans*
$(Ph_3P)AuCl$	63	90 : 10	1 : 1	1 : 1
[Au((*R*mp)-**6**)(Cl)]	40	97 : 3	77 : 23	-

Table S9. Summary of reactions leading to cyclopropanes **14**.

Cis-**14**

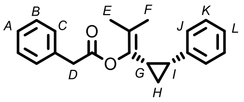

δ_H ($CDCl_3$, 400 MHz) 7.38-7.28 (5H, m, ***H***A, ***H***B, ***H***C), 7.21 (2H, tt, *J* = 7.2, 1.7, ***H***K), 7.15 (1H, tt, *J* = 7.2, 1.5, ***H***L), 6.95 (2H, dt, *J* = 6.9, 1.9, ***H***J), 3.61 (2H, s, ***H***D), 2.27-2.21 (2H, m, ***H***G, ***H***H), 1.47 (3H, s, ***H***F), 1.34 (3H, s, ***H***E), 1.13 (1H, td, *J* = 8.8, 5.4, ***H***I), 0.82 (1H, td, *J* = 6.4, 5.4, ***H***I').

δ_C ($CDCl_3$, 101 MHz) 169.8, 139.4, 138.4, 134.1, 129.5, 128.7, 127.7, 127.4, 127.3, 125.6, 123.5, 41.5, 24.1, 21.7, 18.6, 17.5, 11.4.

HR-EI-MS m/z 306.1607 [$M^{+\cdot}$] (calc. m/z for $C_{21}H_{22}O_2$ 306.1620).

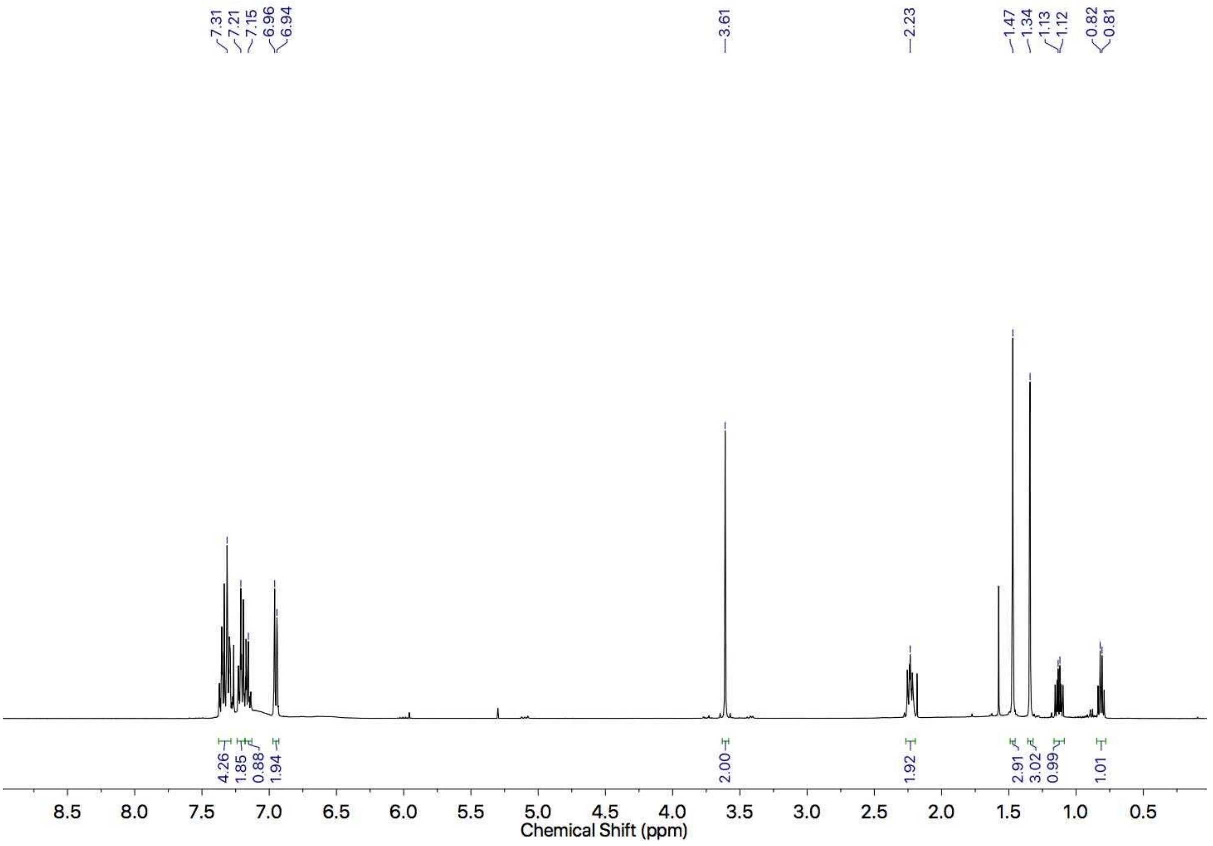

Figure S112: ^{1}H NMR (400 MHz, $CDCl_3$) of *cis*-**14**.

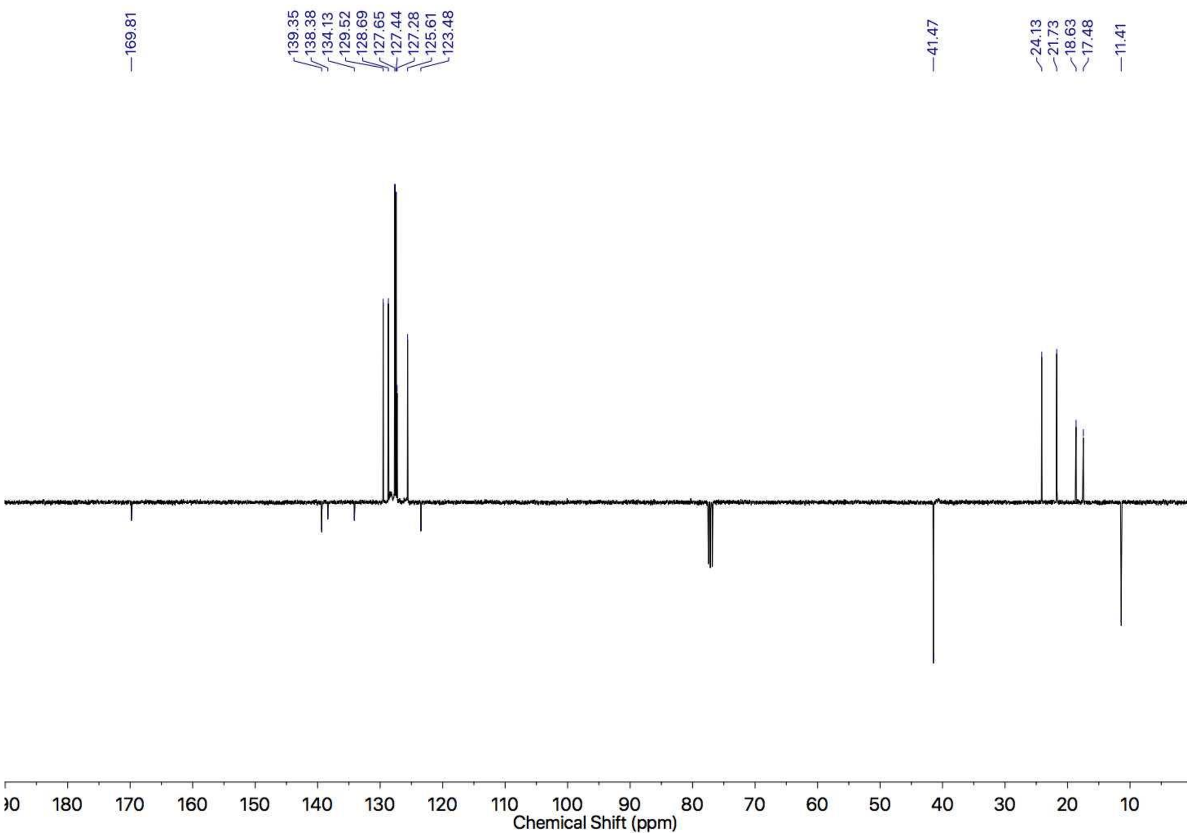

Figure S113: JMOD NMR (101 MHz, $CDCl_3$) of *cis*-**14**.

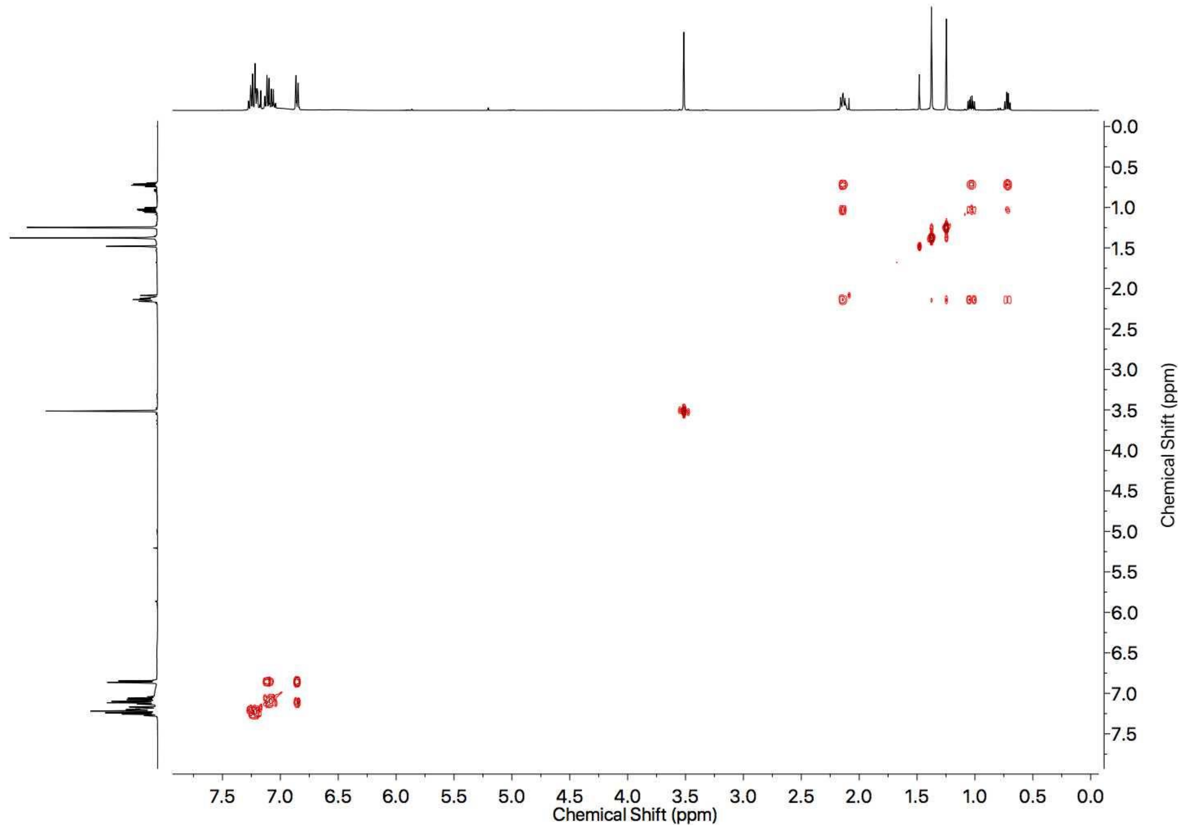

Figure S114: COSY NMR ($CDCl_3$) of *cis*-**14**.

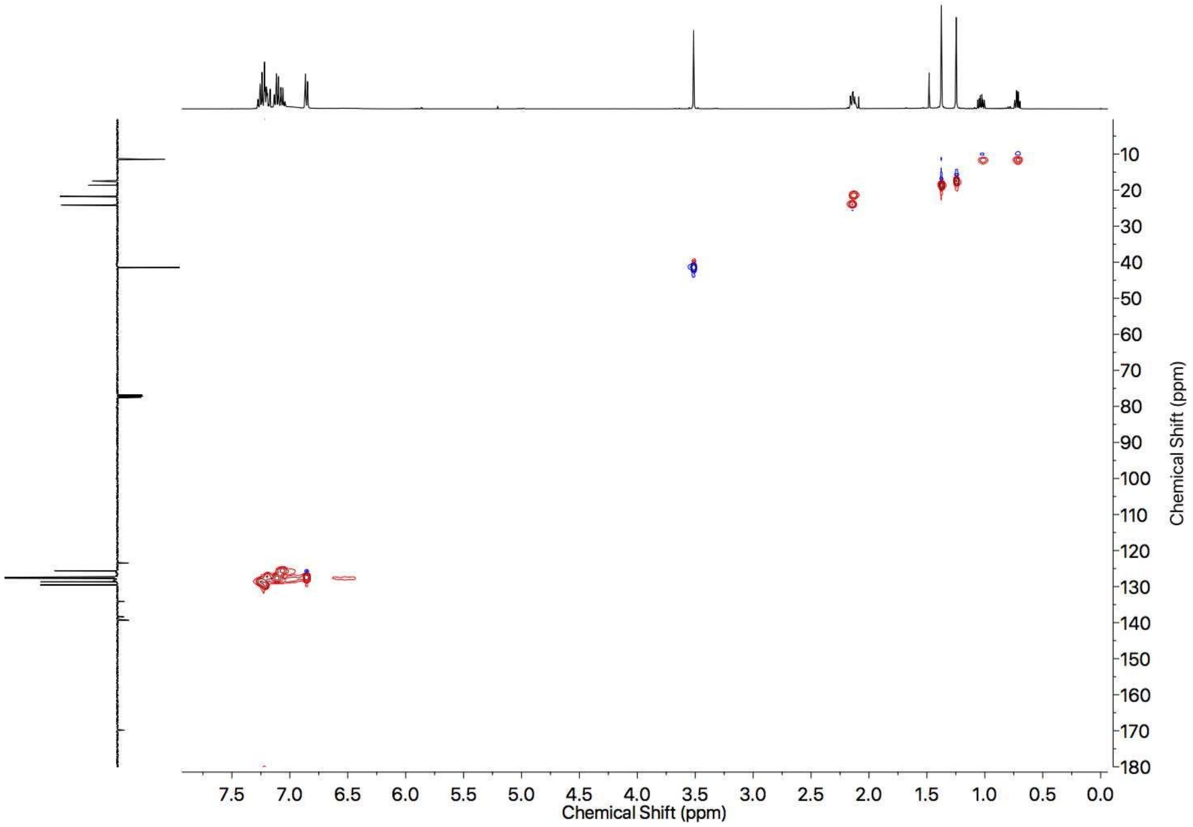

Figure S115: HSQC NMR ($CDCl_3$) of *cis*-**14**.

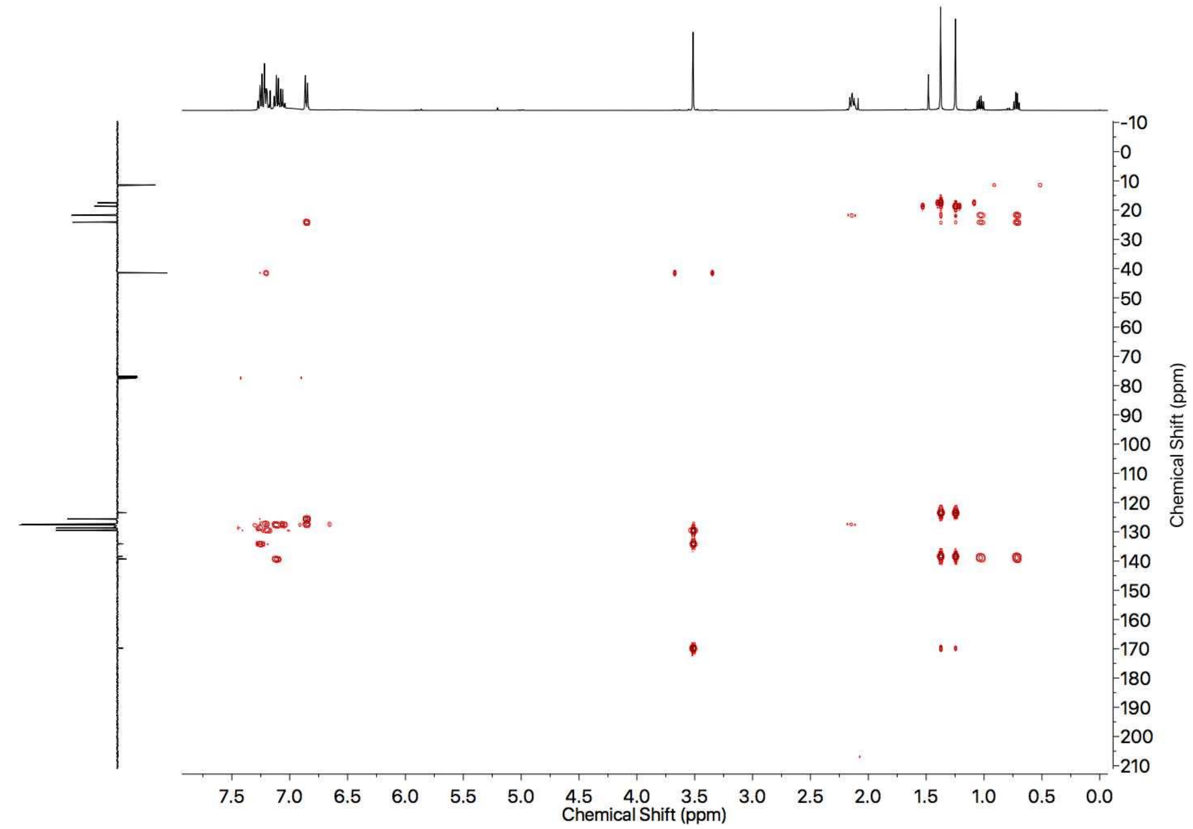

Figure S116: HMBC NMR ($CDCl_3$) of *cis*-**14**.

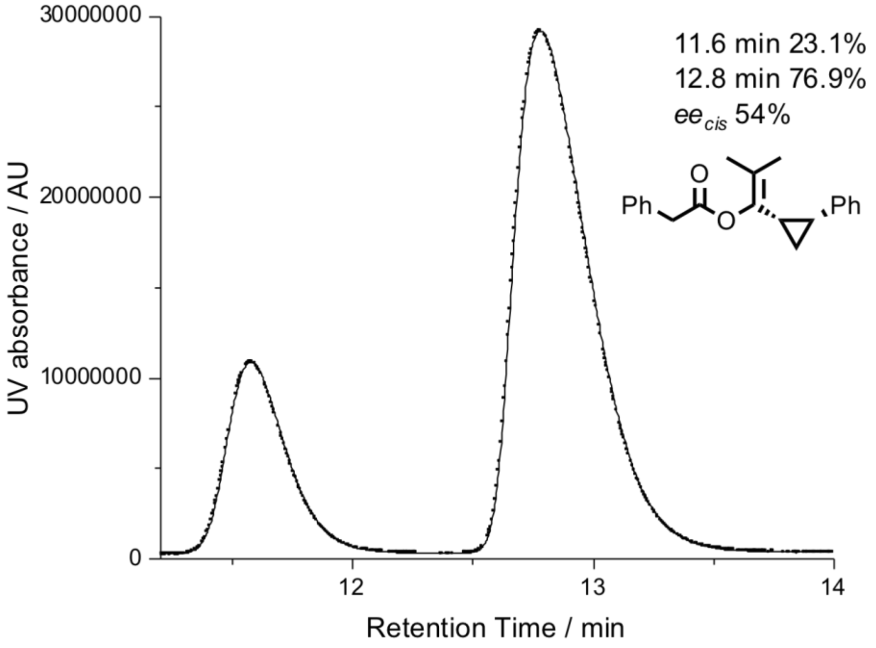

Figure S117: Chiral Stationary Phase HPLC ((*S*,*S*)Whelk, *n*-hexane-isopropanol 99.5 : 0.5, 303 K, load petrol, flowrate 0.50 mLmin^{-1}) of 77 : 23 *er cis*-**14**. Retention times (min): 11.6, 12.8. The absolute stereochemistry of the products was not determined. The (1*S*,2*R*)-**14** isomer is shown for illustrative purposes only.

Cyclopropanes **15**

**Catalyst**	**Yield / %**	***dr***	***er*cis**	***er*trans**
$(Ph_3P)AuCl$[a]	79	82 : 18	1 : 1	1 : 1
[Au((*R*mp)-**6**)(Cl)][b]	45	96 : 4	73 : 27	-

Table S10. Summary of reactions leading to cyclopropanes **15**.

Cis-**15**

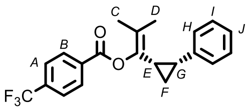

δ_H ($CDCl_3$, 400 MHz) 7.88 (2H, d, *J* = 8.2, ***H***B), 7.66 (2H, d, *J* = 8.4, ***H***A), 7.25 (2H, tt, *J* = 6.5, 2.0, ***H***I), 7.20 (1H, tt, *J* = 7.1, 1.5, ***H***J), 7.09 (2H, dd, *J* = 8.4, 1.7, ***H***H), 2.47-2.28 (2H, m, ***H***E, ***H***F), 1.70 (3H, s, ***H***D), 1.48 (3H, s, ***H***C), 1.33 (1H, ddd, *J* = 14.3, 8.8, 5.4, ***H***G), 1.09 (1H, q, *J* = 6.0, ***H***G′).

δ_C ($CDCl_3$, 101 MHz) 163.5, 139.4, 138.9, 134.7 (q, *J* = 32.4), 134.5, 130.4, 127.9, 127.8, 125.9, 125.5 (q, *J* = 3.7), 124.0, 122.4, 23.7, 21.2, 18.9, 17.8, 12.2.

δ_F ($CDCl_3$, 376 MHz) -63.4.

$\delta_{F\{H\}}$ ($CDCl_3$, 376 MHz) -63.4.

HR-EI-MS m/z 360.1325 [$M^{+\cdot}$] (calc. m/z for $C_{21}H_{19}O_2F_3$ 360.1332).

Trans-**15**

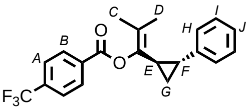

δ_H ($CDCl_3$, 400 MHz) 8.24 (2H, d, *J* = 8.2, ***H***B), 7.78 (2H, d, *J* = 8.2, ***H***A), 7.30-7.23 (2H, m, ***H***I), 7.17 (1H, tt, *J* = 7.5, 1.3, ***H***J), 7.09 (2H, dd, *J* = 7.8, 1.5, ***H***H), 2.13 (1H, ddd, *J* = 9.5, 7.7, 3.2, ***H***E), 2.03 (1H, d, *J* = 1.2, ***H***F), 1.87 (3H, s, ***H***D), 1.62 (3H, s, ***H***C), 1.20-1.15 (2H, m, ***H***G, ***H***G′).

δ_C ($CDCl_3$, 101 MHz) 167.6, 164.5, 141.1, 135.5, 134.5, 130.5, 128.5, 126.1, 126.0, 125.8, 122.7, 121.6, 24.0, 23.3, 19.0, 18.3, 14.9.

δ_F ($CDCl_3$, 376 MHz) -63.4.

$\delta_{F\{H\}}$ ($CDCl_3$, 376 MHz) -63.4.

HR-EI-MS m/z 360.1326 [$M^{+\cdot}$] (calc. m/z for $C_{21}H_{19}O_2F_3$ 360.1332).

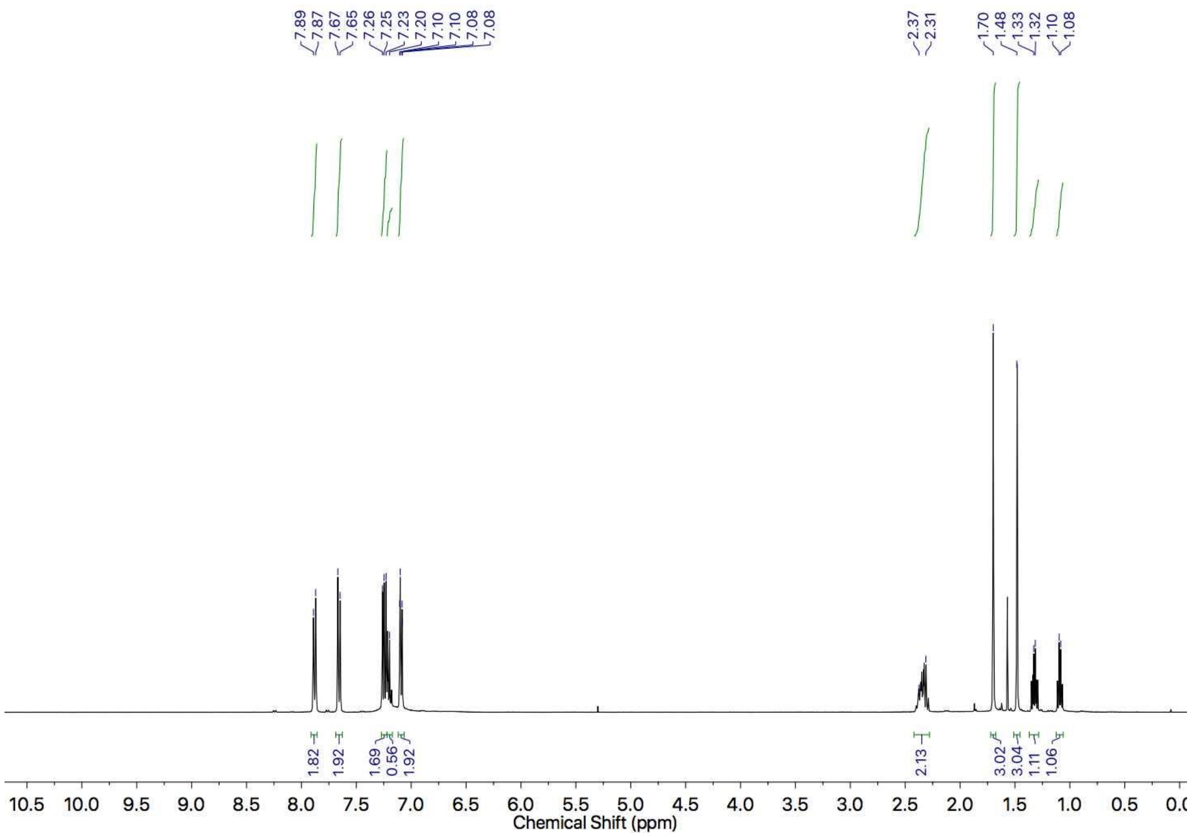

Figure S118: ^{1}H NMR (400 MHz, $CDCl_3$) of *cis*-**15**.

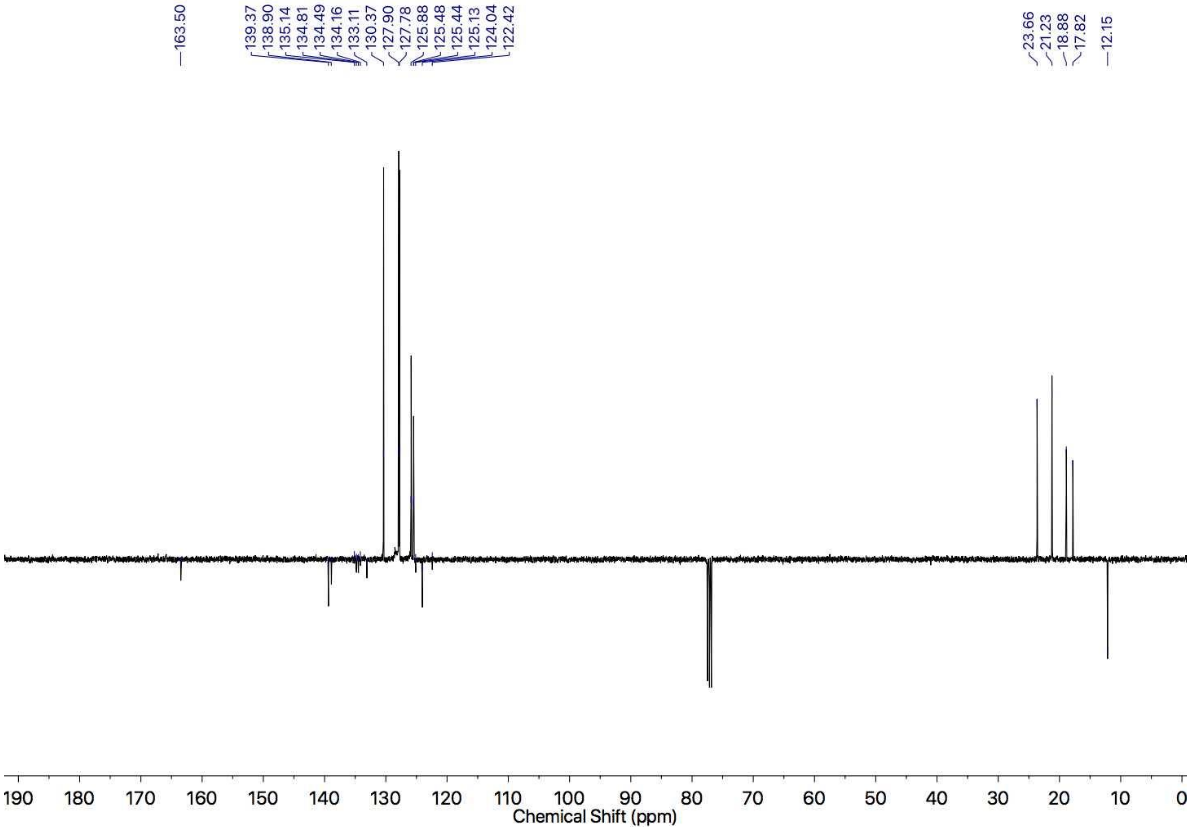

Figure S119: JMOD NMR (101 MHz, $CDCl_3$) of *cis*-**15**.

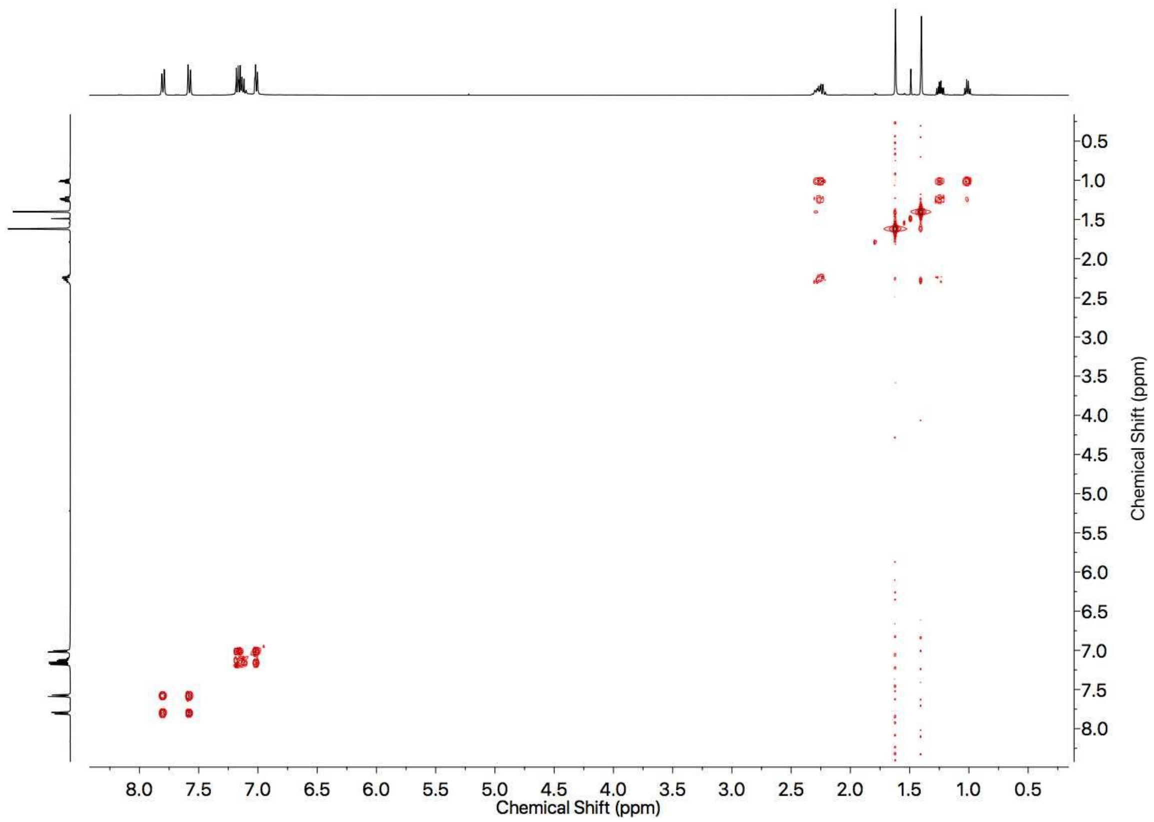

Figure S120: COSY NMR ($CDCl_3$) of *cis*-**15**.

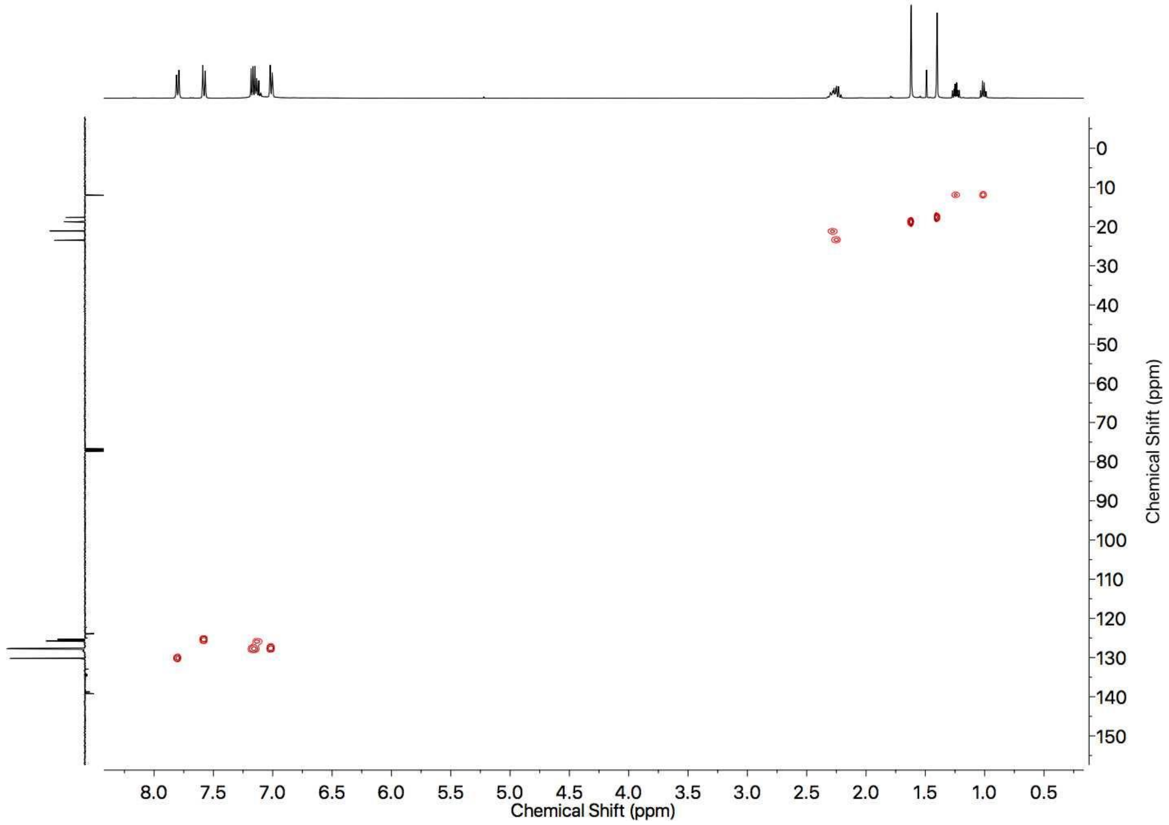

Figure S121: HSQC NMR ($CDCl_3$) of *cis*-**15**.

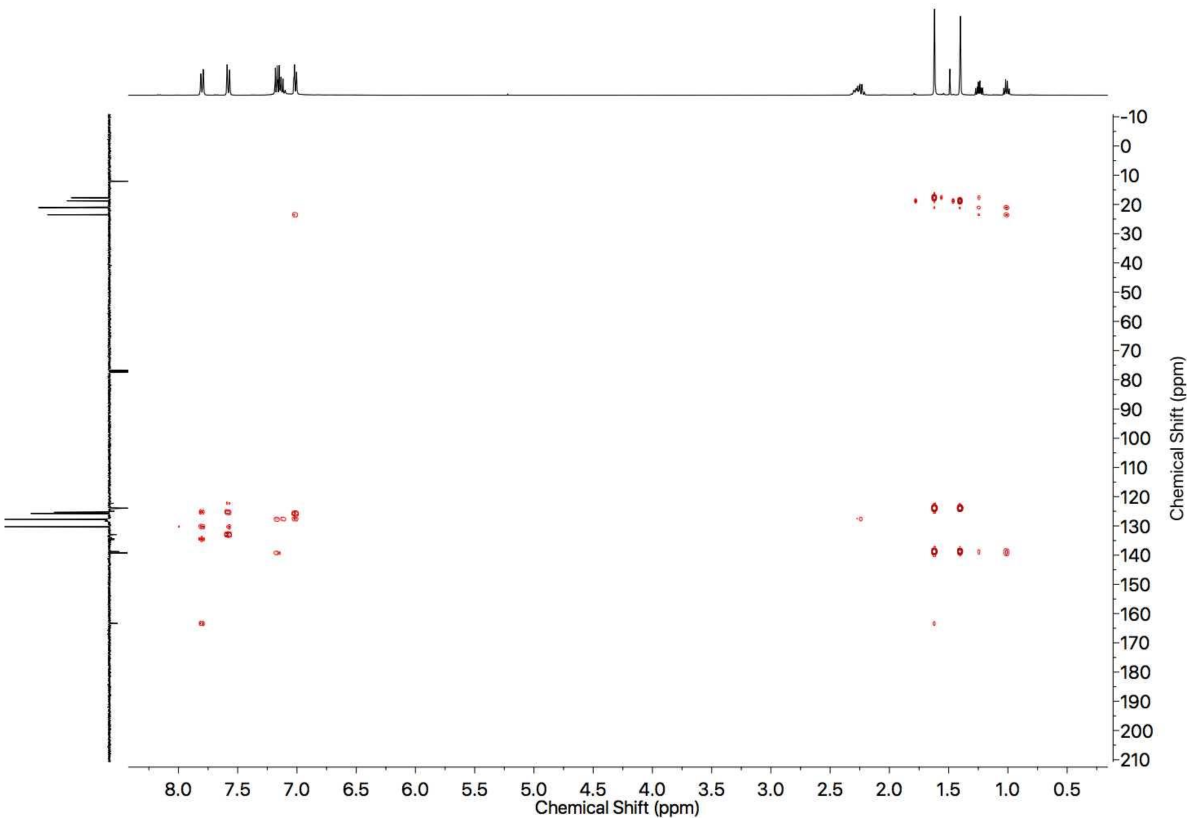

Figure S122: HMBC NMR ($CDCl_3$) of *cis*-**15**.

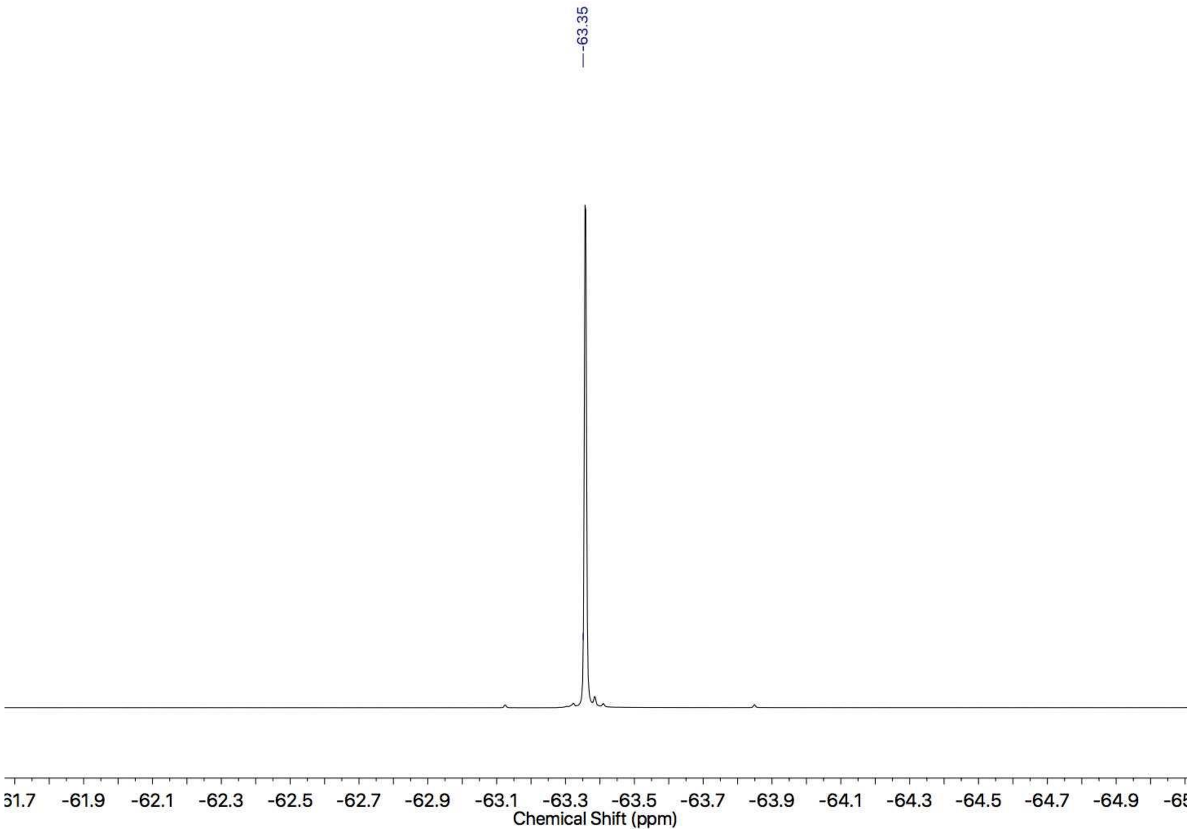

Figure S123: ^{19}F NMR (376 MHz, $CDCl_3$) of *cis*-**15**.

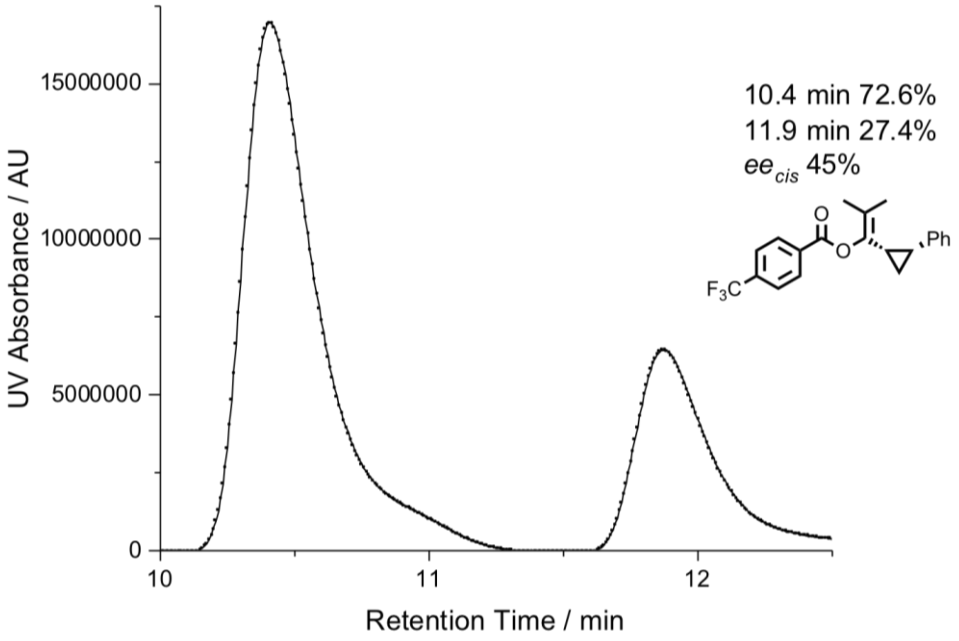

Figure S124: Chiral Stationary Phase HPLC (RegisCell, *n*-hexane-isopropanol 99.9 : 0.1, 303 K, load Et_2O, flowrate 0.75 $mLmin^{-1}$) of 73 : 27 *er cis*-**15**. Retention times (min): 10.4, 11.9. The absolute stereochemistry of the major product was determined to be (1*S*,2*R*)-**15** by reduction with $LiAlH_4$ and comparison with the product of the same reaction with cyclopropane **9** (see **Figure S168**).

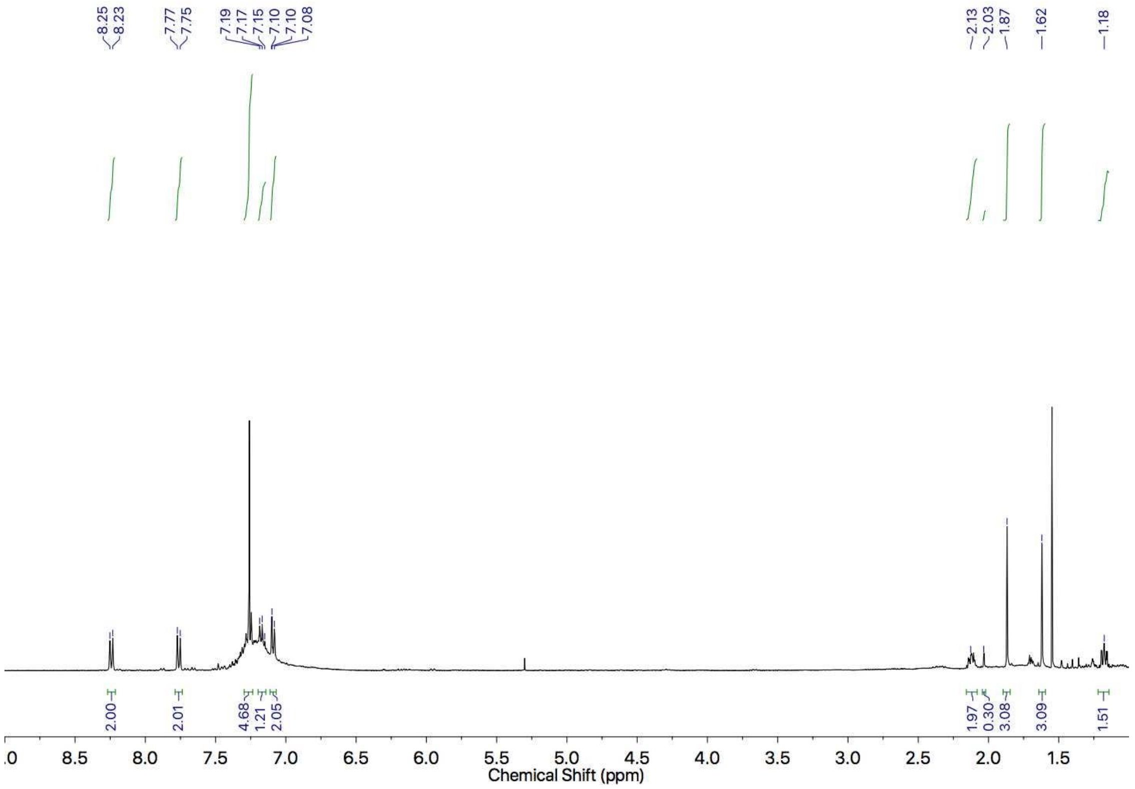

Figure S125: ^1H NMR (400 MHz, $CDCl_3$) of *trans*-**15**.

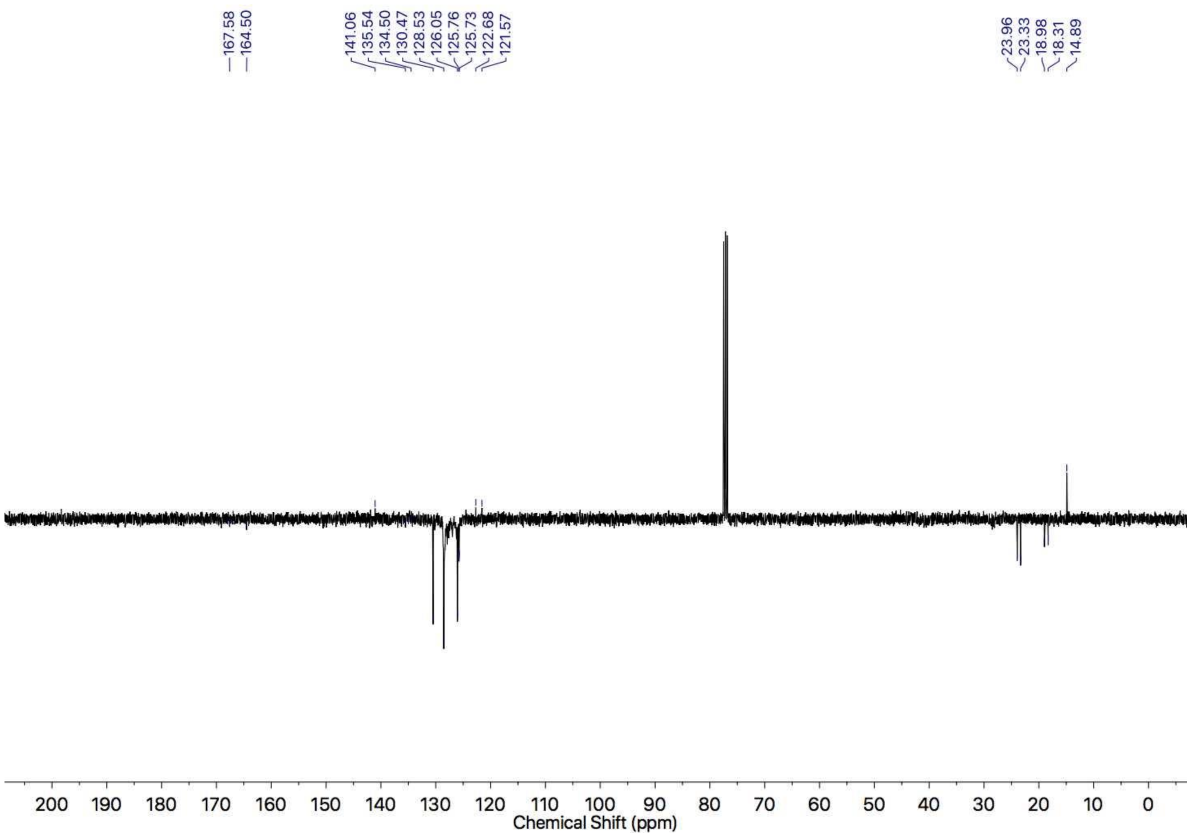

Figure S126: JMOD NMR (101 MHz, $CDCl_3$) of *trans*-**15**.

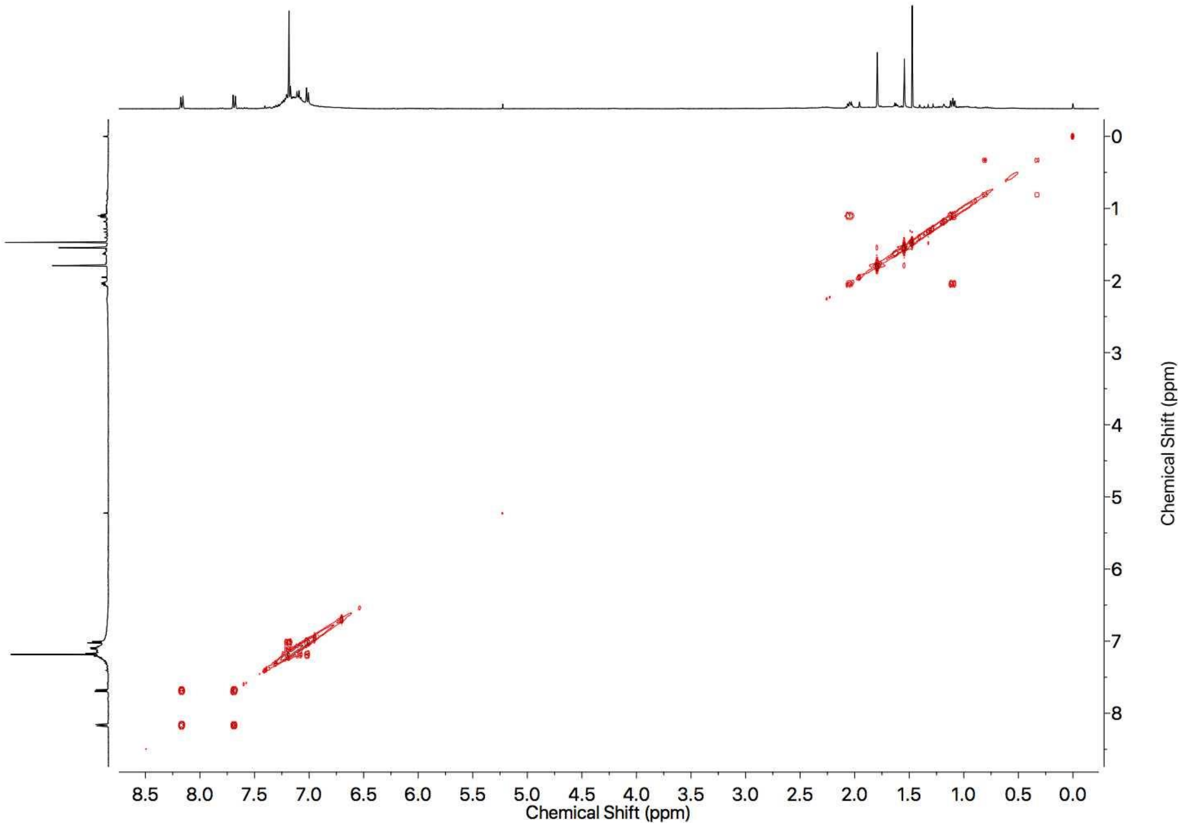

Figure S127: COSY NMR ($CDCl_3$) of *trans*-**15**.

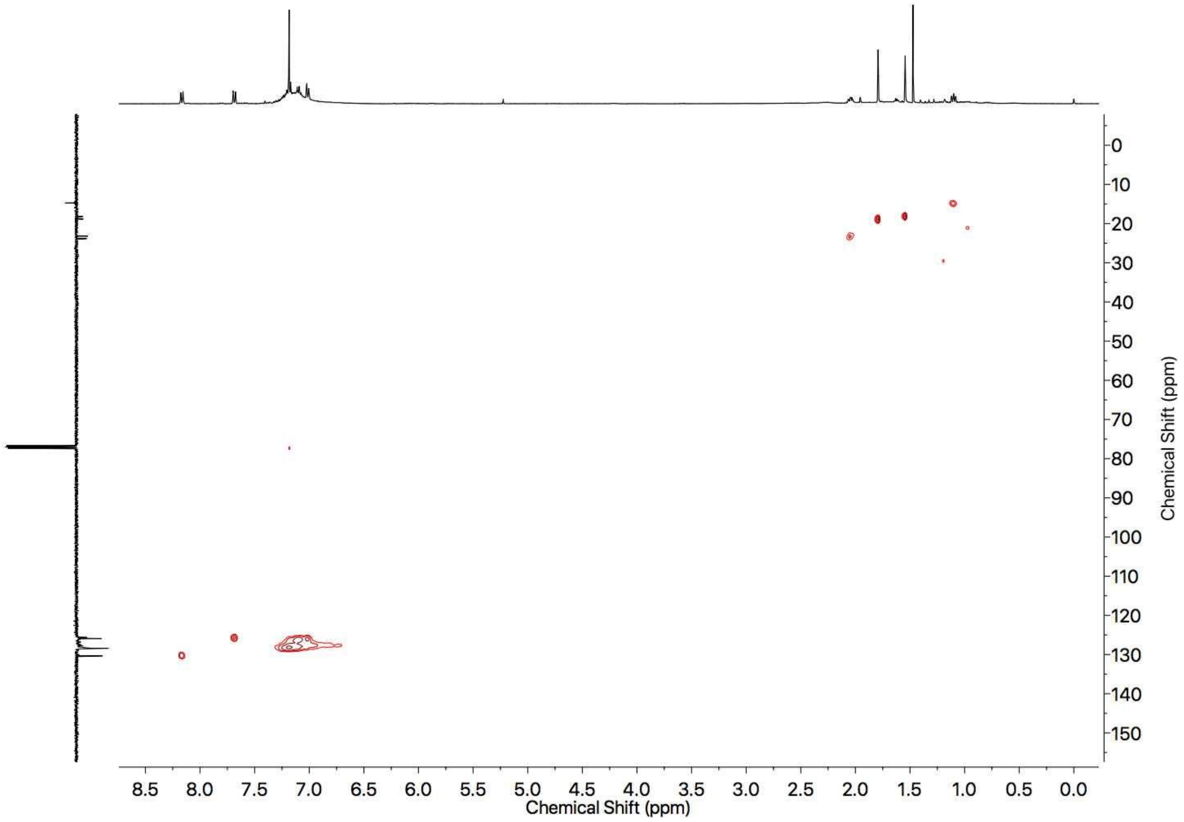

Figure S128: HSQC NMR ($CDCl_3$) of *trans*-**15**.

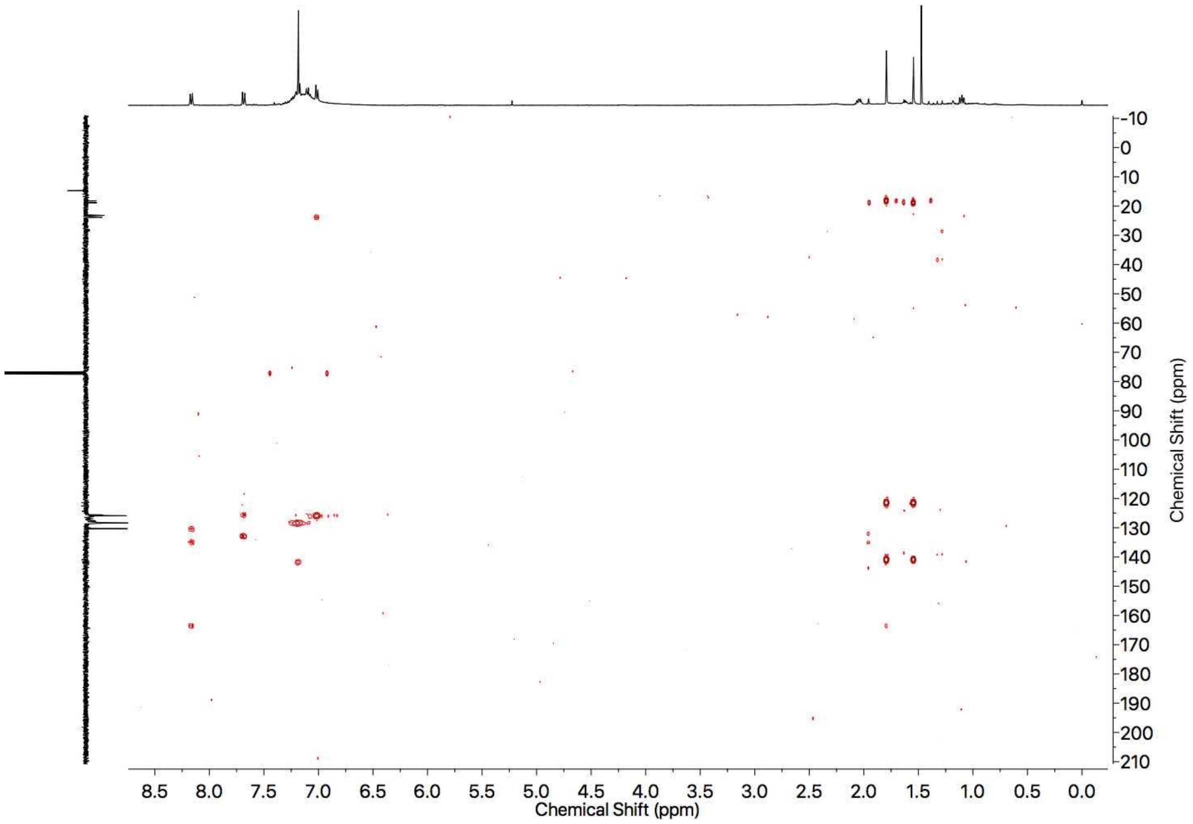

Figure S129: HMBC NMR ($CDCl_3$) of *trans*-**15**.

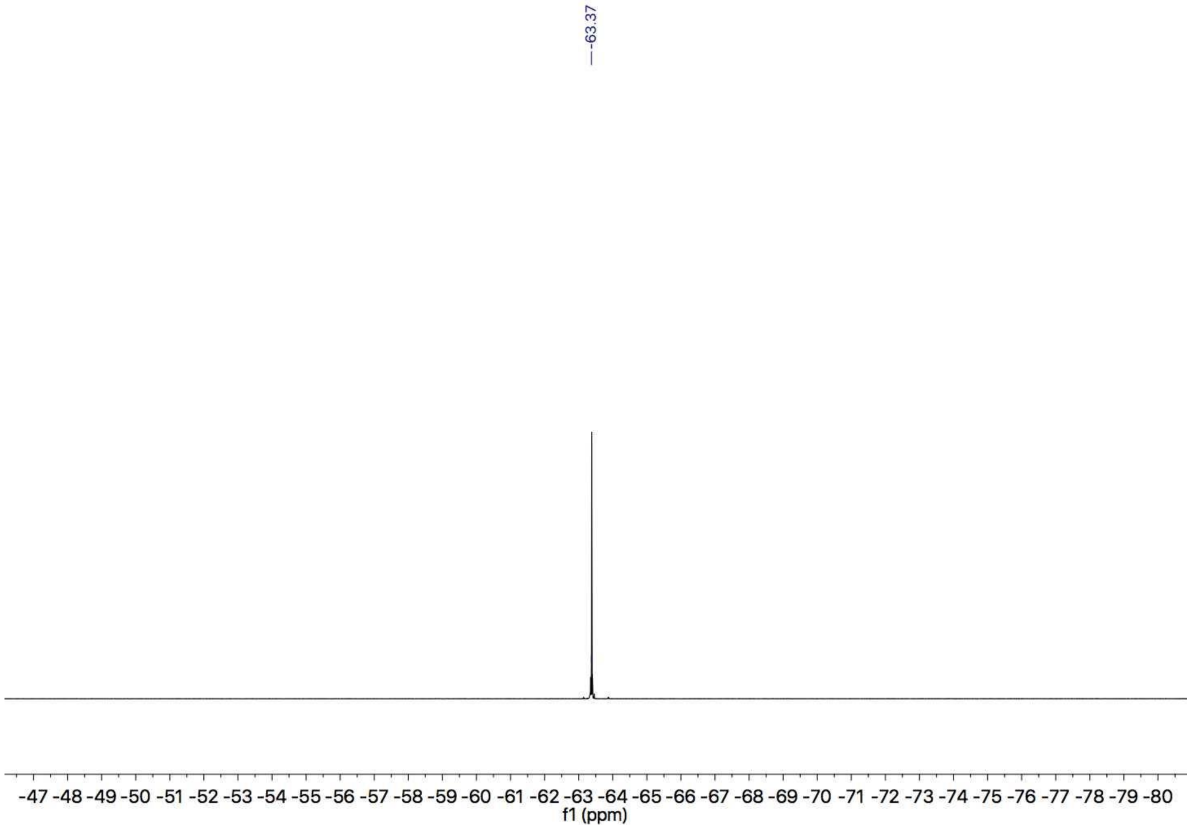

Figure S130: ^{19}F NMR (376 MHz, $CDCl_3$) of *trans*-**15**.

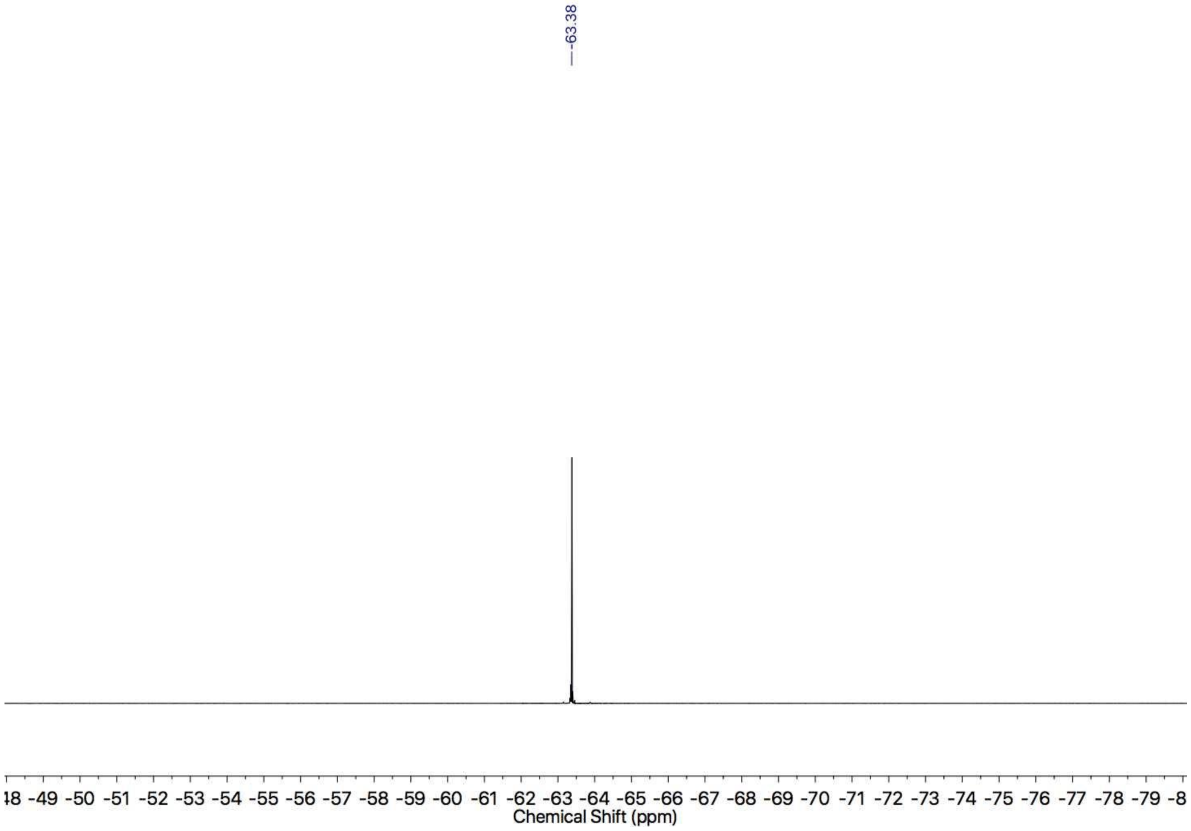

Figure S131: $^{19}F\{^1H\}$ NMR (376 MHz, $CDCl_3$) of *trans*-**15**.

Cyclopropanes **16**

**Catalyst**	**Yield / %**	***dr***	***er*$_{cis}$**	***er*$_{trans}$**
$(Ph_3P)AuCl$[a]	62	90 : 10	1 : 1	1 : 1
[Au((R_{mp})-**6**)(Cl)][b]	48	94 : 6	71 : 29	73 : 27

Table S11. Summary of reactions leading to cyclopropanes **16**.

Cis-**16**

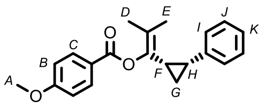

δ_H ($CDCl_3$, 400 MHz) 7.81 (2H, dt, *J* = 9.0, 2.4, $\mathbf{H}_C$), 7.24 (2H, br. dd, *J* = 7.7, 7.2, $\mathbf{H}_J$), 7.18 (1H, tt, *J* = 7.2, 1.5, $\mathbf{H}_K$), 7.10 (2H, dd, *J* = 7.6, 1.6, $\mathbf{H}_I$), 6.89 (2H, dt, *J* = 9.0, 2.4, $\mathbf{H}_B$), 3.87 (3H, s, $\mathbf{H}_A$), 2.40-2.27 (2H, m, $\mathbf{H}_F$, $\mathbf{H}_G$), 1.63 (3H, s, $\mathbf{H}_E$), 1.46 (3H, s, $\mathbf{H}_D$), 1.26 (1H, ddd, *J* = 14.2, 8.8, 5.6, $\mathbf{H}_H$), 1.09 (1H, q, *J* = 5.9, $\mathbf{H}_{H'}$).

δ_C ($CDCl_3$, 101 MHz) 164.6, 163.6, 139.6, 138.6, 132.1, 127.8, 127.8, 125.7, 123.4, 122.3, 113.7, 55.6, 23.9, 21.6, 18.8, 17.8, 11.8.

HR-EI-MS m/z 322.1556 [$M^{+\cdot}$] (calc. m/z for $C_{21}H_{22}O_3$ 322.1563).

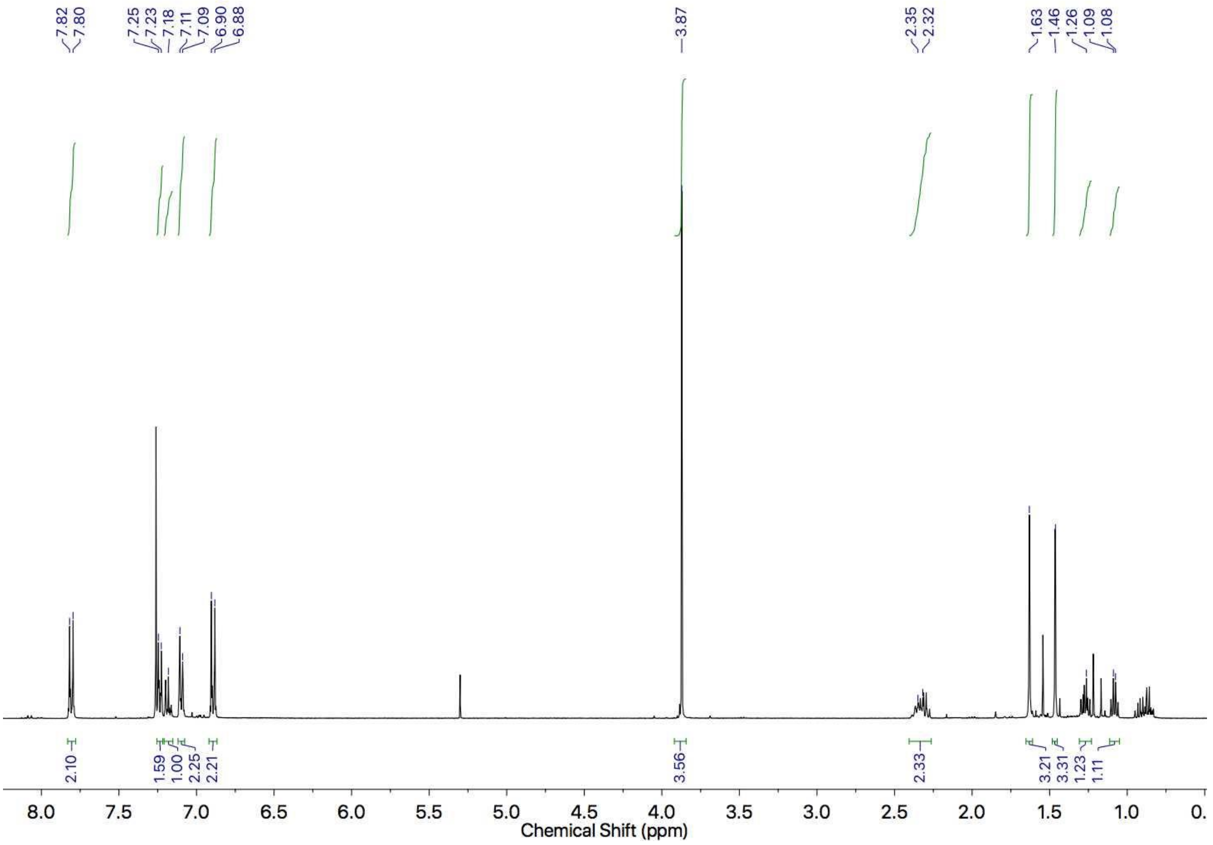

Figure S132: ^1H NMR (400 MHz, $CDCl_3$) of *cis*-**16**.

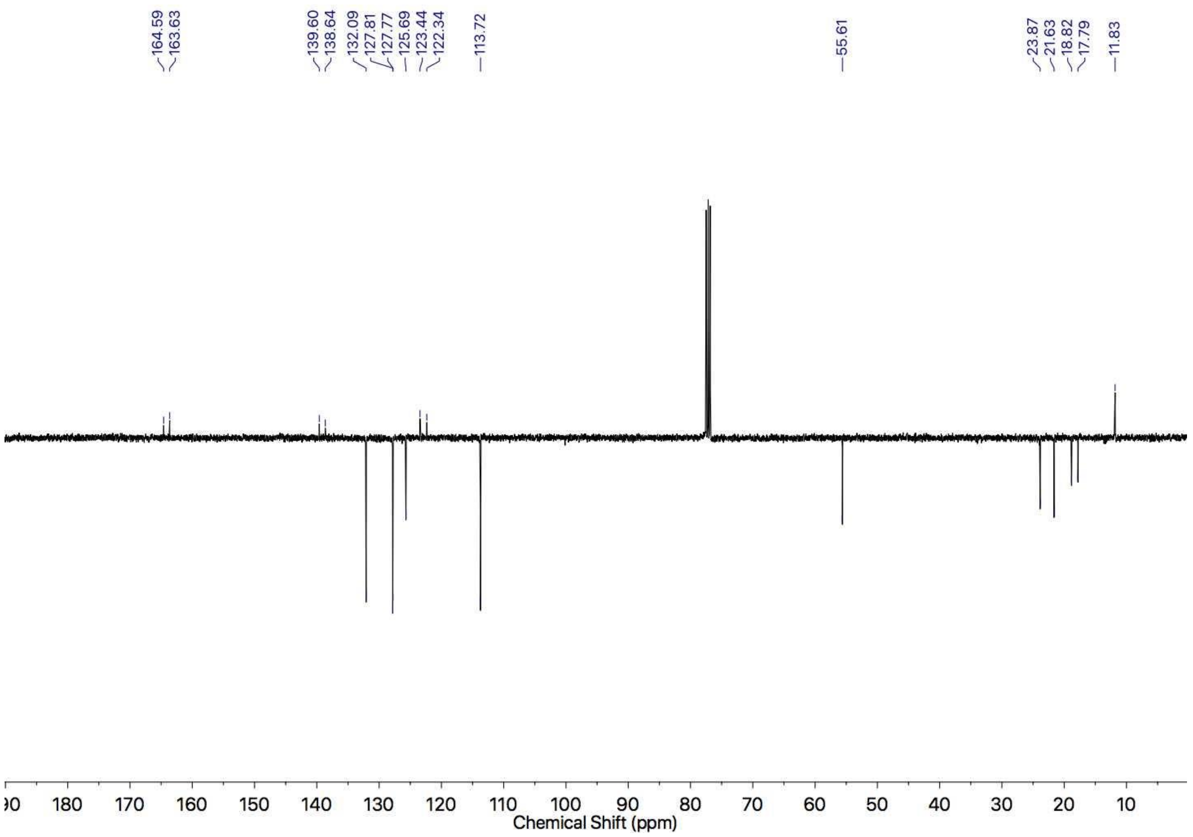

Figure S133: JMOD NMR (101 MHz, $CDCl_3$) of *cis*-**16**.

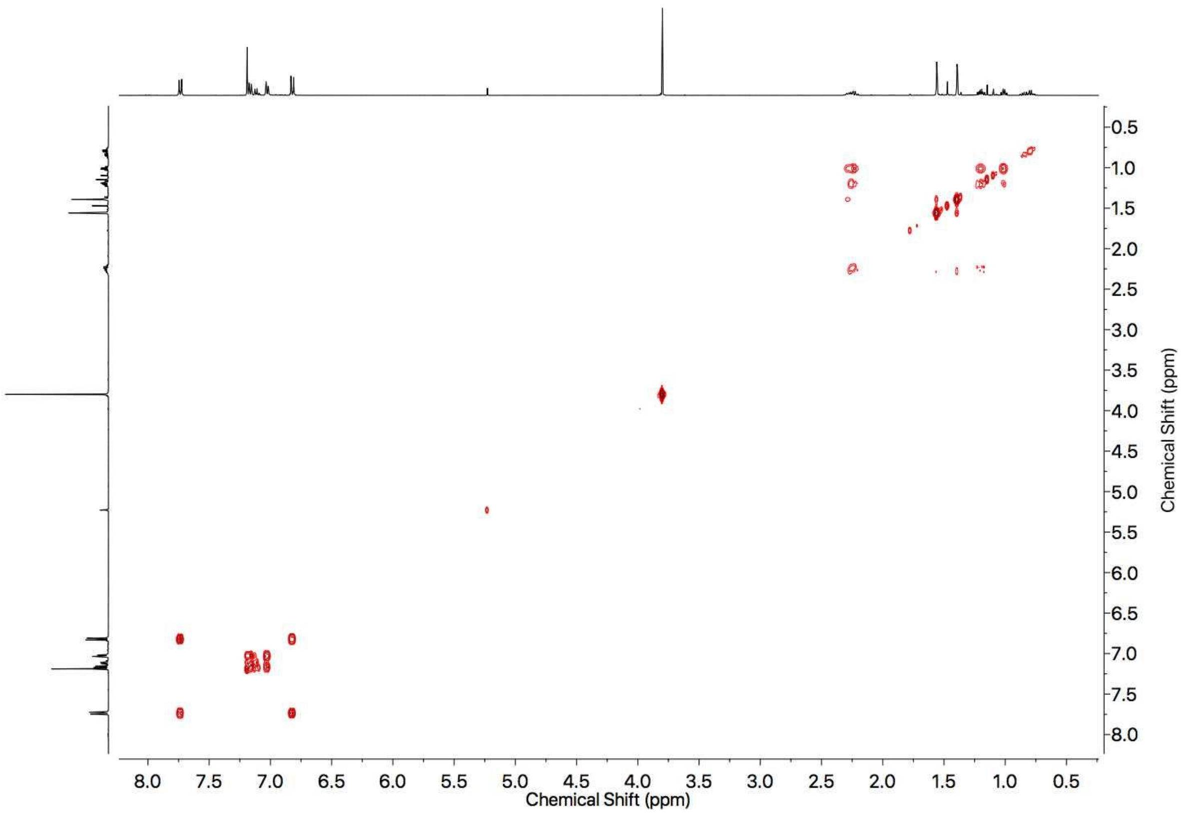

Figure S134: COSY NMR ($CDCl_3$) of *cis*-**16**.

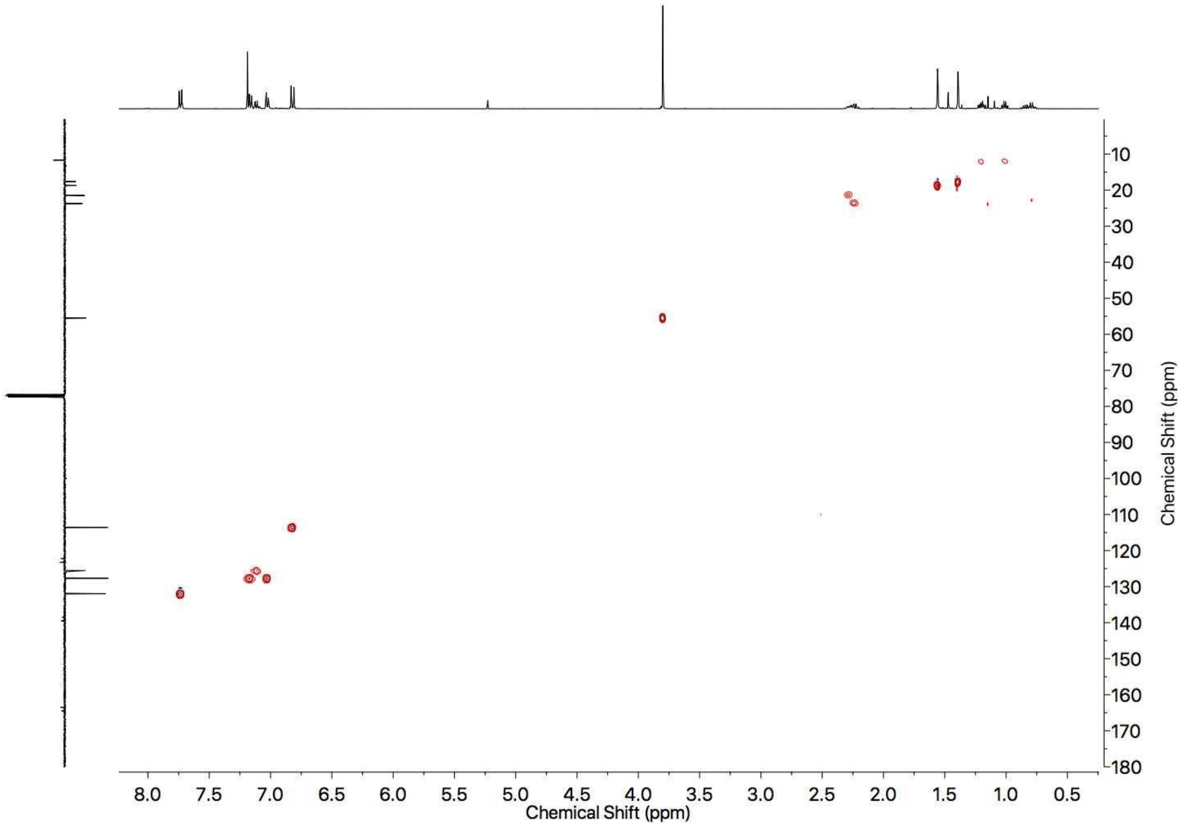

Figure S135: HSQC NMR ($CDCl_3$) of *cis*-**16**.

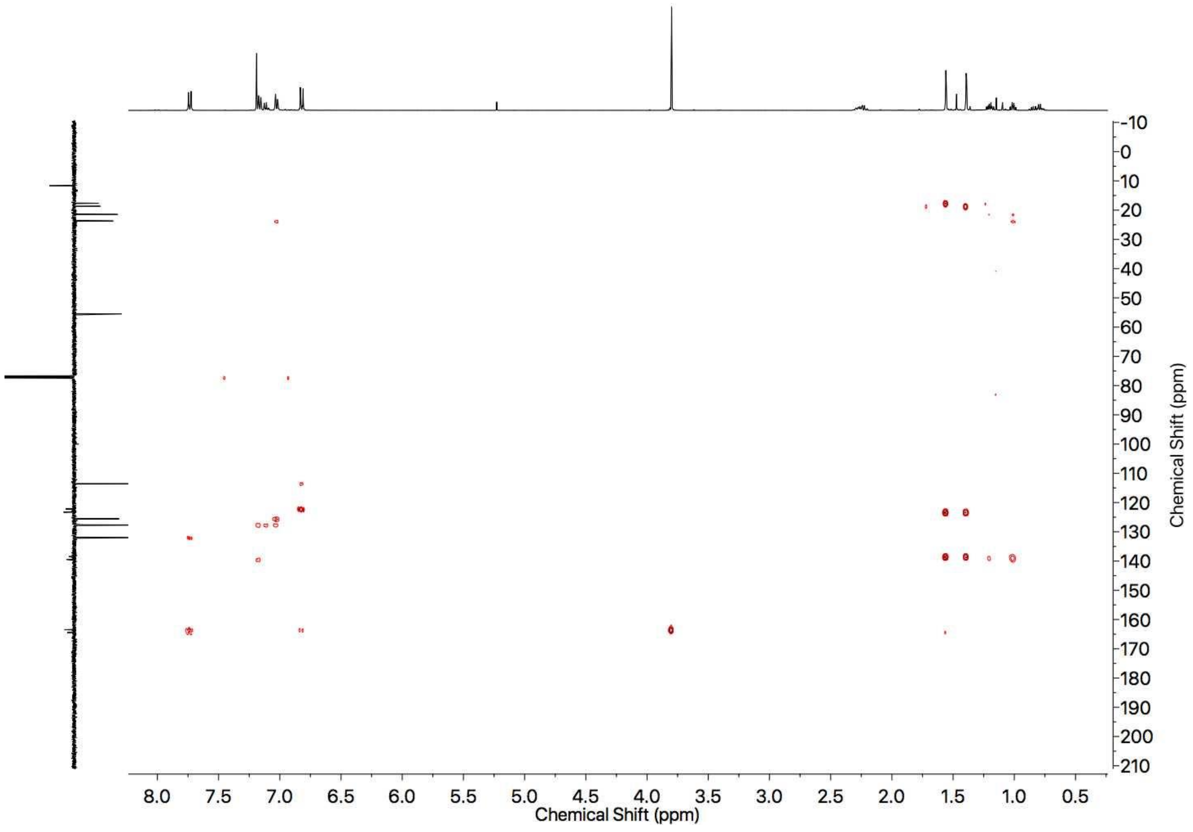

Figure S136: HMBC NMR ($CDCl_3$) of *cis*-**16**.

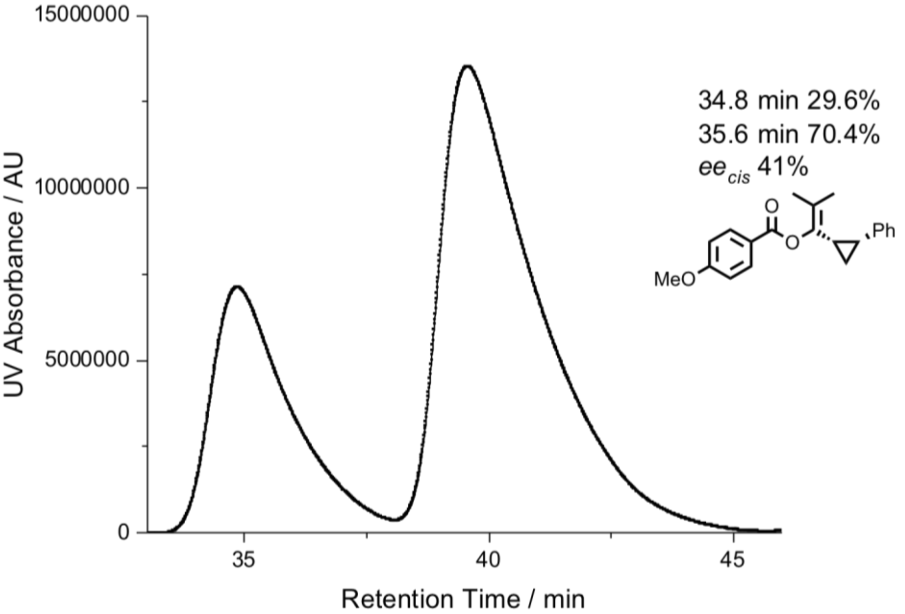

Figure S137: Chiral Stationary Phase HPLC (RegisPack, *n*-hexane-isopropanol 99 : 1, 303 K, load Et_2O, flowrate 0.25 $mLmin^{-1}$) of 70 : 30 *er cis*-**16**. Retention times (min): 34.8, 35.6. The absolute stereochemistry of the major product was determined to be (1*S*,2*R*)-**16** by reduction with $LiAlH_4$ and comparison with the product of the same reaction with cyclopropane **9** (see **Figure S168**).

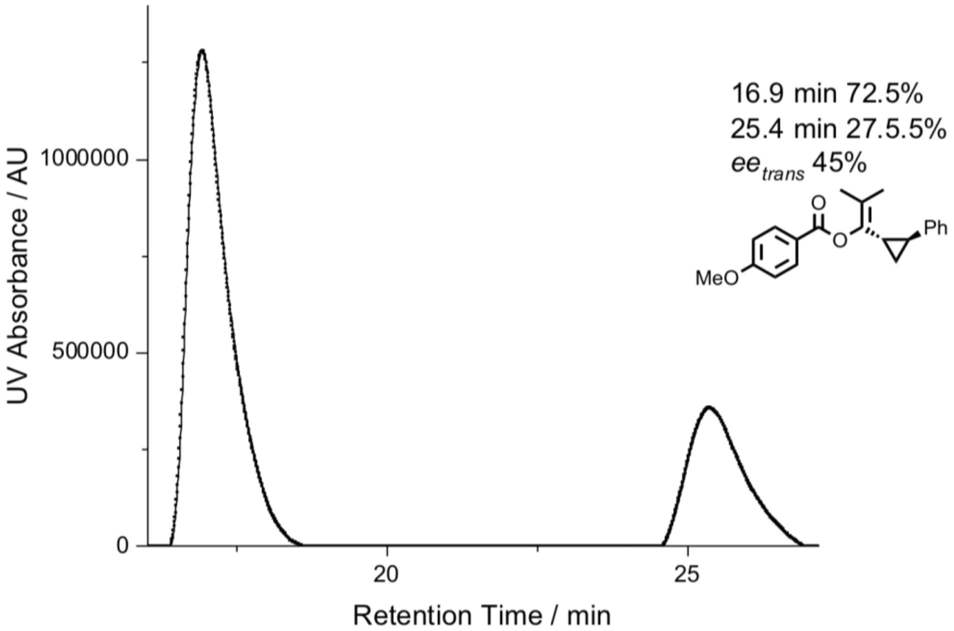

Figure S138: Chiral Stationary Phase HPLC (RegisPack, *n*-hexane-isopropanol 99 : 1, 303 K, load Et_2O, flowrate 0.25 $mLmin^{-1}$) of 72.5 : 27.5 *er trans*-**16**. Retention times (min): 16.9, 25.4. The absolute stereochemistry of the products was not determined. The (1*S*,2*S*)-**16** isomer is shown for illustrative purposes only.

Cyclopropanes **17**

**Catalyst**	**Yield / %**	***dr***	***er*$_{cis}$**	***er*$_{trans}$**
$(Ph_3P)AuCl$	87	95 : 5	1 : 1	1 : 1
[Au((*R*$_{mp}$)-**6**)(Cl)]	79	95 : 5	87 : 13[c]	65 : 35

Table S12. Summary of reactions leading to cyclopropanes **17**.

cis-**17**

Colourless oil (*dr cis-trans* 95 : 5, NMR yield 71 mg, 0.202 mmol, 87%)

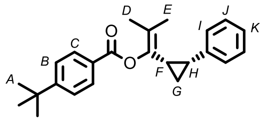

δ$_H$ ($CDCl_3$, 400 MHz) 7.84 (2H, dt, *J* = 9.2, 1.8, ***H***$_C$), 7.45 (2H, dt, *J* = 9.1, 1.8, ***H***$_B$), 7.26 (2H, tt, *J* = 7.1, 1.5, ***H***$_J$), 7.19 (1H, tt, *J* = 7.2, 1.3, ***H***$_K$), 7.12 (2H, dd, *J* = 7.5, 1.7, ***H***$_I$), 2.42-2.28 (2H, m, ***H***$_F$, ***H***$_G$), 1.62 (3H, s, ***H***$_E$), 1.47 (3H, s, ***H***$_D$), 1.36 (9H, s, ***H***$_A$), 1.30-1.24 (1H, m, ***H***$_H$), 1.10 (1H, q, *J* = 5.4, ***H***$_{H'}$).

δ$_C$ ($CDCl_3$, 101 MHz) 164.8, 156.9, 139.5, 138.5, 129.9, 127.8, 127.7, 127.2, 125.7, 125.5, 123.5, 35.2, 31.3, 24.0, 21.7, 18.8, 17.7, 11.7.

HR-EI-MS m/z 348.2081 [$M^{+\cdot}$] (calc. m/z for $C_{24}H_{28}O_2$ 348.2084).

Trans-**17**

Colourless oil (*dr cis-trans* 95 : 5, NMR yield 71mg, 0.202 mmol, 87%)

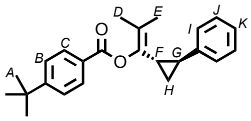

δ$_H$ ($CDCl_3$, 400 MHz) 7.84 (2H, dt, *J* = 8.9, 2.0, ***H***$_C$), 7.50 (2H, dt, *J* = 8.8, 2.0, ***H***$_B$), 7.25 (2H, tt, *J* = 7.4, 1.2, ***H***$_J$), 7.15 (1H, tt, *J* = 7.3, 1.9, ***H***$_K$), 7.09 (2H, dd, *J* = 7.2, 1.5, ***H***$_I$), 2.17-2.06 (2H, m, ***H***$_F$, ***H***$_G$), 1.85 (3H, s, ***H***$_E$) 1.61 (3H, s, ***H***$_D$), 1.36 (9H, s, ***H***$_A$), 1.20 (1H, ddd, *J* = 11.1, 6.0, 5.0, ***H***$_H$), 1.13 (1H, ddd, *J* = 11.1, 5.9, 5.0, ***H***$_{H'}$).

δ$_C$ ($CDCl_3$, 101 MHz) 164.9, 157.2, 142.3, 130.0, 128.5, 127.0, 126.1, 125.9, 125.7, 122.8, 120.9, 35.3, 31.3, 23.8, 23.6, 19.0, 18.3, 14.7.

HR-EI-MS m/z 348.2077 [$M^{+\cdot}$] (calc. m/z for $C_{24}H_{28}O_2$ 348.2089).

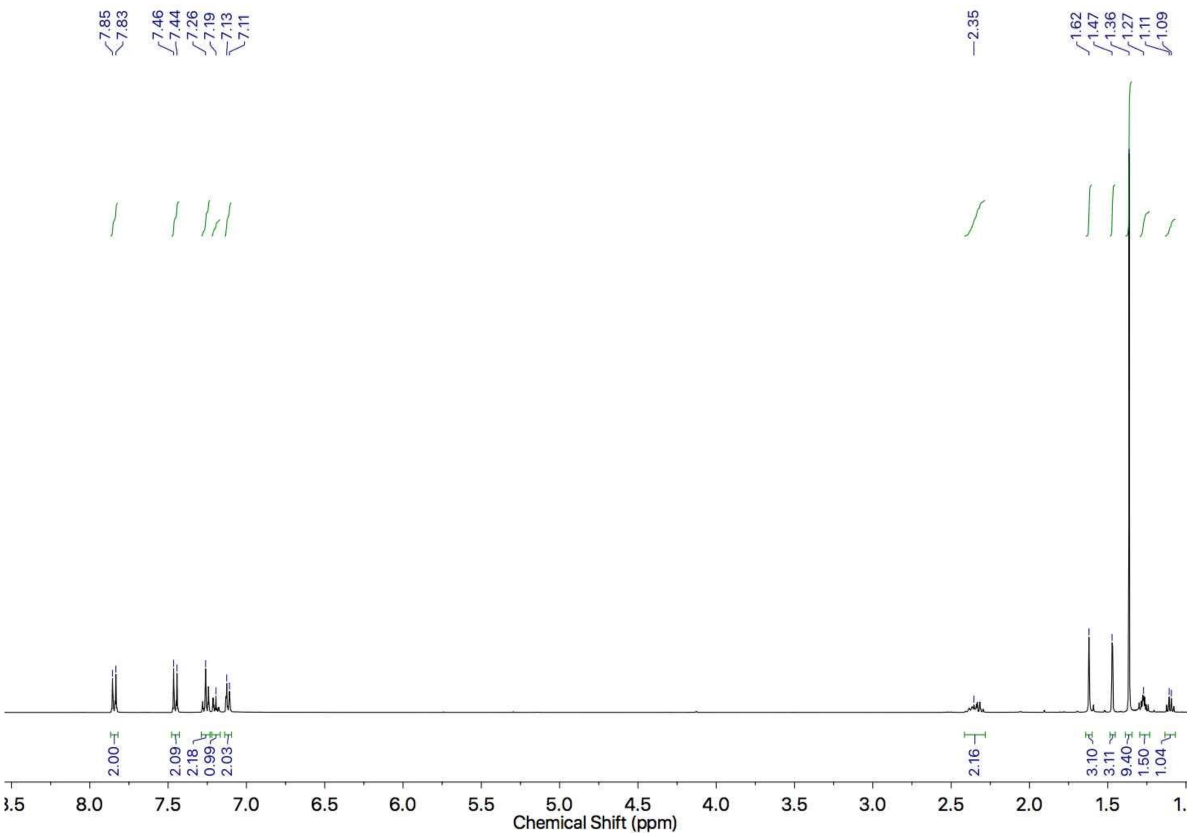

Figure S139: ^{1}H NMR (400 MHz, $CDCl_3$) of *cis*-**17**.

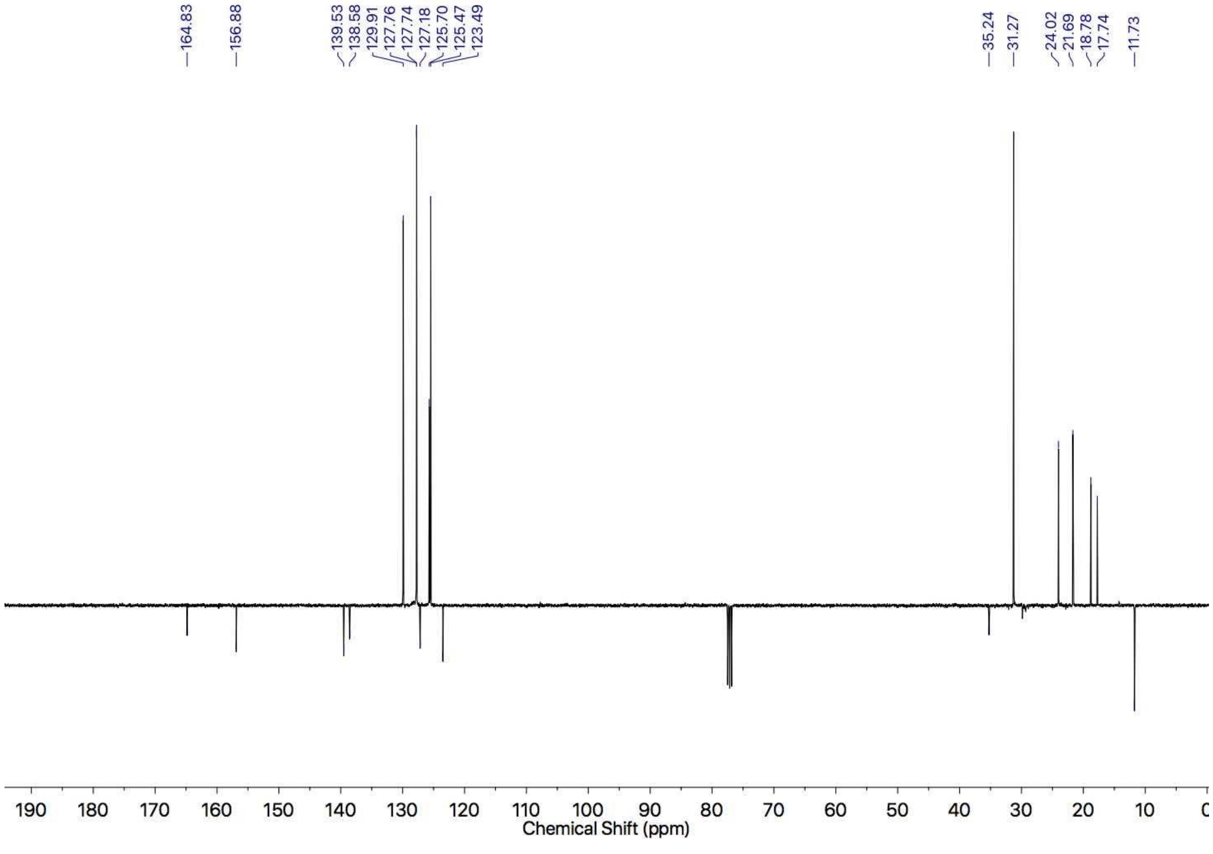

Figure S140: JMOD NMR (101 MHz, $CDCl_3$) of *cis*-**17**.

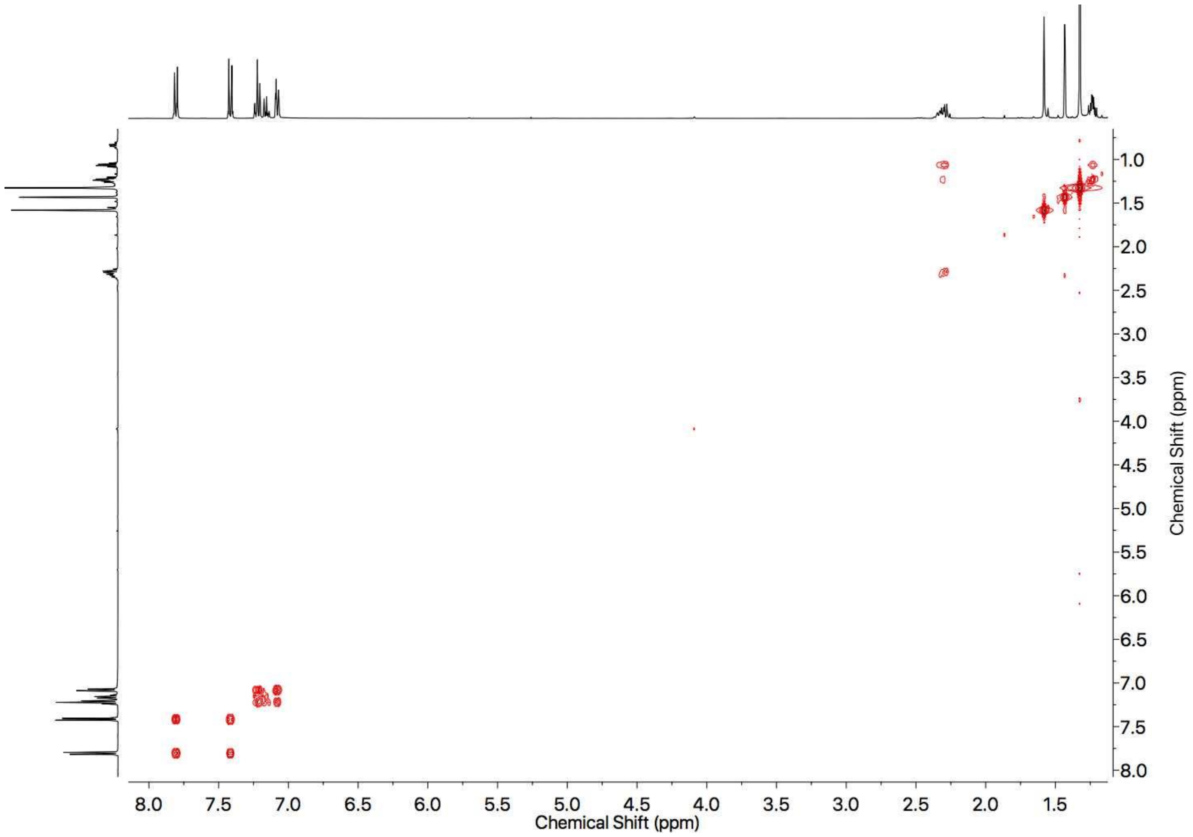

Figure S141: COSY NMR ($CDCl_3$) of *cis*-**17**.

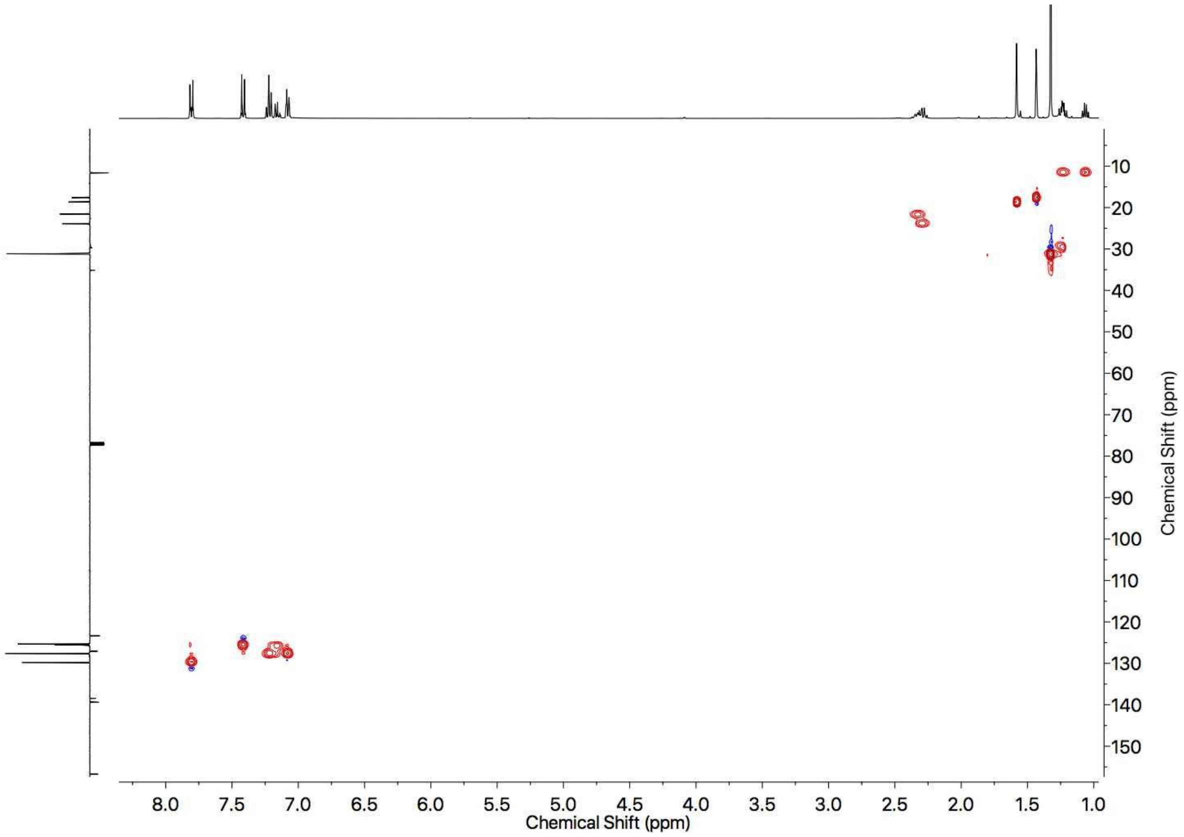

Figure S142: HSQC NMR ($CDCl_3$) of *cis*-**17**.

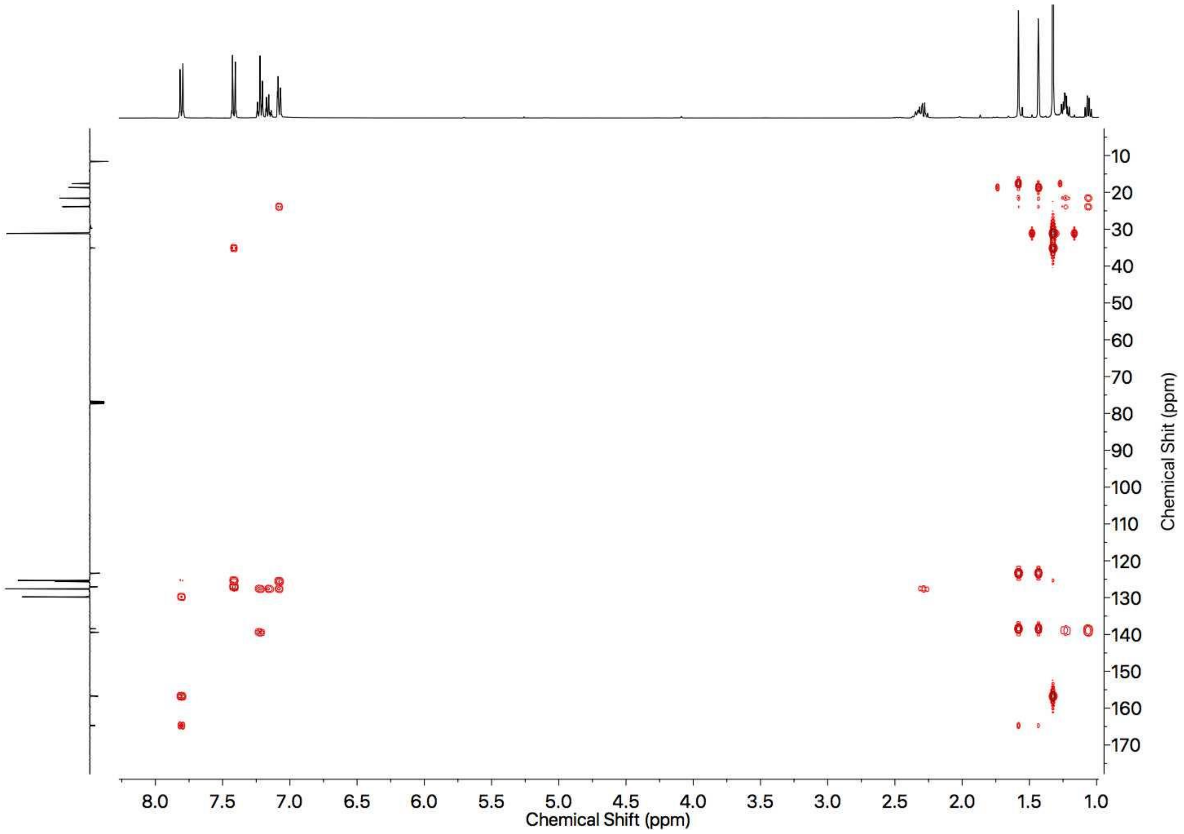

Figure S143: HMBC NMR ($CDCl_3$) of *cis*-**17**.

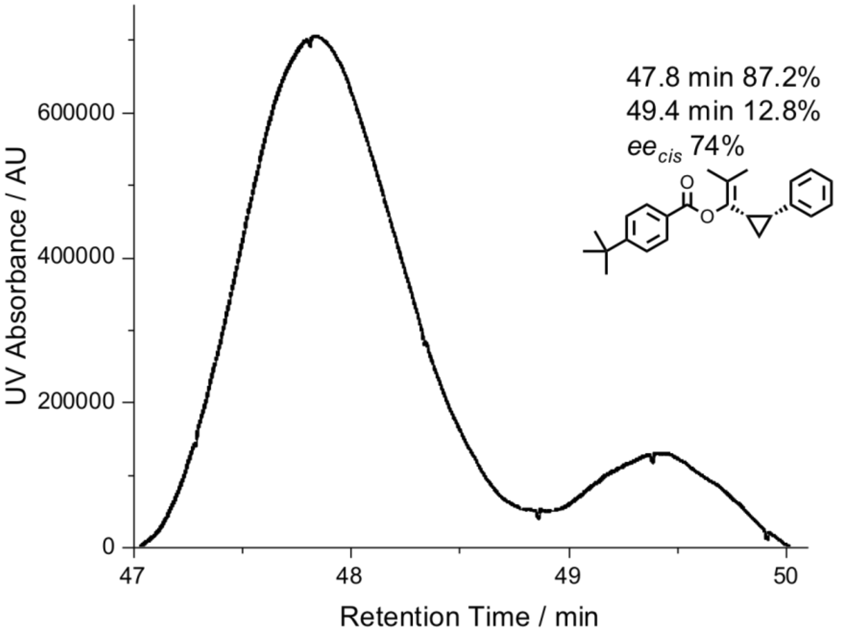

Figure S144: Chiral Stationary Phase HPLC ((S,S)Whelk, *n*-hexane-isopropanol 99.5 : 0.5, 303 K, load Et_2O, flowrate 0.25 $mLmin^{-1}$) of 83 : 17 *er cis*-**S17**. Retention times (min): 47.8, 49.4. The absolute stereochemistry of the products was not determined. The (1*S*,2*R*)-**S17** isomer is shown for illustrative purposes only.

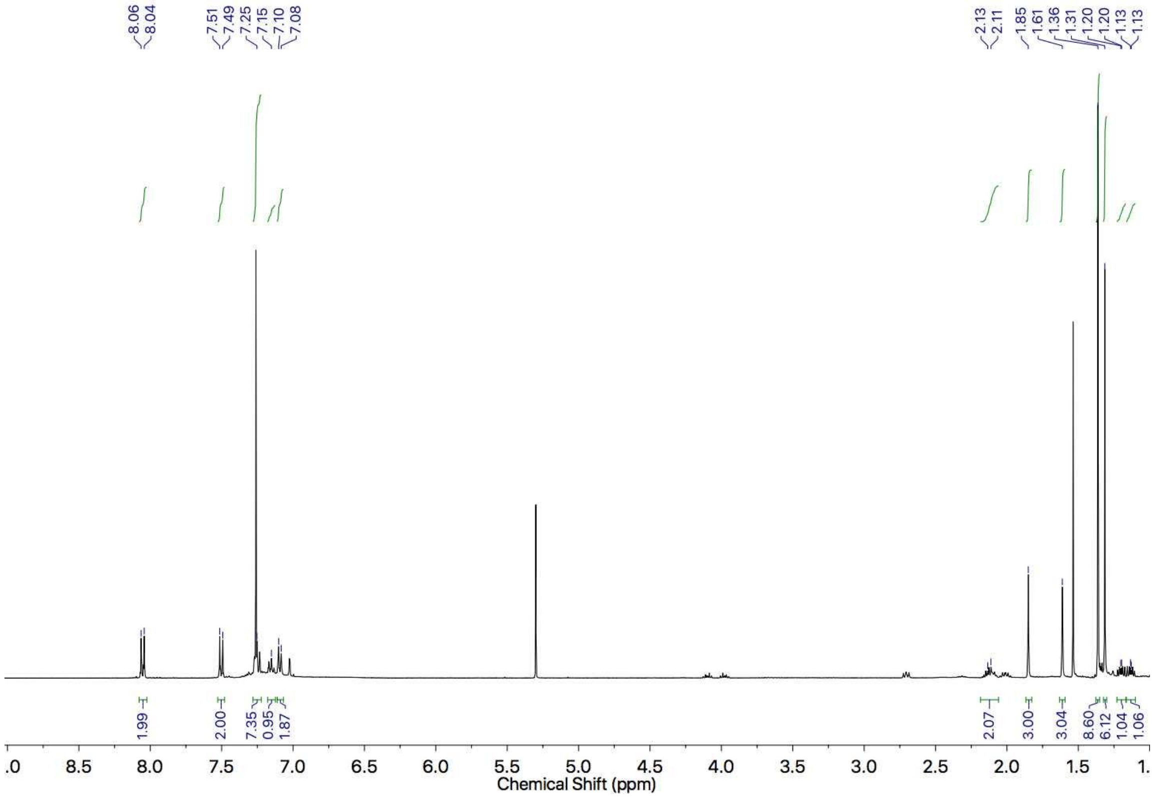

Figure S145: ^{1}H NMR (400 MHz, $CDCl_3$) of *trans*-**17**.

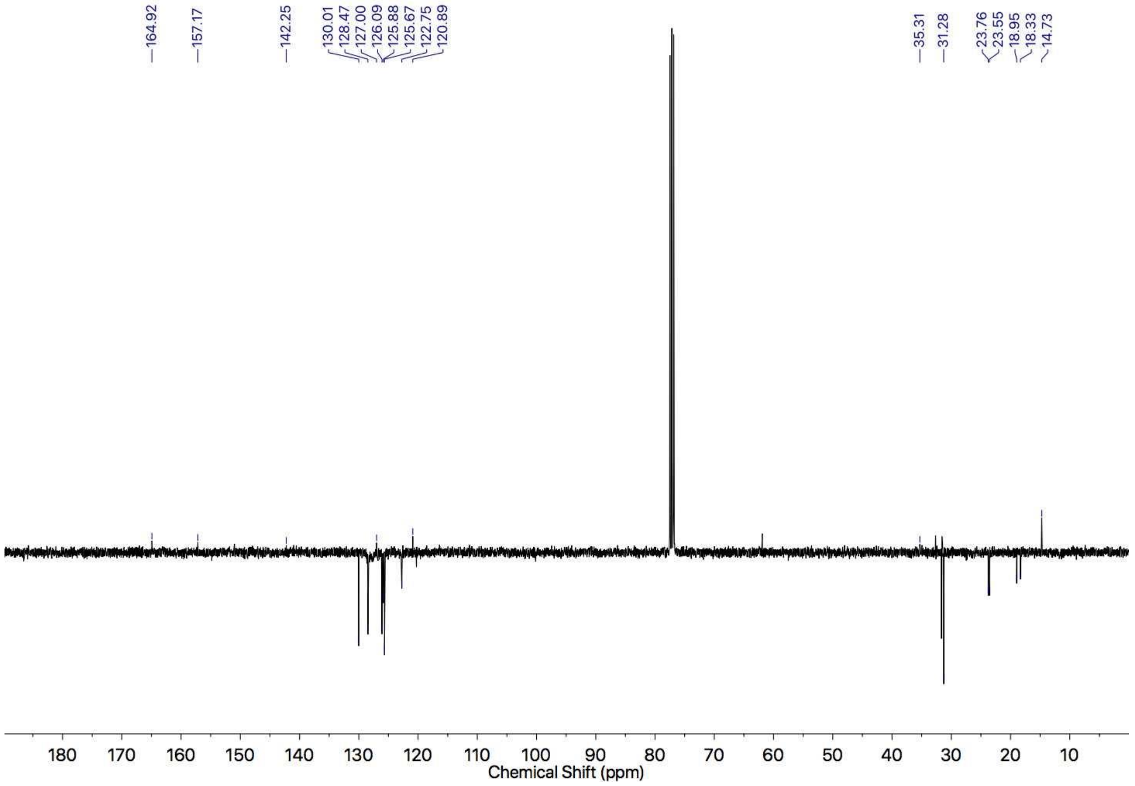

Figure S146: JMOD NMR (101 MHz, $CDCl_3$) of *trans*-**17**.

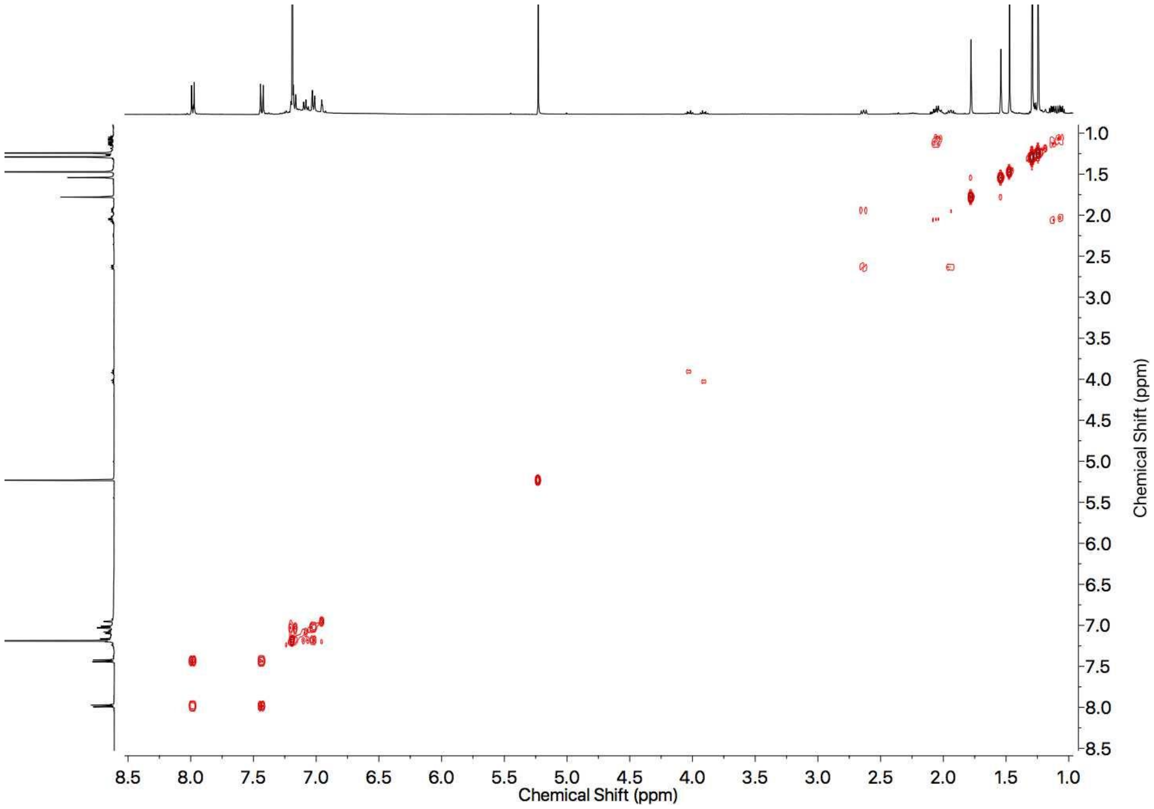

Figure S147: COSY NMR ($CDCl_3$) of *trans*-**17**.

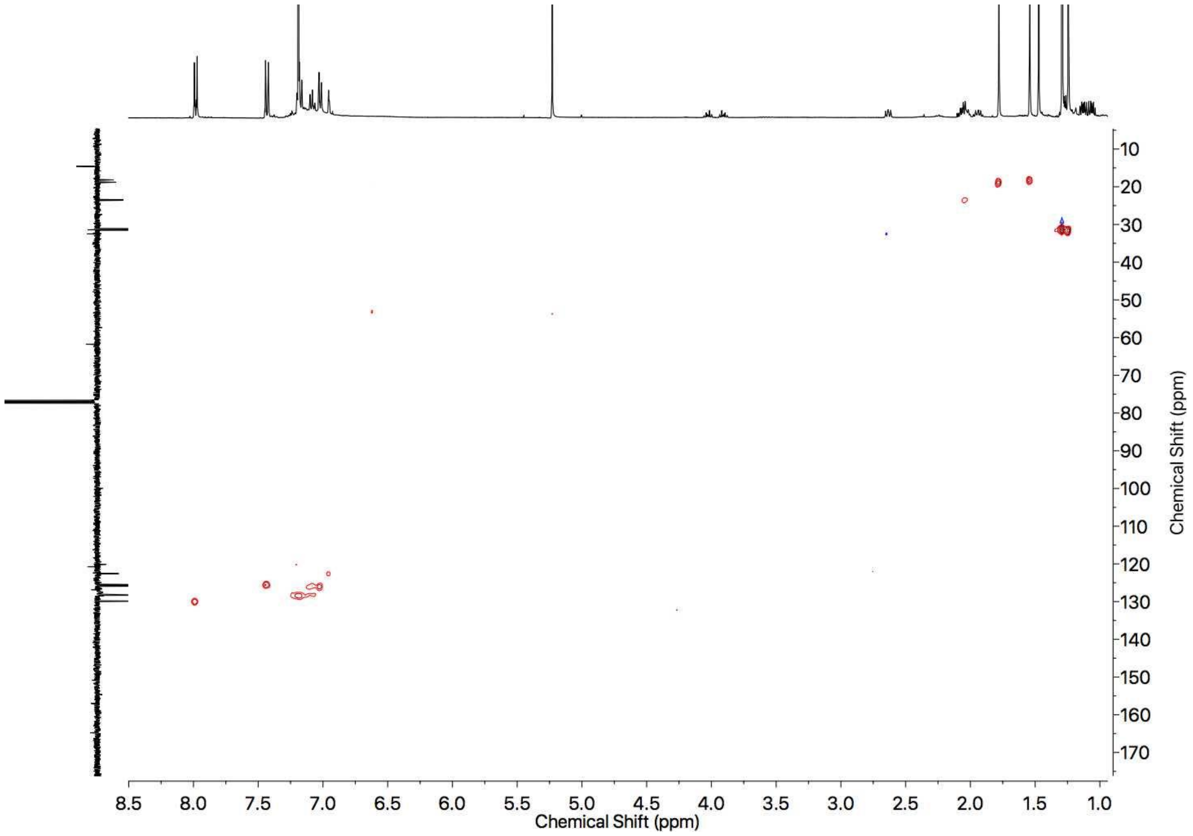

Figure S148: HSQC NMR ($CDCl_3$) of *trans*-**17**.

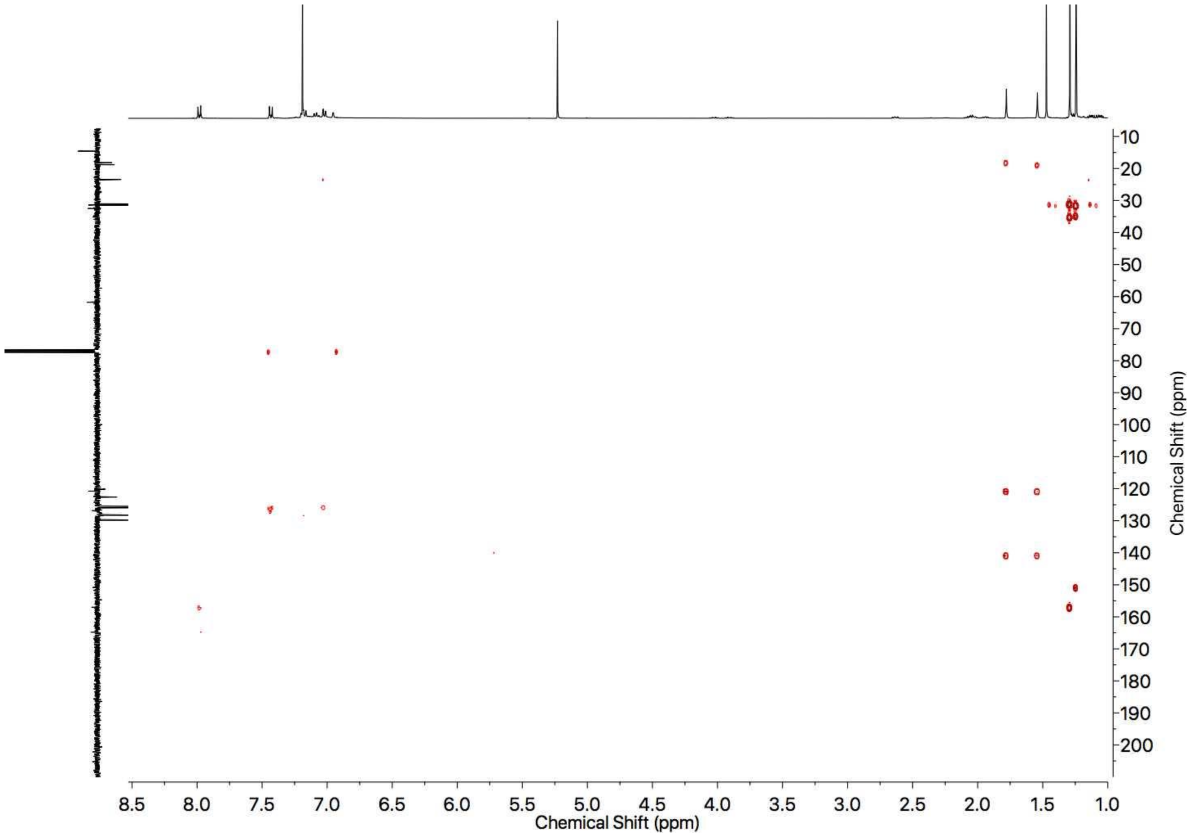

Figure S149: HMBC NMR ($CDCl_3$) of *trans*-**17**.

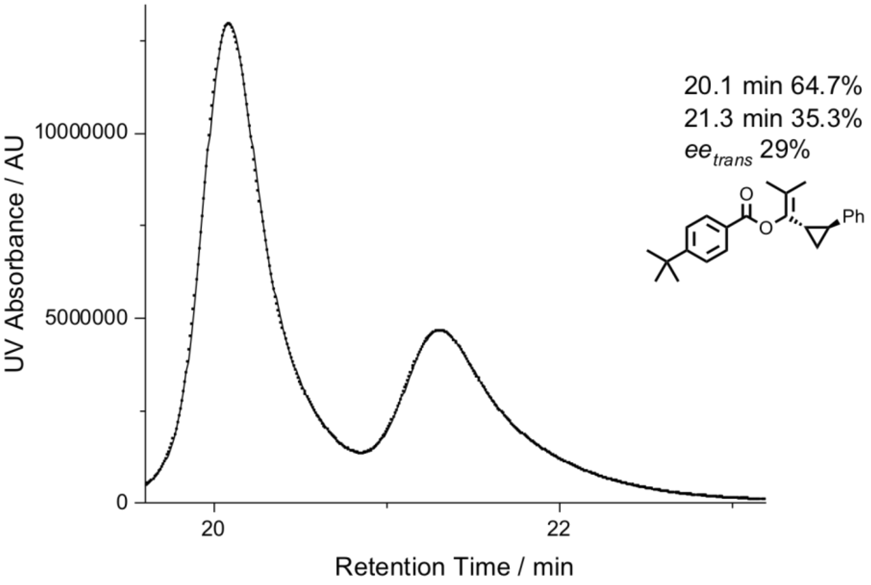

Figure S150: Chiral Stationary Phase HPLC (RegisPack, *n*-hexane-isopropanol 99 : 1, 303 K, load Et_2O, flowrate 0.25 mLmin^{-1}) of 65 : 35 *er trans*-**17**. Retention times (min): 20.1, 21.3. The absolute stereochemistry of the products was not determined. The (1*S*,2*S*)-**17** isomer is shown for illustrative purposes only.

Cyclopropanes **18**

**Catalyst**	**Yield / %**	***dr***	***er*cis**	***er**trans*
(Ph_3P)AuCl[a]	99	95 : 5	1 : 1	1 : 1
[Au((*R*mp)-**6**)(Cl)][b]	68	96 : 4	89 : 11	70 : 30

Table S13. Summary of reactions leading to cyclopropanes **18**.

Cis-**18**

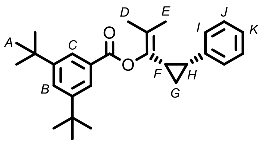

δ_H ($CDCl_3$, 400 MHz) 7.84 (2H, d, *J* = 1.9, ***H***C), 7.66 (1H, t, *J* = 1.9, ***H***B), 7.25 (2H, t, *J* = 7.6, ***H***J), 7.18 (1H, tt, *J* = 7.3, 2.0, ***H***K), 7.15 (2H, d, *J* = 7.1, ***H***I), 2.42-2.29 (2H, m, ***H***F, ***H***G), 1.67 (3H, s, ***H***E), 1.49 (3H, s, ***H***D), 1.37 (18H, s, ***H***A) 1.32-1.26 (1H, m, ***H***H), 1.15 (1H, q, *J* = 6.1, ***H***H).

δ_C ($CDCl_3$, 101 MHz) 165.5, 151.2, 139.5, 138.7, 129.3, 127.8, 127.8, 127.5, 125.8, 124.2, 123.3, 35.1, 31.5, 23.9, 21.8, 18.8, 17.9, 11.8.

HR-EI-MS m/z 404.2703 [$MS^{+\cdot}$] (calc. m/z for $C_{28}H_{36}O_2$ 404.2710).

Trans-**18**

Colourless oil (*dr cis-trans* 95 : 5, NMR yield 94 mg, 0.232 mmol, 99%)

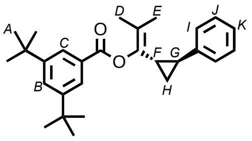

δ_H ($CDCl_3$, 400 MHz) 7.97 (2H, d, *J* = 1.9, ***H***C), 7.68 (1H, t, *J* = 1.9, ***H***B), 7.26 (2H, tt, *J* = 7.5, 1.3, ***H***J), 7.15 (1H, tt, *J* = 7.4, 1.9, ***H***K), 7.10 (2H, dd, *J* = 7.4, 1.5, ***H***I), 2.19-2.07 (2H, m, ***H***F, ***H***G), 1.86 (3H, s, ***H***E), 1.63 (3H, s, ***H***D), 1.38 (18H, s, ***H***A), 1.23 (1H, ddd, *J* = 11.1, 5.1, 6.0, ***H***H), 1.15 (1H, ddd, *J* = 11.0, 5.7, 5.1, ***H***H').

δ_C ($CDCl_3$, 101 MHz) 165.6, 151.4, 142.0, 141.0, 129.1, 128.5, 127.7, 126.1, 125.9, 124.3, 120.9, 35.1, 31.5, 23.8, 23.7, 19.0, 18.4, 14.8.

HR-EI-MS m/z 404.2711 [$M^{+\cdot}$] (calc. m/z for $C_{28}H_{36}O_2$ 404.2710).

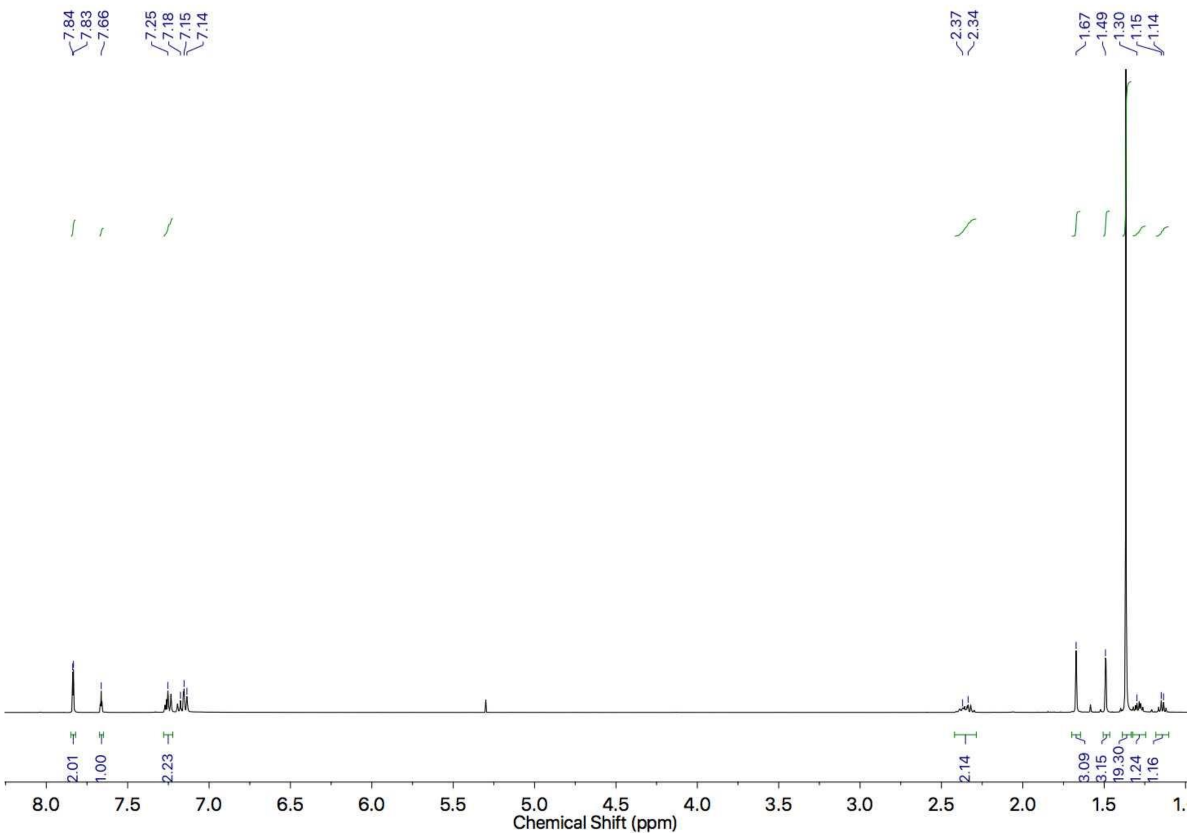

Figure S151: ^{1}H NMR (400 MHz, $CDCl_3$) of *cis*-**18**.

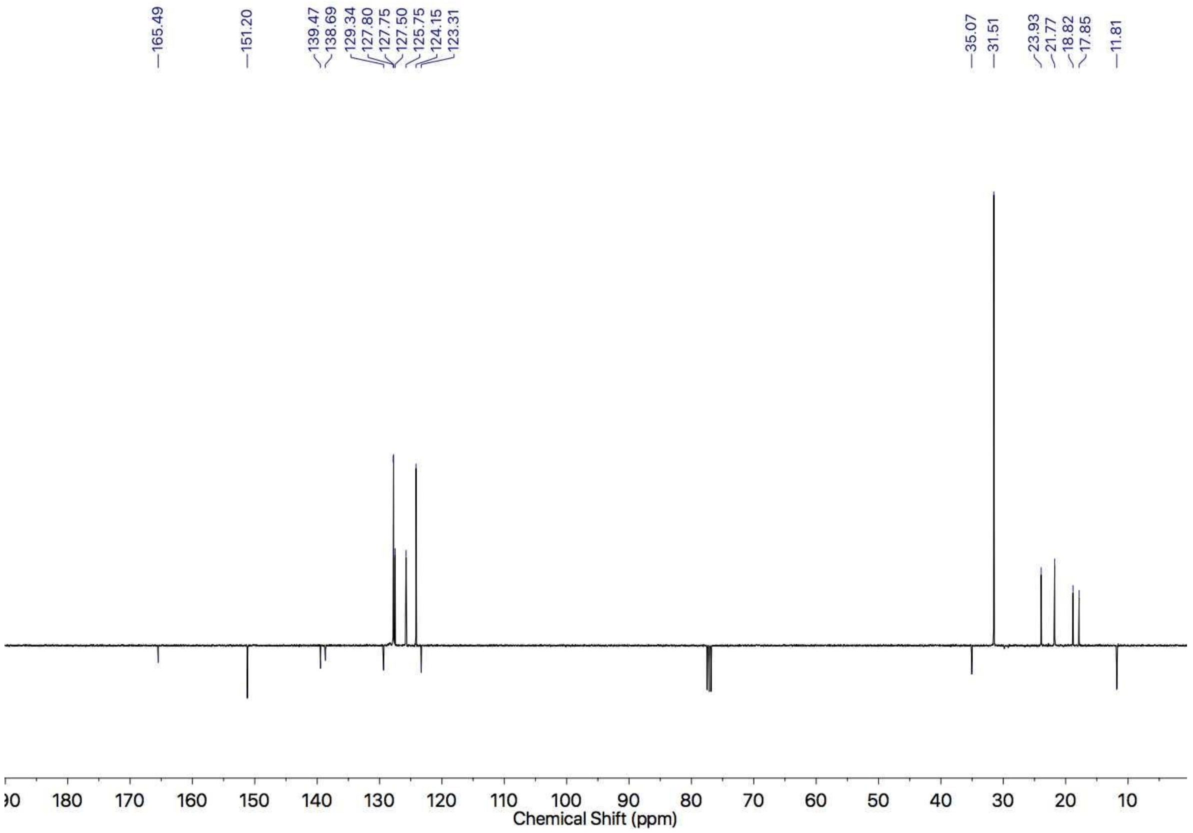

Figure S152: JMOD NMR (101 MHz, $CDCl_3$) of *cis*-**18**.

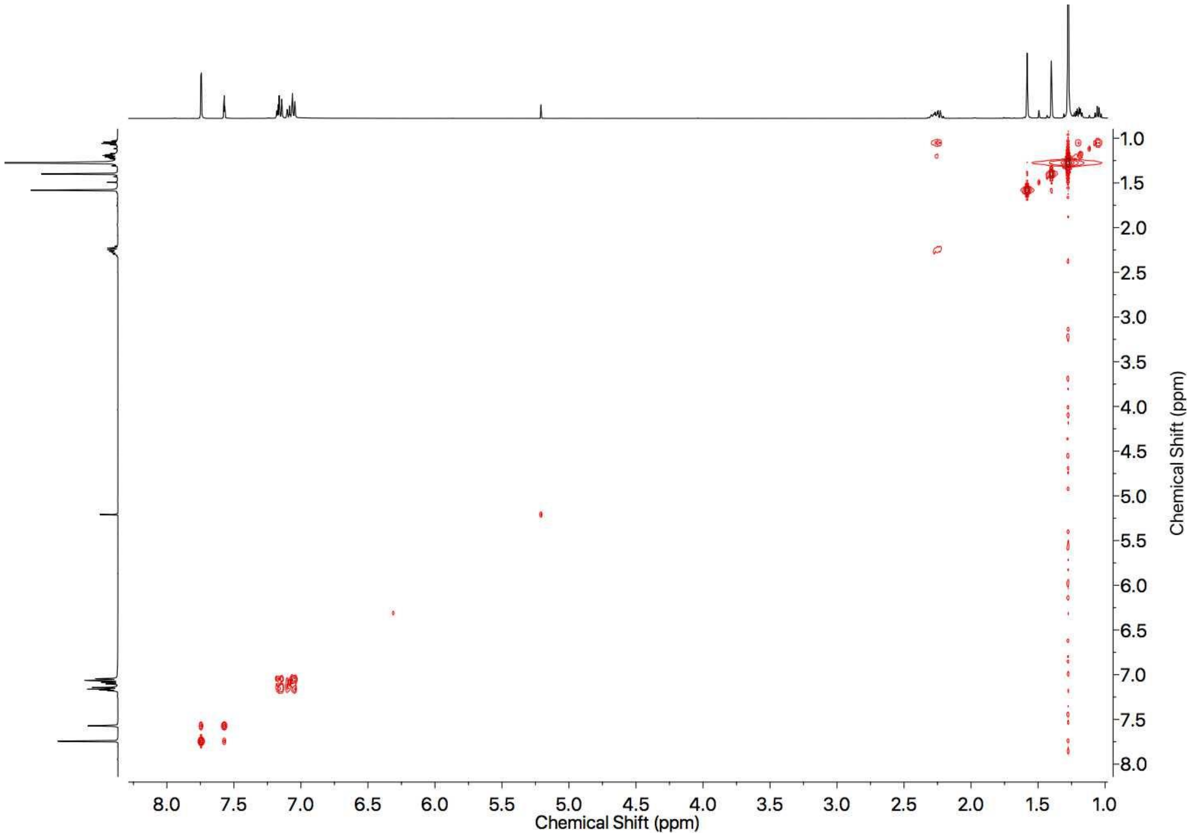

Figure S153: COSY NMR ($CDCl_3$) of *cis*-**18**.

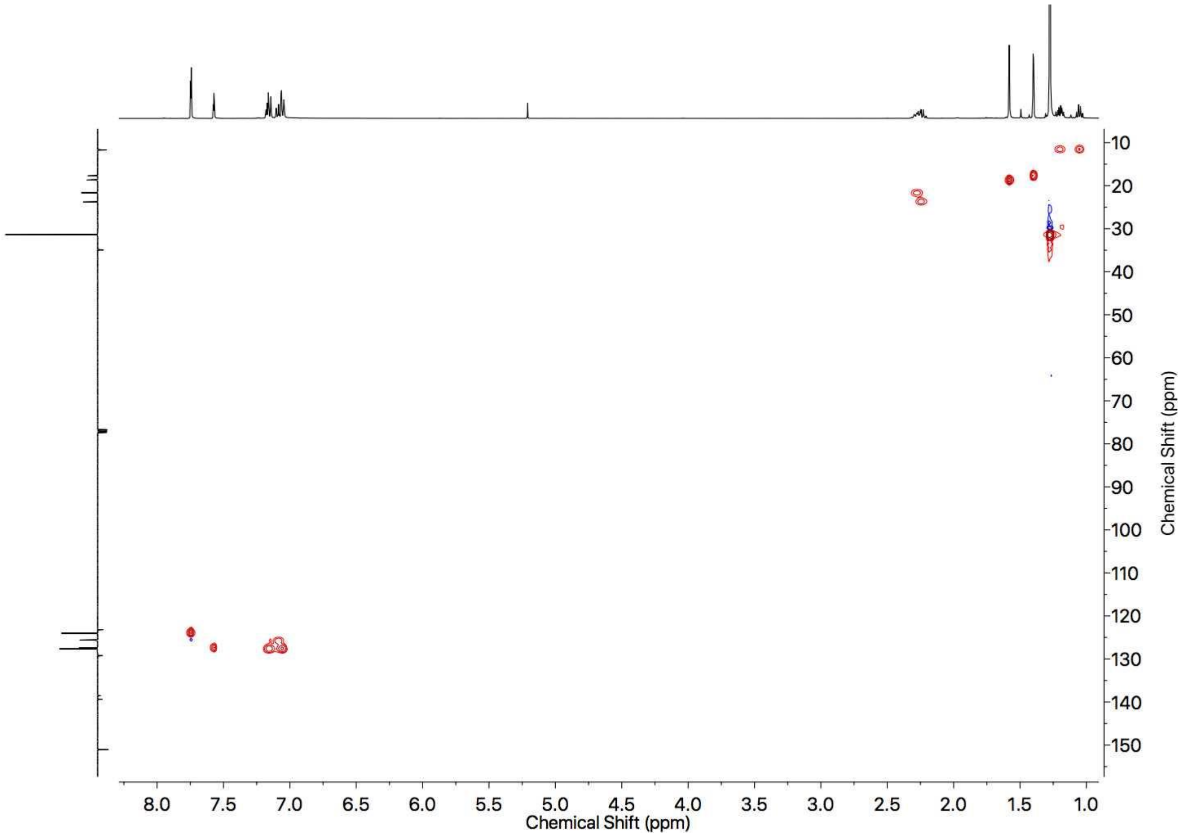

Figure S154: HSQC NMR ($CDCl_3$) of *cis*-**18**.

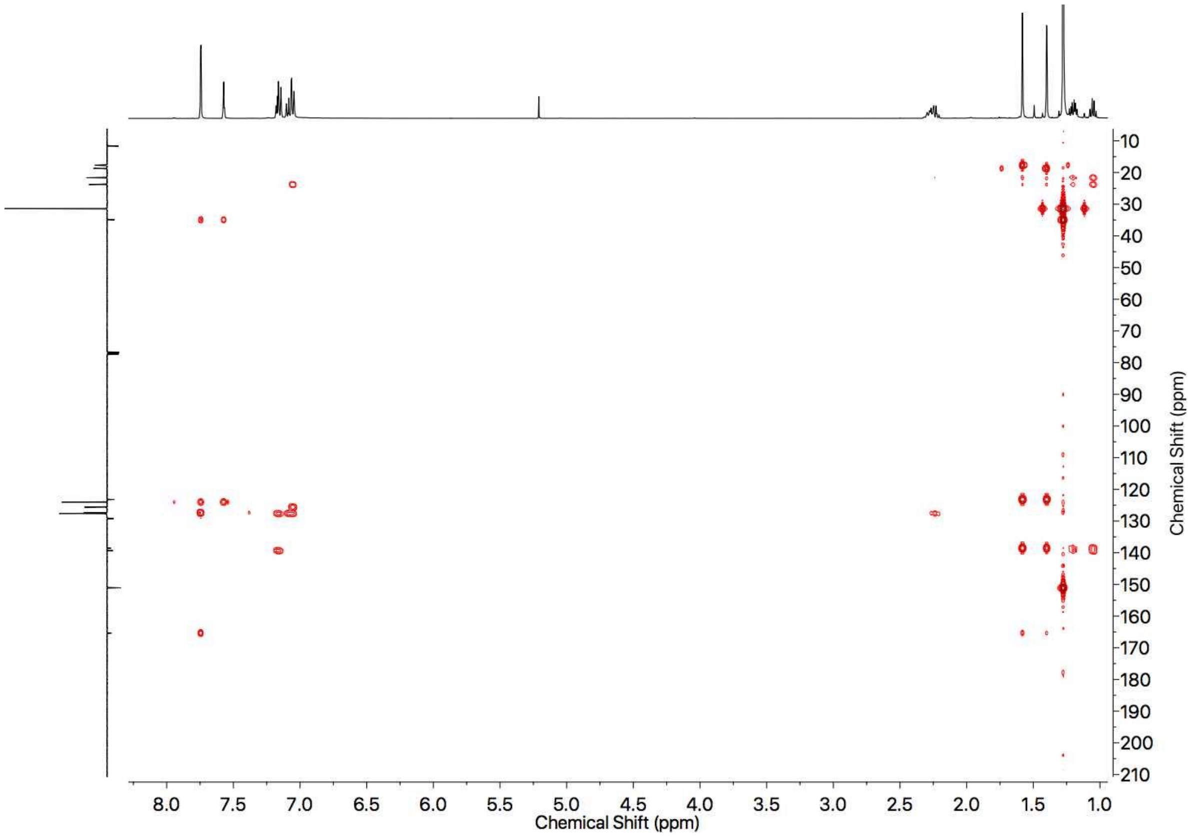

Figure S155: HMBC NMR ($CDCl_3$) of *cis*-**18**.

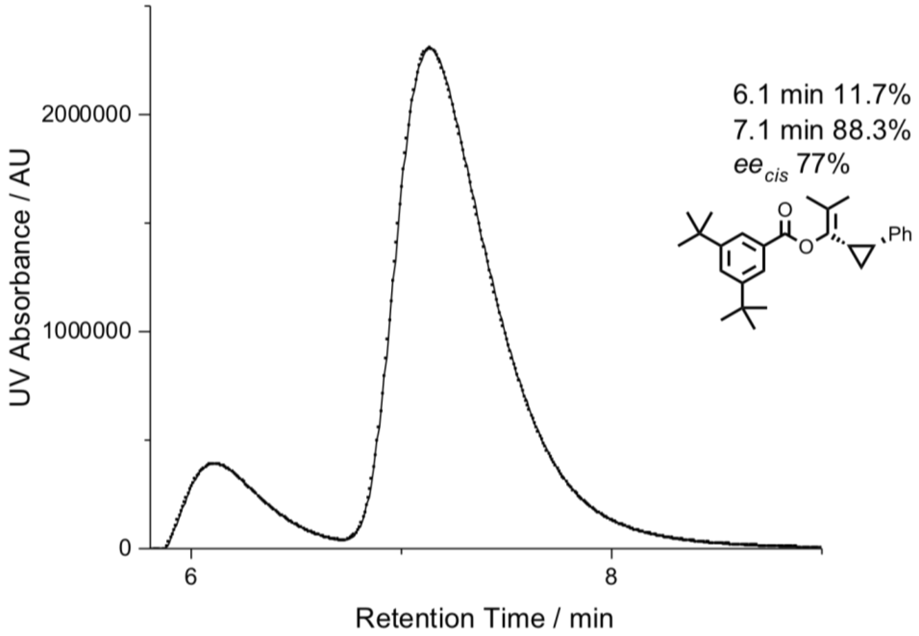

Figure S156: Chiral Stationary Phase HPLC (RegisPack, *n*-hexane-isopropanol 99.8 : 0.2, 303 K, load Et_2O, flowrate 0.75 mLmin^{-1}) of 88 : 12 *er cis*-**18**. Retention times (min): 6.1, 7.1. The absolute stereochemistry of the products was not determined. The (1*S*,2*R*)-**18** isomer is shown for illustrative purposes only.

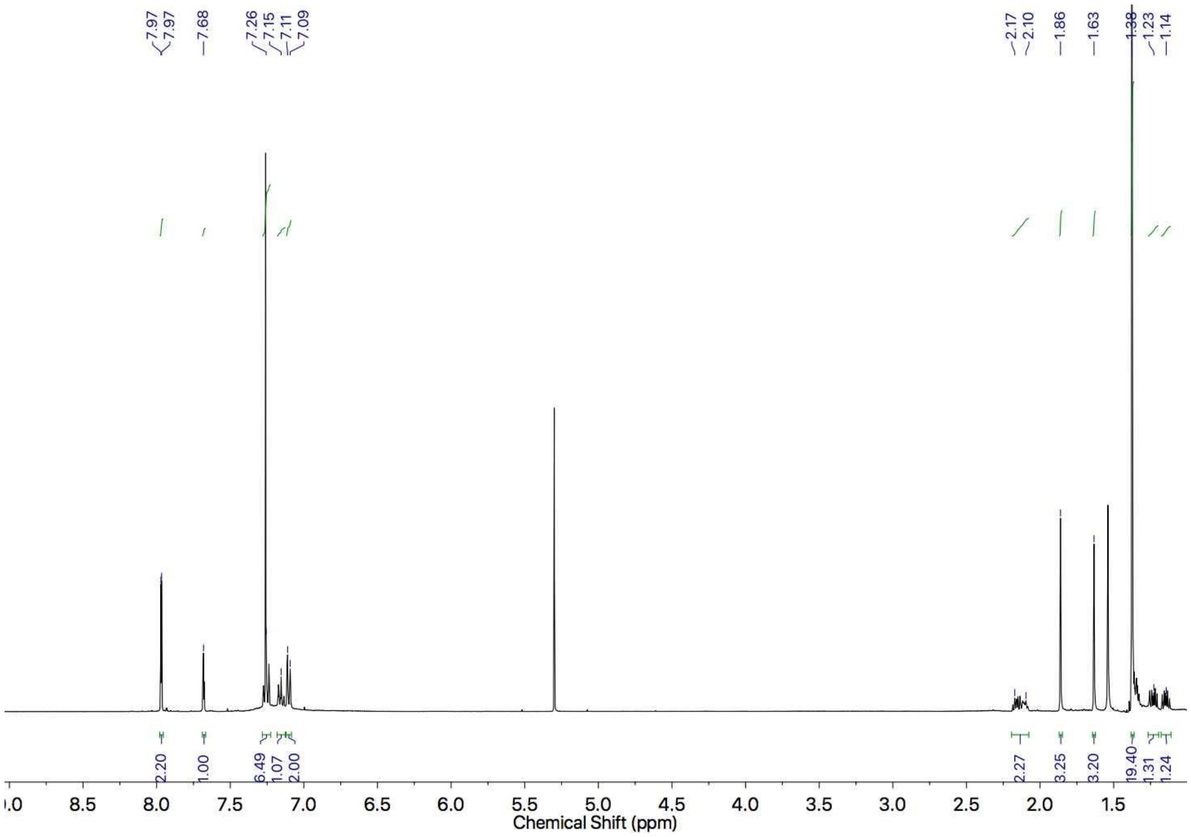

Figure S157: ^{1}H NMR (400 MHz, $CDCl_3$) of *trans*-**18**.

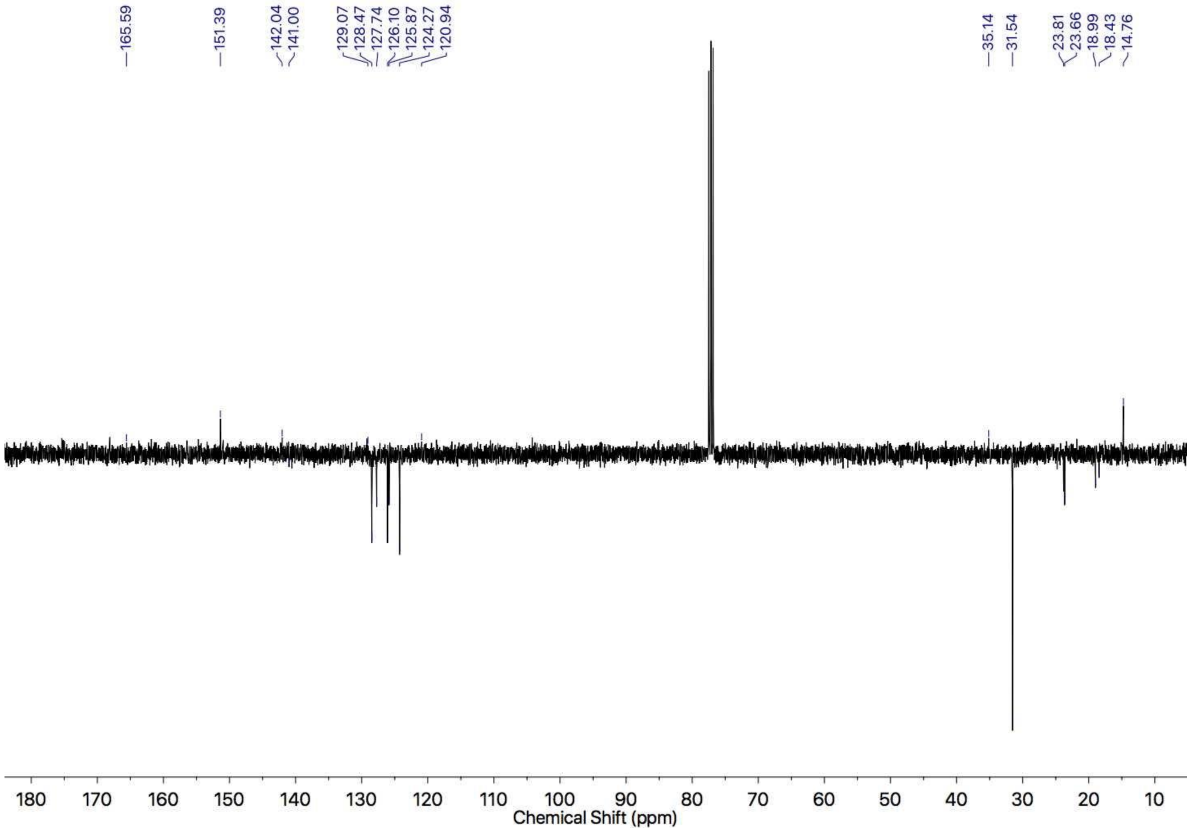

Figure S158: JMOD NMR (101 MHz, $CDCl_3$) of *trans*-**18**.

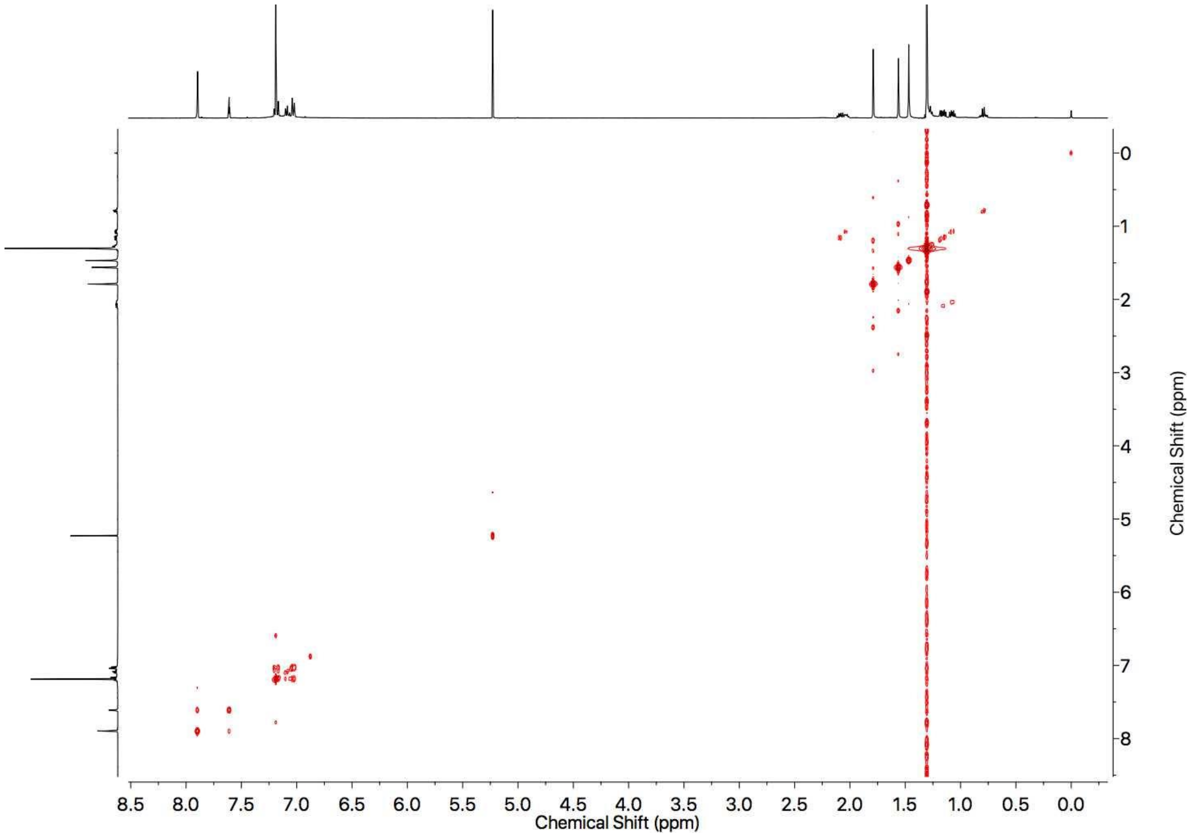

Figure S159: COSY NMR ($CDCl_3$) of *trans*-**18**.

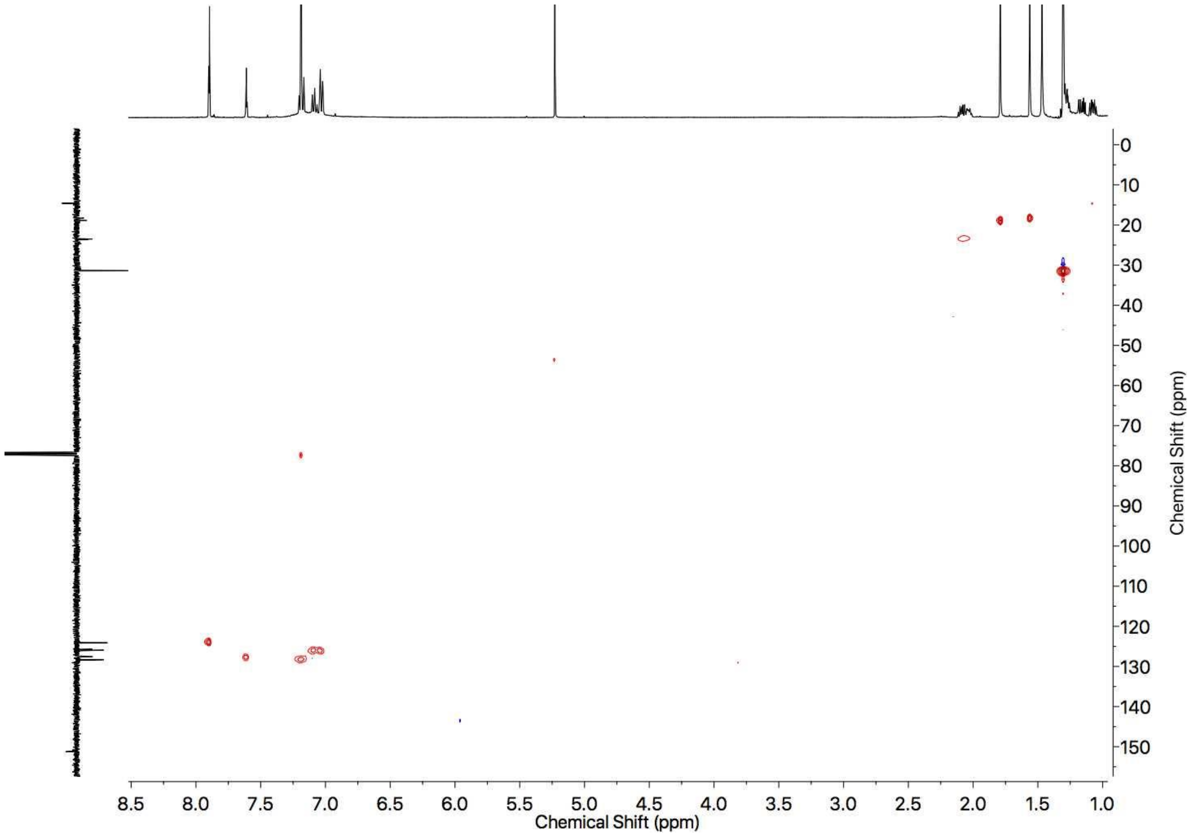

Figure S160: HSQC NMR ($CDCl_3$) of *trans*-**18**.

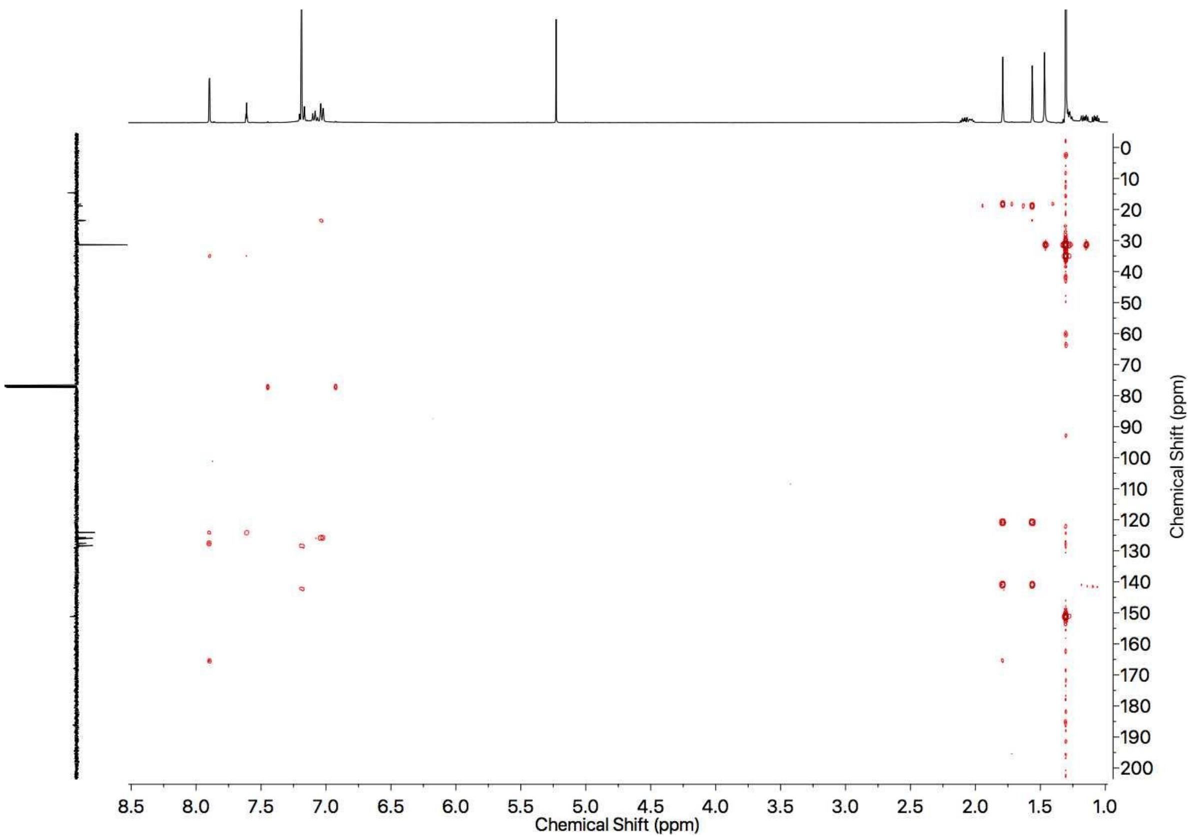

Figure S161: HMBC NMR ($CDCl_3$) of *trans*-**18**.

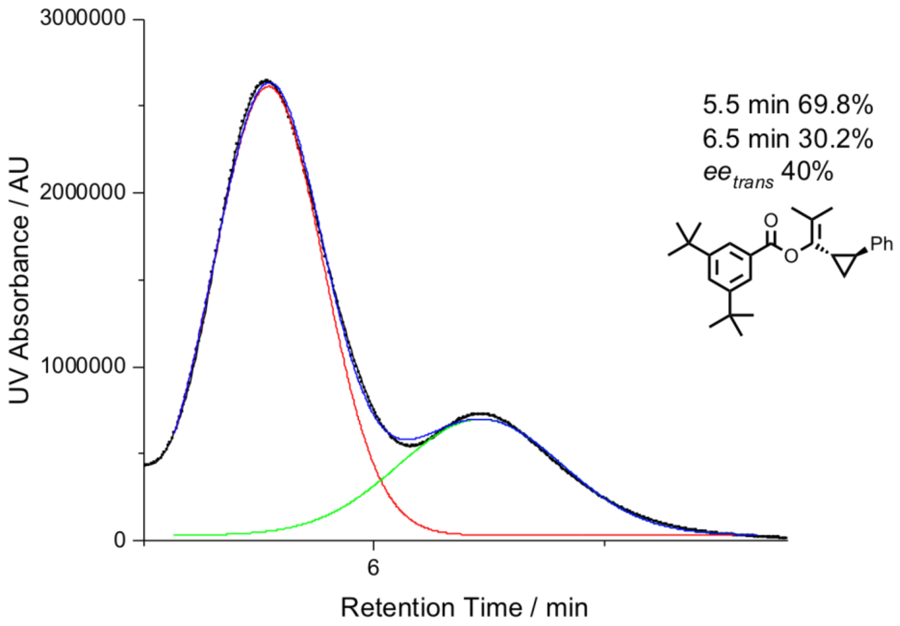

Figure S162: Chiral stationary phase HPLC (RegisPack, hexane-isopropanol 99.8 : 0.2, 303 K, load Et_2O, flowrate 0.75 $mLmin^{-1}$) of 70 : 30 *er trans*-**18**. Retention times (min): 5.5, 6.5. The absolute stereochemistry of the products was not determined. The (1*S*,2*S*)-**18** isomer is shown for illustrative purposes only.

The absolute configuration of *cis*-**9** was determined by comparison to known selectivity of (*R*)-DTBM-SEGPHOS$(AuCl)_2$ by Toste *et al.* *cis*-**9**, *cis*-**13**, *cis*-**15**, *cis*-**16** were converted to ketone **S9** as per the approach to Toste *et al.*[6] Converging HPLC traces demonstrate the matching absolute stereochemistry for (1*S*,2*R*)-**9**, (1*S*,2*R*)-**15**, and (1*S*,2*R*)-**16**.

cis-**S9**

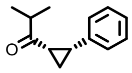

$LiAlH_4$ (50 µL, 50 µmol, 2 eq., 1 M) was added to a solution of *cis*-**17** (8.7 mg, 25 µmol, 1 eq.) in anhydrous THF (0.2 mL) at 0 °C, then stirred at room temperature for 2 h. The reaction was quenched with MeOH, washed with 5% w/v $LiCl_{aq}$ (15 mL) and extracted with CH_2Cl_2 (3 x 15 mL). The combined organic phases were dried ($MgSO_4$) and reduced *in vacuo*. The residue was dissolved in CH_2Cl_2, filtered through a Celite® and the filtrate reduced *in vacuo*, yielding *cis*-**S9** as a colourless oil (2.4 mg, 13 µmol, 51%).

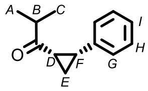

δ_H ($CDCl_3$, 400 MHz) 7.27-7.14 (5H, m, ***H***G, ***H***H, ***H***I), 2.68 (1H, ddd (app q), *J* = 8.3, ***H***E), 2.59 (1H, sept., *J* = 6.9, ***H***B), 2.50 (1H, ddd, *J* = 9.2, 7.5, 5.8, ***H***D), 1.87 (1H, ddd, *J* = 7.6, 5.7, 4.9, ***H***F), 1.31 (1H, ddd, *J* = 8.5, 7.5, 4.8, ***H***F'), 0.99 (3H, d, *J* = 6.9, ***H***A), 0.93 (3H, d, *J* = 6.9, ***H***C).

δ_C ($CDCl_3$, 101 MHz) 209.4, 136.2, 129.2, 127.9, 126.7, 41.9, 28.9, 28.3, 17.9, 17.8, 11.7.

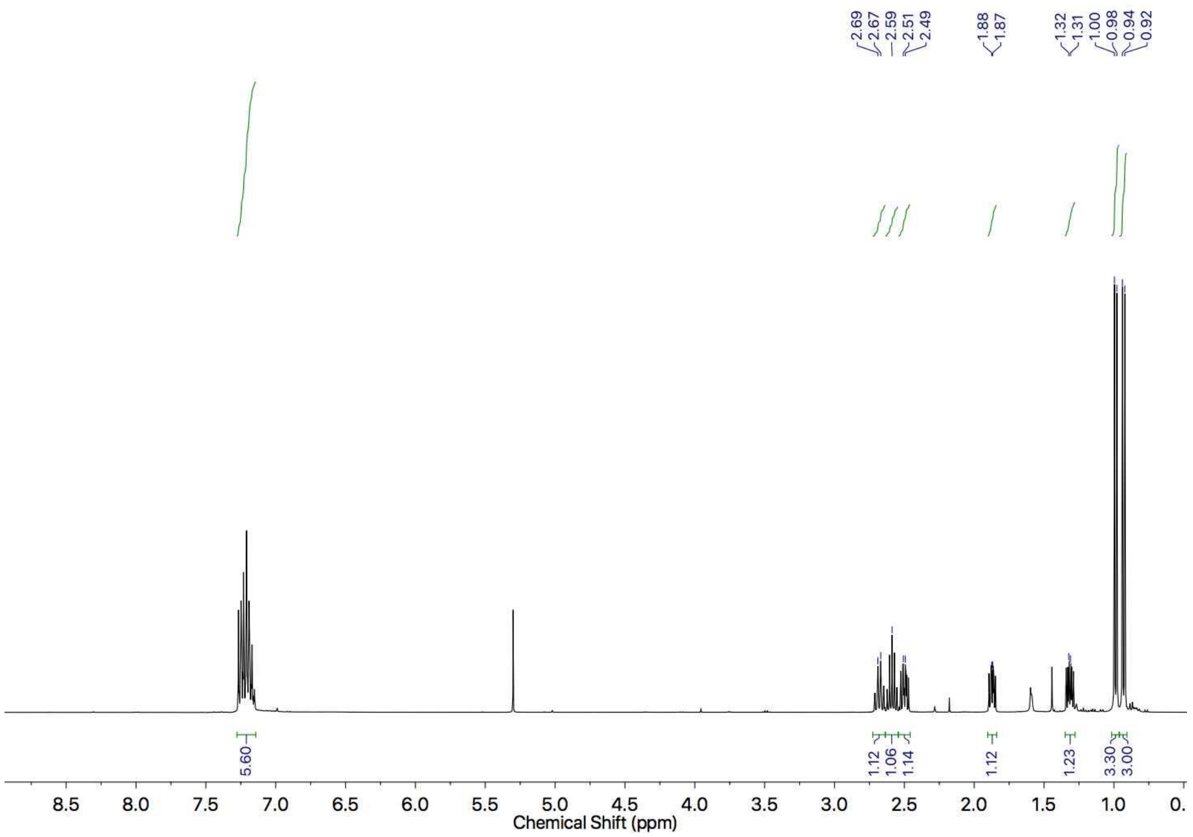

Figure S163: ^{1}H NMR (400 MHz, $CDCl_3$) of *cis*-**S9**.

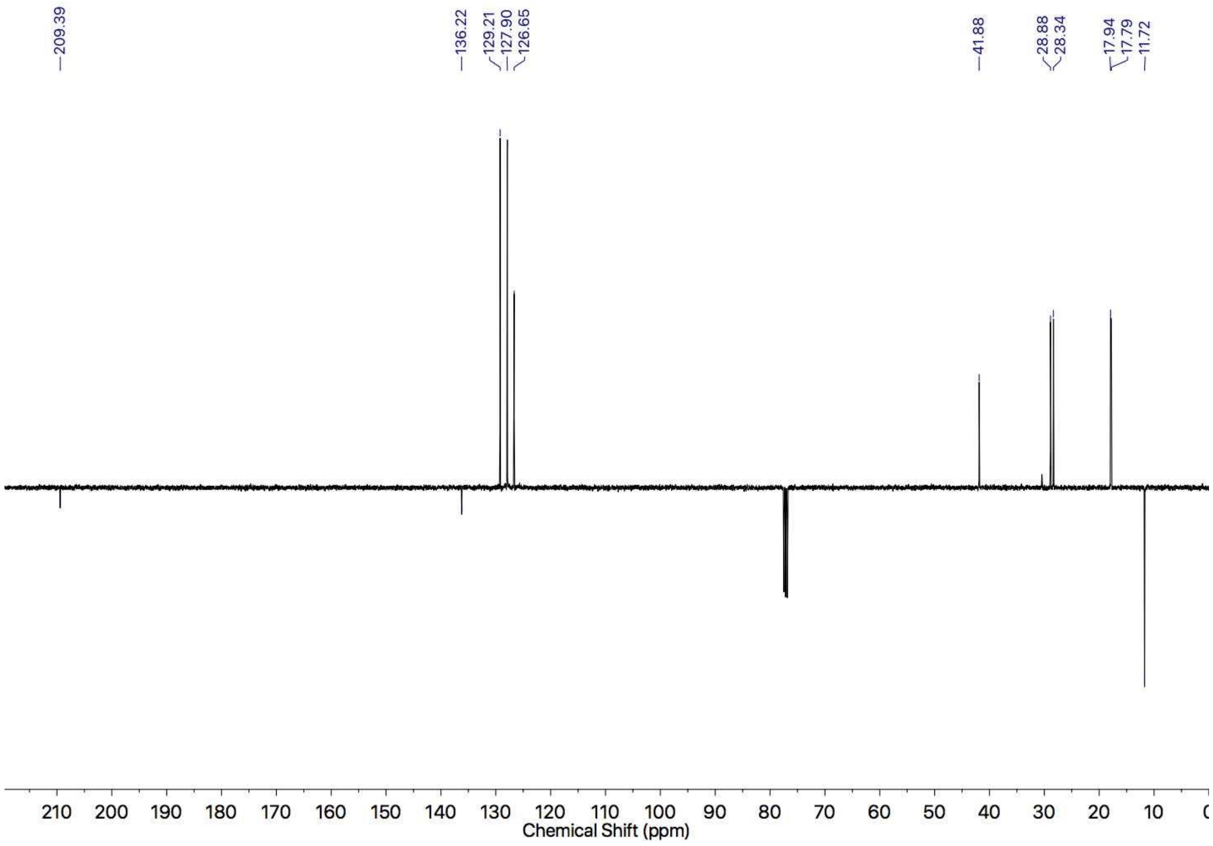

Figure S164: JMOD NMR (101 MHz, $CDCl_3$) of *cis*-**S9**.

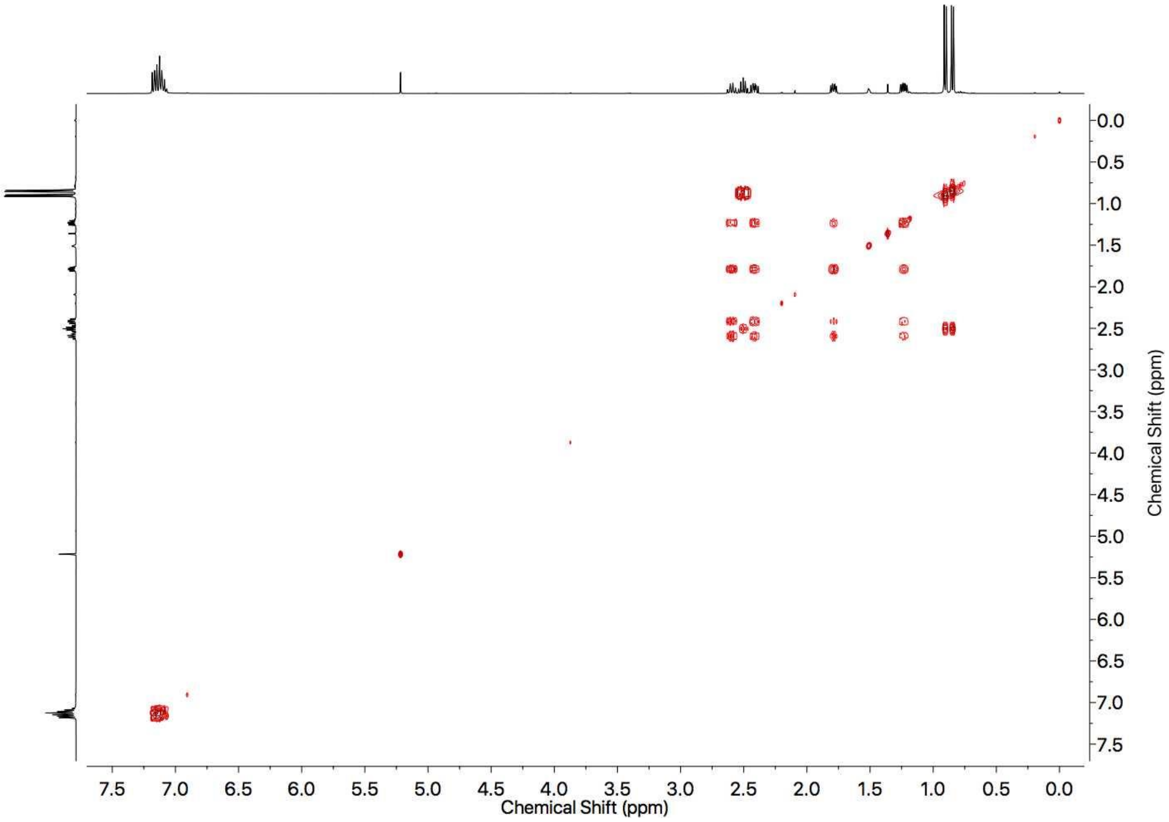

Figure S165: COSY NMR ($CDCl_3$) of *cis*-**S9**.

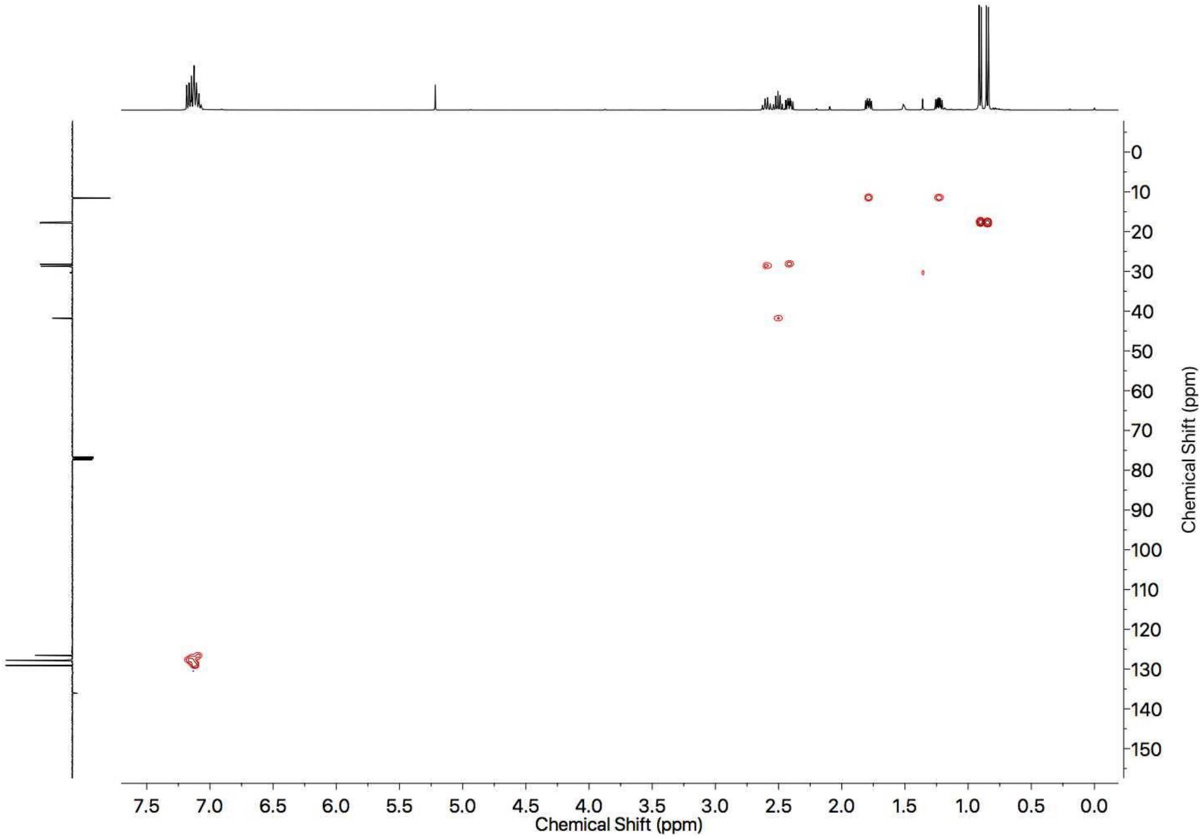

Figure S166: HSQC NMR ($CDCl_3$) of *cis*-**S9**.

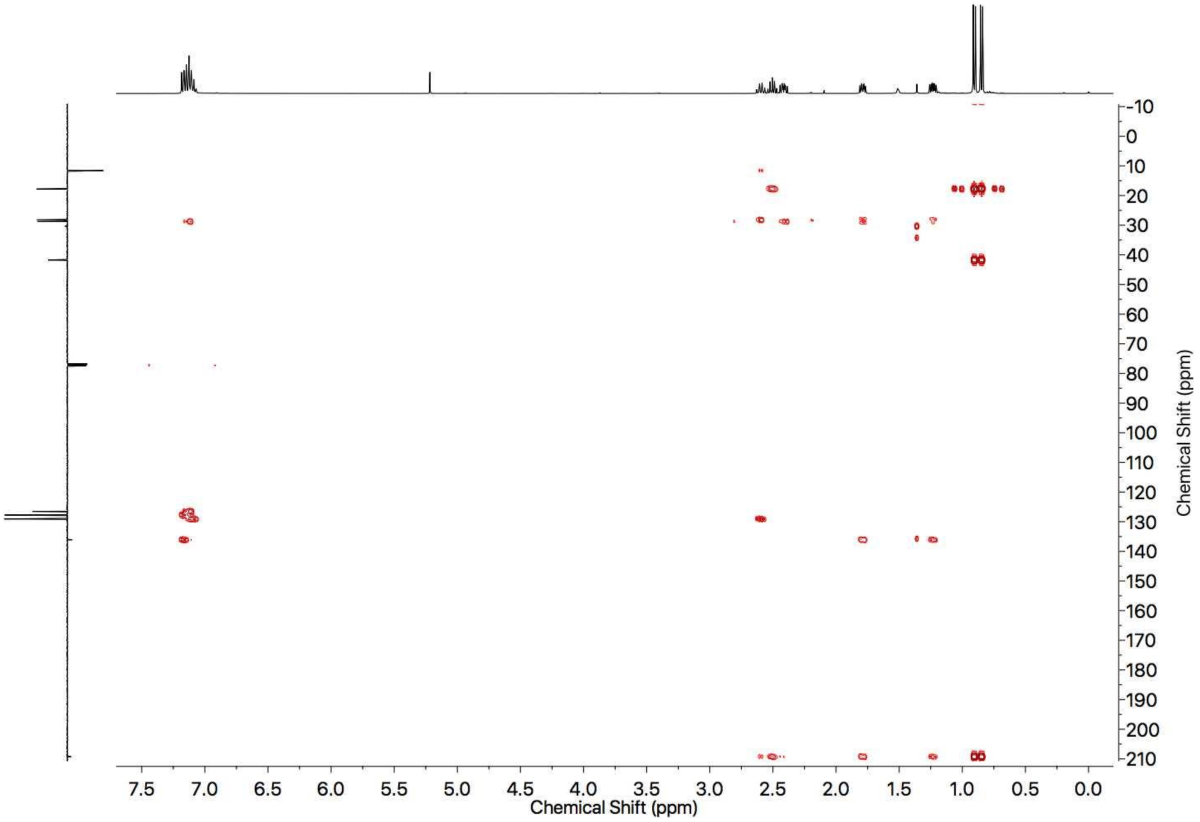

Figure S167: HMBC NMR ($CDCl_3$) of *cis*-**S9**.

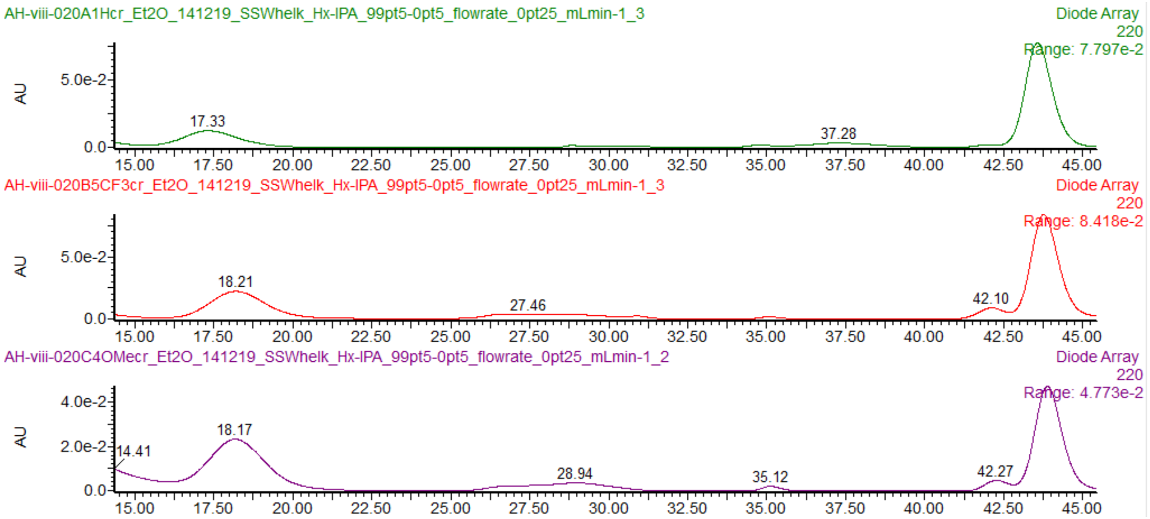

Figure S168: Chiral stationary phase HPLC ((*S*,*S*)Whelk, hexane-isopropanol 99.5 : 0.5, 303 K, load petrol, flowrate 0.25 mLmin^{-1}) of *cis*-**S9** crude reaction mixtures. 220 nm UV absorbance shown. Retention times (min): 18.1, 43.7. (1) Reduction of *cis*-**9** (*er* 22 : 78) to *cis*-**S9** (apparent *er* 22 : 78 integrals 22221 (17.3 min) : 81636 (43.6 min)). (2) Reduction of *cis*-**15** (*er* 27 : 73) to *cis*-**S9** (apparent *er* 29 : 71 integrals 36466 (18.2 min) : 90032 (43.7 min)). (3) Reduction of *cis*-**16** (*er* 30 : 70) to *cis*-**S9** (apparent *er* 39 : 61 integrals 32137 (18.2 min) : 50259 (43.9 min)).

6. Additional Reaction Scope

To investigate the potential of [Au((*R*$_{mp}$)-**6**)(Cl)] in other reactions we performed two previously reported cycloisomerisation reactions and an intramolecular hydroamination reaction to compare the outcomes to those achieved with (*R*)-DTBM-SEGPHOS®(AuCl)$_2$. In all cases little or no enantioselectivity was observed with [Au((*R*$_{mp}$)-**6**)(Cl)]. Similar results were obtained for (*R*)-DTBM-SEGPHOS®(AuCl)$_2$ except in the case of the hydroamination reaction (modest enantioselectivity).

Cycloisomerisation 1[13]

Enyne cyclisation substrate **S10** was synthesised according to a literature route.[14]

Compound **S11**

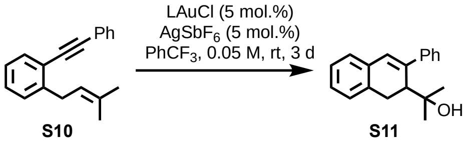

General procedure: A foil covered CEM MW vial was charged with AgSbF$_6$ (1.1 mg, 3.1 µmol, 0.05 eq.) and, in the case of [Au((R$_{mp}$)-**6**)(Cl)], [Cu(MeCN)$_4$]PF$_6$ (1.2 mg, 3.1 µmol, 0.05 eq.), then purged with N$_2$. [Au(L)(Cl)] (3.1 µmol, 0.05 eq.) was transferred in PhCF$_3$ (0.50 mL) and stirred for 5 minutes. **S10** (15.3 mg, 0.062 mmol, 1 eq.) in PhCF$_3$ (0.2 mL) and H$_2$O (2.8 mg, 0.155 mmol, 2.5 eq.) were added. The mixture was stirred at rt for 3 days. After 3 days, the reaction was quenched with 1 drop of NEt$_3$, and concentrated *in vacuo*. **S11** was purified by column chromatography (SiO$_2$, petrol-EtOAc 0→20%) yielding a white solid. Enantiomeric excess was evaluated by chiral stationary phase HPLC. Spectra match literature.[13]

δ_H (CD$_2$Cl$_2$, 400 MHz) 7.58 – 7.50 (2H, m), 7.43 – 7.34 (2H, m), 7.33 – 7.25 (1H, m), 7.21 – 7.09 (4H, m), 6.86 (1H, s), 3.32 – 3.19 (2H, m), 3.11 (1H, dd, J = 7.1, 2.3 Hz), 1.18 – 1.12 (1H, m), 0.96 (3H, s), 0.88 (3H, s).

δ_C (CD$_2$Cl$_2$, 101 MHz) 143.9, 140.9, 136.0, 135.2, 129.1, 128.4, 128.1, 127.8, 127.6, 127.2, 126.9, 126.8, 75.3, 46.0, 31.3, 29.2, 28.1.

LRMS [M – H$_2$O$^{+\cdot}$] 246.1.

Table S14. Summary of reactions leading to cyclopropanes **S11**.

	Catalyst	Yield / %	*er*
1	(Ph$_3$P)AuCl	25	50 : 50
2	[Au((*R*$_{mp}$)-**6**)(Cl)]	18	48 : 52
3	(*R*)-DTBM-SEGPHOS®(AuCl)$_2$	9	57 : 43

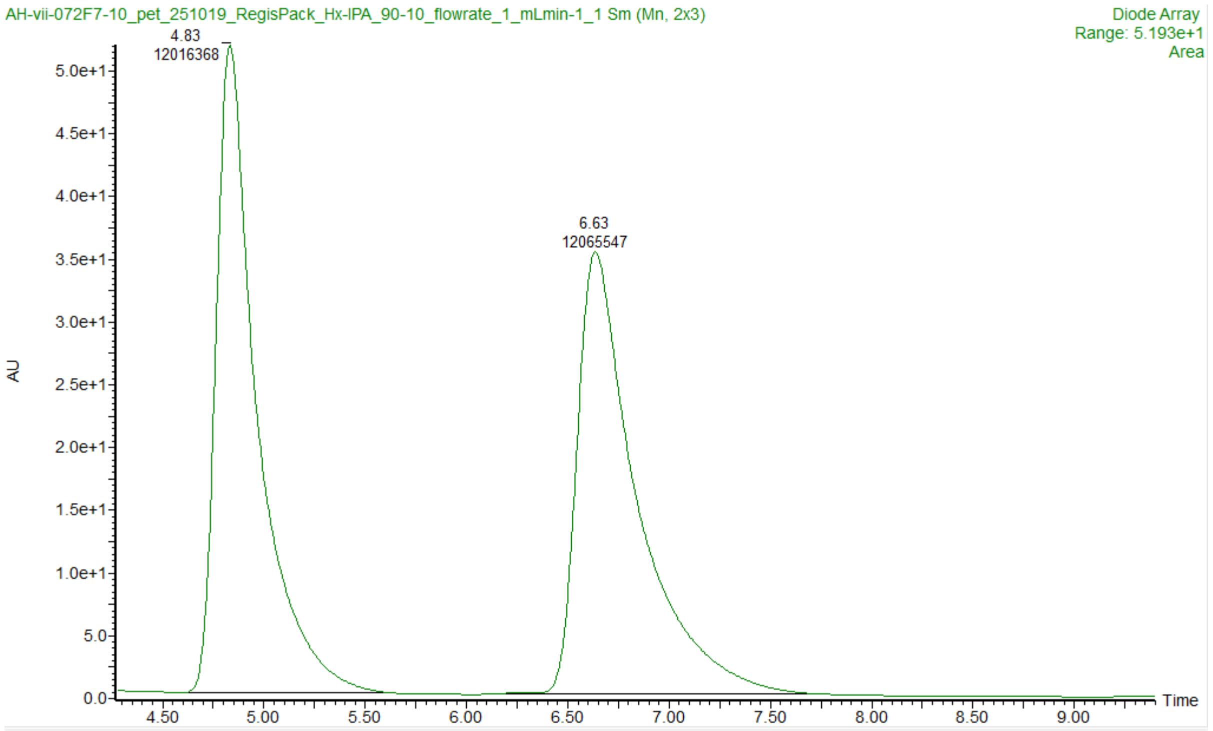

Figure S169: Entry 1. Chiral Stationary Phase HPLC (RegisPack, n-hexane-isopropanol 90 : 10, 303 K, load Et_2O, flowrate 1 $mLmin^{-1}$) of 50 : 50 *er*. Retention times (min): 4.8, 6.6.

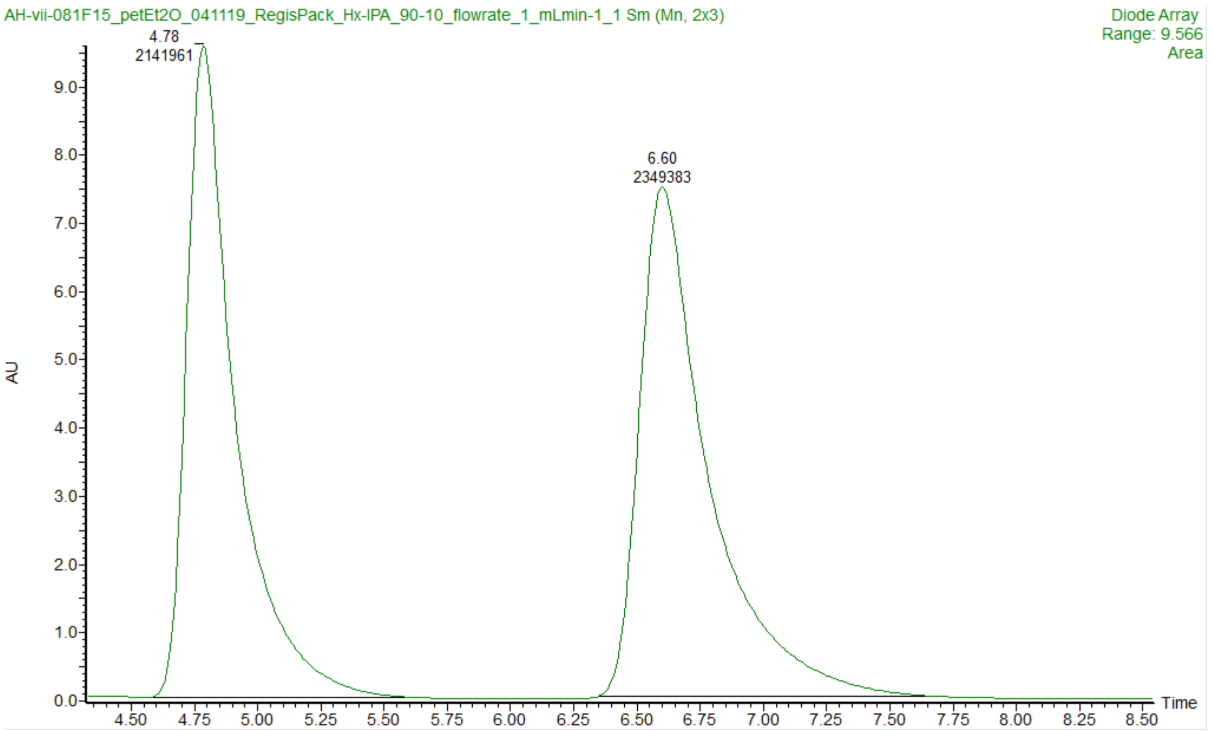

Figure S170: Entry 2. Chiral Stationary Phase HPLC (RegisPack, n-hexane-isopropanol 90 : 10, 303 K, load Et_2O, flowrate 1 $mLmin^{-1}$) of 48 : 52 *er*. Retention times (min): 4.8, 6.6.

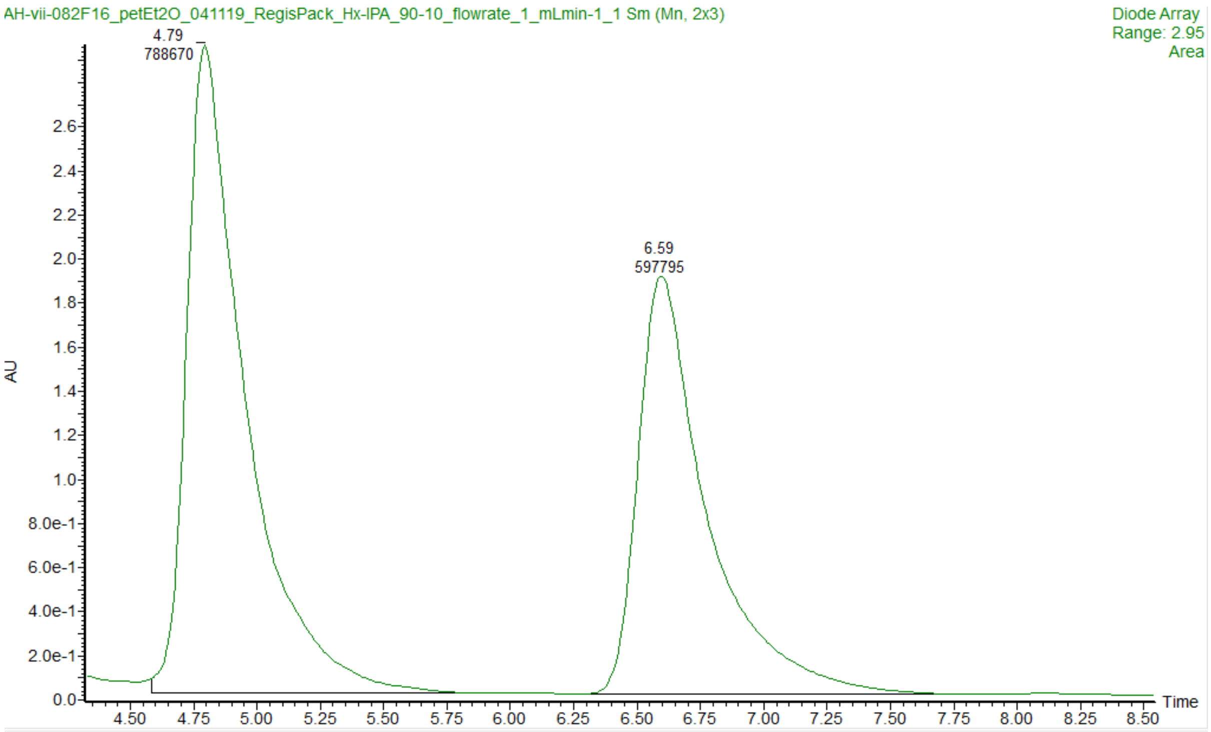

Figure S171: Entry 3. Chiral Stationary Phase HPLC (RegisPack, n-hexane-isopropanol 90 : 10, 303 K, load Et_2O, flowrate 1 $mLmin^{-1}$) of 57 : 43 *er*. Retention times (min): 4.8, 6.6.

Cycloisomerisation 2[15]

Enyne cyclisation substrate **S12** was synthesised according to a literature route.[16]

Compound **S13**

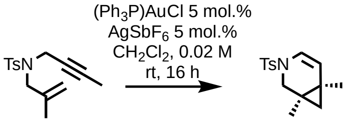

General procedure: A CEM MW vial was charged with [Au(L)(Cl)] (4.4 µmol, 0.05 eq.), $AgSbF_6$ (1.5 mg, 4.4 µmol, 0.05 eq.), and in the case of [Au((R_{mp})-**6**)(Cl)], $[Cu(MeCN)_4]PF_6$ (1.7 mg, 4.4 µmol, 0.05 eq.), and purged with N_2. Alkyne substrate **S12** (24.4 mg, 0.088 mmol, 1.0 eq.) was added in degassed anhydrous CH_2Cl_2 (4.4 mL, 0.02 M) and the reaction stirred at rt for 16 h. The mixture was filtered through Celite© and concentrated *in vacuo*. The residue was purified by column chromatography (SiO_2, isocratic pet-EtOAc 95 : 5). Spectra match literature.[15]

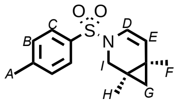

δ_H ($CDCl_3$, 400 MHz) 7.65 (2H, d, J = 8.3, **H**$_C$), 7.31 (2H, d, J = 8.4, **H**$_B$), 6.25 (1H, d, J = 8.0, **H**$_E$), 5.17 (1H, d, J = 8.0, **H**$_D$), 3.77 (1H, d, J = 11.5, **H**$_I$), 3.68 (1H, d, J = 11.5, **H**$_{I'}$), 2.42 (3H, s, **H**$_A$), 1.11 (6H, s, **H**$_F$, **H**$_H$), 0.72 (1H, d, J = 4.3, **H**$_G$), 0.33 (1H, d, J = 4.3, **H**$_{G'}$).

δ_C ($CDCl_3$, 101 MHz) 143.7, 135.2, 129.9, 127.2, 120.2, 118.3, 46.8, 29.5, 26.4, 24.0, 21.7, 18.8, 17.6.

LRMS [$M^{+\cdot}$] 277.0.

Table S15. Summary of reactions leading to cyclopropanes **S13**.

	Catalyst	Yield / %	*er*
1	$(Ph_3P)AuCl$	76	50 : 50
2	[Au((*R*$_{mp}$)-**6**)(Cl)]	37	51 : 49
3	(*R*)-DTBM-SEGPHOS®$(AuCl)_2$	20	50 : 50

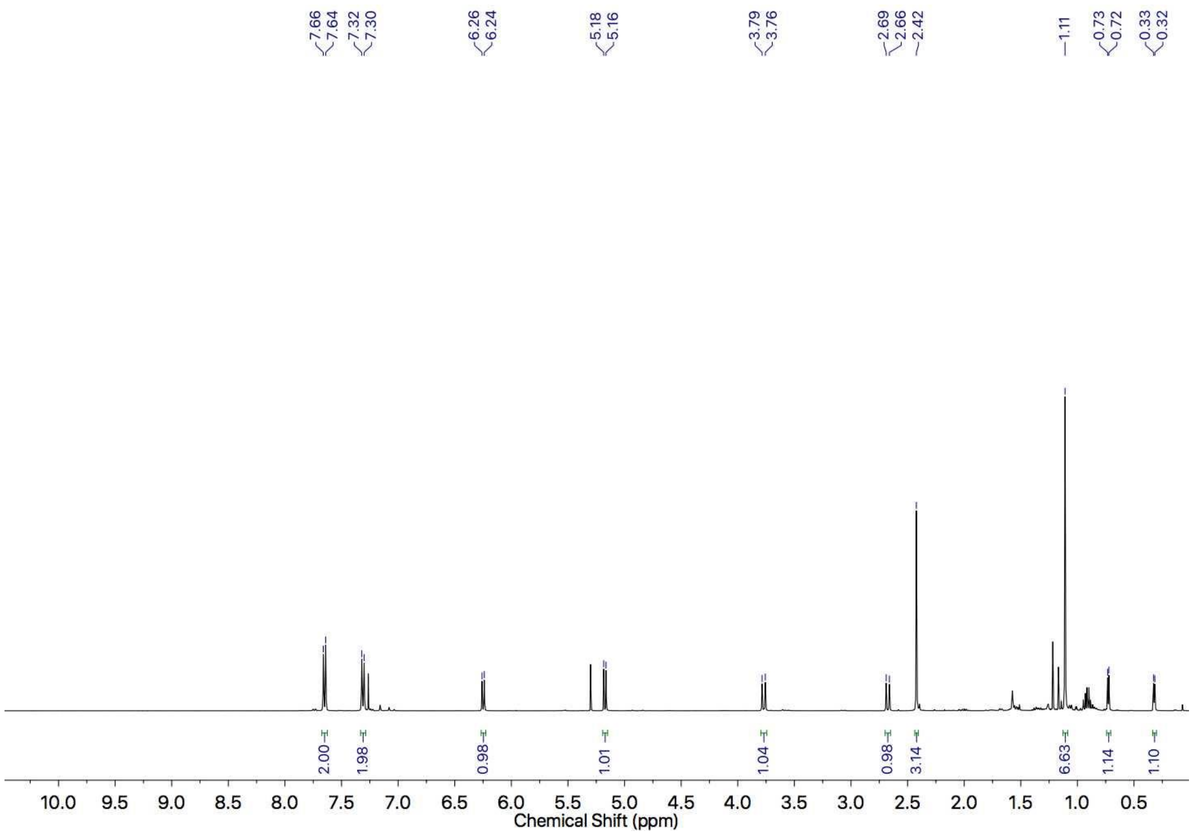

Figure S172: ^{1}H NMR of **S13** ($CDCl_3$, 400 MHz).

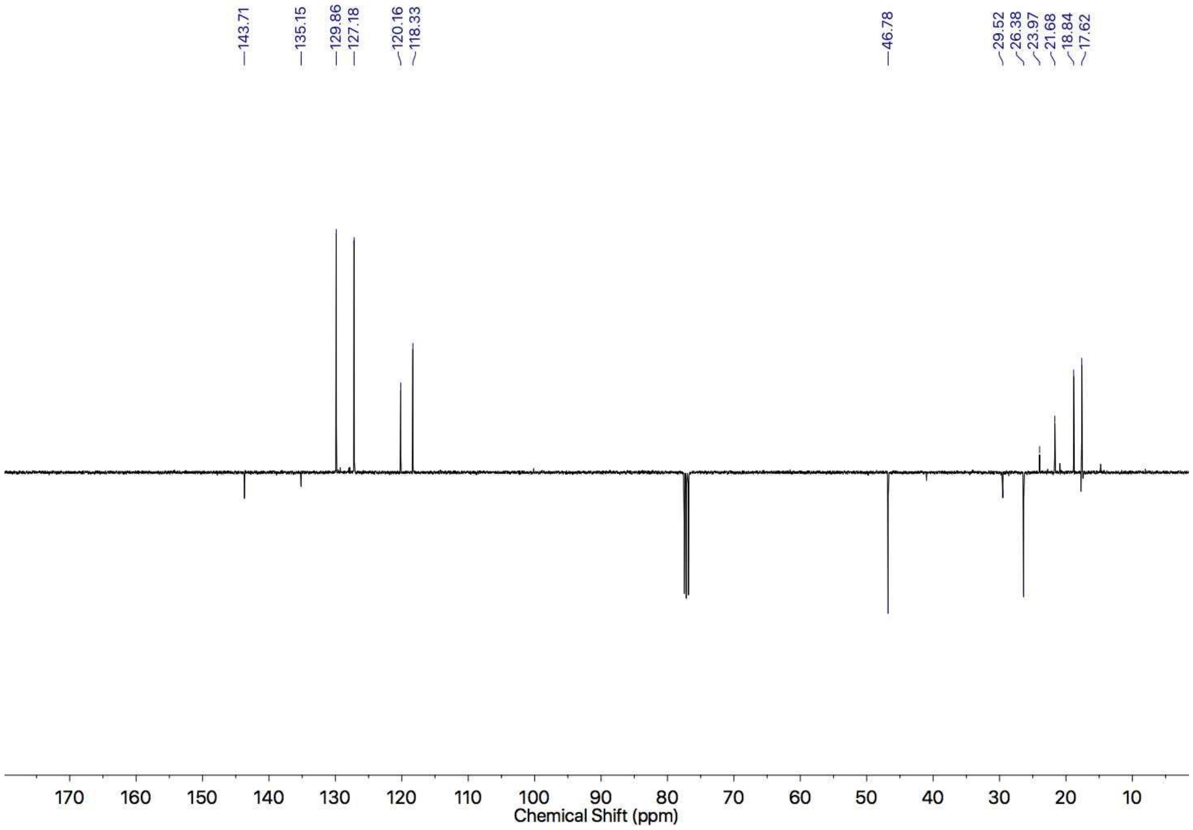

Figure S173: JMOD NMR of **S13** ($CDCl_3$, 101 MHz).

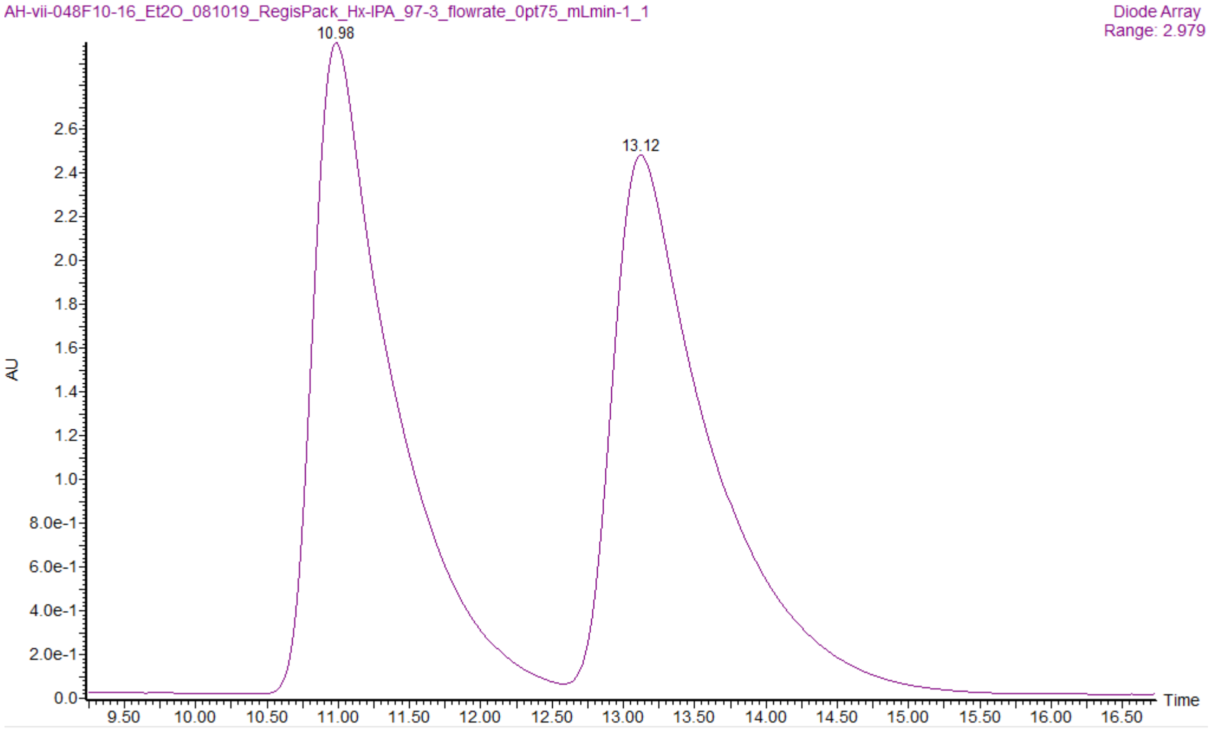

Figure S174: Entry 1. Chiral Stationary Phase HPLC (RegisPack, n-hexane-isopropanol 97 : 3, 303 K, load Et_2O, flowrate 0.75 $mLmin^{-1}$) of 50 : 50 *er*. Retention times (min): 11.0, 13.1.

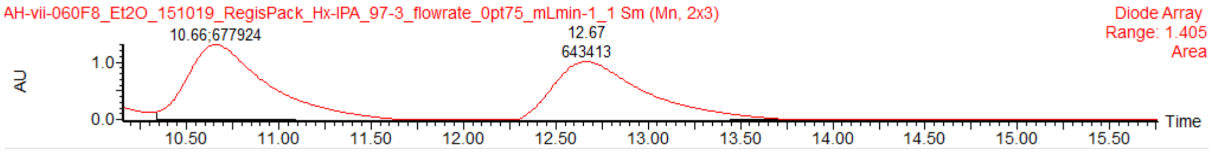

Figure S175: Entry 2. Chiral Stationary Phase HPLC (RegisPack, n-hexane-isopropanol 97 : 3, 303 K, load Et_2O, flowrate 0.75 $mLmin^{-1}$) of 51 : 49 *er*. Retention times (min): 10.7, 12.7.

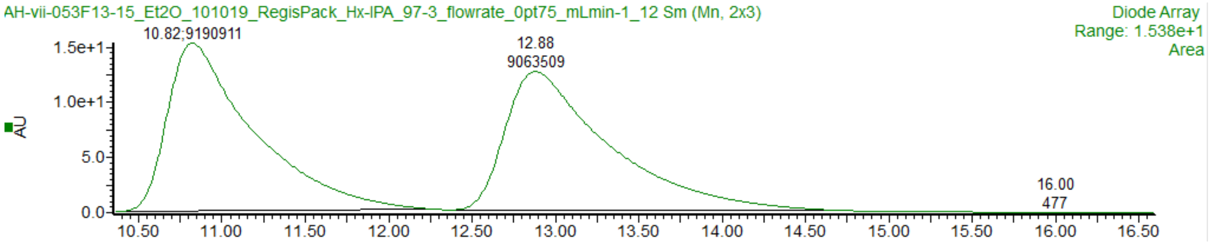

Figure S176: Entry 3. Chiral Stationary Phase HPLC (RegisPack, n-hexane-isopropanol 97 : 3, 303 K, load Et_2O, flowrate 0.75 $mLmin^{-1}$) of 50 : 50 *er*. Retention times (min): 10.8, 12.9.

Intramolecular Hydroamination of Allenes

Compound S14 was synthesised according to a literature route.[17]

Compound **S15**

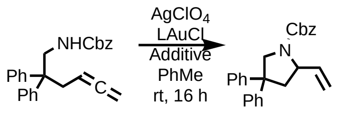

S14 **S15**

General Procedure: A CEM MW vial was charged with $AgClO_4$ (0.9 mg, 4.5 µmol, 0.05 eq.), LAuCl (4.5 µmol, 0.05 eq.) and in the case of [Au((R_{mp})-**6**)(Cl)], $[Cu(MeCN)_4]PF_6$ (1.7 mg, 4.5 µmol, 0.05 eq.). The flask was purged with N_2, and allene **S14** (34.7 mg, 0.091 mmol, 1.0 eq.) was added in degassed anhydrous PhMe (0.60 mL, 0.15 M) and stirred at rt overnight. The mixture was concentrated *in vacuo*. The residue was purified by column chromatography (SiO_2, pet-Et_2O 0→25%) yielding a colourless oil. Spectra match literature.[17]

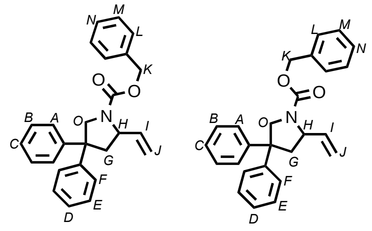

S15

A mixture of carbamate rotamers was observed. δ_H ($CDCl_3$, 400 MHz) 7.48-7.07 (15H, m), [5.78 (td, J = 10.5, 7.1), 5.77 (td, J = 10.5, 7.1), 1:1, 1H], 5.33-5.06 (4H, m), [4.76 (dd, J = 11.6, 1.9), 4.60 (dd, J = 11.5, 1.6) 1:1, 1H], [4.17 (dd, J 16.0, 7.4), 4.11 (dd, J 15.6, 7.3), 1:1, 1H], [3.72 (d, J = 15.6 Hz), 3.71 (d, J = 15.6), 1:1, 1H], 2.88-2.81 (1H, m), [2.47 (dd, J = 12.5, 9.8 Hz), 2.42 (dd, J = 12.5, 9.8 Hz), 1:1, 1 H].

δ_C ($CDCl_3$, 101 MHz) 155.4, 154.8, 145.5, 144.9, 139.3, 138.6, 137.0, 136.8, 128.8, 128.7, 128.4, 128.3, 128.2, 127.8, 127.7, 126.9, 126.7, 126.6, 115.8, 115.2, 67.0, 59.6, 59.1, 56.3, 53.2, 52.8, 45.8, 44.7.

LRMS [M + H^+] m/z 384.3286 (calc. $C_{26}H_{26}N_1O_2$ m/z 384.1964).

Table S16. Summary of reactions leading to cyclopropanes **S15**.

	**Catalyst**	**Yield / %**	***er***
1	(Ph_3P)AuCl	5	50 : 50
2	[Au((*R*$_{mp}$)-**6**)(Cl)]	51	50 : 50
3	(*R*)-DTBM-SEGPHOS®$(AuCl)_2$	38	67 : 33

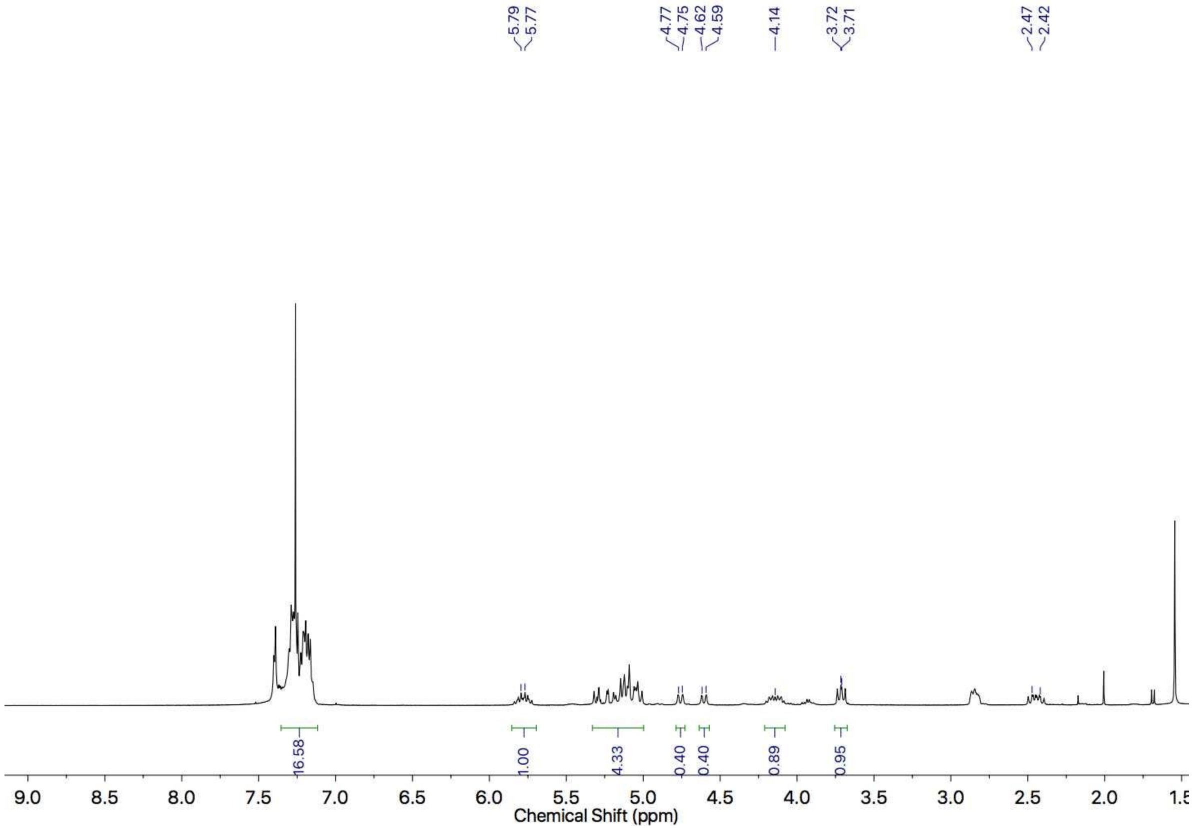

Figure S177: ^{1}H NMR of **S15** ($CDCl_3$, 400 MHz).

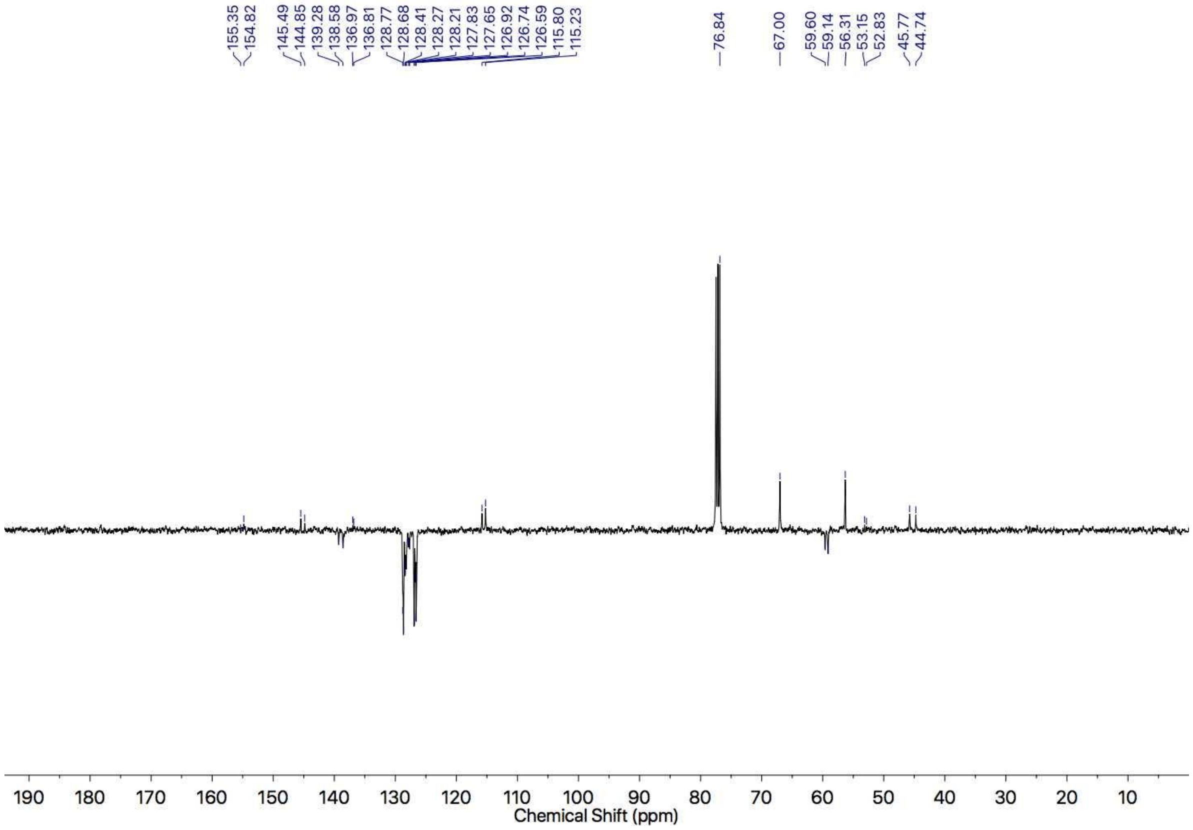

Figure S178: JMOD NMR of **S15** ($CDCl_3$, 101 MHz).

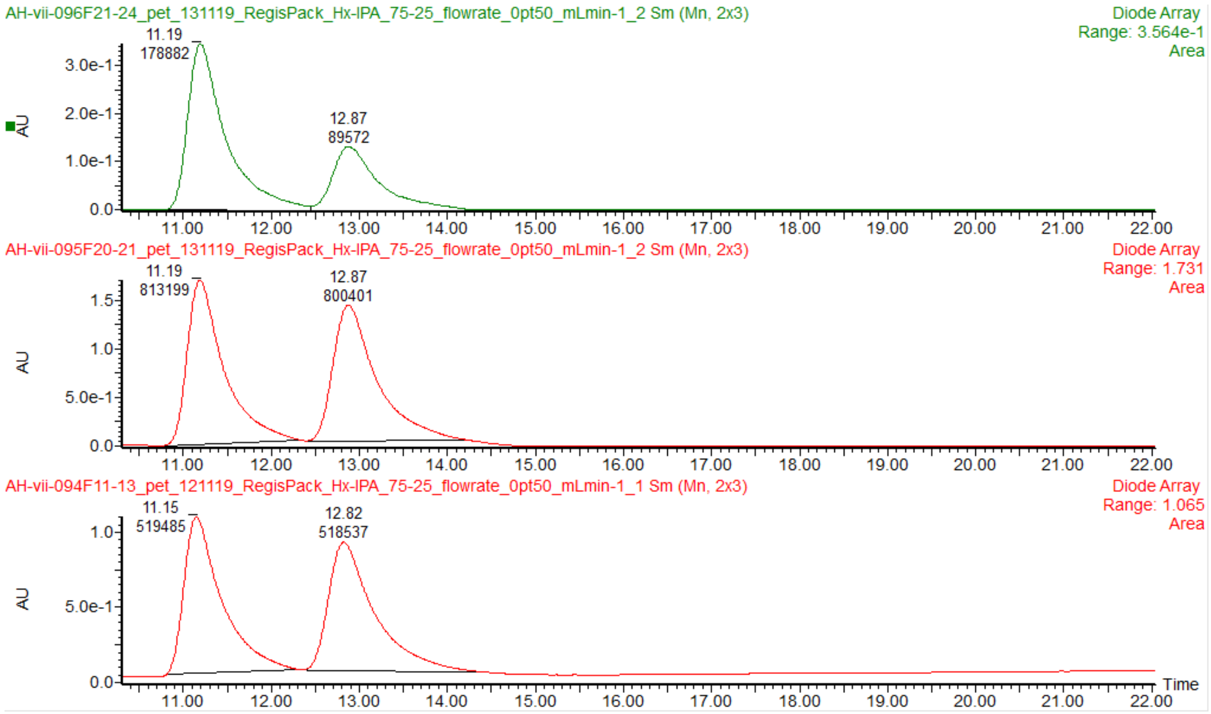

Figure S179: Chiral Stationary Phase HPLC (RegisPack, n-hexane-isopropanol 75 : 25, 303 K, load pet, flowrate 0.50 mLmin^{-1}). Retention times (min): 11.2, 12.9. Top (entry 3) *er* 67 : 33, Middle (entry 2) *er* 50 : 50, Bottom (entry 1) *er* 50 : 50.

7. Molecular Modelling

Models of the reaction of ester **7** and styrene (**8**) mediated by [Au(PPh_3)(Cl)]

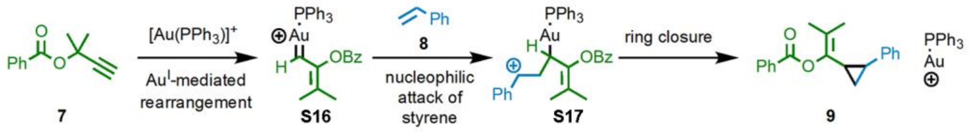

Figure S180. Proposed pathway for the reaction of **7** and **8** mediated by [Au(PPh_3)(Cl)]

We initially investigated the reaction of ester **7** with styrene (**8**) mediated by [Au(PPh_3)(Cl)] (**Figure S180**) to determine a reasonable transition state for the same reaction mediated by rotaxane complex [Au(**6**)(Cl)]. Models of the transition states for the reaction of presumed carbene intermediate **S16** with styrene were constructed based on transition state structures reported by Echavarren and co-workers in a related reaction by modifying the substituents of the phosphine and carbene.[18] Scanning of the distance between the carbenic carbon and the β-position of styrene, followed by transition state optimisation of the structures that corresponded to energetic maxima on the scanned coordinate yielded four transition states **TSI** (**Figure S181**), two leading to *cis*-**9**, two leading to *trans*-**9**, of which *cis*-**TSIb** was found to be more stable by 10.3 kJmol^{-1} than the nearest alternative pathway. All transition states were determined to be first order saddle points with a single imaginary frequency mode.

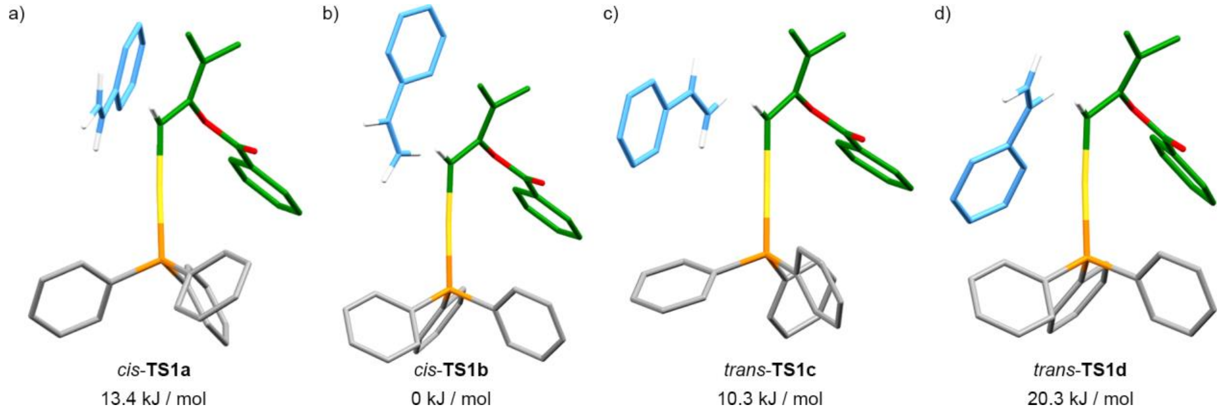

Figure S181. Transition states and their relative energies for the reaction **S16→S17** determined by DFT (CAM-B3LYP, 6-31G*(C,H,O,P)/SDD(Au)). For structures see "Model_AuPPh3Cl_TS1.cif" submitted as electronic supporting information.

It should be noted that, in accordance with Echavarren and co-workers' previous report, in all cases IRC calculations indicate that transition states **TSI** correspond to a stepwise mechanism as shown in **Figure S180**, in which the styrene acts as a nucleophile on the electrophilic carbene to generate α-aryl cations **S17**, that then undergo ring closure with extremely low barrier to give the observed cyclopropane product. Specifically, in the case of *cis*-**TSIb** (**Figure S181**), the reaction of

S16 to produce **S17** was found to proceed with a barrier of 3.0 kJmol^{-1} and to be extremely favourable (-45.2 kJmol^{-1}). Subsequent ring closure was essentially barrierless, passing through *cis*-**TSIIb** (first order saddle point, single imaginary frequency mode) to generate cyclopropane *cis*-**9** initially with the Au^{I} catalyst associated with the carbonyl of the ester. (Note: although **S17** and **TSIIb** were found to be minima and maxima respectively on the potential energy surface by frequency analysis, their structures are related by an extremely small atomic reorganisation and were energetically indistinguishable at this level of theory).

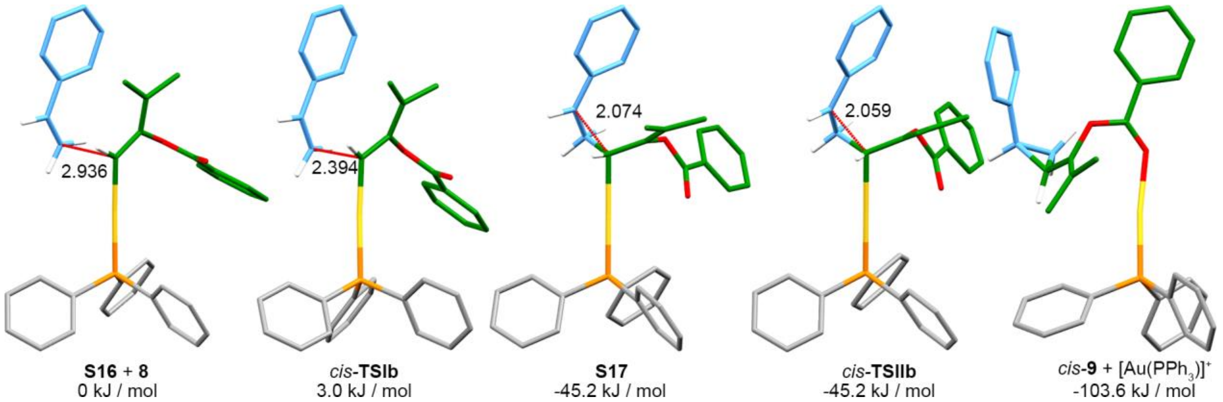

Figure S182. Intermediates and transition states and their relative energies for the reaction from **S16** → cyclopropane **9** determined by DFT (CAM-B3LYP, 6-31G*(C,H,O,P)/SDD(Au)). For structures see "Model_DFT_AuPPh3Cl_full_reaction.cif" submitted as electronic supporting information.

Diastereomeric transition states for the reaction of 7 with 8 mediated by [Au(6)(Cl)]

A model of the transition state for the reaction of **7** with **8** mediated by [Au(**6**)(Cl)] was constructed by joining *cis*-**TSIb** with a model of [Cu((R_{mp})-**5**]$^{+}$ by replacing one of the Ph substituents of *cis*-**TSIb** (highlighted) with the rotaxane framework with the highlighted atoms removed (**Figure S183**). The diastereomeric model was generated by using the same rotaxane framework with the enantiomer of *cis*-**TSIb**.

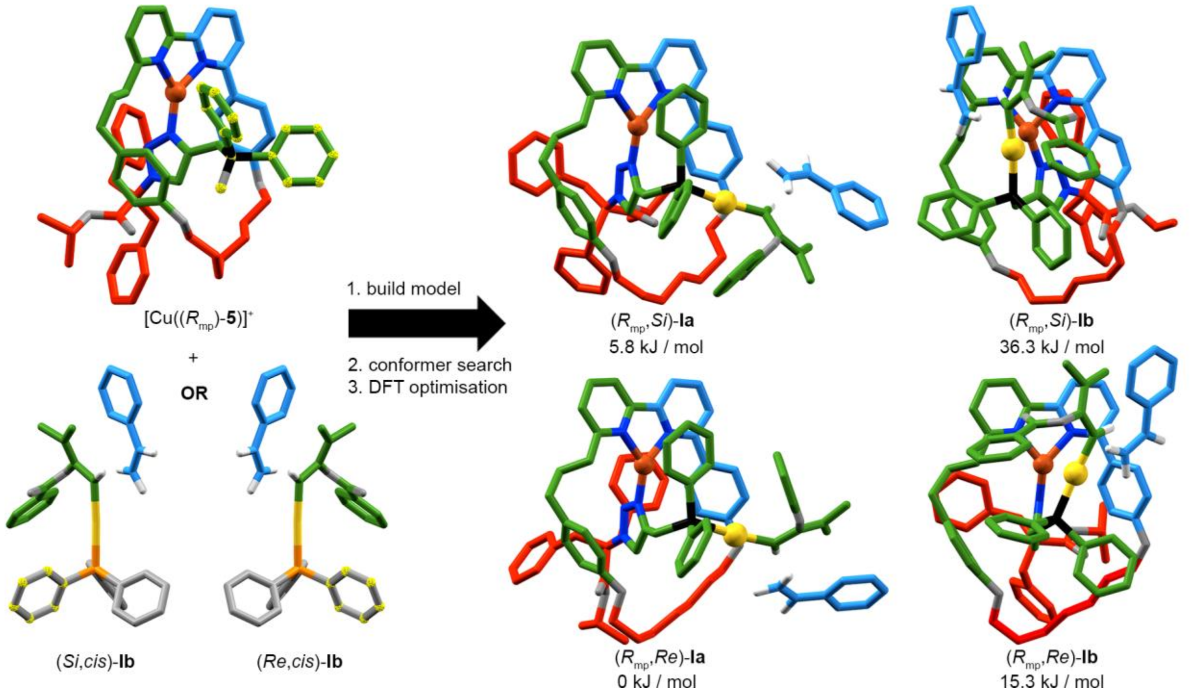

Figure S183. Procedure for the construction of candidate conformations of the transition state for the reaction mediated by [Au(**6**)(Cl)], the four lowest energy conformations found and their energies as evaluated using DFT (CAM-B3LYP, 6-31G(C,H,O,P,N)/SDD(Au,Cu)). For structures of **Ia** and **Ib** see "Model_Rmp_confs_I.cif" submitted as electronic supporting information.

A conformational search was performed using these models of (R_{mp},*Re*)-**Ia** and (R_{mp},*Si*)-**Ia**, where Re and Si refer to the face of the carbene approached by styrene (Note: (R_{mp},*Si*)-**Ia** gives rise to the observed major product (1*S*,2*R*)-**9**). The transition state fragment (carbene unit, styrene moiety, Au and P atoms) was frozen, the Cu ion was restricted to a trigonal planar configuration, and a conformational search was performed (Spartan '10, MMF) by rotating triazole-N^1-C, and the single bonds of the C-C-P fragment in 60 degree increments followed by re-optimisation using the same model with no restriction on these bond angles. This search was repeated several times starting from different initial states and yielded reproducible results; for both diastereomeric structures, two families of low energy conformations were identified, one in which the P-Au bond was projected parallel or nearly parallel to the triazole-N-Cu bond and the other in which the P-Au bond is projected towards the same O substituent of the macrocycle. The two lowest energy conformations included one of each family and were predicted to be more stable than the next lowest energy conformation by > 10 kJ / mol. These conformations were then reoptimized with the transition state fragment frozen using DFT (CAM-B3LYP, 631-G(C,H,O,P,N)/SDD(Au, Cu)) with the result that conformers **Ia** were found to be more stable by >15 kJ / mol (**Figure S183**).

Conformers (R_{mp},*Re*)-**Ia** and (R_{mp},*Si*)-**Ia** were taken forward for transition state optimisations and

the results of these calculations are summarised in **Table S17** A transition state search using an ONIOM method was carried out initially with the transition state fragment (including the Ph substituents of the P atom), in the high level (CAM-B3LYP, 631-G(C,H,O,P)/SDD(Au)) and the rest of the molecule in the low level (UFF) and frozen, except for the methylene linking the different fragments. The outputs of the ONIOM calculations were then subjected to a transition state search using DFT (CAM-B3LYP, 631-G(C,H,O,P,N)/SDD(Au,Cu)) with no frozen or restricted atoms. This yielded two diastereomeric transition state structures **TS1** with a single imaginary mode. These structures were re-optimized in the gas phase (entry 5, CAM-B3LYP, 631-G*(C,H,O,P,N)/SDD(Au,Cu)) and in $CHCl_3$ (entry 4, PCM($CHCl_3$), CAM-B3LYP, 631-G(C,H,O,P,N)/SDD(Au,Cu)), the latter of which was subjected to a single point energy calculation (entry 6, $CHCl_3$, CAM-B3LYP, 631-G*(C,H,O,P,N)/SDD(Au,Cu)). Attempts to directly locate a transition state with these latter parameters proved prohibitively computationally expensive. The structures of Re/Si **TS1-Oniom** (**Table S17**, entry 2), **TS1-631G** (entry 3), **TS1-631GCHCl3** (entry 4) and **TS1-631GG** (entry 5) are provided as supporting information (.xyz).

Examining the computed relative energies of **TS1**, the outcome is strongly dependent on the method used. When evaluated using an Oniom method (entry 2), the calculations predict a very large preference for the (1*S*,2*R*)-**9** major product observed experimentally. Optimisation of the entire structure using DFT with a 6-31G basis set in the gas phase (entry 3) predicts the opposite enantiomer. When solvent is included ($CHCl_3$, entry 4), or a larger basis set employed in the gas phase (entry 5) the correct product is predicted, both with reasonable agreement to the experimental value. Re-subjecting the output of entry 4 to a single point energy calculation with a larger basis set (6-31G*, entry 6) results in an even higher predicted preference for the experimentally observed major product. The results in **Table S17** suggest that although the structures of **TS1** determined using DFT are useful from an illustrative point of view, the relative energies determined are not particularly reliable, especially given the relatively small difference (~3 kJ / mol) predicted from experiment.

Table S17. Comparison of the outcomes of different calculations for **TS1**

Entry	Method	Basis Set	Solvent	Imaginary modes (cm^{-1})	ΔE[*Si* –*Re*] (kJ / mol)	(1*S*,2*R*)-9 : (1*R*,2*S*)-9
1	Experiment	-	$CHCl_3$	-	–3.0	79 : 21
2	Oniom (CAM-B3LYP:UFF)	6-31G/SDD(Au)	-	–175.19 (*Si*), –51.23 (*Re*)	–25.4	100 : 0
3	CAM-B3LYP	6-31G/SDD(Au,Cu)	-	–159.89 (*Si*), –118.84 (*Re*)	1.7	32 : 68
4	CAM-B3LYP	6-31G/SDD(Au,Cu)	$CHCl_3$	–176.85 (*Si*), –175.54 (*Re*)	–2.3	74 : 26
5	CAM-B3LYP	6-31G*/SDD(Au,Cu)	-	–149.80 (*Si*), –120.42 (*Re*)	–4.9	90 : 10
6	CAM-B3LYP (single point)	6-31G*/SDD(Au,Cu)	$CHCl_3$	-	–6.4	94 : 6

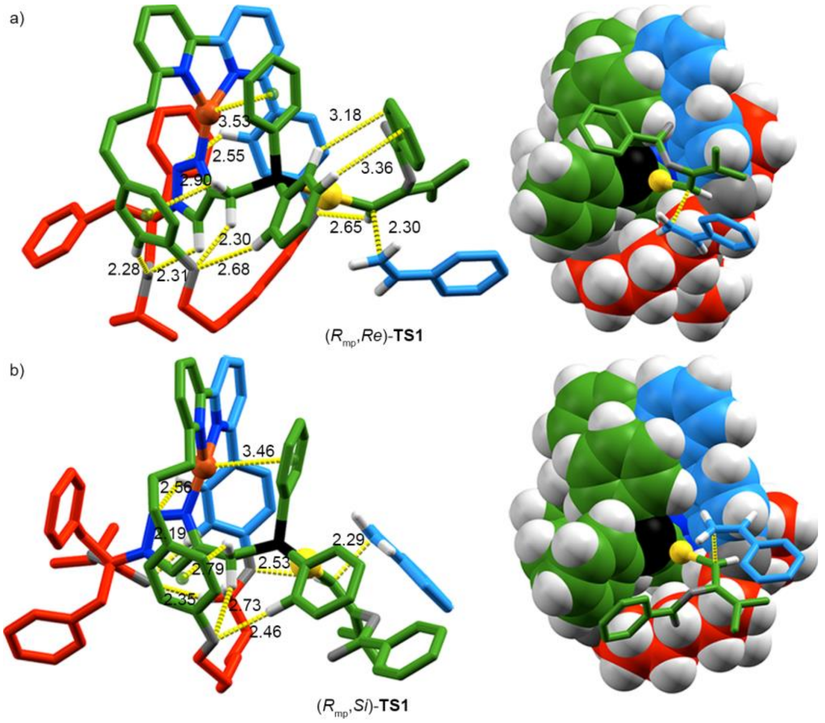

Figure S184. Transition states **TS1** determined for the reaction mediated by [Au(**6**)(Cl)] using DFT (CAM-B3LYP, 6-31G(C,H,O,P,N)/SDD(Au,Cu), PCM($CHCl_3$)) with selected intercomponent interactions and distances indicated. For structures see “Model_Table_S17_structures.cif” submitted as electronic supporting information.

Transition states for the formation of cyclopropanes 13, 15 and 16 mediated by [Au(6)(Cl)]

Transition states for the reactions leading to cyclopropanes **13**, **15** and **16** were constructed by modification of diastereomeric transition states **TS1** (**Table S17**, entry 4), followed by repeating the transition state search, first in the gas phase then in $CHCl_3$ (PCM). The structures obtained are shown in **Figure S185** and the results are summarized in **Table S18**.

Table S18. Comparison of the outcomes of different calculations of **TS1**

Entry	Method	Basis Set	Solvent	Imaginary modes (cm^{-1})	ΔE[*Si* –*Re*] (kJ / mol)	(1*S*,2*R*)-*9* : (1*R*,2*S*)-*9*
			Cyclopropanes **13**			
1	Experiment	-	$CHCl_3$	-	0.4	55 : 45
2	CAM-B3LYP	6-31G/SDD(Au,Cu)	$CHCl_3$	–190.27 (*Si*), –175.54 (*Re*)	3.1	16 : 84
			Cyclopropanes **15**			
3	Experiment	-	$CHCl_3$	-	–2.2	73 : 27
4	CAM-B3LYP	6-31G/SDD(Au,Cu)	$CHCl_3$	–177.19 (*Si*), –160.62 (*Re*)	–3.1	80 : 20
			Cyclopropanes **16**			
5	Experiment	-	$CHCl_3$	-	–2.0	70 : 30
6	CAM-B3LYP	6-31G/SDD(Au,Cu)	$CHCl_3$	–194.11 (*Si*), –170.46 (*Re*)	–3.0	79 : 21

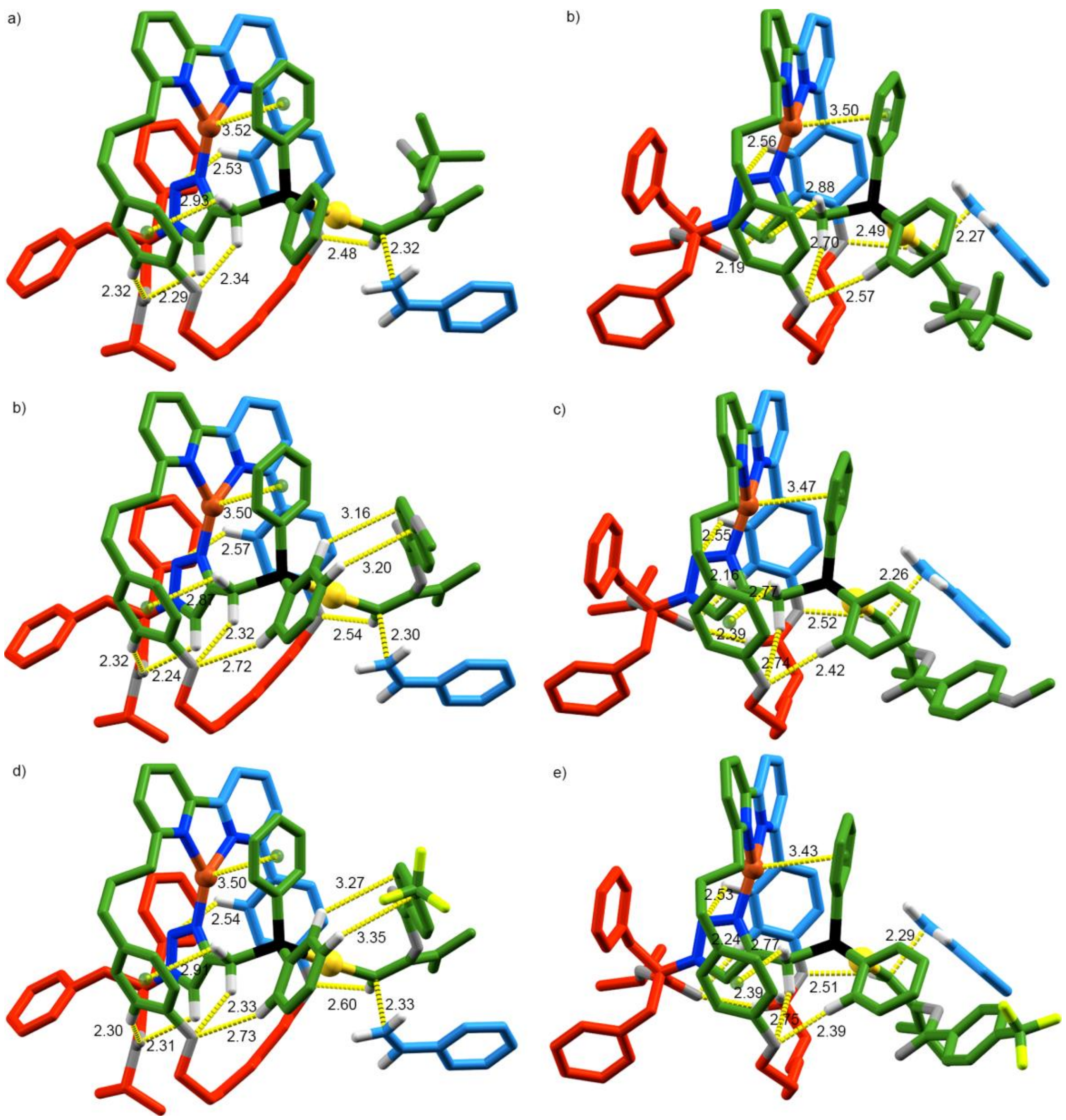

Figure S185. Transition states determined (DFT (CAM-B3LYP, 6-31G(C,H,O,P,N)/SDD(Au,Cu), PCM($CHCl_3$)) for reactions leading to cyclopropanes a) (1*R*,2*S*)-**13**, b) (1*S*,2*R*)-**13**, c) (1*R*,2*S*)-**15**, d) (1*S*,2*R*)-**15**, e) (1*R*,2*S*)-**16**, f) (1*S*,2*R*)-**16**. with selected intercomponent interactions and distances indicated. For structures see "Model_Table_S18_structures.cif" submitted as electronic supporting information.

The results obtained for cyclopropanes **15** and **16** are in reasonable agreement with experiment. The fact that modelling does not predict the correct ranking of *ee* for **9**, **15**, and **16**, is not surprising, given the small difference between the experimental values (~1 kJ / mol). The large discrepancy between the experimental and modelled data for cyclopropanes **13** may indicate that, in the case of pivolyl esters, different/additional conformations of the catalyst-substrate complex may be important in product formation. Indeed, given the potential for other catalytically relevant

conformations, further detailed studies are required to assess their relevance and provide a more detailed framework for discussing the selectivity induced by the mechanically planar chiral stereogenic unit.

8. References

1. Pigorsch, A. & Kockerling, M. (2016). The Crystallization of Extended Niobium-Cluster Framework Compounds: A Novel Approach Using Ionic Liquids. *Cryst.Growth Des.* **16**, 4240–4246.

2. Schuster, E. M., Botoshansky, M. & Gandelman, M. (2008). Pincer click ligands. *Angew. Chem. Int. Ed.* **47**, 4555–4558.

3. Jinks, M. A., de Juan, A., Denis, M., Fletcher, C. J., Galli, M., Jamieson, E. M. G., Modicom, F., Zhang, Z. & Goldup, S. M. (2018). Stereoselective Synthesis of Mechanically Planar Chiral Rotaxanes. *Angew. Chem. Int. Ed.* **57**, 14806–14810.

4. Lewis, J. E. M., Bordoli, R. J., Denis, M., Fletcher, C. J., Galli, M., Neal, E. A., Rochette, E. M. & Goldup, S. M. (2016). High yielding synthesis of 2,2′-bipyridine macrocycles, versatile intermediates in the synthesis of rotaxanes. *Chem. Sci.* **7**, 3154–3161.

5. Jamieson, E. M. G., Modicom, F. & Goldup, S. M. (2018). Chirality in rotaxanes and catenanes. *Chem. Soc. Rev.* **47**, 5266–5311.

6. Johansson, M. J., Gorin, D. J., Staben, S. T. & Toste, F. D. (2005). Gold(I)-Catalyzed Stereoselective Olefin Cyclopropanation. *J. Am. Chem. Soc.* **127**, 18002–18003.

7. Sheldrick, G. M. (2015). Crystal structure refinement with SHELXL. *Acta Crystallogr. Sect. C Struct. Chem.* **71**, 3–8.

8. Wu, H., Guo, W., Daniel, S., Li, Y., Liu, C. & Zeng, Z. (2018). Fluoride-Catalyzed Esterification of Amides. *Chem. E* **24**, 3444–3447.

9. Pagar, V. V., Jadhav, A. M. & Liu, R. (2011). Gold-Catalyzed Formal [3 + 3] and [4 + 2] Cycloaddition Reactions of Nitrosobenzenes with Alkenylgold Carbenoids. *J. Am. Chem. Soc* **133**, 20728–20731.

10. Schiessl, J., Stein, P. M., Stirn, J., Emler, K., Rudolph, M., Rominger, F. & Hashmi, A. S. K. (2019). Strategic Approach on N -Oxides in Gold Catalysis – A Case Study. *Adv. Synth. Catal.* **361**, 725–738.

11. Although compound **S4** has previously been reported (Journal of Organic Chemistry USSR, 1972, vol 8, 884), we were not able to access this article and so characterised the compound in full.

12. Soderberg, B. C., Neil, S. N. O., Chisnell, A. C. & Liu, J. (2000). A [3 . 3] Sigmatropic Rearrangement of a , b -Unsaturated Fischer Chromium Carbenes : Synthesis of Alkynol and Dienol Esters. *Tetrahedron* **56**, 5037–5044.

13. Zuccarello, G., Mayans, J. G., Escofet, I., Scharnagel, D., Kirillova, M. S., Pérez-Jimeno, A. H., Calleja, P., Boothe, J. R. & Echavarren, A. M. (2019). Enantioselective Folding of Enynes by Gold(I) Catalysts with a Remote C 2 -Chiral Element. *J. Am. Chem. Soc.* **141**, 11858–11863.

14. Ref. 13 and Liang, B., Dai, M., Chen, J. & Yang, Z. (2005). Copper-free sonogashira coupling reaction with PdCl2 in water under aerobic conditions. *J. Org. Chem.* **70**, 391–393. Sanjuán, A. M., Martínez, A., García-García, P., Fernández-Rodríguez, M. A. & Sanz, R. (2013). Gold(I)-catalyzed 6-endo hydroxycyclization of 7-substituted-1,6-enynes. *Beilstein J. Org. Chem.* **9**, 2242–2249.

15. Benedetti, E., Simonneau, A., Hours, A., Amouri, H., Penoni, A., Palmisano, G., Malacria, M., Goddard, J. P. & Fensterbank, L. (2011). (Pentamethylcyclopentadienyl)iridium dichloride dimer {[IrCp*Cl 2]2}: A novel efficient catalyst for the cycloisomerizations of homopropargylic diols and N-tethered enynes. *Adv. Synth. Catal.* **353**, 1908–1912.

16. Shibata, T., Kobayashi, Y., Maekawa, S., Toshida, N. & Takagi, K. (2005). Iridium-catalyzed enantioselective cycloisomerization of nitrogen-bridged 1,6-enynes to 3-azabicylo[4.1.0]heptenes. *Tetrahedron* **61**, 9018–9024.

17. Zhang, Z., Bender, C. F. & Widenhoefer, R. A. (2007). Gold(I)-catalyzed enantioselective hydroamination of N-allenyl carbamates. *Org. Lett.* **9**, 2887–2889.

18. Pérez-Galán, P., Herrero-Gómez, E., Hog, D. T., Martin, N. J. A., Maseras, F. & Echavarren, A. M. (2011). Mechanism of the gold-catalyzed cyclopropanation of alkenes with 1,6-enynes. *Chem. Sci.* **2**, 141–149.
